# Reassociation kinetics-based approach for partial genome sequencing of the cattle tick, *Rhipicephalus (Boophilus) microplus*

**DOI:** 10.1186/1471-2164-11-374

**Published:** 2010-06-11

**Authors:** Felix D Guerrero, Paula Moolhuijzen, Daniel G Peterson, Shelby Bidwell, Elisabet Caler, Matthew Bellgard, Vishvanath M Nene, Appolinaire Djikeng

**Affiliations:** 1 USDA-ARS, Knipling-Bushland U.S. Livestock Insects Research Laboratory, 2700 Fredericksburg Rd., Kerrville, TX 78028, USA; 2 Center for Comparative Genomics, Murdoch University, South St., Perth, Western Australia, 6150, Australia; 3 Department of Plant & Soil Sciences and Life Sciences & Biotechnology Institute, Mississippi State University, 117 Dorman Hall, Box 9555, Mississippi State, MS 39762, USA; 4 The J. Craig Venter Institute, 9704 Medical Center Drive, Rockville, MD 20850, USA; 5 The BecA-ILRI Hub (Biosciences eastern and central Africa - International Livestock Research Institute), PO Box 30709, Nairobi, Kenya

## Abstract

**Background:**

The size and repetitive nature of the *Rhipicephalus microplus *genome makes obtaining a full genome sequence fiscally and technically problematic. To selectively obtain gene-enriched regions of this tick's genome, Cot filtration was performed, and Cot-filtered DNA was sequenced via 454 FLX pyrosequencing.

**Results:**

The sequenced Cot-filtered genomic DNA was assembled with an EST-based gene index of 14,586 unique entries where each EST served as a potential "seed" for scaffold formation. The new sequence assembly extended the lengths of 3,913 of the 14,586 gene index entries. Over half of the extensions corresponded to extensions of over 30 amino acids. To survey the repetitive elements in the tick genome, the complete sequences of five BAC clones were determined. Both Class I and II transposable elements were found. Comparison of the BAC and Cot filtration data indicates that Cot filtration was highly successful in filtering repetitive DNA out of the genomic DNA used in 454 sequencing.

**Conclusion:**

Cot filtration is a very useful strategy to incorporate into genome sequencing projects on organisms with large genome sizes and which contain high percentages of repetitive, difficult to assemble, genomic DNA. Combining the Cot selection approach with 454 sequencing and assembly with a pre-existing EST database as seeds resulted in extensions of 27% of the members of the EST database.

## Background

*Rhipicephalus (Boophilus) microplus*, the tropical/southern cattle tick, is a livestock ectoparasite which has negatively impacted the cattle industry throughout the world. This tick is a vector for the pathogenic organisms which cause bovine babesiosis and anaplasmosis. Moreover, heavy tick loads reduce cattle productivity and irreparably damage hides. Annual economic losses attributable to *R. microplus *infestations have been estimated in Brazil and Australia to be approximately $2 billion [1] and over $100 million [2], respectively. The cattle tick was a serious pest in the U. S. with estimated losses to the U. S. cattle industry of over $130 million in the early 1900s, equivalent to approximately $3 billion in 2009 dollars [3]. An aggressive control program led to the eradication of the cattle tick from the U. S. However, *R. microplus *has developed resistance to almost all of the chemical classes available for control programs and novel control technologies are desperately needed by producers and tick eradication programs in countries where eradication has not been possible. Also, it is imperative that cattle producers in the U. S. be proactive in preventing the very real possibility of the tick's re-establishment.

Driven by the need for novel *R. microplus *control approaches, molecular studies have been initiated in laboratories in several countries. Rosario-Cruz *et al. *[4] reported a survey of *R. microplus *tick populations in Mexico to determine the molecular mechanism of resistance to pyrethroid acaricides. Lew-Tabor *et al. *[5] comprehensively analyzed events in the *R. microplus *transcriptome during tick attachment and development, identifying specific transcripts associated with these activities. Kurscheid *et al. *[6] characterized the RNAi pathway in *R. microplus *and identified 31 RNAi-related proteins. Canales *et al. *[7] evaluated vaccination with a *Boophilus *tick protein for effectiveness as a control strategy in response to *Boophilus *tick infestations. Additionally, genomic databases and other resources have been developed which provide the foundation for a *R. microplus *genome sequencing project. For example, Ullmann *et al. *[8], using reassociation kinetics techniques, estimated the size of the genome at 7.1 × 10^9 ^bp. Wang *et al. *[9] reported comparative studies of a *R. microplus *EST database (presently updated to BmiGI Version 2.1, http://compbio.dfci.harvard.edu/tgi/cgi-bin/tgi/gimain.pl?gudb=b_microplus) which then consisted of 13,643 unique transcripts assembled from over 42,000 expressed sequence tags (ESTs). Guerrero and Nene [10] analyzed a *R. microplus *BAC library and this library was subsequently used in chromosome studies to investigate genome organization in the cattle tick [11]. *R. microplus *microarrays have been developed and utilized in studies of acaricide-inducible gene expression [12] and in profiling gene expression induced by infection of *R. microplus *with *Babesia bovis *(F. Guerrero, unpublished data). Additionally, the genome sequencing project for the black-legged tick, *Ixodes scapularis*, has been completed ([13]; http://iscapularis.vectorbase.org/index.php). The first *I. scapularis *gene set was released in December 2008 by VectorBase and GenBank and is available for downloading and browsing from the Ixodes genome project data page at VectorBase.

The large size of the *R. microplus *genome and its highly repetitive nature has precluded whole genome sequencing project with present technologies and costs. To date, *R. microplus *sequencing efforts to date have largely focused on acquisition of ESTs from various tissues and lifestages. However, EST sequencing provides information only on the coding regions expressed in a given tissue or set of tissues. Other reduced-representation sequencing techniques must be employed to obtain low-copy sequence regions (e.g., promoters, introns, and non-expressed genes) missed by EST approaches [14]. In this regard, Cot filtration was reported to be an effective protocol to include when exploring the gene space of large genome species. For example, sequencing wheat genomic DNA libraries prepared after Cot filtration resulted in a three-fold reduction in repetitive DNA and a >13-fold enrichment in genes compared to data from non-filtered genomic DNA [15]. The wheat genome is over twice the size of the genome of *R. microplus *[16] and contains over 85% repeat sequences [17]. To test the utility of Cot filtration in exploring the *R*. *microplus *genome, we used reassociation kinetics to filter cattle tick genomic DNA and sequenced six flow cells of the resulting Cot-filtered product using 454 FLX pyrosequencing. The 634 × 10^6 ^nucleotides of sequence generated was assembled into a database of contigs and singletons and the contigs analyzed by Blast. In order to gather sequence information on the *R. microplus *genome structure with a more traditional approach, we also selected five BAC clones which were sequenced to closure. Two clones were selected at random and three were selected by hybridization to known cDNAs of interest.

## Results and discussion

### BAC clone sequencing and annotation

**Figure 1:**
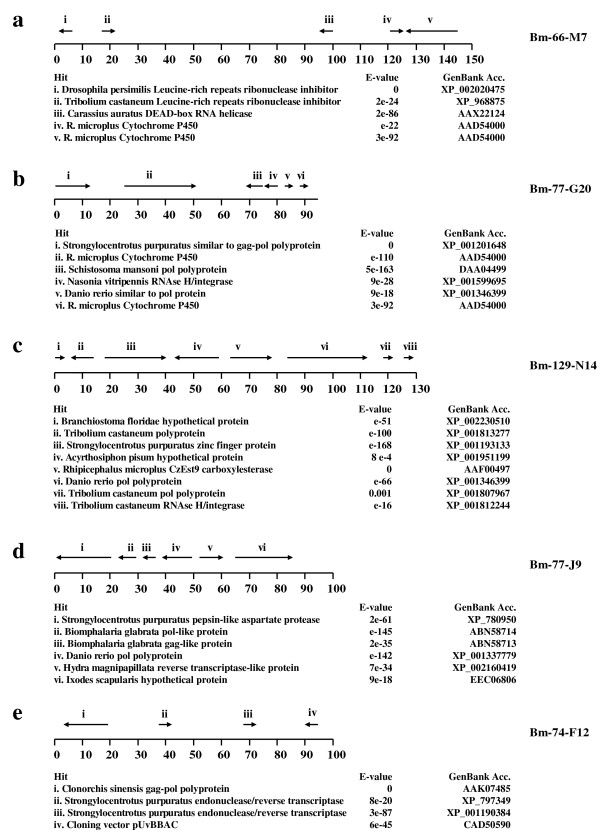
**Maps of the sequenced BACs. Each BAC is represented on a number line marked in kb**. Arrows above the number line represent where Genscan search found GenBank sequences that had significant sequence similarity (e < 0.001) to regions of each BAC. The direction of transcription (5'-3' positive direction shown as left to right) is indicated. BAC represented are a) BM-66-M7; b) BM-77-G20; c) BM-129-N14; d) BM-77-J9; and e) BM-74-F12. We also indicate the identity and e-value for the statistically significant Blast hits (e < 0.001) for the gene predictions indicated above the number line for each BAC. The complete Genscan results are in Additional Files 1 and 2.

As part of the first steps for the development of resources for complete genome sequencing and analysis of the tick *R. microplus*, a BAC library containing large insert sizes (mean insert size ≥ 118 kbp) was constructed. The library consists of 46,080 clones, providing ~0.8 X coverage of the *R. microplus *genome. To acquire basic knowledge about the genome structure of *R. microplus *and to identify entire gene coding regions for specific genes of interest from the tick, we selected five BACs for complete sequencing. BACs 77-J9 and 74-F12 were selected at random, BAC 129-N14 was selected based on its hybridization to a probe from a pyrethroid-metabolizing carboxylesterase (CzEst9; [10]), while BACs 66-M7 and 77-G20 were selected based on hybridization to a mixed probe containing equal amounts of cDNAs encoding a putative CYP41 cytochrome P450 family member [18] and a putative acetylcholinesterase AChE1 [19]. After sequencing and assembly to closure, the BAC clone insert sizes were 150,974, 95,687, 94,838, 103,645, and 126,498 bp for clones 66-M7, 74-F12, 77-G20, 77-J9, and 129-N14, respectively. The annotation of the open reading frames predicted in the BAC sequences was conducted using Genscan. The coding region for CzEst9 (GenBank Accession No. AF182283), the cDNA sequence for the cytochrome P450 (BmiGI Version 2 contig TC7171), and the coding region for AChE1 (GenBank Accession No. AJ223965.1) provided guides for BAC annotation. Figure 1 shows a diagram of each BAC with regions of significant sequence similarity to known sequences and direction of transcription indicated by arrows. Additional files 1 and 2 provide the complete results from the Genscan analysis.

The randomly selected BAC 74-F12 did not contain any protein coding sequences other than the *gag *retroviral fragment which appeared to be a full length hit to the *gag *coding region (Additional files 1 and 2) and 2 regions of similarity to partial fragments of an endonuclease/reverse transcriptase (Figure 1e). The other randomly selected BAC, 77-J9, had 3 regions of sequence similarity to parts of regions coding for *pol *or *gag *retroviral sequences and one region with similarity to the full length coding region for an *I. scapularis *hypothetical protein (Figure 1d). The lack of known tick expressed gene coding regions and the abundance of retroviral sequences from these two randomly selected BACs suggests a rather low tick expressed gene density in the *R. microplus *genome. In addition to the low known tick gene content in the two randomly selected BACs, all five BACs contained a significant level of repeats and transposons. The functionality of these transposons is not known, however, nearly full length hits to the gag-pol polyprotein nucleotide sequence were found on BACs 77-G20 and 74-F12 (Figures 1b*i *and 1e*i*; Additional file 1). The microsatellite repeats (both tandem and dispersed) identified in these BACs include (CAAT)_*n*_, (GAA)_*n*_, (GA)_*n*_, (CAT)_*n*_, (TA)_*n *_and (TGAG)_*n *_(data not shown). NUCmer analysis (discussed below) also was consistent with a highly repetitive genome. These findings are in general agreement with the previous estimation that as much as 70% of the *R. microplus *genome may be repetitive [8]. Also, Van Zee et al. [13] reported a ~95 bp repeat element in the *Ixodes scapularis *genome which is estimated to occur over 1 million times in that tick's genome. Highly repetitive genomes present difficulties in sequencing and assembly steps of whole genome sequencing projects. In fact, the highly repetitive nature of the *R. microplus *genome presented problems in the sequencing and contig assembly of a BAC not reported in this study, but selected on the basis of its similarity to a GGY protein domain-containing gene (P. Moolhuijzen, unpublished data).

The three BACs selected with specific probes were targeted because of our interest in identifying molecular mechanisms of resistance to acaricides; the esterase and cytochrome P450 family of enzymes are known to be involved in acaricide resistance in *R. microplus*, though information on specific genes involved remains limited. The BAC selected with the CzEst9 probe, 129-N14, contains eight regions with sequence similarity to GenBank entries. Three of these were *gag*- or *pol*-like retroviral sequences, one was similar to RNAse H/integrase, while two were similar to hypothetical proteins. The entire gene coding region of CzEst9 was found in 129-N14 as were sequences with similarity to a zinc finger protein. Only the CzEst9 and *Branchiostoma floridae *hypothetical protein hits appeared to be full length (Fig 1c; Additional file 1).

BACs 66-M7 and 77-G20 were both selected with the P450/AChE1 mixed probe and each contained two Genscan predicted copies of protein coding regions with sequence similarity to the *R. microplus Cyp41 *cytochrome P450 reported by Crampton *et al. *[18] (Figure 1a and 1b). The P450 genes comprise a very large gene family of enzymes that are present in most organisms and which function in many oxidative detoxification pathways, often in mechanisms whereby arthropods develop pesticide resistance [20]. We were interested in sequencing genomic DNA corresponding to the *Cyp 41*-like TC7171 from BmiGI Version 2. Crampton *et al. *[18] described *Cyp41 *as most similar to P450 families which metabolize compounds such as pesticides, and Guerrero et al. [21] found that expression of the transcript that corresponded to TC7171 was very abundant in an organophosphate resistant strain of *R. microplus *compared to an organophosphate susceptible strain. Additionally, the susceptible strain responded to organophosphate application by reducing the relative expression of TC7171 transcript compared to overall gene expression, while the resistant strain responded to organophosphate by increasing relative expression of TC7171. Thus, the discovery of these BACs with cytochrome P450-like sequences will be very helpful to our studies of metabolism-based acaricide resistance genes. BAC 77-G20 appears to contain the full length *Cyp41*-like sequence and a downstream tandemly-arranged partial copy which encodes the N-terminal part of the *Cyp41*-like protein (Fig 1b). Interestingly, the two *Cyp41*-like copies in BAC 66-M7 are arranged in a head-on fashion (Figure 1a*iv *and *v*), transcribed from different strands of the genomic DNA. It is possible the BAC assembly incorrectly oriented the sequences corresponding to Fig 1a Hit *iv *because the Genscan analysis discovered this region coded for the C-terminal region of *Cyp41*, while the Figure 1a Hit *v *sequence coded for the N-terminal and middle areas of *Cyp41 *(Additional file 1).

**Figure 2:**
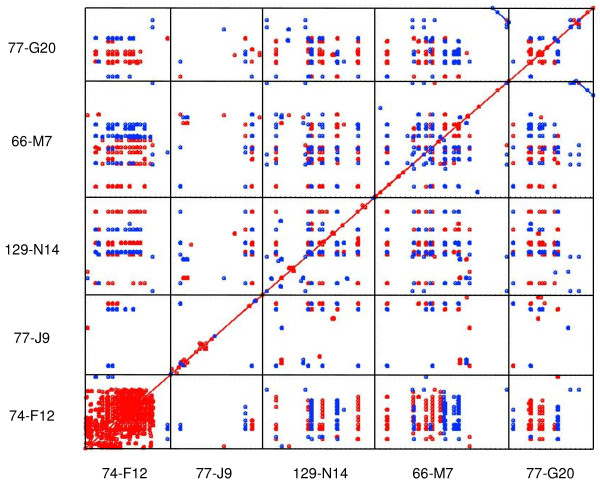
**NUCmer analysis of the BACs.NUCmer Version 3.06 was used to plot all 5 BAC sequences**. Matches unique in the reference sequence but not necessarily unique in the query are shown in blue. All other matches are shown in red.

A dot plot analysis (Figure 2) of the five sequenced BACs shows that repetitive DNA is quite common in these BACs. The most striking aspect of the plot is the highly repetitive nature of the randomly selected BAC 74-F12 with tandemly arrayed repetitive features prevalent through most of the BAC, visualized in the comparison to self. Interestingly, the other randomly selected BAC, 77-J9, did not have many matches to self, in fact, far fewer matches to self than the other random BAC or the three BACs which were selected based on their gene coding regions. The pattern of matches arranged in linear fashion in the cross-BAC comparisons is another striking feature of the dot plot, indicating how common tandemly arranged repetitive elements are in this tick's genome. In particular, the comparisons of random BAC 74-F12 to both 66-M77 and 129-N14 show numerous tandemly arrayed matches. In contrast, the other random BAC, 77-J9, which has six significant hits to GenBank entries (Figure 1), contains less dot plot matches in the cross-BAC comparisons than any other BAC. This BAC contains at least three significant GenBank hits to retroviral genes (Figure 1d) and perhaps the dissimilarity between these retroviral genes and the tick DNA from the other BACs is responsible for the lower number of cross-BAC matches in 77-J9 compared to the other four BACs. The long match at the 3' end of the cytochrome P450-containing BAC 77-G20 when compared to the other cytochrome P450-containing BAC's end, 66-M7, shows the two sequences are related but inverted. This is consistent with the assembled sequencing results which showed a cytochrome P450-like sequence at the ends of both BACs, although the transcription direction was in the opposite directions (Figure 1a Hit *v *and 1b Hit *vi*).

### 454 pyrosequencing of Cot fractionated genomic DNA

Given the size and the extent of the repeat content of the *R. microplus *genome, the Cot fractionation strategy was chosen to facilitate the generation of sequence information from the gene-containing regions of the genome. Cot fractionation was performed as previously described to enrich for single/low-copy sequences [22,23], processing the Cot-fractionated DNA for 454 sequencing. This included generating dsDNA with the Klenow fragment of DNA polymerase I and random 9-mers, then blunt-ending the reaction products with mung bean nuclease. Six 454 runs were completed using the FLX machine producing an average read length of 237 nt. Table 1 shows a summary of each 454 run, presenting number of reads and nucleotides, average read length, and number of non-redundant sequences. The total output from the 6 runs was 2,671,561 reads corresponding to 633,656,380 nucleotides. Table 2 shows the newbler assembly data summary. The 2,671,519 reads assembled into 15,221 large contigs, considered contigs of over 500 nt, making up a total of 9,801,045 nt, or ~1% of the nt from the overall 454 dataset. The average size of the large contig dataset was 643 nt and the largest contig was 1,797 nt. About 31% of the total reads remained as singletons, reads which did not assemble with any other read. Around 60% of the reads either totally or partially assembled. The total number of all contigs was 456,985 which contained 110,333,787 nt or ~18% of all nts. Thus, a large part of the nucleotides did not assemble into contigs. In fact, we found that approximately 49% of the 454 sequence reads were redundant to this assembly and did not provide new sequence information. We also used BLAT analysis and determined that 452 of the 15,221 large contigs mapped at >95% identity to 499 members of BmiGI Version 2.1 (data not shown). Interestingly, the remaining large contigs were all found to contain potential open reading frames >100 bp and Genescan analysis identified genes/exons in 2,388 of these contigs (data not shown).

**Table 1:** Summary of sequencing runs generated by 454 pyrosequencing.

Run	No. of reads	Total no. of bases	Avg. read length	No. of non-redundant seqs
1	372,821	91,115,654	244	280,859
2	417,596	101,746,015	243	279,784
3	508,619	124,597,626	245	209,344
4	390,263	89,660,849	230	212,275
5	421,619	97,803,973	232	171,554
6	560,643	128,732,263	230	218,709
Total	2,671,561	633,656,380	237	1,372,525

**Table 2:** Summary of the raw data assembly of 6 runs on 454 using newbler assembler.

Category	Quantity	Percent
Total number of reads	2,671,519	-
Total number of bases	633,429,850	-
Number of searches	2,081,652	-
Seed hits found	218,338,521	-
Overlaps found	6,694,880	3.07
Overlaps reported	4,244,624	1.94
Overlaps used	2,699,511	63.6
Number assembled	487,166	-
Number partial	1,135,496	-
Number singleton	829,693	-
Number repeat	185,527	-
Number outlier	33,637	-
**Large Contig Metrics**		
Number of contigs	15,221	-
Number of bases	9,801,045	
Average contig size	643	-
N50 contig size	624	-
Largest contig size	1,797	-
Q40 plus bases	6,689,319	68.25
Q39 minus bases	3,111,726	31.75
**All Contig Metrics**		
Number of contigs	456,985	-
Number of bases	110,333,787	-

**Figure 3:**
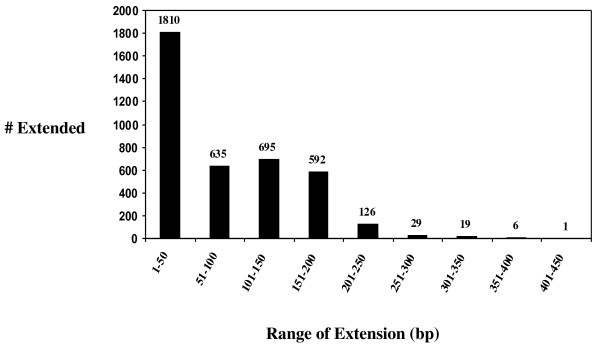
**Distribution of the BmiGI member extensions resulting from reassembly with the Cot-selected genomic DNA sequences**. The range of length of the extension is noted on the x-axis in bp and the count of the number of members of BmiGI Version 2.1 that were extended within a specific range is noted on the y-axis.

To further the utility of our 454 data, we sought to re-assemble the data with existing tick sequences, most of which were from BmiGI. The 1,372,525 non-redundant sequences were used to create an assembled sequence dataset using the members of BmiGI Version 2.1 Gene Index as seed sequences in an attempt to extend the BmiGI entries, particularly in the 5' direction. It has been found that the BmiGI entries are often lacking in N-terminal encoding nucleotides or the 5' untranslated regions. Also, since the BmiGI database was assembled from ESTs, 5' gene promoter regions and sequences downstream from the polyadenylated 3' end are absent. It was thought these deficiencies could be partially addressed by sequencing the Cot-selected genomic DNA fraction and reassembling BmiGI. The most current BmiGI dataset, Version 2.1 http://compbio.dfci.harvard.edu/tgi/cgi-bin/tgi/gimain.pl?gudb=b_microplus, contains 14,586 members with a total of 13,476,681 bp. Following reassembly with the Cot-selected 454 sequencing data, we were able to extend the length of 3,913 BmiGI members, resulting in a total dataset of 13,776,990 bp. The BmiGI Version 2.1 sequences extended by this analysis are listed in Additional file 3 titled "BmiGI CotDNA Extension IDs.xls". These sequences can be viewed at http://ccg.murdoch.edu.au/gbrowse/cgi-bin/gbrowse/tickbase/. They are also available in Additional file 4 titled "BmiGI CotDNA Extension sequences.doc". The 300,309 bp extension of the BmiGI dataset was only a 2.2% increase. Figure 3 shows that the approximately half of the extended BmiGI members were only extended from 1-50 bp. However, there were approximately 600 members which were extended in each of the three categories of 51-100 nucleotides, 101-150, and 151-200. These extensions correspond to potentially up to 33, 50, and 66 amino acids. There were also over 100 BmiGI entries which were extended between 201-450 bp. Although on a percentage basis, the extended BmiGI database does not seem much different from the original dataset, this new information would be very useful in cases where N-terminal amino acids are necessary, such as when designing full-length gene coding regions for expression studies. One interesting point from Table 2 is that the newbler assembly classified 185,527 (7%) reads as repeats. Since the genome analysis by Ullmann *et al. *[8] indicated that around 70% of the *R. microplus *genome is repetitive, the Cot selection has reduced the repetitive content 10-fold.

## Conclusions

In summary, the Cot-filtration approach provided a valuable intermediate dataset that bridges the gap between focused EST sequencing and whole genome sequencing. Our Cot-filtered DNA 454 sequencing allowed us to add from 50-450 bp onto 2,103 members of our preexisting BmiGI Version 2.1 EST database of 14,586 unique sequences. This corresponds to potentially 16-150 new amino acids added to the extended BmiGI members. Further bioinformatic analysis is required to identify promoter and intronic sequences in the Cot-filtered DNA dataset. This would include utilizing the *I. scapularis *genome sequence to align our dataset contigs and help identify genome components and elements such as putative exon/intron boundaries, 5' and 3' untranslated regions, promoters, transcription factor binding elements, regions of synteny, among others. The 454 sequencing dataset from our study is available from Genbank and it is hoped this dataset will provide a resource for genome studies of *R. microplus *specifically and ticks in general.

## Methods

### Tick strains and rearing

Ticks from the Deutch strain of *R. microplus *were reared at the USDA-ARS Cattle Fever Tick Research Laboratory in Mission, TX [24]. This strain originated from an outbreak in Webb County, TX in 2001 and has been reared in the laboratory since the outbreak was discovered and sampled. Although inbred and started from only a few individual adult ticks, the strain is not genetically homogeneous.

### Genomic DNA extraction, library construction, and BAC screening and sequencing

In our laboratory tick-rearing protocols, at each generation approximately 50 engorged females that have dropped off their bovine host are collected and used to obtain ovipositional data. Eggs from this group of females are combined in a petri plate, mixed with a spatula and weighed into vials containing approximately 3 g of eggs. These vials are labeled with generation identification data and stored at -80°C. Eggs from the f7, f10, f11, and f12 generations of the *R. microplus *Deutch strain were pooled and a total of 10 g used to purify very high molecular genomic DNA following the protocol from Sambrook *et al. *[25]. Following 4 d of dialysis in 50 mM Tris, 10 mM EDTA, pH 8.0 dialysis buffer, changing buffer twice daily, 15.6 mg of genomic DNA was recovered. Analysis of a 100 ng aliquot of the DNA by 0.4% TAE agarose gel revealed that the average size of the genomic DNA was over 214 kb (data not shown). This sample was stored at 4°C for Cot enrichment experiments described below.

Approximately 2 g larvae from the f8 generation of the Deutch strain were used by Amplicon Express Inc. (Pullman, WA) to isolate genomic DNA to synthesize a BAC library of approximately 0.8× coverage. Our intention was to produce the BAC library from a Deutch strain generation that was relatively close in time to the introduction of Deutch from the field collection into laboratory rearing, but not so far removed that genetic differences from laboratory rearing might have been introduced to the genome. Egg samples from f1-f8 and adult or larval samples from f1-f7 were no longer available. Thus, we selected larvae from f8 as our best available sample for producing the BAC library. Briefly, high molecular genomic DNA plugs were prepared and *Mbo*I partial digestion used to produce compatible ends for ligation into the *Bam*HI site of the BAC vector pECBAC1. Transformants (46,080) were robotically picked and arrayed onto 384-well plates. Twenty BACs were selected at random and average BAC clone insert size was determined as 118 kb. BAC clones were either randomly selected or selected from hybridization screen with genes of interest for complete sequencing as briefly described below. Hybridization probes were synthesized using the Strip-EZ DNA Kit (Ambion, Austin, TX), labeling with α-^32^P-dATP (3000 Ci/mmol, 10 μCi/μl, Perkin Elmer, Shelton, CT). *CzEst9 *probe was synthesized using *PfuTurbo *Hotstart DNA Polymerase (Stratagene, La Jolla, CA) with gel purified primers FG-328 and FG-329 (Additional file 5) on a DNA plasmid containing the entire open reading frame of the esterase. Probes for AChE1 and the putative cytochrome P450 were synthesized from template derived from reverse transcriptase-PCR performed with the AccuScript High Fidelity RT-PCR System (Stratagene) on total RNA isolated from tick larvae using the Totally RNA Kit (Ambion). Primers from these reactions are also noted in Additional Datafile 5.

BAC sequencing was performed at the J. Craig Venter Institute (Rockville, MD). For complete BAC sequencing, a library of medium size inserts (4 - 6 kb) was constructed in pHOS2. The BAC DNA (2 μg) was sheared using a nebulizer and the resulting fragments of 4 - 6 kb were blunt-ended, ligated to adapters, and cloned into the pHOS2 vector to generate a library. An aliquot of the library was used to transform competent bacterial GC cells. From each medium size insert library, a total of 1,152 clones were randomly selected and sequenced from each end, yielding read length of 720 - 760 bp. The resulting sequence data was processed for vector removal and used to assemble complete BAC sequences. Targeted PCR amplification and sequencing was used to close sequence gaps that remained after the assembly. The BAC sequences have been submitted to GenBank with accession numbers of HM193853-HM193857 for BACs Bm-74-F12, Bm-77-J9, Bm-129-N14, Bm-66-M7, and Bm-77-G20, respectively.

### Cot-enrichment and 454 sequencing

Total *R. microplus *genomic DNA prepared as described above was processed by Cot filtration to enrich for single/low-copy and moderately repetitive DNAs. The Cot filtration was performed as previously described with some modifications [15,22,26]. As the Cot filtration protocols were described in detail in those two references, we describe briefly the overall approach related to the cattle tick. The Cot analysis performed by Ullmann et al. [8] had determined the genome size and reassociation rates for the foldback, highly repetitive, moderately repetitive and unique fractions of the cattle tick genome. Using that data in conjunction with the mathematical approach detailed in Lamoureux et al. [15], we determined a Cot cloning value of 660 M.s. At this value, 90% of the single copy DNA should remain single stranded and we aimed to isolate and clone that single stranded fraction of genomic DNA. *R. microplus *genomic DNA was sheared with a HydroShear DNA Shearing Device (GeneMachines, San Carlos, CA) to a mean fragment size of 1.5 kb. Two hundred μg of the sheared DNA was denatured at 95°C for 5 min, then quickly transferred to 70°C for 1 hr 48 min 6 sec , which is the calculated renaturation time equivalent for Cot = 660 M.s. Following the renaturation, DNA was immediately diluted 100-fold in 0.03 M NaPO_4 _prewarmed at 60°C and applied onto a hydroxyapatite column equilibrated in 0.03 M NaPO_4 _at 60°C. The non-reassociated ssDNA fraction that contains the unique fraction of the genome was eluted using 0.12 M NaPO_4_, and concentrated using a Millipore Centriplus YM-30 column, concurrently changing the buffer to 10 mM Tris (pH 8.0). The ssDNA was used to make dsDNA by second strand synthesis using DNA polymerase I Klenow Fragment (3'-5' exo^-^; New England BioLabs, Ipswich, MA) and random primers. The resulting dsDNA was digested with mung bean nuclease to remove any single strand overhangs followed by purification using a Qiagen DNA purification kit. To enrich for DNA fragments of sizes suitable for 454 FLX sequencing, 250 to 600 bp fragments were purified from the agarose gel using the Qiagen Qiaex II Kit. The gel purified material was processed for 454 sequencing using the FLX machine following established manufacturer's protocols [27]. Six FLX runs were performed using the sample prepared sample of Cot-filtered genomic DNA. This data has been deposited at DDBJ/EMBL/GenBank as Whole Genome Shotgun project under the accession ADMZ00000000. The version described in this paper is the first version, ADMZ01000000.

### Sequence Data Analysis

The fasta and quality files were extracted from the 454 reads (unpaired) in the Standard Flowgram Format (SFF) files using the python script 'sff_extract.py' and the random first 9-mer and 4 tcag clipped. Mira V2.9.43 [28] was used to assemble the 454 reads against an existing sequence backbone of the BmiGI Version 2.1 gene index [9,29]. Mira options were selected for an accurate highly repetitive 454 assembly. The 454 reads were mapped to the assembled BAC sequences using Maq Version: 0.7.0 alignment tool (http://maq.sourceforge.net/maq-man.shtml#intro) and the Newbler assembler Software Release: 2.0.00.20 [30]. The alignments were visualized using BioPerl scripts [31]. For our BAC study, BLAST hits having e-values below 0.001 were considered as significant hits. NUCmer version 3.06 at default settings [32] was used to visualize the repetitive nature of the BACs. Genscan was utilized to identify open reading frame matches to GenBank entries [33].

## Authors' contributions

FG participated in design and coordination of the study, prepared Cot-filtered DNA, and drafted the manuscript. PA led bioinformatic analysis of BAC and 454 data, prepared figures, and helped revise the manuscript. DP helped design the study, prepare the Cot-filtered DNA, and revise the manuscript. SB, EC, and AD helped with coordination of the study, bioinformatic analysis of the BAC and 454 data, and drafting the manuscript. MB helped with bioinformatic analysis of the data. VN conceived the study and helped coordinate the data collection and bioinformatic analysis. All authors read and approved the final manuscript.

## Supplementary Material

Additional file 1 **Complete Genscan Analysis of 5 BACs**. Genscan was used to identify open reading frame matches within each BAC to entries in Genbank. An Excel file with hit descriptions, e-values, reading frames, and start-stop locations noted. Click here for file

Additional file 2 **Diagrammatic representations of Genscan analysis of BACs**. Complete Genscan analysis with hits, putative identity, and e-values mapped onto the 5 BAC sequenced to completion and reported in this study. Click here for file

Additional file 3 **Expressed gene sequences extended by Cot-selection**. The BmiGI Version 2.1 entries that were extended by our Cot-selection approach are listed in this Excel file. Click here for file

Additional file 4 **Expressed gene sequences identified in Additional file **3. This Word document file contains the BmiGI Version 2.1 sequences that were identified in Additional file 3 as extended by our Cot-selection approach. Click here for file

Additional file 5 **Primers for BAC probe synthesis**. This Word document contains the sequences to primers used to synthesize probes to screen BAC library for BACs containing sequences from CzEst9, AChE1, and TC7171. Click here for file
