# Supplementary figures and images for: Reassociation kinetics-based approach for partial genome sequencing of the cattle tick, Rhipicephalus (Boophilus) microplus
Source: BMC Genomics. 2010 Jun 11;11:374. doi: 10.1186/1471-2164-11-374 (PMC2893602; doi:10.1186/1471-2164-11-374)

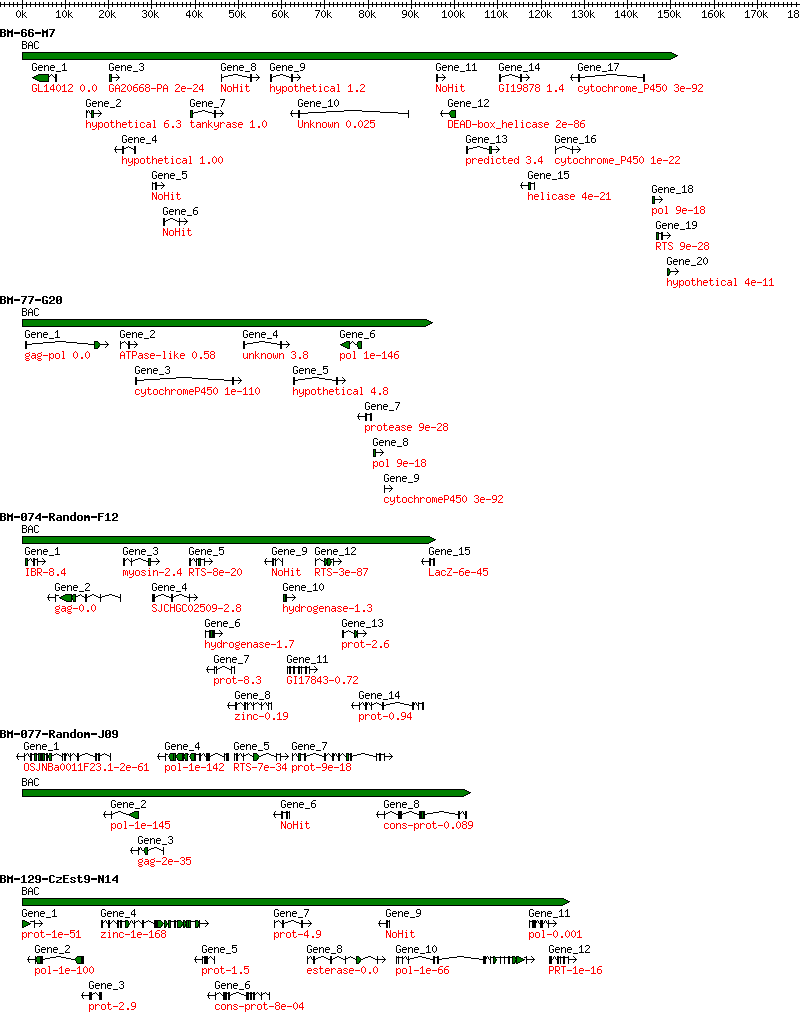

Supplement: Additional file 2 — Diagrammatic representations of Genscan analysis of BACs. Complete Genscan analysis with hits, putative identity, and e-values mapped onto the 5 BAC sequenced to completion and reported in this study. [file 1471-2164-11-374-S2.PNG]
