# Supplementary material for: Reassociation kinetics-based approach for partial genome sequencing of the cattle tick, Rhipicephalus (Boophilus) microplus
Source: BMC Genomics. 2010 Jun 11;11:374. doi: 10.1186/1471-2164-11-374 (PMC2893602; doi:10.1186/1471-2164-11-374)
Supplement: Additional file 4 — Expressed gene sequences identified in Additional file 3. This Word document file contains the BmiGI Version 2.1 sequences that were identified in Additional file 3 as extended by our Cot-selection approach. [file 1471-2164-11-374-S4.DOC]

>CK172636_bb No definition line found

GGGGGTACCCCTGCCCAAAAAAGCAAAAAATGCATGTACACAACTACCGTCTCGTGTACAATATTCTGGGGGCCGAGGTT

TTGTGAACCCAATGAAAACAAACCTCTGAAACCGGCGCCACACAATCTTTACACAGGGAAAAAGTCTCTCTTCCCCTTTG

CCAAATGGTAAAACTGTATATATGCCCCTGTAAAACCCAAAAACCCTGGGCCCAGTTTTTTACCCAAAAACACGACTACA

AAATCTTCATCCGGCTCCGTTCTGTTGAATGAAGGCAACATACACTAAACCAGTGGTAAACACAAAAAAACTCTGAGTAG

TGTCAATACAAATAAAAAACATCTTACAATCAAACTGCCTTCAAATCTAGTGGTAACTATTATGGGTGCAACGTTGCCTT

CATGTTGCTTCAATTTCAAGGTTGTGCCMAAGCGCAGGCTGCTAGGGAAGGAACATCTTACAACAAAACTGGCTTCAGCT

CTAGGCACTATGTTGTACGACAATGTTCAGGGTGGAAATAAAGATTATCACAGGGCTCTTCTTATAGCACAGCTTAATGT

TAATTATCTCTGCATGCTTTCATGAAAAAAAAAACATTTTTTTTCTTCGCACCAAAGGTGCCACCTGCACATAGGTTGCA

CAGAATGGCTAAAA

>CK172739_bb No definition line found

TTCACTCCGGGTACCTGGCACTACGTGATGTGAACCTCCGTTACCTCTCGCCCATCGGCTCTAAGGCAGTGATGCGCACA

CGATCCAACTCCGTGACACGTGACGAGCTGTTCTCTCTTGAAGACTCTGTGCCGCAGGCGGCCTTTGTAGGGTTCAACGG

AAAGTACGTCTCTGTCAAGCAAGGTGTTGATGTCACTGCCAACCAGGACGAAGTGTCGGACCACGAGACGTTCCAGCTAG

AGTGGGACAAGGAAACTGGCCGCTGGTTTGTCCGCACTATGCAGGACAAGTACTGGTCGCTCGAGAGCTCGTCTGGCATT

CAGGCCAATGCCGACAAAGGCTCTGCCAACAGCCTTTTTGAGCTTTGCTGGCAGCCTGACGGCAGTGTCACCCTGGTTGC

CAGCAATGGCAAGTTGGTTGGTGCCAAGAAGAGCGGCCACTTGTTCGCCAACTGTGAGCCTGGCGACCCCGCTGCAAAGT

TTCACTTTGCACTGGTCAACCGCCCCGTGCTTGTCCTGAGGTGCGACCAGGGCTTCGTTGGCCGCAAGGGGCCCTCTTCG

CCGAGACTGGAGTGCAACAGAGCCAGCTACGAGATCGTTCATGTGGAGCGTGCTGACCGTGGCGTTTGTCACCTCAAAGG

TAATAATGGCAAGTACTGGGGCATCGCTGAAGATGGCTCCATTTCAGTAGATTCGGACGACACCTGCGGCTTCTACGTTG

AGCTGCGCGAACCTTCACGCCTTTGCTTGAAGACTGCCGACGGTTCTTATCTCAATGCTGACAAGAACGGCGCTTTCAAG

GCTGGCGCTGCCGACCCAAGCCAAGCAACACTTTGGGAGTACTGAGCTGCCTAGACCGTCTGGCTACGGAAATCAATCGG

GCATGGCTCGACTYRGATCAACACAGCAGCATGTYTCGCTCCATAATTTGCTTTGATGTTCCTTAGGACGTTATTTCCTA

GGCTGAAACCATCCCTTAAAATTTT

>CK172817_bb No definition line found

GCACAAAGAACAAGAAACCACGCCAAAATNAGGCCGTTACAGTGGGATTCATCTCTGCCTAAAATCATGCACCACTCGGT

ATGTTCACCACACTGCTGCTGTATTTATTCTCTTGTGCAGACACCACAAACGWCCTTTGCGCARTCTCCATTAGGAATAT

GTCGCGAATTTATTGTCCCCTATCCCC

>CK172933_bb No definition line found

GTTGCACAAGTTTGCTCACGTCTCGCATTATGGTTGTTGCGTACAARTGTGGCCACTAGGATGGGCCGAGAGACTACGTT

ACCTGTTTTTCAAAGCTGTCTAAGTTGAGAGACGGTTTAAGAATGGCCATAGTACTGTGAAAGTAACTGTGAAGWWTTAC

TGTAAAAATGTTTCTGTTGCGCAATATAAAGCACTGTGATCCAGCATATCTGGTGGGATAAAGCTRTCGGTCTTGCACAG

TCTTTCAGTTTTCTTTRGCACCTGCKGCGTAACTTCATCTATTATATCTGCCAACCAGTGTATGTACAATAAGGTGGCCT

TGCCTAGCTGTAGCTACCACTCCAAAGTAATTAATCTACAAATGCAGGTGGTACCCAGATGGTGATGTAAAAGATCAATG

TGCTGTGTGTTTTCCTCTGAATTTTTAAATGTCCTTTGGCATGTATTTACGCAGCTCAGTTATGTGTTTAATGTTCTTCA

CTGTACACACTAAGGTCAGTTCAGGGTGCTGCAATGCTTTTTTGCTTGAATGCTCAAGTCCTAGAGGCCAAAGTTAGTGT

GTGTTTTTAAGCATCTAGAGAGTGCATTTGTCATTGGCACGTCTTCAGTGTGCACCAAGCTGTGGTGCTGCGTTCAACAT

TCTAGTGCAAATGTACATATACCTAGAATAGTGGTTTCAACTAACCATTATCATGTGAGTTGTATTTAACTATGTCATTG

TATTCATTGTAATAAAAGGAAATGTGATAGACTGT

>CK173045_bb No definition line found

CTATAATTCTTTTTTTAGGACAAAGCCAATGGGGTGGTGTTTGAAATGTAGCAAACAAATTTTTGAAATTTCTTTCGAGC

AAATTTTAAATTTGCTTTATTTCTGGGTGACTGGTGGCACTATTTCAAGTTGCTCAAGTTCAGTTAGCTGTAAAATCTCT

TACAAGCAATTAATCAAAAAACTGCTTGCTATGTTGCAATCACCAGTGTTTCTAGTATCTGACATATGCAAATTATAGTT

TTTTGATGAATACTTTTTTTTCTATTGTTACGGAAAAACATGGCGGATTACCTCTTATTATCTTGAGAACTAATTGGCTG

ATGGAAAAAATAATTGCATATTTCAAATCAGCAAAAAAAAAGCAACAAGACTGGCAAGTTTCGTTAAGTTTGCTTAAAAA

GTAAGCGAAATTGTTTCAAGACCCATGTCCCCCCTTAATTGTCCTTTTTCCTGTGTTTGAAAAGCAAGCGATACACGTGC

GAATGTGTCCTTCAATTCGTGGAAAAATGCGGCAACGTCTGTGTTCTGTTCCAGTGTCGTGTCCAGTGCCACAAATTCCA

TTGACAAGCGCCAGCGGTTTATCTGCTCGACAAACCAGGCTGTTGTCTCCGCTTCCTTCGGAAGAAGCTCGACAGACCGC

AGGCAGCGCAGCCCCGTCACTGTTGCGCAGTTCAAAACCGCGCCAGATACGCTCGCGTCTTTTCGCGTATATTCGGGGCT

TAGGCAATTTAGTTCGACAGCACGCAAAAGT

>CK173153_bb No definition line found

ACGTACAGGGCACTTCAGGCCATAATCTTTAATTTTCTGCTTCAATTCATCTTGATGCAATCAATGCAAGCATCTGCAGC

AACGCTGTAAAGACAGGACAGACAATGAAGAACTGCAACCGAGTTATTCCTACACATAAACGATTCAGCACGCTCGTGGG

AGGGGGTAGGGTTGTAAAAAAAATATATTAGTGCACTTTGCATGTACAAACAGTGGGGAACAAAGGGACGACACTACGGG

GTATACAATGACATGGAAAAAATGAAAGAGGGAACGACATGAGAAGCGTACGGTAACTGGACAGAAAAAACAGAGCACAG

GTGGAAACGAAGCTTCAGCACTTAATTAGCACAAGCAAAGTACCGTCTCTAACGGTTCGCCATACTCGAAGAAAAGTGGG

CAGTGATTAAAATGCAAGGATAAAATTGCTGAATGGGAACTGCATGCAGCTATGTGGACACATTACTCTCAGAGTACTGT

AGTGAACAAAAGTTCCTAGGACTCCATAGATCACCTTGTGAGCTCGATGCAGTAAACCAGAAAGGTAAAGCCAGTCAAAT

AGAATGCAGAGCAAAGAGAAGGGCTCGTCTGTTCTTCTGCCTCTCGTGTCCTGCACAACCATTGCTGCTCCATCAAAAAT

ATTTGTAGCACACAACAGCTGAGCCCTCCTCCTTTCTTAGAATGAACTGGCTGTCACCACTGATTGGCTGAAACGATTCC

TAGCCTTCCTTTTGCACCGCACGAAGTTACAAGACAGAATAGAGGCGCATCCAAAAGAGCACCCGCCCCCATTTTACGTT

GTCACTGGCGTGTCCTAAGCCCTTCTGTCCATTTCTGCCACGTAATGCT

>CK173241_bb No definition line found

TCACTTAACTTAAACTTTTATTCTTAATAGTTTCAAGAAACCACAAAACCGAAAGAAAAACATCACGTATACAGAACGTT

ACAACAAATGAGCGCAACAAGTTTCGCACTATGATGTCTGGCATAGATTGCACAGGTAAGACTAACAAAAGATTCAACAG

CTCGTGCAGCCAGAAAAGATGGGAATTCTTCTGGTCGTAATGTTCACGCATTATTGGGCACGTTGTCATCTCACGGCGTT

ACATTTTGTGCCGACACCTTCCAACAATCCAAAAAAATGGTTCTGATGTAACAATAAGAAAAGTTGAAGGCAGAAACTAC

ACCATTGTTTTTGATTAATGCAAAGGTGTCAGTGCAGCGGTCCTATGTACATGGGGACGCGGCCAAMTGGTTTCAGAATC

ATTAGAATGAAAGTGATAGATCATTGAGAAATGAAACGAGGATGGTTTGGGGCTGGAAGATTGGTGGGGTAAAGGCGCAG

GGCATAGAAAAATAACGTCTCCAGGATAGTCGTCAAACCTATAGGATCAACGACGCGACAAGGTYGYCAGACGACTTCCT

GGCCACACCGTGATGAACCAACTACAWRCAATCYCAGCAACGCCTGTTGGCGTACTTSACTTGTTCTTTTTTTTTGCTGC

TTCTGTTCGCCACATTTTCCCCCATTCTGCACTCTCCATTTCATTGTTCCATTTCTTCGCAATTGGCTGCAGCTCCTTGC

ACATGGGGCTTTGAGCCGCTTTACATTGGCACGCCCTCCTTACTCGCCATCGGTTGTTGAGCTTGAGGTCAC

>CK173242_bb No definition line found

GAAGAAGTTGCCTGGCAGTTCGACGGCGCCGAGTTACAGGCATCTGAAAATGTTATCGTCTCAAAGAACTTCCTGGTGAT

TCAGCGGGTGCAGACATACCACTCGGGCCTGTACTCTTGCAGTGCTGAGAACTCGGAAGGAAGGACTCAGGGCGAGACAC

TACAACTTCGTGTGCAACACGCGCCACTGTGCAAGGCAAACCAACACGTTGTGTACGCTGCGTCACGGCATCAGGAGGTC

GAGGTGCATTGCGAAGTGGAAGCGGATCCCGGAAATGTCACCTTCGAGTGGCGTTTCAACAGCACACTTCAGCAACGGCC

CCTAAAGAGCTTCACGGCGCAAGGCACGAGTAGTGTTGCCCGGTACATCCCCCACAGCCACACTGAGTACGGGACCCTTC

TCTGCACAGCCTCTAATCGCATCGGGAAGCAACGGCAACCATGTCTATTCCACGTCGTTCAGGCAGGACCCCCGGGCCCT

GTGGAGAACTGTTCCATCACCAACCAAACTGAGGGATCACTGCACCTCGAGTGCCAGGCAGGATCAGATGGCCATCTTCC

GGCGCACTTCGTACTCTCCGTGCATGACGCACTTACAGCCACAGTGTCGGCAAACTTCACCGCCGACAAGCCAGATTTCT

GGGTCCACGATCTGCTTCCTGGTGTGGAATACTTTCTTGTGGTCTCTGCCGTCAACGAGCGCGGCCGAAGTCCAGAGCTC

ACAATCCTGCCTCCAATCATGCCTCTCGTGGGAAAGCTGACAAAGTCCGGAAGTGCAGCCAAGATTTACTCGAGTGCACT

CCTAGTGACGGTGATTGCCGCTGTGGCGCTTCTGTGCATCCTGCCTCTCATCGTGCTGGGAGCYGTTAAACTGAAAGGGC

GACGCTTCTTCAACAAGAGCYSRGWGCTCGTTTTACATTTTATTTGGCGCTGTGGCCAAGCCTAGTTCTATTTTTAGTAC

TTGA

>CK173245_bb No definition line found

GCTTGAAAATGGCCCCGTCAAATCTGGTGGCATGCCTGGCGCTTGCAACTATGGCAGCTGGTGGCTTTCTACCGGGTCGC

CAGGAACCTGTTGACTTCGTTAGGGTGGTACAAGTGTTCCCACGAGCCGTGACCGTCTACACCTCAACCAACGACACCGT

TATGCTGTGCACAACTGCCACTCGTTCATACATGGATCTTGAGCAGAAGAAGGCCGGCTACGTCTGGGAACTGAAAGGGC

ACGGTGGTTCTTCCAAAAAAAACATTACTGTCAATTGGCAAGAAGGAGATGCCCCTGACCGGCCCACCTACTACATCGAC

GACGATCTGAAGCATTATTACGTTGGCCATGGCCTCTATACCGACTACGACACATGTTTGGTCCTGAAAATGTTTGTCCT

TGACTWTBAWSAGTGCATGCTGTGGGTAAAGCCCGAAGTCGTTGATGAAATTCCTCGACAYTGCCGTCAAAACTACRAAC

GGTACTGYCACGTCAGACAMGCGACGTACAACCGAGAACTGTGCGAGGACAACCAATAAAGACRAAAATGCCGTCCAAGC

TGAACTTGAGTGTGCCTGAAAGTGTATTACGGTCGTTGTTGACGAGCATTTTATACAGGCCTAACTGCCGTGACGTCTGC

CTTGTTCTCYTCGTCCCCATTCACTCGCACCCTTCTCGGTTGTACGTCGCTTGTCTGACG

>CK173248_bb No definition line found

ATATTTGGCATAGGTAAATATGTAAACAACAACATGCTTATAGTACAATATTTACAGCACTGATACACTATGCAACAAGA

AAAGCAGGTCTGCGAATAAAAACTAATACGAGGAAACAGCTGTTAAAAGCTGCAAGATGGTACGTAAAACAATAAATTGT

GCCTGACTGTGATGTTCCGGAAAAAAAAAAAAAAAAAACACGAGGAGGGCTGTGCCTTTAAAAAATATTARTATCTTTAA

AAAAAAAAATAAATGAATTCAAARACAAAAACTGCCCAACAACACAAAGCCMCACTGCTGGCTTTGTCAAACACAACAAT

AAAAACACAGGAACSCATAGTCTATGCAAGAGTATTAGCCCCACAGAATTGCCACAGAGGCATTCAAAAGCAAATCCAAA

TGGTCTATCATTTCTCGTAGCGGAGCTGAAAAGGAATGAATATTTCTGAGAA

>CK173249_bb No definition line found

CTCGTCGCATCACATGTGGTTTTTTTGGGGTTTCCGGCATCTAGACCCTTGTTCACAGATAAATGATTCCATTTCTACGG

TTATTTTCTATCGCAACATCCACAGCACAAACCTTATGAGGGAGCCACAACAATGCATCATGACAGTTCCACAGACAACT

CTGACATCATTTCCCATCATGTCAACTAATGGCTGTCGTTTGCTTCTGTTTCCTAAGAAGGGGATATTACAAAGCGTCGG

CTGTGTCTGAGCCTACGGGGTTGTCGTTCATGCTCAAGATCACTAGGTAGGGGCACCATAGTCAGCTGCTCATTTGTCGC

ATCTGAATTAATCCCTCCTTGCTCATGTCGTATCATTCCGCTGATGTATACTCAATCTGAGCAAAGGCAGGTTAGTAGGC

GCATGAATTTTTATTGTCTTTTTTTTTTTACGGATATGGCACCCTTTTCTTGTCTTTCTTCTTTTCTTCATGGAACTGGC

CTCTTTGTTACTGCTTTTTGCGTGCACCAGTTATGACTGCTTGCAGCATAAGTTGTGCAATGTACTGCACATGTTCCCAG

GGCAGCTGAGCTATGCTCTTGTTCCTCCCTTGCGCATAGTGAGGACGTTGGTGTGGCTGCACTATTGCCATTCATTTTCT

CCTGCGTATCCCTCTTTCCTCCTGCGCCCTCCATGTTTCGACTTTAGTGGGCACCACTGGTGCTGAATCGAGGTTGTCCT

CTACACAGTGGGATATGCATATTGGGGGCCAGGCTGTACAGCTTGTGTAGTGGGCACTGCAGTATTTGTTTGCGGCACAT

TTTGCTCGGAAGTGATGTATTCGGGACTTGGGACGGGGTGTCCAGGCATGCCTGATGCTGTGTGGCCTATCTCTCACTTG

CCATTTTGCAGTCGCTATCGCGAATGCTGAACCCCTTTGTGCATTTTGTGAGGGGGTGAGCGTGAAGGCTCTGGGTTGCC

TGTGGAGTTTCCTT

>CK173252_bb No definition line found

TAACATTCATAAAGCTTTAATGTTTAAAGATTCAGTCCCCATCTCCTTCTGCACACTCACAGAGTGCAGCAGCCCAACTG

CTTTTAAGTGCAAGCCATGTGTAGCATAACTGGACAACTCGTTGGGTCTTTTAGACATTCAGGCACAACCTCAACTTTTG

TTGAAACCACGAAAGTGAAAATGAACTTTGCTCTTTCAGTGAGCGTGATATGTACTGCGAATGTCGCAAACACACCGTAT

GGAGCTTTTCGTTAGCAGACAATGCGTCTTTACATGGCTCCAATGTAACAACTTTGCAAGGCCCTGAACACTTTTCGAAC

AAGCTTGAATTTGCCTTAACAATCTAGTGGTGATCGAACGTCAGCTTCATTCAACTAGGAMRTTATCTGTTATTTGCGTG

AYTCGAGTCTGCATCCACATCACCTAAATATWAAAAGACATGAGAGTCCTYTTCCTGCTAGCTGTGAACGTCGGCTCTCT

ATTACCTGTGTGCAATCTCAAGTAACAAAACAAAAAAGAAGCGCACAGAAAATCACACATCGCACAGTAAAAAAATTGTC

ATTATCAACATTTACATTTCAAAGCAACCCCCCCCCCCCCTACCACATACACACACACACAGGTCAACAATTTATGATCA

ACAAGCCTCGACTACCGCCTTAAAGGCACAAATTTCGCACATATGAATTATGAATATCAATGCCAACAACACGAGCTCTT

TCAAAAAGCAACATGTCTAATGAACACTGCAGTGGA

>CK173314_bb No definition line found

GGTGTGGCCGTTGAAACCGCGAATTACTACTCGTATCTAAATACTTTACCGTAACTGACGTGGAAAGTACCTCCCTACTT

GAGTGTCAGTACGTGTAGAGGATAGGTCAGTTATGACCAAGGCCCGTGCTCATCTTGGCCACACCCTCGAAGATGTTTCC

AGCCAGAACGATTTGGGTATCGTACGCGCTGCACACGAATGGGTGGCAGCCGGYGCGGGACGCTGGTTGCTCCACCTTCC

ATCTTCATTAAAAACATTGTACATTCCTGAGGCAGTCAGATCGGACTCATGCGCGGTCGACGCGGTACACTTGGCGACTT

CACTTAATGGTACATGGGATGTAAATGCTCAATTCACATAAATGACACTTCGTC

>CK173449_bb No definition line found

AAATAAGACGCCTGCAATGAGATGCCAGAAAATTAGATGCTAATACTAAATTACAAAAAGGTCTACAAAAGCCACTCCTC

CAGCTTACGCTGTAACTGTGCTGTCCGTTCCGAGCGTCTGGTGTTTTTTTTTCTRTGGAACTATACTCAGCTCYTTATGT

ACATTTWCGCCAAGAAGAAGCRCTACAAAGAAWTAGGAAAGCGAAAGARCTTCCACAAGRGAWAWCTGGACAGTACAATT

TCGGAGCKTTTACTATGTGGTTGCCAAGCATATTCCTTTCCTACACCACGACAAGTGTATTGAACATATCATTTATTATT

ATTATTTATTTACAGTTCATCCTTCAACCCCACCTATTTATTGACCGCCCCACCACAGAGGTCTAGTGGCTAAGGTATAC

TCGGCTGCTGAACCGCAGGTCGCGGGATCGAATTCCGGCTGCGGCGGCTGCATTTTCAATGGAGGCGGAAATCTTGTAGG

CCCGTGTGCTCAGATTTGGGTGCACGTTAAAGAACCTCAGGTGGTGGTAATTTTCGGAGCCCTCCACTACGGCATCTCTT

ATAATCATACGGTGGTTTTGAGACGTTAAACCCCACATATATATCAACCTATTTATTGTCCACCAGAAGTAATTGCGTAA

TCTACACGAAAATACGTGCGCACGATGCGGTCAAGTATACAAAAATATTTCAGGAGATATGCTTTCACCATTGAAATCAT

TCAAATTGTACGAGCTCTGTTATGTCTTTTCAATACAT

>CK173464_bb No definition line found

TTTTTGGTACACGTTTTTAGTAGCCCCTAATAGATGCTTATTATACCAGAAAAAGTTATACTTTTACTTGCTTATTTTCA

TTAAATCTTATTTTCATTTTGCCAATTGTAACCCTTGAAGCTGGTGGAAATAACTGCTAACCATGGTTCTGCCAACAGTC

TTGTTACCAGTCCAGCAAATGTGCTTTTGTGTGCAGGGAGAAAATGTTATTTTTCATGCGWGCATGCATGTGTGGGTRTG

TGTGTGAGAGTGAATGGTGTTTTCTTGTTACATTGAGTTCATTCACCATGCGCGGCATCTGCAAGAGCGCCAGAAGGCTC

GTGGCTGTAACCTCGGAGCCTTGCTTGTGTCGTCACTTCTGTCATTTCTTTTATACATCGCTTACTGAAATGGAGACTTA

GACAAAGGTTGTAAGCAACAAAAAAAGGCAAAGAAAAGAAAGGGAGGTACCACAAACTCCTGAAGACCTGAAATCATTTT

TTTTTCTTCTCTATTTTTTTTTCATGAATGACATGTAAAGGTTGATTTTGAGTGCCAAACGTGTGTAAAGCACGTGCAAT

GTGACGCATCGTCATGGGACGCATTGTTTCGTTATTTTCTTCTTGTACACATCTCTTTTATCCTTTTTTTTTTCACCCTT

GTACAGATGTTGTAGCTGCATTTACACAGCCATGATGGACCTTTGCTAAGTTGGAGAAAAGGTGACCTAAGTGCCTTAAC

TGCTTGTAGCACTTTTGTGTTGAAGCCACGTAATGTAAGAAAAATTATAAAGGGCCATAATAGATGAACTGTCCATTTCA

GGGACAACTGGGTGTGTCCTGCTTAAGCTCCAAGAGAATTTTTAACCATGTCCTTTCTCCCTACATGCCTTCTAGCTATC

CATAAGCTTGTTGAAACTCAATTAAGGTATGGAGTTAGCATGCCTTGCTGCTCTTGTTCACTTATTCTGCAATGTGCTAA

AGTGATCCCAGATGCAATGCA

>CK173490_bb No definition line found

ACAGTTTTGAATAACAGTGAAGTTTTAATAAAATGCAGGTTCTCTTGGGCAGGGCTGCTGCTTGTAACAAAAAAATGTCC

AACACTAATAAGCACGAATTTCGGCCGCATGTCTAGATGCTTGCAGTTTTTGCCAATGACTGTGCAATAATGATTTAAAT

AGGGCTTAGGCCAAACCAGGACCAAGGCAGATCTAGAGTGGCGTTTCTGGATTTCTTTGGAACAACTGACGATTGGGCAC

AGCGTGGCTCCAATCAATATAGTTAAACCATGAACAAGTTTTCAAAAAGTAAAAACTCATGTGAGAAATCACTACATGCT

TTCAATGGCCTCTGATAATATCCTAAAATGGGTCAAAGTTGGGCCATGGTAGTCGGCATAAATAGCACCCACAACTTTTG

ATGCCACTGGCCTTGTCATCGGGTAGTCCTCTGCTGGATGTGACCTTGCCCTCCAGACTGAACAAAACATTTCCTGTGGA

AAGAAAAAGCTTGAAGCAACAATGAAAAAGAAAAGTTGTGTCATTCTACTCCTCCATGTCAAATGGCTGCGAGCCGTCTA

TTCCTTCCAAGCTAGCAAAGTCTGAATCCTCTCGACCCATATCCATACTCTGGGAAATGGAAGACAGTGTGTCTTCAAAC

GTGAGGTCCTGCGTGTTTGACAATGACTCAAAGTCGCTGTCCTCAGA

>CK173551_bb No definition line found

CCAGGCGGCGTCCGGCAGTAACTCGCCGTGTTTCGGCCTGCYGGCCTCTGTGGCCACCAGCGGTGGCAGCGGCGGGCGCG

TCACTCCAGGMGCGGATGGGCCCAAAGTCGTGCACAACTTGTACAACTCGCCCATTCATCTCTACTCAACCCAGAACGTG

GCCGACACGCTGGCCGCACAGACGGGCCTCAAGGTCGGTCAGGGCAACCTCCAGCCATCTGGAACCGCCAGCCCTCAACA

GCAGCAGCACTCACCGCGGAGCGTGGGCAGTGCTGGCAGCGGTACAGGCATGGTCAAGCCTGGCACCAAGCTGAGTAGCG

CAGCCGACATCACCATGTCACCCACCTACCAGATGATCCATGGCGAGGAATGGCGCGACATCCGCAAAAGCGATCTCCAG

GAGCACCGGCCAGTCCAGAATCAGTTGTATGCTGTGCAGCCGGACTATGGCAATGCGATTAACGCGTTCGGCGCACCCAA

GGGACGCATCCAGCAGTCCAACAGCTTCAAGACTATCATGTCCACTGTCATGACACCAAAGCTGCTCTAGAGCAACTACC

TTTGATGTATGCTCCTGTACACGAACTCACGTTCACAGAGCCACACAAGGTTAGAGAAGGTCAATGTGAATGTGCTTTTT

TACTTTCTTTTTTTGTCCAGCAAAATGTGTGAGTCTTTACAAACTTGAGTTCGTCTACAAAACCCCGTTACAGGAGTGCC

TTTAACCTTCAAGATTGCACAAAGAAGCTGGCGAGGTCACTGTTGAAAGCCCAATGTTGTGATGCACACGAGGTCCAGAG

GCACCAAGAAACAAATGCTTACACAGTGTCCTCCAGCTGGAGAGAGGCAATTTGCAACAATTTTGTGCAGGTCCTTTCAT

TCAGGTGACGTWGTCAAA

>CK173651_bb No definition line found

AAACATCAGATTCCAAGCACAAGGACAGCAGTAGCAGTAGCAAAGACAAAGAAAGGCACAAGGACAAGCACAAAGAGCAC

AAGGATAAGGACAAGCACAAGGACAAAGAACGTGAGAAGCACAAGAGTTCATCGGAGAAGCATAGGGATAGCACTTCTCC

TGTGAAGATCAAGCAGGAAGCGGGAACTTCTAAAAAATCTGGGGCTGAAGGCATGCCCCCACCTCCAGCAAAGGTGAAGA

CTGAAGTGTCATCGCCACCCCACCCTCCGGCTCCACGGATGACCCTTGAGACCGAGACTGTAGACATCAGTGAGATCAAG

GGCGAGGTGCTTAGCGATGATGATGTGCCACTGTCTGCACGTAAGCCACAGCAAAGGCTGTCTGATACCTCATCTGTCAA

AAACAAGCGTGCTTTGGAGTCAGATTCTGAAGAAGATGAACCCCTTGCTATCAGGTCACAGAAAGCTTCCAAGAAGAGCA

AGTCTGAAGAAAAAGTCAAGAAGAAGCGGAAAGCTGACGACAGTGATTATGAAGATGATATTAAGCCTGCCAAGAAAGCA

GCAGCTTCTAAGCCTAAAAAGGTCAAAGTGGAAGTTAAAGCTGAGCCCAAAATGGAGGAGGGCAGTCCTAAGAAGAAAGG

CAAAACAAAGAAGGAAGAGGAAGTAGAAGTATGGAAATGGTGGGAAGAGGAGAAGCACAATGACGGGAAGAAATGGCACA

CACTAGAGCACAGAGGGCCAGTGTTTGCCCCAGCATATGAGCCTTTGCCAGATAATGTCCGCTTTTACTACGATGGTGTT

CCGGTCAAGCTGAGCCCCGAGGCTGAGGAGGTAGCTGGGTTTTACGGTAGGATGCTTGATCATGACTACACATCCAAGGA

TGTGTTCAACAAGAATTTCTTCCATGACTGGCGGAAGTGCATGACTTCTAAGGAAGCAGAGCTCATCACAGACCTCAAGA

AGTGCAATTTCAAAGAAATTGATGCTTACTATAAGCAGAAGTCTGAAGAACGCAAGGCCATGACCAAGGAGGAGAAACAG

GTAATGTATAGCGTTAATAAAGGATAT

>CK173703_bb No definition line found

CATCTCATCATGAATTGAATGCGAAAGTTGGCTTCGTCGCTATGCATGTGTTGTGCAGTGAAGCAAATGTTGAATCGGAA

AGGGCTGCTGTTTCTGAGCATAGTGCGGCTTCAAAAAAAAAAAGTCCCCCCCCCCCCCCGCAGCAGCTTTGCAGACACCA

CCGATTTGAGGACCACTGCCATAGAGGATTACCTGTAGCTAAGAAGAGGTGCAAGCAGCATCTGAAGCAAAGTTGTGTCA

CGCATAACGTGAGGTTACAGACTGATTTTCAAGGAAAAATAATCCCAACATATGTAGCCACATTTGGTGGGACATGGTTT

TTTTTTTTTGCCTTTTTTTTTTGTTTTCTTAGCCTAGCTAGTTGTTTCGTGTTGCTTGGTATTTAAAGTGGGCAGAGGTG

TTGCAAGTACTACATGTCGGCATTTCATGTTGGTCACTTATTTCTTGGTGCACGACTCTGTGCACCTTCAGTGGGTGCAG

GCCTCTCCTCTGAATGGTGGCATGTGTGACCAAGTACTTCTACTACGTGACACTATAGCAAAACCCAGTTATAGTGAACT

GGTTCATATACTAAAAATTGTTCGACACCCAGGTTACCTTGTGTGCTGGAGACGCCTCTTATTCATGTCTTGCACCACTT

ACATGAAAGATTCATTTGTTGCGGACAAAACTACCTACATGATTTAGTATGCAATAATTTTGGTGTGTAGCTTTCACATT

TGCAGCATCCTCGATAATAGCATTTTCAATGTGTGACATTGTATAAAATTATCGTGGGCTATTTCCTTCAACATTGGCCT

TCTCAATGCAAGACATTGCAATGAAAGCAGCAGGACATTATGATCTCAATTGAATTATCAGTTGTGTTGATCTCATTAGG

TTTGAACATGCTGTCAA

>CK173730_bb No definition line found

CAAGTCTACAAAATCTTTATTTCCATTATAAATGAACACTTACTTTCCAATTACATGTTTGTATCACATGAATGAAAGCA

GTTTCAAAGGCTTTATTATCTTGCACGTGCTCAAATCGTGCATCTTTTACATCACTTCAAGGAACATTGACAACACTTTT

GAGCACATAGCCGCTTTGAAGCACTATTTCACAATAATGGTTGTCACGATTTTTGTTGGTCAATACGTTTACATGAGATG

TTTCCCTTGCTTAGGCATAACGAATACTGCTTTTCCACGCACCTAGTTAGACTTCTCAGTAAACCTTATCTCATACAGAA

AAATTCCTAGGATGCCTGAACGGGATAGGATGTACAGTAGTCTACCAAAGAGCACTCGCTACTCTGCGTGGTAAGCTATC

CAAAGCCATTAAGTGGTTYTAACTTATCCCTCAGTAGTCTTTCATGTCTGCTTTCCAGCARAATTGATCTATGCAGTCTA

CTAGCATGCATGGTGATAACCCAAAATAGCATGCCTGCAAAAGTGTCYMARAAGTTGAGTTTTGCCAGTAATCCAATTTT

CTAYGCACTWCTTTTCGTAATTAAATATTATCCAATTAGTTAAGCCTTCCTTTCCCATGGACCCTGTCTAAAAAAACACA

CTGCGATGGCTAAATAACACGCCAACAAAATATCATACATAAACACACATTTCACAACAAGTGTTTAATAATCGCTTCGT

GCGGTCCCGTGGTGGGCCGGGGCCGTGAAGCCGAACGGTTTACGCAAGTGCGTGACGTAACCACACTGACGTCATGCGAT

GACGCACGCGACATTTTT

>CK173731_bb No definition line found

GACCTGCTTTCAGTGACGACGTCGTTGTACTTTGACGTGGTTGCTACATTTCTGCCAATTTGCCCACTCGGGTTGCGGCA

AACTTGGTGACGAGAAATACGTAACTGGTAAGTGCTCTGCGTCGACCCTCATACAATAACAGTCGGGCGTGATGTCTTTC

GTTGTGTGGTGGTATTTTTCGAYTGCTGGTTTAGAAATTAGCCGAACTTTGGAGCCCWGATTTTTGGAAACTGTGTGGAA

GATGTCTGGAGCGGTATCGCTGCAAATATGGCAGGCTGCCAGGGCTGCTATCGCGCTGAATTTAGACGCGTGCGTATCCG

TTAGGCCTAACTGTACATCGCGGCTGGTTCGATCGCGGAACCCCCGAACGCGGGTGTTCCGTCTGGTGTTTTAACGTGCG

YCTAGTCGGGCAGTTCGRTARGATCCGAGTGCGCGAAGCTGCTCTGCCGKKTTAGTAACCTCAAGTCTGCGGCTCTCCGC

AGTGGTGTGGMATGAASTTGCCGATTCGTAGTCTAGTGTTTCAAGGTGCTTGCGAAACCTTGTCGCCCGACTGTGCTGCA

TTCGATTTCTACATTGGCGACTTGCAACGGTTTGCGCGTGATACGGCGTCTGCATGTTCGTGTCTGTTTTGATAGGGCAT

CTCGCCGTCACTAGGCTATTTCAGTTTCTGTAGCCTAACGTCGTAGGTGTGCTGCGGTCATATATTGCGCGCTACTTTAG

CCCCTTCCTTTCTGTGCACTCGGTTCTAGCGCGCATTCGGCCAGCTGGCGTGGCTATTGTAAGGCAGATATACCACATTA

TTTGCAACGCAGCGCGTGTTATACGGATGCGGCCATGAAAGGTGCTGCGCCGCGCACCTGGGCCCGCAGCGGCCTTGAGC

CAAATAGTTTTGCAACGTGGCCGCGAGGTCGCTTGGCGCGTAGGCCATCTTGATGCTCTCAAGTAGATTGACCTTAGTAG

CAGTTTTAAGACGCGATAAAATACGTTCTAAGTCAGCAAAAGATTGCAAAGCTGTTAGCATGGGCCTATCGTGTTATCGG

CGTGTACGTCCGTCTTTCACTTGCGGTCGCGTTGATATACAACGCGTTTTCCGAGCGACTGAACGCGTAGAATTTTCCCT

CTGTGGTAGTCGTAA

>CK173839_bb No definition line found

TAGTAAAAAGACCGGATTTATTCATAATTTGGTTTCACCAACTGAGCTTGACAAGTGCACTGGAAAGTTTGCAATGTTGG

TCAAGCTTGGACTGCAATCCACAGTTTCCAAGTTATATTCAAGGGTGAAGAAAAAGAAGATCAACTTTACTTCAATGCAA

GATGCCTGGTGTGTTTATATCAGCTCAGGGGTGCACAGCAGTACATACCTTAACACACAGAGCTATATTCAATTGACTTA

CGTCGGCAATTCACATGGCCATCAGCGACTGATTCATGAAAAACGAAATCAGCTTTAAGTTCAAGGCAGTAAGGCGAAGC

GAAAGAGTAATAACTAATAACCTTGGACACTGGTAAATATAGAAAGAAATCAACACTTAACAACATACAAGTGAGTGGGT

AATAGTGAGGGAAAAAATAATATACGTCTCACCCAAGCACTCTCATAATGCTGCTCTCTCTGTAGACGTCGCAATGCCTG

TTTTAACAGTGCGGTGGCTTTCTTGCATGATAATTATATACAGTGAAGAAATAGAAAGCGGGCCTTTGCTGRCAAATGTA

CAACATTGTGGGCCTGCACTTTGATTTCATACAGCACAGCTTGTGCGTAACTATCTGTGTCATATATACAACTAGAACAA

GCAACTCCAGGTCGCCAGTGTTTCCATGCATCTGCTGGTAATATCCAAAATTAAGAATCGCAACAGCGAGAAGCGTAAGC

AGGAAAACTGTAAGAGGAAAAAAAAGTGAAAACAAAAGGTACACAAAAGG

>CK173843_bb No definition line found

TTTTAAAGACTCCAAGGGCTGATTGTTATTCATGACTATATACCAAGTCATATAGTGTGGATACAAATAAAAGTAACACT

GAGAAAATTTTGACAGTTTACAAAAAAAAGGCATTTTAACTCGTACTGTGGCTTTACTACTTTTGCAGTGCAACATTCTA

AGTGAAATATGGCCACCACCATATCGTGTAGTATGCTTTGAAGAAAACTTGTACACGTTTTAAAAAAGAACGGTCTGTTT

CAAGCATGAAAAATGTTTGGCTACTGAAATACTTGACAGCTGTTCAAAATTTGTCTTCCTAGCACAGTGCCCCAGTCCGG

TACTCTAGTGGATGCTTGTAACTGCTCGTCACTGTAGATTGTTCCAACTTTTACACTAAAGTAWGCAATTTAGATTAAGA

GTTCACAWMTGTGTACCACATACTGCATTTTTCAGCTTATTTATGCTTTGTAACAATCTATGGACTATTTCATGAGTTTG

TAACAATCTATGGACTATTTCATGAGTTTTGCTTAGCATAAATTACCTAATGTTCATCTTCACTGAGGCCTTACCGACCA

GCTGAAATGCTAGTAAAAACAGGGTATAAAAAAATTCTCAGTTCAATAGGTCTGACAAAATAGATACAAACAATGATGCA

CCATGTTTCACTTCTATATTGAAATTACTTAAGCGAGGAATGGCACTAGAACCAACAAGGAGACTTGCCAGTAAGG

>CK173871_bb No definition line found

ATATATGCATAAATATGTCAGTATATGCTCCCAAAAACAAGCTGCAAAGGTGGAATTGAGCTGCAGAAGAGATTATCAAG

AAGCAAATGCTCCTTCAACTCTCTGTGTACAGTGCCTGGTGTACAAGCATCTTGACAACTCCAATGATGGTGAGAAGACC

AGTCGTAWWWTCACTTGTTTCAAAAGGCTCAATTTATTTGCAAATAGGTGCCCTCTGTGAAACTTGGACGTGGATAGCTT

CACAACCTGCACTGTGCATATGTGACACTGCACTTGAACTTAATTTACACGCAACGCATACAGTARATAAAGAGGTGGAA

AAAAGAAAAGCCGCTTACTTGGAAACATTCAGCAACTGARAGAAACACAAWGGGAAAATGGTGCACCATTTCTTCTGCCC

ATCACGAATTGCACTCGTCTATAAAACACAAATGTAAGCATSMGGCCGATTAMGRMYAAACAATTGTCCTTGATGCGTAA

TACAAATCAAATATCAACGAAAGAAACGATGTTTTAAAATTATAGCGGAAGCGGGGATTGTCTTCTGACCCCGGTTCAGC

AACGTGAGAGTGGCAGGAGGCCCCTCCTTTGGCAAGTGAACTTCAGTTGAGGCAGTCTTTGCCTTTAAACTCTGCCTTGA

GTTGCTGAATTAGGTCAAAGATCTTGAGCGAAGGCCCCACTTTCATGTCGGTCAGGGCCATGACAGATTCTTTGGTCAGC

TCCAAGAGTTGTGCTCCATCAACCTTCTCACGGCTGCAGTGGGCTGCGGGCTGGCACTCACACTGTTGTCCGACCGGTTT

AGGGCACTAGCTTCAGATGACGACGTGACCATGTCATCAATATCAGGTTTGGTGTGGACGTCGTCATCGCCATAAAGATC

GTCACCGGGAGATCCACCTTTGATGGTTCCCCCACTCTTGCGGCTTCCAGGCGTGAAGCGCATCTTCTTTTTTTT

>CK173877_bb No definition line found

AAAGCTCCGTTCTACGTCGCAGCTGTGCTGGAGTAGTGCCTTCGCATGCAAGAACAGTCGAGTGAGCTTTCCCTACTGCA

CATGAGTGGGGGGTCGTTTCTTTGGCCAAGTGGTTGTGCCTGCTTCGCTTCCATGTACGAGCGGGCTCTATTCCTGGGGC

TGTTACCGTTTTCGCCACGCTGCTTCACGACGCGGTGTGACCGCTTCTACGCAGGTTGTTCTTGTCCGGTATGGGTGCAC

ACCTAGGGCGTACTCGAGATATGCGAATGGATTTAACTGTTGTTTCGCTGTTGGTCTAGTAGTCGTTGTTATTGGCGGCG

GGCGTTACTTTGTAATTGTCTTCTGTAGCATTGAGCCCTTCGTTGCTCTCGATTGATGTGCTCACACCTACAGTGAACTG

TTGTCGACACAGAGAGATTTGCTGCCGAAAACGACATGAAAGTGTAGATACAATCGAAGGTCGTAATATATATATGTATA

TGTAAACATACCGCATACACATGTTTTCAGCGTGTTCTGAGCTGACGAGCCCGTTATAAATGTGCGTGCGTAAACTCTGA

AACCAAGACAATAGTGAACGGCGAACACGCCCAGCGATAAGTGTGGCGTGGATGCATTTCCCTTTTAGGCCTATAGCTTT

TCCGCCATTCTTTTTCTCTTCACAACACTATAATGAACAAAACAAAAGAGAAAAATTGTCTCCTTGTGGGGAAGGAGGGG

GGCAYAAGAAACAACTTGTTAGCTCAATCACGTGTTCGTTCAGGAAGGCGGCCAGCCTTGCTTAACACTCTGGGAACGTT

ATTCGGCGGCCTCGCCGTGTTCCTGCCCCCTTTTTGATGGTTGGGTTGTACGTACTTTTGTTTTTCTTGCCGCGTGGCCA

TTGATGGGAGTTTTAGCTCATGCTTCAACTTTTACGGAATACCGTGGGTGTGA

>CK173882_bb No definition line found

TGGTCATATCAACATCATTTATTTACACTAAAAGACCCTTTTCAGGGTATTACTCAAGAGGTTGGGGTAAAAGCATCAAT

AACATGATTATTATTATCAATAGTAAGCAAACAAAAGAAAAAAAAGTTCGCAAAATACAGCATACATGTCTGGATGTTTA

CAGTATACAGTAAACCTACAACGAATTTTATTAAAACTGGACAAAGCATCCAAATATCCAAAAATGCAAGATTTCCAAAG

GCCTCTGACCTTTTTAGTCAAACTAAAACCAACAATGAATTTTATTCGAATTGGACGAATGTCAAAAATTGAAAAATGAA

ACGTCTTCAAAGGCTATAACCTTACTTTTTTGGTCCTAACAAAACCCACAATGAAACTTTTACGATTGGACAGCACATTG

ATATAACCAAGAAATAGGATGTGTTTAAATGGTATTCATAGGCTTACAATTTAAGTTAAATTAACACCTATAATGAATTC

ACATTGAAGCACAATATTTTTATATATATGGAACACAAAATGAAGTCCCAAAATATGACTGCCCTATAATTTTTTTACAC

CCCTAGGAGGTATAATGRAAAAATAAGTCTGAGCTAGTTTASAGRCCATTAAATGGAAAAGAACAGTTGAAAATTCTTTG

TCTGAGGAAACGAMTGACTCKTGCTAGAAGGGTATGTGAAAGGCAAGTAATAAGTTTTGTAAACARTAAGTGTTGTTTAA

ATAACGTTCTTTCAGAAACWTTTCRWGCTATAAAATCCTGCTTCGTAAGTCAGCACATCATTGGCGTATTTAAGACCACC

CTCCTACGAGAACAGCTTGCAAAC

>CK173901_bb No definition line found

CAGAAGTTTCATTTTTTTTTCTTGTCTTACTTCATTGCTCATGGCAGCTTTCACAAGTCTCTTCTGAATGGGGTATTTGG

TAAAGGGCCAAGAATAATTTTTGCTTGGCTTGCGCAGGGTCAATGTCTGTGTCAGCAACTCAAAGAAGTCGAAGGTAGAA

GCTGTGAGAGCCAAGGCTAAGGTAGCGCCAGGGTCAAGACCAGCATTGCGTCCAAAGAATGTTGAACAAGACCTCATGGT

TTCAAAGCTGTCAACAAAGCCACCCCAAAAGAAGCTTGCCAAGACTCCAGCGTTGAGGGATGTGTCAAAAAGGCTAACGG

TCGCCATGTCTCCAGCCTTTGCACTCAAGTTGCGAAGCGAAACATTGAAAAAAAGAAAGGAAGCGGAGCAGAAAGCAAAT

GGACCTACTGGAAGCAGCGCAGAAGCAGAAAGTACTGCCGGTACCATGAAAACAAAACCTCGTCGAAAAGGTGCAACAGC

AATGGGAAAGTCAGAAACATTAAAAAACTACACTAAATCAAAGGCTGATGAAAGTGTTTCTGGTGCAACGACCAGTCAGT

CTACTTCATCAAAGAATGTCAGCCAGAAAGGAAACAGCGCTGTTAACAGTCACAAGAAGGGAAAAACTCTGACTTCTGCG

GCTTCAAAGGAGAAATCTGGAACCAAAGGAGGTAGTGCAAAGAGGAAGAAGCCTTTGGCAGCAGCCAAGATGACTCGTAG

GGCATCCTCATCACACCATGTCAGCACTGTGATAAAGCCGCATTTGCAAGGGATTAGCGAAAAGCTGTCGCAACCTGTCA

AGACCTTCCGAAGTGGAAAAGTACAGCTCTAACGCCCCATTAAGATGAATGCACAGGATCGCCCACAGGAAAAAATGAAA

GATAAGAAACCTGTAAAGATAGCTGCTACACAGAGCTCATCAGGCTCTGAGACAGAAAAGCCGACTCATTTCAAGGCAAA

GCCTGCACCAGACAGGAGCCGTGTCTTCCGTCTGCGAAACATGTCGGCACCAGTAACCAAGCCAAAGCCCTTTAGGCTCG

CGTCCCTCGAGAGACACCAGGAGTGTATGTTGGAATTTCAGAAGAAG

>CK173910_bb No definition line found

GGCAACCTGTGCTACATCTGTAACAATGTCATTGGTGGGGATGGTACGTAAGAGTGTGTTTTTTGTTTTTGYKAGCACTT

CGTTCTGTGCCAAGTGYGAGAAGCCATTCCTTGGCCACCGGCACGTACGAGAAAAAGGGCTTAGCCTACTGGCGAGACAC

ACTACCACCAGCTATTTGGCAACCTGTGCTACATCTGCAACAATGTCATTGGTGGGGATGTGTTCACAGCACTGAACAAG

GCCTGGTGTGTTCACCATTTTGCCTGCTCGGTGTGTGACCAGAAAATGAGCCAGAAGACTAAGTTTTTTGAAGTGGACCT

GAAGCCCGTCTGCAAGCGGTGCTTCGAAAAGTTTCCGAGTGATCTGAAGAAACGACTGAAGAAATCTTACGAGGTCAATT

CAAAGAATAACACCTGGCAACCCTGTGCATAAGGAGAGCAAGGCAGCTTATCTTTGCTCTGTGCAACATTTTAATCAACT

AGTGTGCCAAGCTTACAAGAGTAATATATTTACTATATTACGCACAGTTCTCTAGTTGTCACATAGCCAGGGTCTTAAAT

TGTGCATCGATTCTTATATACTTGTTGAGTGTTGTTTACATGTTAACAATGCGTGCTGTTGCTGTTGATGTCAGGTATAT

TTGAAATTGAACTTYGCAGGWGAGTGGCATTGCCTGTGGCGAATATATTTTAGCTTTCAGAGTGGGCATTTTCAGTGCTG

GTAAAAGAGCTAAGAGTTCAGTAATTGTGAAAAGTCATTCGTGCCTTCTTAAAGTTATGGCTTTCGCAATGAATACTTTC

AAAAATGAATTGAGCAAATAATTTGCTTGTTAAAACTTCAGGCCTTCACCTCCTATTCTCTTTAAATGCCTACTGGCGTC

AGGATGTCTCGTATTGAAAGCATCAATGTAATTTGAGACATCCATTGAAACGCAGGCCTGTTAAGAGATTTATACTATTT

GAGCATGTTACAACTTGGAGAAAATACTGTCTATTAGTGATGGCACACAATTTTCTTAATTTCATACATAGTAGCATGGG

TACATTGTTGGTCATGTGTTTCTTGAATGGG

>CK173913_bb No definition line found

AGCTGCCCTCACCTGGCTCCGATTGTGATCAGCTGTAGTAAATTGATAGCAGTGTGTACAAATAAAGCCGTAAAGCTGTT

CTATTCYTGTTTCATTCCTGTTKCACCTTTATTGCAAGAGYACATAGAGCATAACACAATCRGAGCSWGGTGAGGGCAGC

TTGAATCGTTCACAAGCGCGACAGGTGTGTGTACCTGCTTTTAAAGTATTCGTTTAGACAATACAGCTTTTACATGCATA

TCAGCCTCGATCATGCGGCATATTTTTGTTGAGTGCCACCATTTATTTAAAATTTGACCCTGCTACATAAACTTGCATGT

ACATTCTGCAAAAACCATAAGCAGCAATTGCGATATTAAAAAGCAACAAAAAAATTAATGTTACTGTGACTGGTTTTAAA

ATTTATCTATGCAAACCTGCAGACTAAAGGCACTTAGGCATGCTTCACTACAATGCCTATCACACCACCCATGGAGCTTT

AGCACCATGACACACCAGCAAACTCTTCTGAAATTGTTGCAAAGTTTTCAAGTTCAGCATCAAGTCTCACTAAGAATTGA

CTCTGTTTGCAGAGAGTTTTTAAATTATAGGCTGCATGAGCAAAAGCAAAAAAAGAAAAAACGTGGCTGATCCCTCTATT

TAGAAATCAGTACAGCACGAAAGTGAGACGTGTCCTCCCATAGATAGTTGAGCGTTCATTGTGCATTGTTTCGCCACAAG

GGCAAAGGAATTAATGCTACAGCAACAAATTGCTTTGTCATGCAAAGAACGGCAAGCAGCTCGAAACTTCTAGCGCACAC

CTCAAGCACAAACCATGCACAAAATGAGCATGCACAGGACGTGCGCGGACAAACTGTCACAGCTTGACACTATG

>CK173914_bb No definition line found

TAATATGTTGTAAATATTATTGTAGTCACTCGTGACAGTTGCRTATTCAATTAAAAACTACYTGTGGTAACMCATTGCTG

CATTCTGMATCTGATTTCTGTTCTGTGTTTTAAGCACAAGMATTGTACATAAATGCACGCCTTGTGTTCTTCCAATAAGT

GCCTTTAAGAAAAGTTTGGGCAAACTGACYAAATAASCRCAGCGTGTGGGTACCCATACTGAGCAAGGGCAACTAATGCA

GCTGCTGGGTTGGATTTTTTTTTTGTTATATGAGATGTGAATATACACAATAAAGGCAGAAATAGCATGGGGATATTAAT

GTTTTGATTGAAATGTGGATGAAATGTCTTTGTGCAGGCAGGACCTAAACCATCTTCATGTAGTCTTTTTCCTTTAGAAA

TGCTTCGACAGCTGTTGGTCGGCCATTAGCTTTCTTCAATGTGTATGTCTTCTTCTTTAACCCTGGGAAAATCGGCAATC

GCCACCCACGGACTCTGTAGTAGGCACCACGATTCGGGGTGTCTGTAGATCATTGGAACCCTTGAAAGCCCTGGAACCTT

TGAAGATGAGTATGTTGTTTAGGCACTGGAAACCACCGAAATAATTTTTTCTTGAAATCGTAAGTAGCGTGTTATTAGAA

TAATGGAAAAAAAAAAAAGAACTTTCAAACCTGACCTATACTTTCTAAGGCGGATAGACGTGGAACTGGTGAAATCTCAA

TTTTGGAAATGGTGCATAGTAGAACTCTGGATGTATAGCATCTGGAAGGAATACACTTGGAAGTTCATTATGCTGGTAAT

TTAGTATAAAAAAATGTTAGGCGCAAGTACTACTTGGCTTCACTGTAATACATGCCCATGTTGCAGTGGAGTATTATTAG

GCAAATTTGTCATTATAAAATGCAAACGAGGTTTTGCTT

>CK173940_bb No definition line found

AAAAAGGGAAAAGAAAACGCTATTTTAATAAAGGATCAACATCAGCTCAATTTTTTTATCTATTTTGTCAGCTATATTTA

TTTTCTAAGACATCCAGAGAACAGATTGTTTACAAATCAAAGAAAACAAATACTAGATGAAAACTCCGAAGGATCGACTT

GAATGTTTCTGCTGTGTTGGGGGCTGGCTGGTCCCGACRAAGCAGAAGAAGAAGACGGCYGACGGAGWCGWACGCRGCCG

GCGGGGACGCCGACMAGCCCGAACACGCGGGTTGGCGCGAAACGCCGAAGGAAAGAACAACAACCGCACTCCACGGTTAC

CCAACACACACTCGTCTTTATTTTACAGTCGCCTTGAAWTCCYRGATGCCAGAGCGGCGCCCCCTGGCATCCTCACATGT

CAATCCGAAACCGAAAACCGAAAACCCACAACACAACAGTACTTATATCTCATTCTGGACACACTTTTTTGCCTCTAAAA

ATTTGTGGCCATGAGTCATTCATGGCTCATATTTCTCCACGTTGACTGCAGATTGAAAATAACTTGAGAAATGTGTGATA

AATGACCTTGAGAAATACGTGTTTTCAATGTAGGAATGACAGCTCAATGTGGCAAAGATGGGCTTTTCAATATACTTTTT

TTCATAATAAAAGCTTGGGAAACATTGTTGTCTTGAGCTTTGGCAAACTTCTCATGCAAGTAGCTAGGTGACACTTTCTA

GGGGACTCTTGACAATAAGCTCTTGTTAGCTTTTAATGGCCACGTTTTGCTCTTTAAAAGTGATTTTAACCTATATTGAG

TTTAATTACAATGTTGATTTTTTTTTTGTTATGAAAGTTAAGAAACAACTTATGAAAGCAAGTAAATGCAAAAAAAATAC

ATTTTTGGGAAACATTCTTGTGTTTTTCTAGTTACATCTCTTTTCAAAACAGCTCAATTAACATCCCACATGTGTTCCTT

AGTTTCATTATTTTAGTTTTTATT

>CK174023_bb No definition line found

ATTTCTTTTCAACATTAGTATGGGAACTGCATGTTAGCAATAAGTGTGTGAAAAACATCTTCCTAAAAGCAAGAAAGATC

TCACGGTGGCCATACCAGCATTACGTTCATGTGCGAATACACAACTCGTGTTAAAATCACGCTACAGTGAAAAAAAAAAA

AGCCTTCCATACATCGAACGACCATAAAAAAAAGGCTATTCACCTACACCTTGCATGAATCCCACAGAGCACATACAGCT

CAAGTAAAAAATGTGCAACACAATGAAGTAACAATACCATATGCATTACAAATACAAGCACAACGCACATGTTCAATGCT

TGTTGGTAACAGTGGAGCTGCCAAATATGATCTATTTACAGTGATCCAACCACTTGATTTGCAAAGATTCTTCTGATATC

TCACACAATGTACCAGGCACATCAATAGTTTGTGAAAAAGTAATTAAGCCCAGCTAGATCATTGAAACAACACTTAAAGC

TTGCACCACCAACTGGCGCCTGCCTTGTGTTATCCACTGAGAACTCGGTAGCAGGAGAGTCGCACGCACGATGCATTCAT

AGCTATAGTCAATATACATGTGGCRAGAGAATTRCATGYGAATGCACACATTAAAAAAGAAATAGGAAGGTGGGGAATTC

TCTCTAGYAAACCATACTTCATGCACTTCGGCACAACACAATCGTGCCACGCAGGCATTCGTGAGCRATTGAGYACAAAA

CTGAGTGCTGTCTTCGTTCCTAGTACTATGCAACACTGCACGTTTGTTTGTACTTTGAGCATACAAAAAAAAGAATTTAA

GAA

>CK174030_bb No definition line found

CCAGTCACATTGCATTTATTTTKATTGCAGGGTGGACCARAAAAAAGACACCAGGAGGGGATTCTTCAAATGAACATGAC

CATTCTCTAGGCTGGTCTATGCAAGGACTCCGTGCATAGTTCTCAAGCTGCCAACGCACCGACCGTGTGCAATTCCATGT

GTACATACATACATACATACATCAATGTGATACATGCTCACGTTGTACTTGACGGTATAAGACCAACCGGTACACTTTTT

ACGAGAATGTTTGCARAAAARAAAACTGCATGCTTGGCWTGTTTTAACGATATTTTAATTTARAAACATTGTGATTTTTT

AACTGTCTGCSCTATACCWTTATATAAATGCTCACTGCAATAAGTTGCAGTGCAGCAGCTGTGCACTGCAAATATTTGCA

CTATTTCTGTTCTAGCAGCATATGGCCATCCTTGAAACAAAAAAAAAGTGCTACATGCCACATATGCGTAGCTGCACAAA

TTAGTGCAAAGCATATGCTGTACTTTGACATTCCTTTTGGTTTATGCAAATTCTTTATCTCTACCGTGCAGTATGCTTAC

CATATATGGTTACAATATAAACAATCCTGCACTCTGTAAATATAATTTGTTCTTGTTGACTGTTTATGAACAAATATGGT

GTCTACTTTGTGAAGCTTAATTCGCTGGAATATGTATAAGGGCATGCTTGACACACA

>CK174234_bb No definition line found

ACTATTAACTAAAACACGTACACACTTGTCACGCATCGTCGAGACACAAAATTTTTTTGTTTGTTTGTCGAACTGTTCTC

TCTACGATAAAACTCCACAATCTTCTTCGTAGCTCTTCAATGATAACATGGAGAAGGAAGAAGTTGTGGGCCTCGAATGT

TATGAGTGGCACAAAACACCCGCCAATCATTCTATAGATATAATACGMACATTGATAACCACGGATGAGAAAAAAAGGAG

CGGCTGCCGKTGCTCGTCTCGTATGARTYGGTGATGTGGTATGGACGTAAGTGAATTACATTGCTAAATTATCATCAYGA

TCTTTCCTATTTCTCTTWTTTTTTTTCGCTCACAACGTTGACARATACGCATGACAACCRTAATGTATGCGAAGTGGAYC

GAGGGAAACGAAGTATTGTAGCAACGAACCGGTCCGAAGCTAGGCCTAACCCGTTAGGCCTAGCTTGCGTCGAACAGCAC

CATCAGCACGCCACAGCTGACGTATTATTTTTTGAAAAAAAAGTAAAAAAAGAAGAGACAGCGCATTTACTGTGACGAGC

ACAACCCGCTTTTGAGCAAATACATGTACCGAGTAAGCCAATRTATAGATGACATGGTCGGTGCGCTCTTTCTTCAACTA

TGCATGCGTCATGCAATATGAAATCGTCAATAAAAATTTACCACTAGGTAATAGATTCGCGTGATTCACAGTCTTGCTTG

TGCATTTGTGGATGAGAAGTTGCGGAGAGCCTTAGTACAAAACTGGGCGAACCTGCACAAAATTCAAAAAACGTTTCAGT

TTTTCACATTTTAGTTTAAACGGTATACGGGGTGAGTTAGTGTTTTTCGACGGACACGATGCAAACAGACCAAGTTTACG

TGAACTTAGTGTTTATGTGCTTCAACTTGCACGTAAAT

>CK174235_bb No definition line found

GTAAGAGTGAGTGAGGTGTGCGTGTATGAGTGTGTACTACGTAAAATTCGTCTCGTTCACAAATACTTGAAAATGCAGGA

AAAAAATCATTCATCACCAGGCTGGCGTCTGGAATCTTAAACGCTTCCTTTGCTGTTTCTCTTTTCTGTTTCTCAAAAAG

TGGCTGCTGTGACTACCATAACGTATAACGGTGATAATCTTTTCCTTGACCAAAATTTTATTGTGTTTTTTATTTGGTCA

AATATTAACAAAAAACTTATTCTCTTCCTATTAATTTCATTTTATTTGCATATTCATCACTAATCACATGTCGTCACAGG

TCAGAGACGATTCTGAATAATTTTCCATTTGTGACACATTTCAGACAGCACCTCGGAACGCACTTGCATGTTTTTCTTTA

TTCTTATTGTTTTATGCACTTGAGACTAACGTTGAAATAAATTTGACTTCAGTTTTGACTYGGTCGCTTMATCTACATTG

ACTTCATGATTGTGTGCAGTGCCCCTTCGTAGGCAGTGTCTTGTGGCTGTAGCGCTACTGTCTAGGAACAAAAAGAGAAC

CAAATGTATGTAAAAATATGACGCATCTTTCGCGTCGTTCATTCATAATCTGTGAAGCAAATTAGGACTACTACTATGTT

TCAATAGCACGTGCGAGATACCCGAAAACGCGATTTTCTTCCTGGCTTCATTGTACAGTTTTGTCAATTTTTCGTTCACT

TCAAAGGATTTCCTCACTCGACCATGCGAAATACCTTTTTTTTTTTTTCAAATGTCCGTCTTATAGTATATTTTCCGTAT

TGTTCAGATGTGCTTCTCAAAGCTGCTGCATCRTGGTGRAAGCTGTGGAGYGAGTGTCAGCTRTTCGGGCGCATATTCTR

CAAATCGATTTGAGGGGGCTGCCGGGTCAAGTTCTCACGCTATAGAAGAGAATTTGTGTAGATGGGCTTTTTCTTATTTA

TGGTTAAATATATGTGCAGGAAAAGTTGGCATAGCGCACAGTGCACGCGACTTCTTCAGACTTAGGCCAACGGTGCA

>CK174253_bb No definition line found

CTACTATTGCAATTTGTTAAGCCCCTTGGGGAAAAAAGCTGTTGGGAATATTAATGACATGAAGGGGATCGTCAAGCGCT

TTGTCCCATTCGGTTTATCTGGGACGTTTTTGTTTTCTCCTTTTTTTTATATAGTATTTGTTGGTTTGCTTGGCGAAAGC

TGTTGGTTGACGTGGTGTGTACATAAGACTTGAAGCCAGAGAGACCGTTGTTGCATGCCAGTTGGTCTATTTTTAAGAGA

AAAGAAGTGGATTGAGATGAGAGAACGAAGTCGTACATGGAGCGTGCAGTGTTTGTTGCATAGTACAGAAAGCAAGAGGA

ATGTATCTATAAACCTCGCTCTTCGTATTTCCCGTCTGTGTGACTGTTCATTGCCCTTCCCACTTCTCTTGTCACTCTCC

ATCGTCACGTAGTAACTTAGTGCCGGGCAACTTTTCCTTTTCTTCCCCCTTTCTCTCCCATTCAAAACTTTGGCTGTTGC

AGTCCACATGGTGATAGGACATTGTAGGAACATTTAAGCAAATTGTTTGTACATATAAAGGTCAAATAGACGCACAGTCG

CTCGCTTCCCAAAAACGAGCTGAACCTCAGGCTTCATCACTTGCGGACGCGCGTTTTCTGGTGGGCGTTTGCAAACGTAG

CCGACTTAGTCTAAGAGAGCAGCTCGTTTGTTGCGACGTTTCCTTTGCGTTTTTTTTTCCCCGGCTCCTAACAACAGGCG

AAGAATAGAGCGTACACTTTCACGTCACCACCACGAAAAGTTAGCTTTGACAACGTACACTTTGCCGAGAGCAGTCGTCG

GGTGCTCGCTTTCGCGCCCAGCGCCAGCTGGTTAACAAAAG

>CK174265_bb No definition line found

TAACATGTTGAATGGATCTTGGNGTAAACGACTGCCAGTTTCAGTGGCCGTGATTGTAATCGTCGTAAGCGTACTATTTA

CTATTTCTCGCCGGTTTTACGAGTTATAGCGCCCCGTATGTTGTGCACTGTCCGAAGGCTGTGTGCGCTTTTGCCTCCAA

AGGTATTGGCTTATTATCCGTACCGAATGTGCGAACACTGGACCGCTCCTTTGTTGCAGTTACCGCAACGAAGAACTAGT

CTCGTATGAATCTCTTACTGTTGAGCTTCGAGTGCATCGGTTCCGTGAGTACATCTCCGGCGTGGCTTTAGTTTCGCTTA

GGGCCCCGAACTGTACTCGTCGGTATGCACCCTGCGCCTGAATTGTAGTGGGACCAGCGTGAGTGCTTGTTCGCTTTTGG

CGGGAGTTCGCTACCTCATCAACTCGCAGCTATGGGCACGTCGTCCGAAGACGTCCTTCTTATGGTCAGCAACGTGCGCT

ACAAGAAGAACAACGGGACCCTCTATTTGATGGCAGAGCGCATTGCGTGGATGTTGGAAGGCAAGGATTCGTTTTCCGTG

AGCCACAAGTATGCGGACATCAAAATGCAAAAGATATCCCCCGATGGCAAGGCCAAAGTGCAGCTTCAAGTTGTTCTGCA

TGGCGCGGAGGGCTCTGCAACCACTTTTCATTTCGTCAATCCCCTGGGCACCCAGGCTCAACTGGAAGACAGGAACAGCG

TGAAGGAGCTGCTGCAGCAGCTACTGCCAAAGTTCAAGAGGACCTTAAATTCTGACCTCGAAGAAAAAAACAGGATTCTT

CAGGAAGACCCACGCCTGTTCGAGCTGTACAGGAATTTGGTTGTCAGCCGTGTCATCACCCCAGAAGAGTTTTGGGCCAA

CCATGCTCCTAAGAAGACTGGAAG

>CK174319_bb No definition line found

ATCTGCAGCGCACGCTGCTTCAGCAAGCATGGGTAGCATGCGAATGATGGGTATAGTACATGGACTTGCCTCATCTTCCT

TCATACTTTCGGCTCCGTTTGACTTTGCGTAAATTATAACTGCGTTGTGTCCAAACGAAAATCAGTAAACGTTCAGCAGC

TGCACAATTCACCACCTTAACGTTTCCGGCTCGCCAATTCAAATCGGGTCACGAARTTCACAGTGGTTCGTTCTTTTACT

CTACACAAAACCAAAACGCAGCAATARACAAAGCCACAGGTGCGTTCTAGGCCCGCAGACTACGTCAATCCCATCATTCC

CGTGCATTCTCGCTGTATGATCGGAGCGAGGAGCACTGATCTGTTTAT

>CK174332_bb No definition line found

CATTGTCGGACGACGGCGCAATGAAAAGTACCTCGGTTTTTGTGCCCCTTCACTCGACTCTCCTGGGCGGCGGGCTGCCT

CACAGGCCGGGCTGGCCAATGCCTCGCCCTACTCAAGTGCCAGTGATGACAATGCGGAGCGCGATGCTCTGCTGCGTGGT

GTGGAGGCTCGGGACCTTATCGAGTTTGGCATGATACCTGAGTTTGTGGGCCGCTTCCCTGTGCTTGTGCCTTTCCATAG

TCTGAGTGAAGACATGCTGGTGCAGATCCTGACAGAGCCGCGAAATGCACTGGTGCCTCAGTACCAGATGCTGTTTAGCT

TAGACAAGGTGGAACTTACCTTTGAGCCTGAGGCGTTGAGAGCCATTGCTCATCTGGCCATGGAAAGAAAGACCGGTGCA

AGAGGGCTGCGGGCTATCATGGAGACCATTCTTCTGGAGCCCCATGTTCGAGATCCCAGGTTCAGACGTGACGAGTGTGC

ACATCACACACGACTGTGCCATGGGAAAGTGTGCACCGCTGTACATTCGTGGCCGACCAGTTGAACAGGACTTTTACGAC

GACAGCCCAACTGCTGACCAGGAGAGGGCCGTCAGTTCTTAGGGATGTTTGAATTCATCCCGTGTGCAGGACTGTTCTTC

GACARAGAAAAAAAAAAAAAGAAAAATGCCGTGACTGCTCAGTCAGATCCGAAAAGCTGCAAGTGTTTTCCTGCATCTTG

GAACAAAATTTTCACTGCGGCAAACTGTTGGTTGAAGCCATCTGCTTGTTATCTGCCAAAATTATATTTGTTGGGGTGCA

CCAACAATATTTCTCACCATACTTTTTTCTTTCCTAACTAAATGATAGAACTCTCGCTGGATTCTAATTGTGTCAATGAG

CTGAACTGCAGGCTTGAACTACAGCCGGTCAAGTAGGCAGCATT

>CK174346_bb No definition line found

ACTCCATAGGGTGTTTTTCCAAAGCATTTTCAAGCACTGGCATAGCTCTGCGATGGAATACTCAATGGTGAATCAGGGTT

CCATTCCARATCAAACCAAACAATGCGGCCATCATCATATGAACCATTTAAGGCTTTTGTCTTAGTAATGACCTARACTG

CCTCGATTGAAGGCACCAAGGCGACACAGCATCTTTAAAAGACTGTGTAGTGATAGGCTTCGGAATAGCTTTGACTGCCT

GAAATTTCTTACAGTACATCAAGAGCACAGTACACAAATGAAAGAATACTTCTGCATTCACTCATGATTAAAATGCAGCC

AAAAACAGTCAGACAAAAAACATAAAACCTCACACGTCAGCCATTCGAGCTGTGCGGCAGGTTCGCTTTCAGTGCTTCAA

GATGAACAAGTGTGCTAAGCACCAAATTAACCACTTTAAATTCCTTAAAACAAACAACTACTGTGAGATTTCAGTCACAC

CGACACAATAATAAGTCAACTTGAAGATATGATGAATATGCAAACAGTTTTCGGGGGAGCCATGAATGCTTGATTGTGTT

GTGATCAAACCCTTTCAAACATCAACATACTCCATCCTCAGAGAAATGACACAAAAAAGACTTCCGAGAAATGACAGTGG

TGGTCAACGTTGTGGGCTGCCGTTGATCAGGAAGTAAGTTCCCTGAGAA

>CK174347_bb No definition line found

CCTTACTACGCGAGCTTCACCTTGTGGCTGTGGAGCCGGGTTTGTTGAGGGGTCTTCAACAAAGGGCAGCGTCTCTGCCA

CCTCCATATGAGGCGGTGGCCACTGGCTGCGACTCTCCCATGTGCCGCGGTTCAACAACCGCTGCAGCATTTCCACTGCT

CATGGCAACTACAGAAGAAGACCCACCTTCGTACGAGGAGGCACTCCTTCTCATTCGTGGTGGCTCTGGACCCTCTCCTG

CTACAACAAAAACGTTGCCAGAGGTGACGTCATCGCCACCATCGTCGCAGTCTCCTGCATCAGCACCTGTTACGGTGATT

GCAGTTCAGGAGGACTTGCCCGACCGGTAGCAGTTGTTCGAGTGCAACCCCTTGGTTGACAACGTTCTGGTGGCTAGATC

TGCTTTGTGGCATGTGACCCCGTAATCGATGAGTCCTCTGCTCGGTCATTGTCATTCGGTGGTTACGCTTGCTTTTTATA

TTGCTGTGATTATGACTTAACGACTCGACTTACAGCATCGTCGATTTGATTCCGCATTTGGTTGCAGCAATGCGGAGCTC

CTTGGCGGGCGCCTTGTTGCAGAATGAACAAAAAGWAGAAAAMGATTTGTGATGTGTTTAGACGTGAGTGTGATTTAAAG

TGTGACTTTATGGGGGATCGTTAAACGATGGTGAATGTGTTTTCAGTGTTTTAGGTATATTAGATAGGGACAACTAGAGG

CTTAGCTGTGGGAGGAAATGTGCATTTATTAAGAGTTCTGTTCTTCACTCACTGATGCAGATTAGCTGTCTGGTTAAGAA

TGCTCGGTGCACGCTAGGATCTTGTATGTAAACATATTTTCTTTGTAATTGCCAACTAATGTATTTTTGGTCATGATACG

TTGATCGTGGAAATAGATTGACTATCCACGTCTTGG

>CK174361_bb No definition line found

TGTTGCTGGGGGACGTCACCTTGCCAGTGCTGTTGCCGAGCAGGTAGTGCAGCGCGCACATTACTCGCCTACTTTCGGTT

TTTAGTCCAGTCATCAGTCTCTCGCATCGCTCGCACGCACGCCCAACCAAGCCGTAATGGAGACCGCCTCGCTCGAGAAA

ATTAGCAAAGAGGAAACCATCCGTCTTCGTGACATGTATATCGGKTKMSTTYKMRSYGCTYYRWGCCCTAACCACCTCAA

AGACCGCTGCTGCTGCCAGCAGCATCCAGCACCGATTCGACAGCAGCCCTTACTGTCGCCGCAGGTGTTTCATGGACAGA

TATCCTGCTCGGCTGCTTGCAGTGTCCCGAACTTTGGGAGAGCTAGAGAATGTAGTGCTAGCACATGGTGGACAGGTGGA

CAAAGGAGAGATACAGAGTGCATGCTCTCTAGGCTGACCGAAACAGCCAGAGGCAGTGCTCTGCCAACCGTCCATGGCCA

GAGGAGACCCGCTCGGACTACTCTGGGAGAACTCCCGTGCACAATGAACTCGGAGATAGCTGTAGCCCTCCGTGTGATCA

GATCTCTTGTGATGCCTAGAGATCCACCGTGAGGAAAAAAAAGGTGGGCACCCCGCTTGTTGAGCAGCGGGTGTGGCCTG

CACTTCCCGATGGTAAATGCGTATTGACACGGCAGAAAAGAGGGGAAGGAAGTCGTGTACTGTGTTCTTCAAGAGCAATG

GGCCCCTGAAGATTGTGCGTGGCCAGGGCCAATACATGTATGATGAATCTGGCAATGCATATCTAGACTGCATCAACAAT

GTCGCCCATGTGGGGCACTGCCACCCACACGTGGTCAAGGCTGGTGCAGAGCAGATGGCGCTGCTTAGCACCAACAGTCG

CTACCTCCATGA

>CK174374_bb No definition line found

CTGCACAAAAGCCATTACTTGGAGAATCATCCAGTATGTCACTTTACAAGAAGAATGTGGCGATTGGAACCAGCCTTAKA

CATTGGCGGAAAATGAAATGCGAAAGAGAATGGTTTTTTTATCCTTTGCGGTGGTGTTTGAAGCTTGAAGACTAATAACT

TCCATTACTATTYACCGATATTGACAATTTGTTTTCTGTTCATCTCTTGATATTCAGCACT

>CK174385_bb No definition line found

GTTTGTTTCTTTGGTTCTTTGACATAACACACGGTTATCACTCGTTGAACAGAACTTTCTGAGTTCACTATAAACGTATT

ACCTGTATATTTCCCCCACTACAAGCAGCGAGAACTTCTTATGTTGTCCGCGTAAAGCCCACCGAGATAAGGAGCATTTC

TTTCGCTTAAAGAACACGGGAGTGTCCCACGTGAGAAACAATCGTCATCACATCAATCCTGACCACATGGAAAGCACACR

ACGTAAGCCCGAAAAAGAAAYGCATYTTTAACAACAAATCAAGCAACGTCTGTTAAGGCCCAGTGAACGGCTAAACAATA

TGCAACCAGAAGCACTCTCAATATAATAACTAGCTACAGAATGCAACAGACATATATAAACGCGTACTCATCCGAGCCGT

ATCTTTTTATCRCAAATGAAACAAACATCCACTYCTCGTAACCAGATAAAACCTTACTCAGCTGTACAAACATCTCTATT

TCTTTTTTTCGTAAAATAAAAAAAATCTATATTTTTTATACATTTATATAAGGCTCTGAAAACTGTCATCATCCACTGTC

GGCTTCCCCTGAGTACACATCACACACTACTATAGGGGAAAAAAAATGTTTCCGCCACGAGAGTATTTGGTATGTTCACG

CATGCTCGCGTCGAGAGCAGTGAAACGGGAAATTTTGGCCGCCAAACAACATTCTCCGGGTTTGGCCCGTATTCACAGCG

GTAAAGGCGAAACGTCAAAGAAACTCGCTACTACACAGGCACGATCTTCTCAACCACACAGTATCTTTT

>CK174386_bb No definition line found

CATTTGTGTACTGATTGGCTCGTTTAAAAAATGACCCAAACCGCGCTGAGGCACCTGCACGAAGATAAGTATGTCACATG

ATTTTTATCTTTTTTTTTCGTCTCTGCATAGATTGCTGTTGCTTGAAATGGTTATATGTATGTCGAAGAAATGGTCACAG

TGAGCTAGGACACGTTACCATTCTGCTGTATCGCTGCGCTAACCAAGCTATAGAATAACGCTTTGTGCCAGCTTCGCTCA

ACGACTCGATTATAGTTAAGATGCGCATTCTTTTTCCTGCAAGATGAGGTCAAACAACTCGCCGCAGTGTTCCCCAGKTG

AGGTCGGTAGCGCAGAAAAGTTCGCCGCACGGGACTCTACAGAATCTCACGAATCAAGTGTAAATAGTTTTTATTACCTA

ATCTCGCCTATATAGTTGTTGTAGTGAACGACCCTTCTTATCGACCATGCACATATATTACGCACCAATGTTTCGGGAAA

TCCTCTCTGTTTTAGCGAGAGGCCTTTTCTTATCTGCTCGACCAACGCGAACGATGACTGGCAAGCCGTAACCACTAATC

TATTTCGTTCTCTGCGCGTAATAACCGACGGAATGATAAAGGGGCCCTGAAGTATTTGCCTTATCGTTTGGCTCTCTTTC

ATTGCCTATTTTACGTCAGAAACACGTTTAATAATAATTTTAAAGCTATCACGCCATGAATGGTAGATTGATGCAATCAC

AGCAGGCACAAAAACAAGGCACAAGTTTAGGAAAATATAGAGGCATTCGAAAAGGATATGTTTAGACAATTA

>CK174405_bb No definition line found

TTGGCCACGAAAGAAAGAACGGCATTCGAAGACCTCACGTCTTGCAGTGCAAAACTGTCAGAGAGTAACTGCGTGCAAAT

TGTGAAGCGCTAGCCCTGTGTCTTTATTCACCGGTTGTGTGCCTGAGAACACGACTGAGAGGGTAATCTATACATCGCTA

AAACTGCTCCTTGGTCGAGTTGGTTCATGATTATTTAGACCGATTTATCAGCTCAAAAAACAGAACATTTAAAAAAGACA

CACACACACACACAGCGCTGTGTACGTGTCTTTTCCAAATGTTCCGTCTGTTCTGCGCTGATAAATCAGTCTAAATAACC

TATATAGTACTACACATTCTGGAACTTGTCCCTTGCCGAAGGTGAGACTATGTCTTGCTGGCCGGATCTAAGAGAGACCC

TAAATCCTTCAATGACACAGTTTGAGATCCCCTCTTTGCTGGCTTGGCCTGTGATGGGTCTGGCTTCCAGCCGATAAAGG

ACAAGATGTGAAACGTGGCCGGTATAGATCCGTCCTCATTGCCATAGAGTTCTTGGTAAATCGCCGCCGCTGCGACCATC

GTATCCCTGTGTAGGTGAGACTTGCGGTTCCACGTCACGTTGTTCTCGGCCATGCCTTTGAGGTCGGCCATGAGATGGAA

CGCCGTCGGATAGTTGACTGTCATTTCGTCGGAGTCTATCGTAAGTAGTACAAAGCCAGCGTGGTTCAGAAGGGCAGCCA

AATCCGTAGGTTGGACAAACGGGGATATGTGGGCACCAAATCCACCTTCACGTTCTGTTTCCGCAAGCTGCAAGGAGCCC

CTTAGCTGG

>CK174422_bb No definition line found

CCTCTCAATGGTCACACGCAAGTGGACTCCAGCRCTCTGAAATATTTTTATTTCTTTTSAATAAACACGTTCTTCAAACA

ACAGCCGATCAGCAGTTCTAGATTATCAGGTATGGCATTACTTMTCAAAGTTTATTGGCTAGGTTCATTTTCRAACATCC

ATTGCCKGGYGCTATCGATGATCATTAGTACCTGCGAAAAAAACAAAAACTGTAAATCAAAACGGCCAGATCGTGTCAGT

TTATTTACAACATTGTTGATAGAATGCTAGTATGAGGGTTTTAAAATGTGGTATTACCCAAAGGAACTATTCGCCAGTGT

CTCCGTGTTCTCTGTTAATTTCTTGTGCATCAAATTTATTATTGCTATAAAATAAATGTTCAATTAAAAAACCTTGGAGC

GGAACCACCAACTGGTTACATACTCAACAATGGCATGCGACATTACACATTCTATTGGCAGCGCAAATAAAATGGTGGTA

AACTTTTGTACATGCGCGTAACCGAGTCATGAACTGAATGAAACACTCCAAATACTGAGCGAGTATGCTACGCGAACGTT

GAATGTTCTGCAAGTGGCCCAAACAAGGAAAGAATGACACTAAAATACATTAACTCACTTAGACACTCGAGCACATTGTC

ATTGTTCAAAAAGCGAGGTGATGTTGCCAGGCTACTAAACCAGGAATAGAAAAGATAGAGTGTATAGAATAGGTATACAA

GCTTAGTGGTAGTGTTTTTAGTTGGTGTCTTTTTAAACAATTACTTGCAATGGACGCGTCTAGAACTTCC

>CK174450_bb No definition line found

CGTGCCTGTGATGACCAGGTGCGTATCGAGAACCTGGCCACGGGCGACGACGAGGTGTATGAAGACGGCGCGAGCAAGCC

GCGCGTGTCGGTGCCCATCACGCTGTGCTTCGTCATCATGATCGGCTACATCTCCGGAGGTGCAGTCATCTTTTCGCTCT

GGGAGGGCTGGGAGTTCCTGGACGGCTCCTACTTCTGCTTCGTCACGCTGAGCACCATCGGCTTCGGGGACCTGGTGCCC

GGCGACACCGTGGTCTCCGACAGTGGCTCTCAGGAGAAGCTGGTCATCTGCTCGCTCTACCTGCTGGTGGGCATGGCGCT

CATCGCCATGTGCTTCAACCTCATGCAGGAGGAGGTCATCTACAAAGTGAGAAACTGTGGCAAACGCATTGGTATTATCA

AGGACACTGACGACGATGAAGACTTCTCCTGAGGCATGTTATCACGACACCTGGATCATGGCACCTTGGCCAAAAGACTC

TGCAAAGTGCGAAATGGACTTTTTTTAAAACGTTGAATTGTGAACGTGCTTGGGAACGTTCTACTAGCGCTGTTCAACTT

AAATTTGCAGCAACCGCAGTGGCCAACAGCTGTTGGCGTGGGCTATTGTGACCAAGCAGCAGCGTGGCGTATACTTTCGT

CCTTTGGCACGCACTATCATTCGTGCCATCAAAACCGGAATGCTATTTTATGATRTTCGACGCACAGTTGAGGGGTTACC

TCCTGGAAGGACGTCTGTACGGAAGGGAAGGGCGATAAACGGCACTGCTTATATTTGACGGACATTACCATAGTCTTTGT

TGCCGTGAGTAGTCATATTCATGCCGAATGATACGTCCAATTATTCCTACAACTCGTTTTACTTAACCTGAGGCAAACAA

GAACGTTTTTCTTCGGTCAT

>CK174474_bb No definition line found

GCAAAAGTTATACTTATATGGTAATTGTGTGTGTTTGAACCAATCTTAACATGGGTACAAAGTCTTTAAAGGCTTTGTTC

TACATTTAGGTAAATATTAATGTTCAGGCACCTCTGCCACAGAACACAAGTGCCAYCAGCATCAACATATCATGTYCTTC

CTGTTTGCACRCTTGTAAATATTGCTGTATTTCTGAATGTCGAGATGCACCTTTTGTCAGTAATATGAATACTTGTGCAA

AAAGTTATCTTTTCTTCTGGACCCGTCTTTGGTCACGCTAGTGCCACTATTACTGCGTCTCTTCCTGGTTTTTATATCTG

AACTGCGCACTGCATAAAGAGGCTACCTTATGCAGTTAATCAGCTTCACTGTACTTTAGGCCATTTCAGACCATGCTTGA

TTCAGTACCACTGCTTCATCTCAGCATAATGTTGCATTAACTTGTGGACACATAAGTAACACATTAATAAGGTCAGCTGA

ATTCACAAACTCTTTCTTCTCCATTACACTTGTGATCAATCCTTGTTTCTTCCTATTCGCATGCTGAAAACCTGGTAGCG

ACCTGACTTTTTACTGTGCTTTTTTTTGTGAAGGTCGTGAGCAACTCATGTAACACAGCAGATAGAT

>CK174476_bb No definition line found

CTCGCAGAAGCCCTCAAAGAATCGTAAGCATGCCAGGTCAATACGTTGTCGTTCTCTTCCCAGAAGAGGATGATACATCT

GGGATCATCCTCAAAAGTTGGCTGAAAGGCGACGGCTGCCTGTGGCCTCGACAAACTAAATATGTACATAGTTTGTTGAA

GGCGAAAGCGACTCCAGGGGCTGACTGGATAGAAGTGCCTTGCACTGTTGTCCGAGAATTTGATACATATGCCGAAGCAC

GGGCAAATCTGCCAAGGGTAGAAAATGGATCCAACTTGGACGGCGAGGCAGAACTTGGGAAAGGCAGAAGAAAAAACTTT

GATGGCGATGAGCACGCTTTTACCCCATAAATGAAGAGTTATATTTTAAACATTTTTTTTTTGCTTTCGTTGGGAAAGTG

ATAGTGGCATCACTTTGCTGCAAAAACTATGGCAACTGTTATATGTATGGTCTGGTCACACATTGTTTCAGTGCTTAAAA

CATTAAAGTATCTGTTTCTGCTTATTCTTATTTTTTCACAAATTCACTTAATAACCTAACTTTCAACAATTTTTCTGAAC

TTATTCCAAGTTTTTGTTGAAAACTAGGTTGTAAATATATGGGTACACTGAATCACAGCACGCAATGAAGGTGCTTCATC

TCAGTGTCAACTCTGCATGCGTATTCAGGTCCTAAAAAGGCATCACTGAGCATGCTAACAAAATAACTGAAGCCATGTAG

GCACAAATTTCTAAAACCGACAATAATGTGCAGAGAAGCCATAAACAACTGTGTGCTTGTTTTGCTGTGTTTAGTAGCAC

TTCTCATTTTCAATGTTATCTCAGCTTTTGCCATTTTATATGTTGCAATTCAGCGCTATTACTTCATGAACACGTCTTTA

CAGAATACGTGCGTCAACTCATGCGAATTTCGCAAACTATCCTGATGAGGCTAGA

>CK174532_bb No definition line found

GGCTTTATTAAAGAATATATGTACAAAAAAACTCCCGTCACCACATTAACCACATCATGTTCACACCGTGCTTCACACGC

ATAACTATTTACAAACGATCACTACAGCACGACAATTTTCTGTTTCAAGACGGGGTTGCATCATGTGGCAACCTCTCAAA

CAAAGAGTAATAAAGCCAAATCAAAGCACTAGCACACAAAGCTGTCACATCTCAGGTGCAGGCAAGAGGTGTCGCAAGTG

TAACTCGCAGTAGTGTCAACGTAAAATAAAAAGTATAATCATAACCCTTCCCCCAACATCCTCTGATTCGAGTTGCCTCT

TCGAGTTTGACGACTACATTGGGAACTAGCCATARACGATGGCGTGAAAGGCGCAAGACTTAGAATACTGAAAATCTCGC

ARACGCGATGGTTCACAACTTAAGTGTGTTCTATCATAGTCATAGTCATGCACTTTTAAGTTGCTTGTACTATTCAATGA

AAAAAGTATTTGTGAAACACTTCTTTCCTCCCAAGCTAAAGTTCCTTCCTATCAAAAAGAAAAAGATCCCTTAGTAAGAA

CTACATTATAAAATTAATTCTTTAGGCTTGAAGTGGCAATATTGTAAATACTTTTATAGAAAAGACACTCGGCAAAGTAA

AGTTAGAAAATTGCACTCTGGTCCCTAAAAGCTACAGAATGATGCAGTGATGTTCAAATAAAACCTAAAACATGACGATC

TGACSCTTAATGCAGTGAGCATGGAGCTGTAGCATGGTCCCCTGCAGAGTATACTTTGAAGTGCTTGCACGGGAAATTTA

TGAAATACTCGCGAGGCACTTATGCGACATTCTCCAACACAAAATGAGCTCGCGAACAGAAAAGGTTCAACCGTGCTTGA

AACATTGGCGATGAATGTGCGTGCACGTAACCTTCGTTTCTGGCCTAATTGCCATGCAATGCAGTTATGCTTATTTTTAT

TTAGTGTTCCTTACGAACCT

>CK174560_bb No definition line found

ATCTTGCATGTATTGGTTAATTGCGCTTCGACAATGACACAGAACCGAGAAATCGGGGAGGACGGGCTTGAAAAAACCTT

CGCCACGAACACACTTGGTGTCCACATCCTGATTACCAACCTGATTCCTATGTTGAACCGGAGCTGTGAGCCACGTGTGG

TGCTGGTCACCTGTGGTACCCTACTGATGCAGCGGCTGGACCCTGTGGACCTTCAGTTTGAGTGCATGTTCCCCTTCAAT

GGGTTGACAGCCTTCACACAGACCAAGCGGCACCAGGCTGTGTTGGTGGAACACTATGCCGAGTCTTTCCCAGGCATCCA

CTTCTCAGCCATGCATCCTGGCTGGCTTGAACCCAAAGTGGTGAAGGACGCTGTTCCAAAACTGCTGAGAAACGTGTCTG

TGCGGTGCCGTTCTGTAGAAGAAGCAGCCGACACAATTTTGTGGCTAGCCATCTCACAGGCGGCTCTCAAGCACTCAAGT

GGCATGTTCTTTCAAGATAGGAAGACAACTTGCCCACACATTCCCTTTGGCAAGACGAAGACAAGCCTGGATGACGAAAA

GTTCTTTATGAGGAACTTGGAGGACTTTGTTAACAAGCTGGCGCTCTCATAGCCACTATTCCATAAGCAGCAGCAAAAAG

CGATACGCCTCTGAGCGCTTTGGCAGTCTTTGATGCCAGTCTTACTTAGCCACACTATCATGTACTCAAGGATGCCTCTG

GCTAGAGTCTGTTGCAGAATGCTTCTACATATGTGCTAGTTTTCATGTCCCAAAAGTCCATGTACCTGGTGTAATGTGTT

GAAATTTTTATGCATTTTGTGCAAGTTTTCATTGCTTTCAGAGCTGCTTGTCCATGCAATTTCCCTGATCGTTGTCTGTA

TTCCAGAGGTAAAACTACATATGTAATCCAAGAAAAAAACATTTTATAATTCCTCATGGAAGCAGTCAAAGTGATAGCAA

ATATGCCAGTGTTTCCA

>CK174568_bb No definition line found

AGAGGTACGAAACAACACTGTGGGCGTAAATGAGTACTTTGTTCAAAGTATACACCGTATAGGCCTGAGCACAAAGATCT

CAATGATAAACGAGCCGAAAGAATTTTTTCYCTTCAGTTCCTGTGATTKYGRYGACCCCAGTCTCTCACATCGTCCTCGA

GTTCGTCGCCAAGCTGAACRCTTCAATTSCAGAKKTTCTTCCTRTTWATTTCATATACTYTTTTCACGTGTTTTTTTTGT

ACCGTCAACATCCGCATCCGCCAACCATTACTCCCTAAGCCACCCACATTGTTGATGAGATCGGCCGTGATTGTATGCCT

GATCACCGGTCGTCAACCAAGCTCTTACGGTCTTCTGGAAAACGGGCGCTCCATTTTGAAGAAGACATGTCGGAGAACCA

GTGTTCCTCGTATACGGCCTCCATGCGGCGATGAATGGCGGCTGATTCGACACCTCCTGCTGCCGGAAATCGAACAACGC

CTCACTTCTCTCGCAATCGCCAAATTTCGCAATGGGAACGACATCTTGTTCTCGCACGCGCTTCCACGTCGGTTGGACGG

CGTGCTTTGTAAGAGTATCTATCTACCATCCCTATGTATGGCTACGTTTTCCAAAAGCGGCATGGGCGCCTGTGTCAACG

GTAACGTTTTTAGGATACTTCTTACCTTCTCATTTGAACATCCCCC

>CK174575_bb No definition line found

TGCGCCATGAGCTGGCCCTCAAGGCCTCCCTGCTGCAGGTGTACACGGCGCAAGAGGAAGCCGACGCTGCCTTGCCTTTG

CCCACTGACGATGACGATGTTCCAGTGAGCACCTTAACCCGCAAGATTCACGAGCTGGAGGAGGAGAACTGCAAGCTCAA

GGCAGAGGTTAGGCTTGAGGCACTACCAGAGACTGGAGCATTGTTGGGCCTAAACACCCCGGAGGAGACTTCAATAGTTT

GTTTGGTTGGCTTGCTTGGTGTTTGAGATAAAAAAAAATCTGTAAAGAGGGGTTGTGACGGGCCGACATTTCGAAACAGG

ACTTGTCTTCCTCAGGGCTAGAACAGAACCTATTTCTTTAGCGCTAGCGTGCCTGCGCTTCGTACCCGCTTGTCCATCCT

AGCAAAAGAAGGACAAAGGGCAAAGCACGAGAGACGGGGGTCTGGGGCAAATTGTGCGAGTGAAAAAAAAACAAGCATTT

CATGAATAAAAAGAAAATAAGGAAGCAATGGGTAGGTGAGTATGCACAYGTGCCGCGGAGTTTCTGTTGTCAGACCGGTT

GTTAGAAGTTAACAATCGCAGCTTTGAAAGTCCTTGACGGAGGGCTTAACTAGCAGTGGTCTTCTTTGTTTATCTAGCAT

ATCGATTGGATTGAAGACTGCCGCTATAAACGTTAATACCTACTGGCTGGAGTGTGTATTGAACTTGTGAAAAAGCTACG

AATCCGTACATTTTCTGTCTCTTGTGGACCGGAAGCTCGAGTGAAGAATGTCTGCCTCATTAAAATGCTGAGCCACTGCT

TTTGGTTATTTCTTTGCCGCGTGCACGTGATTACCATTTAAATTCACATTAACAGGCTTTCCTGTTTCGCCAATGTACTT

TTTCTGACAATATGAACCTTCGATCATGTAGATAACGTTTTCACTAGTGCAAGTAAAATTTGATTTTATACGATGGACAT

AATCACT

>CK174598_bb No definition line found

CGTGTCAATAATGACAGCTAGCTTGGAAAGTTATGGGCCGACTCAGTTTCCTTTTTTTTTTTTGAGCTCATGGGCATCTA

AAGGGCACGCCGCCACTAAAGTATGGCAAGCGTGGTGTTATTACTCCGCTAATCATGCGACCAGTTGTCCATTGTCTACT

CCTAGAGCGGGCACACTTGTTGTTTATAGAGAATTTTTGTTTCGGTGCAGATCTTTGAAGGTCGAGTGGTAAACAAATAT

ACGGAACATACATTTCTATACTCTGCCCGTGTTCGCGTTATCCCCAACAAGCAGTTCTAGTTTGGTTTGTGAGTGATAAA

TGATCCTCTTGTCACTCATTTGAACAAACAGGAAATCGTTGACTCCCGGTTGCTTACTGCAGGAAATGATTCTTGCGGGC

TTCACTTTCTTAGCACTAAACATCAAATATATCCGAGTTCACGGAAGTCAGTTAGTACCTGTTGGTGACAGAATGCAATC

GAAAAGTTCGATAAATTTCATTGAATGAGCATAAAAGTTATAACAAAGTGCTCACCGTTCTGGGCCCATACAGGATCATA

TTAGCCGAAAAATTGGGTCAAACTTCACGTCGAACTAACTTCGCAAACTTACGTAAATTGACTTCCAGAGCGGCACGTCT

ACGGCCCACCCCATAAAATACTACCCCAGAAAAAGTTGGTGTGTTTTTCCACGAGTAATAGTTGCGGGACTCCTTACCTA

TGAGTTCTTTTTGTGCGGCAATATTTACTGAAATATTAAGTAATCAGCATCTTACAATTTGTCAAAAAAAAACATTCCAG

AAAGCCATTCTACTGCTCGAAGGTTTGCTTTGTATACTAATGCCGTTGTTGTTTTGAATGTGCAGACGCTGCACCGGAGT

GAAGTTATCAGTGTTCTTGCAAACGGCGCGTTGTACCAATTGCAGATTGCAGTGGCGCCATCTTGCCATCGAGGGCAACA

CTGACATCCTTACATATCCAGCTTCTTAGAAACCGCGCTGTGTTTTGAGTTGAAGTTCGCCATTCCACACGTTAGCGTGA

ACTAACTGAATGGGCCCTCCAGAAACATGAGGAGAGAGTGAAGTGATACTATTTTTGGAACAAGGAAAAGTGTCTTCTCC

CCCAAAGCAAAGCCTTGC

>CK174644_bb No definition line found

TTCGGACATTGCACGCTCCCACTGATGGCCCTTTCTCCAGGATCCATTGCTGTGCACTTGAAGTGGACTCGCGAGTGATT

CCTCTGAGCAGGCACGCCAACTGATGACGCGCCCATTGATTGCGTGCTCACTGATTGCGTGCTCACTGATGACGTGCCCA

CTGATGGTATGCTCACGAATGACATGCCCACCGCTGGCGCACTGACCGGCCACATGCCCACTTCATTCGCCAAGTCTACT

TGGAAGTAATTTTCTTCTCTAGAGCTGTGCCTGCTGGAGACATCTTCAATGCAACCCGTTATAGTGCGTTTAAGTGAACT

TGTYGCTCCAAAAAAGCTATCGTTTCYATCGGAGCATATCTTTGATRGAAAACTAGGTGAGCACATGCACAGTYTATCGT

TTYGTAAGCCAATTATTAGTGACWTCCGGTACCAAAGGCACCTATCAATTTGCGGMAGATTGAATTGTGTATATTATTCA

CTTACCTCGTWYATGATAATGAGGAGTAGCAGTTTGGATGGGTCACACTGTCTGTGGCAGTCGCTGTTGACTCTGAAGTG

AGCGGCAAACTGATTCCTTTGAACAGTCGTGCCCACTGATGACTGGCCCCTTTATTCGCCATGTAGACTTGGAGGCTATT

CCTCCCTCTCTAGATGTATGGTGTGTGCACTAAGAACCTTCTTAATGTGTCCGGTTATTTTGTGCTTTAGGCGGGCTTTG

CAAACTTCACTGAAGAAGCGTCAGCTTTTTTCCATTATGGCTTTGAAGGGAGCTTATAAGCCTTTACAGAGTCCCTTCTC

ACACCAAAGTAGCCAGCTGAAAGCTGAAAATTGAATTAGACATATCGTTTACACGCCACAGTTGTGATATTGAAGAATGT

TGCTGTCGCACGGTAAGGTGGGTTGTACTGTGCTGCTGTGGGTGAATAGGTCACTTTAGAGGCTTAAACAAGA

>CK174666_bb No definition line found

GCCCTCCAGAAGCACCTTCGTCAGCACCCACGGTTGCTACCACTAATTCAATGAACTGGAAAGACGATGAATCTCCAGCA

CCACCTACTGCACCTCAGGTCAATCATCAGGCGCTTCCGGAGACAAAGACTTACGCCAATATGGTGAGCAAGAACTCTGT

ACCAATCTCTTCGGCTGGATTTACGTCACCAAGCCCTGCGGCACCTTTTGGTGGTGCTCCAACCAGTGGAACAGGACATG

TTCCGGCATCTACAGGGCACTCTGGAAGGGGATTTGGAGGTGAGCCTCTCTCTGGGGGCCTGCCACCAAGGCCAGACCAG

CGTGGAGGTCCGCGGCCTCAGCAGCAAACGCGGGCACCACGTGCCTCACTGCCACCACCCACCAAGCGGGCTGAGAGTGG

CCGAAATGAAAATGCACTTAGCAGTGATGATGGGACTGCGCCTCCGCCTCTCCGGTCTAGTGTCAAGCCCCAGTACCCTG

ATAACCAACAGGTGTTTGTGGGAAACCTGCCACACTCTATCACTGAGGAGCAAGTGCTGAAACGCTTTGAGGAGTTTGGC

CATGTGCTTGAGTTCAGGATGAACTCCAGGTCGACAAGCAAGATGACAGCTGGCGGCAAAGCTGTACCTAACTGCGGCTT

TGTCATCTTTGAGAGTTGTGAGGCTGTCGAGAYWGYACYACACTRMTTCGCAGCCCATCTACATTAATGAAACTCGTGTG

AACGTTGAAGAGAAGAAGACCAAGCAGAAGCTTGCCACAGAAGGACGAGGAGGCAGCTTTACGTCAGGCAGCCCTCGCAG

TGGAGGAACGGGTATGATGCCAAGAGGACCTCCGGGAGGGCGTGCTAATGGGGCTGGTGGGCGTGGCACCTTCTCGAGAG

GTGGCAGCCGTAACCCTCTCTGGTGGTGGCGTTGGCATCAG

>CK174935_bb No definition line found

AGCAAAAGTTCTCCTATACGGGCAGAACAAACACGTAGACGCCACAGCTTAATTCTACATCTATTAATGGTAAAAAATTG

AGCCTAAGAAAGCAGTAACTCCAGAGTGTTGCCTTTTCCGCATTCTCACCGAGCACTGGCATAAAAGTTTGCTGCCATGC

TGCAAACAACACAGTTGTGTATGCCACAAATAGAGACATTACARCACCCAACAATTTCATTTGTACGCTACTCGAGTATT

TGGCTCATTAATTTTCAATTACCCATGTTCCTGTAAGAGTTCTTCCAGACCTCGGCGAAGGTTACGGACGAGTGAAAACG

GTAGAAAATGCTGAGAGGTAGCGAGGCACGTATGATGACTGTCCAAGCCATGAACCCGTAGAAGCCAAGTTGCACGGGGC

TAGCCTCAACCTTTTGCTTCTCAAAGGTGTAAATGATCCAGAGCGTAAGATTTCCGATGAGCAAGAACGTGATGACCTGC

CGGCCCGGCTTGCTGTGGTCGTGCTCGGGCAGGTATGTCATACGGCAGGTTATGTCCGCAATAAACAGCGACTGCATCGT

CACCTGGACGATAGTCAGAATGCTGGTGACCATAACCAGAAGGTTCGGGATGTGGTCTTTGGGCAGCAGGCTTCCGGCTA

TGATGCCAAACATGGCATAAACGTAGAGTCCGAATGCAGCCACACTGAGCAGTATGCTGCGCAAGTGATCCTTCCGGTCG

GGGTGAAATCGGAGCGACTTGATGCGAAAGAAACCGATCGTTATGGCCCCGATGGTCAGCACCTTGATGCCGCAATGCGA

AAGGTCAGACAGGTAGATGGCCAGCATCGCGTATCGCTCATGCTGGATCAGCACAAAGAAAACAATGAGACAGATGGTAG

AACAGACGAGCACCAGGAGGCCAAAGAACAGACCCTTGCTGGAGCCTGTGCAGTTGACCTTGGAGCCCGCCTGGAGGCTA

GACGTGCGCGATATGCCATCGTCGTGGCTGTTCTCGAC

>CK174957_bb No definition line found

GAGATTTGTCATCAATTGTGAAGAAGCTCCGAGGGATATTTTGGCACCGCATGCCACTGATCGCTGCAATATACATATAC

AATGACTAAGCATCTCTGGTGTTTAGAAATTGGTGTGGCAGTTTTGATTCATTCTCACTATGACAGTGCATTTTTCAAGG

ATTGTGTGTTTACTTATATCGCATGCCCATTCTGTCAACAGGCATGCTAATACCAGCACAAAGACCACACATGCTCTCCT

GTATTTAGTATGCTATGCCAATGACATCTTTGTTCACTGTATTCTACTCCAACGTGGAATTTACTTACTATTTGTTCTAC

AGGGGACCGCATGTTTTTATGCATTTCAGCTACTGTATACAAATGTACAATGATTTTTAAATTTCCCAGTCTTTACACTG

GATCATCGGATCAGGTACATACAATTTATCGGTTTACCCTGGCATGTTTTTACAAATTCAAAAATCGTGTCGTGTGTCCG

TGCAATTTCGGGAAGGATTGTATACAGTTCTACCGGCGAAGAGGCGTGAAGCAAGTGACCATTAGCATGTGCTGGTGCCT

TGTACTAGTGTAATTGAGCTGGTGTTTGAATTCAATTTGAATTTTCCGTTCCTTGCTCTTAGTTTAGCATTTGGAACCCT

TTATTTTTATGCGTGTATTTTGCCTCTTTGTGATGGTCAGTTCCTTTACTACTGCCATATTTTTTGTCCCGTCATGCATT

TGCACTGTTGGCGACAAGTAGTACAGGTGGGAAAGATGTATTTGTGTGCTGTTAGAATTAGTAGCAGAATGCGCCATTGT

AGCCATTTGCAGCATAGCACTTGGTTTCAGGTTGACTGCTTACGTYCRATCTTGCAGGTTATTTCATATAGGTCACCAGG

CCTTYMATTAGGGTAAGTGAGGCATTGMTTTGGTAAAGACAGAGTGCACTTTTGTCATTTATGCCCCGAAAGGTGA

>CK175028_bb No definition line found

CTGAGCAACCTGTGCGCAATGAARCGRTGCTGTGGCGAARCTAATTTCACATTAATTTTGTTGTTTCARARTGSAATAAT

AGTTAAAGAAAGACCATGGAAAGGAGAATGAACWTCGGAGAGCACATTACAGCTCGGATCACGGTTCACTCTAAAAGAAA

AAAAAAACGCAGTTGTAACCCATGGTGGCATAATTCTTACACCAAACAAATATAGCCATTACCTATCTTGTGTGCCTCTC

CTTGCTTTATTCCCGCGCACGTGGTATCTG

>CK175262_bb No definition line found

AGCGATATGGCTCTCTCTACATAAAACAAAAATTATGTCGGTACAATTCCAAGATTTGCACACCTGGTAAGTATTGACAG

TCTCTAGTTGGTTCTATGCGCCAGCCCGAACAAATCTGACAATTACTGTCTGCTTTGATAATTAAATTACAACGTTCTGA

GCAAAGAAAGATGCAGTCCTCCACGAACAGTCAGCTTGCTGAATGGCGCAACGGAGCGGTTGCACCTTAGGTAGTAAAAA

AAAAAAGTGAGACCGACTAACACCCCTCAGCGGAAAAAAAGGGTCAAGCACAAGTGAACATCAGTCTACTCATAGAGTCC

GCGAACTCCAACAACGTGGTGRTCATGTCCAGTCAGCCYCTTCGYTCAAAACGCGTAAACGTTGACGCGTTCCAACACTT

TCGTGGGCATTGCGCCCTGCGGGAGTATCCCGCGTGGGCCCGCCCACACCCAATGCGGGGGCGGGCCCACGGCCTCGCGG

TTCACATGGTTCTCATAGCTCGCACCAGGCGTTGAAAAAAACTTATTTTTGGTGACGCA

>CK175272_bb No definition line found

CACGGCAGAGTGTGCTCTGCTGTCGCAAAGCCACCCTTCAAAAYCTGGGCAGCAACTGGTGGCATAAGTAATCATTTCTG

GAGTTTCACATCATGCTTAAAGTTGGGCTGGTTTGCCTTTTGACAGCGGTTTCAGCCGTCTAATGTGCTCTTGACAGGTT

ACATTTGCACGCAGTTTCTCTTTTTCTTATCCCGTCATCTGGCCATGCATAGGTGAGTTATGTGCACCGAAATATTGCAT

AGGCTCGTGAGAGAACTAAGGTTATAATGTTTGATCAGCTTAAATGTAGGAAGATGACAATACTACACATAATTTAAGTG

CGTGCTTCAGAACAAAGAATAGTTCTTGATTTGTTGAATATTCTTTTTCTCCGTTGACAAGCATTGTAGGTGAACTATTG

CTTTGCAATTTCAGAGCAAGCCTCAACCATATTTGCTCATTTGCATAGTTTCTGTTTACGTTTTCCCTAGATTCATCACT

TGTGCCTTGTGTTAACTTAGTTTCTCTTCCCAGGTTTCCTTTTTTTTTGTACACATGGAAGAAAAAAGTTTTTCTATCGT

GTGTTACTTGAAAACTCAGTGGTTATTTTGGCTGAACTACCTACTTCTTGCGCTCACCTTCTTCGCAAAACTTTGAACAT

TGCAACGAGTTTTGTTGAATGCCCTGCTCAAGTGCAGTACCTACCTTTTACATTTCGTCCAGAACAACTTTATCAAAAGA

ATGCTTTGTATGCAGCAGTGTGACAGCCCAAATATAATTCATTTCAGTGATGGAAGTGGTGGAAGCTCCTGTCAAAGGCG

TACGTGCCAAGCTACAGCCATGTGCAATATGAAGGGGAACATTAGCCTTGTGTGACAGTGGCGCATTGCAGGCTGTCAAG

TATC

>CK175281_bb No definition line found

ATATTGTTGTATATAGCAAATTGGGTGCGTGGGATCATAGGCGGCTGAACGACTGGGGCGCAGCGCCGGTAGACGATGAT

ACGTAAGCTACCACCAGCAAAGAAGAGAAAAGGAATGGTAAAGAACATGAGAATGAGACGCTTTCCTTGAAACTGCCTAC

TGTAATTATATGTTGTATAATAAATCTCGTCAAGTTTTTACTCTTTTGG

>CK175346_bb No definition line found

CTGCTGTGAGATAAGAAGCAACAGTAATCACCTATTGGGGAAGTAACTACAACACAACTGTATTTTGTTTAGAAATCTAT

TGCACTGTAGAATGTTAATGTCCAAGCAGCTTACTTTTGTGTACCTACGGACAGTAGAATGTCTTTATAGTGGACAAATT

TAGGACTGGGTTAAATATGTTATATTGGTGTTACTGCAAGTGAAATTGCTCTGAAGCAACTAGCGGTGTCTGTTATGAGC

TGGACATCGAGAGCTTAGTTGTTGCCATGACAACCACATTTCCACGAAGGTTAGAAAAAAAAAACAAACAAAAAACAAGA

AAGTTCATTTACCATGCAGTAGATGCATGATAAGGAACTTCATAAGGTCCAAATTAATTCCCTCACTGTAGTGTGCCTGA

TGTTCTGTCCATCCAGTTTTGAAATTTTTACAGAAAGCAAATGCAACAAAGAGTTGGTTCAAACTAAGAATCTAGATCTA

GCCCTTTAATTACAAGAGTTTTAAAAGCATGCCACCTTCATTATCAATAGCTTCTTGCCTTAAAATTGTTTGAGTTTTTT

TAGTGCCAAACACATGCATTCGTTCGTCTACAAACACTCGTAGAAATAATAGAAACAATTCTGTGGGCAAAATTTTATTT

CTGAAATTAAAACGCATGACTTGATACATGCATGCTTGGAAAAAGACTGTTAAAATATACCAAAACATCTACAAAAGAAC

ACGACAGCTGGCAAGCAGTTATCTAGAATAAGTATTCCATCAATGAAGACTCAATTTGTTCAAACTTGTGCAGTTATTGC

ACCCCTAGAACACATTTTCAAAACAGTGTAATCACAGCTTTACAACTAGAAAACCTGGCAAAATACTTGTAAGCAGTATA

>CK175439_bb No definition line found

GATGCCTCAATGCAAGGCCCCGCAATTCAAGCCTGGTGAGCACACTGGATGCAGTCTCTTCTTCTGGAAGTGAAGGTACA

AAGGACAAACTGTCTCAGTTACTGGAAGATCGGCCAACTACAACCACCCTCCTCGATGCAGCCGCAGTTCATGACTCGTC

AAGTGATGAAAGTGGAAACGACTCGTTTACGTGCTCAGAGTTCGAATATGACAATGGATATGATAAAGCCCACCATCGGG

ATTTCCGCCCGGGTAATATGATCTTTTCCAAGCTTGCTGAAGAAGACAATGAGAATGACGAAGACCCATCGAAAACATAT

GATGGCTTTGACTCATTCCGGGGATCGCTAAGCACTTTAGTGGCCTCGGATGATGACCTTTCACACCTTTCTTACAAGCC

CCCCAGTGGTGCAGTGCTTGGTTGGGACTGCTTGCTCAATTGGGGACCGAACTTTGAGAACTTAGTGGGTGTGTTCAAGG

ACATTGCTGACTTGCCGGACACAGTCAGCCCACTTACGCCTGGGCATCCAAAGCCCAATGAAGAGTATGTTTGAGCTTGC

TCTCTATGAATACTCAGAGGAACCAGCTGGGTTGGAAATGCACAGATGCTAAAAGAAGAGAGTCAGGACATGCATTGTTA

TTCAGGCAGCTCRTTTCCATAAAAAGCAAACTTMAATGTAGCTGTGTAGTTTCACGCAACAACTATTAAAGGGAAGCCTG

ATAAGTTCAATTTTTGCAGGAAGTGTTRACGYGGTAGCCAGGCCAAYWTGTGGATGATTCAAACTGCATAGYTTTCAAAC

AAGTTGGGCTTAAAATCTTATAATAGTACATCTCCCGATAAACTTATCAAAGCTAATATTCATTACAGATGAGCTACACA

TGCAATCAGAAGAGTGTTTAAC

>CK175491_bb No definition line found

TTCTGTGCACGTTTCTTTATTCAAATGGCATACCAGCAGTGTTGCCCTTGATTTTGGAGCTGTCAATCAGCCCGAGGGCA

TATAAGGCCACGAGTGCAGCACTTTGCTCTGCATATTTCTTGTTCTTCTCTCTGCAAACATGCATAAAGGCTGTGAGTAA

TTATACTTAAATCAAAGCACCCACACACAAGCTGATGTAACCTTCCARAGATTCATTTAGTAGGCARCTAGTAGTGCAAT

TTATAAATGAACACTAACTTCARAGCTATACGGTGGACGTGGTGAGAAGACCTGGAAAAGTAGTGTGCAGCTAATATTTA

ATTTTTTATGCCAACATCAAAAGGTTACTGTTAATTTATGGTAAGATTCTCAGCATTTAATATAACTTGGGTGCCTATAT

GCAAAATTCAATTTTCTCCATTTCACTGAATAAAAACCAAAGGTTTCATGCTGATCAAGCGGTGTAGCACAAAAAATGTA

GCTATGAGCCAAAGCATTCCACACATACATAGCATATCCATTGATGTGCTGTCTCAAGCACATAACACATCTGAGAGTTT

TGTCCAAATTAATGTTTTCACATATGCATACAGGTGAATCTGGCTACATGGTGAGCTTAAGCAGCCCTCTGACACATTCA

TCAGTTTGCTTTTTTTTCTATAGCTAATCATATGGTAAATACGTAGAACAATACAAATTTATGCCTTTTAATGCCAGGCC

AAACAGTCAAAGTATTGAATGCAACAATGAGAAGAGCATTGATG

>CK175497_bb No definition line found

GTGTAGGAAAACAGGCAAGCAAATACCTGTTTGTCTCCGTTTCCCCAATAGAGGACAGGGTTTCGGACTCTGTCACTGCG

CAATAAACGTGGTTCACCATCAAGAAACCATGATACAGACGTTTCGCATAAAACGCGTTTATTTCACAGTAAACCACAAG

CACAGACATTGTACAAGACTACAGGATCAATATTGCTTTACGTGCTGGCGTTACTACTACAGTGACCAACTATCACACTT

AAAAAACGGAAAATTTGAAATCAAAGAGAGCATGAACACATAAACGCATACACACAAAGCAAGCAAAATATCTCGACCAA

GCGCGTTTTTCACTGTAAGCCACTTATTTCAATGCACGTACGACACTAGTTTGCACTTTTCACATCCACATCGATAAATA

CCAATCATCCCACCACAAAGCATACTCCCCTGATTATGTTTTAAAGCTTCATTGTCCCAGTATGAGCAGCGCCATCGCGG

AAACATAATTATTACATGTGTAAAAACAGAAAAAACCTTTTGAGAATGACGCTAGGGCCGCTGTAACAGAAAGTATGTAA

AATAAAGAAACTATACTGTTAGCAATAAAACAGTATGCACACAGATTCCACAGTCAGCATTGGTTGAACCAAATAAAGGC

GTACTGAATGAAACATTTACGACATTAGCCGAAATGGTGCTGTATAACTGAAAAAATGCGAAAGAACAGCGGGCAAACTC

ACTAACGATTCAATCCTAAACAAATGCTTTTGATAGCATTCGGCGTTTTACTCTCTAACCATCTCGTTCAATTTGTTATT

TTGATCAAGTATAAAAGTGCTCGCGCCCACAGCAAAATTTACCGATTAAGACCTGAACATAGATTTATAATTGAATACAA

CTAAATCTACTTACAGATAA

>CK175507_bb No definition line found

TTGYCAATGCRTACATACAACTGAGGATCATACAGAACAATRCACAATTGCCCAATGTGCACCASATTTTCAGGCTGTGT

AGTTGACTCTGTAAAAGGGATGSTCTACATAAAGYGCATGTTTGCTTAAATAATTTTTGCAAAAATGTTTATTGTTCTGG

TATCAATCATARAGCSCTATTTCAAGCGTGCTGTTGTCAGTTTGGTGGGCCTTGAAACTTTGCCTATTTCAAGCGTGCTG

TTGTCAGTTTGGTGGGCCTTGAAACTTTGGAATGCTTGATTTGACTTGCTGTGCTGGTGTAAACTGTGGCGAGCTTGAAA

TAGTGAGCTCTGTTGAATTGAATTGAATTGAATTTTATTCATCAAGCATGAACATGGTTAATCAGGTATTTGCTCTCATT

ACCATTCGTCATAATACTGATTCCATGTACCGAGTGTTTGTCAGACAGAAAAACTAATACAAATACAGCGTAATGCATCT

GATCTATCAGTTGCATGAAACATGTTTGCCCCCTCCCCCATTTTGTAATTGGCATTGCATAAAAATGTGTACACCCTCAC

GCTTCATATCAAAAGTTTGTGAGCACAGTA

>CK175510_bb No definition line found

CTGCTGTTTTTTTACTGTTCTTTTGGTGCATGCTCTGTGCATATGACTCAACKCAGCAWGAGTACCGGGCAGCGAATGAC

CATGGAAAATCACCAGTGCGCCTATGCAAGCGATTCATTCTGTCATKCA

>CK175556_bb No definition line found

CCTGTTATACTGATTCCTATACAGGGGAATCAGCTTTGTTTTATTTAAATATTTTGACTGAAGTCATAGTGAATAACAAT

GACCTTCCAACTGCCGTAAAGAGCTCTTGCGGCTGAAAGGAACATCATGTAACAGGATACTGAAAAGACACTGCAGCAAC

TGTACATGCAAGTGTGAAAGTACGTMAGCCGTGGGGAAAGGGCTTGCGAACAACTACATAGTTTGTATRAAAAAGACACT

TTGCTAACAGRCGTTTATGACATGAAACTGCAATGCAGCACTAGATMCAACACACACGGGTGAACGGCTTTCAGTGCGAA

CGCTGCCACRCCGTCTMGCRRAAATTYCTTCCACAATAAYARGGSCACGTGGCAACAGTGGGAAAACGAGATGGCAAAAC

ATTTTATATGCTACCGCCAGCAGCTTTAAAAAAAAGTAAAGTTCYGGCCATCATAGGACGACAGAAAAGAGCCGTTCTCT

GGTGGTAAATCTTTTGCGATGATGACAAACGGGAACCAGTGGCAGTGAGGTGTAAGTAAAACTACTGACAACAGGTAACT

GGTTTTACATGGTGACAGTCTAAAACATTCTATAAATATATATACTGACTAACACACTTTCAACGTAGACCACGTGGACC

GTATTTAACAACTGTATGAATGAGTATGCAAACGCTATCTCTTTGACGCTTAACGGAGACACTGTTAAACATTACAAAAC

AGCCGAAGAGCATGTTGTGCTGATTTAACCAGGAATTATTCGGTAGTAACTACATATAACAACGAACTTCTTTCATATTA

CTCTCTGAAGG

>CK175612_bb No definition line found

CATGTGTAATATTCCATGAGGCTGTGCTCTTGTGGGTACCATACAGAATCTTTGCTTTGTAATTTCTTGAAGCCTTAGAA

CTTGCCATGACGTTAAAGGGCCCCAAACCACCCCGAGGTCTTAGATTAGTTGTGGTGTTGCAGACGTAAATGAGTCAATG

ACGAACACATAGTCGTCAAAATTTTACGGAATGGTGCTATAATAGTGGAGTTGCACGCAATTGATGAGGCCCTCGTTTGT

TTCGCTCTTTCCTTCGCCATGCTCCGTTCATTGGCTCGTCATTCCTCCTGGCCAAGCGCTCGTCTCCACCATGCAAAAAG

GAAGAGTGGCAAGCTCATGTACCTGCGTGCAAACAGGTTATTTTTGATGACGATGAGCAGACGTGAATGTTGACGTCACT

TCGAGTGCCTTGGACTGCCGAGAAGAGCCGAAATGAGGGCACAATTTTTGGAATTTGTGGGGTACCCATGGCACGTAGCA

ACGCTACGTTTTGCATTGTTGATCGTGACAGGATTCTGGACTCTTACGTGCACATTTACTTGAAATGTTAAAAAAAAATT

ATTCAAGGCGGCTTGGGGCCCTTCAAGATTTATTTACACACCATTTTAGGAATTGCAGTATATTTAATAAGCAAAGTGCA

AAGTTTCTACTGACATTTGGGAAATTATTTTTTATTGCATGCGCTAGTGTTGTACAGAGAGTATTGGCTGGCACTTGCCC

TCCTTCCTAGTCGTGCTGTTGAAAGTGAATGGAGACTCTTGGAGCACATTTATTGTTCATATGCAAATTTGCTGTGTTTA

CTGTTGATTTGTTTATTTCACRTTGCTCTAGAGTGTCCCACACGTTGGGTTTACATATGAGATTCCGTGTGGGGGCTGGT

TTATCTAGGAAATTCTTTCAAGGGCTAGACTAGGAAGAAGGGCAAGTGCCAGCCAATTCTCTCTGTACAACACTAGCGCA

TGCAATAAAAAA

>CK175626_bb No definition line found

CGTGGTAACATGCACTTTTATTCAGATCATCAATAAATAAATAAGTGAAATGTAGAACATCTCATATATCATTTATTATA

TACAGTTATCTCACGCGGATACATATTTAGTTATCGCGAAGGAATATAACARCATGTTTCCGAYTACATAGTGGAAAAAT

AATACMTACTAATAATAATGCKTCCTTTATACAACACTCCATGCGGGGAACGATGGGGAGAGAGATTTCGACGCACAGTG

GCGCTGCGAAAGGAGCCCCCCGTAAAGAAGTAGGTAGTAGCATGTATAGGGAATAGGTACAAATGTTACACTGACTACAG

GCAGGTTTTTACAAGTTCACCTGAACAGCAGCTATCTAAAAGAACAATCTCACTTTCTCCCGACAGCAACTGTGCTCAGG

ATCAAGCAGATACGTTGCATTTACGAAGAAACATTGAGCCTAATGCACTTATCTGAGAAGTACACATACTAACGGCTTAT

AAAGTAACTATTTAGGAAGGGAGGGAGGATGTTCTATAGGTAAGGCGGTGCGTGGAAATGCGGCATAAATTCTGCAAGCT

TTTACAATGCGACACGCATAGGAAGGAGAACAGTCCCTGATGTAGCAGGTCAAAGGGCGTAGTAACATGTGAACGGGTTG

GGCTGCATTTCAGCCAAAAGGCGCCCTGGCAAATCAGAGTCGTTTTTCATTTTCGCGACACAGTACGTCACTAGAAACAT

GGAGGGGAGGGGGGGTTGTCTTTTTTTAAGTGTTGTATTTCCGTTGCTAAGCGAATTATTTTTTGAACGACTCTGGCTAC

AAGTTTTTTTTTTCCGAGATGTTACGGACTGAACAG

>CK175627_bb No definition line found

GATCAGAAAAGGCGCAGCGCTTTCCGTTTGAGCTGCGATGAATTGCGCCAAATTTTGGAAGAAATTCACAGGTATTACTT

GAACAGGTATACGAACGCTCGGTTCGCCCCGTCATCGTGGTGCAAGTCGTAAACAGTCTCAGTATCGTTTTAATTTAGTT

TTTTTTTTATATATCTCTGCATTCGTGTCTAGTGTCATGTACATACATATACATAGACAAGCAATGCACTTTGAACATTG

TGTGCACCAATTTCCGGGCCCCTATGGCTTCACGCATAAACGTGAATGTATTATCGATTTCTGTCGTCCCGATTGTTAGT

GGTACACATATATATATATATAGATATATATCGATGACGAAAAAACAAGTTGCGCTACTTTGATCATACCTGCCGTAGTG

GTATTCTGTACACAGTACTGTGTATAACGTTCATGTCACGTGCGAGAGTGTGTCGTCTGTCTGCATACGTTTACCAATCG

TGAGACCCCGTAGTTGGCMGTYGGCCGTCRSTGTATTTCGACGCGCTGTCTTCCTGGCAAACGGCTCTCTCTGGCGTGCG

TGTCCGTATCCGCCCTGTAGRAAAAACCATCCCGGTGACWCCACTCMYGTGGTATRCCAACCTTGTCTCAACCCAAGAAT

YAGTGGAAACRATATTTCGRCCTGAATWTTGCGAATCAKTTCCACGGAATGCCACAGYATGCGGCATTCATCTGTTTGCG

ACACAAAGTTGCGAGGCGATGGAGTTCATATGGACTCCTTGGAAAAAGTTTCTTATCGGGTTAGCCTAATCGGTATAGAG

CTGCCAACMCGAAAAGGCCTTGGTCGCTGGCTTGAATTTCACGAGTGGGCTATTTTTCCGWGAAMTAAGCGTCTTTSTTT

CTGAGAAATCGGGCCAAATGTTCTTTTGTCTACGCGTGCTGCAAACTGGCGGATATCAAGTCTCCTTTTTCTATATTGCA

ACCTT

>CK175793_bb No definition line found

TACTGTCACTCGGTTTCGATAGCTTCGACGCGCGTGCCTACGTAGTGCATATTTTGTTCTTTGTTCTGTGTTCTACTAAC

GCTTTATTTTAAATTTTACTTGTCCTGCGGCAACTGTTGTGTGAWGGCTCCAAGAAGCGACGTGGTGGTAACGCCTTGCT

TGACCTAAACTCCGTAAATACGAAGATGGGAAAACGTTACTTTTGTGACTACTGCAATAAGACATTCCCTGACAGTGCGA

ACAACCGCAAGAAGCATTTGCAAGGCGCGTTTCACACGCGGATGAAGAGAATTCACTACGATGCATTTCGTGACGCTGAA

ACGGTCTTTAAGATCGAATCCGTCAAGAAGCCGTGCCGGAGGTTTCAACAAATGGGTGCATGTGATTATGGCACAACATG

CAAGTTTTCACACTTAAGTTCGGCTGAATTAGTTCAGTTGGCAGCACGTGCAGAAGCTGAAAAGTATGCTGCTAAACAAC

GTGCTTTGGTACCCCTTCCTAAGAGTGTGTCTGTCACAGTGTGGGCTAATAGGCACTTCAAAGCTCAGGAGAAACTTCCA

GAGCCATTTGCATACAAAGATATGATCGCCACAGAATTTTCATTTTTCAACAACTTAAGCATATCTATGCGAGCACCTAC

TCTTGATGATTTGTTGGCGTGCAGGCCAAACCACTGGGGCTGAGTCTTTTTTTATCGTAGGCTGTACATGCATCATTGCA

ATGTCATTCAAGTTTATTTGAAGTTCTCTGTCTATGCAGCAGTGTCATTTGAAGATATTAATTTTTTTTTTAACATATCT

CTCAAGCTTGCACCTACGTTTTGTACAATGGACATTCTGTTCTCATCATGTTACAATTTTCCATAACCCTGTATGTACAT

TGAAAGTGCATTGAGAAAATAAATATTTCTTTG

>CK175803_bb No definition line found

AACAACAGGGAAGCTTCTTTTAACACTTTTGTCTGGACTGACCACGAGCTGCAGTGCCCCCCCTGAGTGTAGCAGGTATA

GTAAGTAGAAGACCTGCATTTTAGGTGCATGAGCAAAAGTTGCATTTGCATGCACTGGAAGCAGCGCTATCTAATTGTAA

CAATGGCACMAGGAGGTTTTGATCTTAACAACTCCTAAAATTTGTATCTATTATTCTGCTCCACTGTTAGCACACCTGGT

CTAAAAGAAAAAGAAACTAACCAACAAAAAGCTCTTGTATTGAAACAAAACTTGGCAGAACATGCTTGGCCGCTGTCTCA

AAAGGCTCAGGTTTAATCTCAGCCCTGGTGGTCCCATTTCGATCCATGTGAAATGCAAGAGATTCATGTGCTCAGATTCA

AGCGCATGTTCAGTAACACTAGGTGGTTGAAATTTTCAGAAGTCTTCACGACAACATGCCTCATTATCGTATTGTTGTTC

TGGTACGAAAAAACCATAGCCGTTATTACTGATGTGGAATAGAAATGTCTGTAAAAAAATATGTAGCAATTTTCGGTTGT

ATCTTTTGTTCATAAGTGTTCAAAGTGCTTCACCCATTGACCAGCATATTCAAAAAAACATTTATTTCCCATTTGCGGTT

CCTATGACTATTTGAACCAGATGGTGCATTTGCTGAAAAGAGGGAAGTAGTACTAATTGCGAGAACATGGACATAAGGTC

TTCATGGCAAGCCAAACAGGTGAGCCTTAACAGTGAAAGGAACATTTAGCGTAGAGCCCTCACATCATTGGTAACTTATC

CAGCTCCAAGATTTTTGCATCACCTTGTAACTTTCTGCGTTCAGAACTAACTTATCAAAGGCATTGACTCAGAAGAGGCC

CTCAGACGGCCTTACAAGACGTGCACTCCAACCTGCATCTGAAATTATCAAATCATGGTCCCACGCAAAAAGATGTTTTA

CATTGCTGGAACTTTCGAAAAGCTTTGAAAAAGCTCTCGAGACGGCTTTCTGCACCATCCAAACAAAATATTTCCTGATT

TGCATC

>CK175810_bb No definition line found

CCAGAAGAGCTTACGTGTTGTGTTCCACTAATCCTCCCATTCAAGGAATAGCTGAGTGAAATCGTGCTGGAGTCACACGA

GTAGCTATGAGAGCATTTCGCTAAGGTGCGCAGTAGCCAGCTGTCACACTGACCCGGCCGCAGAGAAGTGTAGGTTTTGC

TGGAAAGTATGTAATTACTGCAACAAAGTAGTGTGTACTAGCATATATATTATTTTAACTGCTCTATACGAGATCACAGC

TCTGGCTGCCATTACCTCTAAAACACAAGAATCGATTCAGACCAAGATAATCGATATACAATCATAGTACTAATGTAATG

CTGACAAAATACTCCACTTCTCGGACATCTGTCAATCAAGTTCTCCAAACGAGGAAAAAGAAAAAAAAGCCAAACTGCAC

CCATGCACTGTCCTGCAGAACATAACTAGTTTCAGAGAAAAAAAAATAAGCTTCCACAACAACTTTCTGTGCAATTGTGA

TGCTGTATAGAAAGCGTGTGTGATTTGTTTACGATTGTGCGTGTGTTGTCAAGTTTTCAGTCCAATAAAACTGAGACGTG

AATAAATGAGTTTAT

>CK175819_bb No definition line found

GTTTTAAATCTCGTGAACTATAAAATGGAAGCAAGAAGGCTTTGTTTTGCTTTACTATCCTTGTTACATGGCACGCAGCC

ACTTTGGGGGATTGGCCAAGAACGCACCGTAGAAACTGGTAAATGTTACTTTAGGTGCGCTATGAAATTTTAAATTAAAA

ATAAAAAAATAAAAGGGCGAATATACGTAAACTATTACACTTAACTGCAAGTAATATGCTTTTGAGCCTGCATTCCAGAC

TGCCKTAATAACCTGACGGGAAYAATTTAGAAACTTAAGTTTTAACCTAACGAAACGACGAAGAATTTTAAATACCAGAT

TAAAATGGCACTCGAGTATGTATAATGAAATTACACACGGCATCAAATGCATCCTTGTTGCAGTACCCCTAGCCTGAGGC

ACCAAAGATTAAAATATTTTCCTCCGATGAGGTCAATATACGACTTCATTGTCTTTTCCGCAAGAACATCTTTGTCTTCT

TTCGAACAGCGGCACATACAAGCCAGCCAGCATCTATAGAAAAAAACACTTGCAGAACTTGTACTAGTGGCATTATTTTG

ATCTTCAATGCTTTGAAATCGAAACATGGGAACCTTTCCCAGCAGGCGTGTCCCCTAATTAGGCTTGCATGCTCATCTCA

CAGTCGCGATTGACCGGCTTGACGAGTCCGATGAACGTCGCCGTTGAGCAGCACGCGAAGCAGGGACCTTCTTGTAACAG

CACTTCGTCTTGGCCACGTTTCAACCACGTGAGTCTTCGCTCCGAGCCATCGCTGCGGTTCGTGTGGTGCATGTGCACGC

ACTTCAGCTGGGTTGAGCCGGCGAAGAAGTGATGGGCACTTTGGGATGCGACGGAGTCTAGCACTTTCATCCAGGTCAGG

AGGCACAGGTGCTGCAGGCGGGGCATGCGGGACAAGCAGTCCTGCAAACTCTGATCGTCGATCGGCAAGTCTCCACGCTG

GAGTACGAGGCAACGTACAGCTTCGAACTTCTCTAGAAG

>CK175822_bb No definition line found

CGGCCTTCTGTTGGTTTACAGGGGTCTTGCTAGCAGAGCTGGTCAGGTATTCAATTGGCCCGGTTTGCTTTGTAGATAGG

TCTGTGAATGCCTTGCTCGCACTTTGTGCTGCACGCTCGCATGAGTTGTAGAGCAAGGCATTGGCTTGGACTAGYTGTGT

GGGTTCTGTGCTAGAAACAGGTCTGCGAAGATGGAACSAGCAAAGTGTTGTCGGCATTTATGTCGTCTTCTGGTCTTTTT

TTCTGTCTTGGTATGGAATTTGTGCACCATTCTCGAACTGCAAGCAGCTATCTCATGTCATGTTTTGTCAAATCCTTGGC

CATATTCTCCATGGAACACTCGTGAGGATGTCACATTTGTTTACGCTGACTGGTGGTCTTGCCTTCTAGTAGAGGGCCTC

AGAATAACTTTGAAAACTTGTGGACTCTCATTACACATGCCACGTTGGGCATACAATCATAATTGCATTCCACCATGATT

GTAATTCAGCTGTTGTGATGGGAAACTTGACCTGTAATATCACGCTTGGCAGTGCATAGTTGTCGTCATCATTAAGGTAC

TGTTACATGGTAATATTTTCATCTTGCAGAGGTTGTTACTGCATATTTCTTCGTATAAGCCTGAAGGTATGCCTCAGGAT

TGATAAATTGAGACTATGTCCGTCAGTTTGGAGTGTAGACCGCAAATGTTTGCAAGGCCTTTTCGTCTTGCTAATTCAAC

TTTGTCGAGATGTATGGAGCGTGAAAGAAGTTGGATGTCGCGCTTTGCAGATACATTTAGTTAGTGCTGGGCAAATCCAC

ATTCCCTTCGAGGCAGCAAAGGCACGCATTTTCACTCACCTCCGGTATAAACCTGTTGTACTTTGGTGTCTGGTGATTTC

CCCCGCACACGAAGTGAGACACTGTCGTATATTGTTGGACTGAGACATTGCTCGAGTGGAAGAGTTTCAACTTGTACACC

TAAAGCGGGTTGCTTTGTGGTCTGGTGATTTCCCCCGCACACGAAGTGAGACACTGTCGTATATTGTTGGACTGAGACAT

TGCTCGAGTGGAAGAGTTTCAACTTGTACACCTAAAGCGGGTTGCTTTGTGGTGCGTGTAATCCAGCCTATTGTGACTTA

TTTACTTAATAGTCTATTTTT

>CK175958_bb No definition line found

AATGTCATATTTTTATTAAAACAATGCAAAAATAGTTAAATATCAACATTGCATTTATGATGTAGCAAAGTGTTGCACTG

CATAAGTACAGCAGTGCCAGCTTTCATGAACATTCATCAGGCATAATGTATAATAGGAAAACATTGATCTGAAAAGCTAT

ACAGGTCCATTGCAAAACAGCAATGGCAGTAAACAGATCGAGCAACAAGGGATAGTTTTCCACAAAAGCAAAAACCCTTT

CTGCAAGGTACATCAAATAATTAAATTATATAGGGGGGGAAGACAGTCACACTATCACCACTTTAAACAAAAATATTGGG

GGCGGTTTCTACCTCCTAATAACAATGCATTTCATGCGCAACTTGAAATAAATGCTGTGCCATATGTGCTTGCATACCAT

AGCTTGTCCTTGTTGCACACATTCAAGCATAAACACTATTATCCACATTGCAGCAGTCCAATCCTTAATGAACCCACGTT

GTCACTGCTCACAGATTTGTGCATTTCAGCGATCGTAARAACCTGGCCTGCAACTGTCAAGGAATGAAAGCAACCCCCTG

AAGTGACAACTACAAAAAGTGTTCGTATGAACAGTTTTGGTGGCTAACGTCAGCACTGACTTTCAACAGCCACTATCAGC

ATCATTGACAATGCTGTTTTATTCTCTTAAGACCCTGCAAATGTTCCTTACATCATTAAACTTGGTGGATTCTAACTGAA

CACAAG

>CK175982_bb No definition line found

TAGATTATCCACGTCAAACAATCGCACAACCATGCAGGACGAGGTCTTGCTGCCGATTGATATCCAAACCAACAAGTTGC

TGGATTGGCTGATAAGCAGACGACACTGTAACAAGGATTGGCAAGCGAAAGCATTGGTTGTGCGCGAGAAGATCAATGGT

GCCATCCAGGACATGCCGGAGGTGGAGGAAATCACTAATCTGCTCGCGGGAACGTACATCCACTACTTCCACTGCCAGAG

GATAGTGGAAATCTTGAAGGACACAGAGGCCAGCACGAAGAACCTGTTTGGCCGATACTCGTCACAGCGGATGAAGGACT

GGCAGGAGATTATTCGCCTCTACGAAAAAGACAGTGTTTACCTTGCGGAGGCTGCTCAGATGATCATACGCACTGTCAAC

TATGAAGTACCAGCATTGAAGCGTCAGATTGCAAAATGTCAGCAAATCCAAGAGGAAGCAGTCAAGAAAGAGGCCGATTA

TGCCAAGCATGCCCAAGACCTGAGGGACAGGTACCAACAAGAATGCAAGCAGTTGGGCATAAAGGGCGAGTGCATCAAAA

AGGAGCTTCTCTCTCTTCTGGACGAGTTGCCTGGCCTCTTCGAAAGCGCCGTCAAGGAACTGCACAAGGCTCAGCCGGCC

ATTGACCTGTACAGGCTGTTCCTGGGCTTCACAGTGCCATCAAGCAAAGCAGAAGGCAGCAGCAAGGCAGTGCCGCTGAT

GCAATTCCTTATCACTCATGGCAATGTTACAACGTATCAGTGGCGGCATGGGGAGCCGCCGGAGGTGGTTGAGGAAACGC

TGCCCGTGTTACCAGTGGACATCGATAATGGCAACAGCGATGATCAGGTGACTTTTCTCTGGCTGCTTTTTTGTTCAGAA

AGATTTAATGTGTTTGCTCGATAGCTGATAAGCTGCTTTGTTCGTTTCTTGTAAGGGAGAGTTCAT

>CK176066_bb No definition line found

AAAACACCACTCTAACCGCGACATGACGATTGGTAAGCGAGAAAACGTGCGAGAAGAAAATGCAGGTGTAGGCACCACTG

AGAGATTCTTGCACCATTGTGACTTCGTAATTTTTGGGGACGTCTACTGGGTCCTACATAGCTTTTACTCGATRAAAATT

ATGTACATTGTCCTCTGTGGGTGCCATCGGCATAGCACTCAATATTTCARAAAATTTCATCGAGGTAATGTCGTGAAAAT

ACAAAACGTACATTTGGGGATTTGCTACGTCATGCACGAATATCGCGGCACGAAATTTAAATATAAAGCTTTAACCTTGA

TTTTCATCGCTAATAATGAATTTATGATTGTGAAACATGTGGCATTTGAGTTCTTAGAGTCCAGTTCATCAATCTAAACC

AAGTCATTGTTTCTCTTTAGTGTCCCTTTAAGCTCGGTAGTGTATGGGGAAGAAAGGCCCGTAGAAAATCTTCCTCCGTT

GTTCCGAGCATTGGATATTAGTAAGATTGGAAATGAAGCTTCATTCTTGGTAAAGATTGTCGCCATTTTCTTTTTTTGGA

AGCATCTGATCTGATGGGCATTCTGCCTTTTTTTCGCGGTAAAYGCCTTCTCTGCTTGCACAATGTACTCGCAGTTCTCT

GTGGCTTGCTTCTGCACAAACAGTGCGCATAACTCTTGCTTRGTAAGGGCTAGTATTATTTAGTYTCTGTGTTTCATTTG

TCAATRATAGTTGGGTTTGGGGTTGCAAGACAAGTACAAAAAAAAATTTAAAAAAGCGCAATCACTTTGTTGAGTTTCGC

TGTTGACACACTCTCGACTCCGTTGCTAGTGCTTGGGATTAGTGTCTAAGTGAAATGGATTTGCGTGTTTAAAGATTTAT

TATTAGTCTAATTGGTGGGTGGGCATATGTTGAGAAAGCTGTTAATCAGAGCAGTGATTTGTTTTAGCTAATATTCTGTT

CACACATGGCTGCAAGCTTGACAAGGTCTCGACGTAACTAAMGGCTGGTTTGTTTTACAAACAAAACGCACACTCTGGCT

ATCTGGTGCCTGGCTTTGGCTATTGCTTTGCATATATCGAGTGACCTGCCTATAGCAGTGCACGTGCACAAGCTTTCGCT

TTAGTTTTGTCTTATACCTACTGGTGTTTTACCGCACAGATTTTACCGAGGGGCACTATTTGTT

>CK176115_bb No definition line found

CATCACTGTCTTCACTTACGAACACAAAATAAATAATCTGTATTTGAATCATCATCATTCAAAGATGGTGAAATTAAAAG

CTCTTTTTTGTGTTGACGAGCCAGCTATACATGGGTCGCCSCCMCCSCAAAYTATCCKTCCATATGAAAACCGTGCTTTT

GCCARAARARAAAACTGAATTGTCAAGTAAATGCTGCCATCTTGKGGATGACATGCAGTCTAAGCCATAAACGAACGCTT

CTCGTCCTGAACGGTACAGGGTACTTGTTCATCCAGGACAAAAAAAGCAGTTCGGGAACAGTTTGAGCAAGGGGAGCTCT

GCTCAATCAAAAAACTTGAGGGAGTAATGAATGTCATAAGGTGCAACTTCAGTTTTCGTTGAGTTCAGTCCCAATGCACC

CATTTGTGCAAGCTACACGAAAAATCCAATTATGGTTTTAAAATTTGATTAATATCAAACTTCATTACCTTCGGAAAGTG

GCCATGTGCCAGTGGCATAAGAACCACTAATTTATACAGTGTC

>CK176167_bb No definition line found

TTTCGTGGCAAACATGAGTGTACATGATTTGGTGTTTTCTTTTGACAGCTCATCAAGGAACGCTGCGAGACTGAAATATT

GGCAATGGTGCGCCGGACAAGCCAGGTGCTTCTTGACACAAACAATGGTTCTGCACAGACAGCTTTAGCTTTGCAACCAC

GTGTACTCCTAGACTTGTTGGATCTGCTCTTTGAGCAATTTCGAAGTGTGGCCTCTGGACATGAGTGTGTTCTGGAGAAC

ATGCATGCTGTGGTGGATACCCAGGGACCTGAGAATGGTGTACCAATGTACACTTTGGCTGATGTGTGGGCCAGGATACA

GGCTGTGATGGAATATGTTGTGGGAAAGTACTTGGACATTCACAATACTGGTTCGGCACAGCAAGTCCCACCACCTGTAT

TCACTGAAATGACAGCAACAAATGACATTGCTTCTTACTTCACTCGGAGGAGAAATGTTAAGCAGAAAAAGTTTGCACTC

TTTAGGTTTGACTGCTCTTCACATGCAATTAGCATGAATACCTACTTGAAAGAGAAGAAGGAGGAAATGAAGGACAAGTG

TGATTACATTGAAGTGCCAGACTCACATCAGCAGTTGGTTTGTGTGCCTACTTCCAAGAACATCACATATATTTTCAAGC

CGCTAATGAACTTTATTACGGACATAGAAGCAGAGCTGAAACTTGAGGATGGAAATCACTGCCATTTATATGAATACCTA

GTTGACTGTGTGAAAATATTCTTAGGACATGTGAACAATGATCTGGACAACACTATTGATAATGCCACAAAAAGCTTGGA

GACCTGGAAAGTGACCTCCGATGCAGAAGTCTTAAAATCTCATGGGGTTATGAGGCCATTATTGCAGAGCACATTGTGTG

TGGATCAAAGTCTTCAAGG

>CK176238_bb No definition line found

GAAGTGTCCAGTTTCGCTAGCGTCATTATACTCATTAACTATTAAGGGGAGTGAATTATTGCATGCTGCATTTCGTGTAG

TTTGTCTATGTGTTCCACAATGTGCAACTTTTGTTTATGCTGCTAGTTGTGTACAGTACGATTGGCCAGCAATGAAGCCG

ACGTGATGCGGACGCTTTCTTCCTTGCCAAAAGGTAGGAATGCCTTGCATCACACTGTTAGCTCACACTTGTGCATTGAT

AATGGCCTCCATGCCTCGATAAGTTTATTACAGCTTTAGTTGGTAGCTATTATTGTGAATGCTTTCATTAAACCATGGTC

TACATAATTCCTTATTCATCAGAAAGCACACTAGATAAGCCAGGCTGTAGAAATTCGCATGTCTGCAAAACTTTTATACA

TGGTATTATTAGACATTGTTTTTCTCGTTTACATCATATCTGTCACTGCTTTAGACTGTGACAAGCATTCTACTTTGAAA

AGATAGCTCAGTGAGAAATACGAGAATTGTGCGAACTTGCTTTTTTTTCTATCTCTCTCTCTCTCCCTTTTTGTTGACGA

CAAGTTGATCATCAGAGCGTATTGACGTTGCGCATTCTATTATTGTACCCGTACTGCAATACTTCTGACAACCTGGGGAC

ATTTCTAATTTTATGTTTGCCTATGCCATAGTGTTGTTCTATCTTTCGAAAAACTCGAGAAGATTTTTGTGTCGCTGAAA

TAAGCAAACAACCAGGTGTAAGCCGATGCTGCCAAAGTCTGTGACGTCATGAGTAGCTGGTGCTAGAATTTGAATATGCT

GTTACTAGTAGTATTTTTAATATCTATAACATTTGTGGTATGCTGAAGTTCGTACAGCAAAGCTAATCAATATARGAACA

GCCGCTAGCTCAGGAGCTTGATACACTGCTGTAATGTTACACTTTTTKKTAAAATCTTCTACTGCACATTTGTTTTATTT

GTTTCTCTGAAACAAAGTGCCTTTGGGAGAGTACTATTTTTCTAAGATATGAAACTTTTGCATCTTTCCCAGAAACTAG

>CK176290_bb No definition line found

TGACGTTGAATGCCAGCGTTAGAACTGTGACAAAGTTTCTAAGGTACGAGTGCACATAAAGCAAATCAGGGTGGCACACA

TATATTGAGACACATTTTTTTTCCGTGCCTTTAATATGGCATTCACTTGACCCCTTGTTTTGTTTTGGTATTTAACTTTA

TTGATGTACTCGCAATGGTATGCACAGCTAATGCATACSASAGTGGGTCCTTGCTTTTGTGTGGCGCTGATAGAATCGAG

AAACTGTGTAGAGTACCACTGCACAAATGTACCAGAAGTGTCAAAAGAAAAATGAGAACAAAAAAAGAAAGTGAAAGAAA

TGATTATTTATGCCAGTGTTGTCTGTTCAATGTTGGGTGTGCGAGTTCTGTGGCTTGTACAGATCGAAGTGCCTTGATCT

TATTTGTTGCCTCATCCTTGTATGTTTCTTTTTTTATTTTTCACTTTTTATTCATCGAGAGAAGGAATGGCATTTTTGGC

CCTTCTCTTTTCTTTTTTGTTGTGTGTATGTATGTGGCACTGCGACTTTGTGCAATTGGCATAATGTGCAGCAAGGCTTT

TTGAGGCCAAGTGGGTGCAAGGCAGGCAGTTGCATTACACATTGTTGGAGTTGTTCTGCTGGCTTACTCATGATGTTGTA

CATTAAACTGTGACTTTGTAAATTTATCATGCTCAAGATTTTTGTACAAGAAATGTTTCCTGTGAGTGTTTTTATTCTTA

TTTCTGGAAATTAAATATAGAAAAAAAAACTTGTGTTGATTATTTATGCACATCAGTATCTTTAA

>CK176381_bb No definition line found

TATTCAGACAGGAAAATGTCATTTATTATACATTGCAAAATATTCTCCCCTAATTTGCCAAACAACATCAATAATTGTGG

CATCATATAAAACATTCATTTGTAATACCACTTTTTTTTTACTTTTCATGATTACAATAAAATATTCGCTGACTCTTTCA

AGGCACATTCGTGGGTACAGCTAGTACAAAAATGTACACTTGGACTCGCAAAAATATGAAAATTCTGCTGACATGCACAA

TATGATCAGTTAATAATAGCAAGGCACAGATGCAAGTTTATTCAACAATTGTTATCCAAATTATTACCTTTGCACTCTGC

AATTAATCAACACTGATCGACAATAATAAGGCACATGATTACACTATGAAGTCAACAATTATCTCGATCATCGTTCACAC

AAAAAGCTTATAAACACACTTTCTCACAATTAAATAAACGTATGTCATGCATAGCACAATAAAGACACTTATACATTCAA

CATAAATAAAGATGTTTCTATTTACAAGCATTTCTCAATATTAAATCCTTTTCAGCTTGTAAACATAACAGCACCAACTA

ATGTATACATAACTGCTCCATAAATTCTCTGAGCTACATGTGACACACCACAGTTGAGCAGCCTTAAAATTATTTATCAT

ATAAGGTACACAATGACCAAATGTGTCGACTTCACAAATGTTTTCTTGAGCACAAGCTTTCTCACTCTAAAAAAAAGACA

GATCCTGTGTCATAATCTTGGGAAGTTCCAACGTGTGATGCAGAGCTGCATCAGATGCAGCCATGAATCTAGGGGTAAAA

AAAAAAAAAAGATGATTGAGCACGGCTTGGTTCTCTTTCACACAATTATTGCTTGTCCCGGCTTGTCTTTGATGTCCTGC

CAGATTGTCTTCGCATACCCCGCCGCACCTTGTCTTTGACAGGTGCCTTGCTGACGCTTGAAGCGCTCTTTTTGCCGGCA

CCAGT

>CK176416_bb No definition line found

GATCAGAAACGGGCTTGTTTCCTTTGAATTTATTCTGTGGCAAGTTATTTAGACAGAACATTGTGAGACTACTAGACTGA

TTGCACCCATTGCAGAAAGCAGGGCTCTGAAGATAGTGGTAATTAGTACCATTATATTGCCTATGACCTCTATTGGCCAG

AGCAGCTTGCTCATTTACATTTTTAGGAGCCCCATTGTCATCTTTCACTGTTCTTGTGGGTTTGTTGTTAACAGAGTGCA

GTAAGTTTTTTTGTGGGCAGTCTCTTTAAAGCAGTCAGTTGTCTGCCTGTTTGTATTCTCAAACCTCATTATAACAAAGT

TGAATTTTAAATGAAAATAAGTTTGGTGTATTCGAAAATTTATTGCAAAAGTTTATTCCAAACACCGTATCTATTGAAAA

ACTATTTTTTGTTCACTTCGTTATAACCGATATTTTGTTATGTCAGGGTTCATTATATTGAGGTTTGAGTGTATAATGAA

GTGTGTGAATGCACATTGCCTCAGTGACTTGTGTGCCACACCTTTTAAATAAATGTCTTTAGGCATGCAGCATTCGAAGT

GTTCGTGACGTAACGCGCCTTTTGCAGACCTTTGCTTTCATCCTCGTGGCACCAATTTGCCCACTGTGCCTTGCACTTGT

GCCTTGTAAAGTGCGCTATTACACTTTTGTAACGTGAAAGTTTTTCCGTCAGCTCTTAATGGCCTGAGACATCGACGAGT

ACTATACTGTTCACTAGATAACGGCTCGTGAATGATGAGGTCATACATGAGCTAAATCTACTGAACAACCGTAGGCCATG

TCTAGTATAGCTACACTTTGTAATCACAAAAAACTTTTTTTATTTTTGAACAGGTAGTTTGAAAGTGCATGACACGAATT

TTCGTGGGCTGAAATGCAAGTTCAATAGTCTTAATGTGTTAATAAGGTTGTTGACAAAGGGACGA

>CK176419_bb No definition line found

GGGCCCCAATGCATGTACTTTGTTCATGAAATAAAATGCAGCATTAAAACTTTTAAAGTATAACGACTGGCATGAGCTAT

TGCTCAAGATAGTCATGCCACATTGCTACAACTTTGGACACTTATGGAGATTTTGGCTCTGATGCGAGTGTACAAACCAA

TAGCGCTGGKGTAACAAGAACTGAACACAATGGAAATGAAGGKATGGRCTGKGCTTAGCCCTAAGTTGATGCATTT

>CK176443_bb No definition line found

TTGATAGTGCTTGATGGGGACTCTTTGGCACTCAATAGCCAATGCATGGTTGCAGTTTTTGAGATAAGTTGGATGTCAAC

TTGAAAGAGGTGTTCCAGCAARTATGCTTCTGRAAATTGTGTATTCAAGCATCCCCAGAATATTTTTTTAATATTCTGGG

GATGCTTGAGTCCTTCAAAATTGGCTTTCAGCATGACTGTCATGTATTATYTGCCTTTTCCATAAGCGTTGGCCACGTTG

TTGGTTTGGTGGTGTGACWGATGGCACTGGTGTTCTTGAAGTGATGTGGCAAGCCTACTTGTGWACTATGACGGAAGGGT

CTCTTCTTCATGGGTTGGATGTGTACTTTGTTAACTTCTTACATGCTTGACAGAAAGGTTTGCAGAACTGTCATTTGCAC

CTGAACTGAAGTGAACACTGCTGAAAGGATGTAGAAAGAACAAAAGGTATTTTAAGTGTACATCATCAGCAAGAATGACT

AGTGTGGTGTTGAGTTCTGTATCAGTGAATTCTTTTTGTCTTATATGGCATGCGTGTTAGCCCCACAAGGTGCTTTTCAT

AGCATTGTATTAACCTTCAACATCAGATAAGGCGTAATTGCTAGTGCGTCTCTTTCCTGCCTTTCTTACTATCATAACAT

TAATAGTACACCCAAATTTYGYAGATTTTCAGTTTGGCATGGCATCCAGACGGTGTCTTATTGGCCACCCTGTGCAAAGA

CCAGAAGCTGCGGGTATACGACCCTCGAAGGAATGARYTAYCTGTGSAGGAGGGCCTTGGACCAACGGGAACTCGTGGRG

CGAGAATCATCTGGGTGCTTGGAGGAAGTCACCTGATCACCACTGGCTTCAACAAGTGTGCCTTCTTTTTTTCCATCATA

ACCTGCAATGCAATACAGCAATTTGGCCAAGTTTGACATGGGGGATATATGGTGGATATAAGAGTAGCTCACCACTTCTC

TGCATCAGTAAAGCCTTACAT

>CK176519_bb No definition line found

CAACAGGTAACAATAGTTGTGATCTCGTCAACAAGGTACACACACCGTTGAACACAAACGCAAGTGCAGCAAACGTTTTC

AGATCGCGCAGCGTTGCGGCGGCTGAAACGAGCGCCCCGGGGGGCTCCTCGCCTTTCTCGTTCGGGGGTGCTTTCTTATT

TAGAAAAAAAACCTGCGAGTACGTTCTGGTCCGTAGCGKGACCAGGTAAGCACAAGGACCGTGTACCAAAAGTGTCAAAA

TGGAAAATGAAAAACGTTCGCTTCTTGTCTGTGTGTGTGCGTGTAAGYTTTAGCAAAATGCACGTAGCGTCCGTAACGGC

RCTRMGTACGCTGTAGCTCACGTRCGGCCTACRAAAAAACGGCATTAWTATAAGTGCCTTCCATCATGGCACGAGCGAAA

ACTTTGAGTGCGATGACTGCAATGGACAGCTTGCACGTGACTTCACAGACGCAGGTTCTTAACACACTGGTGCTCCGTCA

TGACGAGGTTTTACGACGTCGGAATATCGCAATGACGTGGCAGTTAAGCATCTACAATGTATTAACGATGCCAATGTAAC

GTTTTCTTTTTGGCTAGTGTGAGTGATTAAATGAAGAACATGCGTGCAACGTATTGATTACGTCAAAGAGACATCGCAAC

TTTCGCGACTCATCCTATTGGTACTCCGGCGTATTGGTTTCTGTGACGTCAATGCAAGCCATCCATAAGCATATTCAAAC

CACAGCACGACATGCC

>CK176555_bb No definition line found

TTTTTAAACGCATTTAGAATGTGCTCACTTCTTTGCACAGTGCAAAGAAGTGAGTCACTTGTGAATTAAAATAAAATAAT

CACTGTACCTAAACACTGTAAAACCTTTCTGCTGTTTTGTTAAACCCGATCATGGACAATTAATTGTTAATCGTTGAACA

AGTAATCATTTATAATTATGGATTTGGTGTTCCAGCTCCCACTGCTCACGATAGCACGTTTTGACTTATGTATCGGCATG

TGATCATTCATTCGCGTGCCGACTGTATCATTCCTTTAGCATTTAATGTGTAGATATACATGCAGGTGCGTTCAGACATG

TATAGTATTTTGGATGTAATAGTCAACATTTGGGGATGCCTCTTAATTTTTTCTTTTTTTGTTTCGGTGCTCCCACGGCA

CCAACGAGCAGCGGGTGATCGAAAACAGCTGGCGCGTTTGACCGATTCAACGTACACACTTTTCCCATAGTGCTTCCCAT

GCCTGCGAGGGCTCTGGGATTGCTTGAAAATGGTCTATTTGTTTTGCACTGAGGGGACTCGGCATGCTCATATGGTTCAG

ATGTGCTAAGGATATTTATGATTAAATTGACACTAGATAAAACTGGTATCCCATCTTGGTGAAGTGCTTATCCTTACCAC

TTTTTCTGTACAACTCGAGAGCTCTCCTAACTGGTATGTGGCACATTATATGAGCATTCGAGTTTTTCTTTAGAAGGGCT

TGTATGCTAATTGTCACTATGTGATTGCGTCATTGTAACTGTTGTATTATATGTTGTATATTCTTTTTTCAATGTGCATT

TTATGAAATGCCATAATCAAGGAAGTTAAGACATTTGCTTAGGGAGCATAGGGACTCGGGTTTAAATCGCACTAATAGAG

TGAAAATTT

>CK176656_bb No definition line found

AAAAGATAAAATGTTTATTTCACAGAGATATACACATGACTGTAGTTCATTCAGCCACTAGATAACCTTCAAGCCCAAAA

CCAACAAATAAAAGGAGAGACATCCAAACAACTATGTCACATATTTTCTGTGAGAGGTGCAGCTGCACAGACAYCATGAG

TTTGTTATCAGTTGCTGCACTCATTCAGCATATTCAATGGCATGTGCAGCATTGGTTTCTCTTAGCACTCGCTCCAATGA

TCAAAACAGAGGGCTGGCACAGTTATACTGAGAAAGTTCTCAGCAGTTTTTACGCATAATTTGTAATGACAGCCATTTAA

GGAATCAGTAATTAAATAACACACATAAGCAATCTTTAACTTATTTTGCATGCTTGTTTGATATGGCAACATAATCTTTA

AATGAGGCTTTAGCATGAAACTTTTTACCCAAGAAATTGGTTCGAAATAATTTGCTCATACAGACAAGTTTACACTTTAA

TAGCAGCATARAATTAGGATAAAATAGTCGGGCTTTATGAGTSAGATTCTTRATCTCCTTAGGCGGAAAAGTGGAGGTGC

TGTGGGTCGATCATGAATATGCGGGATTCCTTACAGTGCACCCGATGCATAAATGTATTTGATTGCAGACATTTGACAGA

TGTCTGTCTGGTTGGGTAAGTAAACTGGGAAACGATGTTAGCTCCATATTTTCACATACTTTTTGTTTGGAGCTACAATC

ATTTCCCATAAATGTATCTTCATTTTACCTTTTTTGGAATGTGGTCTTTGTGGCCTGTATCCAATGCATGACTTCAAG

>CK176657_bb No definition line found

GATTGCAGAAGGAAAATGTTAATCATACTATTTCATACTAGTGCAATTATACTGCACATATGCTTGCTGCCAGGGTATCG

AGATAGCATCTCTATTTGCTAAACGAGGTTCTACATCAATAATACTTTGGTTAATGTCTTCCCAGTTCTTGACATTTGCA

AGATGTGTACCCTGCATGCTGTTCTACATAGGATTGTGATACAAACAGCTGCAGAGTTGTTCTGCAAGTTCATCCAAGAA

AAATTTCACTTCATGAATACTTTCAAATGAATTAGGATGCTCAAACCTTATTATAGCAATGATGCATCTCACAAGAAATA

TTGTTCGTTATATCCGGAAATTCGCTATGAAGGTTATTTCTAACACTGTACCTATTGCAAGACTATTTTTCATGCACTTC

ATTATAACCAATGTTTCATTTAATTTGGGTTCGTTGTATCAAAGTTTGGGTTTGCATACATAGGCAGGATTTTAACGACC

ACCTCACTACTGTGACAACTGGTCTTYACAAACTATTWTCAGCCGACWAAAAAATTTTTTTTTATYTAAGGTGAGCAGTT

TGTGTCCACTGCTTCCCMAAGCCACTGACACTGAAATGGTGTGACACTTTCTTTAAGGCTGCTGTGCCAGAAAGCTAAAA

TTTTGAATAATGTTCTGTACAAAATCTAAGCAGGTGAATTTAAAAGGTGACAATTTCCTAAAAATGCCTCATACTGGACA

TATCTTGTAGCCTGACGAGCTACACTTTTGTTCACTTTGGAAGTTGTTTACCATCAGTGCAGAAATTTTCTGTCAACTCA

GGCCCATGCTAAATGCTTAATTGTTTACTTACATGCAGCAAGACCAAATGGTGGTTGTTCAATGGCCATACTATACACTG

CTGAGCTTGAAGTCATGCATTGGATACAGGCCCACAAGACCACATTCCAAAAAAGGTAAAAATGAAATACATTTATGGGG

AAAATGATTGTAGCTCC

>CK176660_bb No definition line found

TTTAAAAAAGAAAACAACTATTTAATAACACAATATATTACTCGCTTATTTACATCATTATCTGCATTCCCATTGGTAGA

GAAGTTTGCTCCATTATATATACAATACTCTGGGTGTAGCACCATCTTCTAGCTGCGAAGCAAAGCTCACTAATATAAAA

CTACAGCTGATAAAATTGTTAGGCTTGCTAAGTCATATATAAACATTTTTAGAGATGAGGTAGAAGCACTAAAAATATTA

AAAAACTAAATGTCATGCACTTCTGCTTTCTAAGCAAAAAGCAGGACCTCAAAAACAGCGAAGGTATTGTTACAGGCGAA

CAAAAAATACAACGCATCAAAATCACTATGTAAGGACTATTCGAGGCCACAGCGTTTCATAAAAAGGTAACGTATATACT

GGGGGACTTCGTTCAGTGGAACATTACACACTTGATTAGCGTTAAAAGAAAACAAGATAACACATACATCAGCACAAAAT

AAAGTAGACTAACAATACACAAGAAAATTGTTCGAAAAGTCTTGCAAATAACAAATGCACACACACACGAGAGAGCTTCC

TTGATACGAAGCGTTGGCACTATTCATGTCCAAAGGCAGCCGGATGTGCTAGGGTTTCTTGTGTGTATTATCTTGGTGAA

ACGTTTTGTTGATATGAATGAAGGACGTTTCGGCTTTGCTCTCCACCACCTCTGGAGGCACTTGACCACTGTGAGATGAG

GCACAGGTGGTGACGGTGATAGCTCAATGGACGAGGACAATCCACAAACCGGTTCGCATATAGACAGACCATCATATAGT

CCTGTAGGTCTGTGTTCTTTCTTTATGTGCGGTCTGTGTGTAGGGCCAGCCAGCTGTACGGTTTTCTCACGGTGGTCAAG

TGCCTCCAGAGGTGGTGGAGAGCAAAG

>CK176678_bb No definition line found

ACCTGATCTGCTTTCTCACCAGATCACTTTTAGAAAGGAACAGGAATGTTTTTTTCATGCAGAGCAGTTAAATGCGGTAT

GTGAATGGGTTGCAAGAACTTTGCACGACATTGAAAGATCCTTGTCCCTACAATTAAGCACAATGTTCTGTTGTGTTTTA

TCATTTGAAGAAAGAGCTTGACCCACGAGGTCAACCGAGCTGTGGTCCCTCGTTCTCTGAACTCTTCTTGCGGTGGGGTG

ACACCGGTGAATCAGCAATGAATAATTAATACGATTGTTGCCTAAAAAATTTTTCATTGTGCTGTAGAAGCCTTTCATGT

GGCTCTTGGCTGAGCCAAAGGGACCATTTTTTTTTTTACTTGGGACCAGTGGATAAAGGAYATTCTTCGAAGTGACTCTA

CAATGCAGCACTTGCATATTGTTTTGTTCATCTCTGGCCCACTGAAATYACCCAGYGCTTTTGTTTCAGGCTCATAGTTA

TTTAAACAAACAACTTTTGGGTAATCAGGTGTTTTGTCTCTTTTTCCACTCTATTTGTGTCTTTATTTAATAAGATTTAT

CTAAATTGGGTGGTTCTCATGGCACAAGGCACTATTCGTCATTAAATGTTTTCTAGACATGTTAAGTTCATGATTTTTAT

CTTTTGCCTCATTGAACTCATTGTTAGGTACAGTTTTGTATCGCATAGTGGTCGTAAAAAGTCCGAATATTGCCACGTGA

CAACCTTTTTTTACGGTGCCATCTTGGAAACCACCAGCACTGGTGTATTAAACACACGCACAGTTCATGATGCTCCAGTC

TCATATTTTACAAGGACATGTATGTGAGTGAAGGGGTAAAATAATGAATAGTGTGTACTATA

>CK176692_bb No definition line found

GATCACCCACCATAAAGCCTGTGACGCCCCAGGTGCCCTGGTGGGTCAAGGGAGGTCCCAAGGTACCCGTGACGCCCAAG

AAGCAGCCACCCCAGCGCGGCTACCCCTGGAAGACCTATCCGACCCGCAGGCCCTTCCACGAGGTCGAAGGCCACACGAA

GCAGGTTCCCGCCGCCGCCGTGCCCGCCGAGCCCAGGCCAGTCTACCCCGGCGGCAAGCCAGAGGTCGAAGGAGAATCCG

AACACAACGGCCATGGTCACCATGGAGGCAACGATGGCACACCGACTCGGACCTACCCCGAGGAAGAGGAGTTGCCTACG

AGTACCACAACAGAGTACCCGCGCACCATGATTCCCAAGAGGTACTCTCCTAAGGACAAGTGGACTCCAGCTCCGAAAAA

CACCAAGAACAAGCCACCTTTCGCAAAGGGTGGTCCCGCTTACAAGCCCRGCAGCGGCAAAGAAGTTTTCGGAGGCTGTA

AGGGCGTCGGCATGTATAGGCGAATCCCAGGACTCGACAAGTGGTGCTGGGAAAATTGCCACAGGGGTTATTGCCCGCCC

ACACACTGCACTTGCCACTAAGTGTCTCCCGGTAGAGACAGCAACATAAGTTTGAATTTTAGAGTATCCCTTGTTCTCCC

GTTTTTATCCCTTGTTGACGATGTCTTTCCCAAAACGTGACCCCACCCTTCCCGTTGCTACCGCCTCTGCTTCAAACTCT

GCAAAGCCATCATTTTTTTTTATTCTTCACAAACTTCGCCCATGATCGTGTGCGTGAATACCCGGTGTCTTCGAGTTGTG

TCCCGGTGACTTGCGCTTTTTGTGTCCCGTTTTTTGTCTSTTCATGCACACCACATCTGCAGAGGGAAGTTTGAGCTGTA

TGATTACGAACGCGTGGTTTTTATTCGGGTGTGAATGCTTGTTGGCTATATTTATTTGTACGCTTGATGCAATATTTGAA

ACCCGGTGACGAATGATTATTGATTGGTAATATCTACTACTTGAAAGATGTGGTGGCCAGCCTCTTGAGTCTAAAAAATA

CAGGGCAT

>CK176697_bb No definition line found

GTGGGTCAGCATTCTGGTGACTCATCTTGGTCTGTGGGACTGGTGCATTTTTCCGAAATGCTCATACTGCTGCTTCTGGG

CCTCTGAAAACCTCCTGTAAGCACGTGCCATGCAGGAAGCCTCCCAAACGCAAGCCCTTCTGCGAGCAGCTGCCACCAGG

GACGACGGGGCCCCCGCTTCCGTCTCGAGTGCCGTCCGACACGTCGAGCACGTGGAACGAGAACGTGTACGACGACGTCG

AAGTGCCCGGCTACCGGAAGTCTTACCTTCACCTGCGCGACAAGGACGGGCTGGCCGAAATCTCCTACGAGTCCGTCCCT

GAGCTGGACGACGTCGAGATGACTACGTCACGGGACGGCAAGAAAGGCGACGTCTCGCGCGGCGCGGAGGGCTCTGCAGC

GGCGCACGGTGCCAAGCCCAAGGAAGGGGCCGACTCTGCCAAACCCAAAGATGTGGTCGGCTCTACCGTACCCGCAAGCG

AAGAGGAGCACATCTACGAGGACATCGATGAGCTCCATGCAAATTAACGGTGGTCTGCAGGGCTCTCTTCAAGATTTATT

ATATTGTCCAGTGCGGGCTTTAGATATATATGCGCGTGTACCCCCAAGAGGGCGTATTTGACAAAAAAAAAGAAATTCCT

AAATTTAGTGACGAAAAGGCTGTGCGTTTACGCAATGAAAGAATTCTATCGGAAATCGTARAMGCCGGTGTGCTTTTCTT

TTGCAGCGATTTGAACATGGCGTCGACAAGCTTCACCGCAATGCATTCAGTCTATTAGA

>CK176718_bb No definition line found

AATGGTGTTGATACATTACTTAATTGATTGAGATTAATCTATGAAGACTGTATTATTTGCTTTTGCTGTAGCAAAGGAAT

ATCAGTGTCTACATGCACCAAAATACTGAGAAGCAGTGGCATGAATGTCAGGAGCATTTTTACACTATCATCAACTATAG

AGCTACGTAAATTCATTTCCATTACAGGTTTCTTTGTAGTAATGATAAAGAGGTATATATTGATTGCGATGCCAATTACA

GACTTATATGTAGGCTTATAAATGAAATGCAGTATATTACTGCAATAACAGTACTGTATACTACAACATCACAATATGTC

TGTGTAACCACTGCAACATGCCACATATTCACTATTCTTGTGTGCTTCTTTGCTTTTGTTGTTACATATCTGCATTCTAA

ATTTATGTATTAAACTTGCCGCAGTCACTTAATACTATAACAGCATTATGCCTGCAGAAACCTGCCTGAGGCACTGGAGA

GAACGCTACAGAATCAAAGCACGCAAAAGTTTCAGTGCTCCAAGAAATGTTGTAAATTCTTATCAGTGTTATTTTAACTT

GGGAAGAAAAGCATAAATTGGTTATTTACGCCAGCAACATAAGGCAAGGTTGATCCCCAAATTGATAAGGCTCTAATGTT

TTTCTTCAATGTTCTGGCAAATATGTAACAGCCATACACTACGTTGACTCAAAATACTGTTCCAAGGAACTAGACGTCAT

ATTCCGCAATAATACCTTGCATTGTACCTCAGGCGCTGCCTCCTCGTAGCTTGCCCTTTACAGCTGCAGAAAGGTGTTTT

TAAATGTTGCCTTGGTACACTGCCTTGCATAAACTGTACAGGGTGCTTCCTGCAACCCTAGTTRGCACTGCCAGATAAGA

ACTAAAAWAAAAAACTGCGTCTATTGTGCGAGTTCTACTAAAAAATAAAAATAG

>CK176841_bb No definition line found

GCAGTCTAGTGCGTGTACCCGGAYGAACAAAAGATACGAAGATAAATAAGGGCRAACTGATCAGTAATGAGTACTTTTTC

TTTGGTGATTCCTGTACTGGCTGTGTTTCGTCMCTTTTAGCGTTCTAGCTCAGTTGRAAAATGTAGCAGAACACGACGAC

ATAAAAATCGGATGTCTTGTCGTCTTTTGGGACATCTTTTGCTGWTGTGACCACATAGCTGGTTGAAAGATTTCTTTTGR

AAGTCTATGTGCCAACATGTTTTTGTGGCACTACAAAATAATTTGTAAGGCACCATAAGAATGTTTCTCTTGCGACATCA

CATTTTTCGCTGTTTTAAGAAAATTCCTTTTTCTAGTTCAGGTCTCTATTCTAACAATACTCACGTGACAGAATTCTTTC

TGTTGTTAGGACAACAAATGTTTGTTGCCCAATGGACTAGGCTGACACCGCATCAGGAAACTGAAAATAATTACCAAAAA

ATCTGTAGTAACAGCAAGTTCTGTGGTCATTTTCAACTGGGAAATGTTAACGCCCCCCCTTCCCCCCTAAACAAAATTGA

AAGGGTTTAGTCTAAAAGGCAGTGGGCTGAGACTCCCGTTATTTTTTTTWWTGTTGSTCAAGGTTACWACAGAATGGCGC

ATAATCTTAAAAACACACACAGTTGTACATAGCTGSAGTATCGCTATYACACAATAATGACACCGAGTAAATCACTAATT

AATTTATTTTKCTCTGTGAAGTGATCCAACCTTGACATCAGTTTTTGATGGCTTTTGCTTTGAGGCTCTGTCAGAGACGC

TGTAGATAAAATCGCCATTTGWTTTATATATARTATACTTTTCGTTKATATCRAACCTGCTTCTGCCTAAGAAACTACTG

ACTGTTTTGTTTGCGGGGTGGTCGCTCACTCGGATTAGCCACACCAAGGAACTAGTACTCGGCTTGAGACAAAGAATATG

GTATGATGTAGCGTTGTAAGACTTGATCTAGGA

>CK176910_bb No definition line found

GTTTTTTTTTTAGCCTCTTTGGACGAACTTTTTGCTCGCGTCAAGATAGAACGTGTTGAGAGTTATGCGCCAATTAACGA

CATAGCATGGCATTTATAGTGTTGTCCGCCTTCCCTAATCGCGTGCTTCTTTCTTGCGTGTGGTTTACTCTTCCGTACGC

TGCGTCGCACAATGGACCAATCGACTTTAGGACGTTTGCAAAAGGCGCCTTTTGCTGCACTGTCGAGGGCGCCTTTTTTA

CTTGTATGCAGTAAAAAACTGAGCGTCATTTTAGATGATTTGACCAATTCTGGCAAATGCTGTCTGCAAATCGTGTGTTA

TTTCTGGCCTAGTCATAAGAGAAGCACCGGTAAATGTCGTCTTGTCCTTAAAGTGGTGCTGGCACAAATTTCGAAAGGGG

AGCTTACTCTGCCACACATGTCTTCTGTATACAAGGACACCCGTGTGTATATGTGAAGCTCACCCGTAAATGCTGGTATT

ATATTTTGGCAATAAAGTCCATTCCTGCTTGGCACACTCACTAGCATCCACCCTAGCGTGATATCAGGCTACTATGTCGA

TTATGGTGACTTCGTGCACCAGTATCGCTAGTTGTAAAGTTGACTATGGCTAGTTGACGTGATGCTCCTGCATAGAAATA

GAACGCATCAATATTTTTAGTCATGCTCTTATTGTGAAGCATTCAGGCACCAAAACATTCAATACAGCCGCTCGTACATA

ACAAGAAATAATAAAAATGGCCGCTCGTTTATAGCCAACGGAGTAGGGAATCGAGTGGCCGTAGAAACATGGCAGTGTCT

TGCCCAAGCTCACAAGAATCTCATGCTTTGTAGTGAAGTGAGGGTACGGT

>CK177058_bb No definition line found

CACCTGCCTGGCACCATTCGAGAGTGACGTTGACTTAGTCGACAAGGTCAATGGTGTGCCGTACGGACTCTGCGCCAGCA

TCTGGTCCAAGGATATCTCCCGCATACACAAGATGGCTCAACGTCTTGAGGTTGGCACCATATGGTGCAACTGCTGGATG

GTGCGTCACCTGCACATGCCATTTGGAGGTATGAAGATGTCTGGCGTTGGRCGGGAAGGAACGGAYGCTTCCAAGGAGTT

CTATACAGAAGTGAAATCSGTCTGTCTAGACTACAGTTGAGGCATGGTGGCTGMCTAAGAATGCAGCACGGCAATAAAAA

ATTCAGGCTGGACACGTCGGGACGTACGAATTAGTCATGGCATGAGCAATCTATGTAAAGCCTCACACTAGTCACGTTTT

>CK177092_bb No definition line found

CGTGTTCATACAGCGAAGAACAAGAGAGTAAACATGAAGGCTTTTGTGGTCTGTGCCCTGCTTGCAACGTTCGCCGCTGT

GAGCTTCGCCCACCATCTCGAGCTCTGCAAGAAACAAGACGATCAGCTGAGAACTGAACTCCAGTGCATTGGGCTGCATA

TCTCAGCAGAGGCGAACCAAAGTTTTAACAAGGCATTGAAAACTCTCGGCTGCAAAGACTGGAGCTGCGTTATCCGGAAG

TTGTGCGTTGGAAACGACCTTGAAGCTGCCATGGCGAACCATTTTACGAAATCTCAAATCTCGGAAATACACAGTGCTTC

CACAGTTTGCGATCCAGAGGCTGGTCACCACCATCACCACCACTGAAAGAAATTCGCTCCAAGTAAATTGAAGAACTGAG

CCCTAGACGTGTTGACAGTTCCTATACTGCTGCAAACGTGCTTAATCACAGGCTCCTGGCAGCTTCATGTTCTGGCGTAG

CCTTTTATAAAATTATGACGAAGACAAATAAACATTTGTTTCTCTGAACATTTGTTCAATCTTTTTAATGGGCGCATTGC

TTCACGTAGCCAGCTCTTAACTTGAAAGACTGAGCTTTATATCAAAGATCAACGAGGGCAGAGGTATATTGCTGACAGTC

GTATTCACTTACTGGAAAGAGCTTTA

>CK177096_bb No definition line found

GCACGTTCGTGCTTTATCTTTGTTCATTATAGTGATACATGGCCTCTAAACGTCCCAGACATCCAAACCAGCGTAACAGA

CAGTCGGTCAATGAATATTCACCAAGCCGTGCCGCTTCGTCGAGTCGCAATGCAGGCGACACCCGACAATCTGTCGGTSA

TCCWCCACCGRTTGACGCAAAAGAGCGCTCCAGTGAACAAGCTTGCTCAAACATTTGCGGCACGAAAGGCTGAGTTAAAA

AAAAAGCGAAAGGTTGCTCTAGCAACGGAAACTTCCGACGAGCCTTTCTATCTAAGTGCATCCCCGTCGATAAGGCCGAA

CAATGGACGCTCACAAGACGCAACGCCAGCAGGTATAAATGTGGTGCTAGCCTATGGGAAGCAGCGATACATTTAGTGTG

TGTTTCCAGCCGCAGTGCATCACTGAAGCCGTAGAAAGCCTGGTGTTTTACTGTTCTGGGAGAAGAACTTCAACCAGCAG

CCCCACGCGCAGTTGAATTTACATACACAGAAGAAATGAAGCTGACGGGGTCAGTCGTCTTTCTGGGTGTTGTTGTCGGA

GCCTATGGAGCAGTTTTGAACTCACATCAACAAAACCAAGATGAAGACTGGGCAAAAATACATTACCGCATATGGGGGGA

GAGCCCTTGGGACTATCCCGGTAACCCGCCACCTATCTTTCCTCCGGAAGACTTCTGGCGCTACTCCCAGATTGGTCGTT

GGTGGAACCAGGAAAGGCCATGGGAAAACCCTGCAAGGCCTTGGCAGGCTGCATTCGCTGACAGCAAAGATGACGATGCT

AACGAATACCGTCTCTGGGGCAAGTTTCCATGGGAGCGCCCCGGAAACCCACCTCCTTTTGTTCCACCCTSCCGACGTGA

TGCCGTTCTCACAGATTGGTCGCTCGTGGAGTCAGGACGAGAATTCTGGAAGGCCTGAGAGGCCACTGACGYAMGTGTCA

MTTTTAAGTTACACCCCAAGAGCATTTAGTGTCATCTTTATAATTGCAACTGTGACGATGCTATAAAACATGCTTGAATA

AAATGTGTCCAAATATGTATGCTGTGGGGACCTTCAAACGGCAGAATGTTAGTACAACGTA

>CK177125_bb No definition line found

AAAGAATGAAAAAAATTAACGTGGGGCTTGTAACGTAAACATAACACGTCCCTTCACGTGCTGCACGTGGAACAAGGCGG

GGCTCGCAAACGCTRGTTCGYTTARGCAGCACAGCTTGCCTTCTGGGAATGTAGAGAAAAAGGTGAGTTGAGGTTCTCCT

AGCATGTCGACAAATAAACCATTTTGAAAATTATTTCCCTGGAATGGCTTCCCAAGTGGAACTTATCAACTTCCAGTGAT

TAACGATCGATTAACCAAATTTGAAGTGTTATCTGGTGAGGGGCCCTATAAGAGAAAATGCTGCTCATTACAAAATGATC

>CK177143_bb No definition line found

CTAGCTTTTTCTGCCTGGAATAGCGGCCATACCAAAAAGAACACAACGCGAAAGTATGCGCTTGCTGAGCTTCAATTGAG

GGCCCCAGAACTATATATTTAGAAAGAAACGCTTAAGCACTGTTCACATAGTGGTGCTTCACTGTAATGAGTTGTTGTAG

TAATTTCTATAGTCATTGCAAAATAATTCCTTAGCCCTAACGAGCACATCACATTGACAGCCAAAAACTTTAATTAGTGT

ACGTAACTTATTGCATGAATATACACTTCCGTGCRGTGMATTTGCARGTGCTTTCTCATACTCGGTGTCATGTCTTRAGA

GCCGTTTGAAGAAATKCACACCGAAGCATTTGTGAATGYCTGTYTTAAACGAATGCTGCTTCGTCTGAATAAGCGCCGGT

GTATTTCATTGCAGACAAGTTTAATGTCTTGGGATCGACTTATCAAGCTTTCCTGTGGTCTTGGACAGCGAACAAGTTTT

CATGCGCTTTTTTGTTTGGCCAACCAAAATGTAATTAAGACACTAATAAAGCGATGAACCACACATCGTTTCCGCACCTA

ATGTCATCGCTGAAGCTGTCTTCAAAAAACAAAACTATGTAATGCAGCTTAGTACAGCTCAATCGCTAATTTATCAAAGT

CATATGGTGTCACTTGCCCGGTCAGCCTATCTTGCATTCCCCTTGTCTCTTGTGATCTCTCGCTATTTCTTTTTATCGTT

ATCTCTGTGCAAAGTTAGTC

>CK177151_bb No definition line found

TCTCATTTGTGGAAAATTCGAGAGCCTAGAGCTGTTCTTAATAGACCTTTAGCAGACTTGCAGCTGTCCACTTCACTTCA

TTCCACTCAACTCWGTCAGGTTGCTCCCGACTACAGRCGAACGTGGCGAACGGCAAGCGCTTCAGCGCCAGCAARGCATT

CATTCAGCCGGTCATCCGTGAACGGAARAACCTGCACGTGGCGCTACTCAGCCACGTCATCAAGGTAAACTTCGACGGTA

AGCGTGCAGTGGGTGTGACCTTCACCAGACTTGGTCGGCCGGCCAAGGTGAATGCGAGGCGAGAAGTGATCCTCTCCGCG

GGAACCGTGGGATCTGCACAGCTCTTGCTGCTTTCCGGAGTTGGGCCCAGAGATGACCTGGAACGGCTACAGATACCGGT

GGTTGCCGATCTGCCAGTGGGCCGCAACCTTCAAGACCATCCGGCGATCCTCATGGGAGTTCCCGTTAGCACCGACGTGG

AAGTGGGCATACCACCTTTTAGTCTCGCCGACATACAGCAGTACGCCAGCAACGGGACAGGCACGATATCGATACCTGTG

GGCAACGAGTTTGTGCACTTCCTGCCCACCGAGTACGCCGAGGATCCAAATGTCCCGGATGTCGAGGTGGCGACCATGGC

CATGTCGCCGGCCAGCCAGTTGACCAAGGCACTCGCCGTGGAGCTAGGATTGTTGCCCGAGGCTTTTGACGATTTAATTG

GGCCCATTGACGATCGGCCCGGGTTTCGAGCAGCCATGATTCTCAACCGACCGAGGTCACGGGGCTCCATTTCGCTGCGC

TCCAAGAACCCATACGAGCACCCGAATATCGATCCGAACTTGTTCGAGCACCCTTACGATGCGAAGGCACTCGCTCAAGG

TACGAAGATGTT

>CK177158_bb No definition line found

CACTAAGTGGTACACTTTGATGTCATCACGACACCCCATCGTTGCTTTAAGTTCATTTKKACCGCGCAAAGCACCAGGAA

GCAGTTGGGACATTGCAGCTGGCATGTGCTACGTTCMCTCGGATGAACATGGGAGCAACTGCACACTGGAAGCCATCGCA

GGCCGGTCTTTTGGTGTCGATGACTGTTGTGCTGCGCCTGTCAGAGGAGCTCCTGGCGAGGCGTCGGTACTTGTACGTCC

TCACTTCTAGGCTGATACAAGATTGCCTAGAAAACATTTTTTCAGTTGTGAGATTGAAGAAGCCAGTTCCAAGTGCGTAT

GATGTTAATTGTGCACTGAAGGCCATTTGTGTCAGCCAGTTTGTACACACCCCTAAGACTTCGAGCTACAACATTGACGA

CAACAGTCGTCTGGCTGACTTGCTTGACCCAAGCTTAAAAAAGCTAGAGGAAGACCCCATTCAGCAGGCTGAGGAGTTAG

AGAACTTGTTTGCGATTGGCCTGATGGAAGCAGAGTGCGACATCGTAGCGTACATTGGTTGTTTCCTTCTGAGGTCTGTC

TTAAAGTTTATCGACAACTGCAAAAGCTGTAAAGCAGTACTGACTGACAGTGCTCCTGGGAAATGCAGCAGCCTCATCCA

GTTGAAGGAATACGTAAAAAATGCGGGAAATCTCATCCAGCCAAGCATGTCTGTAATGACAATTTTGATGGATTGTGAAG

AAAACTCCAAAACTTTTGCTGAAATGGGTGAAGTCATGAAGGTGAAAAAACCATTTTCAAGCATTCTAGGTGCCCTGCAG

AAGTCTGTAAAAATGCACTTGGGATTTTGTCAAGTTTGAACAAGCCTACTGTAGAGAAGCTGCTTCTCGAAAAATATGTC

CGAAAAAGACTAAAGATTTACCTCAGGCAGCAGCATAAACACAGAGTAAATGGGGCAGCAAGCAAAACCTGTGCTGCGGA

CAAACTTGGAGTAATCCAGCAGCCAGGTAGAACCGTAGAGCGTACGCAGCTGCGATTTATTTTTTTTATACGAGGA

>CK177169_bb No definition line found

TGAACTGATATAAAGTACAAGCATGAGCAGTCACAATAAAGTGCAAGTATACCACACACAATGTGCACGTTTCCCCAAAA

AAACTGGCACACCTATGTGGTATTAAAAGCCATATGAAAAGTGTAAAGTATTGTATAAAATTTTCAAAATATGCAAAAAC

GTGATTCAAAAAGCATGAAAAAGTGAAAGGCACTAAAAGCAGCTCAATGAAATGCATGACATGTATATATGATGCATGAC

ATTGATAAAATGTGGCATATTTTGCAGTATAAGATAAAAAGATGCCATTTGCTCACACGGCGGTACACGAATAAAAGTTG

ATTAAAAACAGTGGTGTGAAATACCCATGCCAGCTTCACTGCTGCAGCTTGTTAGTTACACGAGTAACGAGACCATTAAT

ATCAGTCTCCTTCACAATTTTTACATATGTGCCACGCCGGATGGTTAGTACCTTAAACCAATCAGGGAGCACTTTTGAAG

CAAGAACAATAAGGGACTCGACTTCAGCCACTTCCTGAATGGATGAAAAACTTTCTTTGATCTTCTGCACAAGGTCATCC

TTTGGTATAGCTGCCTTCTGCTCAGCAAGGAAGAGGGACCGAGTGATTCTGATGAACTCTGGAAGTTTAGCCAGTTCAGC

TCGCTCTTTTTCATC

>CK177196_bb No definition line found

AAATGTCCTTGTTTGCCTTTATTTGCAATTTATTAAACTATTAACATATCAATAAGTAGAGCGTTTTCATGGTTAACTTA

CTTATAATGTCCGTGAAAAAAAGACACACGCAATATTCGTTCAGGTCAATCTATGACGCTTCTGAAAATGCTGCTACTTA

CGTACGCATTTGCTCTTCAAACAGCATTATTTTTTATGTATTTAATTTACTTTCCGGCAACTTGGAAGTGAAGTCAGCTG

GAGCTTCCTAATTAGGTAGGCACAGTAAAATTCTGTGTCGACCATCACCTGTCTGAGAAAGCTGCTTACGCCATTTACGT

TAGCAAAGCCTTACTTGTCCTGCTAGAACTTTTCACTACTCGTGAAGGTACAACATATCGKGGTCTTCACGCGGATGTCT

AATCAATTGATGTCGAGGCTCGAAGCGACGAATAAAATTTAAGGATGTTARTTGAAGGGATAAACAAAGCTGCGTACGAG

GCACGCTCACGCTCACATTGGCCCCGTCGTACCAACATTTATAGCGCAGAACGACCGTTAGTGGCCGCCACTGGGATTAG

CAAAAAAAAGCTCACTGCGCCCCCACTCAACGGTTGAGAATACTTTCACTACCTTTCCGCTACAGGCTTACCGATCGGAG

AAATCAAACTCTCCAAAATTTGCCCCAAATAAGACTATCGTTGAGACATCATGGCGCATTACTGAAGTTACGAGCTACCT

TTGTGCATAGGGTTACCGCTAGTAAACACTGACAGTT

>CK177215_bb No definition line found

ACGTTGCAATGTGCACACGCTAACGTGTACCTTTGTTGACTCTCCCTCGGCTGTACATGGAATGCATMTCTCTTTGTTTT

ATGTCGTTTGTCCTTTCCTTTCGATGGTCTTCGCCAGGGCATAGCAGTGATATTACYGACCCTCTTTTGGKACTCTCATT

CCGGAAATGTATATGTTTAGCATGCTTCTGGTGACCGGACGTGGCAATTTCCGCGTTCGTAATAGACGAACGGACATTTA

TACACTTCTACCGAGTATAGCTGGTCGGCCACGGGCTGCATAACCATACAACAGCGAAGGTGACTAATATGAGGATGTAG

AGCTGGAGCTAGCTTTCCGTAGTTAGTCCTTGTGTTACTTCTGATGTAGAGGAGTTCTCGTAAGCTTGATAGTGCAGTTA

TTACATTTAGGAAACATAGTGAAGCGCAACGAAATTGTAGGCTAGGTGCTTCAAAGGGAACCGGTGACGTATAACGCAAG

CACTTGTTTCCTTTTTGTGCTTGTGCCGTACATCACCCATTCATGTCGAACTGCCTTGGTGTAGGCGTGGTCATGGTATC

GTAAATGTACCAATAATACTTCGGTATAACCGAGCGTGTGTTTCACAAAGTTGGATTAAAGAAAGTTCGCTTGAGT

>CK177216_bb No definition line found

TTTCATAGAGTTACGCCAGATCAGATCCTAACGAAGTTACTTATGTATAACAGGCCAATGCATTCACTACTTCGACTAGC

AGAAAACAGTTTATTTTCACCCGTAATCCAGCCCAACTTCTGTCTCAAGCACATCTGCAGAGATGCACATTGAGAGAGTC

AGAACGTAGGCACWWTARTTATCAAATACATTTATTTGTTTACATTTGCTGTACAAATACCTCTCAGTACCTTACCTATA

CAAAGCAATCGATTAAAAAAATGGCGAGACTAGCACTTCTCACCTTGTCTCGCGTATGTACAGAAAAAAAGTGGGCCCTC

CCCTAACCTGCTTACATTTTGTAAAAACTCCCTTCTACACAGTGCCCTTAAGATTCTCAACTTAGACACTATTCAAGACC

ATTTTCATTATAACCTCCTGGCACATATGCACCTCTATGCCTTCGTACTTTTTGTTCTATTGGAGCACACCATACAGCAA

TAGTTCCGTGCAGCAAAAGGTAGCTGCACATTCCTTATAAAAGACGTACATGCACAGCCGCAGTTGTGCACATTTTGCAC

GTTATACGTACGCAAAAGATGCAAAACTGCGACAATGCATACTCTCTTGGCAGAGCATAAAAAAGATTCGATTTGCTTTT

GAATGTGAATAAAAATTGTTAAAAGAAACATTTTTAAACAACGCCGATGTAAATTCTAGTGACAATCCAGCAAAAGCCGC

AGACAAGTCTGCATTCAGCTGACACTCGGTGAAAACGTTTTTCTGTGGTCATTGAACAGTGCTTCGCAAGATAAAAAAGA

CAGCAAGAATCACAATATTGATCGACCCAACATTAGAGGACAAGCCAGCTATGCTTATACGTCG

>CK177217_bb No definition line found

GCATTTTCCACACTCGACGAACTTCTCGTTTTCTGTTGTACGAGACCATACTACCACGTCTTAATAGAAGTTTGAAAAGA

AGTAATCTCAACACATGTACTACTATTGATAATGATGCCCAACATCTGGATATCTATCTGGAGTTCTACGGATATGTTTT

TTCCTGGAAAACGTTAGAAAAAAAAAAAGAAAAACCTGCTGTACAAAGGTAGACTAGCTTCAAGAGCAGGGTGGATTCTG

GGTTTGTCTTCAATATGCCCATGTACTCTGTAAAGCGTAAACTGCGACAAACACGCTCCCGACCTATTTTTGGCATTTCA

TTGCACGGCCTTGTACTTCAGATGACCTAGGCTGTCAGATGACATAGCTCTATTGTCTGCCAAAATGTCTTGCATGATTA

TTAACTTGAAACAAAGCCCCACTGTATAAATGCCTAGTTCACTTTTACGCTGTACGTTGTCACACTGAAAGATTGTAGGA

AATATCTTCTCTCCGATGTAGTTCTTCGTGGCTGGCTAGCTTAGGCAAGTGCGTTCAGCACTGTTGCACACCTCTTTAAC

ATTTCTCTTCTTCTAAGGTGAAAGGGGGTAGTTGYAGCGAGCTCTCATTGAACTGGCTACAGTTCGGCACCTTAGTCTAG

CCTTTACATGCCTACAATCTAAAGTGTTGGCTGAATGTGCACAGTATGGTTTCCTACGAGTCTGTTCTTTTTTGTGCCCA

GAACACAATTTTTCTAGGTTCATTGTAGGATRGGTGCATCGTTTAGGCACTAGCTGTTCTTCCCTGTCTGCAAAAGTCCT

CTTATCGTCACTCTATCATAGTTTTGGAATGTGACTCGGCAGTTTTGGCAAAAGGCTCTAGCTTTCACGCAGTCAGACAA

AAGTGTTGTACACAGCAGTACTTAGATATATACAATGAATGTTTTATTTT

>CK177234_bb No definition line found

AGTCTTTGTTCTTCTGTTTGAGCTGCTTGCCTTGAGTGAAAGACGTTTTTCAGCCTGGCATAGTGGGCAGTCTGCTGTAG

AAGAAGTGTTGAATTAGCTCATGTGACACAAACATGTCAAAGATTTCCTCCACAGCTGCGCTGCCTGCCAGCGTAGTTTT

TCGCAACATCAGCTGTAAATTGTAACGTATCAGTGTTCTGTTGTTTYYTTTCTCCCCTCAGTGGAGTGAT

>CK177339_bb No definition line found

ACAATACTATTAAGTGTGAAAAGAAGGCATTATACAAAAACGTTAACTGATGGTAAAATCGCTGCTTTCTACTGCCGTCA

TGAAATCTGTGCAGTGCGCACAAGCGAGGGTGCCTTAAAGAGGCCATGACACCCAATTTTCCCTTATAGTCTGTCTTATC

TAGGTTAATCCTTGGGTCCACACAAACTAGCTGGCAAAAATTCAGTACATTGGTTCGAGAACTTAATTTATAATGAGAAT

TTTACAAGCAGCCYGAAAATGTCGCAACAGTGATGCACCCTTGAGCCGTCACATTACAAGCTGCTTATTTTGCAAGCGTC

TATTCCAGCCAGCGATAATGTTTACTTATCAACTGTGTGTACAATAAAGCTAGTAAAACATTCTTTAGTTTGTCATTGAA

AATTGAATGGTTAGCAGCAGTTGAAATTTGGGTAGAAGATGACAGCTCCACGCTATGAAGCACATGATTTCGCCCACTTC

ATGGCAAACTGCCGATTACCGCGGCGGGTGGGGTTTATAGCTGGCAGCTTCCAGGGCTAATTTACCGATGAACTATTCTT

TTGCAGTTCATTTTTCATACATGTACAGGTACAATAGATCCTCTACTTCTGTATTTTGTTTAAAACCACTATTTGGTGGG

TGACCGTGTCATGGCCCCTTTAAATGAAAAAGCAAGATATGTGTGTACCATAAGAACTCAATTTCTAATATGCATCGAGG

TGTCAAAATTGTCTTGAGTATATATTTATAGAACATTCACTAAAGCACTGAAAGTACATGACACGAGGAAAATGATGGCG

AGGTAGGTCAGTGAGTCTTCTGTCAGTCTACAGTTGAACCTCATTTCGTCCAACACTGATAGATCGTATGATTGGTGATA

ATGAACTAAATATAAGTATTAATTTTCATTGGCCATGCTTCATTTTAGGGACACGATGTTTCTAATGAAACCGAAAATCT

GAGTACCTCCACTATATTGGCTGTAATGAATAAATATTTCGCTTGTGCGAGGCTTAAAACTCAAAGGGTAGCATACAAAG

CAAAGACAGTTCACAACCGCATTTCTTTTGAAAATGTGGTTGTGGCAGTCAGAACTTACATAAGAAAATTTGTCGCATTC

TTTTT

>CK177354_bb No definition line found

TTTTTTTGTTTAATTTCTTCAGTGATCTTGATCAGGATTATGCAGCTTGTTCTGCTGAWTTCAAATATTTAATTAGTTTT

CCTKTAACYCAYCTTGTTKCTGARATAACTTAAGTTCACCACCTATTGTTTAAGGCCACCAATGAAAAAAAGTGCCTAAT

TAAAAGTGATATTTAAATTATGCCAACATAGACTAATGACTAGAGTGAAAGTATCGAATAATTTTTTTGTTTTTAGGCCT

GTATTGTTTATTTTACAGCAAAATGAACTATCCATTTTCAAAAGCAGTTTAAGTTGTGTTACAATTATGATTAGGTTTCC

CCTGCAAAGAAAATTATTTTCGTACCTGCCCTAATATTGAGGCCTGAAAATTGAACAGTCGCAAATTTACTTTTTTGAGA

AAGATGGAAAAAAACTGAAAACAGTTTCTTCAAAAAGCAACAATCATGGTGCACATGTTTTGCAATCCTCATAAGGGCAT

TCATAAATTGGTAGTAGAGCTTGAGGCACAGGTGCTGGCAAATTATAAAGAAGCCCCAAAGTTGGTTTTCCATTTTTTTT

TTCAGTTTCTGCATGTTAACAGCATTAAGAATCTGGACAGAATGATATTACAAAAAAATTACTACTTCCTGGTGCGTGAA

AATTTGCAGTGAGATAGAAAAATACTTGCAGAAACCAAATATGTCAATTTTAAAAAATGAATTTTTTTTGTCACTTTTTG

GTCTCGGAAACCAGTCTGCCCCCTTAAAGCACATTCACACTTGGCATCAAAGGGAGAGAAAGCAGCAAAACTCATTGAAC

ACATGCTT

>CK177370_bb No definition line found

GATGTCCAGCTGTGGAACGTGCTAATGGGCAACAGGAAATGACCCTGATGTACCTCCAGGTGCACCCATACGTTTTTTTT

TTCTTTAGATGTCACCGTCGTTACTTTGAAACTCGGTGGTGTCACGTAGGGCATCTCGCACGCTCCAGCCTTGTCAACAA

TGAAAAACAGAAATACGCAATCGAAAAGAAACTGTCGCTGCTGGACTGCCTTCATGAGTTGCGTTCTCAAAGGAACCACG

GGCACTTATTTGGAAAGTGAGCCACGAAACGTCTCACTCGGCTGCGGCTGCCAGAGCGAAAAGATCGCGCACGTCGTCCA

AACTTCACTGAAGGCACCGGGCAGGATGAAAAAGTGCTACTTCCGCAAAACGTCCATTGTCGCGGTGCAGATTCGCGGGC

ATGTTCATCGAGACAGTGCATTTACGAGGAAATTCGAAAGGTGCGGTTGAACTTGCTGCGGGCTTGGCTGCTGTTTTCGA

CGTGAAAATATGTTTTCAGTCTTGCCATGCGGCTATTTCGTCGAAGTCACGAGGTGCTTTTTTTGGACCATACATGCACA

TATATGGTCCAGACGAGCCGAGCATCGGGTCCACGGCTCTTTGAGGAACAACGTTTTTACACGTTTTTTTTATAGTGTGT

TGTTTGATTACTTTTTTTTATCCAGCGTGCCTGTGCAGCGTGCCCGTGCGTGAATGTGTGTGGCAGTTCGAATGCGAGGT

TGAAGAGTGCCGCAACGTGGAAGGTTGCACCTTCCGCGAAGGATTAGGAAAAGGACACACATGTTGGTTGCGAGTGCCCC

ATATGCAGCAACGACCGAGCGACAAAGATGGTKTTCTTGTTTTATTTTTATTTTTTCGTAACGTGCGCGTTGAAACTCCT

GTGAGWCTCTTTGCGACCAGCCTGACTGTACATTTTTTTTTAGGAACCAAGCAAACACCCCTATCCGTCGTCGCCACTGA

CTTCCGCTACGTGCGTATAGCAGTGTGCTTCTGCTTGCGATGTTTGTTGTGGCCTATTCCTCTTTTTTTGTTCTTGCTGT

TCCAGCCGCACGCAGGCGTTCTAGCAGCCAGAAATGTGGCTGAAGATC

>CK177457_bb No definition line found

ACAATTTTTGCTTTTCATTTCATAGAATGTGTTTTCCTTTGTCTTCGTCAACACCTGTGAGTAACGAAATCGAGACTGTT

GCAACACTACGACGTAGCAAACTCGTTAACTGTCTGCACACATGCAATTAATCTAGAGAAGATGACSCCGGACCTTTAAG

GCACTGTGAGTGCGGYGAGCACGCCGCCACCGATCGGTTGTGATGTCATCACTTCATGTAGCAGCAGTTTTTTTATTTAC

GAGCGTGCCAAGTTTTTGGCTGAACGGCCCCTTCGTATTGTACAATAGTGTCACAAAAATCCTTGTTGCCAATGTAGTGC

ATTTATGGCGAATGTCTTTGTCGAAAAATTCCACACACACACACACAGTGCATGCAGTTTTTCTGAAGATGGTGTTATCC

AGCTTGGGCAGCGAAGGCCAAGCTTTTCATTTTCTGCGGTCGAGATGCAGATTGCTGTTAAAGAATAAGCATTGGGCTGC

AATGCTTGAAAATAAAAGTTGCTGTAGTAAAATTTGAGGTTTGTGAACAGCCTTTGAGCCTCATATTGATTCTCAGGGTG

ACTGTGTGTTTGTTTTGAATGCATATAAACATGAAAAATCGTGTTTGTTTTTGTTACTTTTTTGAAAATCTCTGGACCAT

TTCCAGTTATTGCAGCTCACGATGTTAGCCATTTCCATTCAGCCTCCAAATTACGAACATGTGCAAAAGATACAACTATC

AAAACAAATGTTAAGATAAAATCAAATTGTGTGGAAACTAGATGTTGACTTTTCTTTCATAGTACTATACATGCATACCC

TTTATATATATATATAGTATATATATACATATATATTAAAAAGGGCTACATTAAGCCCTGTAATTAGTCAGCCATAATAA

TGATTACAATGAACGTTGGAGAAGCTGCATTTCAGTGCTGCGTTTATCTGAATCGACGTCTACTTCACAAATAACTGTAT

TGTTTGTGATGTGCTTCTACGATAGCATAAGGTTTGTATACAATGGGTACCTTACAACATGTAAATAC

>CK177466_bb No definition line found

TGAATTCTACATTGACTTTTTTTAATTACAAAAATACCAATCACAACATCTGATGTAACATGCACRCATGATTTACGAGA

TGTTTCTGGGAGCTGTACTGGTACAGATGAAGTATACCGTCAAGTTAAATGTATGTATGAGGTATCGTTGCCGATGGCAC

AGTCAAACACTTGGCTGAGGTACATGGTATGTCCAGTTTCTTGGCCACAGCTGGTTTTTCATGTCTACCACCAGCCCCCG

CCACCACCGCCTGCAGCCGTTCCACCTCCAGATTGCCAGCCTGCTCCAAGGTCACCACCAGAAGCCTGCCATCCACGAGC

GTTGCCGCCATGCCCGCTGCCACCGCCCGCCTGCCAGCCACCAGACGAACCACCCGTCTGCCAGCCGGCACCACTGTTAT

CGCTGACAGCAGCGGGCCAGCCAGCGCCTCCTCCGCTGCCTCCGCCTAGCGTTGCTCGCCACACTGCACCAGCAGCGGCA

CTGGCAACTGCACCATGGCTGACCGCAGCTGCACCTCCGAAGCTGCTCACTGCTCCATGGCCGCTACCGTGGCTGCCATG

GCCCCCGCCGGCGGAGGTGCCATCGGTCCGAATGACGATGATGCTTTCAGGAGCGCCGCCTCCAGAAGCACCGTGATGAC

CATTGCCGCCATGTCCATTTCCGTGAGCGCCTGTGCTTCCTCCGTGGGTACCATGTGCTCCTCCGTGGCCACTAGA

>CK177478_bb No definition line found

ATAATTGAAAATGTGCAAAATCGAGCGGTCCGTTTCATCCTTCACAACTACCATCGAACTGCCAGTGTCACTGACATGAA

ACGAACACTTGGCATTCCCCTTCTTTCTTCTCGTAGGAGGTTATCCCGTCTTTGCCTTTTTCATAAAATATATTATCATA

ACAATGAGTTGAAGTCCCGATACATCACATCCGCATCCTATATCTCCCCACGTGTCGACCACCCTTTCAAAGTTCACGTA

CCATCACAGCGCACAGTCACCTACTCATATTCATTCTTGCCAAAAACATCTTATGAATGGAATCACCTGCCTGCGTCACT

AGTCGCAATCAGAGACCCTGACCGTTTCCGGAAAGCATTAACGAACACGCTATAAAGCTGCATCACGTTCACAAATGCAT

TTACAACACACTGAAACTGCAATTTACTTCTTTACCACCTATCTCTGTACAGGTCTTGATCTCAACAATTTTTTTTTTTT

TTTTTTTTTTTTTTTGCCTTGCACTGATGTTTTCTTTTGTGTGTGTAACTCGATAAAATTTGCTTTTTCATGTATCATTT

TTTTTTTTTTTTTTTTTTTTGAAAATGGGTATTGAAAAGGGATCAGTCGATTATTTTT

>CK177523_bb No definition line found

ACTTAGATCCTTAAAGGAACAGTCATCTCTGAAAAATGTCCAACAAAATGTACTGTTGAAGTACTGTCGAGTGAGGTGGT

TTACCTGTTCCTCTGTGTTGCTTCAATGTAGGTGCACTCTGTTTCCATCTGCAATATCGGATTATTTGTTCATTGTGGTG

ACGAGTGTTGTGAATTCACACAGCACAGGCCACTGTAGCATTTTCTGTAAGCTCAGAATAACCCCATTGTTGGCATGCAG

AAAGGAAAGCAATATTTATTCCTTTGTTAAGCACTTTGCACCAGGCTTCAAAGCATTTTTTTGACTGCATTGAAAACTGG

GACAGCATTTGTGAATGTTGCCATTACAAAATTTAGAGTTAAAACAGAATGGCTACATACGACTGTGCTCAATGTACTTA

ACAAAGCAATGTTGCACATTGTTGGCAAAAAAGGTGTCCTCATTTGAAACCTACCCAAGTTACTGCTGTAATAGGAGATA

GAAACTGTGTTAAAGAAAAGCAGGCTTTTTTTTTTTTAATTTCAATATTATTAATATTGTTAGTCTTTCATGAAAACACA

GAGATGAAGCTTTCCAGAGTTTTGTGTAAGCATATCAAGAAGAAAGCCTTATTGAAGAGAAGACACAAAAAGAGACTGGA

CCTGCTATTACAAGCATATCAAACACTTCTTAGTAAGCTTAGCTAGAACATCTTGGACTGCATTGGTGTCACCCTCGTCT

TTGCACCCCCTTTTTTCTTTCTTTTTTTTTTTGTGTGTGTTGTTGGTTTGTTATTAACCTTTTTCAGCATTAATGTGTTG

GAAATTGATATTATGGCAACACTGTTGCCTCAAATATTAATTTTCTTATATTGTAAGTGTAGTATCTTGTTTTTTATGAC

ATTCATTAGTGATAGTTAGTGATAGTATTGCCTTCCCTCTTCCCCCATCTCTTCCCCCCCCCCTTTTTTTGACAAACCTT

GTATGCTTTTG

>CK177571_bb No definition line found

CATAAACACCAAAACATGACATCACATGAARMTGCAAGTACACTCGAAGCCGCAAGAACGAACRTTARGCAAATCCGTGT

TMTACCTAACTATTCGCTTTGCCCGTGTCGAGGGACYATGGACAMGGATCCAGCGGCTTCCTTAGCTATTCTTACCGGAA

AATCAASGCCACCATATTGTTCCCAGGTTAAGGAGCATCATCGAAWGGGWGAKCGTAAGTGTATGSYKASMMTTCAGTTT

CGTCAAGCCTGGTCTGCAATGATCAAAACCATGGAGATTGTGCATGCATCACCTGTGGTCCCTGTATTCCTGGTGATTAT

ATAGGTACGCTCAAAACAAGGTGGCTGCCACGCTGTTCTCTAGTGGGCCCAACCAAGTCCCGGATCTAACGTGAAAGTGC

TTTCGAGGAAGTTCATGTGCCATGAAAAGAAAGTTCGTATGCTGTTTAAGCAAAGCATCTGGTCTTCATTGGCTGCTTCG

TAACCTTCTTGAGAGTGTATGCTGTAAACCTGTACTGATGGAACTGCTATCGTGAGTATTGCTAATAAAAAAATGAGAGC

GCATGGC

>CK177575_bb No definition line found

TAGAACTTCCTTTCTTTTTTAAGTAAGCAAAATAGTGATTTCCTGTCCCGAACAGATCCAGATGTTTGACAGCGACCTGG

ATGACTTTGTGGACCTGGCACCTGAAGACGACATTCCAAACAAAGCAAAGCTGCGTGTCGTCTCAAGGAGCACTTCAATT

CTGTGCAACGCAGCAGCGGTTGCTGCCGGTCAGAATGTTGCTCCCAGTTGTTCTCAAGACTCGTGWGTGYWRTYCAGCCG

GCTCCGATCCGCGAACAAGGGGAAGTTGTGTTGCAAGCATGTGAAGCAGCAATTCAAGAAGATGGCAGCGATGCAATGGA

GACATCTCAAGCTTTGCTGATCGTGACTCGAATCGAAGATGCACCATGTGCAGTTCTGTCTTATAGACAAATGAGCGGCA

GTGACGAGGTGCAAATTGAGGACGCTGCCATCAGCCACGCGTCAATGCACGACAATGAGGATCATCTCAAGTTCCAGCTA

CCTCCATCCTTCGGCATCTGGTGTGACACTTTTTTGCGGAGCAAGCAGCCAGTCACTGCGAAGGTCAAAACGCACATTAT

TTCTCATCTCTTCAAGGCTTGCTACAGGATGACTTCATGTTCAACACCGAGGCTGTACAACAAAGTACTTGACTCGCTCG

TGGCAAAGTACCCACATGTGATGGAAGGAAAGTACAACAGGCGCCGCTGGTTGATCGCCTTGAGGAGCAGGTTCAAGAGC

GAACGCGAGAACCTCACGGGGCCTTCAGCTGCTACAGTCGAAGCACAGGAAACGTCTGGACTCGACCTTTCACAACCTGA

TGCTGACGATGCATGCACCTACGCTCACACACAGCATGCGAAGACACAATTGAGTTCATCGGCCTATTCACCTGGTTGCG

AGGACAAGTCAGTTCACGAGGAACATCTCGGCGAGTTCACAAATGCATCTGCTCATGGTGCTGAGACCTATATAGCCGAC

ACCTTGCCCTTGGACTCCATGTGCCCTGTGTCCGACTCAGTGGAATGTCTGGAATCTGAGGACGATGAGGAATC

>CK177579_bb No definition line found

TTTTTGGKTGARAAGGTGTATGTAGGGAATGTAGCACAGCAGTTTTCATGATTTCCTGTGCAATTCCTCTCTCTATTTTC

ACTCCTTCCCAAATGCTGATTGTGTTGCRTCRCAGTGAATGCTTATAGCATGAAGCTCATGCTGTATGAGGCTTTGGTTT

TTCAAACTGTRTTCAGGCACTTGTGGTTGTACCTTTTTTTTTCCCCACAAATTATTAGTCTGGCTGACATTTCTTTTTYA

AGAGGGAGAAGGAGGCTCCTAAAAATGGTCTAGCATGTGCTCAGCTTGGTAACTGTGGTTCTCAATTTCTTGCAGGTTTA

ATTTATCATTAAATGGTACTTTGCTTTCTGTAATTTTTTTGTGTAATGCTGTCTGGCTCAACTTGATTTTTTAGGTAAAT

TGTCAAGGTCTTCTGTGAACACGCACAACCATTCTTACGTCATTTTGCAGTGTAAAAGAAAATAAATTTGACAGTGACTA

GAATTTAGGATAGTGCTTGGTGCGGAAGGTAGTTAAAGAAAGATGCGACTTGACATGTGCTTAATTATGTAATGTTCCGA

ACGGCTTGCCCAAGGCAATAACTGTCMATTTGGTGGTTAGTGTAATTGTGTTGTATTCATGTTGCAATCATTGTAATTGC

AGTAACCAATTTCAGTGTTATCTCTTGTWGCGGTTCAGGTTTGTAAAAGCAACTATATTATTGCACTAGCTTTGGCCAGC

TTTTCATTATAGCTTTTCTAAATTACTGGCAATTGARCTATCTATATTTTTGAACAAGTTAAATGGAACAGAGGTTGGGC

ATGGTCTGCGGAGAAAGTCGCTAGATTCAATCTCTGTGGGAGAACATATGAGAAYATTTGTAGGCCTGCATCAAATACAC

GCCCCGTAATACTGTTTAGACGCTGAGCTRGTAAAGCATGTWAACTTCATCCTAAACATTYMACTTAGCWGTGCCTGTTY

YTTATCTGCGGTCAAATCTTTGCATCTGCACTGGCAGCTGCAA

>CK177583_bb No definition line found

TTTGACGCAAAGGACAATGAATGCTCTTTATTTCTTGCACAGTCATTTCTATATAAAACATCATAAAGGCTTGAGCCTTT

CACAGGGAGTTTACATTACAATCCCACACACCAAATCTCGATGCACAAAAATAACACGTAAGGCACAAGGTGAAAAAAGG

TGAAAAGGTAGTTTAACATCAGTTTGAAGGTTGCAAGATTCTTCTTTACAGGGCAAAAGGCTTCACTTAAACATTGCATC

AACAATGCCAAAGTGAGCTAATACTGGAAGATACGAAAATAGATGCACCTGTAATATGATGTATACATATATGTGTGTCT

ATACATGTAATACACAGGATGAATTCTAGCTGYACCAAATATTTTTATGATGACATGCTGTTTTTAGTGGYRGAAGGTGC

AMCAAATTTTTTTAGATAACAGCAASGKRAACTACGGTTGCACTCCCAATTGAATTGATAGGCTGGTGACCTCTAGATTG

TTAYGCAGTCATAAGCAGTATCAYGAAACACTTTTGATCACAACCATGCTTGAATGGGAAAATATTGCAAAATACAATAA

ATGGATTATTTTACCCTAGCGTACTCGGTGAAATTGACAGAGCAAAAAAGTGCCTACAATGGCATTCTCATCAAAAATGC

CGTTCATCACAAGATGACTGCAATTACTCCAGAATTGAGTACTTTGAAGTCTGCTTTAGCTCTTCCTTTATTTCTTTTCC

GCTTAAAGC

>CK177585_bb No definition line found

CAGCGATGACGAAGAGGAGGCCTGGCTCGAGGCCCTGGAGTCCGGCCGCCTCGAAGAGGTCGACGACGAGCTCCGCAAGA

TGAAGGACCCGACGCTGATGACGGCGCGGCAGCGCGCACTCCTCGAGAGCAAGTCTAAGGACAAAGATGAGGACGGCCCT

CCCGCCGTTCAGGTGGAGCCTATCCAGGAGATGACTGAAGAGATGATTCAGCGCAGAATGCAGCGTGCCAGAAAACGAAA

GCAGCAGGCTGAAGAGAAGAAAGAGAAAGACAAGAAGCAGACTATTGAGCGGCTGCTCAAAAAATCAGAGTCTCGGCTTC

GTGCTTGCAAAAAGCCAGCCAAGAAGGCAGACATGCCTAAAGTGTCGCTGCTACGAAGCAGTGAAGCTGGAACGCTGTTA

GTGTTTCCTCCTGGTATTCCGTTCCCACTTGTTCCTGCTGTGGCRCCCTSCGAGTACCCACGGAGGGTGCTATGCAGCAT

CAAAGGCTGYTCGAACCCCAAAAAGTACTCCTGCTCCAAAACGGGTGTGTCCCTTTGCAGTTTGGAATGCTATAAAGCAA

ACATGCTGCAAATGTGTGTATGAGGTGTCGTTACAAAATAAGCTATTTTCATTTTGTTTACCCAAATCA

>CK177586_bb No definition line found

ATGTAACAAAACAACTTTATTGTTCAATACACATGGAAAAAATAATACATCACTCTTTGGTCTTGGACCCAATGCACAAA

TGCAAGCAATAATCATTGTACATTTGGGCATAGTGGATGCTGCTATATGCAAACAAGATATACTTTCAATCAATTTAGTC

AGGAATATACAATAATCACCATGTGGTTATGCAACAGCTRGCACACAAATATCTAGTCTCTTTTGCATAACATATTTTAA

ATGACCTGCAATACCTCAGCAGTGGTTAGYTTAAACTACAAGAACAGCTTTGCAGAGCTTTTTTAATTKGAGCCCTGTAA

TTGGCATGAAAGTATAACAATAGCGATACATATCAGTGCCTTTATGTTMATTAGTAGTATTCGTCCATGTCTCCACAAWT

GTGCTGCCGTTCAAGTGATTATGATCAACCAACTAATCCACCAATAACTTCTTAGCAGGAACCAGTGACGTCACCTCAAG

CATAGAGGGCATTTTGTCAGTTTTGTGGCTTTCACGAGACTCCCTAAGCACAAGTTTTGTCAAACATGCTGGGAGGATAA

AATAGGTGTAACTAAGTTAGTTCCACATGACATGTGAGCATAAGGCAGCAACTTGAAGGGATGTGTTTGTATATGCTGAA

ATTCACACCCCACGATGCAAGCCACATAATACAAACAAATATGAATAAGTAAGCCATTTTGCCAAACTGTGTTCAACATG

GGAATCGTTTGGGGGGCCTTCTTTGTTAGACAATAGAATAGATGTTGGGAAATGCATT

>CK177587_bb No definition line found

GTTTTGCCACCGACCAAACAACAAGTATGGCGAGAACATTTACATGGCCTGGTCATCAGACCCCACAAAGGAAGTGACAG

GGCAAGAAGCTGTTGATTCGTGGTACAGTGAGATCAAGAAACACCAGTTTGGCTGCGAGCCACGCAGCCTTGGTTCTGGT

CATTTCACGCAAGTCATCTGGAAGGGCAGCAATGAACTGGGCTCGGCACGGGCCCGTACGGCTACTGGAAAGCTGATTGT

TGTGGCTAACTACAACCCTGCAGGGAATCTCATAGGCTCCTTTGCCCAGAATGTGCCACCACCCAAGAAGTGAGCGCGGC

ACCATTTTGAATGAAACTTGGCATGTGGTGGCTTTGTGAAAATTGTGTGTTTGTATGTATATATATACACACACACAAGC

TTTTTAATGTCGAAGTTGGCTATTGGAAAGCCTTTGCATTTTCCAGTTTAGAAGATTATTTTACCAAAAAATGCCTCTTG

CACAGTATTACCTTTGTAATTAGGAAGAACCTTGGCATCAAGGCATTGTTAACATTGAGCACAATTGCTCATGACTATTG

TGACTATATTCAACGTTTTGGTCCAAGTATGAAAAGAAAGTGTGTAAGTGCTAAAGATGATTATGCAAACATCCATTTAT

AACACTGTGTGACGATGTAGCTAGGGCTGCTGAAGCAGCAAAAGATATGTCTTTAGGGCACCAAGATTTGACTGTAGTTG

GTCCAAGCTATTTTGAAAGTCATTTAAATGAAGGGAGCCATCTGTGCCCCGTCTGTGGCAGCAAACGTGAGTAGGCACCG

ATATGTTGTAAAGTTACAGTAACTCAGGCATTTATCTTTAGCCACAGGTTTCTAAACTAGGTACTATTTCTGGAGCACAA

ATGTGTATTATTTTGTGAAATGTATCTCTCAGCTGTTCTATCTAATGAGAGCTTTTAAGAGTGTAAGAAACAGCAAACA

>CK177607_bb No definition line found

ACCGGTACATTTATATAGGTCATAATGCGCCACATCAAACACCCTTTTTTTTTTTCGGTAACATTGATGATGCATCAGAG

GTAATTCCAGCTCGGGAATGCCAATTGCACATTTTCTTTAAGAGTGGACATTGCTGTATGTGTGTTTGTTTTGTGTTTTA

TGTGCTTCTGAATATCCGGTGACACGAGTATTAATGTAGCAAGCTTGTATGTGATGGCGGTAACATCTATTTACACCACT

TGATCAGGCCATAAGAAATTAATGAGGCCATTGTACGCATACATTGTCTTGAGTTATTTTTAATTGTAATTTTTCATTGG

TCTAGTAGTGCCTTGTTGAAATACTATCGCTGCAACACGTGCTAACATGTGTTATTATAGCCCAACGTGTGCATCTTTTA

ATAATATTGTTAGTATATGTGACCTGAAAGGTATTGGTGCATTATACTAACTGCACATTTTAACCAGGAGCTCAATCAAG

CCATGGTTTGCCTTTATGCCTTACACTTTTTGATAGTGTTTACTGTGGCAATGGCTTCCAGCATCAGTGCGAGTGATATT

TAATTGTCAGAGATTATGGCTGTGTTGTATAGTCGAGTCGTGCCAAGAGAGATGCGTTTTTTTCTTGTTCATTAATTTCA

CTCTGTTGCTTTTGGAGAACTACTAGATTTACAAAAAATAACCAGGGTTTTTTTTTTTCATGCTATATAATATAGSAAAT

CATGAAATAGAGCTCCCATTCTAGTATCTTTGCAGTTAAAACACAGCCATGAATGGCCGAAAGTTAGGTTTGGCGGCGAC

CTTGGGAAATTCGAATGTTGAAAAAGGCATTGGCTTGCACGAAAAATATTTAGTTCAGAGGCTAGGTATTAGGAGAGGCC

TCCAGTCCTGCTGTGGCCTTAGAATAGGCCGACGGTCTTAAATAGCCGCTACACAAATTTTTATACTGCTGTTTCTTATA

AATAATCTCCGACTATTTTTGGGTCCA

>CK177618_bb No definition line found

GTGAAACAATTGGTTTTACTTTGCCTTCAGTCAACAACTATAAACAAACCTGCAGACAAGCAGAGAATATACAGCAATGT

ACAACGCAAAGAGGAAGAATAATTAGTTTGGAGAACAAACAAAATGTACAGCAAGAAGCATGGCAGTCAAAAGTAACGGC

GCCCTGCGCCGGTTCTTCTTGCGTAAGTTAGTGCAYCCGGTACGACGAACTAAACCCCCTAAAACTAAAGTCCACGGGCA

AAAYACCACAAACCTGACCTCTCCTACAAGTCCGTACACCAGTATTAAGAATACAGTGGCACACTGGGGCTACCGCACAA

CTTGCCAAAAAAAAGGTAAACAAGAGGCATGAACTTTCAAGAAKCAAGTTTTCCGTGAGCACTTAAGGGGGAAAAAATTG

GAGGGAGRRCTGAGAGAAATAGAAAAAAAAACATTCACAYTTATTATTTCTAAGCACTCAAAAGGGAAATTACATTTACC

AAAGGCCCCTCCAATTGTTCTCAGTCACTGAGGCACTTGCGCCACAGTTGTCGAAAGGGATAGCACCTTGCCAAAATTAA

ATAAATATAGGAGGTGTTCCCCCGTGTCCGAGGGGGGACAAAATAAAATGCGAATTTGGGGCGTGGTGCTTTACTCCATA

AGGCAAGGCCACTTCCCCTATCACCAGGCTATACGACGACGCTTCCCACCAACTAATTGCAGTGGCCTGGA

>CK177634_bb No definition line found

ATGAGGGTGGTGTCATGAGCGAGCACTTGATATTTCAATAAAAACTTCAAGTTATTCTCCATTATTTAGAACACACAAAT

GTGCTAGCAAGCATATTTTTATGGTTTTTGCATTATTCTCGGGCGTCTGATGCAGGCAATCTGGAAATATTTAGTGTTCA

TTCAAGAGACRAYGGCATATATAGCTGCCAGGCTTCCAATCGACTTGGAATCATCTTCGCAACGTAAAACAAAGTTGGTA

GTTCAAGAGCCTCCTCACATCGTCACACCACTGCAAAACCACATTGTACAGCACGGAGGAGAGCTGCGGCTACAGTGCAC

AGTACAAGGTAACCCAGTGCCTACAGTCCATTGGCTTCATAATGGCAAAAGGGTGCATCATACATCCCACATCACAATAT

CAGAAATGGGGATCCATATTAGAAAGATGACAAAGCACCAAGGAGGCATGTACCAGTGCTTCGCAAACAACTTTTTGGGC

ACTGTCTACTCAACAGCGCATGTCACCGTTCTCGCCGTGAATGAGTCTCACATTCCTGACAAAAACGGCGAGGACGAGAA

TGAGCCAGACAGCATCAATGGTGCCACAGAAAATGCTGGAAAGAGAAAGGACAGTCACAAAAGAAAAAAGAATAAAGGAG

TGAAGCTGGTGCTGCCTTCCCGGCCCGAAATATCTCGCCTCTCCGATGACTCAGTTATGGTTCGTTGGAATGTGCCGCAC

AATGATGGTCTGCCCATCTCGTTCTTCAAGGTCCAATATCGAGATGTGTCAACTCCACACTCGCACTGGAAGACAGTTGA

AGAGGACGTGCCCCCACACATCCACTCCCACGCTATTGTGGGACTCAAAGCAGGAGGAAGGTACAGGTTTCGCATTGTGG

CCGTTTACTCCAACAATGATAACAAGAATGGGCCAAACTCTATCAAGTTTCTGCTGCACAAAGATCCTCCTAAGAGGAAA

CCCATGCAGGGACCCATTATTCGCCGTGTCAAAGCTGAAAGCTCAACGGCAATCACTCTGTTTTGGGAGTACTCAGACTT

GGATGCCGTGGACGTGGAAGGTTTTTACATCTACTACAGGCTGACACAGAGTGCTGGTGACTACCTAAGATCACCGTGGC

T

>CK177690_bb No definition line found

GTGTGTGTGGTGTGTGGTCTGTGCGCGCACACGAATTATCTAGGATGTGCAAAATGTTTTCCGTGACATGGTAGCGGTGT

TCGTCGCGTTTGAAAGAAGCATGATAAATGTTATCGCGTGAGCGGCTGGTAATTTACCAGCGTTCTTGTTCAAACTGTGA

TAATCGCCGTCGCAGTGGTCGTAAACATGACTCTGACGTGTTGCGTGCCGTTCTGCTCTTCGCACGTGGCAAGAAGCAAA

TCCGACGGCGAGCCAAGTGTGTCGTTCCACGAGTTTCCCGTGACCGACATAAGAGAAGCGTGGATCAAAGCCATTTCTCG

TGAAGGTCCTGACAAGATGCTGTGGCAGCCGCACGAGACAGCAAAAGTATGCAGCATTCACTTCAAGCTGGAGGACTACA

AGGAAGGCATGAGAGGGCGTCGTCGCTTGAAACCCAATGCAGTGCCTTCTATATTTCCAGGATACCCTGCTTACATGCAG

CGCTCTGCCGAAAGCATTAGAAGAATCGCTCGTACTTTACCGCAGCAACGTCTTTCAGAAGCATATCCTACAGTATCGAC

TCATCCGCCACGATCAAAGAAACGGCTCCTGTACAATGGCCAAAGATAACAAGAAAGCAACCATGGAAACTACTTCGGCA

ATGAGCGAAGCTCTAGACAGTGCCCACATTATGGCAAAGAAGCCACACAAAAATGTGGATACAAACGAATCGGATACCTA

CCCCACAGAGCTAACCATAGAGTGCATTCCATCCTCAAAAGACACTCACGCTCCAGCTAGCTCTTCACATCCGCTAGTTA

TCAAAGTGATATCATGTGAACAGTGCCCTTCGGTGACAAACGAGAGAAGTACGTCGAGCGTGCCAGATGTAGAGAGCCTC

GATGGCCACAAGACATTTGGGACGCAAAC

>CK177738_bb No definition line found

CGACGAATTAAGGCATGACCAACCGCCATAAAAACCTGCACTTAATATTTCCTCTTTTAAAGGTTACTGGTAAGTGCATC

TGTACATGAAAACAATTGTTAAGCCTTTTACAACATTCTAAGCAGTAAAAAAAGAAAGTATCTCTTCAAATTAAAATCTC

TTCAAATGAAAAGTGCCAAAGTATTATCAAAGGACACTAAAACTGTCAAAAAAAAAAAAAARAAACAAGCRTGAAAAAAA

CATTTCCARGAGGCAASTGGCTTTGACACTGCAAGCARACARRATTCTCTCTYTTTCYGTCTTTWCTGCCCTCGTATTGT

CTCTCGCACTGATGTTCAARATTGTATAATATGCARRGTTTTACACTACCTCCGTATAAATGACTRCCATGTCTACAGCA

CACAAGWCTTTTTTYTTTYCTGGCTTCCTTTGARAAAACCACGAAGTGCCAAGAGCTGAAGTGCTTAATGTGTATGGAGA

GAAAAACAGTGTGCTAAAAAGGACATTRATACACKATGTAAAACCACATAAAAWTAAAAAAAAAAACAMATTTAGCAATC

CCAAATTGMYGAGCTACAACWGARACAGACTAAGTATTGAGGCACRAACACCGGGAAAATGTAARAAGCACGGCTCCTGC

ACAGCTCCCAGGCACAGTGAAGCAGTGAAGGAGATGCCAAGCACAATAATCGCGTGCTCTTCGATTGCACAACGTTATGA

CCAGCTAGAGTGACTTGTGTGCAGCACCCAGTGCTAGCACGCTTAAATGCAGTTCAAGCAGAATGCAGCGAATTGAGA

>CK177775_bb No definition line found

CGGATGCTATCGCTTGTTACGATAATGCTGTCATATTCTTTTTTGAAGGTCAATATAAGCAGTTAAATGAAATTTCCACA

GTGGCTGCACTCTTTGCTTTTCACAAATGTATCTTTTACTCACTCTCTTTTCTTTTTGATCACCCAATTGATTCCTTGTG

AAGTTCTTCTTTGATGCATTCAAGACCCGAAGTCAGCCGTTCACCCTGTTGGCTGTTTGCTAGACCAGTAATGTACCTGC

AGCTTGCGATACATTACTTACCTGGCAGTAGAGTCGAGTAACTCTCCATACATCTAACCATATTCACAATTATGTGCCTA

ATTTAAAAAAAGAAATTTCAGTACAGTTCAGAAACCATAGCACACAGGCCATAATGACGTTCCAAAMMAAAAAAGTGACC

AGTCTTTTGACTAAAGCACAACGAACAAACTTCAAACACTAAAGTTTTGTTTTCTTTCCACGACTCAATAACCACCCAAT

CATCCATTAGCACACCACATTGTTACAGACATCCCTTATGTTGACCTCTGGTGAAAATTTTTTTCACATTCTGCGTGAAA

TTTKTGAACCATCAGCTTCAATGTACCCAGCATGCACTCAACCTTCAGTGCTGCATCTTTTTTTTTTGTTTTCTTTGTGT

GTGTGTGTGTCAGAGAAACAGCTAAGGCCTTGGATTGTTGGACTCGATTCAATACCTCCCAAGCAAGAGGTGCAAACACC

TCTTGAGCACATGTACAAACATTTTCCTCCTCTGTTAATGACTTGAAGGCACTTCCGCCAATAAATGCCGAAAAT

>CK177789_bb No definition line found

ACGATTGCAAAAACTGGGCGTACCGTACGTGTGTGCGGGCCGCGCTGTTCKKGCYGCGYCRYTMKTGTGTTCCGCGCGCT

MTTACGCGCTTGGGCATATCGGGCACWTTTCTTGACATGTAAGCACGTACAGTCGGCGTCTAGRACTTGTAGACAATTTT

CGCGTGTGCTGAACTTTCTCATAGCCAGCACCATGCCGCCTATTCATAACGGCAGCGACCCGAAGGACACCATAGAGAAG

ATTATCAAAGGAAACAAAGTAGTGATATTTAGTGCCTCTGATGACCCAACGTGTGCGCGGACGAAGGAAATATTTACCAG

CCTAAATGAACCTTACGTCTCCATCGAGGTCGATGATGAAGGCTATGGGCCACCAATCCAAGAAGCACTGAGCCAGAGGA

CTGGGCTTTCTGGCGTGCCTCAGGTGTTCGTCGGTGGAGAGCTGTTGGGTGGCAGCGAAGACACGGCTTCCGCACTGAAG

AAAGGCACCCTGGGCCAGTTACTTATAACCGGCATCTCCTACGATTATGACTTAGTTGTCATAGGTGGTGGATCCGGCGG

CTTAGCTGCTTCTAAGGAAGCTGCTAAGCTGGGAAAGAAAGTAGCTGTATGTGATTTCGTCAAGCCGACCCCAAAAGGCA

CTACATGGGGTCTGGGAGGCACGTGTGTCAATGTTGGCTGCATACCAAAGAAGCTTATGCACAATGCTGCGTTATTAGGT

CAAGGTATTAAGGACTCGATGAGCTTTGGATGGGAAATGCAAGATGTCAAATTCAAGTGGGAGACGATGCGGGGCAACAT

TCGTGACTACATTGCATCACTCAACTGGAAGTACCGTGTCAGCCTGCGGGAAGCTGGCGTTGACTACATGAACGCGTATG

CACACTTTGTGGATCCACACAAGTTGAAGTTGACTGACCAGAAAGGTAAAGAGAGTTCATCACGTCGAGGGATTTCCTCT

TAGCCATGGGGTGAA

>CK177899_bb No definition line found

GAAATCTCACTGCTGTGGGCGTGAATCACATCGTTTCTGAACTGGTGGATACCACAGCAAGAAATAACCTGGACCATTTT

TGTGCCTGCTGATTACCAGCACACTAGGGGTGACTTGAATCGCTATGGGAAAAAACGAAAGCACTCAGCAAGTGCAGGGG

CGGAAAGTATCAGACGAAACCCAACAAGCAATGCCTTTGTTCTTTTTTTATGCGCGTGCGCCACGTTTACAAATCTCGCC

ACTAGTTCGCGTCTCGCTGACCACGCGTTCGCGTGTTCCCTCTAACAGTGGACTGTACTGCACGTGCCCAAATGTTCATT

GTGTCACTTGGGTCCACCGAAAAAGAAAGGATAGCTCATGGAAGCGTTGCTGTCACACTCGAGACTCTGAGGAGGTTAGG

GTAGCCCACTTTGTAACTGAAAGCTCGTAAACATCTGCAGCGAGGATGAAAATTAGTCCAGAATCAACTTAATCTTCCAT

GGCTTTTTTGTTTGTGGCATTTTATTTTGTTCCTCAATATCTAGCTGAAAGGTGCTGCTAGACTTATTAAAACACCTAAG

CTGCAATGCACCTTCCCAAGCACGCCAATAAGACGGAGCTTAAAGAAGAGCAAGAAATCCCACTTTAGTACCGTTCAGGC

AGATATGTGAAGCGCTTGACAGTATCACAAATCAAAGAAGCGCATCGAATCACTCAGGGACTGAAGAGGTGCATAGCTGT

GCTTGATGACTGATAAGTCAAGGTACAGAACCCTCCCCAGTGGCAAAGTCGACGGAATGCGGCCAACTAATCTACATTTG

GCGAATGCAATGACTCGTTTAAACCCTTTGAGTGCAAATTGACAGCTAGTGAATAGTGGCTAGTAAATTATGGACAAGTA

TTGCCAAAACTCTGCAACTACTTCAAT

>CK177967_bb No definition line found

CGGGCATATCATTTCATATATATTCTTATGTATTTCATCATGGACACACATGGAAAAAAATTGTGCACATCCCGCTTGAG

ATAGAGGAAAAAAAATAACGTGCACAGTTTGCGCATAACAAAGAATAATAATAAAAATGGAGAAACAAACGCGTCTATAG

GAAACATAAACGCTCTGTCGATGCAATGGCAACAAGATATATGAGAAGAAAAGAAAACAAAAAAGCTGGTCCCCGGAAGA

GGTAAGAGAGAATTGGTCAACACATTTTCATCGGCAATCAAAATGCACTCTGCCACACGTATCAATACACTCATACATAC

ATTCGTAGATACATACGCATACACTAAAATGAAATGTAAAACCAATGACTTGCAATGGCATGACGGGGCATGTGCGTAGG

CTCGTGTTTAATGATTGAGAAAGGCCCAAGATGGCACACATCCTGCTATCCTAGACAAGATCAGTCAATACCATAACAAC

ACACAAGGATTACAGAATTCAGTGGCAAAAGACACGATGGGAGTAGGAAACGGAAAAAAAATGCCAGCAGTAATCTGGCA

TTATATTCGGCTGCACCTGAATGGACACCTGAATGTGCACCTGAATGGACAAAATAGGTTATGTCCTAAAATTTTTATAG

GCAACTAGGCACCACGGAGTAGCAGTTCACTGGAGTTTTCCCAGTTCAGTTGTTAACTTTTGCAGCAGAGTTTCTTTGAA

CACAACCATCAATTGACATGAATTTAGGATTCCGATTTTTTATAAAGTTTCTTTTTCCAACCAAGGAGGTTGAGAATTTC

AAAGAGCTTTAGGTTTTCTAGAAACTTTAGGGCAACAGTGTAAGATGAACACCATGCTGTGAATCATTATTATAATAATA

ATCATAACCA

>CK177968_bb No definition line found

AATTTTAGAGCGTATTTGATTGCGGATGATTCGCRAAAATGCTTATGCGAGACAACAGCTGGATCCATTTGCCCAGGAGA

CCGAATTTACATTCAGGCCTCTGCCTCAAATATTTCTTCTAATCTATAGAAACCMATTAGTCCCAAAACTTTAAAGTGCA

AATACGACGATTTAGAAGTCTCATGAAGAGATTAGTAGTGTATATGAAGCGCTGTCCGAAACACCTAGGCATAGTTTTGT

ATTCTTTAGATGYATCTTGAGGTGAAGTTTACAGAAATGTATGGTTTCTCATCGCCGAGTTAAAGGTGCTATGTCTAAGT

TTAAACTTGTTTACTATAGCAACTTCCAATACTTCTGTGTTTAAAAAATSATGTGTTAAACTAACRTGCTTGCTATACAT

TAACTGTTTCTTTGAWATGCAACTACCATTTTTTCAAYAAGGGGAAATGTGATTGCTGAAAAAAAATTTTGCGCTGGGTT

CGTAGCTYCGGAGTAAAGGCTACCTCTTCAGAAGGAAAAAGAAGAGTATATTTTATGAAAGAATGAAAACTTCCACATCG

AATACACGCGAATCGACGATGCCTTTAATACGGGGCCACTATTTCTATTGTGCTCTTTCCTGCGCCTTTTTGAATGCATC

CCTATCTTCGCTTTCTTTTTTTTTATAGTGTTCTATTTTTGTACGTTTTCATCATGCATCACGATGACACGTAACAACTG

TGAACACATGCTTTTAGCTTGCGACAACAACCCGCGTAAAAACCTATTTAGAGATGCGCTTACAACCCTTTTATTTTTAT

ATATT

>CK178073_bb No definition line found

TATGCTACAGCCTGCGGTGTCCCGAAGGCCGCTTTTACCGCTGTAAAGTTTGTTTTAGCGAAACCCATGCAAGATTCAGC

CAAACAGACGCATCCATGGATTCTCGCCGTCATCACAAATCGTGCTGCGCTGAGTGGTGTCGGAATTCTTCACGACACAC

CGGCATAAAATTCTTTCGGATCCCGGCAGGCGAAAGTATGCGAGACGGCAGGACCTGGCTCATTTACCACCTCGTCAGAT

CTACAGCAGCTACCGCCTGTGCTCTGCCCATTTCACCAGCAGAGACTACGCAGACCCTGGACAGACAGGGCTGCTAAGAT

GTGCTGTGCCAACAGTTTGCATCTTTGGCGATGGGCAGACACACGTCCAAAGTCCAGGCTCTCGTCGGCCCGCGAATACC

ACTGCAATGCCCGCTTACAGCGCCGATGAAGAAGTTCAACAGACTGAGCCCTCATGTTCCAATCCAGCGGGTCGACAACA

CAAGCCTTCTAGGKGCTCRCCCCGGACAGCCAGAAAARTGAAGAACCTGGAGAAGATAAATTCAAGGCTTCGGAAGGCAC

TGTCAAGAATTGGCAAGAGAAAGGTTAAAAAGCTTTCCCAGTCCGAGGYRTTGAATGCTATCAGGCCCTACGTCCGCAAG

ACATTCTTTAGATTGCTTGAGACTCAAGTTAAARTTAATGGCGTGAAACGTAGAGGACGGRGGTGGTCGCCCGAGCACYG

CCAGTTCRCTTTAAACTTATACTTTCATGGTCCGAAAGCGTACAGACATCTGTCA

>CK178103_bb No definition line found

GTTGTATCGTGAGGCTGACGACGGAAAAAAAAAAAAAAGACGACGTGCGGAGGCTTTTTCCCTCGCGGAAGTCCTGCCGT

CACCCCCACTTGTAATTAATGCCGACAAGAGGAACAGAAAAAAAAGTATATATAAATTTTGTCGCCCTTTTTTACCCGGT

TTATTTTAATTTCTTTTGTATTTTTCACTCCTTTATTTTTTTCAACCTCTCCTCTACCCCTTCTTTGTTTGACGTCTTCG

AAAACGACCTATTTGTGAAGAGGAAAAAATGCTGCTTAGCTGCAACTTTATTGCCAATCATTGACCTCCCCCACCTCTTA

CTGCCTACACCTCCCACCTGTTTATCGTAGGTCTGCCTTACATGAATTGACGCGTCTTATGGCAAAGTCTACCACGGGAA

GGTGTTTGCGAGGGTGCTGACTTTCCACCGAAACAGCCGGTTTGATACTGAAATACTGAGGGGAGGGGTGGCTGCAGCGG

CTGTGAACAGCTGACCTCTCTTCCAAATGCCTCACTTTCCTACACACCTCCTCATCCTCCGGCACGCCCGCTATCCCCAC

TCTGTCGGTCTTTCTCTTTCTCGCCGTCTGTGGTGGGTTGTACATCTATGGCTCTTGAGGGGGAAAAAAAAAATTATGTA

CTATAGAGAATTGTGATTGCTGGTGTGAGGTGGTGAAGTTTAAAGRCCCTCCCCTCTGAAAACCCACCCCKTGCCTRTCC

CTCGGCCCAACCTTTTTTTGAGATCTTGCTTRGGGATTTCACGRGGATGCTGGGTTTGGGATGAGTTTGCTRAAGGCTCG

AATTGGTATTCAGAGCCTAGACTTCTGATCCTCCRCAGTTCGTTCATTGTTGCTGAAACTCCTTTATTCCTTGACTTTTT

CCTTTGAGCAGGTCTTTATCGGTCCCACTTAGCTTGTATAGAATTTTCTTAGTTA

>CK178109_bb No definition line found

CCTGCGTGTAATCGACTAGTCGAGCGAAGTCCTGGTTGTCCCTGTAGCTGACGATGAGGTCGATGCTGAACACGCTTTGC

GCACTCTGCAAAAATATTATATGCGAGTAAAAAAAAGAACCAAATTACCATCAGGTTTTCGCGTCAACGATATCACAAAT

AGCATTCTAAATAGTACCTGAMCAAGYGTTCTMAYTTTGAACGATGCACAGCGAGCTCATGCTGGTGTACSTATTCTTTA

AACACATKGGACTACACAAAAAGGCAACTGCATCCTACTATAATTAAGGGAGGACGCTCGAGATTCTCGTAATAAGTTGA

GCAAGAGGGCTTGATAGCGCTCTGCTCGCATCGCCTCCTCAATCGGTAGACTCGCTTCGCTACTTGCGGGACGCTTCAAC

CCTTCCGCAAGGGCAAAAACAGCTTGGCCAGTCCCAATGGCTGTTGACAACATTCCTATCTCTTTCGGCGCTGACGGTGT

CAACTGGCACCACCTTGGTGTTACATTTAGCTTGAATCTTTCAGGCGACATTCACATCGACACATGCAATTTGTGCAAAA

AGCTTTCAATGACTACTAGAGTTATGTCGCGTCGCCACACTAACTCTTCTATTGCAAGGGAAACTACAAACTTATTATGC

ACTCTTGGCATCACATATCAATTATTTTAGTTTAATATGGTTGACCCCTACACAACGTACATT

>CK178230_bb No definition line found

GTTCGTCAGAACTAGCGCCTGAAGGGGTTATGTACATAGTGTTTCTTTCATACTTGCGTCAACTCTTGTACAGTGTATTG

TGATGACTCCTCTTTCATACGTTACTATAGTCACATATGTTCAAGGCTTCTGGCGAGATTCCTTCGGACATGTACTCCGT

CTCATAATAATCTTGCATTAGAGTGGAACCTACTATATATGCTTAGAGACGGAATCTTGGGTTCGTTCAGTCTTTGTCAA

GTTTCCGGACATTCAGTTTTGATTCGCAGCTGGAAGGTGGCTGTTGTTTTCAAATCGCTGTCTTCATCTGTCGACGGCAA

GCGTTTGAACATCAGAACAAAGACAGAATCTCTACTGTATATCATCCCCAGTATGCAGGACTTAACAGACTATGCTGCTT

TACGGTCCACTTCAGAGGTACAGGGGTTACGGTGCTTCGTTGCTGACCGGAAAATCACTGGTTCGATTCCAATCATGGGG

ACTGCATTTTGATGGAGACAAAATGCTTGAGGCTCGTGCACTGTGCGGTGTCAGTGCACRCTAAAAGACACGAGGCGGTC

GAAAGTTTCCACAGCCTTTGACTGCAGCATGCCTCGCAATCGTATTGTTATATTGTCATCAGAAATTATGTCTTTTTTTK

TGTGGTTTGAGGCTGTTTTCCTGCATTTGCTACATTTTTCTGGGTCATGTCAGCTTTAGAAGCTCCCACTTAAATGCCAG

ATTTTTTTTTAATGGGGCACGGTAGAGGAATTTTTCTTACACAGGCCACATGCTACATAGTTTGRAARCTTTATTGGTGA

GATCATTTGCACATATCTGTTGTCGKACAAATCAAAAGGACTTTTAAAGATAGGTGTCCAGAGGCCTTGGTTGTTCTCAG

TGCACATGCATTGAATGCAAAGCACGCTTCCGGTTAGCGCAACAGCCACTTTTCTTTTTCAAGTAGTATGCGTACCAAAT

AAACCCAAGGCCTGAACACCCAGTTTTAATACTCTAA

>CK178239_bb No definition line found

GATCTCAAGGTTGACTTCGCGCCCATGATTTGGCAACCCAACCAGTTGGTGGGCGCGCCGCTGGGCGCAGACGTGACCCT

CGAGTGCAACCTGGAGACGCACCCTCGCGGCATGACCTTCTGGGAGCGAGACGACGGAACCATGCTCATCTCGAACGCCA

AGTACGACTCGCTGGTGGTTGAGACCGGGCCCTACAGGCTCCTGCTGCGTCTCACCATACGCGATTTGAAGCCTGACGAC

TTCGGCACCTACAAGTGCGTCTCCAAGAACCCACTCGGCGAGACCGAGGCCAACATCAAGCTCCAAGAAATACCTCGACC

GACGTCTTCGACTGCGGCGTCCTCCAAAGAGTTTCAAACCAAGAAACACTGGGAGACAACCTCAACGCAGCAATCCAATG

GACCTCGACATCATTTGGGAACTCACGCTTCAAACGCTCTGCTGAAAGAAGGGCGCCGAGGAGAGTATTCTACTGAAGAA

GAAGGCACTCAAGGGTACCCTCCTCCAAACTTTAATCAGGACTACGAGAGATCCGCGGACAAAGACAGCGCGCGACCATC

GTCAGGTCACAACTCGGCCCCGCAAAGTCGTGCCACGTCTTCCGCAGTTGTCCTCCTGGTCGCAGTTTGCGCCTTGCTGC

TTCGCGCCCACGGCGTCTGATCAGCGCTTTCAACGACAACCGTCTGCAATCAAGAACCACGCGTGTATTGCTTCCAGGCG

CTGCGCGTAGCCAATGTGAACAACAACAAAAAAAAAACTGAACGATGGAACGCGTCGCAAGGAAGAGTCGCTTCTTTCTG

TTCATTCGCACCCGCCATCAGCTTGGCTCGAGCTCAAAATGAGGAAGACGAAAAAAAAAAACCAAAATTCAGAAAATGAC

CGGAACTATCACCGTTAAACGTGCGACCTCTTTGCGAAAGAACTGATGGACGCTCAGTGCATGTTGACAACGGTACCTTC

GGTGCTCCTCGCATCTCAACAAGTGTCGCCACGGGAAGACTCTGCAACCAACCGAGTTGTGAAAAGCAACAAGCTCCTGC

GCCGAACAAAAAATGAAGAGGACGACAAGTACGGTTGGTCTGCTCGAGTAACATGCAGGCACACCTGT

>CK178253_bb No definition line found

TGTTGTGCTAGCAGAATGCATATCTTACTCCTATATCTACACATTGGCTTACATTTGGGCAAACATTGTTCCTCAGTTGC

CTGATGTGCGATACTGTACTTTCTTTTCTCATTCTAGTGCGTGCATAGGTGTACGAGACAACTACTACCAGAAAATAATG

GTGTATGTGTGCGGTGGAGCTGCTGTTGGGGGAATACTCTTTGCTCTTTCACTCTTTGCCATCAACCATGACAGCTCACT

AAATACCAGGAATGGTAGCAACGGAGTGTCAACGAATACCAGGCCTGGGGATATTGTTGACGTTCGGTCCTCTAGTCCAG

CCGAATATGAAGAGAGCATTGGAAATTACTTGCCAACGTCACCACTCCTAGGATCAGGGTCAAGTGCATAGATCCGTCGA

GTGTGTGGCTCGCATGGTGCGCGTGGGAGATTCGTTCGTTCAAGGACGCACKTGTGTGTGAGACATTCCTAGGGACATTT

TACTCGTCGTCTTCCATYACTGCGTTTCGGTGAYGTCTGTTGTGCCCARAGGGTTGCGAACCCCAGGTGGGATGGGGAAG

AGCCAGAAAACAAGATTTGACAGRGTTAYTCGCACATTCAGCTTGTTGGTCCGGACATTATGCAAAGCAKGAAACGGYCT

KCATTGCTTTCTCTCCAGCTTTTCTTTTTCTAAGCTAGTTTTGATTGATGCCAACACATTTTCAAGCATTTGCATTCACG

TAGTAATGTACACATTACGTGCATCACTTTGCTGTGCACGTATACTTTTGTTTCTTAGCATTCGGAAAAAGAAAGGTGCA

TTTGGAAGTGTTAATGTCTGCCACATGTTACTTCTTTAGAACATTTGGTATGTCCTTCTACTTCTACAACACATATTTTC

ATGGAGAACTGCAGAACGTTTAATGTCTCTTTTTCACAGCCTACCTTCCAAGTAACAGGCATATCTCAGTGCTTGTGCAC

TGTCATTAAGTGGGTATGCATAGTACTATAAGTATATTTATTGAA

>CK178269_bb No definition line found

GGGGCAAAGCAGCGGATGAAGCAAAGGACGAAGAGAGTGCGTGCCGTGAGGCGGTCCGCGATTTATTTCCACGCACACCA

AATTTTTTACACTGGCTAAACAGCTCCACTGCAGATAAGGCAGTTCCGCTTGGCGGACACTGACGAAACAGCAAAGCTGC

ATGATGGCGTGCAGTGAGGTTTAATGAAGTGGTTTGGAAGAAGGGGGATTATTATTCTGTGTTATTATTATTTATGGTTT

ATTACCTATGCTATAGCCGTATTTTATTGTATGTAGCACTTTTTTATTTTTTTCTAGCACGTTTTATGCACATGTATTTA

TTTTACACTTTTTCTTCCTGCAATCTTCGTGCTGTAAATCCATGTAAATCGCTGTATATGTACATACGATATGGTATTCT

GTTTTACTTGTTGGTTTTCTCGTCACTGCACCGGACTGCTTGTKGTTACTSCCCTGTATAGTCTGGTCGYTGGGTCTCGT

CAAGCTACTTAYGTAGCTTTTAACCCAACGCAACCATCCAGCATGTATAAACTGRAAAATAAATTGAATTGAATTGRATC

TCACGCAGCACTTTTTGAGAGAAGGCTATCGTCTTTTGACGACATTTGCAGCGAAGGGCGCA

>CK178350_bb No definition line found

ATTTCGGGTATTCTCACTTCCATGACAATATTTTGTGAATTCAGAGTAATCCTCGAATTTATGAAGAAAATTTCAAACCG

CTTCTTTTAGGCCTTGTTAAACAATGGTAGGCAAGTACCATTGTGTCACTTTAGTGTCCCTTTAAGGAAAAATAAATGTG

AGGAGTTTGATAGCAAGCACTATGTACATGGCGCATTGTCAACATTCTAATTAAATGGTTTTCAAAGGAGCGAGTTGAAT

ACAATTTGGCAGTCATGGTCTCTGATTATTCACTTTTATCCCACTTTATTTCACATCACAGGATGCCATCAGCACATCAA

GAAATAACATGAAGTGGTATGATGAGTCCATTTTACTACGTCCAGGTGAGCAGTTCAGTAATAACATAAAAACTGGACAT

CAAACTTTCTGTAAGCTGAAGTATCATGCAATAAAATAAAAATGTGCAATTCCCCGTTACGACACACAAGACATTGCATG

GGCTGTCCCATGTGCGGAGCACAAAGGAAGTTCATAAAAGTGCTGTGTATGCGCATTGCGCCAGTGTTCTCACCCTACTG

CAGCATACATTTCCAATTCTTTCATTTTCAATTTTTCATTTCAGCATACATTTCAATTTGCAATCGGAACTCATTCAGAT

GCAGATGCTATGGACATGTAAGCCTCGTTCTGCATTTGGGGGCAACAGAAAAACTAGAATGTTTCGATAAGTCTCGTCGC

ACCAAGTAGATCATAAGCAAAGTTTGGTTCTCGGGCATTCCAAGGGCCTGAA

>CK178423_bb No definition line found

TAAGTTGCTGTGGTCCACTTTATTGCAAGATTGTGGGAACCACAAGCATTTTTGCAACTTTTTGAAGATTGAGGTACTCA

TACTGGCTGGATGGCCAGCTAGGGCTTATCAGTACAAAAATCATTGTTCATAAAAGTCAAATTTGTTCCTGAAAAGGTAC

AATGACTTCTTCTCGCATTGACTTAGCCATACWGAATGTTGAGTGTCGCTATATTTTGCCAAAAGGAAGCCAGTGAAAGA

TCAAATGAAAGTTCTGACAGCATAGTTTATACAAAACAAGGCAAACCTAAAACATAATCACATGAAAACAGATAAAATGG

TGCACAACAAAAGTTCAAAGCAATTTGTACAGCTTTCTTCTGTGTAAGTGATTGCATAATGTCTGAAAATTGGTAAAGTA

ATAACATAGAACCTCATAACTAACCACTATGTTAGTATTTTAGAGAGGATAACCCCACACATTTACATATTGTGAAAATA

ACATAGTGTGACCTACAAAATACAGTAGTGCTCCAATCGGCTAAGGAGCAGAAAAAGTGCGAATTAAGATTAGTTGTTGC

AATGYAATACWRAGCCTGCAACCATCTATTACTGTWAAACAAARTACTGGTTTCATGTATACAAAATYGTAAGGTCAACA

CTTGGCTAGCCATTGATCATGCCGTTACTGAAGCAAAAATCTGAACAAAAAATGCTTCAAATGWATTTTCCGAGTGTGTC

AGAAGAGAAAGTTGCGTTTATGCTGGGTATGTAAATTACAGCGAGACAATGACTGAAGAAGAGTCATCTCTTCTTCAGTC

CTTGTTTCTATATTTGCGTCCCTCCATGTGCGATTACATAGAAGCCATCACTTGGCAAACTTTTYAGCGGGTGGTTTTAT

TGACAATCTTGTGAGCTTGTGCCAAGCCAGCAGGCWAATACAAGTTGGCAGGCATTACGTCAAAATACMCTCGTGACTGT

CGCATAGCTGCTCATCATGGACTGCAGTTCTGGTCCCT

>CK178424_bb No definition line found

GAATCTTCTTAGAGGAATGGGTGGGTGGGAGTTTTTTTTTTGGAATGGACACCTCTCTTACAGGTGCCATGTTTGTATGT

TACGGTGAAGCCAACACATGGAGTTTGCCCACTTAAAGTTGATATGGATCGGAAATTTTGCGAGTGGCATTTTGCACCAG

AACAAATGTTTAGCAGCTGAAAATGACAATAAGCACAAACTGTCTTGAAAAAAAAAGAAAGGAGAAATGCCGTTTTTTTT

GTGGTATTTCGTAGTCTCCCAAGAAGCTGCAATTAGCTCATGACACACACACCCTCATGTATTCGCTCATGGTCATACAG

TGTTTGTTCATCTCTGTCGGTTCTCCTCACTGGTAGCGTGCCCGAGATGTCGAAGAAGCTTATGTCTTTCATGTGCTTTC

CCCCAGTTAGTCACCAGTTTTTAAGCAATCGTCCCAGCATGAGAAATATCGTAGGTACTGTTGCACTCAAAACAGACGTT

TCCTAGATATAGGAAACTGTGTAGGCTTGGGAAATGCTCTGCTCAACCACGGTGACCAAACTGGACATCTCTAAAAAATG

ACAGTGCAGTGAACCAAGCTCCATTTGAAAAACAGGACAGTGGTGCCATCTTGCTTTACTACACGTAAATGTGGCTCTCT

TAAGTTTGCTGCGCTGTGGTAATGAATTCAACCGGTCTTTGCAAATTTCACTAGTACTTAAATGGCTTCTAGAAGTGTGA

ATGTGACAGTAACTGCTGCAAGCAAGACTTGAAAGGGCTGAACATGCTGTGTCTCATCGTGCAAAGACTAACTGGGCARC

TCACCAACCGCTTAAGTAAACACAAAACGACAAACTTCGATTCAGTTGTGATAACCTTCATGAAAGCTGGCATCCTCGCC

AACAATCCCCGACGGGCAAATCCATGGCCTCCTGCTTTTCTTCATTGCCCGACTCTCGTGACTGTTTGCAGTAATATTTT

TTTTCTTGGCAAAGTGAATGGTTTTCACCGTGAGAG

>CK178527_bb No definition line found

GCAAAATTATAGTCCCCTCCACCCACCCTAAATTGTGCATTTCCGTTCCTCGAGTTTCTTTTGGTTCCCGTCTTTTTTAT

TAAGTGTAGTTGATAGTTTGCGCTTGCGGTGTCCACTTTGGGACGTGTGTCCTTCCTAACAACATTTCTCCCAGCGACTG

TAGCGCAGTATTTTAATCATGCACAACCAGCTAGCCCAACACCTTACTTTGCTTCCGATGCCTCAGTGTTAATCTGTTTC

TGYTCATACAGATACTCATGTCTCGAGTTTTCATTTAGCGCATCTGTGCCACTGCTTACTGTACATGAAGCAGTTGAAAG

TAGCACTCGTCCCATGTCGTATCTTTTTCTTGTGCTCATAATAGTGTAAYTCTTTAACTAATTTTTCTTTGTGAGCTACA

GTCGTATATTTTGTACATTCTTGTTTTTACAATTTAAACCCTATCATCTTGTTCAAAACCAGGAGCTATTTAAAATGGAA

TTTTTTATGCCTTTGTTTTGCACCATAGTTCTGTGATAATAATACAGATGGTGGTTACAGAGCTGTGGAGAGTTTCTTTT

TTTTTTTCTTGCTGATGAATTTGTTACTTGTGTGTACATGCTATTTAGTTTTTTGCACCCTATTCATAATAGCATCTTAA

CTTGCACGAGCGTTAGCGACGTATTTTTTTCGTTTGTGTTACCACATAGCTGCGATACATTACAGTGTCATCAGAATTGC

AACTGAGAAAAGAGTGATGCTCTAATATAGCCAAATGTGTGTGCTTGCAATAAAACATCTGAAA

>CK178529_bb No definition line found

GCGATTTATTGTGTTTTGTTCTTTAATTAGGTTTTTTATATATTAATATACACCTGTTTTCTTCATAGCAAATGCATTCT

GATTTTTCGCCTTCCCTTGATGGCAAACTGCCAACAGGTTCTTCCCAAGCATGTGAAGGAGGCATATCGTCTGCTCAACA

AGTCCATCATTCGTGTGGAACAGCCAGACATTCACCTCTATGAGGAAGAAGACGAAGAGAATGAGGCAGAAGGTGGAGAA

AATGCAGAGGATGCCATGGAGGTTGACGAGCCACCTGTCAATGGCATTGCTGGGGATGCACATGGTGATGGAGACGATGT

GAATGACATTGGCATCATCCAGCCTAAGCAGAAGAAGGAAGCAAGTGCCAAGAAGCCACATCTCACAGCTGCGCAGCAGA

AGGCTCTTACACTTTCATACGAGGACTACAAGAAGACCGCGAACCTGCTTGTTTTACACCTGCTGCGTGAGGAGGCTCGA

ATGGAAGCCGCGGAGAAGGAAGGGGCTGCATCTGACGATGAAAGTGGTGTACGGCGCATGGATCTGATCAACTGGTACCT

GAAGGAAATAGAGAGCGAGATAGAGACGGAGGAAGAGCTGATTCAGCGCAAGACCATTGTGGAAAAAGTAATCTAACGAC

TTGTGAACCGGGATCACATTCTCATCGCACTCTCCAAGAGTGGCCTGAAAGGCGCTGAAGCAGACAGTGAAGAAGACGAA

AACCCAATCTTGGTCGTTCACCCCAACTTTGTCCCAGAACTCTCGTGAAGGGCACTCCTCAGGCTTGCCTATATTGTTTT

TAGCCTGTGGCACTGTCATACCGTTCACATGCTGCCACTTGCTGTAATGTAAAAAATGTAAATACATATGTACTTTACTA

AGTATTGCCCATTGACTTCCAAGAAAACTGGGGCTCAGCATCGCTCTATTTTTATATGCATGTGCACTTTTTAGGGCATT

AACTGTCATCCGAATTTAGCAGTCCAGTGTTCATTAGCACTGGCATTTTTGTCGGTGAGCTTTTATATTACTTTGAATTT

ATATACCATACTCTGATTTTGC

>CK178575_bb No definition line found

TGTTCTAGAGTTTTATAAAACTTGGGAGAGTGATATTTGCCCTTTGCACTGTCTCAATAAAAAGTTAACCTGAGTGTGCG

ATCCCAAAAGAGGGTGAATATTGTTACKTAAAAATGACACGAGGTGTGTCTATTTAAAATCTATATTCAAAATGATRCGT

ATGGCGTCGACTAAGATGGAAGCAGCACGCACAACCGACAGACCACTTCCTCGTTGTCGTCTTCTCACCGCTGATACGTC

AGCTACGTAGCAATATTACAGCAAACTAACAGACAGTGATGCCAGGAGAAGTATACTGTGTGTTATAGACACAATATCAA

TGACAACTAACA

>CK178686_bb No definition line found

ACGCTCCAGGCATTTATTTGTAAAACTTCAGTGAAGTTTTGATATACATACAACACCCAGTCATCTAATTTAGCAACCAG

GCTAGCAGCCACACAATGACTATAGAAAACAAAACTCTGCTCAGTAGTGCATCTTGAGTACTTGAGCAAAAAAATAAAAA

AAAATACTCTCAGCTTGSACGCAAACGGGCCGGCCAGCTGTGCGATGCATTCAGACTAWAGTGCAACTACCGTGAACTTG

AATGCCCAGAAATTGGCCACAGGTAAAAATCCCTACACAAGTCTTGGKGCACACTACTCTTCTCTCTATAAAACCTCCYT

GCATCAAGGKGTGCTCAGGTCAGTTCATCCTAAACTGCTAAATATTTTTGTGCCATTCCTGGAGTGCAAGATCTCAGGCA

TGCAATGAGTTGGATGCTTGTGCTTTGTTCTATCATGCATGCTTCTTTGGACAGGACACAGTCCTTTTTTTTTGTTCTTG

CTTAAACTCAAGCCGTGGGTAGGCTGGCCAGCCAGCCCAGCATCTTTGGCGCCTGCTTATAGTGCTGTGAATGCCAATGT

AGCATGGCTTGTTGCACTTACTTGCAATCCTA

>CK178704_bb No definition line found

AGTGCATGTAACACATAGTGTAAAAATGYTTACTGTGTACTTGAACGATTSTTTATTCAGMATAGTTGTGGMAATGTTCA

RGYAATAACATTCTGAAMAGCTYTCATGCTAGTTTGCGGTCTCTGGCCTTCATGTCCATGACGCCTTGCTCAGTTCTTGC

YCTCCTCCCTTCTTCCAGGTTTGACTTTATCCTGGATTGCGTTGGTACCCATCAGGGCCTGTCTCCAACAAAGCTTCTTC

ACAAAGGGCGCCTTTCTACCTATGTTACTGTGGTGTCGCCTCTGGTTTTCCAATATCTGCCATTTTAGCCTGGATCCGAC

TGATTAGCCGCCGGCACTGGCACTTCAGGGAGTGTCGGGTGCCGGGCATTTGGGCCTGATTCAATTGTTTCAACAGGTGC

TCCCCTTGGTGCATTAGGTGCACAAGGTCTCTACTAGCAGCATCAGGTTGAGATGCTGCACGCAGGTCAATCTCGCTGTT

GCTCCTGGGCAAGCCCCCGTTCCCGACGATGCAAACATTTCGATGTATGCTGTCTGTAAAGTCTTTTGCTACGTTGCTTT

GTGGCTTGGCTTTCTTCAGGAGGGACACAAGGTTCTGAAGATTTGCGGGGAGCGAGTGACTTGCAGCAGTACTCTTGCTG

AGAATGAAAGGCACATCTGCAACAGTGAGCCTACAATGGTCAGGAAAACGAACAGTGTGCGGAACTGCAGATGTTGACCT

TGCTCGGCGATCATGGCTTGGGTTTTTCATAGGCACTGTGGCTTCAGGCATCTTCGATGCCGTTTTTCTCGACACCCTTT

TCTTGACATCTGTGTCGGTTCCCTGGTGGGACTTTTGGGGGAAAAGTTGAGGCTCGGAG

>CK178745_bb No definition line found

TTTTACATTCTGTGAAATTTTATACACACTCACAAACACATATGCCTATACGCAGGAATATGTGCAAGAAAGACAATCAT

TCATTATAACAGCACTTGGTCCATGCGTTCAAAAACTTTCATTCAACACATTTTTCACATCTCTATATATAATATAATAT

ATCCTGTGTACCATGCAAATTTATGCACAATACTCGTGCAAATTCTTATTTTCATAAAGTACAATAATCCAAGGCAATAG

AAGGGAGAAACAAACAGTAAACAATGGGGAAATAATGACTGCCTAGAAAAGCTCACAAAATTTTGAAGAATTCACAGGCA

TGTGAGCAAACTGTAAAGGTAACCGTAATGGGAAATCTACTCGAAAGTCCCAATGGCCCAATTAAAAAAAATAAAAAGAC

GAAAAAAAACCGGGTAACATTTTGGCAGGTTGAGAAAACACTAAAGATACATTTAGCAGGTAGAATAGGGTACCAGATGA

ACAAACAGTTCGAGCTGGAGAACCATAACCGAAGGTGACTGCAATTGAGTACAGACACTTCTCATCAGCATACCTGCTTT

TAAGCAACTTGTATATGTTGGGAACAAGCAATAGTGACAAGACATTACATTCTCACTTGAGTTAAAAGCCCAACAAAAAA

GAGGCAAACAGGAAAAGAAGGAGCATGCTGAAAGGTGTCATAAAAGACGCCCTCCAAAACTTGCTAAATACTACTTGGAA

GCCATTGAGAACAGCATGTTGCTCTCAGAAAATTAATTGACCAACAGT

>CK178746_bb No definition line found

CTTCGAGGTAACTGAAAATTTCCTGGGGTGCTTTGTAAGTGCTATGCAACCACACYTCAAAAAATTTGGCATTATTTTCA

TTGGTAGGTAATCACATGTAACTAAATAGGAGTAGACTCTTTCCAGTTCTGACATGTGTGTCAAAAGTGCCTCTGCCAGT

GGACAGTGCCATCATCTGATTTTTAGTACGCAAAACTATAGCGTTATAGCAACTGATAAAAGTGAGGTGAAATTGTAGCC

GGTCAAGTAATAAAATATGAGTTGTATAGTCAAATTCTAATATTGTTATCCATTGCACATTCATAGAGTCACTTTACCAC

CAAACACAAAGTTTAGGAAAAAGACTACATCCCTAATGTTTTAGCCGTATGATCTTTAGTGACATATGTTGTCAAATTGA

TTGCTATTTTTATACCCTATATCATGTATTTTTATTGGCAGTTGTATGTATAAGTTTGAAGGTGCTGAAGTCTTGTACAC

ATTTAAACTCKGTTTAATATTTAACATAAGCAAACAAGCTACAACGTGGCTGAGTACTTCAGGGGTCCTTGAGACTGCAT

GCAGACTAAGGGACTCCAAGATTTGCTTGTGGTACATSAGGTAGCAAGTGGAACCTTCTTTATCAGAAGATGTWGGTTGC

TTAATTTGCATGTCCTACATGCTGTCCTGATCACGTTGCTATGTGCTGTTGTGTAAGCTATCTTTTTCCTTTTTGCTAGA

GTCCTCTTTCTTTATTTTCAAGTTTTCTATTATAATTTCTACACTGTTGGTCAATTAATT

>CK178761_bb No definition line found

TTTGATAATTCCGGAGTGTTCAGAAGGTACTTCCTTACACTTGCGCACTCGTGCACTGGCATTAATAGTGTCATCATGTT

CAGATTTTACTTGCAACTTGTATGCTGCATGTTTGTTTGTTGTGTAACGTGCATGTAAATATGTATAAGTTGTGAAAATG

TGGGCTTGTTTGAACAAGCCAAAGATTGACAACCGTTGCGCGAATGATAGTCTCTATCGATGCTCATAAACGGTCATGTT

GTGTTGCAAGAGGGATGCATATGTCTGTGGCAAAGGACACTTGGCATAGCTACAATGCATGATAAAATCTTTACTGTTGC

AAAGAATAATGACCGAAGTAAGTACGCCTGAAATGGTTTGAGCATAAAGCAAGAGAGTCTATACCTCACAGGGAGGAAAC

GTTTTTTAACTTAAAATGCTGTGGCACTTGACTGCTTGAATAACCAAGTTTAGGTGGTGCAGACTATAGCTTGTGTGGAT

TTAAAGATAAAGTCTGAATTGTGGAAGGCTACAAGTGTGTGAGTGAACAAATGCAAAGTCAGGTGAATCAGTTGCAAATT

TTGTTTAGATGCCACCTTGCCAAGACGGGGCACTATCACTTTAATAATGCCTCTTTAGAAATGCATGGGAATATTATTTT

AGCAAGTGTATAACTTTCTTTCAGAATGCTACCATAATTTTCATTTAACAAAGCTTTATTATCACCCTTGTTGCTATGCC

TGCTAATGGCTTTCCCTGTAAAACTTGTTCTGCTTTAATGAGCATCATGATGAATGCTGTAAATGTCGAAAGATTGATGA

AAGCTCTGTTTGCGTTGCATGTACGGACAGTGGATGTATAACATAGATTACGATGTTATCTTTACACCTATCTCTTGTTA

TCACTTCTGCCTGCTAGTCCCAAAAAATGCCATAGCCATCTTTTCAGGCCATTATTGCTTCACTGTGCAGATTTCGTGAT

AAAAGGGGTCATATTTGGAATTTGTTGTGGTTG

>CK178769_bb No definition line found

AAAGGGGAGGAAAAATTAATGTTAGCAAATGCACTGTGTGCGTGAGTGAATATGCTGTATTGGCAAAAACGACTCTGCTA

ATATCAGAAGGCAAAGTGTTGTGACATATCCTGAAGCATCCCTGTTCTTGTTTCCAGTATGGCTCTGCAGTGTGGTTGAG

CAAGTGGTCTCAAGATGGGGATGTGTCACAAAGGCATTTTTACATCACTGGCTATGCACTGTTTTTGGCATCCTACGTGG

TCTTCAACTTTGTGTTTTGGTCTATATTTGTTGTTGGAACGCTGCGGGCAGCCATCTGGTTTCACCAGCAGCTGTTGAAC

GGCATCCTGCGCTCCCCGCTCTCATTTTTTGACACTACACCCATGGGCAGAATCATCAACAGGTTCAGTCGTGATGTTGA

GTCAGTTGACAAAGAGATACCGATCAATGCAAACATGACCATGTGCAATATTGTCTGGGGAATGCAGCTGCTGATACTGA

TATGTATCATGTCACCTTACTTCACAATTGTGGTCGTCATGGCTGTGCTCTT

>CK178868_bb No definition line found

TTGGGGCATATTGGAAAGTATTTATTGATTCGGAGACTTGTTCAAGAGAACTATGTACACAAGAGCCTCATACAAGCAAG

GAATAATAGAGAGATGCATTGTAAAGACAACCGGTATTTTACATTTTTAAAAGATGCAGTGCACCTTACCACCTTGGGAA

AATATGATGCGTCTTGTGTCAATTAGTCATCATATGTGGGAGAGAGAGAGAACAAACAACTTTATTTCGTTATTCTTTAA

GTGTCGTCTTTAGATGTGCAAGTAAATGTGCCCAGCTTTGATAACAATGATCATTACTTCAACTGAGTTTGAATAGCCAC

TAGGCTCATAATTGGCTGTATCTGTGATAATGATTTCGAGCAATGGGTTTGTAGTTTCAAGTAAATGCAAGGTAAAAAGT

CACACCAGTACAAATATTATGTAGTAGATAGTACAAAAATTTTTATGAGAAAGCAGTCAAAGCTCTTAAAAGTGAGAGCT

AAATAAAGTAAACTACAATGAAAATATTAGCTAACAAGCATTTTCTGACTTATTTTTTATGAGCAAATTCCTTTGATTGC

ATTAAATAACTTGGAATAAAACAGTATTTTGGCACATGCAAAAGTGCCAAGGGTATTTTTGATTCACTAGAAAAGGCCTA

GTTGCCTACTGAAAAACCAAATCTTATTCAGTACCCACCAAAATCAGCCGATATTCACAAGGAGCTCTGATGCCTCAATT

TATTTTTAGCGGTCAGTTTGCAGCCACTTCAAACACTGTACTAACTATTACAACAGGAATAAAAAAAGAACYTAGGTATG

AAWAAAACATTTGTGAATTAAGCTCAGCACACCATTTGACTGCTAATTA

>CK178879_bb No definition line found

ACACATTTTTATTTGCAGAGTTCATACAAAAGAAGAGGGTTCTAACACAATTTCACAGTGGAAAAAGGTGACACCAGCTT

TTGATGACTGAGCAAGTGAGGACTGTTCCTAATACATACGAGAGGTTGATTTTGTGCATCTCCTGACAGTCCTGAAGATA

TTTGGTGGCTTCAACGGTCGGTTATAAGTAAGGTCCGTAAATTTATGTCTTTTGCATGCAGATATGATCACTTTCCAGCC

AGTTGAGTTCATTCATTTCACAATTTYATAAAGACCTTTGCTGAGTGCAAGACCAACTTTTTCCTGAGCCACAGCAATAG

GTGAGAATGCTCTTGTTGTGGCACATCTTTACTTTCTCGCTCATTTGTGTTTGTTAGGCCAGAAGAAGTCAAAATGACAA

CACTCAATTCAGCACCACACTTATACCTGCTGCAACTGTCACTCGACTGATAGAGTTTTTATATTGAGTGTATTCATTTC

ATCATATAAACACAGGCTGTCTTTTTGCACCTGCTGCAGGACTATTTTGCTTCACCTTTTTATAAGTTCTGAATAAAGTG

CTAGTTATTTAGCTATATTTTATGACTGTTCACATAAAGCAAACATCTTTTTGAAAACAAATGTTTAGAAGTTCAGACTA

GTGTTAATACAAGGCAGTATGCAGTGAGGGTGCTATATGGGTCATCTTAAAATATGCTTTGACTGCGAGGGTGAAAGAGC

CGAGTTACTGAAAACACAGCTAACTGAAGCTCTCATAATAACGCTGCATTGTCAAACATTTCACGGCCGTTTATACAGAT

AGGTCTGTCTAAGCAACTGTTCACTCTTTCTACTGTATAAAAGAAATGTGTAGCATAATATGTTTTCTTTAC

>CK178880_bb No definition line found

GTGTCTCAGGCCATTGAGAGCGGTTGAAACTGCATGTTAGTGCCATAATGTATACCACAATCGTAAACTAAATACTAGCA

CTGTTGTCATGAAAAGCATGACTATTTCTCATTGCATTGTTTTTTTTTAATATGGGACATATGAAGAAAAAAGTAGAACT

GAAATTGAAGAATGGCCACAAATAAACAATAAGCCATTTTTCGTTTTTGCCCTGCTGGCTTCGGTTGCTCTAACTCTACA

ATCCTGCTCAGGGGCATAGCCAGATGTGGCACACCGGGCCCAAGCTCCACCCCCGAAATTTTTTTTCCGCCATRGCATAG

CGAGCGAAAAATGACCGTGGCTACGCGCCTCGTCCTGCTGAATCATATTTGGCCTTTTTGTGTTAGTGCGATTGGAGCAT

GATGGCTGTTGGATGGGACAGAGTCTAGAATATTTCAATCTCTTCCTTCCAATTTCCAGTGTTGCTTGACCATGATCTCC

CTCAAAATGTTTTCGAGTGCTTTGGTGTGTTCAGAAATAGAGCTTCAAGCTTTCTTTGAAACCACTTTTGAGCAAACAAA

ACCTTACATCTCGTGTTTATCGTCACTTCATACTTTACAAGCACCAACCTGCGTAATAGCTGTAACCCTACACCACAACT

TGTGATGAGTGACATGTGCCACACTTTTAAGCAATTACTATGTACTTGGGCTTCTTTTTCTAAGTCATTTGAGGATTTAA

TAATGAAGAGTATTAGTTCACAATATAATATTAATAGCTTTTCTGCACTGACAGATGCATTACGCACCTAATAATAGCAG

AATAATGTAGCCAGAATTGAAAGCCAAGTGAGCTAGCCATAACAAAAGACTATGAAGAAATCCTCAAAACTTCTGTTTTT

ATAAGCT

>CK178894_bb No definition line found

GTTTTATCCCTAACTGCACATTATATTTATGTGCGAAAGAAATGTTTGGGCAGCAATACAGAAATGTACGATTTATGAAA

AGTAATATATATGCACTAGCTGTAGTTTCATTATTTAGGATTCATAAACAAACGTGCTACACCTTGGGGTGAAATGTTTA

CGAGCGAACAATCATGCAATGTTTTTCCAGTGATGCCTTACTGCAGAGGAATGCACTCGTTGAGCAGTTTTAACTTATTT

GCAACGTCTTTTGACAGTTTTTCAATCTGTTGTATTGTGGCTTCTACCTCTGCTACTGATTGCTATTGAGCAAAGCTATG

ATGTAATTTAAGTATTCTTTGTTGTGATTATTTCACACTGCAACACTGCCACAAAGTGCAAACGATCTTGCTCGTTGCAC

AATGCACATTGACACTAGATTAGTTTAATTCTGTGGTTCCTGGAGCCTGATTGCAGAAATATTTACAGTGATTTAAGTGG

TACTATCCCTACAGCAGAAATTGAACATGCTTATCAAAAACGAAGGTTTGCGTATACCATGCAACAATTTGAAACGTTTC

TTTCAAGCACTGGTTAGTCTTGCTTTCGTGGAAGTAACGCACAGTGCATCTTTTCTTTTTCAAGCAACAACCACGCTTTC

TCGCACATMGAATCGTAACCTAATATRGAATTWGTAGGATAGGCGTGCGTCGTAGTTGCCAATACAGGATATTTACAAAA

ATGTTATCGGATGTTCAGGCCATGGATCAGCGAAAGGTTGTTCACAGGTGCCAGCACACTTGTTGCCTCTTTTTGACGGC

ACGTGTTGCTAGTTATTCAGTGTACATATTCTTACCAATATTTACAACTTCTCAAGGACATTAATCTCTTTTCCAAGATC

TGTTTGTAAATATGGAGTTCTGTTCGAGGCTTTGATAGAAATGCT

>CK178916_bb No definition line found

TTCCTTTCGAATGTGCCAGTAATGCTGTGTTGTGCACAACACTTGTTGATATTTGATGAGGAGCCGTGAACTATAGAACA

CAAACACGCAATCAATTGCGGGTTTCTGCAGCTGTCGCACTCTCAGCACTGCAYTTGTCGCCAGGGTACTCTCTGCACAG

TGTTCATAGTGTAACGATGATAGCAGCATCTAAGGAGCTGTTTGTTTCTTGAAATTTGACTTTACTGAACATTACTGCAC

TTGCAATCATGCGCACAATTGACTGTCGGCTCATCTTTTTTTCCCCCAATTAGGTGTTATTCGAGCAGTGCCCCTGTGTT

TTCTTTTCTTATTATGATCTAGCAAGCTCATTATTATAAGCTTATCTGCTGGCTGTGTTCAAAATGCTTTATTTTGTCCA

CATTTAGTGCCCAAATTTGTGTCAGAAGTGTTTATTCTTCAGCCCAAACTCTTTTATGTAATAGCTCTTGAATAGCCTGC

TAAGTGTTTCTCCCTGGCTGCGCTGTTTGACCTCTGGGACCAACAATATGCAGTCGTCATCCTGTTCCATTCTTTCTCAA

AAACAGCGAAATCTCCCCAGTAAAGCTTTTCGACAGTCAAAATAAAACTGCCCATTCTGGGAACCATATTACATAARAAC

ATATTGCTGACACCCTGACGTTGCTGGACGTAACATCATACTGCTTGCTTTTATAGCAATCAGACTGTGGTTATAACTCA

TAAACACTTCATTTATTTTGTACTGCACTGTAAGCTCCTTAAATACATGGTCATGCTCTGTTGCAAATTAGTTCTTTTGT

ATGTATATGCTAGTGAGAAATTATAGGACAGTGATGTCAGTGTTTTAGTACAGTAATTCGGTCTTTTTCAAAGCATAAAT

TTGTAGTTACTCAAACACCGAGCTCT

>CK178961_bb No definition line found

GAAATAAGCCATTAAACAAGCATCCTAGTTTTGATCAAACCACAACAATGATAGAATTATCATTTTTGTCCTTTTGTCAA

TGTGCCAGTCAGCTCGAACCCAATTGACAACCGTTGAAGTGTATTTTATATTTGTGATTGAAACAACAAATAGTTCATAC

ACTTGATGYTATGWATATYCTTTTGGCACATTCCAWTTTAATGCCTTCAGGTGTCGCACCTAATTGGTTGCAAGGGCTGA

TATACAACCAGGGTCCAGGCGTACTAGCAGTTGGGAGGGCTGTGTTTCAAATAGCTTGCTGTGCAGCGAGGACAGCACTT

TTATGAGCAGCCGCTTATTACTTTGGAACTCTTGTTCGATTCTCTCAAATTCTGAAAACTGCCTTTGAAAAAGCTGTTTG

TCGATAGAAACAGGAAAAATGCCATGTAATGACTTGCTCAAAACAAAAAGTGACTTGAAACAGTTATGCACAGGTTGCAA

CATTATCATGCTGTCTTTAAAAAGGGAAGCGAGGTAGGCGTTGTGCACGTGCTGGATGGCTTCAAAATCTCGAGTCTCCA

TCACTTGTGTAAGCAAACAACCCAACTGGATCTCTATCACATCCACCTGGAGGTAGTACTGGATGCTGTTCAAAAGGTGT

GCCATTTCTGCCCGCAACAGCATCATGTCTCGCATCAAATCTGATTTGTGCAAGTGCCCTGCACGTGCAACCTTGCACTG

AAGCATCCAGCACTCCTGAAGAAAAGTCTGAGCACGCTGCAAGCCAAACAGCAGCCGAAACAGTCGGTTGTACTTTGACA

TGATAGACGAAGTGAAAAACACGTGAAGAGGTGGCTCAACCTTGTAGTTTTAAGCCTATTGTCTCCCAGCAAGCCAAATG

TGGCTCACTGCTGTCGTGTGACYSTAGGAAAGACAAATAGATAAATTCATTATACACAAGGACGCGGTGCAGCATAATCT

ACAAAAGCATAAAATTCTCAATCTAGCAACTACAACAGCACGCGCACTGTGGCTATGTGAGATAGAGC

>CK178993_bb No definition line found

CCTTAAGGAATGGGGGTGGGTGGGGAGGAATACAAAGGCACACTGTGCACACACTTGGATATGTTGAAAAATTTAGGATA

ACGCTAATTATTTCACTATTTTATATTGTCTCAGTGATTTATCAATTGGCTGTTTCATTGTGGAGAGAATTGAAGTTTTA

TACATGCTAGAAATATTATGAAAACTATATTTTATTTCTTCTCTGGACTAATACATGATCTATGGATGTTTTCTGTAATA

CAACCTATTTTAGTTATAGCATAATCAAGAAATAATGTTATTCTTTGAATTATATTTCATAATTGTACATTGACAATTTT

ATACAAAGTTGGTAGGAATTTATAATAATAATATCTGGGGTTTTATGTCCAGAAACCATGATATGATTRTSRGARRCATC

GTRGTGCRGGGCTCYRGAARTTTTGRCCATCTGGTGTTCTTTRATGTGCACTGACATCATAGAGCACATAGGCCTCTCAT

TTCGCCTCCACCGAAATGTGACCGCGGCAGCCGGGCTAGGAATTCATGCTAAAATGGCAAGGTAGCTAGTGACTCCTGCT

TCTGATGTAGTATCTTTATCTAGCTACACTTGGTTTATGTACGCTCTGGTATATTTGGTAGAATGCATAACCTTCTTGAT

GACAGTGTGAGAAAATGGGAACAAAGAATTATACTGAACACTGCAAGTGCTTACTCACGACTAATGTTCCATTGAAACAG

ACATTTGAAGGCTGACAGAACCAGCCGTGCAGTTCTCAGAACTGAATGATACCTCTTCTTAGAATCGGGGCACAAAAGAA

ATAAGGATGATGCAGCTTTTAACGTTGACCTCTCTTGGACAACA

>CK179098_bb No definition line found

AGAGTGGTAGAGCTGCGGTAATTTATTCTAATGGGGCCAGAGCGAGTCGTGTCGAGTGTTGTCAACCTTCGTTTCAATGA

GATCCACTCGCATTTACATGATCCCCGAGAGGAAAACGTGCGCCAGACTGCAGCGCCGCTATTGTCGAGTGCGTGTAAAC

GAACCGCGTGTCACTCCTGAGCTCGACTGCAATTGCTGTCGAACAGGAATCATGTTCTTATGGATTCCTTGCGCCTAATG

TTATACGGTAAGTCAAGTGCATTTGCAAAGCGCTTGTTTGAGCGAAGACCCTATTTGCGTACTAGCACAACTTGCATCAG

TGCGTGTTGTGACTTGGGAGCGCTAGAATACAATGGCTGCCTATAGTACGTGCACCTTTAAATGGATTTTATTTGTTTTT

TTTTCTTTTTTAAAATTATTATTGTAGCTTTACCTCGGAAGCTTCAGTTTAAGGGTATTTTGTTTTGCTGTTAGGTGGTT

CAACACACTTATTATAGCAACATAAGGAAAAAATTTTATCGATATGCTATACCCTGGCTGTGAACCTTGATCACGAATAA

TTTATGTAGATGTACGTAGCTTCAGCTTCTATTGTACAGGGGATATGATGAGCGCTTGAAAAAAAAATGGGTTCTCCATC

CCGTTAGGCTGGGTCTCCCTAAATATGTCTCGCGATGGCGTGTTGTCGTCATGCGTAGAACGTACAGTAAGTATAATGCG

TAAGTATCTGGTCTGCCGGTGTGAGTGCGTGAAATAGTGCGACCAACAATCAAACAAATGAAAGAAAAAGRCTCCAAGAT

AGCTGTTTTTAAACTGCGATTTGACAAAGCAGGCTTTGTTCGGCGGTGCGTCAACCGGCGAACAAAGTTCATCGAAGAAA

CGGTTGAACAAGTCGATTTTATGTCAGATGAAATGTACAGAGCATTACCGATTTCCTTGCAGAAWAATTGYGAYGCACGA

AAAAATGCAGAAACTTAGCCAAAACTCTCAC

>CK179138_bb No definition line found

ATCTTGGCTGCGTCCTACTCCACAGCGTGTTTCTTTCCATCTCCTTCRTTCACTATTTGTTATTGCGARCCGCGTCCTTG

GATTCGGCCSGAGGCATCATCAACCTAGAGGGAGAGAGKGAAAGAGATACATCGTAGATCCTCCCCCGCTTTAGAGGCAA

AGCAACGACCCATCAGKTYKATGATAKMCTYGTTTAATCTCGGGCACTGCACAGTGTCGTTCCTGTCCTTGTTTGGTGAC

CACCGCCCCCCTTCGTAAAGAAGTCTGCTCGTTCCGGGATCGTGTTTGTGTTCCGTTACGGCACTCGGCCCGTTCGAGTG

AGTGGTTTGGCGCTGCAGCTTGCGGAGCGCGGTTTCTTTGAGCGTCGAGCTCGGACAGAGAAGGACCACCGAGACACCGT

GGCGCTCGGACGGTCAACGCTTGCCGAGGTGACTGCCAGTGTCATCGTCGCCGCTCCGAGCGGAAGATTTTCTTCCGAAG

TGCTTCGGACAGAGCGGCATTGTCGACGGTGAGACGGTAGTGCTGCAACAGAAAGTGTCGTCGGCAGCGGCAAGCTCGGA

GACATCATGGACATCAAAAAACCGGAGGAGATTGGTGGCCGTTCCTGGTGGAACCGGCGTACAAAGATGGAACGGTGCCT

GCTGGTCAGCGTTGGGTTCCTCACCGCCATCGCCATCATACTCATCGCGACCACAACGCTGGCCATGGCCAGGCCCAAAG

CATTGAAAAGGGGAAGCCAGGAGCTCTCGACAGAGGTGTGCAACTCGACGGTATGTCATGAGCGAGCACAACTGATTCTG

CAGTCCATGGATGAGAGCGTTGACCCGTGCCAAGATTTCTACGGTTACGTGTGCAACAACTGGATGCGAAACAACCCAAT

ACCGGACGAACGGGC

>CK179199_bb No definition line found

ATGCACATTAAACCTTTATTGGGGGAATTGGGAAACCAGCTAAAAAAATCATTTTTGAGCTTTACATAGCAACCTTGCAT

TGATTCCGCACTTACATTATCTAAAATTCAACTTGAATACATACATACAGGTATGTTGCGAATTCCCAGCATGCCACAAA

AGCAAGGATCTTGTAACAAAAACGTACTTAATAACTTTGTAACATGTCGGGGGCAACCTCTTGGGTACGTAAACACCAAC

AAATTTATAGAAAACTGAAGTTACGTCACTCAGGCGAGAAAATATGAAACGAGAGATGTTCACATGCATCGTGCTTTCCA

CCATCAGCTACACAACACCTGCTTTGAGCATAAAATTCTTKACACATCACAGTGCTAGATATTTCCTGCTGTAAGCTATG

TTGTTTGTGAGTACTTCGCAACTGGCGTTAAAGGCGGCACACATTTCGTGACTTGCTTGCTCAAAGTTTTCTGGAATGTC

ACATATTGGCGGCACACGTCAGTAAAGGGTTTTGTAAAAAATCACCTTTTAGTACAACAAATCAGTGATGTAACCTCCAC

ATTAAAAACACTGGCACCCCTGCCCTTCTAAGCTTCATTTGAATGACGTCTTGAGATATGGCGACTGAGGGCGATTTTCT

GTGAAAAGGATTCATTGCAGTAGATGC

>CK179215_bb No definition line found

TTCTGATTATTGCAAATACGTGTTTTATCTCTTGGGGCCTAGAATGCACTCATTTACTTTATACTCTAACTTCGTTCTAT

ACCGTAAAGTTTGAGAGCTTTTGAAGCTCTATGGGAGCTTGCCGAACCGACTGACAGTTTCTTTCCGTGAATAAGCGGGG

CGTGACTAAGAGCTACCTAATGACACTTCCTAAGTATGCTTGAASTTSTAGTTMTTCATGRGTTTATTTGCTGCCGGTCA

GCTATATGAGACCTCAGCTCCCAGTTGGGTCAAGCTGTTCTTTTTTGATGCTTGAATCTCGCTTAACTGATTATTTTCAG

CAAAGCGATTTTACTTATAAAAAAACATGCCATTTCATGACAAGCACTTTACACTTGTTGCTTTCACAATGAGTACACCA

GTCCTTATTAAAGCTGTAGACATCAACAGAAGCGAAACTCGAATAAGCTTGTATTTGCTTTGGCATGAGATACGATGACG

CACAGAAAACAAAGGACAAAACAAGTCAAATGAAAAGAAATAAACTTTCAAGTTGAAAACCATCTCCAGTCATTTGTGTT

CTTGGTCTCCCTTAGCTTCTAGGCGTTGTTCTCTACTAAAATATAATAATAATAATAATAATAATAATAATAATAATAAT

AATAATAATAATAATAATAATAATACCCTAGTTTGCCTGTGTTTTTTTTTTTGGACTAATCCACATCATTTGAAATGTTG

CCATAGGCTTGTGAAGTTGTGATAAACTATAACAGAATGCACACGCTGCCCACATATGCCTGTTTGTTTGATGCTACAGA

TTCATATGGGATTCCAAGCAGTTTTTCTGCAGTGAGAACTATTTGCATAATAAAAACGAACAGTTTCAATTGCATGTGTT

CCTCTTTTTCATTCCCATTAGAGATAATAACAAATAAAATTAAAGTGATCTGTTGA

>CK179228_bb No definition line found

CAACTAGAAACTAGCAGCACTGAGCATGCAAATTATACTACACGCATGGCTGGCTGTTCTGTTATCTAACAAATGCACAA

CTTTCATTCTCAAGCATCTTGAAGCTGGACACCTATGTTTTCTTTACCTCAAACAAGGGCAAAAACAAAAACGTCTTGCT

TTGTTTCACAGTCTCATCTCCACGATCACCAAGACCATTACTGTCAAGTCTCTTCTTCGTTTGGCTACCACAACATCGCC

CGTTCAGTTCTTTGTGTACTCATCCGCTAGGCAGAAAAACTACTTTGAAAAAGGTTTTGCCAGTTGTAGATTGTAACACA

GTTCACCTGCGAAACTATTCACGTAATTTACGTTATACAAGGGTTAGAACTTGCTTTAGAAGTAAGTTTATTCCTTTGAG

CATAACTATATTCAATTTTATGGTCTGGGAAATGCATAGCTTTTCTATTCACTGCAATATGGGTAACAGCCATTGAGGCA

GTTCTTCCAGCTAAGCAATTTCTAATAAAGCTGGGACGTAAACATCATGCTTGAAGTTTAGACGGCACAAATGTGATAAT

GCCATGCAGTTCCAAAAACAAGAGAAGGAAGTGTTAACAAAAAGTAAAGTCGGAAATAAGAGGCATTGATGAGAAGCATT

AGAGATGCTATTAAAATGAGCACTGTGTTCCTTATGTTTTATAGAACCGTACTGATGCACTGAGCTGCGACATTTCATGC

AAAATAAAGCAAAAGTGAACCTC

>CK179275_bb No definition line found

TAGCACCACGAACCAGTTTTATTGCGAGTCGAAAAATGCTAGCCTTTGCCTTGACGACAAAGTTCGTTGGAGAGGACACC

CGCCCCTTCCCCACCTATTCACATTGTGCACTTATGCAGTGGCGTGTTGAAGCTCTGAATTTGTCTTAGCGATATGAAAA

TAAAGGTAGAACCAAGTAATAAAAACATCGGCGGCCAACAAAAAAAAAAGGTTCATCTGCAGGAAAACAGAAAAYGAGTA

CTTGAACTGATCACACATAAGGATGATTATAGATTATGTCAGTTCAATGGATAAATCACTCATATTAGTCAACCAGTCAA

GGCTTAATGAACACGCTCAGTGACCAATTCCAATGAGGGTGTTATATGGAAAGATAGTGACCACTATACGAGTTAGACTA

CCGCAACGCGTCGCACGACATTGTCGGTTCTTCACCAAAGGATGCAACCCGATTTGCCGCTTGAAACTACATCTTTGTGG

GCACAAGGAGAATATGGGAACCGAGAAATTTTATGAAACACTTGCTCTTGGCAAGAGAAGTCCGAAAACAATTTATCTGA

ATGGACGAAACAACTGCGATCCATTAAACTATTGGACCAGAACTAAATGTCTGCTTCGAACGCTTCTTCACAAAGTTTTT

GCACGAAATTGATTGATTGGTTATAGCCAGCCTCAAGGTCAACTACCCAAGTATCAAGCATCGGGACAAAACCAAACGGA

CTTCTAGTGCAACACGACGAATTCTGTGCAAATCAGTTCGCTGACTGATGGGCAAGTTTCAACGCCGGAAGAAAGCCCAA

CGGGCTTCGACCTCTGCAACACTTAATTTCGTGCTAA

>CK179372_bb No definition line found

ATTAGGAGCTTCATGGAAGAAACCAAGATTTGCGCAGCTCTTCTAACCATGGTCATCTATGCTATCGCCTTGCCCACAAG

CCTGAACGAGCCCTCAGGCAGCCCTCACATGAACTGGATGAAGGGTGTAAGTAAGGTGGCATTAACCAGAAAAGAAACCC

GAGCACAAGAAGCTCAACAAAAAGTGCCTTGCGAAACAGATGGAGCACCCTGCAGAAAAGGGGTTTGGCAAGGCCAATGT

TTCTATGGTGAATGCGTTCAGGAATATATCGGCGCCCCCAATAGCGACAAGGACGACGAAAACGAAGAACTCTGCTTGAA

GTACAAGCTAAACACGACGGTGAACAATGCGGTGACAACTTGCTCTTATTGGTGCTCAGAAGGGTCAACGCCATACAATG

TTTATGTCCTGAATGGGATATTGTKTSMKAAATCYGAAAACACCAACGGTATCTGCATGGACGGMGAGTGCGACACCRAG

CAGCAGCTCACGATGGCGGTTTTATACAATCCCACGCCYAGAAAGCAGAGCGATGAACTAGCCTCTGGAATTACGAGCCT

ATCACTTTCAACCRAAAGCAATTTTACGCACAGCACTACCAGTTCCTTTGGCGTGACCTTCCAAACAACGACGAACTCCA

ATCCTCGAAATAGCGAAGCCGTAACGAATGTCCAATATACGAAAGGTATTAACGCAGCAACTAAGCTCGAAGGCACACGT

CCAGTTGAAGTAGCGTATACAACCAGATTGCCGTACGTTATATTCACCGACTTCTTTCAAAAATTTGCAACTGCGGGAGT

GCCGCTAGAGTGAAATACCCTTTCACAGTGACAAAATTGAGTGTAACTGAACAGTAGGCCTCTTTTTTGACTCCTTCCGC

WATTTCAAATGCTATTTTTTTCTATATGTGATATATCCCGGTACCAGTTCTTAGAAACATCAGCTGTTGCCTTGCCTCAG

GTTTTCACCAACGTTTTTAATGGAGTCGGATATCTTAAGTTAGCGCAACCTTAAAAGACAGAGCTATTTGTACTAAAAGG

TGTGATAATAGAACTCTTCCCAA

>CK179416_bb No definition line found

CTTTGGAGCACTTAATTTTATGTACACAACTTGTTCATTTAATCAACTTTTCTTTTTCCATTGTATCAAGAAATATGTCT

GCTCATCACTTAATCTGATAGTGATATTTAGTAAAATAACATTCATGCTTGGTTTATTATCTTTCATAGAACAAATTTGC

TTACATTTAATGTTGGGAAATCACTCAGTTGGGAAATTCAATGCGAGAAATTGCTCAGTTGTGAAATTCAATGCCAGGTG

AAGTTAACTCCTGCTTTGCATATTTGTGGTTAAAAATTGTCTTTAAGCTGTGTTCACAACTGTTGACTAATCGGTGCCCT

GCTTTGTGTTGCCCTCCACTACGGCATCTCTCATAATCATATGGTGGTTTTGGGATGTTAAACCCCACATATCAATCAAT

CAAGAACAATTTACCAGTGCAAAAAGACGAAACACTTAAGAAACACACACAGTGCTATTTATGTGTGTATGTATGTCCTT

GAGCGTTGAGTCTTTTTGCGCTGGTAAAAATATCTCTTCTATAGTACCCGCAGTATGAAAGAAAAGTCGTTAGGGGTGAC

TATTCCGTACCACAGCAAATACTTCAGTTCGAATGTATCAATACTAATACAGAAATGAACAACAGTCTAAATGGATGCAA

AAGTAAGAATTGCTTGGGTGCCTGGTGGATGCTTTATCTAGAGTTCGTTGTGTTATTTGAATTGGAACTCAGGCTTACAG

CTATATACACTGATGACTCTTTTCTGTTGTAAGCATGCTTCCATTGATTCTTTTATGTTAAAACCACAAATTATAAATGC

CAAGAACTGTTACACCATTGCTTGCACAAGTTCCTACTTAT

>CK179477_bb No definition line found

GATATTTATTATAGGCTGGGTAAGAACATTCTTGACTTCATGTCCATGAAATCCAGTGGGCTCCCACAGAACGTAGTATT

TACCAGGGGAGGGAATCGCTCATTTGCTCACAGAGATCAGGATGCCAATTTTAGTGGAAAGAAGAACATTGGCATCTGTA

ATTTTTCACTCTCTGTAATCAGGTAGTGCACCTTTATACTGAGGCGTGACATTCCCTGAGCTTGCTATTGCTGTGAGGAA

CCCTCGATGCGCTATTGCCGCTTGCCGTTCAGTTAAACTGTGCCAGCATAGTGCGAGAGACGGAGGGGCCATTTCGACTG

AGGAAGCTTGTGTATGGCTTGAGCACTGCACTGCAAAGGCAATGAGTTTTTATGGCGATTCGAGACATTTAGTAAGTTTT

CCTGTGGCAGCTATTGGTCGCATTTAAAGAATTCATGTTCAAGTCTGTGGTCTTGTCTTTTTCAAGCTACACTCACTAAT

GCCATCATCCAGTGCTGTCATTGTCTGAAGCGTGATGCAGCTATGCTTGCCACCGATAATGCAGTGTTACAATGTGGTAC

TGCCCTCAGGTTCTGCTTGCTGTCCCGGGTGTATGTTTGCTGTCTTTGCCTCGTGGTGAGCTGTTAGTAAGGGGACACCA

TTTTTATGCAGTTCTTCATTTTGCTATGACACAAGATGGCCGTTATTTGCACAGTGTGTGTAATGCTGACTCCAGGTCAT

AATACTATCTGGCTGTATGGCTAAATTAATATCTTGTGAGGCCTCATTACTAGGCAGTTGTGTGTGGCAAATATTTTGTC

TTGCATTCAAGATCATTTTGCTCTCCTGTCGTTTGCATTACAGGCTGTGAAGCATCGAGCAGGTATAGCAGTAGCATTGC

GTTTCGTTATCCACATTCATTATTTTATTATAGTGCATGTCATCATTTTAGTA

>CK179508_bb No definition line found

ATTTGAACATGATGAAGCCCGTAGGTTTTGACAACTGTCGTATCCCAGCTTATCCCCTTAGAAGCACAATGCTAGCAGAG

TGGAACTGCAAAAAGCGCTATTGATCGAAGCTAGGCACCACACAACACCGTGACAACTGTTCTTTAGAGCACAAGAGAAA

AAGTCTTGCAGGTACATAGTGCCTAACAAATAGGCCCCGTACAGGACAGATGCCTTGGCGGAATAAGTGATTTTCGTAAA

CAAGGCCTTGGTCAAGGGTACATATGAACCCTGCAAATAAAATGAACTTGGAAAGTCTGGTTAGAGCGGCTGCAATTACG

CTTAGCTTCAGTCTATGTGTGCATATGTCAGTAGGCAATTAAAAGCTGTCCAGCATGCCGAGAAGAGAAGAAACTGTCAA

ATTAAGCTACACTGCAGAACCATTAAGAACAAAGGTGAACGACTACGGATTTCACTGCGCTGATAAAACTGTACCTAGAT

GTGTGGTGAAATAAGTGAAGTCTAAGACAAGCTTGGAAGACCATGTTATGTAAAGATTCTTATCTTTTCTTCAAACTTTT

GTCACGCCCTAGTAACTGGAATAGTATCCCATTCACTAGAAATGAAACAGGCCATTCACAGCTGTTCTGTAATGCTTATC

ATTTTTTACGTTTCTTGTTATATGTCGTTCGTTGGTATATTGACATACCATTATTGCTAGTATAGGACATTTAGTGCAAT

GGGTAGTGGAAGAAAATAATTCTTGCACAGTATCTCTGCACATTATTATTTCAGTCCTTTAATATACCATCTTCACTCAA

GAAAAATAGGAAGCCAGCAAGGGATCTTGCAGTGACATATTGCAACACTCCACTCTCTCTCTGTGACATGTACAGT

>CK179548_bb No definition line found

GATTGAGCACCAGGAGTACAGCAAGTTCAGCAACTTCCAAGTGGCCACGAACGTACCCTTGATCATCTCCCCACCAAGGT

CCCAAAGGAGCTCGTCACCAGCTAAAGTGGCTGGACGCGTGGATGACCGGGTGGCATTGGTAGACTTATTCCCCACGCTG

GCCTCCCTGGCGTCACTTCCTGCGCCTCCCCGCTGCCGGTCATCTGCTGATTCGTCGCTGAGTGAGACGACCTGCACAGA

TGGTGTGAACTTGGCTAATCCAGTGCAACGAAAGGAGGTGCTGCACCAGTACCCACGGCCTTCTGCCACACCGCAGTTGA

ACAGCGATCAGCCAGTGCTTGGAGATATCCGCATCATGGGCTACTCAATGGTGTCGCGCGATTACCGCTACACGGAGTGG

ATCGGTTTCGACGCGACCAACTTCCGCCGAAACTGGACCAACGTGTACGCCCGTGAACTGTACAACTTGAACTCTGACCC

TCGCGAGGACAGCAACGTGGCGAGCTCGCCAAAATACAAGGATCTTGTCGTCGCGCTGTCTTCAAGGCTTCGCGAACTCG

TCGAAAGCTGAGTGGTACCTAACGACGCCCCCGTGGACTGAGAGAGAGAGAAAACATTTGTTTCCTAATCAAAGTGAAAT

ATTTAGTTGGCAGTTTTTTTTTTTTCAGTTTGAAAAAACCTTTTTCGATTCTTGTTTTCGTGTCTGTTCACGCGAATTGC

GCTCGGAATCCTGGTCTGAGGCTGCGAGCCAGRRGCGTGCACTCAGAAAGTTTTTTTTCGGGTAGRAGAGSGGGAGCAAA

CATTAYGCYAACCAGATAATCATCAAGTAAAAGCTTACTTGAGTATTTTTTSRAAGTGTCRCATGCAGTTGATRMAGGAG

ATTGCCGATATCACCACGCTCCGGCTTGTCGACGTTGAATTGCTGGCCGTCTCTATTGCGCAAGTACTTTTGCTGGTAGC

TTACTTATAGATTTTTTTCAGTTTCTTACGTTGCCACTCCCGTTAGATCACATATATATTTCGTAAATGAGCCCATTAAA

AAAGCGTGGCCATTAGTGACATGGAGCACACAAGAATCGTGTCACGCTGTTTTGTTAAAACTCTCTGCGCAAGGCATGGT

TTGAT

>CK179552_bb No definition line found

GATATTCAACATCCCTGCAGGTCATGTTGATATGCAACATGTAAAGGAAGCTCACAAAATAGACTCCTCCAACGTGACCC

TTAAAGTGATGCCGGGCATAACGCGGTGTCACCTGGATCCTAATGGATTTGAGAAGACGAGGGTCAGCTATGCCTTCCAG

CTTTTTGGGACAAAAGTCCTCCAAACTTTCCACCTATACAAAGACAAGTTGGAAACAACACTTGAAAGGATGGATGTAAC

ACAAGAGTTCTTCAGCAAAATCCACCAGCTCATAAGAGTGATGACATCGCGGTTCCCTGCTGAAGCCCTCAGACCGTGTT

CATCAGGAACAGCAGTGCTGCAGGATTTTCTGTCCTGCAGCATGGGAGCAGCATACGAAAGGTGGAGGAGGCTTTCTGAG

CAATTCCACTGCTGCTGGCCTACGTGTGACCATTTCGAGTACACTAGAGTTGCTTGACTATCTCACTGAAAATGTTGGCT

TTAAATACCTAATGACTGCAAAAACTAGCCAAGGATCCCTTGGAAAACCTTTTCGGTATTATCAGGCAATCTTCTGGCAG

TAATGATCATCCAACACCAACTCAGTTTTTGATTACAGTAAACWGCATGTCATTCTATGGTTTAGTTAAAGGTGTCAGTC

AAGGAAACTGTGAAGAGGGCATGCTAGGTTCCCTTTTGGAAGTTAGTAGCCCACGAGACACATTGCAAAATGCAGCAGGC

ATGAATTTGGCACCGAGCTCTTCTCCGAATGCACAGGCGTTGGTTATACCTTCCCAGGACCACAGCCAGCATGTTGCGAG

GAGCGACTCGAGAGTTATATTTTACATTGCTGGCTACGTGGCACGAAAGTGCATTTTGAAAAGCAATTGCAAAGATTGTT

TGATACTACTTACCGCGCCTGCCCCAGACGAAAGCTTTCAGCTTTGCAGGTTGACACTATTTCGTGACCACGGTGGACTC

CTCTACCCAAGTGC

>CK179636_bb No definition line found

CTTCTTTTGTCCCCTGTACTCTTGTTGCGTCACGAATAGACAGTGCCTCTCCCCTCTCGCCTTTCGTTTCATTGACTGTT

GTTCGAGACACCAACCACACATACAGACACAGAGAAGAACTAGCGCTCTTCTTATGTTTTTTTTTTTTCTTCAGCTCTAT

GCATTTCTTCCCCCCACCTTTTTTTTTCTTTAATTTTGGCAAGGTTGTAAGTATTTACATCTTGTTTTCCTGCAGATGAA

AGGATGTGTACCGAGTGCTCCCATGCTTGTTTTTTTTTTTTTAATTGGGATGGGAAAGCAGAGAGTGTTAAGTTTTCGCG

CCTAGGAATTCGAGGAAAGTGTTTTTTTGACACGCTGTACTCTAGACATGACTGGATGCCTTCGSAGCATTGCCTGCATC

TTAGGCACKCCAGCCATTTCTAGATGAAACAAGGTTGATTGATAAAAACCAAAACTGCCAAGCAATTTGTATGCCAATAT

ATCTGGTAATAAAAGAAAATCATATTTAAAGGTGCTCTGGCAAAAAGCGGCGTCTTCTTTTTTTGTGTTCTCTTGCCTCT

GGCACTGTGCTAGTGTTAAATGAGAAATCGCGACTTTGCATAGCATTGAACGCAAAAAGACGTAAAGGCCACAGGAATAT

ATTAAGTGC

>CK179690_bb No definition line found

GTAGGTAGCTAGGTGTCGCTAGCACAGAGGCTAAAGCAAACTAAAAGCAAGTTCTGGTGGAGTCATACATCCAGTGTTAT

AAAGTTGATGAGGTCATAAGTAAACATGGCGTCGTGCGGTGCAGCTGGGACAGGCCCCCTCGTCCTGAGGGTGATCTCAA

ACACAGTTAAAATAGTTAACAGTGCGGGAAARATTATTAAGGACATCATGAACAGCGGGAACCTCGGCATCGTCGAAAAG

GWWGGTYRTCAATGACTTGCAAACCGAGGCAGACCGCTCTGTCCAGCGCTGCATTGTGACCTCCTTGTCCCGGCAATTCC

CTAAGCTGACCATCATTGGAGAAGAGACGCTAGAAGAGAAGAAAATCAGTGATGACTGGATCATCACAGACCACGACAAG

GATGTCCTGTCAACGACGCTGCCAGATGAACTCAAGGATGTCAAAGAGGAAGATCTTGTGGTTTGGGTGGACCCCTTGGA

TGGGACAAAGGAGTACACGCAAGGCTTCCTGGATCACGTGACCATACTGGTTGGGATTGCTGTGCAGGGTAAGGCAGTCG

GTGGTGTCATTCATCAGCCTTACTACAACTACCAGGTGAGGAGGATTCAGGTGGAGAAAGACGTCTACAAGCAAGGACGG

ACAATGTGGGGTATCGTTGGTGTTGGTGCGTTCGGCATCAAACGCACGCCGCCCCCTGCTGACAAGAGAATCATCACCAC

AACTCGTTCTCACTCAAGTCCGGTCATCAACAGCTGCATCGAGTCCATGAAGCCGGATGAGGTGCTTCGTGTTGGTGGAG

CAGGACACAAGGTGCTGCTTTTGATTGAAGGCAAGGCACACGCCTACGTCTTCCCAAGCAAGGGATGCAAGAAGTGGGAC

ACCTGCGCTCCGGAGGCAATCCTCCATGCCACTGGAGGACTCTTGACAGATATCCACGGCAACCGGCTACAGTACCACAA

GGATGTCGAGCATGTCAACAGTGGCGGTGTCCTCGCCGCCTGCCTCAGGGAGCAGCATGACTGGTTCAAGAATCACATT

>CK179782_bb No definition line found

TTTGAAATTATTAAAGTTTATTACATTGAATTGAGTTTATTTGCAAAAGTTTGCAAGGAAGACCAGGCAAAAAAGCTGTA

TTGAGCAGGTAGAGAGAAACATGGCCGCCTCATACCAGCAGCGGAGTGGTCGAACAGAAGTACGCCGTATACACATCGCA

TGAACTAACGGAACGGTAAAGACGGTAAGCAATCACAGTCAGAAATAATGAACATGGTTCTTGTGGAAACATCTTTATGA

GATCATCCTTGTATGTTAACGACATCGCTTCACCACCGACCTTCGGGAACTCATCGAACACCGTCTTGGCCCTGGTCAGC

ATTTCACGATGAAGGAAGCCCAGTGCATTCAGATCTCTTAGAGGTCTACGTGTCATCCCAGACATGTAATGAGTTGAGGC

TTTGAGTTTGGCCGCCATGTTCAGTGCATTTGCAGTCGCCGTCAGCATGGATGTTGGATTCGGCAAATAACCTCCTTTGA

AGACATCCACATGAGCCTTAAAGAAGGTCACAAAGTCCTGGCACGATATTTTTCGCCCATTAATCTTTTTGACCAACAAA

TTTTCTGGTGCCAAAATAGAAAGAACAAGTTCCGGAAGCTTCTTTCGAAATTCTTTCTCGGTGTCTGCAAGACGACCATC

GAACGACTTATCCATTGCTACCTTCTTTCCGGGGTGGGGCATGAGGAAACAGTCAAGGTCCGAAAAGGAAGACAAKATAT

TCTGTCGCAACGTCTTCAGCTCTGCCRCTTGACCACTTGTTGTCTTTAGACGACTCGT

>CK179807_bb No definition line found

CACGACACGTCACTGTAGCGTTCACTTCTTTTGTTTATTGTTGAATTGTGATTGCTTGTGTTCTTGCATCACCGTGTCTT

GCAAAAAGAAAGATTTCCTGATGGGCAATGAATGACTTGGCATTATTCAACACCTTTGTAGTGATGTAAGGCATTCCAGT

GCAATGTACAGTGTGGCAAGTTGTCCCTCCATGGAAGGAAAATGTATGCTGCTCATTACAAAGCGAAGCTTGCTTGCTTA

TGTAGCACATGCATCGCGCTAAAGTTCAATAACTATGATGGCATTCCTTGAAAATCATTTCATACTGTTTTGAGGCATGA

TGAATGAATGAAAAATAGGGACTAGAGAAGAATGCACACTGTCATTTTGGGTGTTGYATGCACTGTGCTTGTTGASTTGC

ATKTGCGTGGTTTGAAGCTCGCATGAACACACTTCGGGTGTGTTGGGTTRAAAYGTTCCAAGAGYGGCACACTTGGAAGT

GCCATAGAAGGTGCTGGTTGTGTTCATTCAGCTCTGGCATGAGTTGTAGCTATTTGCACATAACGCTTTCTATAGAAATA

GGCTGTATTTGAAAAGTGGCAGCATTTAGAACTATGGCAATTGGTGTATAAATACTGACATTGTAGAGAATGCATTAAAA

AAAAATGTGAAATCACTGT

>CK179809_bb No definition line found

AAAAAAAACAACAAAAGGTCCACGCCCTTTTCAGAAGCTCTGCAATCCTACTCTGCCCCGTCTGAACAAAGTGCAATCGT

GGTATGCGAAAAAAGACCCGCTCCGAGTGTTAACTGAAAAAACGTACCTAAGACTTGTTCCGGAAAAGACGCCACGAGTC

ACTTAAAGATGTTTTGAGCTCTCTTAAGCATCTGAAACGAGACGTCTACGAAACGCAGTCGCTACAACGCAGATCGAGAG

CATGAAATGCATCCGCATCTAACCACCATATCCGGCTCCCAACCCATCACACCCCCCCCCCCTTTTTTTTTTTGCAGCAG

AAAAGAAGCCTTTGGTGCCAAATGTGACAGACAATAAACGAACAACTGCTTACGTACGCAGCAGCAGCAAGATTTTAACA

TGTTCTAACCCTTCTGTAAAGGTTGTGCGGTCGGTGCCTTTGTAGGCTGAACCGAACGGTAGGCTGGTCACAAGAAATAT

GCATCCAGCAGCATTTTGCATCGTAGCTATAGGCAGTATTTAGCCAGGTACCTGAGTTTCCATTTCGTTCTGTGAGTCAA

AAAGACATATGCGTGTTTTTTTAAGGTTGCAGCTACAGGACGTCAAGGGTTCTACGAGGTTGTCCAGTTAAGACGACGAC

CAGTGGCCGACGGAAGGCGATATAACTGTAGTAGCGCGCATTTCAGAAAAAATGCGCCGAAACGAGAGGTATACACTGCA

KCAACACTCCAAAAAAATATSGAGTARAAAGGGTGTCTTTTTGTCCCACAACWATAATCGTCAYCAGGCTTGCGTGCRCT

TCCTTTCTTGAAARCTCGGCGCTGGCCACTTTCCTATCGAGAATGCAATGTAACYYTGATAATGGGCATGTCGATCGTGA

CCGGAATAAACCAGGCGTGACACGATAGCGTAAGGATGGAACGCAATATAGATGACGATTATTCTTGTGGGACATAAAGC

CACCCTTTTTA

>CK179846_bb No definition line found

TTACGTAGTGTCATGTTTTATTCGGGAAAAGTCTCTACATAGTTACAGAAATCACAAATATAGCGCGGATGTACTGGTGA

ATAGATGGCGGTGATGGATGATAAGATGAAAGAACGCGCACGACCGTACATGTTTCGCAATGGGACATGACAAGACAGAT

GGCTTTCTTCGTTTGCACGTGAAGGAACGGCAGGAGTACAAAGAACAAAGATATGACGTCATGAACAATATAACAAGTGG

CAATGAAAGCAAGAGAGTCAACACAAAGAAGTTACARCMTCGTTTTTCTACGYGGGTACTGTGATCGYACTTGTAATTGT

ACAATAATAAAACCYGYAACCCTCATTGATAAGGCGGGCTATTAAACTTCTAAGACAAGAGAAATGTGGTGTACACGATC

AACGTTTGTCAGGTTCTGTTTTCCTCAAAAGGTATGGCGTACTGCCCAAATGCGGGTGAGAGAACCTTGTCTGGGGCACT

CAAAGTAAAAGCAAGTAAGAACTTGTGTTTGTAAGAGGGTCGAACATTTTTATTGCGGACGTTTGTATTTYGAAAGTTCG

AACATCTCCGTTGCGAAAAACTTTGCCAATGCATGAACTATTATACAGTAACGCTGGGACATTCAGTGCACATGTAAATA

TGTACTCGTTACTTCTYTTATCGGTAATTTAGTTAATGACAGTGCGGTGGGATAACCATCTTGTAAGAAATGACCAGCGG

CCAGGAAACCGACGTGTACACTAATCTCTCCTCAAGTACACTTTTG

>CK179849_bb No definition line found

CACGCCAGTTGGCCGTCAACGCGAAGCAGCTGAGCTCGTTTATATCTTGTCATGTGTTAATCGTATTCCTTAGGCGCGGT

ACAGAAGGCCAGGTCAAACTAAGCCGCGCCTCCGCGTGTGATCCGATGGGCAACTCCATAAAGCCACATATCCAAAATGC

CGGCAAGACTGGGGTGTGTACGCTGCCCAACAGCAACCTCAAGGAGTTCCCCAAGGAGCTGTTCCTTACCGCGGGCGTGC

TGCGGACACTCGACCTTTCGGGCAACAAACTGTCCTCGATCCCGGCGACGATCAACAAGTTCGAGCTACTCAAGCATCTG

ACGCTCTCGAACAATCGCATCGCATTTCTCCCGGACAGCATGTCTAAGCTGAAAAAGCTCGAGACGTTGAACCTCGGGAG

CAACCACCTGTCGCGGCTTCCAGAAACGCTGTCTGGGCTCAGCAATCTCCGCAACGTCAACCTCAGCGACAACCGGCTCA

CCGCGTTTCCGCACTGCTTCTGTGGCCTCAAACATCTGGACGTGCTTGACCTCTCGCGCAACCGGATATCCGAAGTGCCG

GACTTTGTGGGCGACCTGCACGCCACGGAGCTCAACCTGAACCAGAACCAGGTGTCCATGATCTCGGAGAGCATAGCAAA

CTGCCCGAGGCTGAAGGTGCTGCGGCTGGAGGAAAACTGCCTGCAGATCAATTCCATCCCGACGCAGCTCCTGTCCAACT

CCAATGTCTCTTTACTGGCGGTCGAGGGCAACCTCTTCGAGCTCAAGGACTTCCAGGAGAAGGATGGCTACGAGACGTAC

ATGGAACGTTACACCGCCACCAAGAAGAAGATGTTCTGAGACGACGTGAGGCCCAGAACCTCTGCAGCACGTGGCAAAGC

AAGGAAGAGCACCCGATCAAGCTCACACTTCCCTCGCGATCTTCGCACAAAAATGATGACAGGGCAGTAGTGCCTTGGTG

CGGGAAAAGAAAAAAAAAAATTGTCGCTGTACAAGTAGCCGCTTGCAGCGAGTAGTCATTCCAGGGGCATCGGAGGCACT

GTCAAGAAAGGACG

>CK179908_bb No definition line found

CTCTCCAAAGCTTTTCTGACTTCTTCTATCATTACTGTTGGGGTGTAATCTGGGTTACTGCTAGTTCTTATAGTATTAAG

GTCGTGGTTGTCTCGGCTACTGTACAGATCTCTGTAAAACTCCTCCGCTATTTTAACTATCCTATCCATATTGGTAGTTA

TTTTGCCTTCTTTGTCCCTTAGTGCATACATCCGACTTTTGCCTATCCCAAGTTTCCTCTTCACTGCTTTGACGCTTCCT

CCGTTTTTCAGAGCGTGTTCAATTCTCTCCATGTTATACCTTCTTACATCGCATACCTTACGCCTATTAATCAACTTCGA

AAGCTCTGCCAGTTCTATTTTGTCTGTTGTACTTGAGACTTTCATGATTTGACGCTTCTTAATGAGATTCTTCGTTTCCT

GGGAAAGCTTGCCAGTGTCCTGTCTAACTACCCTGCCTCCAACTTCCACTGCACKCTCCRTAATGATACTSGTCAGATTA

TCATTCATTGTATCTACGCTAAGGTTGGTTTCCTCACTAAGAGCCGAGTACCTGTTCTGCAGCGACACTCTGAATTCCTG

TACTTTCCCTCTCAGTGCTAGCTCATTGATTGGCTTCTTGCGTATCAGTTTCTGTCGCTCCTTTTTCAAGTCTAGGCGAA

TTCGAGACCGTACCATTCTATGGTCACTGCATCGTACCTTGCCAACCACTTCCACATCCTGCACGATTCCTTGGTGTGCA

GTCATTATAAAGTCTATTTTGTTCTTATTTTCGCCATTATGGCTCCTCCATGTCCACTTGCGGTTTCTCGTTTTCGGTAG

AAGGTATTCAAAATCCGCAAATTATTGCGTTCTGCGAATTCTACTAGTAGCTCTCCTCTGGCGTTTCTAGTGCCGATGCC

ATAATCTCCTACTGCCT

>CK179918_bb No definition line found

TAAGATTCGAGCCTGTAAGAGTGCATGACCTTACGCGTCTTGTGTGGCCACAGCATTTCGAATGTTGTAGGTAGCCGACG

GATCTAACCTAGCAAAATTTACACACAATTGCCCCACCTGATTTTGTCTTTTCCAGTTGTAAGCAAAGCACGGGCACATT

TTTAGATAGTCGCACGCAACGCCGCATAAGCCAGTGTCCTGCGAGCTATTCCGCATGCAGGGTTGTACCGGGGACAGAAA

TATTTGGATTCACGCTTACCTGGACTCATCTTACTTGTTGCACGAAACTGCTACCAAGCTCTATAAAAAAATGTCTTTGA

ATAAATACTTGCCATACAATGAAGCAAACAATTAATCCGACAGAAAAAAGTGGGTCATTAGCTACTTATCGGTGCAAAAT

>CK179984_bb No definition line found

TGAGAAAAGTTTGCTTGTCATAAGTTTTTTGTTTTCCTTAATGTCCGAGTACGGTACAGACTGTTTAGTTTTTTTTGTCG

CATTTTTAAGTTAACTTTATTCAAATTGCAACGGCATTGCCGCACTTTCGCATCTGTCATGGCGGGAATGTAGCATTTCA

AAGTGCAGTTTTTGCTCAATCTTTCCTGCTTCTGCATCTCACAAAAATGTGCTCATTATCGTCGTTTCCTCGACCGCCAC

CGAGAGGTCTCATCACCCTGTTCGAAAAGGATGGTGACTTGCACGAAGGAAATGAACGTGAATGGAAAGAACTCGCGGAA

CGATTTCCAGCATATCCTCGTGTATCGATAGTCCGGGAGACCACAAGACACCCTGCTTTTGCGGAAAGTGCTGCTGGTGC

CTTTCTTCAGCAACCCGAGTCCATTTGGAAGCAAGCATTTGTGTCATGGCAAGAGAAAGGATTCATTGGCTTCTTGGAAC

AGTTATCAACATACAATGAGTCATCTATTGACTTATCTATAGCACGATTTGCTGCAAAAAGTATTTTATTCGGCACGAGA

TGGTTTTACAAGCAAGGACTGCTGTTTCCACATTTGCTCTTGTCCAGTGAAGATATGTGCAAGGAGTTTTCATTAACGTT

AAACTGGGCAAGGGGCCACGTACGAGCGTTTGCCTGGCACCCGCAGTGCCCCAAGTTTGCTGTGGCATCACACGGTGACA

TGATTCGCGTGTATGCACCCAACATGAGCATTACGCCTGTGCTGAAGCACAGGTCTCAGAGGAACATCACCGACATGGCT

TGGAAGCCTTACAGCTCTGCAGTTCTGGCTGTTGCTTGCCAGGAGGGGGTCCTTCTCTGGCAAGTGGAACCGACCTCTCT

TATAGCAAGGCCCTCTTCAGCATATGCATCACTCCTCTCTCGAGAGGGGCACAATCCAGTCAGCAGCATTGCGTGGCATC

CTAAAGGAACACTCCTAGCATCAGCTTCTGCAGCTGACTGCTCACTTCTTATTTGGAATGTTGCTATGG

>CK180029_bb No definition line found

CTCGGTTCCACGCAGAATTCACGGCAAACCTTGTGCACAGAGAGATTAGAGGCTACGACGCACAACCTTAACTAAATACT

ACTGAAGTAATGCTCCTGGAAACAACAAAATGTACACATATTTTCAWTCKGGYACTTGTTTKAAAAGCCTGARAGCATGT

AWTTACACTRMTAGCAGGCGAACTTTGTAATAATTTSAAACKATACACACGCACAAACAGAGTAACAACCATCAAGTGAT

GAAAATTAAACCRATATGCTTGGCTTTGTGMACATTCGGGAGCCGCAAAATTGCTCCGCAAARGAAAAAAAAAACACMMC

ACACACACACAAAGYATTGYGCGATGGATAACTTATCCATGACCATACAATGACGTCGAYAACAATTRAAAAGAGTGCTT

CCAAACWGGCTGGGAGTGACGTCGTATGCCYACGCACCCTGATTCARAGTTARAACTAAAATTGACTCGTTCAACATTGT

ATAGCTCGYCTTCGACSACGCAAAATYRTTGACCCAATCGTGAAGCCCGCASWMTTTATTTCCAATAGTACCACGTGCCT

GTTGGTACTGATCATCGCTTATCCTGCTCATTGATTTTTCGCACAGAATTCCTCGCTTTTAGGGTTTCCCATCGGAGGCC

CTTATAAATATTAGATTAGTGAGAATTCGTTTTAGCATAAAAAAATTTTGGTCAAATTGAGTGCTGTCAAGGAAAGACTC

GTAAAGCTTTGAAAATCATCTCGTTTTTATCCAACTGTAATTTCTTTTACTTCTCTCGGGCAGAATCTGCCCTTCGCTCT

TCTACGCCCCCCCACCTTTTGTTAATTTTTTTTGCTTTAAATATTAAC

>CK180068_bb No definition line found

TTAGAGGGAAAAAAATGGGTTTAATATTGCACTGACAAACCTTGTGGCAACATGATTTGCCTTCTTTTTCACAGCGGGAA

ATTAATAACACGGATATAATCAAGCAGGAAAAAGATTAACAGCTGGGCTAAAAACTTAGATGAAAGAAATGTAAGGTATA

ACATAATTCAGCTTTAAGCATCAGTATGCTAAAGCTTCCACATTTTTTCACAGCGCGAAATGATTGAAAACAGATGAGAG

GACAACACACAGGCACTGTGTTCTATTGCACACAGTACATGTATTGTGTCCTGTCATCAGTTCTGGTTCATGTAGTGCTG

TGCAAATGTGTCAATGCTATATTACCAACTAGCCCAACGTACCTTTCTATCATGCTGATGCTGACACACACACGAGACAA

GAAAATTTGGAGTTGTAAGAAATCGGACAAACCTATACATTTCTTTCTTGACATGGAAGCTCAGCAGGTGTCAACAGACA

GTGAACGACTTGTACCAAAGACACAGCTACGTGCAGTCAAAGCTAAGATGAATGTCTTAAGTTTGCTAACGGCTTTGGCT

AAAGAAGATTAGCTGGGAAAACACTGAACCAGGTTTCCTCGGGTTCCTACCCTACTGRTCTCCAGAATCAGGCATCACTC

TGAGCTGCAGAATATGGTGGTGGAGGGAGTGCCCRAAGCTTGAGTGTTCCTTGCCGAGACSKKCRTGGAAGCGAACCCAG

CAAAATGTACTCCTCTATCAAAAGAAGTCTCAAGTCAAGCCTGTTATCTTCTGAATGATATTAAGA

>CK180069_bb No definition line found

TTTTTTTTTTTTCTTTCTCCAGGGCACCAGGACACCAGGGGCCGCTGAGGTTGCAAGGAGAAAGCGAAAGTAAGCAGTAG

CGCATGTAGCRAAAGTTTGGYGAGCTTYATTAGCTTGATCCTCCTTGATATTGCATATTTTAATGGCTATTGATCGTGTT

CATCTTCTTTTRTGTTRGCAGTACGAGAGGCAGATGTATRTTTTGTTGCCTCTAGACAATTTAACCGTTGGGTTGACGCT

CAAGAGCTCTGTCTCTCGTACAAAGTTGATCGATTAGCTATCGGTACACACAGCTAGGCACAGCGGTGCTGCTCCCGAGC

CTCCGCGCATCGAGGCAATCAGTACTGGTGTGGTTTGTGTATAGAAGGGAGACAGACAAGCCAGATATCATGTTGCCTGT

AAAGGTGTTTGTTTCAGCGACGTTATTGCTTAGCTGCAGCTGCTTAGGTGCGAAACTTCAAGACCCCAGAAACCAGGTGC

TGCAGACTACTGAAGTTTTTCATTTTTATATTGATAAAGGAATGTTTGACGCGTCAATAGATAGCTACGGGCATACAGAA

TTCAGAGCTGCTTTGCTAGGAAAGCCGGACTTACCAAAGTGGATGTTCCTACGGCAAACCGGCAACTCAAATAAAGCACT

TTTGTACGGTTCGTATCAAGACGATGGGGAACTCAACATTGAGGTCTTTGCTATTAACAAGTACAACTACAAGACTTCTT

TACGGGTAATTAGTCTTCAGGTGGAAAAAAGGCCAAAGGAAGCACTTTACGAGGTCGAGATGAAGTTCTTAAATCTCAAT

ATCGAAGACCTCTTCGAGGGTGACTGGTTATCTGATTTAGAAGACATTTTTCGAGATCATTTATGGAAAGAAAGTCCCGA

AATCTATGTGACAAAAGTGGCTTCAGTGGTGGATATTGGTGGCAGGGTACCTGTCAATCCTAAGGACAAGGAAGGGATAG

TCGTACGCATTGGTGGG

>CK180090_bb No definition line found

CTCTGTGTGAACATTTTGCCACACAGCCATGTCAAAGCTAGAAACCCCTTTGTGAAAAGGCCCTATACTGGGCAAAGWWT

TATGGTAATAGATGTAATAGAGGAGTGAAATAACAGCCAGGCATCACACAATGCAAATTGCATAACGAGCTTCCCACACA

TAGTAACAGGCCAACAAAGTGCACATAATGCCTTGCAGATGTGTAGCAGGCACCTCACTTTTCCACATAATTATGGATAA

TGACACAGCAGGGGCCATGCAACTGTATTTGTAGTAAACACCCCAAGGGGGCCACTCCATGAAAGAAGAAAGTTTAAAAA

GAAGTATCAAATGTGGAACATAACAACATCCAGCAGGTAAAGCAGTAAATTACAATTCAAATCACAACACTAGCCACTTG

TTTACAATTTTCTGGTGTCCATCAAAGAGTAGGCTGAAATGAAGTGATTGGTGAGGTCATCATCAAGTGTCTTCACAGCT

CTGTGGTAAAGGACACCCGCTTGCTCAGGCTTTCCTTCTGGGTGCTTCAGCTCAAAGAGGATGTACTTCATCCAGACATC

AACATTTGATCCTCCAAACTCAGCTACTGCATATTCAAAGTAGCTCCTCAAGTTGTCTGCATCCGGTTGAACTCTGGAAT

TCTCCAAGTCAATCATGCGTTGGAAAAAGTTCACTGATAGAGGTTTCAGATTTAGCATTCTTTTGTAGAACTTCCGTGCT

GCATTGTAGCCATGGTACAAACACTTCAATTCGAGGAGTTTCTCCTTTGCCCAGATGCACACATCATTTGGGTAGAACAA

TGCTTTCTCAAGG

>CK180182_bb No definition line found

AAGTACGGCATCGCCCAAGTCCCAGCCATCGTTTACTTCCGGAGGAAGTTTCCATCTATCTTCAGGGGTGACCTGATGAA

AGAAGARGAGGTYCTGGAGTGGCTGAAAAAAAATCGGTACAGGCACCCTGAACTGAGCCTGTTCATGTACGCGCTGATAG

CCATATCMACAGCGTTCATCTTGTACACGCTATTTCTCATTCTTCTGTATGAAGGCCCCCCGTGAAAAGAAAGAGTGATC

TCAGTGCAAGCCCGGTGTGAGRCCGAATCTYGCGTCGCCCCGAGAGAGAGTTTATGGTTGCGAACGAGAAGCATTTGGGG

AGAAGCTGTCGACACAAGCCAGCTCGGTTGAAATAAGGGCTCCGGATTCCCTAAACGCGGTTTCCTTCTGCCCGTTCCTT

ACTTCGTATACAGCGCAATGAAAAGAACCACGTTCCCATGATCGTATTTAACTATCCGCCGGAAGTACGGATGTGGTTGT

CGCCCAGCCGGTGGGCGTTATACAGCGTCCTTAATGAAAGCAATGTTGTCCTCGCTGTTTAAAACTGCAGCACGCCACAA

TGTATACACTGCGGTCTGTAAACTCTCACCGAAGACAAACTCAAATCGAATATCGGAAATAGGCGTACACTTGAAACTCG

ATTAAACGAAGTGATGATCACGCTCAAAGTATTCCGTTGAATCGGGAAGTTAGTAAATTTCAGAAAAAAATGTGGTCCTC

CAAAGCCTGAAATTTCGACGTATATATAGCGACACCCAAAAGCTACCGCGAAATTATTGTATCTGCTGTCAACCAGCACC

CACACGAGCGGAAAGGGCGGCCAGCGTCCGGGCGATGCAGGCAGGGTCCCGTGA

>CK180218_bb No definition line found

GCTTCCAGTCCAGGAGAGAGGCACCACCATCTATCGCGCCGGTCACGTGATCACTGTTGAGAACGACAACGGCCTGAAGA

TCAGCTGCAACTTGGCATACGACCAGTGCGTGACGGAGGTCACCGGAGCCTACTTCGGCAAAACGGGTGGTCTTCTGGGG

AACCTACGACTACGAGGACAAACTGGACCTCATGACACCCGACAGGAAGATCGTACAAGACACAACGGCGTTCGCCAGTG

AATGGGAAGTGGGCCAGGGAAGATGCCGGAACCAAAGAAATCTCGCCCAGTCAGCGGTCACCGAGCCGAAAGCGACCGCC

TTTTGTTCCAAGTACTTCGAGAAGGAAGAGTCCCCGCTACACAGTTGCTTTAAGATGGTCGACCCAAAGCCATACATGAA

GATGTGCCT

>CK180223_bb No definition line found

GCCTGAAAGAAGCAATGCCAACCAAAAATAGTGATCTGCAAGACGTAAACCTTGGCATCTGCAAACTGCCAGTGCCGTTC

TCTCCAACATTGCGGTCAAACCTTGCTGTGTCCTGCTTGGGTGTTTTAGRGAAAAGCATCTACCAATATGTGATGCCAAA

TGGCTYCAAATCAAGTACATAGTTCGCAAGTTCGTTGGCTTGCAATCGAATGTTGTAMAGCTGTGACGAGCTTAGCAAGT

TTAGCCTGGCTACCCAGCAGTAGAAATGCTGGATCAGACTGTGATGAGCTCAGCTTTGCTGAAAAAAAGCCAAAGCAATT

GTTGTAATTTTCGCTCGGTGATTGCACTCTCTGGCAGTGGCTCTACAGTGCTAAGGTGAACATTGCTTTGTTGTTGATGA

GAGGTGCAGAYGTCTCTTGGYGTTTGTCCCATGGACACGTGAGTTATGCAAATTGCTGTACATGTGGCAGCCTGTTTGAA

TGTTCATCTAGGATCTTTTGGGGTGGGGATGTTTAAATATGACTGTGKGCGAAATGTCAACTCCTGGTTCCACAAGAAGT

AACTGAATTCAGTACTTCTGAA

>CK180241_bb No definition line found

ATATCTTCGCACTTTCAATAAACCATGTCAAGTCAAGAGCTTCAAGTATGCTGTGTTTTTTCTACCCCATTATTGGTCTA

AAACTGCAGCATGAGGTAGACGAGGCTCTATGCACTTGCACTAAGAGTACGTCATCGCACCATTTGGGCATGATAAACGG

CGAGAGGTACCGGCGTGCGGACAGAAAAATTGAGAGGTATTGGTGACTTAAGYGGCAGACACCCTCTCACGGGCCCTTTC

GGTATTCCASGTGTTCCAATGTTCGGAAGCAWCTTRTAAAGTYGGTAGTTTACTGCTGGCTGAGTGAAGCCTTTATTAAC

AAAATGAGCTCRTGCATAAGTTCAAGTTGTCTTGTGGCACTTGCTCAACGTTGTTGCTAATAGATGCTGCCGACTTTTCC

TTCTGGGAATGTGCTCATCTACAATGCATCATTCATCTCAAAAACTAGCACATAGGCGACTTGGATTGTTATTCGCTAGT

GGTTGATTAATGTCCGAGACCTGCCAAGTGAAAGATTATGGCATAATCAACGCCTATTGTTTCACTAGATGGTACAGAAT

TGGAAACTATTTTACACATATAAGTAGTTTTAATTGTCGATTTCTGTTGCATGTCATACCTTTCGTTGCACAGCATGAAA

TTGCYATGTTTTACTTCTACTGGTGGCCTATGCACGTCATGTTTTATTGTTCAAGTCTCGCCTTTCGTGCTGCCTTTGTA

GGTGTGCTAATACTTGTGATCCACTGGCATGTGGATAGAACTTTTCAGGAGAGAGAGAGAGTTGTCGCAATAGTTTCAAT

AGGCCCAATGACATCTTTTTACCGCATGCCATTGTTATATGCGAGAGTTCAAAAGGTCTCGATAAGACCTGCATGGTTCT

GATGATGAAATGAAAATAAATTATGTGCTTTAAATGAATACCGGGTATCATTGCACCGTACAGAGCTTACTCCTACTGCC

AGTGGTTTCTAGCCTAAGAGTGTAGCTGCATGTTGCCTTCTTTTATCAATGTTATGTACTGATGGTATTGCTTTGCTAAT

TTAAAAAGCCTRCTTTAGTCTTCCACCAATGGCGATTTCGGTTGACAAGGTGATTATACCAAAGTGTCGTTGCGACCATT

TCCC

>CK180268_bb No definition line found

GAAGCACTTTGCAAGAAAATTTATTCTTTCGGAATTCAGGGCAAAGCTCCAAACATTCAATATGGTAAAAATACTCCTTG

CTGTACATTGCATGTGACTGAGCAACAATGTATCGGTGACTAGAGAAACTTCTGATACGTCACATCATGCAAAGTTAAGC

CGCATGTCAAATTGTGCCACACCTATTCACAACAACACATTGTCACACAAAAAGGTCACATACAAAATAACGCACTGCAC

CGAAGTAGCAGTGACCTTGTCATAGCCTAAGGAACAACAGATTTCCACACGAGCTGCATCAGGCTTGAGTAAGGTGTCGA

CTTCTTTGGAAGCACAAGCTAGGCACGCACTGCCAGAACTGCCTTTAAATCATAAACAAGACAGGCATATAAATAGAAAT

GATGAGCAAGCAGATGGCGTCAACATATGCATCACACATAGCTCAGCTACCATAGATGACGTCCTGCAATCTACAATGCA

GATTATTTCCTACATGACACATCGATTCTGTGAAAAAGATGTTAACACAAGTCTGCAAGGTGTCAGTGCGGTCAAGAACT

CAACGGACACTAAGCATCAAGATACAGCCGTCCGTTCAGCATGCGAATTTCCCAAGGACACGATGATTTTGGTAAATTTG

TTCTAAACACTTTATTGCTTATGTCASCATAATGCATTGTTGGCAAAGTTGGTGCAATGCATTAAGGGAAATATTGAGTC

AGAAACTGACGTCAGTCTCGACTGATTCTATTCCAAGCACTCATTTGACTTTATGTGCACATGTCATGTCGATGTTGCGA

TTATTTGCAATACTCAAGCTTGATGCCTCACACATGTGCCACATAGCTTGCTAGGTGAATATGCAAATGTTACTGTAATT

TAAACAACTGACAATACGGCGAACACT

>CK180283_bb No definition line found

ATTAATTGCAAAAGCTTTAAATGCCTCATCAAACGCAAAAGTTCATCGCCAGCACCAGGGTAAACACGAGTGGCGAAAAG

ATCTGGACAATTTGTGACGTCATCGTGTTGTCAAATGACGGCATCGTGACATGACATCGTTGCTTGGTCAAATGGACTGA

ACATGGAGGCAGTGAAGAAACACTTTAGGTGTAGAAAGCTTTCGGTGGGAGGCCGGGGAGTATCGATACAGGTGGTTTTT

GCCTATCTATGAAGAACGCATAAGAGACCCTGTGAGTTATTTGCATTCACATACTGCATTGTTAGGTTGTCACTGAATTG

TACGTACGAAGCGGTTCCTTTGAAGCTATCGGAACTTTTGTATCACTAAGAGCTCATTATACAGGGAGCTATGTGTACAA

TCTTGTCATTCAGTTATTTCAATGAGTTTCATTCATGTGCTGGATTAATTGGCATAACACAGATTTATTTTTCAACACCT

TTCGTTCATCAGCTAGTAAGCACTTAGAATGTAGATTCAAGTCTTGCAGTGCGCTTCCGTTGACCACAAGGTTTTGCACA

CCATTGCATACAGATAGGTAATGCATTTAAACCGTAATATAACAAACCAGGATATAACAAAATATTGGTTATAACAATAT

AAATGAAGAATAGTCTTGAAATAGATATGGTGCTAGGTATATACCTTTACAGAAAATTTTCAGATACAATGAACTTGTTT

TCATGTAAGATGCAACTTCGCTTTTAATGAGATTGCATAGTTTTTTTTCCATTTAATACACCTTGCAACCTTTATGAAAG

CATTCCTGGGGTTTTAAAGCATGTATTGCATATTATTGTTTCAGAATGCTCTCATTTGCAAAAGTCAACAGTGTGCAATC

A

>CK180355_bb No definition line found

TGAAATGGGACAGAAAAATGCAATATTCTTTTATTAACAAATCATGCACAAAGTTCAATGCACTTGAATGTGAGGGTTCC

AATAGGATTCTTGTGGTGTTCCTCACAAAGATTCATATATTCTCTTTCATTCACATGACTAGAGCAATTACCTGAGCGTC

TTTTTCAAGTACTAGCTTAATCGAAGTGTCCCTTCTCTGCTCATTTTCTAAAGTTGCGGCGGACCAAAATGCTACAATAG

GTCGGCAATATATCTAGATCCAATAATGAAGCCCATTATGAGTGAATCACACTGCTGCACAAAAGTATTATAATAGTTCA

TATATCAATCGGACAGGTGCACACTGCCAGCAAGCGGGAGACTAGAAAGTGAAGCTCCTCACATCAAAAAAACTCGTACG

ACACAAAGAACGCGTAAGCGTGCAATGTATCACACAATGCAAATGACTCGCAAATCATGTCAAGCACACGACAGGCATCA

CAGATAATCTTTAACACAACCAAGACAAGAACGCTACATTTACATTCAAACATTTGTGTCCTGGCATCCTGGTTCATGGC

AGTTCGTAAAGTGGCATGCAGAAGCACAGGAGCAACATGTGGCAATGACCGCATTCACTACCAGTAAGAGTAATAACAGT

ACATCCAGGAAGCCATGTCAGTGTAACAGACTACTCGTTACACCGCAATGAAAAAGTACTGGACAGGTACTGTGTAAAAA

AACAGGAACTTATAGGGCAGGCACAAGTTGCAACCTCTTAATTTATCACATTCCAAATAAAT

>CK180362_bb No definition line found

GATACCACGTACACATCACCAAGCACGCCACCTTTCTTGTATAGTGTCAATGGGCACCACAAAGACACTGATTGAAAGAC

TGTTCGGAGAGCCGGATTCACGACTGCGTCAATGGCGATGAACAATGAGGCAGTAGGCGAGAGGAAGGTCGAGCATTGTG

TTCCCTTGTCTCTGGCGTGTCGGATGTTCCTGTATGCCCTCCAGGGTTACTTCATCGAGGTCACGTTCACAGCCATCTGT

AACCTAGTAGAAACTGGAAGCCTCGAGCTTCGTGGATGCAGCAGCATCTGGAGCCTGATGATTTACAGCATCGCCACCAT

CAGCATGGAGAGAATCAACGCTTTTCTCAAACCACTAGGCTGGCCRCTTGCGGCCATAGCGTTTGTTCACATGTGCTGCA

TGTACGTGTGTGAGTTCACAAGTGGCTGCCTTCTCAGGACGATCGGTGCATGCTCCTGGGACTATGGGGACTTTATGTTC

AACGTCGCTGGTTTAGTGACGCTAGAGTATGCACCATTATGGTACCTCCTTGGAGTTTATTTTTAGAAAAAGTCTATGCT

CCAAACATGGGCAGGTTGGCGTGGGTTTACCGCGCAAATCCTGCTCTAAAGCTTCAGCTGCCACCCTGAAGAAAAGAAGT

GTCTCTTCGAAAGAAGCCTATCGGTATAAGGAAGACACCTCCATTTGCCTTACCTAGTTCATTGATTTTTTCTAGCAACC

TGCAGCAGCGAAGGATGGGAATCATTGGTCAGACAGAATTTCTAACATAAAAAATAAGGGTTTGACTGAGATAAAACCAT

AAAAGTCATGACATGCGATATCAGATTATTGCTAAGCGTAACGTGAAATAAGGAAGTAGACATGCCCATTACATGCAGCT

TCGACCACTGTGAACGTAAGGGCTTCGATATCGCGAGTGCATAATGGGCACGCTCGTTTCTTGCT

>CK180392_bb No definition line found

AGCATGCAGACATGTCTAGCATACTGCAGGCGTACTCAGCTAATTTTATGGAATGCTTGACATATTCTTGGGTCATATCA

TTGCGCTTTTATGTACTGAATTTATTTTTCAGTACTGAAGGAATGCTATTCGAGTACATGAAAGCAACACTGTGAGACCA

CTGCTTCTTTAGTTAGCGATTTCCTAGAATCATGTAAACAAAACACTAAAACAGACACACATAACTCGCAAAAGTTGTCT

GCGCTTTCGACTTCGTGGCATGACTGTTAGGCAACTTCTTTTGTTGTTTTGGATTTACTGAAAAACATGTTAGATCATCT

CTATGCCAACCTTAGGTTTCCTGTAGCGTACACTTCATCGCAAACCACTTGAGATATTTTTATTGACAAAAGTACAAGGT

TACTTGTGTTTAATACAGCCATAAATCAGCCAGATGTGAAGTTGCTTTTCGTATAATAGTGAATGAGCCTRYTGAAATTG

TACATTGACTGAAATCAATAGGACTGTTGTGTTGWYGACACTTGKGGTGMTTTTTGTGTGATGCTTAATCTAAGTKCACT

GCTGGGAGTGCCGCTGGGGTTGTCTTTYACTTACTTAGTTATTAATGAGCGTTGTTCTTTTTCCCGACATAATTCTTCTA

TAGCAGTGTGAACAATTGTATTTGCTCTGTGCAGAAATATTTGGACGAAGCTGAGAAAGACCGGGAGCGTTACACGAAGG

AGCTGGAGCAGTACCAGCAGACAGAGGCATACAGAATGTTCACAAAGAAGCAGCATGAAGAAAAAAGCAAAAGGTATTTC

TAGCCATACTCGTTCCCTCTCACAATGCACRTGTGGATGTGCRACAAAYTCTTTACAGTCATTAATTTTTTTTTGTAAAG

TGGGGTGAAGCCAAGAAAAATGTGGCAGACACGCCCTATCTCATTCAGCAGTTCAATG

>CK180405_bb No definition line found

TCACAATGATGTAAAGTTGGGTCAATTCACTTTGTTCAATGTAGCTGTGCGCATGAAGACAAATTTCTAAATAAAGCGAC

TCAGTGGGGGCGCTGTCACTACCTGCAGCTTTGCTTATACTTGAATTATCTGAAAAATTGTGGCATCGCATTCCTGAGTC

TATTCTATTATTACGTAGAAAATTACTTCAGGGCCTCAGTAGAGCTATATGAGCAACCAAATAAATGAGCAGTGTTTCCC

AGGCTGTGTTCAACTGGGGAATTCATTGAAATGGCAGCCATAGCCAGCTGCCTTCAGGCATGCCATATGCAAATATGTAG

TCCACACTGTGGATTCYTGCAGGAATTCTTGGCAAGCAAGCTATTATATGCGCTGAACTGCAGGCCTGTTCTCTTTAAAG

TAGAATATGTAGTTCGAAAACTGGGGATCAACATAGATTCTTCAGTGTGATGTTGCTGCTAGGGTCGGACTAGAYGTCTT

GAACACTGTATTGAAGTGTAACCTTCGAATCGCGGYAGCATTCTGCAGAGTCTGGGAGGCAAAGCTTGCAGCTGAAAAAA

GGTGTGTGCAGTCTGCTTTACATGCAGTGCCTCATGTGCAGTACAGGTGCCTTTTTTATATGTATGAATAGGTCACACAT

GTTGCCAGGGATTACAACATGAATCATTGTTTACATTCCTAAAAGGATAATGAAATGTGTATTGATATTACAGCCAAATG

TTTTTTTAATTACGTTTACATTTCAGGAAATTTTTTATCATTCTATACCTCTCTAAACTCTAGCAGACAGCCAATTTTGA

GTAAATACATATGAGCATAGGGTGGTTCACCCATTTTGTGGTACCGTGTGAAATTCTGTACATAAATCAAAATCTATAAA

AAGGAGCTTTGGGTAGAAAGCGAGAATTTTTTAGACAATGCCA

>CK180454_bb No definition line found

CTTTGTAAGCAGTCCTGGAACGATAACTGAAATCATTGTAACTTAATCTACCCTTTGAGACGTGCCTAGGCGTATACTCA

GTTGGCATCGTGCAGGGGACGCAACAAATCGGACCTTTAGTTTCTTGAAGTATACATATTCGCTTCACCATGGCTTCTGC

AAGCAAGCAAACAGTGTTCGGCTACGGCACAAACCTAGACTGGCGCCCTACGCAGTTCGTGAATGTTTTCTCCTGGAAGA

GGGTGTGCAGCATCTGCGGACTCGTTCCTTCAACGGTGGCCATGTTACCCTGCTGCTTCTGCGTTCTCTGCCTACCCTGC

TACCACCGCGACGCCTTCGATTCCAACTGTTGTCCCCTAGACGGAGAATTATTCCAAGTGAAAAACGTAGTGTGGTCCAC

TTTCGCCAAGGAAACCGTCCTTAATCGCAGGATTCGCTGCTGGAACGCAGATAACGGTTGTGAAAGCGAGGGTGTCGCTT

CGGCGATGTTGGAGCACTTCGCCAATGACTGCCAATTCCACGCCGTCAGCTGCTCCAGCTGCCGGGGAAAGTTCTTGCAC

CAGGACATCGCGAATCACGTTGTGTCCGTCTGCACTGCGCGTTCTCCCGTGAAGCGAAATTCGAGACATCGCCCTCAAGG

CAACGAGTCATCAGATGTGATGGCAGAGTTAAAGAAGATCTCGAGCGAGAATGCCCTCATGAAGAAGCAGCTCGAATCAT

TGGAACGTCGGATGATCAGCGACAGCGATAGCGCCCGAAAGGTCTCGTCTTCCGTAGTCGACAAAACTGTGGCAACTGCC

TGTGAAAACGGCACCAGTTCAACGGGCAGATCCTGCAGCGAGAGCGAATTCGTGCGTGAAGTCTTGTCGCAGATCCGCCA

TTTCCTGGCTGAAAACGAGCGTGGAACGAAAGCCTTTATCACGCAGGAATGCAACGGCATCGTGGAATCACTAACCGAAA

AGCTGAAATAAGCTTCTTTCATTGG

>CK180538_bb No definition line found

TCTAACCCTGTGTAATCGCATCCACTACATCCCAACATCAAAGATAGTGTGATGTAATGAATTATTGAACTGAATTCAAG

TTAAAAGATGCATGGAGCTTGATATACATGAATACCGGTGCCGCAATGTATGTAACCACCTGTGGATAAACGAGCGACTT

GAGTGGGTTTAATGCCGTCACCTTGTTGCTCTGTTGTGGTTGATCGGCTGACAGTTATCGAAGACCATTTTTTTCTAAAA

GTTGTTTGCCTGTTAGCCTGTTTTAGCTGACAGCTAGAATTTTCAGTATGGTGACTCATCACGACCACTGTTTGAGCCTA

ATTACTTGCACTACATATATTTGCATATTTGCAGCTATTGTGCAGCTTTTTAAATTTTATTTTTACGGCACAAACTGTGA

TATGCTTGCAGGCCAACGGGAACAAAAGATATTATAATGTAGCTGTACCTTCATCAAGTCTCCTCTTCTATACTGGACTC

ACAGCATGGAAGGGGAGACTTGATGGAATAGTATTTGTAATAGCATTTATAGGAATAGCTTTTGCAATCACATCAATTTG

CTTAATGATGCATTTCTTTACCGCTGTGTGGCCGTCATTCTGCGTGACAGATATTGAGGTCTCCCCATGCACGCTGAGCT

TCCTGCTTGTAGCAGTTCTTGTTTCCTAAATTATAACTGTGAAGCTTAGGAGAGCATTAGCTTGATGAAAGTTGAGTAAG

AGAGTCAYCTTCGCACTGCAGTGGAAAGTGGCCTTTAATTGAAATGTGCCATATGGAGCCCCCTTCTGCTCCAAATATCG

AGATGATCTCACCCTTATTTCACCTTCTCTTTTTTATTTTCAAGCTTTTCGTTCGTGGTGAGGTGAGCGAAACATTTCCA

TAGCTCCCATTCCGTGATGTTTCTTTTAAAAATGATGCCAC

>CK180542_bb No definition line found

CCCACAAACATTGGCTACTTTTCTTACGGAGTACAACAATCAAGAAAAGCGGCGGCTATTTGACCTACAGTGGTTGACGT

GCCAAGTGTGCCTGACGTCCAAACAGGGCTGCGACTTTGAGCTGATGATTGGCTGCGACCACCCATTCTGCCGAGACTGC

CTCCGCGAGCACTTTCGCATACAAATTGAGAGTGGCTGTGCAAGTCAGCTCCGTTGTCCACAGGAGAAGTGCACAACTCA

GGTGTTGCCCACGCAGGTGAAAGCACTGGTTGGTGATGCTCTTGGTTCACGCTATGAGGAGAGCCTGCTCAGTGCCTACC

TGGACTCCCAGGAGGACCTGACCTACTGCCCCCGACTACAGTGCCAGCGGCCGGTGGTGTTGGACCCAGGTCTATCCATG

GCACAGTGTGCCAACTGTTACTTTGTTTTCTGCCTCTACTGCCGTATGGTCTATCACGGGGTCCAGCCATGCCGGCTAAA

ACCTGGTGAGCAGCGGGCTATCCGAGATGAATACCTGTCAGCAACTCCAGCGGGGAAACAAGCAATGGAGAAGCGATATG

GGAAACGCACACTGCAACTCCTGGTGGATGAATCACTCACCCAGGACTGGATGCAGGAGAACAGCAAGAAGTGCCCACAC

TGCAGCATTTCTATTSAGAAGCARGACGGATGCAAYAAGATGACCTGYTGGCGCTGCGGAACATACTTCTGCTGGATCTG

TATGAAGGCCCTCAGRTCATCCGGCAACCCATACCAGCATTTCAGTGACCCCAGTTCAGGCTGCTTCAACAAGCTGTTTG

AAGGAGCAGAGGAGATTGTTGAAGAGGATGACCATGAGTTTGGGGTAGTTTTTGTGAATGTTTGCTACGTCTGCTGTTCC

TGTTAAAGACAGTGTAAATTTATTTTCTTTTACTTTACTTTTAACTTTTTTTTTACTTAGAGAGAAGCATTTACTGCATG

TCCACATGACTGGTATAATGAATCAATTTATTTCCACTT

>CK180603_bb No definition line found

GGAACGGTGTAGGTCGAAGTGAATTAATTCGAGCAAAATGTTTGCTGTGCTTGCCCGTTCCGGTGTGACCGCGCTACGAG

CCGCTGCCATCCCCAAAAATGCAGTTCTGCCAACTGCAATTTCCTCTGCGAGGCATATGTCCAAGCACAACCAGGAGACA

GAGGAGGAGTTTGACGCTCGCTACGAAGCGTACTTCAACCGCAAGGACATTGACGGCTGGGAGATCCGCAAGGCGATGAA

CGACCTCCAGGGATTCGACGTTGTGCCCGAACCCAAAATCGTCATCGCCGCGCTAAAAGCCTGCCGACGGACTAACGACT

TCGCCCTGGCTGTTCGATTCCTCGAGGCCATCAAGTACAAGTGTGGCAGCAAGGTGAAGGAGATCTACCCATATATTCTT

CAAGAGATTCGCCCGACACTCACTGAACTGGGCATCAACACCCCTGAAGAGCTGGGGTACGACAAGCCTGAGTACTACGT

GCCTCTGGACTACGAAACGTAGAGGTTTCGACTGCATGCTGGAAGGATGTACCTGCCATGGCTTAGTATGCAAGGTTGTT

TGTTCCCGCTGCCTAGGCTTAAAATAAATGAAGCACTGGTGTTCTCTCCGCTCCAAAAAAAAAAAAAAAAAAAAAAAAAT

GAATACACGGCTCCTATTGGCCTCGGGGTATTCCACACGGGTGTAGAAATTTACGGAACAGAATACGCGTACGGAGGTCA

CCCTTTCCCGTTCTCGGGAATCTTCGAAATCCCGCCAAAATTTGCCACCGACCTCGGAGACCAGTTCAAGTACAAGCAGA

GCATTCTGGTGGGCCACACGGACTTCAACCAGGTAGACGTGCGAAAGATTGTCGAGGAGCTGGGAAACGAGTACCGTGGG

GACCGATACCACCTGATGAACAAGAACTGCAACCACTTCTCGGGCGCCCTTACCAAGGTGACATGATTTTCTTTTCCTCT

TGGTGCACATCAGGATATGTAGTCATCTACAAATTGAATGGTAGCGAGACATTACAAAGTAGAA

>CK180709_bb No definition line found

AATGCATACATTCATGTCGTATTTTATGTTGATACAAGCTTTTGACATTATTTTTTTTAGGAAAAAATGAACCATTGTTA

CGATAAAGACATCTCAAGTGCGCTGACGATGAGGCTGTTTTTTTGTTTATTTKTACAGATGGAAGAGCTCGGAACACCGA

AATCAAAAGTCCMAAGCTACGGAGCTCTTATGGAGATAGTGGAAGCGGCRAGGAATTCCCCTGGCTTGTGAARGTCGTGA

CATCRAAAGCACCAGTGCCTTTGCGCCGGAGTAGCTGTGGGAATATCTAACCAGCGGGCAACGATTACAATCGAGGATGT

CTTGCTAGAAAGGCGTTGTAAATACTGATCAACGGGGCTGTACATACATCTTTGTAACATAGTAATATACTGTGGCTGCT

GAGCAGCGTCTCTTTTTGCATCACATTGCATCGGGGTGTGTCGACGGACACTGTTGGCCTATGCGCGCAAAAAATAGTCA

GTTGGAAAGCTGTTTGACTAGCATTTGGCACCTTTCACTGACTTTGTACAGCTTACTCCTCAAATAGATACAGCTCAAAT

AGATGACTATTTCAGTTCATTTAAATTATAGAACTCGTTGAGCGGCTAGTAGATGACACATTCTCACTCAAAAACACTGT

AGTGCCACACTAGACGAAAAGACTGAGAAGTACGTGACAGCACCCGTCCTATTCTGTACTTTTCAGTCTTGTCTCTGTCC

AATATGGCGCTACAACACTTTAAAGTAAGAATGTGCCAACTTAATTAGCACTGAACAGGTTTCTTGCCCTTTTTGTCCAG

AAATAAGTGGAACTACAACATGGCCCTAATTTAAAGATAGGGTCAGATGGACATTTTTGGGTTGACGATTC

>CK180904_bb No definition line found

CGACAACAGCACGAACAGCAGTGTCGACCACTGCAGCACACCGTCCCCCGTGGCAGCGTATGACCCCGTCCTTGCTGCTG

CGAACGTGCGTGAGCGTGTGCGGATGAGTCGGTACTGGGTGCCCACCGATTTGGGCCAGGTGCACGGCGGAAGCGGTGGC

TCTGGCGGTGGCCTGGTGTTCACGGTGATGTCGTACAATGTTCTGGCCCAAGGGCTCCTGGAGGACAATCCCTACCTGTA

CCAGCACTGCCACGAGGATGTGCTCCAGTGGACCCTGCGCCGAAAGAACCTCCTCGCCGAGCTCAAGGAGATCAGTGCTG

ACATTCTCTGCCTCCAAGAACTCCAGCAAGACCATTACGAGACGGACTTCAAGCCCGAGCTGGASWMAWTCRGGTTATGG

CTGCCTATACAAACAGCGCACTGGTGACAAGCGAGATGGCTGTGGCATTTTCTTTCGCAAGTCTGTTTTCGAGCTGGACT

GCTTCGAGCCGATCGAGTACGCCCGCTCCGACGTTACCGTCCTCGACCGTGACAACGTTGCTCTCATTGCCATGTTGAAG

CCGGTTGCTGGCAATGCCAAGTTTGGTGCAGATTTCCGGCTGTGTGTGTCGACGACTCACCTGCTGTTCAACCCACGCCG

CGGAGACATCAAGCTCGCCCAGCTGTGCCTCCTGCTCGCCGAGATCGACCGGCTGGCATTTCGCGGAGACTCGCCGGATG

GCACGCCGTTGTACGTTCCCATCCTCTTGTGCGGAGACATGAACAGCGAGCCGCACTCACCCCTCTACACTTTTCTCACC

CGTGGAAGCCTGTGCTATGAGGGGCTTCTCTCAGGCGACGTTT

>CK181093_bb No definition line found

TTCAGCACAGGCTTTTATGAAGAACCTCTGGACACCTTAATTATGAGACTGCTCTCACTTATGCCCCATTTCAGCAGTGC

AGGCAGGACATTTCTAATGTTTCATGCATGGCTTTTTGTACTTGAAAGGATAGCATGTTAATAATGCCTGAGCACATAAA

ATGAACTGTTTACACTTACTGGCCACATACAAATCTAGGCTGTCGAAAGATGAAATTTATTTGCAAACAGAATGTTGATG

CAGTGCTACCCTTTTGAACATCATTGCTGGTGCAAAAAAGCAAAATCATTTCAGTCTTCTGCACTCAATATATTGCTTCC

CTCCATGCGTACTCGTGCTTGTTTTGATGGAATTGTGTTGGTGACGTGCTTTGTAAAGTGTGAGTTATTGAGCAAAGTGT

AAAATTTTGAGTAATGATTAAGTGTTTCATATGTATTAGTGCACTTCTAGTAAAATACAAGGTGGTGAGCACCTTCAGGA

CATTTCATTTTTCTAGAAACATTGCATAGGCATTGTAGAGGCCTGCTTTAGGAATTGTAGTGACAATTTAGGCAGTGTAT

TGAACTCTGGTTCACAGCTCCGAGGCAGAGCCTTTAAATAAAGCTAGCTTTCTGTGGCTTGAAAAAAAAAAGTTAGGATA

ACAGGCACCACTTTCTGCTCTTTTTTTTTTCCATCAGTATTGTTTTATACTCGGTAGAAGTGCAGCAGTAGATTATGCAT

GCTTCTGGTATTTCTGCATATGTTATCTGTCATGTTGCCCCCCCTGTAGAAATTCAGGCACCTACTTGTTTGCCCACTGT

ATCTTGATGAAACGGTGCATTACTGACCTCTATAGTTTTTCCAGTACAGAATTTTCGCACTTCTTCTAATGAACACAATA

TAAATTCACTTTTCATTTAGCTATGCTAACATTAATAAACTTTTTTGTTCCTACGTGAAATTTTTTGGTATATA

>CK181108_bb No definition line found

TACTAAGGAGGCTAAATTTCCCTTTGTGCAACTGTAATCACTCTCCCCTTTAGAAATTTTGTTTGGGTGGAGCCATTCAG

GGCCCAAACGTATTCGTTGGTGCCCCCCGA

>CK181147_bb No definition line found

CCGACTCCGAAAAGATCTTGAAAGCGGTTGCCGAGAAGCTGCGCAATTGCATCGAGCAAGTGTTGCGGGCGTCGCAGGAG

AAGCCCCGCCGCTTCGTCGAGACAGTGGAGCTGCAGATTAAGCTGCGGAATTACAACGCCAGAAAGGACGAATGCCTTCG

GGGAGACATCAGGCTGCCCCACATACCGCGCAAGAAGTTGGCGGTGTGCGTCATCGGAAATAAAGCTCAATGTGATGAGG

CCAAAAGCCACCAACTGAGTGCCGTTCCCGATGAAATTTTAAAAGGGAAGACGAAAAAAGGACAAACAATGAAAAAAATT

GTTGAGAAATATCGTGTCTTCATGGCGACTGAGCCTGTGATAAAGAAGATTCCACGTAATATTGCCAATGACATGGTCAA

GAAGGGCAAGTTACCCGTGGTTCTTGGCGAAGAGGAGCCAATGCAGGCCAAGCTGTGAWSAWGTCCGGACCACAGTCACT

TTCAGAGTGAAGGCACCATTGATTGGTGTTCCTGTTGGGAACGTCTCAATGACCCCAGAGGAGCTGGGCGAGAACATCAA

CATTGTTATCAAACACATATTGGCTACCTTGGAGCAGCACAAGCTGGTCGTTCGTTCCATGCATACCAAGTCTACCATGG

GTCCCCCCCAAAAACTCTACTGAAACCTTTTCTCGCAACCAGCATATTCTTACTCCTTACCAATAAATGTTCTATATACT

>CK181169_bb No definition line found

TATCTGAAAAGTGTGGATCTAAAACTAATAATCAGATGAGCGCGTCAGACTTGAAGATCTGGCTGATTTTTGGGAAGCTC

CCATCTTCGCAGTTCATTTGCGGAGGTGCATGTTAGCTTACAGAAGTTGAAAGGGCGGCAGGAGTGGTGGYGYGGTAGTC

AYCTGCTAKCCCTCGTGTTGTGTAGGTGRAAGCCTGCAGAATCAGATAYCTGAGCTTCATCGCGGAAGGTCGAAGCTGAC

GGGAGGCCTATTTCAGAAAGTGCTGACTGCAGCTCTTGCATCGTAAGGTCAACTACCGATAAACATTGTACTGATATATG

GAGAGTGGTTTAACTACATACGAATTGAATATGAGACGGCCCTTTGGTATAGGAGTAGARATTTATAATAAGYGCTTTAA

AGCTATAAGCTTTACTACTGCTTTCAGGTATTGCTTGACTGGGCTTGCTATTATTCCATTAAAACTTATCAAGAGCCTCG

GGTGAGAAGATTCTCAGAAAAGAAATGAACACCTCATTTGATCTTTCTGTTTCTACTTACAATAAAATATCAGCTTTGAA

ACCTCTRAAAGCCACTTCTCAATGTTCTTTTGTTGAATAGCATGAAGATAGCTCTTGTGCAGCTTTCACTCAGTCTACCT

GCTTTCACATCTTGGGAAAGAGATGACAAAGCCAATCTTAACGTTTCTTATGGTATTTTTAGGTGTGAACCTAGGTAAGT

GCTGTTGAAAGCTACTTCAAAGGCTCTTCGAGTTGCTATCTCTTTATTTGCTTTATCTGGTCTCTTTTCCATGCAGTCTA

CTCTAGCGATATATTTGGCTACTTGTGGGTAAATTGTATATTTGCTTTCCAAGATCTTCAGGGATGTATGTACACTATAA

ATACGTGCGAGATTTTTTCCCTTTGTTATTTTTAGTGACTCGTGTGTGACCTTTTCATTCGCTCATAATAATATAGCCTG

CCTTCATATTCAAGTCATAATCAATTTATAACAGATTCTCTTTTTAGTG

>CK181183_bb No definition line found

ATCACGGAGAAAGGAAACTTCCTCCGTGGATGGTATTCTAGTCACCTTAGCGATGGTTCCGAGTGTAGAACTGGTCGAAC

TCTAGAACTTCTAGTACACTCTAGCGATGGTTTGTTTGTTTCCTGTTGACTGTGTTWTAATTTGCAAAGCCGGAGAAACT

ACTGACAGTTYGGCATGCGATGAACATGCTGGCCATYTTGTTCTTGGGCGTAGCGTCTGAGCTTAGTCGCGAAAAATGAA

GGGAATGACACTCTCGCTTCTCTCTGGTTTCCTCGCGGCGCTCGCTTCCCTATGCGGGAAGTTTTCAATGGCTGGTGGAG

AAACTTCAAGCGTTTGTCAAGTTGTCTTCTCGCATTGGTTGTCTGGTCCGTCAGCTCACTACATCTGCGAAAATCTTATC

ACGGTGATACGAGTTGTATTTTTCTTCCTAATGATCGCCTGCAATGCTGTCATGTGGACAGTATTTACAAAGGCATTGCG

ACTTTGCACAACCACCTTGGAGGCTGCAGTGACCAACACGGCGTCGAACTTTTTCTTCACCGCTGTTTTCGGCCAGACGT

TGTTTGGTGAGCAGCTCACACTTCTCTGGTGGCTCGGAACTGGGATGATTCTTTTTGGCCTGCTGATGATGCACCAAGCA

AAATGTGGAGACCGAAGCTAAGCACCCAGTGCAAGGGCTTGTTCGCCAGAAAATCAGCTGAATGGGGGATATGTGGCACC

ACTTGTGATGCATGGACAGCTGTGTCGATAACTGATTTTGCACTGCTTTACCGTTTTTTCATCTTCAACTATTTTTTCTA

ATAAAAAATAATSATGCYWMTKTGTCWMTGCTGWYSYATGCAATGTTTTAGCTAACCTATAATTTGAAATACAGACTGGT

GACGTGAAAAGATGATAGTCAACTTTGTATCTGTTA

>CK181184_bb No definition line found

CACCAACCGCGACACGACGCTTAGTACGCGCGCACGATGCTTGCCAAGCGAGAAAACGTGCGATAGGAAAATGCAGGTGA

AGAGGCCACCTTGAGATTCTCGCACCAAACACCGAGACGTCGTAAAATTTGAAGGCACTTACTGGGTCCTATGTAGTTTC

TAATCGATGAAAAAGAAGTACATTTTCACCTGTGAGTGCCACAGACCTTGAATATAAAGTTTCAGAAAATTTAATTAAGC

CGATGTCACGAAAACACCAAAAATGAATTTTGAAATTTCTGAAGACACGCACGGATATTTCAGCACTAAATGTAAAAATC

AAACTTTGACCTTGATTTTCTACACAAATAATAAACTTATAGTGAAACATGTGGCATTAGAGTTGCCAGAGTGCAGTTTG

ACAAATAAAACCAAGTTACTGTTTCTCTTTAGTGTCCCTTTAATTGTAAACTTGAAGTTGGCAAGGGATATGGTGAGAAA

AGAAATCTATGGAGAAGGGTTGCTAGGTGAACATTTTACGCACAATAGAAGACGACTCCCAGTATATATATGCCAATGCA

GGTGTTCTGCCAAGCACCAAGATTCTTTTGCAGGTCTCATAAATTGTTTTCAAATGGCAGTTTATCTATCAGCATTATTT

GAAAAGCAACTAGTAACATTAACATTTACTGTGTCATGATTGCACTGTTTTTCCACCTTAATTTACAAGGGGGGGGGGGG

GGGGGAAGGGCAAAAATATAGTGCCCCTGCTCATGCAATTGCAGTGTTAGCTCCTGACGAATTTTGGTACAGTCAGCAAG

CGCAATATACAGCTTAGGCTGCTACCGGAAATGTTATAGTGAGTAATGGAGGGCTTTGACATGTTTTTTCTGATTGGCCC

TTGGCTTCAACACGAGAAGTGTCGCTGTTTGAGGTACAAGCCCAAGCT

>CK181281_bb No definition line found

CAATTTTGTTAATTTTATTTGACTGTAATTTCAGTCCTTTTGATTTTTTTCGTGCTTATCGTCTTGGGCCATAACCTTAA

AATAAAATGCTGATCCACCAGCATAAACCAAGGTTTTGATGTAGTGGAACAATTTAATTGATGGAAAATGAATTAAGGAA

AAAAAGCTTTACAGGAAAGGGGTAAATTGATGCCAAAGGACATGCAATCAATCTTGTTTGTTCTGAGTGTCAGTGTCTAC

ACCCTTTTCCTAACAGCAACTAAATATGTTTCACCATCTACACCAACAAGGAACGCTGCAAAATTACGGTGCCAAATAAG

CTTGGCAAAAAAGTGCAACCTCCCTTTCACCAGTTATTTTTGGTAACTACTCCCATATTCCACTATTTATTCACATTCAC

TAAATTGCACACACAATAAACCAAAGCATGAAATAGTTKCCTGACCAGTAAATGGCACGGAACTTARAATTGTTCAAAGC

TGAATGTTGAGTTGCAAATGAAATGCAAACAAATGATGGGAGGGGGGGGGGGGGGGGGATTAACTTCAACACC

>CK181340_bb No definition line found

TTCAAATAATTCGCGGGCAACGTAGCTTGGCGCACGCGCATGCATGCACGAGCCGGTAAACAATGAGCACAGCACGAGCT

GTGCTCATTGTTTTTCTTCTCTTCTTCATCTTTCTTTTTTCTTCTTTCACTTTYYTTTTTCTTCATCTGGCAAGAATATT

GGCCTAATATGCGAACCGTGGCCAGAACGCCGAGTGTGCGTTGCGGTGTGAGGTACCTGCGATCATGAAGTGGTGGTGCC

TGTTCCTGATCAAAGATGATGCTGGCAAGGATATTGTGGAAGATTATTTTTACCGTTTGAATCTTCTGATGGATGCTCCG

ACATGGGAGCCGGACTCACCGACGAGCCACTTGCTGCCGCTGATCCCAAGTCGTGAGGTCCACTTCGCGATCCTGCCATT

TCTCCTGAGCCCATGTGAAGAACTTCAGGCCTTTTGGAAGACTTTCCAGTTGGATTCCTTCCAGAGTGCTTTGCAAAGCT

GGTCAGACGACATTGCGAGGTTGAAAGACACCTACCACTTTCCATCTCAGCTGTCGCCGGCTGTACTTACCAGCGCTACA

AAACGCATCGAAGGTCTGCTCGACAATTTGCCGACCCCTGGGAGGGCAATGCTAGAGATTGTAGTGGACAGGAGTCTGTT

TAGTGATCTAGATGCCATGGACAATGGCATGCTCTATGGCGCCCTGTTCAGAGCCTATATGTGGCATCATGCTACTTTTA

GACCTCTGGGAGTTGGTTCGAGCATACAGATGGACAAATGGATGAATAGCCTGACAGTTACTGGCATGGACCTTCAATAT

GAGTGGCTTCCCGTGTGGAAAGGACGCTTCACTGATGGAAACACTACGACAGCATGCCACTTTGAGCTTCAGTGCTTTGT

TCCTGATTCTTACGACCCGAGCTTGAAGGAGTTTTTGCCACATTATGAGAAACCTGTAAGAGAATCCTGCGTTATTCTCC

GACTGTCATACTGGGATGATGTTGACATGAAATCAAAACCATCAACGTCCTCTAGCACMGATTGGCAGCATAATCTACTG

ATGGAAAAGCCATGTCAGTGTGAGTTTGCCTCTCTATTTTTTTTTTCTGTTTGATTGTTAAGATTAACTCGTATTAAATT

TGCAGCTGCTTAAGTGGGTGACCAGTGCCATCAGGAACCAAGTGGCCACTTGCACTTTGTATTAATGCCAGCTGACAA

>CK181375_bb No definition line found

CCAGATGGTCGAAATTTCCGCAGCCCACCACCACGGGGTCTCCTATTGTCATGTCATCGTAAAACTTCAATAGTTATTCT

TATATTTTTCTATACCGCCGTTTATGGCATTTTTGAAAGATGAGGTTGATTGGTTAAGCGCAAGAACTGCTACGKMAAGT

AACCCTTCAAAATACCCATTSGGTACCCAATGTGAACTTGTCAAGRGKTCACTGTATAACACGATGAAAAMGATTCAGTG

TTTCTGTCATCGAAAACCTTATTGAACGGACATCAAATAAATATGTCACTGTTGCAATAACGCTGGATATCCTACACTCT

ACGTTACAGCCGACCGCTAGCCAGTGGTACCAGTGTCGGCATAAAAATACCACAGTTTAACTTGACCAGAACTTATGGGA

CACCATTATATATTAAGATAAAGCAAGGCCCACTTGCACATTGTTTCCAATAAGGTCGGTCGTGACTCTGCATTGAGGCA

GGATCGAAACCGCGCACCACGGCGTTTCACATACGTTCGCCGGTAAAGGGCTTCTCGGTGTGCTATGACTCTCCAGTGCA

TCTGCCGTGGAGTCCTGTTGGTGACTGGGAGGAAATCGCGACTTTGTAGATATATCCCGTTGTAATAGCGGCACACCTTC

ATTGACCTGTACGTCGTCGCAGCCATCGAAAGCCAATGTTGTAGAGTACACATCTGTAGCTGCGAGTAGTAGCATTTCGG

CGAGCATACGATTTTCGAGTTGATAGGGTTGAAACAACGGAGTTTATTGGAGGTTAAAAAGCGAGAAACGGAAAGGAGAA

ACAATGCATAGTTTCTGACACAAGAGCATCGAGACACGACGTACAACTTCTGTACACTCAGCTTTTGGAAACACTAATTC

ATTGGGATTCAATAGCATAGACACGTTAGTACACGTCATGAACTCTCCATGTGTCGTGTCACTGCGTTTAACAAAGATGC

CAGTAGTTCGCTGCGAAACAAGCGCGAACTTTTCTGTTCGAGTGTAAATCCCGAATTGCTGCACTGACGTTGCCTCTGTG

ACGTCTCAGATCTTTTTGTGTTGTCACCAAATTGGATAATTCTTTA

>CK181435_bb No definition line found

TTTTGATGTTGAAGGAACTGCTGAAATATCAGCTGCTTTATTTGTCGATTCGGCAGTACCAGCAAAATTTTACCACATTC

TTGAATCCTATACAAACACAGTTTTTATTATATTAAAGAAGGAAACTGTGTACATCCAACTAAAATGAACGTTCAATGCT

ATAAAAAAATATTGTAGCATGGCTTCTATAGTGRTGGTTGAAAAAAAGAACAAGTAAAACCTGCAGTGTCAAAATTTATA

GAATGAAAATTTATGCATCSAATATGGCCATTACSRCCCCTTGTTAGCAGCTCTTCATTGCTGTGCACAATAKAAAATCG

TTTGCTTTCTGGTCAAGCACAYAATATAGTCCAYGTTTTTTAGATGACCATGCTACAATTGGTGATGGTGACCCAAACTT

AAGACTAACCAGCTTGTCATGAGTCACAAGACTCACCGAACACACAAAGCACATTTGTATAACTATGGTCGTGCAAACAA

ACAGGACACTAGAAGGCAATATGCACACGTAGCTCTACTTTGTGGTAGGCGTGAACATAGGTAAACGACAGCCTGCTTGA

GCATAAGAACGAAGTGAACTAGATACCGCCAGATGGACCCCTGTCTCTGCATTGCAGGAAATGCAAATGCAATCCACAAT

TTAAAGATATCGCCATATTAAGTAGGGGTGAGTGCGAAGTAACACCTTTTGAAGACTGAATAGATAGATAT

>CK181535_bb No definition line found

TACAGACATGTATTCGTGCGATGTGTGGTATTACGCAAACGAACACCAAGACGTGAGTAATGAGGAAGGTGACCCTCTAC

ATGTATGTACCTGTTGTGGCCCATCACAACTGGTYCTWTTGAAGCGTCATGTCCTTTAAGAACTAGTTCAGTGACAGTTC

CTTGCAGAATCTGCCAGGGACTTTGTGCAAMAACTTTTAACTAATGCAGGRAATGCTGCAAAACTAATTGAGCACCTGGC

TTGTGCTATGCGAAACAACTGGCGGTGACATACATATAACACCACGTCGCATTGTGGTGAAACAAAAAAACAAGGTTGAG

CACTTACTAAACTACAGTGAAACATTGCATTGATGTTAAGTAGGCACTTTTCAAACTATACTTGCAAAAGTGCCTAAGCA

ACTTAATGAAACATTTCATTACAATGCACATACTACTCAATAACACTTACCCAAGTGCTGGTGGACAAAAAGAATGACCT

GTCATGTAGCAATACCTCCGCTCAGTATGATGCGCATCAACGCAGTCATACTAACAAGAGCAAACACACATCACTGTAGC

CGAACGTGCACCAAGCGTCATACTCCACACTCGATGAGCATGTGTATGCATAAATAAAAGACACACGGTTGATACAAAAT

ATGTGTATTTAATCGCTACTACAAAAACAGCGAAATCGTGTAGGCATAATATAAATAACGAAGCAGTTTGTTCTTGAGGG

TTGAGGGTTATGTATATATACAAGGTCTTGATGCTGATTCGAAGATATGAAAAACAGGTGTATAAAAAAACATGAGTGGA

GACTGATGAACACATAGGGCATATGCATGCATGCACAATGTTTACACAATCTTTTTTTTGTCACATCCGGGTTTGTTTCC

AATACTACACAA

>CK181623_bb No definition line found

CTACGGTTACCAGTGCGAGTGACTTAGGCTGTCTTTCTGATGTGTTCCAGATTATGGCTCAGCTGTCTGTAAATGAATAC

ACCGAGAAGWWWYAATCTTCTTATTCTTCTTTATTTGGGCACATCTTGCARTGCACAGAGGACCAATTATGAAGTAACCG

CTTACTTTACCCTWTGACTTGTTAGCCAACAGAGAAAGCATGCAACTCTGATTTTGCAGCAACAAAAATAGTATTCGTAC

TCGTGGATGGAATGTTTAAAAGAAACAACTTCATTTTGGTTCTAATTTTATACTTGTATCAAAAGTTAAGAGCGCACTTC

GATCTTTTTTTTTTCTAAAGTAAAATTGTGCAAAGTGCAAGATGCGTGCTTGGGTTGCAACTTAAATTAAAAAAGAAATG

CACAAAGGCAGCGGAATAGAATTGGTGAATTTAAAACACAAATATTACCCAGTGACTATCAAAAGCATATTTGATATTCT

GGCTTTCAATTCTTGTGCAAAAACTAATACAGACAGGTAAATTTAATGAACCTAAAGTATCGTGTTCACAGCTTCAGCTT

AAATGAACTACGGCTTGGCATTCTTTTAATCCAGACTACTTAACAGCACTTCCTGACCAATAACTGACAAGCAAGACAAA

TGCACATATGCAGGACATCCTTGCGGGTAAGCTTAGCTTATAGTGTTTACGACGAGAAAATGAGTCTGCACAATTCTTCC

ATATGAGCCCACGTGAGTCTTTAAAACTTCGTTTCCATCAGTCAAAATTAAAGAGGTCCCGTCAGTGTGCCCGTTCTAGA

GGTATGCATAATCAAGGTCCTGAGTAAT

>CK181624_bb No definition line found

TTACGTGCACCTCGAAGCTCCCAAGGAGGTGGTCGCAGGCTACTTACCATTCCTGGAACACCACGGTGTCAAAACAAAGA

CCATATCCGTGGCGCTCCCCGACAGGTTGTTGTAGGACAACTTCCATTTTTATACCGCCTGAGTGGCACTTTTGCTGTAC

TGATCCTCTTTTATTCACCTGTTCGTCGGCTCRGAATGGTACAAATTGTTAATGGTAATCTCATAGCAATGCATTCTACC

AGAACTTACTGTGATATTCACCCCTGTGTGCCTTAGACATACCACTTTGCTTGGTCATGTCCTCTTGGATACTCCGATCA

GAACAGAGGTCAAAGAGGAATCTCATTGCCCAAGCAATGACAGTAGCCTTCAACATTTTTTTCGTATATTACTCTTTCAT

GCTGATTTCCAAGTGGCACTTCAAAGCCTTCTTTAAAGCATCCTAACACTTGAAAAGGAGAGCAGTTCTAAGCAAGTAAG

CAGACTATGCACTGAACACGAACATTTGCGTTCTAACTAATATAATTCAATATAATAGCCCCTCTCCTGCTTAGATGAAC

AAAAAAATCTGCCACTTCATACAGTAAAGCATCAGATGAAGCATAATTATTTAGAAGGTTGATTGTTTGCATTTACACTA

GTACTTTATTGCCAAATTTAATCTGTAAAGATGGCATGTACAAAGCGCTGTTACACAAAAACTGTAGTGATGTAGCCAGT

GTTCACACACAAAAAAAAATACTTG

>CK181848_bb No definition line found

GAGTGCAACAGCCATATTCATAGATTAACCTCAACTGATCTTTAACTGCCCTCATGTGGATTATAATGCAGCTAGCTTGA

TTAAAGTGGGTACCATGTTTATAAATCCTCGTTAGTTATTTCTGTGGTAAGGAGCTGCTTGGGAAGCACTTTGAAACCTT

GGCATCCTTCAGCAGATGCAGGTGCCCACATTAATCAAGACTACTTATGAAATCCCTGCAATACTGAGGCAGTGTGACCC

TAGCACTCATATCACACTAGTTATGAATTTTGTTATATTTCTGACGTTTTAAAAACTGAGAAGCAGTGATTGAATACCAA

TTGCTTCTATATGTTGACTTTCTCGCTAGACAAAGCGGTCTTATGCATCACGTGTGCAAACTTTTGAGCACAACAAAACT

CTTAAGCACACACTTCAAGTAAAGCTACTCAAGCACTGTTTTCAAACTCGGGTGTATTAAATCAAAGAAGGCATTTCAAG

CACTTCCCAGCAGGCACAAAAGGACGCTGAAGCTGATTGCATCGCCCACCAGTCTACAGTCAAATACAGAAGTGTTCGTC

GTTGTCTGTACTCCAAAGTGCAGTTTCAGATTGACTGGACTTCAAAGTGTAGTTTCTGATCGAAACACTCGCTAGTGACC

ACCTTGGTCAAGAGGAGGAGCTTTTTACGAAATCAGTCAAGCAGAGAGATGATTAAGCATGAAGAGGTGAGAGGCTAGCC

TGCCTTTTTGTTAAAGCAAGTTCCTCATATCCTATTTTTTCACCTTTYTTTTTGAGAAATATGCGAGTGCCTTAATAAAG

ACAAAATGCAATTTTTAGTCTGWGYGTGACATTAACTTTACWCCTAACTAGTATWGTGGTAGACAGGGCTACGCACGCAT

CTCATACAAGCAAGGAACTTTTTCAGATTTGTGGTGCTTTGTATACTGCATACTTAATTGTTGTTCATCGAAAT

>CK181851_bb No definition line found

GATCATGCACAAGAAAACCAGATGACCGTGGCCAACATCGCAGTGTGCATTGCACCGTCCCTTTTTCAACTGGCGGTGCC

ACGAAGCGCGAGTGCGAGTCCACGAAGGCGAGCAACTACGGTGGGCATTCCTGACCAGCGTGAACTCAACGAGAATAGAG

CTGCCCACGAGTGCCTGGCACGAATGATTGTCGACCACAAGAAACTATTTCAGATTTCGCTGGAAACACTGCAACAGTGT

CGTCTGGAACAGTATGAACCAATGACCTTAGATGAGCTGGGAAGCCTCAAGTCATACCTCGAAGGATGCCTTCATGCCCT

TGTTACTGAGGCTCGGGAGAAGAGCAAAGGCTGGGCAAATGTGCAGCACACTGACGTTGACTTGGCCTTCAAAAAGCCAG

GGGACCCTGTTACACGCAGCTTTTYTGCGCACAAATCGATCACTTTCTTTTCGGATTYATCCCACGTTTCCTTACCCCCA

TCAGRCAGACCAAAAAACAAAAGATTGTKTCTACGCGACCRGTTTTCTATTTCGTCCTGATTCAATAAAGCGTTTCTCAT

GCTGTCAACAAGCTGGCTGTGTTGAGTGGATAGCTCTGCCTTTTCCTCGCTTGATGATGCAGTAGGCAAG

>CK181856_bb No definition line found

TGAATAGGGCAAGGTGAGGCAAAACACGACATAAATTTGAATATACTGAATGCAGTTCTTTTTTTATTTCACGTGTTACC

ATGAAACGTCAATAGRRAAGCATTCCTTTGGGTACAAAATATGTGCTACATAAATGTGACCGTGCTACATCTGTTCGART

GGGTATTATAAAAAGAAATAAAAATGCRCCATTTTGCCCCAACCTGYGGSGTCAAACGAGGCAGTGTAAAAARAAAAAAT

ATTTTCACGTGTTGCAGTACAGCCACTTGAAATTCCCTCCGTGGGCCCTCACGCAGTTTTTTTGAACCCAGCTTTTACTC

ATTTAATCCAACAAAACCCAGCTCTCCTTTGCTCCAAACTCCTTGCTTATAAACACCGTCAGTCAATGTGCTAATGTTGT

CTTTTGTTACATGCTAAAACATCATTGGTTTTTTTTTCACTATTGGAAGGCATGTATGGGGTGACATGTCTTCTTTGTCT

CTTTTTGCCGATTTAATGCAAAACTGCGAGGGCATTACGGGATATTAAACTTCGTCAGCCGGGCTTTGTGAGGCAATCAA

TTTCGAAAATTCTATCGCTTGTGATTTTTGTAATCTTAGTATAARAAATRTGTCATTAAAACTCGTTTYCAACATGTGTT

TGTGAACGGCTTGTCTTGGAGATTCTTCCCAYTTTTTCATGGGCCTCTTGAKAGCATTMGTGGGAACGCCGAACAACCTA

AATGCAGCGTGAATGGAATAAGTTCTGTCCTTACCACCAATAAGTGCTTTTTCAAGTCAAACTCCTACCAAGAAGCTCTA

TATGATATTTTCTTGTACGTCCATACCATTCTAAACAAA

>CK182008_bb No definition line found

ACGATGGTAAACCTGCATTTTTMWGGCCACCTGATTCATGGAGTGCAATAGACCTTGTACTTCACTCCACGGACCTTCTG

GTATCATCGACCACGGCTCCAGACAAGATGGGCAGCGACCATTTCCCCATCTTCACCAATATCCTGGGATTTYGAACTGC

TGGTCGGCAATTCTGCAATGTAACACGCTGGGACACCTACAGAGAAGTGTTGGACAAATCCACTGGTGAGCTGTTCGCAG

ATATGTTGCAAAGCAAGCGATCAGCAACTTCATTGCTGAAACTGCCTGACCATTTTCCTGCTCCAGACTTGAAATTGAAG

AACCTCTGCGCAGCACGTAGACGAGCGGAGCGGAAAATAATGAGAACAAAAGGAAATCCGTCTGCGAAAACCGAATATAA

CAGGATCAACGCTGCAATTCGTCGCCATACAAAAAATTAAGACGAAATCAATGGGCCGCTTTCTGTGAGAGTTTATCAAC

GTTTACGCCCTTGACAAAGATATGGGCAGTAATAAATAGTCTTTCTGGGAAGATCCGAACAAACAGGCCATTCGAAGCAC

TCGCTTTGAAACAAGGGATAGATTTGCAAACCCTTGCAGAGGATTTCGCAGACGTGTACATACCTGGTAGATCAGCAGGA

GCGGCGATTCCTCTGTCACCCCAGTTTTTCCACACGATGGATACGCCATTCACCTTCCGAGAGCTGGATATGGCACTTAG

CAAATTAAGAAGACGCTGTGCCGTGGGTCCCGATTTGATCAGCAATCAAATGCTGACAAATCTACCATATGAGCGAAAAA

GGGCTCTTTTGGACATATTTAATCATGTCTGGAGCACGGGAGAGATCCCA

>CK182045_bb No definition line found

CAGCTAATCGGGACCTTTAATCCAAATGAGAAAAGGGTTGTACAAATACACTTGCATAAAGCAGCATTTTCACGCGTATG

TTTCGCTAATTCGTTCAGGCACTGCACAGCAGTCGTACAAGGAAATGCATGCACACTTCAAAGAGCTCACACTTAGTGCT

GTGCTTGGAAATGCTGGTGTGCACAGTAGGATGGAAATTTAAACATAGCCCATGCAACTTATATGCGTGGGCAATAACAA

TAGTTGCTTGCAACCCCCTGCATTAAAAGTGGGGGCATTGCAGCCCAATAGCTTATTCCCCCCCTCCTCCTTACCTCCTT

TGGTCCCAGTCGCAAAATAAACRATAAGCTCACTTGCACAAACTGCATGGTCTATTTCCAGCGAAAAATTTCAGTTCACG

TAYTTTACTGCAGTTSCAACACTATATACAARAACTGCAGCTAAATACTTTCCCAACCTGGTACCTAACACCATTGTGCA

TGCAGCTGACATCTGTGTAGCTCCATTCTTTCAAAGAATATGTATGAGTTTCTGTGTGCTGCTCCCAAAATAAAAAAAGT

TCTAAAATTGAATTTCCTTCTTCATTTTTAACTTTAACACCACCTGGCCTCTCTCAAGCATTTAACCTGATGCCAAAGGA

GGTTTTCCACCGGGTGTTCAGGAGTGCAAAAC

>CK182055_bb No definition line found

AAGCATAAAAAATGTGCACTTTTATTAGTTATTTACAGAGAGAGAAGCAAAGATGGCAGCTTTCACTCAAAGCAAATACA

TAATAAATATCGCCAGTCCATGGCGGTCACACTTCAATCGTGGCTCCAAATCCACACGAGGCTCGGAGGATCATCCGTGT

TGGCAAAAAATATATGGACGACGCGTTATGCAGGATGAATGCCTTGCTGCGCACAAAAAAACTTTTCGTCATATTGCGGC

AGCCCTCGTGTGCGACAATAACTTTCACCGCTCCGAGCTCAAAGACATTTCAGGGTGCACAATGATGCTCCTGTTGTATA

TATTATTGCTACCGGTTATTTGCAAGTGCTATGTATTCAACCTAAAAAAAAATTAAAAAGTAACATTATGATGGTGATGT

GCCCGACACACTGGCATGTGCGTACGAAAACGAAAACTGTCTGCGCACCATGCTAATGTATGCAGTGAGCTGTGAATGGT

AGTTTCACTTTGCAATGAGTTGACACATGCTCACAAAATGGACATAAAATGGATCYTCATAATTTCTCATGTTTTTGCAT

TATCTGCACGAAAATGATGAGCYTAACGGCATTTCTTTCTCTTTAGAAGTAATAGCGAAASYATACAGAACAACAATAAT

GGGAGAGTTCGGTTAGGAAACATTCGCAGACACTGTGAAAGTGTACATAGGTTTGCGATGC

>CK182198_bb No definition line found

CTAAGGTTCTTCACCAGCCGGGTCTTTTCGGGTATTCCGAGGAAGCGCACGAGAAAAGCGGTGCGCTCTTTGAGCACGTC

CCTTCTTATCCTGAACAGCCGGCCGAAGTATGTGAGCGTCTCGATTATGGAGAACTCCGGGTAGAGAGTCAGCTCCTGTG

GCATGTAGCCCACGCCGGGCCCAGGTATGTAGCTGCCCGGCTGGCCCGGCTTGTGGCCGAACACGTGAACGCTGCCTTGC

CGGGGTTTTAACCGACCCACAATGCATCGCAGGAGAGTGGTCTTGCCGCAGCCACTTGGTCCAAGCAGTCCGTATATCTT

GCCCTCGGGCAGGCTGAGGTCGATGCCCATGAGCGTCTGGACTGCGCTCTTGCCGCGGCCGTAGGAGACGCGCAGTCCTC

GGACGTCGACGACCAGCCTGGACGGGGAAGACGTGGCGTTGCCGAACGCCGGCTTGCCGTTGGACGCCAGCACCGGCATG

GCAGCACCGAGCTTGCCGTTGGCCGCTCCGGGGGGCGTCGGGAAATGTCCATCGCTGAACGGGCCGGCCGTCTTCCGGTA

CACCAGCAGCTGAACTCCACTAGCCATGAGTCGTGTGCACTCAAGCCGAGTATTCAACCAGTAGCAGACATCATGGTCGC

TGCAGTCACAATCCTTCGCGTGAAAATGTCGTTACTGAGTCTGGTTTCTAAAACATTGCA

>CK182199_bb No definition line found

AGGCCAGTAACACAAAGCGACGTGGTCAATATTTAAGCTTTGGTATGCGTAAAAAGGCATATTTTATTTCGAAAGAAGCT

ACGCATACGTGAAACGTCAGTGATCTGTTGAGGCAGTATTTGATCTGGGACTCGGATTACGGCACAAAAGTTGCGATACG

CCAGGTTAGTGTGCGCTTGCRGATGCAGAGCACTCCYTTAAGCRGAAACTTTTTCAAGAGTGTCTTGCCGGAAGCGGGAG

TGTTTGTGAATATGTCCGTAGAAAAATGTTAAATGTGCGTCGCAGCGTAACCAAGATCTCGCCCACCTTGATTCCATTTC

TCTGGAAGAACTGAAAGACATT

>CK182240_bb No definition line found

TGCATCATGAGAAGCATTACTGTGCATTACTTCCTTCTGAATGACCCTTAGAAAGGCCTAAATGTTTCATGTTTTCGTAG

ATGCCCCGTTTCGTCTCGAGATACCTCGATATTTTTTGGGACTGTTGCAATACATGAAAACGTAAGCCATGGGTTTGTTA

TGGGCTGTCTACACAGCACACAAGCATCTTCTCCTGAAAGCTTTCGCTCTTGTGATTCTTACCACAACACTTTCCATATC

TGCAAATAGAATGGACAGGTGCATTAATTGGAAAGGCTGTGGCCTATCGGAGCCCGTTGGTAGGGTGTATAACGGAAAAC

CAATCACCAGGGAGGAAGTTCCATGGCTCGTGTTTATTTATGTGCATAGTAATACGTCCAAAGGAAACGCATGCGGTGGA

AGCATAATTACAAGAAACGTCATTTTGACAGCGGCGCATTGTTTGAAAACGGACCCTAGGCCTAAAAAAGTGTCCGTGTG

GTTCAACTCCACAACGTTTCGAGGCAACGTTCTGCAAGCGACGAAGATGGTGATGCACCCAAGATTCGTTGGAAATTTTC

TAGACAAACTCCACGACATCGCACTACTGAAACTCCCACTGAAACTGAAATTCAACAGCATTATGAAACCGGTGTGCCTG

CCAGAAAGAGATATTCACGTCATGGGAAAGCCTCTCACGGTGGTCGGTACGGGATGGACGGAGGCTGCGGATCAACTTAG

CCAGAAGGCAATGTATGCCCAAGTGTATGCCATGAAGAAAAAATATTGTCTACGTCCATCGCTAAGATCCTCAATTACCA

GAGCGAACTGGTGGCCCRTGCAGAGACGTGCTCCCATTATCTGTGCCAAAGGAAAAAACACAACTGCCTGTCCGGTGAGT

AGACTCCAATTATATCTAACATCTATCATCCGTCCAAAGCCGAAAGTGTGAGGAGAGTTGATTACATGCTTTAATAATGC

TATGCTTATCCTATATRCGCAATCGACCATCCATTCAGCAGTAATACTAAGCATTATACTATATGAACCTGAACG

>CK182271_bb No definition line found

AACAATGCACTGAAGGGTAATTTATTCATACTTACATGCACTGTACATATCAATTGTCATGAGTTTTTTTTCTTAAGCTG

AATGAATTAAGCATTAAAACATGATAACACTAGTAGCAAAATAAATGGAAAGGCAACATCATTCACACATGCAATACCCT

TTCTTTACACTTAAGATCTGTATTGAAAAAAAGTATCACAGAAAAAAAAGAGAGAAAGAGAGAGCAGTAAACAGTTCAAA

AATGCTTATTTTGTGAGGGCTTGAACAGAAAAGATGAATAAAACAGATCTGCCATATCAACACATTTTTTTTTCCACCAC

TAAACACAACTTAAACAACGAAAAGCAACAAGAAGAAAATATGACAAGTCAAAAAAGCACACGAACAATGCCATTTCCAA

AAAAGACTCTTTGTATGAGACAGTAAAACAATTTTCTTATTGGCCCCTTCAGATGGAGAGGTGCTTTTAACATACATTTA

ACCACTATCTTATATCTATTTACATCTCCAGACTTGATTGTTACTTCAAGTTTGATGCCATGGGAAAACATAACACGTAT

TAAACAGAATTGGCACTTGCAAGACTCGACCTGTGCTTTGTCTTTTTTTAAATTGCCGATTTCGATTGTGCACACCTGAA

CCTACTGATCAATTTTTTCACTTTACCAAAAATACCAAAGCCTGCTTGCAAGATTTCTTTCCTGTTATCAACTCCAAAGT

TTCTCGTGACAGGAAATGACAAAGATTTTCGCCACAGGACTCCTGCCTACATAAACGTGCCTCATTATGCATACAATTTT

AATCTAACTTCTACTTAGCTCAAATTCTCGACACGTAAAACCACACTGTTGTCCAGATAAAAGGTTATGTCAGTGTATGC

ATGTGTGAAGTTCGACTTGCACCTGAGGACCAGTACAGCACAGTTGATACCGAGTCACTTGTTATCAACCGATTTGGGTC

ACAGCAAACACTCTAGA

>CK182331_bb No definition line found

AAGATTTATTTATTTATTTATTTATTTAAATCTAATCAGCCATGGAGGCACTACATAATAGTTGGTCAAATAACAAGGGA

CAGCAATATACAAAATAAGTAATGAGAACATTGTATGATTTTACAATACTTAATTGCTGGAAGCTCAAAATGGTATCTGC

TAGTTCATCCCTAAAAAATGACACGTCCTCTGTTCAGGCGATATGCATGATTTTACAATACTTAATTGCTGGAAGCTCAA

AATGGTATCTGCTAGTTCATCCTTAAAAAATGACACGTCCTCTGTTCAGGCGATATGCCAAGCGCGATGAAGTTAACTTC

TATGTGTGAGTCTAATTACGTCGGAAGCAAGAATTCATACAGAAACGGCATCTAAATATGAATAATCAAGCTATATATCG

ACGAAACAGCCTGTTTTTTTTATGTTACACGTGTGTCATGTGATTACGAACCCTAGCAAAGCATGACTTCAGCACAAAAC

GTAAAAAAAAAATGTGTGCCTAATATGCCGCAATCTCATCAATGTAACATCGAAAAATTGCTATCGCTTTAAAACAGTAT

GCATAACGGGCCCTTTGTCACTTAATGTAACATAAGAAAGCCGAAATACGTCATTCTGCTTTAATAATGTACATTTTGCC

CGGCAGCTTCTGTGCGCAGCTTTTTCAGACCTCAAGCCTACTGCACATAAATATCTCAGTTCTGCTGGCAACTATAGAAT

CATAGCTAAGACTTACATCATTCATTGGGATCAGTGCCGGCAAGTTTARCGTCGCTACGTAGCTTTGACTAAAASTATRA

CATATTAAATACTGTCTGCGTTCTACCTT

>CK182401_bb No definition line found

TCTTACGACACTGCCCCCTTCAGTACATGTATAAGATGTGCACAAATAACAGTGCCACGCAATAAGCGGACAGCAAGCCT

TGCACTCAGATGTTTAAACCGAGCGAAGGCTGCAAAAAGGCACATTTCATGGATAGAACAATTTGAGCAAGCTCTGCCCG

TACACAGAGACGGGACATTTTTAAGTTTGCAAAGGAAGGGACATTCAAGCATGGTACCTTTTGCTGCCTTTTTAACGATT

GCAAAACACCCTCCCATAACCTCCCTTTGCACTAAGTCACAACACTTAGAGGTAATAGCATAAAATACTGCACTAAAATT

CAAGGCTTTGACATCGCAGTGAAATAGCAAACGTCATTCTCATGCACGAAAACATTTAGTAGTAAGCGTAATATATGGCA

GCCTTACTGCCACAGAAAGCAAATTTTCACACATTATACTCCAGTCACAATGGCAAAAACTGTGTATAATAGACGTTACG

TTGATAAGGCACACAAGCATGCATGTCTGCAAAGACAATACACACGCGGTGTTGGTAAGGCACACAGGTCTAAAGCAAGT

TTGGCTCATGCGACACAATGCACACAGTGTTGCAGTGACTGTGCACTTGCTCTACCCAAGCTTATCAATAATGGACGGTA

ATTGTCCCTCTAAATATGTTTACAGATATCAGCAAATCCAACATCAGTTGAACTGCTGATTAATAATAGGTGCTTCCAAA

ATTCATCAACATAAGGCACTTGAAGTGGACAGCAGCGTTTCATGCAGAATCCTGCACGTAAGAGGCACAGGCAGCAACTT

CTTGGTATCCAAAGCTGGACGTTAAACCTTGAAAACTGCACGGCCTACAGATGTTCCTGGTAGAAACACAAGGCACTGGA

AGGTATGGAGAGGGCGTTCATGCTTCTTGGCCTACATGACTTGGGCTTGTTTYGATGAAATATTAAAGTTGAATGGCAGT

GCACCAGTCTGCTTTTTCTCAGAAGGGAGACAGTCCAAGGAAGAGGTTGGTAGAAAGC

>CK182431_bb No definition line found

AAAATACAAACGTATCTGTCAACGCTGAGAACAAAATACAACAAGTGCACAAAACAGTGTGTGTCTCAACAATATACATA

CATATAATATACGTTTTGCTTACACTACACAAATATGAGTATATAACTATGTACAAGCACAATTTGATTTCGGCTCTTTT

CATAAGCTCTCTATCACCGAACGTCATCTAAAAATATACTCTTTAAAATAAACTACTATATCGTCAACAGAGTCCCGTTA

GTTGCTACTAAACAGCACTGTATAACTGTTGAAAAAGCAGCATTACGAGTTCGTAACTTCTTGTATTCTTTGGCACACCT

GAACAGATGGAGCACATGAGACAGAGAGACACTAATCTCTGATTTCAATGGCCACTTAGACGACGAGGCCAGATACTGAC

ATCCAAAAAAAAACTGTCCAGTATACGCGTGGTGTCGCAAAGGGAACACTGAGGCGGAGCGTTGATTTGGGCGTGGATTT

CGTTCTATCACTTGGTGTATTCTCGTCTGACGAAAAGCAATGTGGTCCAACGCTGGGAAGTTTGTCTTTTCAGCCACTGC

GTTCTGTTGTTCAAGGCAAAGTCGCAGGTTCAGTTTCTGGGCGCAGCCACGGCARCTTGATGGGRACGTATTTTAGGCGA

TCTATTCACTTCCAAATGCTTGTACATAGATGGGTCGAAATCAATCTGAAGTCATGTATTACCGGGTCTCACATCATAGA

ACGYTGCTACACGATGTTRAATCGAACAGTGACTGAATTTACTGAACATCTCTATTTTTATTATTATTATGCG

>CK182432_bb No definition line found

CCGGTTTACACGGTCGACACGGAGAAGCCGCCCAAGGAGGAGAGCAAGACGGAAAGCTCGAGACTCTAGTGCGTTCGTCG

CCACCTTATAACGGGACATTCCCTCTCCATCGCGGACATGAAAGCATTTTCCACTATTAGGCACATCGACACCTTTCCTT

CTTGCGGCAGCTGCTTCCGTGCCATCAAGGGAAGGCCAGACAATGAGACTTAGAAGTGTGGCAGCTTGGGCTAGTTGGTA

ATGCATGACGATAGTTATAGTGCGGGATCGGGGCGGCGGCACCGAGACAAGAAGGACACGAGTTGGAACTTAGCGCTCGT

GTTTTTCGTGTTCTTCTCGTCTCTGTGTCGTCGTTTTGGTCTCGCGCTATAACTATCGTCATTGCCAGACAATTAGACAT

CGTTTGTTCAGCCCTGCCTAAACACCAGCGCCACGTGCAAGTGTAGCGCCGTCCGCCAAATTCGCCTCTTGGCTTGATGA

GAAAGGAAGAGGTGCTGAAGTAAGGCATAGGGTATTGTCCGATGCGCCGTGTCCATGTCCGAGGCATTGCTAGAGCTTTC

GTTCACGAAAATAAATTGCGGGGCAGTAATTTCTTTYAATCGTATACGTTATGYCCATGAGGCAACACTATTTTATCAAC

AGCATTTTRAGATGTTTGCCAATGTGCGCCCCTTCACTTTCAGTCAGCCGATGGTCTTGCAYGAAGTGCGTKTCTSTATG

AGGCCTGTACAGGCTCAGTGGTGCTACGTAGTGACGTTTCACAATAACAATGCAACTCCCTCCTCTAACATTCTCAT

>CK182461_bb No definition line found

GCGCTGTGCAGCTAGGTACTTCCACCGATTTTCGTCCCCGACTTTCCCTCGCTAGCAATCGATTCTACGTGGTCTGTTGC

GAAGGCGTGCAGGTGAGGTGGTTTCCGTCAAGCGAAATTCTGTATTTTTGATCGTTCCTGTATTAACCATGGCGGAGCCG

TCTGATGTGATGGCGATGGAAGAATTCCGCGGTAACATTTCTGAAATGTCTAGTGGCGGAGATGGTGAAAGCGCCGTCGA

AGACACGTATGTCGGTAGGAGGAGACCTTCGCAGCCAGTCGGCAACGTGGCAGCGGTATCGCCGAGCACCTCCTCACCGT

CGCTTGATGAGACCTCTCCGATGCTGTGCACTGAAGAGCAGCCGACGCGTCCGAGCTATTACATCGAGAACATCCTTCGC

CATCAGCAGGCATGGCACACTGGTCTTCGATTCGTTAACCAACGGCAAAGGCGCAGATCCTCACCTGCTTTGGACGGATG

GTTCGAGCGATGGTCCCTTTACTGGAAGCAGTTGCGGCGTGGTTCCCAGTGGGCGTGCGCTCTCCATGTTTTCTTTGTTT

TTATTATCATCGTGGTGCTGTTGGTCATGACGAAGATATTCATGGACAGCCTTACGAGTTCAAACGACCACCAGAATGTG

ACGCGTGTGTGATATCCTACAACA

>CK182681_bb No definition line found

ACTTCATTTGCTCGAAACAGCTGAGAATATGTTATGAGTGCTGAGAATATGTATATGAGTGAATAGCAACAARCTTCTGT

GCAATGAAGTTATGATTGGTCAACTTGTGGCCACCACTAATGACAGTGTGCAAGCTGTCAATTGTTTTCCACCTTGCCAG

AACATTCAGGAGAGGTTGCTGAGAGCATTTTTTAAAACCAGAATTAACATTCTTCTGCGGAAAGAGAATATGCGTTTGGC

AGCTGATGAAGCTAAAGATGCAAAACTGGTAGTCGTAGCATTGGAAAGCAGGCTGCTACAACCAATGTAAAATTGTGTGT

ATGCCTCATTTCTTTCGTGTTTTCCTTCCATTTTTTCTATGATGCATATGTGCTATGGCCTCTTAACCTAGTCTGTAGGT

ACAGTACTCACGGATTGAAATGCAGGAATTATGGTCTCAGTAACGCACATACTTTTGTTGACACTGCATTAAGTTCGGGT

CATATTGGCGTAATTTCAAATAGAACATGTATATCAGAGCCAAAATTATTCTTAGAGACTATATTTATCACGACATGACG

TGCTTATTTCGAGCAGGTGTGCATACCAGTTATCCCCGAAAAATTGATGTTGCGTTTGAATCCCAAAGTACCGTACATGA

GGAACTGCAGTGTTTTGTCCTATTGTGATTTCATTTCAACAGTAATTACTTATCATTGATGCGCACAGTTCAGAGCAGAT

GTTTTCTGTTGCAGTCATAGTGTCATTGAAATTTCTAACACGTTTGCCCTGTTTGTTTTAATGTGTACATTTTGGTCCTT

TATAAGCAGTTCCCTTTAAGTAGCTTGGGTGGTGTCTGGATCTTATTTACGTTGTAATTCTGTGTG

>CK182792_bb No definition line found

TATGTCAAGCATTAGGTACGCTCTTATTACCGGGGCTGGGGCTTTTGCTGTGATATCATGGCAACGTATAAAAATAACGA

CGCGACAATTATGTGAAGATGTGCCCATTGAGGCGCACAACGTTGTTTTGGGCAATCAGTCCCCCTCCTTTTATGTTTTA

AAGCACATTTAGTAACGTAAATATTCTTCTTTAACGTACTAAGCAGCATTAAACACTAATGCTTGCTTATACATTCGAAT

TATAAATTGACATTCTTTCTGTAAACACGGAATCTATATACAATATGTTCAGTTGGCAATCTCAACCCGGTGATTGGTGA

ACGTCATTCATGTTGCGACATATTTGATGGTTAAAAATATCTTTTCCCGTGATCTTTAAGGCATTCAGTGAAAGCTTAAG

CACGAACTTACCTAACAGGACCTCAAAAACTCGTGCGCGTACTGGAAATTAGATTCCGCGTTTTCAAAGGGTATGAGGAA

AAAGTATGTACAGAACAGGAGCGTTTCCCGTTTCTTATTACGCCGAGTACGTTGCACTACCGTGTAATACAAAACCTGAG

CGCGATCTTTGCGGAATACACGGCAACTTCACGTCGCGTGGCAGCTCTTACCAAGTAATTACGCAGAAAACGGAAACTAA

ATAGTTGCCGGATTCGTTGGCCCAACATCTTGCGAAAGGGGCCGTAGAGGCGGCTCTCGTCCTGTGTGCTTCTGAAACGC

TTCTGCTGTCCCCTTCCCAAGATGGAGGGGCACTTGACGCAGCTTAACGTAACCGACGTCGTCATCTTCCGCAGTCAAAC

AAGCTAACAACAAGCGAGCACTGCGCTGGGACGCAGTGCGAGTGAATTCAAAAGAACTTTGGCTAGCCTGTTCATTCGCC

GTGTAATGCGCGGATTAC

>CK182850_bb No definition line found

TTCGGCGGTAACCGCGGTGGATTTGGCGGAGGACTTGGGATCGGTGGCGGCGGGCGCGGCATCGGAGGTGGTGGCCGCGG

CATTGGAGGTGGTGGACGCGGCATTGTAGGTGGTGGACGCGGCATCGGAGGTGGTGGACGCGGCATCGGAGGTGGTGGAC

GTGGCATCGGAGGTGGCGGACGCGGAATCGGCGGCATTAGAGGATGATTCTACACTTCCACGCCGAAACTTCTTCCTGTC

GTTTGAAACGTGGAGGCAGAAGGACCAGTAACGTGCAGAGACATCAGAGGCCTGCGAGATCCCTCTGCACCTGAGGTCGC

CGGAACGTGAAACTGTGATGTGGTTTTATTCAACACGACCTCGAATAAAAATTGCCTTCTTTTATTGGCACCCGTAGACT

GGCTGTCTTATGTGCGCTCAGTGTTTGAAGATCTCATAGTAGCAAAGAAGAGTTAGTTGAACTCTTGTACATTGTATTTA

ATCTGGTCTTTGAACAGTTCAG

>CK182912_bb No definition line found

AGTGTTATATTATCAAATCAATTTATTTTTCACAGCATATACACTTAAAAATGGGTACCTGGGTAAAGAGATCGTCAAGT

TACTTTAAATGAGGCAAGGCAAACTAAAAARAAGGATGTTTTCAACTGCACCCAAATAAGTAAGATTTTGGCATTTCAAT

ATTATATTTTAATGACTATATGCAATKATGCAATGCAAGAGCAATATGCTTAAACTTTTTTTGTGGCAACATCCACACCC

ACGCCGAATTTCCCCCGAAACCAGTCACTTAAGTGACATTACCACCTTTGAACTGCAATGCATTATTACTGAACACAAAG

CTCACACATGGATTGGACAACGAAACCATGCCCAAGCGATACTCCCCAAATAACCATTACAAATGAGGAAAACCTGCAAT

GACTGATGTAATAATGTGTGCATGAAAAATGGCTAACATTCAGGACCACAAACAAGAATCATAGCATGTATGACAAATTA

CTTTRCTGCTTTTTTTTMTCCCCTAARGCATGAGCCAAATGTGATCTCTGCCATGATCTCATGAGTAGCGAGCTGCAGCC

CCAGATAGCTACAAAAYATTCACAAAAGTGTTAAATTTTGGTGCTTTTAGATGATCCACTTACAAAAGGATATCGAGCAA

TCTGCTAATGTCAATAGTCATTATTAAAGTTGTTCAACTCAAGACTTATCTTTTGTCACTGATTGGCACATTGGAATTGA

ATAATCATGAGCCTGTTTTACGTGCGAAATGTTTTGGCAACACTTACTCTACC

>CK182938_bb No definition line found

CGATCCGGGGAGATGAACTCTTTKTCTGTGAAACTATTTATAACCCGTGTCGAACGAATCCCCTTCCCACTGCTTTCTGT

TTTGTCTCTCACTTTGTGTATAGATTTTTTTTTTGTRAAGAGTTTGACGCGCAGGTGCTACCCTTATCCACATTACCGCA

TTGGCGATGCGCGTCTGYTGACGTCAGTGTCTGGTGGCGACATCTCGAGTTCAACTACACAGTATGATAGGAACCGCAGA

AACACAATTGCTTTCGCTTTGTTCAACTAGGCCTAGTTAAGCCGAGTTAGCACTACAAAATGTCGCGCTCTAAAAAAAAA

TTAGCCACGCGTTTTGCTGCTGTAGCATGTGGAGAGGTGCGTTGGAGAGATGCAAGGCAGCTTTGCGTCGTTGACATAGC

AAACGAAGCAATCAAAATGGTGCACTGTCGATAGTTGTTGTGCTGCGCTGTTTTGTTTGGGAACATTATCACTAATGTAC

GAATGTCGATTTTTTAGTTTATCTCCGAACATGACAAACAGTACAGGCAGCGGAGTATTCACGACACCGAGTATTCACGT

AAGGCCTATCCAGTAAGTCATCCACCTTCCCGAAGTGAAACGGCTAATCAATTTTTATCGCGTAATACGCACCCTTCGTT

GAGCGTTTAGAGATCAACAGATCAAGGTGCAGGTAAAATTTTCGCTGTGCTGGCTGTGCAACAAGATAGCAGCCTCGAAT

ACGTAAGGCTGCGTGGGGGCGCTACCTGTTACCACGAGATGTCGCTACTTGGGCTCTGGATCTCTGCGTGGCGGTAATGC

AGGGTGACAGTGGCACACGCGCGTTACGCCGAAACAGTGTAATGACGTCGTCTGACCATTTTTATATCACTGATTTTGAC

TCCGATGTTTTTTTTTTGTTTCTTTTTTTTCATCGTCTGTTTCCCTCTCTTGCGTGGTGAGCGGAGCACACAATGCCTCT

CCACAAGTTTGAGTCTTCTCACATTTTCTATGGCATGACTGAGTGTCATTTCTATTGTTCTTTCTTTCTTCAAACTGAAC

TGTGCATGAACACACAGGATTATGCCTAGCTGTCGGTCTTCTTCTTTTGTTTCTATTTTT

>CK183006_bb No definition line found

TGCTGTTCGCGCATTTGAGAAGAGGCCGGTAATCTATATTCAAGTCTGCCAGCCTGAACAGCGAGAAAATCCAGCTTTTT

TTTGTTGTGGTCTCTAAACTTTTGATACCAACTGCAGATCAGCCCATTGGCACAGCCATTGGAGTACTTAACACTCCCAG

TGGCCCTGCATGCAGTAATAAAAACTCAAACTAACTTWTTTTGCATTGTTTCATTGATTCCTCAACAGCATGAATTTATG

TATATATTTATAATATGTACATCAACAGACACACACTTTCGGTCAGGAAACACAGTTCTGCAGGTGTAACACGAAAACTG

GACATCAAAAAAATATATTGGCAAGAGTAAAGCAAAACACAGAGAGAGAGCCAAAAGTGTGTAAGGGCTGATTACACAGC

AAGACTTACACGTCTGATAGCATCTAGTGGAATGAAATCCTAGTGGCCGTCGAGCTTCCGACAAGTGAGACGAATAAAAA

ATGTTAYGCACTGCATGTCTACAAGTACTACGCCCAACCCTGTTTTGTATCCTCCGAGTAGCCACCTACATGATGAATTC

GCAAAACTAGTTTCTTCACTTTTTTTCAAAGGTCAGCACTTTGAGTAAACTGACATGACAATTTACGAAATCTCCCCACA

CCACGTCGTGGGAGCATCGTATCGGGGTGAAATTCCTTCTCATCTTGCTCAGTTTTACAAAACTTCAAAACTATTTCAGT

TTTAAGTCCTTGGCAGTCACGAACATAGTCCTTTCATGCCCTTGTATTTTATTTGTCCCCTGTACTCATATGCTAATCAG

GCACTCGCACCCAGAGTTTGGCCTATGATGTTTTAGTTTTTGCACAACTTGTCAATCTCTTACACTGAATCCAAAATATG

ATGAAATGCCACAGGCATGCTGTGCATTTGATATGCAATGTGATACATACATCTAGCTCTTCACAAAAACATCAAGCCTA

ATGCTTCTCAAATACTAAGCTTATAGCAGAACA

>CK183072_bb No definition line found

CATATCCAAGGTGTGCATACTTAGAAAACAATATATTTTTTCAAGTGCTTGGAAGGCAATCTGTGCAGTCAGATAGGCTT

TTATTGGCGTGTGCCATAAATAGGTTGCCTTTACTGTGTCCTTTGACTGGTGTAATGTGATACATCCTGCTTGAAAATAT

TACGTAGGTGCTAGTCGATCGAAGAGTGGCAACTGCTGTCGTTATCCATGTTTGGACGGGCATTATTACCATATTGTAAA

TAGGCATGCCTTGTAAATAGTGACTCCGTTTGTTGTTTGCCTTGTTTTGTTGTTCACATTGAAATATTAGTGTTATTTCT

ATATGGAGTGGGCGTGAGTGCCGATAGCCTTAAGTTCTTTCAGCATATTTTTAAGTGTCAGTTTTTTAGAAGGTGGCTTC

AGACTTGGCTTGGCTTGTTTGCTGACTGAGACTGGATAGATGTGACAATGCAGGGTAAGCATGCATATTGTGCAGTAAAG

CTTGTCACATGCATCCGGTGTTTGCAACATGGTGGATGCAGAATGACAGAAAGATTTCAGCAACTCGTTGTTTTTTTAGT

TCATGCTGACTCGTTTACGGTTACCGTAATATTTCCGCAAGATCACTTTTACACTCATTGTGTTGAGCCAAACTACTTCA

AAAGCAAGATGTGTGTGTGACTGACCTAGTCGGCAACATTCCGTTAAGACGCATAGTACAGGAGACCACGTGCTCAGATT

TCTTACTGCATGACAAGGAGAGCATTTTTTGGCGTATCAATTATTCTAAAAAGCTGCCATTTCTTTTAAATTGGCACTAG

AAGGAGCCGTGATATTTCCTCACTTTTATTTAACTTTCTGTTAAATATAAAGGAAAATGGCAGCTTTAGAATGAGGCTAT

TTGGTGGAATC

>CK183089_bb No definition line found

ATTTGAAACGGGAAGTAAATGACATAGAACCTCACACTGTGCTAAACTACCAAGGTTTGCTGGCAGCCGAGAAAATGGGA

CCACTTCTCCCCAATGCTAAAGCTGGTGAGGAAGGCTGCTGGCGCCTGGTACAAGATGCACTRCYARAGTTTATTCCTTT

ACGCTGTCTATCGTACGAAGAGCTCTGTACTAAAGCGATCTGCCAACATTACTGAGAACATTAGAAACCATGTGATCCTC

TACGTTAACTCTGCGTCGTGGATGGATGCTACCTTAAGGCAACAGGCAGTTGAGCGGCTGCGCAAACTGCGTGTGTTTGC

TTTCGTACCTGGCTGGTTCTCGGACACTGAGCGGGTGCATGCCGAGTTCTCACGCCTCCCGCATCCCAGCATGGACAATG

GGCCGCTAGCGTACATCGCGCTCAAGGAATACACCCATGGCCGGCGACTACACGGGAAAGCATGGCCCCTACCACCTGAC

AGCACACAGTGTGCCTTCGACGACACACGGCACACTGTGTACGTCCCACTGCTGGCTACCAACCTAACCAAAGTCCAGTC

TGCACCACTCTTGCTGGCGAGGTTGCCACGTCTGGGTGTGCAGCTGGCAGCTTGCCTTATGCATGCGGTGACCGCAGAGG

CAGCACATTGGTGGGGTGAGCGAAGTTTGCGAAAGTTGGAGCCGCTTCTCAAGTGTGTCGACTCGCAAAGTGCCATCACT

GAGCCTTCAACACAAGGTGTTGTTTTGAATCACATGGCAGCATTAGCACCCGCGCACAGTGTCTACTCAAGCCGTCTGCG

CAAAGAAGCAGCACAGAGCCATGAGTATCGGCTTTAACATGCAGAGAACATCACGATGGAGCAGATGTTCTTCATACAGT

TCGCTGCAGCACGGTGCCACGGCCCTGCACAACTAGTCAATGTTCCACTGAAGAACTACGAGCCCCTCCGCAGAGC

>CK183113_bb No definition line found

ATCGTTGAATGTAGTCTATTGCGCTTCGCGAGTCGCCTCTAGAGCGTGCACAAGACATCGCAATATAAGCGCTCAGCTTT

TATCACCAGGGCTGGCCACATCGAAACAGTGATATCACGCAACGCCTCCTTCCGCTGTGTCAGGCGTCGCTTCCGATTAC

TATACTGTTTCGAAAAAGCACCGTGGTCACTTAACGCCGGTGCCGGCCATCGTGAAAGCGTGCCAACATGCACTTTAAAG

TTCTTTGATGAATAATAGGCCTGCTGAAAAGCGAAAGAAATGTGCTTCAGAAAGAGAAAAGGTCCCGGTACTTGGCACAA

TGAGCGGCTGGACACATACGACGGCCAGTTCAGTGCGACACTCTCTTGATGAGAGGACGCGTCGATGCCACCTGCTGCAG

CGGACGTCAGGTCGGCAGCCGTATTGACGCGAGAAGAAAAACTTTTTGGTGTCACAGTAAACTCCTGCTTTGCCATGGTG

GTACTGCCGACAGCGTTTATTGTCCTGCCTCCGGTGTATACTGTCTTGAACGAGAAGTTCGACGTCAACACCGCAGTAGC

GAGCAGCAGGCAGAAGCCAGGGTCTAGGCATGTGACAGATGCCAGGGCTGTGACTGGGCTGTGTTGTTTCCGATGTGTCT

TCATGGGCCGGCTCCGTCAGCTTTTGCGGACGACATTCAGAGACTACGTGGGTTTGGAAACTCCAATCATGGTTGGACCT

TTCAAGCCAACGGTTTCACTATAGAAGATGCACTTTGCGAGATCCAGCTACTGATTTCAGAGGAGTGGGGGGTGCCTGAA

CTGACATTGGAGAGGGGGCCGTGGTCATGGCTTGTTGTTTT

>CK183170_bb No definition line found

TCCCTGCTTTCAAACAGTTTTATTAAAAAAGTGATTGAGTCCTTTGTCACAAATCTTTGGAGCAATGGACAACAAGAAAT

TCTGCACAAATAATTTTTGTGGCACAACAACTCGATCTCGAAGCTCACAGCAGTACACATTAAGAAAGRAGTYACCGAAA

AGAATCACTGTGTTCTTGATTTAGTTAAAAATTGCAGCTCATTACAYGCTCCAGTATCTGTTATGCATGAGAAAGTAGCA

CTGATGATTCCAAAATTCGAAATACATTTTGTTGTTTTTATTTATCCACACACGTCTCCCCAGCTCTTCAGGTGCCYWAC

GTGCTTATGCTTACACTAACAGAATGCTAATACAAAATGGCGGGGGTTTCCAGAGACATCAACCCATACACTGAATGCAC

GAGGGCAACCATACTATCCTGAAATGACCCACATATGAGGTATGGGAAAAGCTTTCTCCAAGATGGCAATGTTAGCCGAC

ATTTTGTTCGATCTCACCCTCCAAACACCTTTTTACAACCTTTACTGTTGCAGCCCTTACGGGACAAAGAAGCTTCAAGT

TTGTGAATGCGGCCTGCTAGCTGAAATTAAATTGAGACTCCCGGTCTACGTGATACCTTACTTCTTCCTAATTGTTTTGT

AAGCATGACATTAGTGTTTTGAAACCTATTTACTGTGAACAAAATAACAAAATAATGAGTTGTAGCAATACAATGACCAG

AGCCATATTTTTCCACATTGTTCACTTAAAAGCCATTCTAAGCAGAAAGGTAATATTGGC

>CK183171_bb No definition line found

CTGCAAACAAATGCATGCATTCTGCATACAAAGAGTAAAATCGAAAAACACCTTGTTAGCAGACAAAGGGCTTCATTTTA

AACATGTGTCCTTGTTCTAGTTTTAATTCTTTAGCCCTTGTTGTTTTACTTGTATTACAGTGGTCAGTTACGTGTTAATA

ATGTCAATCAGATCAACTTGCACACTTGCGACATGCCACCTATTGCCAATACTACTGCATCAATAGACGTGAGCATATGT

GCAATCTAATCATTCTCATAATTGCTGCATAAACAAGCTACATCTTTAGAGAAGCTGCACACTTAGTGCCCAGCATGCTT

CAAATATCAAATAGTACCATCACATCATATCGAAGGTGCCCTATACATTTGTTATACCGGCTAAAATGTGTKGCATACAA

CTTTTGTGCAAGTTGATAGATAATCCTTCAGGTAGGTGCTACTTCATATCAGCAACTTTTCTTGAGGGTTTCGTGCACGA

ATTCTCGAATGSTTGCTCTTATTCTATTATATTTATTTTTCAATCTTTATGAGCGTATGTTTATTACAACAGAATACAYA

ATAGAAAGGATGATCTCCAGGCCTCTCATCCTTTATAAAAGGTTGTTTTGGGCCGGTCACAAGCAGCTTTTTTGAGCAAG

CTGTTCGAAGATGTTTCAATGCAGCAAACACAGACATGTAGGAGTAGTGTTTGGAACCATATTTATGCAGSACCAACATT

GCACACGTGCGAGAATAGTAAAAGCAGCTGCTAGAGTTTAAAGCTGCAGATCTGGCAGGCTGCTATTTTTATCCTTGATT

TTGTTGTTTATGCAACTCATGTTCTTGTTGAAATGTTTTTTACAGCACTTAGAAGCACGCTTCTTTGTCTAATAATTRCG

CACAAGAGGCACATTATTTGTTTTACATGAGGTTTTTCATTATCCCAGTCACTCATAGAATGCATGCTTCATATCGTACC

CCGTATGTTTGCACATGGGAA

>CK183179_bb No definition line found

CGCTCAGCCAGCGTACGTGTGGTTTGCGTTTTACTGAAAAGACGTGTCCGTATCGTACAACCTGGCTCGGCAAAGTGGTG

ATTCGTAGTCCTTCGCAGCACTGCCGAGGTGTCTAGAGTCAAGCAGAGCAACGAGACGCACCGAAACACGGAAGCTACTG

ACACGACCAGCCATGACGTCTCTTCGGTATTCCTTCGACAACCACCTTACTGAGACGGCACCCCTTCGATATGTTTAATC

CAGGCCGAGTCACAATTCACGCTTTGTGGTGTGCGCACGGAACCAGGCAAATGGCTTTCGTTGTTTTGGCACTGTCAACA

GCTGCTGCCGAAGAATTATCTGACCTGCTTTCCGGGCCCCCCGTCACCTCATCCTATGCAGCACTCTCAAGGCAGCTCTG

GTGCAGCGAACAGCAACATAGCGTCCCCGTATACAACAGTTGCTCTCCACGGTGAAATTAGGTAACCACCGGCTTCGTCA

TGTGCTTCAACTCATGAGCGGCAACACCACCACATCTGACAAAACTTGTTTGCGAGAACAGTTTTTTGCAGCGCTTTCCT

GGAAATTTGCAAATGGTACTAACCAGCATACTTAACTGGGCTAGTTACTTAAGCTTCATGACGTAAGACCGCAGCGCAAA

ARACGGCAAACAAAARAAGGAGGACATAGGACAAGCACTGAACTAGCAACTCCGCYCTTCATTACAAAAGAGGAGAATAA

ATACAATCACTATC

>CK183321_bb No definition line found

CTTGAAAAACGTAAGCAGGGAGGGAGGACCAATCTAGCTTTCCTTCTATCACATCACTATGGACACCGCTACCACGAGAC

CAGAAGAAATGAAGGGACGGTAACAGGGTTTTCAAACATTTATCTACAATGACAAAGTTCACACCATTATTTCAAMAAAA

AAAAATACCTAGAGAGGGCACTATAAAAATACAGCAGCCGCTGCGGCAGCAAGACAGCAACAACATTTGCACAGTCTCTT

CGCGATGGAAATACCTGACGAGGATTCACTGGGAAAAAGCGTCGACACACCTGCCGTCACACAACGTGTAAAATGGTTCA

CGTGCTGTAGGAGGAACATTCGTCTGCGTACGCACGCACTTAGGTGCCACAAATGATGGCAGCGATGGCCGTGAACACGT

TTTGTGTCACTCTCAGTGCAGCTGCCGCGTGTGCACAGCACGTTGTTGCCAGTTGTGGGCACGGTTCCTCGTGCAAACTT

AGGAGTTTTTTTCTAAATTCCGCAGTCCACAAGCTTCATACTTGTAGCGGTGCTGCCGTGTGGTAAAAGTGGTGGGCCTT

GTGTGAAACAAACGCACGAAAAGAGAGTGACCATGGCAAAACGAGGACCAGCGCTGCAGTTGTGTTCAGTGATGAACGAA

TGAGTCCTTGTATTCTGGCACGAGCCGACAGAGGATGTGTTGGTTTGTCCTTTCATCAAACGGTGGTTGTTAAGCATCTC

TGGTACTCGGGCAAGGGCTTGACAACACCATCATTGTACACCTTGAGGATAGCCTCACACACAAACTTGTACTGACTAGA

GGTCTGGATGAGCATGGCACGCTGATCC

>CK183325_bb No definition line found

GTTCAATAAATGAAATAATTGGAGTGTTCGTGGTCTACTTTCATTTTCAGTGCAACTTTCGACTGTTGCTTCAAGAGTTG

TCAGTAATCTGTGTATACACATGCAGTAACGTAAAACTGCAGGGTGATCTTTAATAGAGGCCCTCTTGAGCAAAGCCTTT

ATGGAGTCAGTCCTYTTGGGGTCACGCGGGAACATCGACGTTTTCCTGATATTGTCCAGGCCCAAGTACAGCATCGTAAC

TCGTTCAAGGCCTATTCCACCACCTGCGTGTGGTGGTGCACCATACCGGAAGGAGTCTATGTAGGCCTTGATTTTGCTGA

TATCAACTCCATGTTCTTTGGCTCTTTCTGTAAGAAAATCTGGATCGTGAATTCGCTGGGCGCCAGACAGAATCTCTTCC

CCACGCATGAACATGTCATACGAGTTTGACACTTTCGAGTCATGAGGGTCAGGCATGGTGTAGAATGGCCTCACTTTGAG

CGGGTACTTATCCAGGATGTAGAAATCTGTGTCGTATTTTGCCTTGACCAGCCGTCCCAGCAGCTTCTCATTTGGAGTGC

TCAAGTCGTCCTCGTCGTCCATTTCAACTCCAGCTTCACGAAGCATTGCAACAGCCTCGGGAAATTCTAGCCTCAGGCTT

GGTTCCAGAAACTTGAATGGAGGAGTCGGAAACTGCCTATTAACTGCCGCAATCTCGTCGGCAAACCTATCGCGAAGGCC

CTTGAAGATGTCAACAAACATTTGCCCTATGACATCCAGCACTTCGTGGTAGTGGTAGTTGAAGGCCATCTCCAAATCCA

AGCCCACGAACTCGCACAAATGGCGGTGGGTGTTCGAGTCTTCTGCCCTAAATACTGGMWCCAAGCAKWRKAGAMSTGTG

TCTGACCTGAGTACCGAACTCGTTAATGC

>CK183440_bb No definition line found

ATGCATCTAAAAAAGGTTTAATGAATAAAAAATATGTAGGTGGAGCATGTTTACATTTTTAATAGTACTAATGTTTGACA

ATGTTTTGTGAGCACAGGTTGCATACTTCTGCAATGCCCATCATTGAAGTCATGTCATTTTTTTCTCWGCATTAATAATT

TATTTTCTGGGAGAAAGCTTTCATGAGTTGTTGGTGCATCTGTTCGCATTGCCAACAGAGCTGGTACTTAATGCAACGAA

AATACCAAGCAAGTTCCGCTTCTCCGGAGGCGTAATCACAGCTTGGTTTTGGTCAGGGCTGCTTTGCCAAATATGGCCCG

CATGACGTATCTCTCGTTCTTAAGCCCATTGCATTGGGTTGCCTTTGGAGGCAATGTCATACCTACTAACATATGCACTT

TTGTACTTGTACAAACGGATTTGTGTTCCTTCTACTGGTCCTAAGACATGCAAGGTGTTCTTAATTTTTGTGTTGTGTTG

TTGCAATAGTCTGAGGTGTGCCCTTGTTGCGATTAAATTTCTTATTGATTCGAATTTCTGCATTTTCCTTGTTCATTAAG

TCTGGGTGATTACACTCACTACAAAGGTCAATGGGAGATGGGAAACTATTTCAACAGTTCTTACTTCTTGGAGACAAATC

TTCCAGAAGCTTGTAATAGCAATACTTGATGTCAAATTCCATGTCATACCAGTAATTGAGCGCAATACAACCATGGCTTT

GTCGGACATGGTGGAACCACAAGGAAGGAAGGTAGAGTATATCACCAGCACCGACTCTACATTTAACTGGTGATGCTCGT

CGATAGTGTGGGTATCTGTCGAAGTCCGGATTTTCTGGATCTAAGGGAATCCATGGCACCGATGAACCATCACCTGCACA

GGTTGGCAGTAATGGCCAATTGTGACACTGCTCAAGTGGCAGCAAAGCGGAATACTCGAGGTTAAGTTAAGGTAAAGCAA

AATTAAAGAG

>CK183587_bb No definition line found

CTTTAAAAGTTTTATTGAATAGAAGTTATGTACAAATGGTGTACAAAAGTGAAAAATGAAAAGTGTCTATCAAACACATG

GGAAGACTCTTCACAATGTTTACAGGAGTGGCTACTCCTGCATACCAATATCAGCACTGTTGGCCAACTTATGTAGTGCG

AARAATAACATCAAACAAGTACCACATCTACATGGTGTGGAAGCTTACTGTAAATTTACAGGTTCTACAAAATTGTAAGT

GAACCTTTTTCACCAGCTAAAGTTTGGCCTGAAATGATGCCCGACAAGGCTTCATACGTGGGCACACTGACAAGGCGCCA

TTCGCACACTTGAATGCACAAGATGCACTTATGGTGACATTTTACGACAAAAGCCGTTTACAAAAAAAAAAGACAAACTC

TTCGGTTTCAAAAGCAAGCCAAAAACAAGGCGCCATCCACACACTTGAATGCACAAGATGCACTTATGGTGACATTTTAC

GACAAAAGCCGTTTACAMAAAAAAAAAAAAAAAGCAAACTCTTCGGTTTACAAAAGCAAGCCAAACTTGCACCACCCAAC

TTTTCYTTACAGGTAATCATATCGCAAACATGGCGACATTCTCATCTTGCACTGTGGCAAGAGGAGAAAGCCAGGCATGC

CATAATGATGAAAATATTCACCAGCTGGAATAAACTTACTGAGAAGTGCTTACTAGATCGTAATTG

>CK183588_bb No definition line found

TTTGTTATGGTTATTGCCTAAGAATTCGGTTTTATCACTCCATGTTTCATTTCGTTGATTGCTCACTACAAATGTCTGTA

AAAGTGATGCTGCATAGCAATTTCTCATCGAGAAAACATTGTACTTTGGTCAAGTTCATAAGCTCCTCTTCTCAACTGAT

GTGCTATTGAATGTGCAGAGTATAAAACAAACATGGAGCTTCCAACGCATGCCTACTATGTGAAGATTTGTATCATGGTG

CACAAATTTTGTCACTTTACAGTAAAACATCACTGTACTTTTGCAAACAGCAGAACTCATTTTTACAAATCATGCATTGC

CCTCCTTTTTTTTTAAGGTAACCCATAGCCAAATTGCAAGTTAATGTTCTTTGCCCATAAACATATATATCTTGTTCTTG

TTTCAGCTGCTATTCTGTATCTACTATCCTACAGGTGCCGTAGATGATAAGATAGGGATCCTTGTTTATAAATAGAGCTA

ATTGGCTATGCAAAAAGGGAAAAAAAGTTTGACATTTCATGTTGAGCCCCCTCTGCAAGAGGTGCCAAGTCTCTCCATAG

AACATGTYTTCCCCTTGTGTTAGTGTAAGCTCTCAAAATTCAAAGTAATAGTGATTGATTAGGCTTGACTAAGAATGCCA

GGTATATAAAAAGACTACACAGTGGTAGGGTTTAGATAAATTGACCACAAATTTTTGTATGCTTTTAAAATCTCTGTATT

TCAAAAGCTCTACATTTCAAGTGCACTCTTTAACTTTGCCTTTACAAATTCCCTCCCTTTTGCCAAGCTAGCCAGCAGGC

AGCCTTGTATATAACAGCCCACTGCCAAACAGAAGTT

>CK183629_bb No definition line found

TAACGTTGGGCGTTGGCGTCGCGATGTCCGCTCCACTCGAAGCGCTAGAAACAAATCTAGAAATGTTTATAGAGAATGTT

CGGCAGTTAGGGATAATTGTTAGCGACTTTCAGCCTCAGGGTCAGACCACGCTAAACCAGAAAATAAATCACATCGTCAC

TTTGATGCAAGAGGTGGACAGATGCAAGTCACAAGTCCAGAACATACAAGTGCCACTGGAAGTGTTCGACTACATTGACC

AAGGAAGGAATCCGCAGCTGTTTACGAAGGACTGCATGGAAAAGGCGCTGACGAAGAATGAGCAAGTCAAAGGCAAGATC

GAATCCTACCGCAGGTTCAAGGCACTGCTGCTTCTGGAGCTAAGCAAGGCATTCCCRACCGAAATGGCCAAATACAGGGC

TATCAGAGGYGACGAGCGACCAACAACATAAAARCCTTTTGCAAGAATCTCTGTGAACTGTAAATGCATATGAATTAAAC

CACTTGAKTMTTGAAAAACTGATGCAGTAATTCAATATATATGTATTATATCGCTGAAATTTCTGTGCAGTACTTATTGT

TATGCAAGGTAGCTATTTTTTTTCAACCTATGC

>CK183753_bb No definition line found

CTTTCTTCCTCGCAAAACGACAGCGCATGACTTTGCCTCGGAAGAACGGCCCTAGTAGCAGTTTGCATGTTGTACCGAAT

GTCTTCGGTGGAGGGTGATCACGATAAGATGGCAATGAGCAATGGCATTTAGTGGGCACACGTGTCCTTTTTGATAGCAT

GTTTTTTTTACCCTAGACACCATTACATTGTGCAACTTTAAAAGAGCAAAAGGGAAGAGGTGCGCTTGGTAGGGAGATGA

TGAATTGAAGCATCTTAGCACACATTGGTAACAGGCCAACCTTCGAAGCAGCCTTTGGAATGCAGAGAATTTCAAATCTT

CTCACTCAGGTAAAAAGGGCATTGACAAACTCTTGCCCTGAGCCAAAGGGCAGAACACACGAGGGACTAGGCATTTTTTA

CTCAGCCAAGGGCTTTTATGCTCCACTTTATAAGAAATTGCTTGTTCCAGTGAAATTTCTTCACAGTTTGGTCAAACAAC

ATGCGATACATAAAATGCAATGCACTTTTACCTTTCAAGCAGGCCAGCAGATCTCTTCACAATGCAATCTGACATGCAGG

CTTTTTGCCCAGTATTGAAAAAAAAAAAAAAGTATTGCTTCGTGCTTGTGTGTGTTCTGCTTCATTGTTTTTCTTGGTTG

ATTGGCRAGAGRGTGGGGGGTTTTTTTTGTTGTTGTTGTTATTGTAAGATGAGTGCAAGGGATTTATTTTTGTGATTGCT

CATTTTGATTCCGCTTTTTCATTGTTGCTTGTTTGAGGACGATGCACTTGTTATTCATTTTCTCTGTCTTCTATCTTTTG

ATTTTTTTTTCATTCAGGACTTTTCCTTTTGTTAATTGGTTGCCTAGCTTGTTTGCTTTGCTGCTGAGTTGTGTACACTT

GCATGTTGCTGCTTCGAAAACTGACGGTGTTTTTGCCGCTGCGTTTGCCGAAGTGTGCGTGACTCGT

>CK183756_bb No definition line found

TTTTCTAGTGACTGCCACATTTATTGGACATGCAAGCAAATGTCATTGCATCGATCTGAATATCACATAACAATGAATAC

ATTTACACGAAATCGTGTTGGCAAAGAAACAAGAAATCCCGCAGTAAACTTAAGTGCGGTTTTGTCACAACACTTGGAAT

AATTTCCAATCATAAGCAATGTGCATGGCAATATTTCTGCGAATAATTATGATCAATATCACACAGTGTTTACTGAGCAC

TGCTGAAAAATACGCATAAACTGACACAAACTGAAAAAGATATATACAAGGAAAAACGTGGTATGTCTAGAGCAAAATAA

GTATTGTCATTTATCACTCTGGAAAACGMSWWACAGTAAATCWAWRCTAATTAGCTTARATTTACTTCTCATTGTAAAGT

GAGCTCTCATTCTCACTCGGAAAACAACAGKATCCATAAAAATGACTTGTTTAAGAAAATGCAACCTATARAAKCATTTA

GCAACGGTAAATTTCTGCATTGGCATGGCTTCCTATTAGACCGCAATATTTATTTATGCAAGGCACTTCGGTATTAGGGG

CTAATGACTCGTGTTGTGAACACTCCCGGCCAATGGCTGCTCGGCTAGCATTCGCAATAACTTCACACGCTGAAGTGTGG

TCACAAGGCCCGTACAGAGCATTTGCTTTTGAATGACACTACGCCACTATGTTCGCTCACCAGGACATCGCTACATACTA

ATAAGCTGGTTTGTGCACCGCACAGTACACGCATCCTGTCCAGGACAGGGACACCCGTCCACGCAGAGCACGGCAGTTTT

CG

>CK183890_bb No definition line found

TAGCTCCACAACGTAAAAATTTATTACACGAAGTAAACTGTACAAACAGTTTCAAGTAGCAGTCTGCTGTTAAGAATTAA

ATAGAGCGGCTCGCAACACGTTCTAAAACCTTCTCCACTAAACTCTTAAAATATATAAAACTAACCTGCAGGACCTCCTA

AAGTCTCGAGACTAGCCAGAGAATAAAGAAACTGACTGCTAAGTCACAACTTCGAGTAAAATAAAAAAAACAAATGATCT

CACTGGTTGTCCAAGAATGACTACAAATCAGCAGTCAACGAGTAGGGGAAGCCTCATGCTGTTCAAGATCAACGGAACAA

TGTCCAAGCAACTAAAACGACCCTGCAAGCAACTTACTGGATGAAGATGCTACCCACAGGCTAAAGCACAGCTAGCAGCC

ACTACCAGGAACACCAAMACAAAAAAAAAGTAAAGCAGGCTTTTATACAAAAACATGCAGGAGCTTGAAGGCACCGGGAG

GAGAAGCAAGCCAGATCCCGAAATCTCGACAACTGGACAGCGCTGCAACACTCAAAGTTGCCCCAAGGAAGAAGGAGGGG

GTGCCACAAGCAGGCAAGAGAGCCAGATCAGAGAAGGAGGAGTTTCCCCTCACCGCTCTTCCTCCTTGCGGCGCAGTGAA

GCATTGAGCTTTCTCTGGACTGCCGGGTTCGATATGACCTTGCGGGACGCTTGTGGAGAAGAGCCCTGCGCTATCACCAG

ATGACGCTGACCACCCAGAAGAATCTTTCTCTTGTCCTCAGTCTCGCTGCCTTCCTCACTAGATGACTCGGAGCTTGATT

CGGAGCTCGAC

>CK183925_bb No definition line found

CGAAGTTCGAGTTTCCACGTTGCCTCGAGTGAAACCAGTGAGGCCGTTGTCTCTAGTTGAGGAGCGCAGCCGAGCCTTCA

AGCAGCCTGGTTCGTCCGAACCCACGACTGCACCCGTTGTGAGCTTTGGCACGTGGAAGGACCAGAGAGAAGAACAGAGA

GTCACAGCATCTGTAAGCAGCAGTTTGAACCGCCAAAGGACTGTTGGTGGCTTAGAAGGTGCAGAAGAGAAGACAAGCAA

AGCAAACAAATGCGATGCCTCTGCAAAGACAGTAGTGAATTCAAACGTAAAGCCACAGGCGAAGCCCAAGCCAGACACGA

GGAAGCCCACGGGAATCCTACACGCAGCGCCTGTGGTGCGTGGATTTTCTCCAGAGGCCATCGAGAAGCTGTCGCTAGAG

GCTTCTCTTCAACAGAAGTTGCCACAATCTCAGGATCAGGCCACACCGCACCAGGAATCGCCAGTGGCTGCAAGTGTGCA

GCAGCTACAGTCTTTGAGGCCTTCCAGCAAGGGAAAGGTAACCGTGACCAGCCTGCACGCCAGCTCGGAAAGTTTAACAA

GCATTCAGCAAGGTAACGTCGCAGACCGGCGTTCACTTCCACCATACCTCCAAAGTGAGATTGTTCGCTTGGCAAAAAAA

ACCAAAGTGCGACATCAGAGCAGCACCTTCAAGCCAGCGAGAATGCCTCTTGCAGCAACAAACAAACCAAGCATAGTGCA

GGCCACACTAAAGTCTGGCATCACTGTGGAACCTATGGCTTCATTCACTCTGTCGTCAAAGTGAATGGGCATGCCAGCGC

TGT

>CK183949_bb No definition line found

TGCTTGTTTTTTCGCTTTGATCGAATTGATCTTGGGCAAATCTGTTATGTGCCGCATTCAGTTATCTTGGCGTTATAGCA

CAATTTGTTGTACACATGTATTTTCTCTTAACATAAATGTGCAGCAGCGTGCTGCCTCTCATCTGTGAGGTCATCTAAAA

ATGTTCTCAACTTTCTTCATCTGGCCCAGTGATAATGCGTTTAGCGCAAGTTAGCCTCCCTTACCAAGTGATGTTGTTTC

TTTTGGGTTTTAGGCTTTGTCTCAGCGTTTGCATACCAAATGTCCTATAACCTGCATGTGTCTTTTTGTACACCCAGAAA

TTTGAGCTCCCTGAATATAAAAGTCTATTGTCTTAGCTAATGCCGTTTGTATAGAGGGTTCTGCAAAGTAAAGAAAGAGC

ATTTCTACTTGCTAACTCTGCTTTGCCAGGTCAATGTATTATTTAGTCTCTTTAGATTGTCAAGCGCGTGTTGTATTGGA

GCTGTGGCATAAATATTAGCATGTTTTACTAACACTTCTTTTTCGTTAATCTGCAGCTTTGTTAAAAGCCCTATAGCTCC

AGTTTGGTGGTATCAGTGCAGCCATGTTCTCTATAGACTATTGCTTCTCGTATGTTATGCATTTGCACGTATGCCTCCCT

GTTGTTTAATTACTGTGATTAACTATGACGTTCGCTGCATAGCAGGTGATATGACTAGTAAAAAAGGCGTCTTTATTTTT

GTTCCTTTTTAAAGAGTGTGCATTGCATGAACATCAGCTCGTTAAACTTGTCGTGCTGTTGTGCATTGTAAGCACATTTG

TAGCAGATCTAAGTTAGTACTTGCTGTTGGAGTTTAGCCTCAGCCATGTAAAG

>CK183996_bb No definition line found

GGTATACAGTGATGTGCTGTTCACCGGATAAGCTGTGTGTGTATGCATGCGAAAAAATTACACCAAGGTATACCTACCGG

CTTTACAGGTGATGGATATTATTTCCCTTCCTTAAATGAGACCGCGACTATGCAGCCATCTCAATGCTGAACGTTTGTCT

TTATTCTTGCACCTGTTGTTATGGAAGCCAAGGAGATATCGACGAGGAAACCAAGTTTGGGCATAATGAAACTATACTCT

GTTCTTTTTGAGCTAAAGCCGTTCAGGGCAAGGCGTAACAGGATTGACTCAGCCGTAATCACAAGAATTATATGCTTTAA

GGCAGGACCGATTCAAACGATTTGATTCTCATGCTCATATGCATACACCATCTATGCTTCCATAACTGTATGCTTGCTGG

GGATATTGGAACCTAGTTACAGAAGTGGCGTGCTACATTTTCGTTATTTGAACTGCGCACACTTAGGSAAAATAGACCGC

RCATCAACCAAAACATCAACCAAACAATCAGCCTATGCGTCTTCCTATACGTGGAGCAACCTTCCCGCTGTGTCTGGCCA

AAAAGGAACCGCGTTTTCTTTTTCTTCTAAATTCCGGAAACATAAGTTTGAAGTCCCGATGGAGAGAAACAACATCGGGA

ATGATCAAACAAAGCAAACCTTATTCAGATAGACTTTTTTCAGCCGCCAGCTTTAATGAGAGTACGCTTCATACCACACC

GTCTCTCAKAAGTGGCGTTGCGCTGYTTGTAGGTATTTCCCACGCTATGATTTGTTCCGCACAAATTCCGCGAGTGATAA

ATATTCACTGCATTATCAGTGATGTTATATTTGTTAGCCATATAAAGTGATTTCTTGAACTGTAGGCATATCTCAACAAC

TTACTTTTGAAGAATTCATTGCTGAATAGCACTGAAATACTGACAGTGTCACTTTTGCTCTTGCACACTTTTTATTTAAC

TTTCAAAAGTGCTAACATGTTTCCAGAGCTTGGGACACTTTGAACAATGTATTTTAGATGTGTGCCGACCGGCCATTGAT

TATTTGTGACGAGCACTGTCG

>CK184007_bb No definition line found

GTGCTCAAGTGTGACGTGGGAGTTTTGCAACGCCGATACAACGAAATAGTCATAGAATTTACTGAGCCGATATATTGGCA

TTTCGCTGACTAGTTGACATCAATTAAACCTTGATGAGACTGATAAAACCAGTCAAATTATTCATCGCCTCATATCAAGA

AAAAAACAACAAAGACATTCTGTTGACTTGTTTGGCATAGTTTGACAGGTTTCAAACACCTCTTGGAATCCATCGTGCAT

TCTCATTTTGCTGGTGCTAAGTAACAAAAAAATTAGATATAAGGCTAAATCGCACAGTTTAAACTACGTAGAATTTAAAT

GTGCATGGATTTTGACATCACCAGCTATAGCAAACAGGAAAGGGAAAAATCAGCTGTCTTTATTTTTTGCAGAATGCAAC

TTCACTGATCAGGTGTTTGTCACCACATCACCTTGGCTGTACTTTGTCGCCACGGAAAAAGAAACGCCGTATATGCTCGT

GTGTGTAATGGATGCAAGGTTTTGTTGTTTGCAAGATTTGGGGTGAAAGTTGTAAGTGCAGCCATTGCACAAAGTAAAAT

GTCAGAATTCTTTTTTTCGTGTTTGTTTTTAGGTTCAACTTGCCTTCAGTATTTGCCTGTACTGGAAGAAGCAAAGAACA

ATCTTCATCATTTTCGTGTGGGTGCAAGGATGTCATTCCTAGCCCTCATCCTCCTCCCCTGCACATCGCATTCTCAAATT

CTCCTCTTCCCCT

>CK184053_bb No definition line found

AGTGCGCATAACACGCATACGAGCATATTTTCGAAAGTTAACCTTTCGCAAGAGGGTGAAATAAGGCCCGCACCGGGGTA

AAAAATTCGTTAATGCGGAATCGCTGTTCAAAAACATTTCGTTGCCGCGAGATACGTAATACATGGGCTTCTATGAACAT

TCGCCGGGGGTCCGGAATTATTTCGTTACCGCGGGGCTTTTGTACTCGCGGGCTTTCGTTATTGCGGTTTTCGACTGTAT

AGCGTTGTCAAATTGTCACATTGTGTACATAATGTAATCTTGACAGTGCATTTTTCATTTCAAAATTTTCTGCCCGCTAA

TAAAGTAACCCAGCCAGGTCACCAAGGGTACTAACAAACCAGGCATATGCACAAATTAGATGTCAGCCAAACACTCAAAA

GACCCTAGCATCATTTCTATGGCCGTCAGTTCTGCCCGTACCGATAATTTCAAGCCCAATCAGGCATACGTTATTTACAT

AAAGACATTACCAGCAGACTTGCAACAGCCAGCCTACCAGTGGCAAATTTCCTACATCGTGAAAGTTGTTAGACCCAAAA

AAGTGGCGGGTGTTGGCTGTTGTCTGTGAACCTTTTTTTTCTTAATAACTAGTTAAGTGCAGAGTTAACCAGAGTCCTTG

GTTGCAGCAAGACAAACCAAGTCGTCAATGAAGTTAATGAAGTGTATTCATGAAATATCTGTGATGGAGGCTAACATTTT

TGTTTGGTTCTACAAGCTGGCATCATTATTGTGCAAGATAGTGAACACCCTACTTGCCAGATCGCCAAAACATTGCCATT

ATCAACAGAGCTTATACCTGCATACGAAGATAGTCCTGATGTTCTGGCCAGATTAACTATCAAACTTGCCAATGTTCCA

>CK184054_bb No definition line found

TCAAGACCTTTACCATAACCTTACTGTCCCATTTGCACATGCTCCATCTCCATGAGTAGGCGTGAACGCGTATTGAAATG

GTTACACCACTCTTTGTCCTTGTGTGGATTTCTCATGTCACTCATTTTAAAGCACTAGGCGTTACACTTTGAAGCCCGCC

GCTGCCATTTTTAGTCTCTTACATATTCTTGTGCATTGGCAAGTATAGAATAGCAGGTGTCTTAGTGACCAGCGTCATAT

CATCGTGTTGCCACAGCTTTACAGAAAAAAAAAGAGCTGTGATAGCAAAAATAAAAAGATTTGTGGCATGTATATGTGCT

TCCTGGACAAGTTGTTACACGTGGTTAGTTCATGATATGTCCGATTTCTATATCTTTGACGGTCTAAAACAAAACAAAAA

TTTGCGCTGATTCCGCACAATCAGCTGATGTATTAACAGCAAAAGATACAGCACAAGGGGGGTGAAAGTGTTGGTTAGAG

AAGGTGGTCGAGACAGCAGAGGCATTGAACCTCCCATGACTGAAGTGTCCGATGGATTCGACGGCGGCCAAGTGGTTCTC

TTTTGCACTCTGCGTATTATAGATGCTATATACTTGCCAACCTTCATGATTTCATAAAACAAAAGATGTCAGCTTTAGAG

CTTTTTTACTATTAGCTTCACTTGCACTTGGTCTGAGGCTAAGCCCATGAAGAAGCATTATAGCTAAAGCCACTAAGCAA

TTTTTTAGTGCCAAATTTCAGACACTCCTTGATTCGGCATTTGGGCATAGCCATCTGCACATTTTATGCCTGTTAGATCA

CCAACGAAGTCCCGAGAAGCAACCAGTGTTGGAAGTTTTTGTGCTCGCTGCTGACAGACAAATGCATAGTAATACCTACG

AGTGACACTTGAATGTTTTGCTCATTGTGGTATTTGTTTTTTTAAGGTGAGCTTGCATGATATTGTTGCACATGAACATT

GTTGTTTTCTTAAGCACAGAACCTTTTGCCCTTCACTGCTGACAGTGCTGATGGAACATTGGGCAGTTTGATAGTTAATC

TGGCCAGAACATCAGGACTATCTTCGTATGCAGGTATAAGCTCTGTTGATAATGG

>CK184239_bb No definition line found

AACTGCAACTGCTGGTTTATTTCTCAAAGGCAGACTTCTTATCGTGCACATTTTCATGCATGTGCTCACCAAAAGAAAAA

AAAAAGCACTTAATGAAAAGAGCACCTTTGACTTGAATCCTGGAGTCAAATCACGGACTACTCATTAACAAAAAATGGCA

CCGTATATGGAAACAAATACGAAACAGATAGAAAATTCATAAACGTGGAGTGTCACTAGTGGCGACATTTCAACAAGCGA

ATTTGTCTTCAAGGCTAGCAGCATTGACAAGTCCACTTGTCAAAATGGCAATGCCCCTTGATCACAAGTTTTTCAGCAAG

TCCATCAGTACCTTGAGACTGAACAATCAATCATTCATAGAATTTTTCAATTTGAAACAAAGAAAGATGGAGCAACCACT

ACATGCTACTTCATCAAACAGCCTGACAAGGCAACTCTCAAATAGCCACATAACTACTGAAGGTAGATCAAGAAATAAGT

ACGCTTGCACTTAGCAAAGTGACTAATGACTTGAGCGAATTTAACAGGCCCATAAGCACCATATTTACGCAGGGTACAAC

TAGACAAATCCAACAAGATGCTCCTTCTGTGAGTCTATGTAAARTCTGTGCTRTTTTTGTCTTATAATGGCTCAGCCATG

CAATGYTATTTGTCTAACTCATGGAAATTTCATTCAGCATATCTGYGTCTATACACTAAATGKGTAAGCATGCACGTKCA

TCCACACTTAGTGTTGGTGAAATTAGGAATATTTTAGTGCTGTACAAGTG

>CK184293_bb No definition line found

TCCACACCGCCTACATCATTTGACAGGTGTAGATTCATAAAGGTCTCTCTTAGGAACAGCCATCATTCTGTACTCGCGCA

AAAGCTCTATCAGCACAACACCAATTAACAAAAGGTATTCTACATAAAGACAAAGGAATGTCTGGAATGACAATAGCAGC

AGACTAACAAAAAATTAACAGTGCATTTATCAAAAGCAACATCATTTGGGCCAAAATGATGCTTGCCAGAAGTTTTAGCT

GGCATTTATGCTAGTTCATTGAGGTATCAAAACAGTCACGGAAAGCACGCCTAACAGCAAAAGATTAGGGAATGATGGTT

TTACAACAAATGGGAATATCTGCTCATTTAGTGCGTAATGCTATTACTGTAGGTCCTGACTGTATATGTTGCGCACTAAC

TACAGACTGCAACATTTATATTTGGAGGTTAGGTTGCCAGAAAAACCGAGAATATAACTTTTTAGAAAATTTTGCATCCC

GCAGATCTTGAATATTGGGTGTACTTTTGTCTTAAAAAGTTTTGTGCTAAATGCCATAATTGTAATCAAAATCTATCGAC

CACATTTTTTACGATAGCTCTTATCCCCTTGATGATTAAGCTACCAAAGTTCAGATTCTTTGTCAGCTGAAGGCACTTCG

ATCACACGTGGGGAGTCTGTTAGCCTCGCCACTCGTTCCCCTTCGACAACATCGCCAATTATCCAAGCCGGGTAGCCCTC

AAGCCGCTCGATTTCTTCACAGTACGCCTGAGCCTTCTCTT

>CK184340_bb No definition line found

GATCAAGACATTTGGGAAACAAATGGAAAACCTACAAAAGAAGATGGATGAAAAAGAAGAACAGATACAACAATGCAAAG

TGGACATCAAGCAGCTCAAATCTGACTACCATAACTCTCGGTCAGAGAAAGACAAACAGCTATTGGATCGGAAGAAAAAA

CGCTTGCAGCAGCTGGAAGACCAAATGAAGAAGCTGGAGGTACAAGCCATGGATAAAGAAGAAAACAAGCAGATTGCTCT

GGGCACCTCCAAGCTCAACTATCTTGACCCGAGGATTAGTGTTGCCTGGTGCAAGAAGTGGAATGTCCCTATTGACAAGA

TCTACAACAAGACWCAGAGGGACAAGTTTCGTTGGGCCATAGAGATGGSRGSTCCCGATTACTAAGTTTTGAGCAGAGGC

CTGTCTAGTAGCCACTATCATGTTGGTCCCATTGGGACTGAAGTGACAAGCAGCTCCTGCCCTCCTAGGTTTGAAGTGCT

GGCCAAGCCAGACATTGGGTTCCCCCACAGTTCAACTCACTTAGGCACCACCAGAGGGGAAAGAGCAGAGGGGGGCAGAG

CCCTTCTTCCCACTCCTGTTCCCACCAGCCATCTTCTCACTGTCCCATAGCTGTGGAAAGGGAAACTGCACCATAGCCAG

ATTACTTCATTTCAGGTGTGTCTGTCTGCTCAACGTGATGTTGTTTGTTTGACCTCACTATCTTTATTCCCACCCTTCAT

GTGACCATTGCCTCTTGTTTTTCTTCTCTTGCTTGCTGTGTGTGTTATGAGGGAATAGAACCTCACTATCTTTATTCCCA

CCCTTCATGTGACCATTGCCTTTTGTTTTTCTTCTCTTTGCTGCTGTGTGGTATCAGGGGAATAGAG

>CK184371_bb No definition line found

CGCGTTGGTCGTGCTGAGCGCCATTAAGATGGAAATGGTGGTGCAGGCACCCGTGGCCGGCAAGGTGAAGAAGATCTACA

TCACCAAGGACATGAAACTTCAGGGTGACGACCTTTTGCTTGAGATCGAGGAGGCTTCGTGATCTCGTTTATTGGAACGA

AGAGCCCCTTCTGTTTCTCAAAGTCCATGTCTATTGTTGGAGCAACTTCAGCGATGAATCCCTCACCCGAGCCAACATTT

CCGATAGGCCATGCGCTCGCAGTTCAGAAGTCCACATGCGAGCATTGGCCAATAATGACTTCCAAGAGAGGAACCAACCT

GAATGTTTAATTTCCGGCACCTTTCCATTGTTGTTCATTTATACCATGTTCATTTCTTCTTTCCTTCCACCCCGAGACAT

GGGATATCGATTACAAAGGCGCGCGTTTATGATGTGATGTGTTTCTTTAAGGTTTTCGGCTTCTAGGGGTGCCCTCGCCT

TTCAGGAGCATTGAATGGGTGTCACATATCAACCAAAGCGCCCTAGTCATCTGATGCAACGTGCTTGCTCATTGTGCTCG

ATGAACATAAAGCAGAGCCAACATTCAGCTAAGCAACACACGAGGCTAGTTTGACCACATTCTGTTTTCTCGTGCTTTCA

ACGAGGAACAGAATAATTGAAGAGCGACGCAGTGAAAGTGCAAACTATTAGCTGATTTTATTCGAAGTGAGTGTTTATAC

ACGGTGATCGGTGGGTGGCARRAGTAGCTTCGCGATTTGTTAAGAATTATAAAAAAAATTATAAAGACGAAACRAGCCTG

GGAGTCCTGTTGGCTAGCCCTTAGGTTAGTCTAATGCGCTATTCACGGTTTTGTAGGAATTGAACTTCCTGTCATTGTCT

GTTAGACATACTCTSTGAATTGTGCACAAATTTGCACAGTGGCAGACACGTGTTCTTAATTTT

>CK184374_bb No definition line found

CCAGTCGCCGTCTTCGAAATGATTCACCAGAATTTAGTCCACTCCACTTCATGCCAAGGGGAACTCRGTCAATGGACGCC

ACCACCATGACAAGTCATGTAACACCRGCCCAAGTGGWGATAAATCARCCTCACCAGCCACCAGTATTTCATGGCGACTC

GTATGAAGACGTAGAAGATTGGCTGAACCTMTTTGAGAGAGTGGCAMGTTTGAACGGTTGGGACGAAAGAGAGAAGCTCC

GCCGCGTATACTTTGCTTTGGCGGACTTCGCAAAGACGTGGTATGAGAACCATGAAACCTCCTTTACAACCTCGGATGAA

TTTCGGCGACAAGCTCTGGCGACTCGTGCCAGCGCGGATCGCAAAAAAAAGGCGCAGGTTGCGCTCGAATCCAGGAACCA

ACTCACCAACAAAAGTGTCGCAATGTATATCGAGGACATGATCCGCCTGTTCAAGCGCGCAAATTCCTATATGACTGAAA

ACAAGAAGCTACCCCCCCTCATGCATGGGGTGAAGCAAGAGCTATTCGCTGTTCTCTTCCGCAACCCACCACGAACAGTT

GCGGAGTTTCATACGGAAGCGACAGCCATCGAGAAAACGCTAGAACAGCGGGCTCGACAGTACAA

>CK184417_bb No definition line found

CTCGGACATTCGTCTTCCTGCGGCCCTCGACATCCGTGCCCTGGTCCGCGACATCGTTCGTGAGGAGCTTCATGGGCGCT

GCTCTTCCTGCGCCCCACCGTCTACTTTAGCCTCAACTCCAACCAACTTGCGGGACATCATTAGGCAAGAGATTGCATCT

GCGTCACATTCACCTTGTTCCGCCGAATCCAGACCTCGTCAGGTACCAACGTACGCAGAAGTCGCGGCGCTCTCAGCACC

CACCACTATTCCAGCGGCTACACCAGGTGGTCATGTACCTGTCGCGTCAGTGTCACCACCGATGCGCCCACAGCCGTATT

ACGCCATGCCGCGTCCTCAACGCCCAATTTGTTATTATTGCGGCTATAAGGGTCATATATCTCGCTTTTGCCGAAGGCGT

CAACAGGACGAGCAACGTGGCTACGCTCCATAGGAAAGCGGACGCTTCAGTTCACTCACGAACTACCTGCGCACGCCTTA

CCGGTCTTCTTTTCAGCGTTCGCCCTCTCCACCTTACAGTAGTGGTCTGCCTGTGAACTCCCGTGAGTCTCGCCGCCGCT

CACCATCTCCCGATCACCGTTCCATGTCGCCTCTGCGACCCGCCTCCAATGTTCGACTGGGCGACAGGACCTATAAAAGT

CATTGCTTTCGACATCTTAGGTATTGATACAGCGATGAACTCTGCACATTCACTGGGTCATATACTGAAGGATCTGGTAT

TGTTTTATCTGCAGCAAACTACATGATGGACTGCGCAATGAGGTCGCATATCCGCCCTTATTGACGGTAACAAAGTTTCA

TGTGTCAACCCCCCCCCCCCCCCACCCAAGTGGCAGCCTGAACGTAACCCTACATTGTCAACTTCTATTTACAATCTTAC

CAGCCATCCCAACTGCTTCATACCATACTTCCGCTCACTTTAGTATCAACAAAATATCCAGAAACTGTACGAGTAACTTC

AGTGACTTTGATACTTTTCTGACCTCATGAATAGACC

>CK184462_bb No definition line found

ATGACAAGCTTACTGCAAGTTGCCCCTTCATAAAACTTAGCTCAAACCATGACAAAGCCATACAAAGATTAAAYATTTCT

TACTTTGTAACACACTGCACTTATTTRTTTAATTCTATGCARAAATGAGGGGACAGGGATGRATATCCTTGTYTTACCGT

TTATGGCACTWGTAAGTGGAAGTGCAGGTTATATATATACATGGTAGTGCATCAATATACGGTTGYATTAACCAAAAATG

CAAGTCACATGAGCAAATTTAATTAGTACAAAAGCATGATTTGAGTAGCGATTGCATGTGGTTTGGACTTGCACAGCTTT

CTTAACATGAACAAAGCAAAACTTGAATAAGTATGCTTCACAATGACACAACA

>CK184557_bb No definition line found

TTCGCAATCTTGATACGTACAGTGCACCGCTCTCAAGAGGTCGCTACCGGGCAAYGTGCAATTGCTCACCCTTGAACACT

GGAACAAGGGAGACCAGGTTCTGCTTCGGCTGGAACACTTTTTTGAGAAGAATGACCAAGCGGGAGAATTCTCGAAGCCG

GTCAACTTTAGCTTACAGGCTGCTTTTGTGCGGACGATAGAGGACATGACGGAGATGAACCTGGTTGCCACAGAAACGAA

AGGGAACACCAGGCGTTTCGAGTTCGAAACTGAAGGCAGCCTCGAAGCGAAGGGCCTCATGTCAGGCCACGAAAATGGGT

CAATGGATGTGCATGGGCCAGAATACTATGTGTACCTGACACCGATGCAAATCAGGACGTTCCTGGTGACTTTCTCTAAG

GACGACACGAAGCACATGGTGTGTTCCACCCACTGAAAAGCAGCTGCCATCAAGTGTCTACACGAATGAAGCTCTGGCTT

TCATGCCCAAGTATTATTACTCGTATGCATTCGAAAAGAATAATTTTTTCCGAATAAATTTGAAAAGAATAAACACTCCT

RTGGTGCAATGMTTGTGCCTAGAGAAGCMTTTACTCATGCGATACTCGGCGTTTTGCATCGTACTTTGTTGTGAATATGT

GTGCTCGCAGGGTTAATTCAGAAACTTGTTGCATGAAATATTTTACTAATAAGTATTCATTTATTTATTTGTCTGTTTAT

ATATGTATTCATTTATTTGTTGATTTGTTCATTTAAGGAAGACTTGACGTTTCGGGAAAAAAGAAGAATAAGAGC

>CK184600_bb No definition line found

GCAGAGTTTGCTGCTGTTTATTCAGAAAGCAAGCTTTACAATCAATGAACACATCTGCCACACAGAAAAATATACTTTTT

TTATGCACATAACGGCTCAGCTCATAGCAAGTAAGAGCTGACCTAGAGGTATCAACTACAACAATAAGCTGAATGCCACT

TCAGTTGTGATACATGTTTCTCCACTATCTCTGCATTAACGCACCAATACAATTTTGCAGCAAGAGACAACAAGAGAGCA

GCCAAGAAAGCCCAAGCCAAAACACACTTTCCTACATAAAAAAAGCCTGCAAGCACAAAATGAACGAGAAACGCTTAAAT

CGTAGCTTTGCTCCACACAGAATCCTCATGCATAAGCAAACATGCAAAAAAGTCGCATACATTTGATCTATTCATGTATG

TCAAGTTCCAATTCCGTTAAAAATAGAAATGAATACAGCGAAACAAACTCTTGCATATTTAACACTAGCTGCAATAAGAA

TGAAAATGGGCTTAGACACCTGAAATAGCTGCTTAACTTCGGCTGCATCCACTGAGACTAACAAGCACGAGCTTTCAAAG

TTTTATGTTTTTTTAGTGTCTCCAAAGGTGCCATGACTGAATAGAACAAGGGGTATAAAAAAAAAAAAAACTACTCAATG

GGAAAGGCATGAACACTCAGCAGGGAAATGCTTCAAAAGAARAAAAAAAAAAGCAACTACATGTGTGTACTTTCCAG

>CK184601_bb No definition line found

CTTAAGTTTCATGTGCCCTAAAGTTTTGTAGGTGAGCATTAGTACATGACACAAGGTTGGCTGCTATTTTATTTTATCAC

ACTTGACTTGAATTCCAAAAATGTTGCTTTAAAAGAGTGTCTTTGCCTGAGCATACTTTTCTTTCTCCCATGCCATTATA

ATATACTGTATGATATGCTTTATTGTGGTTAATTGTGTATTGACTGTTTTATTGCTTTGACTTGTCCCTTCCCTTTCTTG

AAATTTTTTGTTTTGATAATTGACTGACCTGCACTTTCYTGCACTTCATGAAGGATGGTTTGAAATATTACATGTGTGAT

TTATATCCTGTGTGGAATTTTCAACTTAATTTTTTTTCTTTTGTTTCTGTTTTATAGTGGGTAGAAGTTGGAGGGAGTGG

GGAAGCTGATTCAGTAAGCTTGCTTCTCCTAATTGCATACTTATGCTTGCTCCTCTTTGAAGGATTCTTCCTTCTTTCAT

TTTTCTCATCACTGGCATGCTTATTTGTTTTGTTTTGAAAAGCTTGTCTTCGTATTCTGCATTTTTTTTTCTTGTTGCTT

ATTTTCGTTGGTCTTAGTTAGTTTTGCATTTGCACTTGCTTTCCCAGAGTCCTTTACCAAAGGGAATTCGCTCTAAATCT

CGAATTTTATAAGCTTTTCAGCTTTATGAA

>CK184614_bb No definition line found

GTCTTCCAGTTCATTGTGGATGTCGTGCTGTTTCACGACTTTTCTCTTACAAACTTGCTTCAGATGATGAAGATGGCCAT

GTTTGAACACTTTTACCACAATCCCTCTTCTCTCTTCTGCTGCCGCCAAGAAGAGCTGAAGGATGTGCTTCAGTGCATGT

CGCAGATGGATATGAACGATGTCCTTGGCCAACCATCATACCAGAGGTATTTGATGGAGCAGAAGCCTTCTTCGGAAAAA

GCGGCTAAGAGAATTTGTAGACTGGTGAATGATTTGCACAGCCACCACAAAAATAGCCTGCACTTGCTACACGTGTTGTA

CAGCTTTGCCAAAAAGCTCCCCAGCTGCTCCCTTGGAAACCATTTTAGGGAAGCGTACATGACATTTCTGCAGGCACCAG

TGTCTGAGACTGAAGATTTCAGCAAGTTGGTGAAGCTGATACGGGTAATGTCCATTGATGAGTTACAGAAGAGAATTGGT

GATGCCTTGTTAGCTTTAGAGTCCCAGACAGACCAGTCGAAGACTCCCACAAACGTTGCTGATTTGAAAGTCCACCTTGA

GGGATACCGTGAAAAATTCAAAGCTTTGACAGATGACGTTCCTGAAGCACAGGATTCAAGTGAAACACCTGAACCGGTTG

TCTTGGATTGGGGGAAGCTGAGGAGCAGGTCACAGTTCCAAGAGAAACTGAAGAACCTTACGAAGACAAAACGAGTGTCT

CCATTTGAAGCCTTGAGAGATGAATTTGCAAGCTTCTTTGCTGATGCCTTTGGTGATCTAAAGCCGCCATCAAGCATGCC

TCTTCACGAAGTGCTCTACTATAAWGATGCGGTGGCACTGAAACAGTACTTCACACCATCTCCTAGGACAGTGCTGCATG

CTGCATTGACCAAGCCTCAAACAGTACTGAGGGTAAGATCTACATTTTTTTTGTGATCTTTAATCCATTCCACTCATTAG

ACAAACTACCCTTGCCGGCACAGCCTTATTGAATATTATATCGATAAAGAGATGTACTTATCGAGTACATTTCTATCTAC

CAT

>CK184639_bb No definition line found

CTAATCACTCGCGGTATCCTCCCATCAGCCTTGTTAGAAAGCGAATTGTGGTGGAGAGGACCACACTGGCTTCACAAGGA

TAAGACGCATTGGCCAACGACCGGTGAGCAGAGCCCAGGGGCAGTAGAATGTCACCTGGAAGAGCGAAAAGTGACGGTGA

TGCCCGTAATATCATCGCCGTTCGAGGCRGTGCTGAAAGTAGAGGAGTTCAGCTCATGCAGCAGAGTCGTGCGCTTAACT

GCGTGGGTCCGCCGCTTTGTCAACAATTGCCGCCGCGGGAAAGAAAGGAAAGGTGGTCCGCTACGAGCTGAAGAGGTGAT

CGATGCTGAGAGGTACTGGTTGGCAACAACTCAAGGAGAAGCATTCAGTGACGACATTTCCAACTTGGAAGCCCAAAGAC

CACTGCACAAAGGCTCTCCTGTTCTGCCACTCAGTCCGTACCTTGACGGGGAAGGTCTCATGCGAGTTGGTGGACGCTTG

CAATTCACCGACAACCACGAAGAGACTAAACATCCCATCATTCTACCTAGCACTCATCCCTTCACGCTACTGCTCATAAG

GAAAGAGCACGTGAGAATGCTACACTCAGGGGTGCGCGACACCTTGGCGTCATTGCGAGAGTCGTATTGGATCATCCGAG

GGGCGCCAGGCCGTAAAARAGGTTTATCAAGCAGTGCCTCATCTGTCGCAAACAAAGTTGCCCTCAAGCCACGGAACCAG

TGGCACCACTTCCAGCTGACAGAGTAACAGAAGGAAATCCGTTCGACACTGTCGGCATCGATTTCGCAGGACCTTTGATT

TGTCAAGAGTCGCGCGGTGCCCGAAAATGTTACATCGCAATTTTAACCTGCGCTGTGACACGTGCCGTCCATCTTGAGCT

CGTTAGCGACATGTCGACTACAGCCTTTCTCCTGGYWTTCAAGCGCTTCGTGGCTCGCAGGGGAATCGGTTCGACTATTT

ATTCAGACAACGCTCTAACGTTCAAGAGAGCAGTTAAGGATCTGAGAGCAATGTTCGCGCTGCTAAAATCAGAGAAAATA

CAGTCATACTTCACCGAAAAACAGATCAGATGGAAGTTTATTGTTGAAAGGGCAGCTGGTGGGGCGTATTCTT

>CK184672_bb No definition line found

GCGCGTGTTTTATATCTAAGCACGCCGAATGTGTGGGTGCTGGATTTCGTTCGTCGCGTTCGTTATTCCATTCTCAAAAA

TGTTGTGATCACTTCTACTGTGTACGGCAGCTCGCTCAGCTTGCTTTTAATGATATTAGTATATGAAGAAGTGCACGATT

ATTGATTTGGAAATTTGATCTCGTATATGCTGGCTTTTCTGCCGTGTGCAAGTGGAGCACCTGCAGCGCAGAGATTCGCT

CGTTGTAACTGTTCTTACGAACACTGTTWTTTGTGTTCGCACGGAAGTTGTGTCATGRAGGTTCAAGAACGTCATCCACG

CTCCACTSTTTGCACACCCCTGTATCTATATTGACTGAATATGTATTTTTGACAATTTTATGGGTGGCTGTCATAATATG

TATTAGCCGCTCCGATCTCATTGCCTTTGTGATAGTAAATAAAAAAACTGCGATTGTTTCTCGCCMTTTTTGTGTCGTCT

TTAATAAAGTACTGTATGCCGCTTTGCCTCGTCACAAGTTACCGGCAAAATTTCTGACAAAAGATTAGGAGCGAGCGTCA

TGGAACCATCTTGACCTTGAATCCTCGCCCTAAAGAATTGAGGGACAAGTAGGCGGTGTTCTCTGCCTTGATCACGAAAT

AGA

>CK184688_bb No definition line found

ACTGAATGCAGCAACGCAATACCTCATGCCATGTATTCATATCTAAACTTTTAAGCGCTCTTGGACGCTGAGTAGGTATT

GAAGAAAACACACTTTGTGCATTTGCGGCAGTAGCAAGGACAAGCGTCCTAACATAACCTAACCTAATCGAGTTGCACGT

GGCCGTGGCTGCAGATAGTCAGGCCAGATTGGCCAAGTGCTTATTGGAATAAGACGGCTGTCTCAGARATCGCAAAACTA

GGGACAGGCCATCGTTATTTCATGTTTATTTCTTTTACGAGTCTRATAGACCATTCCCATGTACCGAAACTTTGTTCAAT

CAAACCACTAACCTTTGTTATGACAGGTTCCGAGACGTTCCCGAAGTACTCTTCCAAATTGAAGAATCGCTTCTAGAGAC

GGCCAGKGAATCAGC

>CK184689_bb No definition line found

GATGTTAGACAAGATCAAGCGTGGAACTGTCACTTCCAGTCGAAAAGCTTTTGTGGAAACAAAAGATTCACAAAAACCAG

ACAAACTATACCAGTTACAAGCTAATATTGGTGCAATTTTACGTAAAGTGTGCGACAAAGCTTCATTCGGGCATTAGTTG

ACAAGTTCGATAACGAACGTGCATGTCTTTGGATACAATGGGGGCTGTTCTACGTGGAACACACAGGTTSTRGAATGAYC

AGCTGTGACTGAGCYAAAGTTGTCTCTCRAGCCCCTACTATTGAGGAACATRCCAATCTTTTGTTCGTCCACTGCTGATA

ATTTCAAGTTTACATCTTCACTCGAACATCAAAGTTCTGTAATTGRAAGGYTCAGTGACATTTCCAGTAGTTTGCCGCCT

GTTCGTTCGAGCAGTCTGAACAGTTCTTGTGTCATATTGTAGGGGCTACGACAGGAAGSAATTYAACTCCCATACCTGAG

TGCAAGAAATTAATGCGAAGAGAAACATTTGCGGTTCCAGAGTGGACGCCGAACTATTTTTGAATTTCGATAATTCATTC

ATTGTTATATCTGTCACGTGTATGMGCACGCGAATAACGCCAGGCTAACAAGATGAAGATATACGCACACAATGAAACAC

GATATAAAACTTGGAATTCTTTACATCGCAATGATGCTTAGCTGGAAAAGTATGGTCGATAAACTTTTTATTATGCTACA

ATCTGAGATTTTTCATGTTTTACGTGGTTATTTTAATTAGATGCACTTGAATTTGTAGATTCGTACGTGCATTSCCYGAA

TTTGTGAAAAGGCTTCCGAATTCTGWTGTCTGAAACAAAGTGGTAAGCRCGAAAAKYATGAAAAATCTCTGATTGTAGCA

TAATAAAAAGTTTATCGATCATACTTTTTCAGCTAAGCATCATTGCGATGTAAAGAATTCCAAGTTTTATATCGTGTTTC

ATTGTGTGCGTATATCTTCATCTTGTTAGCC

>CK184722_bb No definition line found

TCACTTTGCACTGCATTTATTGGAATACCAACAGACTGGACCACCATCATAAACAGCAAAAAAAAAAGAGAAAAAACCTT

TACAATTAATGAAAAATGTACACAATTAGTGCCCTTGATGCTTCATAGATAACACATGACATTACACCATAGTAATGCTT

TTACCTTGAAACAGTTTACGGCAAAATGAATGGGGCAACAGARATTAAGATGTTGTCTATGATGAACAGTTGAAAACGAT

GTTTCTAATACCCTGGCACAAGCATACTGACTAAGGCACAGTTATTCGTGGATGTTTTAGAAAGCTCGATGTATCACAGA

ACTTCTACAACAACTACTACCAATAAAAAATTCATCAGCACAAGTGCTTCTATTACCAATAATAACAAATAAGCAAATCA

AACCTATCAATTCACTTCCAGTACATCACCACCTGTACCTAAATGCTTATGATTTTGATATGTTTGGTGTAAGCTATGAA

ACACCATCGGCACACAATATTTACAGAACACAGGAATTTGTTTAGTGAGAAAGTGTACTTGCAGAAGTGATCATGTTGTT

AAATGTGGTGAAACGCTTAATGGAACTACTAAGTGCTTATATACAACACTACAATGAAAGAGCTAATTCATAACAATAGG

CAATAAAGATAGAGCTCCAAAAAAACTTGAGTGTACAGAACATAGAGGCTGGTCATGATACATAAAAACTGACTGCTGGT

CATGATAAGATCTTATCGCAATTGAACATGATATGATCGAGCACACTACTTTCAAGATGCAATTTGCAGTGCCC

>CK184872_bb No definition line found

GTTTTCAACGCATTTTATGTTGCGAATGACGAGATAAAAGTTCTTTTATCTACAATAATGATACAACATTTGTACTACAG

ATATACCAAACCTCTTTGTCCACGAAATATATTTCGAGTGGGCATAAGGGGCAACACGATTGTAACACTAGTATTTGAGG

AAGCAATGTGTAACTTTCAAAAGGCTGCCTGAGGCTTTGTCGGGCACATGGTTCAGCTGGAGAACACCAAACAGCTGCAC

AACATGCATGGCATACTCGACGCTACATTGGCTTTTCCCAGTGGGACGAGGTCAAAGAACAGAAAAAGGACACTGTGCAG

CTGAGGTGAAGCTCATGTGTATGGGATGCACATACTTTGGATATTTCTTCACATACTAAGCACTGGAAGCTCAGTCTGTG

GTTTCAGAAAAATAAAGTGAGAACATAAGAACATCTGCCTACTTAGATAAGGCAAAAGAAGCGTGCATGAATACACTTTC

AACAATAAATCACCTATACAATTTTCTTCCCAAATTTAAGAACTGGAGAAGACAATGAGCACCATCTCAGAAAATGTAGA

GCAAACTGCAGAACATTCAAGAGAATGAACATATAAAAATTACTAGTTGGAGGAACTTGAAAACCTTACCTGGAATACAT

GTAAATGACAGAGTAACGCACATTTCAATTTGCAGGAAGATGTAGAGGCTGAAAAAAAAACTTCATTTTTTGCATAAAAT

TCAGTACTTTCCAGACACTTGCAATTTGGATGCACTGTATAATCCAGATGAGATTGAATAACCTTTCAAGTCGGGAATAT

TCTCCTCTAAGTATGCATAATTACTACCTCCGATCAGCAACTGCATTTGGTCACGACTGCT

>CK184874_bb No definition line found

ATACTTCGAAGGTTCGAAGGCATGCAACATAGCTGGAATAATCGGTTGAAGTTCAATAGTTCTGTAGAAACCTGGCATGG

GAAAAATTTTTCCCGCTTTTAGTCTTTTTTTTTCTGCGCTGAATGTCAAGTTATTTTTTTTTTTTTTCAGGTGGACATGA

TCTTCGCTTCTTTTATTAGAAGTGCTAACGGTGTGAAGGAAATTCGACAAATCCTGGGTGAGAAAGGCAAGGACATCAAG

ATCATGTGCAAGATCGAAAACGATGAGGGTGTTAACAATTTTGATGAAATCCTTGATGTGGCTGATGGCGTTATGGTGTC

CAGGGGGGACCTGGGAATCAATATTCCAGCTGAGAAAGTTTTCCTGGCTCAGAAAATGATGATTGCTAAGTGCCAGATGC

TTGGCAAGCCAGTCATTTGTGCTACTCAGATGTTGGAGAGCATGACCCACCTGCCACGGCCCACCCGAGCCGAGGCATCC

GACGTGGCCAATGCAGTTCTGGACGGAGCCGACTGCGTAATGCTCTCCGGCGAGACCGCCAAGGGAGACTACCCTCTGGA

AGTGGTGACCATGATGCACAAGATCTGCGTCGAAGCAGAGTCCGCCTTCTACCAGAAGGATGTCTTCGTCCACCTGTCTC

ACATTGCTCCCTGCCCCACGGATGGCACCCACACCATTGCCATTGCTGCTGTCACGGCATCCATCAAGTGCCTCGCCGCT

GCAATCGTTGTCATCACTACTACTGGCAGGACTGCCCACCTGATTGCCAAGTACCGACCCCGGTGCCCCATCCTGGCAAT

CTCTCGAATGGAGCAGACCGTTCGCCAGGCCCATCTCTACAGGGGTATTCTGCCATTGCTCTACCAGGGTGAGCGCCAGG

CTGACTGGCCTGCTGACGTTGATGCTCGCATTGAGTACGCCCTCGAGGTTGGCAAGCTGCGTGGTTTTCTGCGCAAGGAT

GACGCCGTCATCGTCGTGACCGGTTGGCGCAAGGGCGCCGGGGCCACCAACACCCTGCGTGTTGTGTACGTCTAAGGCGG

CCCCCACCACCAGCCAGTGCAATGCGTATATTGCGCTGCGCAATCGCAGTTGCCTGTCGGGAGGTGTGAAGCCGCT

>CK184911_bb No definition line found

ATCAGATAGTACAGGCGTAGTAACCTCCTTATACATTCCAAAGCCAAGCGTAGCCAACCCGCCTCCTTTCAGCCATTACT

TTCTCCTCCTCTCTCCCAAATTTGCACTCCCCCCCCCCTCTTTTATTAGCCAAACTTTCGTAGCTTGAGAACCGCGGCTG

TGTTTGTATCCGTATTGCACAACAACCGACCAACAGGAAAAAAAAATGAATGCTGTAAACGCGTTTGTCTGTAGTGTCAT

ATACAAAGATATAGTGTTGCGTGTAAAAACCCAGCTCTTTCGGAGCGTTTCCAGGAGTTAGCGTACATGTTACATGATTA

CCATTAAATTTTCTGTGTTTCAAGGAGAACAAAAAAAGACACAGTTCGCCAATGACGTAGATGTACGTATCGCATATATG

TGCTGATGTACAAACTATTTTGGAGCACCCTTGTGAAAAGAAAAAAAAAAGGACTCTATTTTTGACCTCTTCCGGGGCGT

TTGTGCATAAGCGTCATGCCTCTGTTATTGCTGTGTACGCGTGAGTGCTTCTGTTGAAAAAAAATATATATGTTTTCACT

CCCAGAATCCTTGCGCGGAGTTCAACACTGCACGCTAGGAGCAAGATSAMCATGTGATACAGARATTTATCRAGCGTTGT

CAYTTTAGAACGTAGCTTATAGCAGTACTTGCCGAGTTTAGAAGGAGAGATATATGCGATATTGGAAAMGTTTGTGAGCG

ARTCGTTGTGGAAGCTTGTTATCTGCGTCGTGARCCCACTTGAGGACGCGTRAACAAATTCATCCRTGCAATTAGCAGTG

TACGCGTCTGGCAACCCAGTGCCAACAAAGTTAGTGCTGTACAAGTTAACCCTGGRTGATATGATACTAGTACTGATGCG

GGAAGTAAGACTACCAACCGCTTCAATACTTCATCTGAATTCTCGCTTTTATGCA

>CK184914_bb No definition line found

CACTTGTGACAACCTTATTGCGAAAGTACCGAGGTACTAGCCATAGCCTTCCTACAGTGCTCATGATGTTTACCTAAATG

TTTGCACATATTCGAGCGCTTAGATTTTTCGATTCRTCTTGCAGACGGTGATGGCATTGTCACAGCACCGGTCACYGAGG

AAAARGCACGGAGCGCATGCATATCAAAAGAAGACGTTGAGACATTGGCATTCATAGGCACACAGATTGAAAAGACTTAC

ACAACACCACAGGATATCGAGTGGGCGATCAGCAATGGCCGGTTCTTTATGCTGCAGTGTCGACCGGTTACTACGTTCTT

CAGGGAGTCTGACTGTGAAATGATTCATGAATTCGACAATGGCCTGAAGAGCGAGAAGGAGGTACTCTGCAAGGCAAACA

TGTCAGAAGTGTTGCCTGGTGCAACGTCTCCACTGAGCTACTCCTTCATCCGCGTCGGCATAGACACCTACTGCAGGGAC

ATATGCCTACGTTTGGTGAGGGCCTGTGACCCGGACCCAACACAATATCATTCCTTGTGGATGCCCCTGCAGCGGTACAA

CTATTTCATGTGGCTGTCTGACGGACAAAGGCGAACGGGGCCTGGGTCATCACTTCTCGATCAGTCACTGATGTACAGCG

TCATGGGACGGGACGTCAGCGACGAAGTGGCAGATGGCGTGCAGAGAGCCAGGCAGCTGAAGAAAACGAAGCTCCCAGAG

CAAATATTATTCACCTTGAAGCTGATGCTTTCTAGTTGGAAACACTTCGACAAAGCTGCTGCTAAAGCTGCTGAGCTGAA

GTTGTCAGACGACGGAATGATCAGTGCGGAGCAGTTGTACGTATACATCGGCCGAAGCCTGCATCACATGAGAGAGCCGG

CCGAGCTTTTAGTCAAGGCATTTGCAACGTCATCGATGTACAATCTCATTATCATACAGATACTGGCTGCTGCAAGTGGA

GAACTGAACAATGAAGTTTTCAGTGAGCTGTCAAAGATTCTTCTTGG

>CK184920_bb No definition line found

CCTTAGGGTACCTAGCAAATGTGCGAGGTATGGTAAAGAGCTTTAAAAGTACTCGAGAATCGATGAGAGATCATATTACT

TTAGATTCGTGTGGCATGTCTCGCTTGGCGAGTCGAGCTACAGCAGCGGCTAAAATTTAAGGGCTGCAGCGTCCCGCACA

GCATTGTATCTGTCTCTTTACTTCTAGTCCATTTGAACAGCGCAGTTTTTTGTCACCTAGTTAGTGGAAAAGGGATGAAC

GTAGCACAGCCACAATCTAACAAATGCCTCGCAACACAAAGACTATGCAGTTATCGTAGATAAGTGTTCATTGTGATATT

GTGTAAATCGTCATCAAAGTAGAATGCTATGTATACAACGTGCGTAGATACAAAGAAATTTATATTTATAGTTGTTAGGC

CCCTTGAATGAGGGCATGTGTGACGAGCGTGCAGTTTATGTGCATCCCCTTTTTTTCATATGCGCTGTTTTTGCATACGT

GCACAGGTGATGTTTTGAACAAACGTTGCCTTTTCCTTGACAATGAACGTGTACTGTTGTAACCACATGCAGCAAACTGA

CTGCTTTCTTTTTTGAGTGAACGGATCAAAGTAGGCTAGAGAAGTTATTTGTAAGTAAAAGAGTAAATGGACATTTTGTA

GTTATTTWTAAATAAAGATTTATTTTTCTTATTGCGAGTGCACACAGCACTACCATTTTGTAACGTGCGGGGCACTTTTT

TTAAAGAAGCAATCCAGAAATTGAAGCGGTCATTTATTGTTCGCAGTTTGGTATCGACAATAAATAGAGTGCACGACAGA

AAATGTGTATCGCAAATGTCATTAAGGAAGATAACCATTTTGAATGTACCGATGACAAAAGGCAATAGATAAGGGTGTCT

ATTCAGAAGGTGTTTT

>CK184959_bb No definition line found

AACAAAGGACCATAGATGACCAGGTGTCACCGTGTGAATGTATTAGTCAGTTGATCCTTTTCTGTTTTTAACAGTCACTG

AGCACTAGCTGAGCTGTGCTATTTGCTAAATAATTGCTGTTACCTCATCAGCATCATCATCCATTTCCTTCAAGTTCAGA

GGACAGTGCTACCCTCGGATAACTTTCTTTTTACAGATGTATGCYTCAGTARGTATTTACATATAGCATATGTGTCGATG

ATGAATTCTTCAACAAATTGCACTTTCAAGCACTCTGGAGTGGAGGGGAAGGTAGCAAGAATGAAATAGTGTGGTTGATG

CTCCATTAGCATTACATACCGGGCCTTCAAGCATATTTTTTCATCATCCTGTGAACAAAGCTGTGTGTACTAGCATCAAA

AGAAGAGTAAATTGGTGCGTAGGCTATTCAGTTTATCGAAAAACTTATCTCGAGGAAGTTATCATTTTTTTTATTATGAT

GCCAAAGCAGGTTTGTTGGTCATTTGAAACTGTTTTCTGCACCTCACATATTGCACAAACAAGGGAAAGAGACTTTCTCA

CTCTCAGTTTTCCTATTAACATAGTGTATTTGCTGTGAGGGAAGAAACCTTATAACGGAGTACGTGTGTGTGTTTATGTA

GCTTTGCGAAAAACGTCCGATTGTGAATGTGTGTTTTGTTTTAGTGTGCTTTGAGCACAAGGTGTGGGTGTCCAGATTTC

CAGCATCGTGCAAGTAGTGCACTTTTTAACACGTCGCCATGTCAAACTTGTTTTACCACCCACTGCTGCTGCTTAGCTTC

CCAAAGTATTACTAGCCAGTGTAACTGCTACACAAGTTTTACGTTGTGGCGAGCATTTTATACCTTTCAAGAGCAGTAAA

AGAAGTGTCAACTTATTTTTGACATGCTATAAGTTTAAATGCTGTGAGGAAGTTGCATATAAGTTTAAATGCT

>CK184968_bb No definition line found

TCAGAATCATGTTTTACTGGGAAGGGTTTAATTACTATAAATGTGTGAATAATTAAACTATGCTCTAAAGTATGTACTAT

AACAATTAAGCACAGCAAGTGAAGAAAAGAGCATTTATTACTTATGCGGACKCTGACACTCTATTGTGGGGCTTTTTCGT

GGGCTCATGCCTTCTAGGCCARCGCAGTTTKTCTGCATTAGGAGCATACGTGTGATACAATATATAGCCCACAATAAGGC

ACAGACAAGGCTAACCAGGGGGTTGTTYTTTACTTAYGCTAAAAATGACACACRTGCTAAACAACCCCGGTTAACATCAT

CATTGTATAGTTGCTTGCGCAACTGGCCAGTTAAACTGCACTGTAATCACAGACCCACCTCTTTAACTCATCAAGCATTA

ATGAGCAAAAAGAATACTGTTTTACTCGAAGGCGAAAATTTGTTTCCTTAAAGCAAGCACAGTGAGACACACGTTGTGCA

AACAACACAAAAAAAGGTACATGCATAACGAAGCCACTAGCGACATGACATGTATGATGAAGACCGATCATAATGTTTGT

TCCCACGTGTCGACGAACGCTGCCTTCATGTTAATATGTCAGCATGGAACAATGCATAGCCCATAAAAATTAAGAGTTTT

CTTCTGTCACTAGACACAGTGTGACCATGCTGCTTGTTATACTGTGCACTTAAGCATTACTATACAATATAACTAAACAC

CTGTTGTAGCAAGTGTTACAAGGCTCATTCATGCATGTCTCTCTCCAAATCTTTTTTTTTTTCTTTGACTGTGCACCTGG

TAACTGGCCCTTACTCTGTATAATTTTG

>CK185006_bb No definition line found

AAGTACAAAGGTGTTTTCTTGTCATGAGACCTCATTGTTACCTAGGGTGGATTATTATATTGACTTGCAGGTCAAGTGTA

TGTATTATGAAAAAGACGTTTGATTGGCAAAGACCTTTGAAGGCGGTAAGTTGAGTGACGTTTGTGTAGCACGGATGCTT

TGTGGTCTCCAATCAGTGGATGTTAGGTTAAACTGGTTAACCTTTCGAGAATGAACAGGATGGTTAATTGTAAAATAACC

ATGCCTGCTCATTATTTTGCTACAAGTTAAAGCAGTGATGTGTCATGGGTGCTCTCTTAATACAGAAATTGGAGCATGTT

GCATCCTGCAGGCTGTTTTCTTATTTTTTTTTTCGAGGCAATCTAAAATCATTCAAGTTACTTTCTTTTTGTTAAATGTT

GATTATTTTCTTCATTTGTTGACTTCAACATATGTTCAGACAGCTCAATCTTACTGCAGCTGACAAGGGCCTGTTGGGCT

GCAACCATGGCGTAATTCATTGTTGTTGTAACACTAAAGCAACATCTGCGCTTCTAAGGCAACGTTAACTATGATAGGGA

CCTCTGTACCATTTAGCATGATAAAATGTTTCTTCTGAGCTGTAGCAGATGGATCTGTTCTTGCTGCACAGTGTTAACCT

TGAGGAACTGCAAACGCGTGACTTCTCAAGCTAAGAGTAAATTCTTGTTCCTAATGGCCCGAGTGTGTACTCCAGCCCAG

AGTTCTTGGAAGGAAGTTTGGGAGTTCCGCGTTAGAAACTCTCACATGGGCGTGTCACAGTTGCTAATGCATCTTCTCTG

TGCAAGCATCAATCTGAACTGGGCTCCTGTTTTTAAGATGCCGAGGCTTAGAAAGTACATGAAATCTTGGGAAATTGTGC

CGTAAACGAAGCGAATATGTGTTACTAGATCTGGACCAAGCAAGTGGCTATGAAGGAGCCAAATGTTGACACCGTCTCTA

TGAGCAACAAGGCGTTTATGCATTGTGCGCTGC

>CK185037_bb No definition line found

CTTTATTCAAGCAACACACATAATAAAACACAAAGTACATAAAATACATCATGGAATGCCTTTTATCTGTTCGCAAGAAT

ACATACACTATCATTTATAAGCTTCTTGCATGAAGCACAATCACACAGGTAAAAAGCGAACATTTGCTAAATTACAAACA

GTCGTCCCTTACAATAGCGATATACGTGCTTTATGTACATATATTGCAAAACACAAAAAATGACACGAAAGACCATTAAT

CGTGGACAAATAGTTGTTTTCTCACAATCAACAAGTGCAAGATCAAAAAGTATTCTACACAATATGGTGAACGCCCTCTG

AATAATTGCGCATATTGTAATAAATTATTACCTACGTCGTGATATTTTTGTGATAAAGATGATCGTAGTTAARAAAAATA

ACTTTTTTCATATTTTAATTGCTTTAAAGACCAAGTAAACAGCATACGACTATTGTTTTACACTCTCCAAACAAGCTCAC

GCATWTTTTTTTTTGCACCTGACCAACCTCCTTCCACGTGCAGTGAGGTCCACTGGCTATTCGTGACGTTGACTTTAGTA

TTCTTTGTTGAGACTTCGTTTAGACCAATCGACAAGCTGCGTG

>CK185055_bb No definition line found

GCTACGTGGCCCGGCCTGTGGTCACTTATGTCCATCAGCCGGTGGCCACTGTCAGCCACACCATCAGACCTGTTGTGCAC

TTGAAGCAGGAGCTGGTCGGCTTCAACGTTCCTTTAGCCGGAGGGGGACTCAGTCCTGGAGGCCCCAAAGGCCTGCACTA

TGGATGGAAAAAGTGAACAGAGTGTACCTTGACGTTGCCTTACATGTCACTGCTCGGTCGGTTCACCGACTTAGTTTTCA

KCGTCTGGCGAGGCTCTCAGCAAGAACTAAAAAAACAACAACACTTGGCGAGCGTGTGAAACCTGACGCGATGCGATAGC

TGTATCGCCACCTGATGTTTTCACTTTGAGTTGCAAATAATCTTTGTGTAAGAAACGCAAAATATTTGAGCAATTTTCTT

TGAATGAGCTTGTGAGCCTGCTCCCGGGTACAGCTCTGCAGAGCTTGAATATATATTTTTATATGTCTCGCACTGCGATA

GCATACTTTACAATACGCCACACAAAGAAAACGCTCACTCAAGCTAATACAGCTTTACCGGAGTGTCATTTCACCGAGTG

TGCCCTATAGACTACCTCACTGAAGCGGGATAACACAAGGCGAAATAATTATGTTTTCTAATAATTGTGTTATCCCTCAT

CAAACACAAAGTTGGGGTGACAGAACGAATGAAGAAATGACGCCATCTTTAGAGGCAACACCGTCAGCTTCAGTGTGGGA

TACTGACGATTCGCCTGGTTGTTAATGTGCACAAATTCTCAAACACCAAAAAAAAAGAGCTCCAACCTCGCATTTTAGGC

TTAGCTCTTGCCACGCTGAAAAAAAGCTGTTCACTATCACAGGTGTAGTTTTTCTTTGCCTTAGCAATAACGATACATCG

CGAGCAACAGCTTTGTGCAGAGATGCAACGATTATAAATGAAGAAGTAGTGCAGGAAGAAGGTGGTAGGTCTGTCATGAA

G

>CK185347_bb No definition line found

CTTGGCAACTCATAAATAGCATTTCCCTACTAGATAACTGTTTTGCTAGAACTGGAAGACTTTCCCATCCTCACAAGAGG

CGTAGTATGGATCTTACATCTTTCGCTGAAACGACATCTGAACCCAGTATGGATGATGCTAGTAGTATGATCTGGCGATA

CATCGGCTAGCTCTATTCAKTTCATATCTTATGGTGAGAAAACGTACTGGAYAGATTCTATGGTAGTTTGCTGCAATCCT

CAGCCTACAGAGACATTCGTGGTGTTATTAGAAACAATATGCACTTACAGTTCGCGGCACGATTTGCAATACATTGCTTC

AGCACTGAAACACCAGKTGCTTGCTTGCTACATTTTAAGACGCTGTARARAAAGCTAGCAATACATATTCYACAACAACG

TAAAGGCATCTCTTTTTCARAAAGCCCTACGGGCTACACAACTGTGGAACCCAAACCACACTCTAGCGTTTAAWGCAACG

ACGGAGCTYRATGCARACGAAATGATTCGCAGGAATGATACATGCAGTATATAATCAAGAATTGGGAAGCTTTTGAACAA

TGCTCTGCAGCTTCAACTGGCAAAAAGTTGAAAGCAGTCCAAATGTTGCAGTGAAAATACTAAAGCGGGCTGCTCCTTGT

CATGGAGTGTTACATTCACTTGCAACTCAGTCACGTGAGAAATAAAATCACAAAGGCAGAAAACTATCGGCTCCGAGCCT

TGTCTCCTTAGTGTGTCATGCTAACAATACCCTCAAACTTTAAAAATGAGCCTATATTTTTTACAGTACTTGAATGCAGG

CTGCCAGCACCAATGAGAAACAGCGAGCCTAAGACAGACAGTTGGCCTTACTGATAACATGCGGCAACAAATTTTCTCCC

GAAGCACTTGAGTGTGAATAGTTTTGGAATACTAAGATGAATGTGG

>CK185348_bb No definition line found

GTGATCGATGGGTGGTAACAGCATTCTGAAGTTCCTTGCTGTGACACTACAAATATCGAACCCATTCTCAAAGGCCCAAT

TAATGTTTTTATCGGTAAGATTTGACTGTGTTCTATTTTAAATGAGACAGGAACGAAAATGAGCAAGTTGTGAGAACCTT

TACTAAGAAGTGACTAATCAAGAAACAATACTCTCTATGATCCCTCACTCAAGTATTAGACTTGTGTTTAAAAGTAAAAA

AAAAAAACCTTTACCTTCATTTCAGTACCATCATCTATTGAACCAATTGCTGTGAAATTAATGAAAAAAGTTTCTAAACA

GTAGTTTGTTAGTCGCTCTAAAAAACTTTAAAGGGGCCTGCGACATAAATTAAGCACATTTTCAYCAMTTCTAGCACTGT

GGAATGCAGTGATCACATTTGTAAATAGAAATRGCAAAAAGAAAGTGTTTATCATTTTAATTAAATRGATAATCTACCTT

AAAYTTCGRCTGCAGCGACACACAACGGCATTTCTTGAAATCGGAGGGGACACTCGCTCGTGCTGTAGATTGACWCTTGT

GWMTGCMTYCWGGTTGGCTTGGCACACCAAACTGTAGATGCTCTTTGTATGCCTTCAGCCAGAGCATACTATTGCGCACT

GGAATATATAATATTGATCTTTACAATTTATCAGGTGCAAAAGAACTTTTTTTAGTCATAACATTTTGAAAACTAGACAC

TGCATGCTGAGCAATGTCTACTACAAGACAAGACTTTCTACATGCAAACCTTGTTATAACAAACCCGAATCTAACAAAAT

ATTGCCTATAACATAGTAAGTAAAGAATGGTTTTGCGATAGYTGTAGTATTAGGAATATACCTTTATGACAAATTTTCAG

ATATATAACAGTTATTTTTGTGTACGATGCAMATTTGTTATWATGAGGTTTGAGTGCATACTATATCTCTGTAAACTGTG

CGCAGTGATTGAAGGTATCCAGCGACGTCACTGAGGGTCAAACGCGAAAGTAAACGTTGTTGTCCTTTCAGGTTTTATAC

TCTGCAGACTGATAACTTCCATTTCCACACACCAGTTTTTATAT

>CK185410_bb No definition line found

TCATTCCGGAGTGCAACATAGGCAATGACGTAAAACCGACTAAACTACAAAAAAGACTAGGTGTCTATTTGTCACCCTTG

CGTTTCAATAAGTGTACGTCATCATCGTAACTGCGTATCCATCTAGGTCTCCGGGCGCTGGAGTATTCTGTGAACTGCAG

TGTTTYTAGGAGAACTGCAACTTCCTTTGTGGCATGACAAGAGAAACGGCGTTTTGTGAGCATCTTTATTCACAAGGACA

AATTACAAACAAGTCGAGAACRCACACTTTCTGACGTCAGTGGCTAGCTCCTGTGTCTGAAATGCTGTCTGTCCACTTCG

TCCAAGAACACCACTTCACCGACACTGGCCTATAACCCTTGAGATACGTACTTAGAATAGTTACAACAAGCAGTACGTAT

GTTCCTCAGTCGCTATTCCGTGCCTATGCAGACTAGCTTGGCCTTATCAGTCATTCCTGTTTTGGAGATCGTGATTAACG

AGGTGTCTCAACCTCSCCAAGTAGTCATTGAATCACGTAAGGGAAGCACTTARAAATAACACTTATTTCACAGTGGATTG

TGAATGAAGCTTACCTCRTGACGTAGTGCAAAAAGAACGCGCTGAAGCGTGACTTGATAAAAAATAGTCAGAAAGTTTTG

TACCGCTGTCAGCGCTACCAAGAAAGTGGTCTCCGATGCTCCTAACACTGTTCTAACAGTTTGCGGCGTCACGACGGCCA

GTCATCAATGACACATCAAGGTCTATACAAAAGAGTTAGTAGATGAACAGAAGAGGACATTATGTGTCACTTCGAGAATA

CTTCTGTCACAGTATGTTTGCACATATACCCTTAAATATTTATTGATGGAGACACTCGCTTGTCTTACTGCAAGCTCTGA

AACCATCATCACATCTATTCACATTTTAGGGGAACCCGTATTCTGAGGTTAGGTAAAGAATAA

>CK185503_bb No definition line found

TTTCCAAATAAAACTGTACTGGCCACCGTTCCTCCAATGTACAGTGATTCCACTTTACATAATGAGCATGTTATAGCACC

TGTTATAAGTTTGCTGCTAAGCGAAACCAGCTTGCATGCTTTTTCACGCTTGCTTTGGATAGCAGGGATAGCGCTCGCCG

AGGCTGAAGAAAAGTGCTCACTGCCATGAGAGCCGACTGTCACGTGTCAATATATGGATCAGAGTAACATTTAATATGCA

ACATCTGTATGACAACAAAATTTTASAATGCTGCTTTCTCAACGATTACAGTATTACCATTTTTTTTTATATTTAAAAAA

CAAGTAAAGTGGTGAGTTCAAGAAGGCAGTGCCAGCAAAACTGACAATGCAATGAAATGCAGCAATATGCATTTGTGTCA

CAAACACTTCTAACCTCAAGACCTGCTAAAAGCTGTGTAAACTGCAGGGCAAAAACAATTGCCATAGCATAACGCCGCAA

AAACAGCGGAACGCACATCCAAGGGTAGCTTCCATTTGAGCAGCTTTCTTCACGTGGCATTCTGAGAAGAGGGCCTGTGA

GGAACTTTGGCTCTATTTTGTTCCGTGGGAACGGCAACTTGCCAACAAGAAACTTGCGTCGACGCATTTGTACCATCTAA

GGAGCTCAGGAAAATCTGTGGGTCATCCAGAGACTCGGGACCGAATGAAGTCAAATTGTGAAACTGCCTGCAGTCGCCTC

CACTCTTAGCAGCGAACAGATTGCTTGAGCAACTTGTCTGCTTGTCCTTGACGCTMTKWAAGAGTGAGATAGATGAGAAG

TGAGAAGGGTTGACTATATAGTATTTCTGACATGGTAGCAAACGTATCAAAGAGGCAAAGATATCAAGAGGAGTATGCGG

AGTTCATA

>CK185519_bb No definition line found

CCTTGCTCCGGTAGTTGAAAATAATCTGTCCTCTATTGTGGCATGTATTATAGACCTTCGGTGAACAGTACCACAGGTCG

GATTCATGACATCTGTGATGACATAATTGACAGGAAAAATAAAGGAGTAGATACCATAAAAACAAACTTAAATGCCAGCA

CTAAATGAACAGTTATTATGTGTAATTACGTGGAGGGCAATGTGCAATCACTCCATAGCTAGATTCTCAGCATGCAGTAG

TCCCGAGTGTATGCTTTGTAGTCTTCATTAAACCACTGGAACAGTGCATTGGAATAATATCTGAATTGTTTGCTGTTTAA

TTTACACTGGTATTGCATTGGAAGAACTGGTACAGGAAATTCCACTAAAGAACCTGTCCTATTTTGCATACACACTAGCC

TGTTGGTGAACAAAATTGGAAAATTTATGAGAAAACTGGGTATTTTTAGTCCAGCGTGTTGCATAACCAGTCATGTGACT

AGACTGAGCACTTGCACCTATTCGTCAGTTGTTAGTCCTCAAGCTTGTTCTGCTTGACAAATGTTGCCACTGCTTGCATG

AAGACACATTCGTACTGTCTTCATTCATACTAAGCATCTTATTATTAGGGTCATGATAAGTGGGCTCAACTGTACCACAA

CATTTACAGCAACATTCACTTTAATTACAAAATCAGCATGTTTGAATAAGTACTTGCAATTGCCATATTTTTYATTGTTA

CCCTGCCTTTAAAGGTTAAGATAATCTGTCCTTATATAGCAGATACAATGYGTGCCTTGGTAATTATTGTTGCTCTTTCT

CTTTCATACCTTTTACTTGATGAAATTGTGCAGAGCTCGCTGGAAGCTACAAAA

>CK185597_bb No definition line found

ATTGTCAGCAGCTAACGTCCGTCCGAATYGATTTTAGGTTTGACGACCCCCGGACTATCAATTCCATTAGCCGAATTTTT

GAGATCGACYAGAACCTTGCAAAAACTAGACATTCGCGTGATGTGTGTTCGTGGAGCAGGTGGGCGAAACCAATGGTGGA

AGCACGTGCTTGAGTCGCTGTCTCTGAACAAGACGTTGACGAAATTGGTGCATACGTCTTATAACATGACCATCCAAGAC

ACCGAGGACATGGCCGATTCGATCAAGCGGAATACGTGCATACGGCGACTCTCCCTATTCTACACACCGCCGGAGAATAG

CAGCGCATTCATTCGGCGCCTTTCGAAAGGTATCGAGGAGAATTACAGGCTAACCGACCTTAAATACTCGGGATACTGTG

AACAGGAACTG

>CK185624_bb No definition line found

AAAAATCTTTTTGAGTTAGCCTGCATTGTTTTCTTTTTTTTTTTTTTGGKTAAAACTCTTCATGGCTGTTTTGTAAATAG

TCAAATAAACTTTGCGCTTGCCATGTAAGCAACCTTGTTGGCTGAATTACTGCTTTAGCAGCTTCCAGCAGATCAAAAGT

AAAAGGCCATTGGGCTTTATGGTCAGCTTCAGAATAGTATCTTGTGCATATATTTTTAGCCCTTAGCATTATAACCAAGT

ATGGGATAGAGCCACCAAACACCTGAGCTGATAGCAATATTCGCATAATTTTATGCTCAACCGAACCACAAAATACTCAT

TATTTCATTATGTGGCTACATGCATGTGAAAGGGTGTCTGTGTGGGGAGCTTTGCATTGCAGCCAAGTGTCTCTTCATCA

AGAAGAGTTGTGCAATGCTTTCTAACTTGAGCACATGGCATCTGGTGACATCCGGTGGTGTTGTCAGTGGTGCTGTTCAC

TCTTTTTGTGGCTCCAATTTTATGTAGGTTAAATACAAGAGTGATTGAGTATGCAGTGAAAGCTCATTAACTTGAACTCT

ATTCGTTCGAACTTGCGCACTAGTCCCGGCACAGCCCTGTGTGTATCTGTGGGGAAAGCTCCCGATTATGTGAACACATC

GGTATACCCAACAATTAAATTGAACTGGGAGTTTCTGGTTTTCATCGCTTTTCACACAGTTCGGCTTGGAGTGAATCGTA

RCGTGTTGCAAACCAAACCAAATTTACCAGTATAGATGCGAAAGAACGAAAGAGCGGCATTTATTAGCAGATGAGAATTC

GAACGTTGCGCTGGAGCTGTTTGGCAACTTGCAAACAATGCTCATGGAGCCGAGGCAAGAAACGGAAGAATGGGAAAATT

ACTGGTTACTTTTAATTTTGATTGCATCGACTCTGCCATGTAAATAAACGTGTTGGAGCAAAAGTGGCATCGCACGTTTC

GATCTTAATCTGCTCAAGTGAATT

>CK185625_bb No definition line found

AACAGGTCGTGCTTTATTAAAATGGTAGCATGAAAAGTACTGTGAGGCATATGGCAGAACTGAATTAAGGAAACAGACCA

GAGCTGAAGATCGTTTTGTTTATTTGGTTGACTCAATGAAAAGACGGTATAATAAATGCGCAATTATAAGTCTAATTGCA

ACAAAAACAGCAGCAGGGGCATAGAAATGTACACAGCCGTATTCCATTTAAGCAGACTGACAGCAGTATTCACGAGCAGT

TTCCAGTAGAGTGGGATGGATATACATCAAGAGCTATTTATTACAAAAAATTTTCTGAAACTTATGTGTATGAGTGCTTA

ACACTTTTTTCTGGGTGCAATACACCTGCACCACTTGTGGAATTTAAAAGGGCAAAAGTCCAGCAGTTCAAGCTTCACAT

AAAAGATTACTGGAGGGCAGTGTAACATTCCAACAATTAAACTATGAAAGATAATCATTAATCTTAATCTGGTAAATTAA

ACAATTTAATTTGCATAGACTTACTTGAAAACTGATATCACTGTTTACATGACAGCAGTAACAACTAATTTGTAACTGCT

AATTTGGTGTAGAGTGTAATTATGGATGTTCAAACAAAACTAATTTAGTGCACATTGTTAGACTGAGCACGCAGTTTGCA

ACTAATGTTGGTAGTTTACATTAGGAAAAAGTCAAGTTGAGCAAGACAGTGGAAAGTTTCAGTACTGCAAATGCAGCAGA

>CK185678_bb No definition line found

GCTAGAGCCTTTATTTCAAATATATTGATGACAACGTCAGACGTGCGCCCATGTGCTCGAACTTGAAGCTATTCTTACAT

TTTTTTTTCTAAAAGGGAGGCTTCCCTAACGTAACACTTTGCATCATTGTAACGTTGAAGTTGTTCTGAAAGTAAACAAT

GTACATGAGGAATTCCGTTCGTCTATTGATGTATTCAACTATGATTTAAAGTCACACAGTGGCCCGACAAAAAATTCAAG

TCTCGCATGATGTTTAATATGCGCTGTTTATCAACATAAACTTAATTTTTTTGAGTAACACATTGTTTTCACATGATGTT

GCAGTGCCAGATGCTTCAAAATTATTCAATCCTACTTCAGAACTAATTAACAAAACATTTCAAAAACTAAACTGTACACG

ACAGTGACAAAAAATTGTTGTACTACAGCCGTAGCAATTCATGGCTAATACTATTGTCGCAAGAGGAAACGACAAACAGG

GACTCSAAGCGAAAYGACCATTTACTCAGCCCCAGCACGCAATCACTTTTTCGCTCTCTTCTTCCGCCAACCAACACAGC

GGGGCATTACCCTCTCCCCAAAGAAAAAAAAAACAATAGGTGTCGCATGATTCCATGGCTCCGAAAAACAATAAAAGGTA

GCCGAAAATGTTTGTGTTCATTGAGAAGCACAAGGACAAGATTTTAAAAGAAAGAAGAAAAAGAGATGAAAGAAAGAACT

ATTGGTACACGCGAAGTATATCCA

>CK185679_bb No definition line found

AAGAAAGAGAACAAGAAAGCGCCTACACCATTCCCACCACCACCCATCCACTCACTCATTGCCTCTTGTTTTCCCGATCG

TGGTGACCCATTGATCACGGGAAGGTGCCCTTGTTTTCTACCGTTGCGCCCTGTTCTCTGCCACCGCTACCAAGTGACGC

TGCAGGATTTCCCCATCGACCTGTTGCCGTCAACGAGCTATAGTGGGTGCTGGGAAAGTTTTAAGGCCGACAACTCGGTC

GGGCTTCGTGACACTCAGTTTGGTTCAGAGACTCAAAGCGCGAGGGAGGACTAAGAAAGACATCACTCCGCATCCTGCCC

TCATTTCACGATGAGGACCGACTTGCTGACTTTCTTGCTTCTCGTGTTTGCTTTTGGCGCTTTGACGGACATGGCAGCCG

CATACGAAGTGACCCACTTCGACATAGGTCTTGGATGCCCGAATTCATTGCTGACTTGTTCCCAAGAATGCCGAAGTACT

TACGGTCATAGAGGAGGATACTGCAATGGGCCGTTCAACATCGTCTGTACCTGCACCTAACCGGACATCAAAAGGGACTT

GCTACGCGGCCACTACGTCGCTATTCGTGATCATCACAATCACGTCACAATTTGAATGATCATCACACGAATGGTGATTG

CGTCACTATTCGTGTTCAGAGTCTTTTTAAGGGTCACTGACTGATGCGCTAATTGACGTGGTATATAGCTAAGCATTRTA

CCATAAAAATGGTCTATTTCATTACACTACCTCAATGTTATTTTATGGACGTTAATTTATCCTATGTCAGGAATAAAGGT

TCTTTGTTACAGGTAGGTTTCTCTAGTGAACCCCAACTGATGTTTCTCGCTAGACGCGCTTTTGCTGTGGTTGCATGCAA

TATGGGTGGATATGTGGAAGTGGATATACTTCGCGTGTACCAATAGTTCTTTCATTCATCTCTTTTTCTTCTTTCTTTAA

ATCTTGTCCTTGTGCTTCTCAATGAACACAAACATT

>CK185686_bb No definition line found

TACACTCGTACCTCGATATAACAAACCTAACTATAAAAAAATACAGGTCATAAAAAAAGGAAGTTAAGAAATACTCTTGC

TATAAATACAGTGTTGGGAATATACTTTTATAACGAACTTACAGWTAATAAAYGAACTTATTTTAATTAAGATGTAACTT

YRTTATGAGGTTTRAGTGTAATTGCATCYACAGGYTTCATRAAGGGCCTGAAAGTRAAGAAACATTTTATGGGTAGAAAA

GCAGATTTAGCAGGAGCACCKAATCGATTTTCTGTGCTGATATAACAGTGCATTACCATCAGGTGATGGATAGGCTATGC

TGTGGTGCAACTGTGGTCTGATCTCAATAAGCCTCCCTCCTATAGTTCRAAAGAGTAACTTATCAAAGGCTCATTTTCTT

GTTATGCACTACTTTAGTGAAATACAGCARACAATGAAGTMAAGGGAGGTGTAGATGAGGATGACTTGCYGTAAATTGAC

TGGGCAYATTATCCGGAGGTTGCAGATTAGGTCCTTACCAGCAGCAAGTTCTTTTTTCATCCACTTTAATTTTTTTACAT

TTAGAGTATAATCACTGCAATAGTAAATTTAGAAGGCTACAAGTAATTTCTCATATACCTTCCTCGGCTGCATTGTACAT

CACTTTTTTTATTAAGCTATTCATAGTTTACAGTCCGAGTTAAGTTTGTCAATAGAATGCCGAAGGGGGCTTTAAAATGG

TGCATGAAAGGAAACACGATTTGTAAAAAGTGGTGTCAACTTCCATAAAGCGGTCGTATCTTGCCAGCTTCATTAAGCTG

TCTTCCGATGCTGTTTTATCTGCTGCATCAGAGCCACTGATCATAAGCACCAAAGAACACCCAAAACACCAGGATATGAA

CAACGATGCACGAAGTTGACAGTGTGGTGGGTTGGCCGTTACCGATCCTTGTGTTCCTCGTCTCCAAAGGC

>CK185745_bb No definition line found

TAATAGTAGAACGTACWTWAAAAAAAACAGCTATTGGGTTTTATTTGTGTATAATAGAAAAAAAMMAAACAMAAGCAACA

TGTAAAATGTCAAAATGCTAAAACAATTGTTTTTCAGTCCAGGGAAGCCTGCGTCCATCTGCAAAGTCAAGCTCCACTGC

TGCCTCAGTCTTCTTTATAAACGCCTTCACTTGTTTGTCTTGTTTAGCTTCTTTCCTGCTGTAAGTTGTCATCAAGTTCC

TAGGCTGGTGCTAGACATGCTCATCATGTCATAGAGGGTCTGTGGATCTGTCACCGTGACGCTGTCGTCACTGTCGTCAC

TGTCTGTGTCCGAGTTGTATCGTGGGTAGAGAGGCGGATCAAACTCTAAGTGGTACCTCATGTCTTGTGTCAGGTCTAGT

GGGCAGCCTAGGTGCGGGTCCAACTCCGCCTGCACTCTGTTCTGCATTTCAGCTGGGATGTTTGCACGCATGGCCATTTC

CATGCGCGCTTGCATGTTTGCGACTTCTCGAGGCTGCATGGCCAGCCTGCTGTGTTGGCGGGGAACCTTGTGAGCCTCGA

CTTGCATCTTGTGTGGCAAACTGGCACGCGTTTCCACCACCACCACGTCGTTCGGCACGTTACTTGGCATCCCGGCCTGC

TGCTTATCAATCATGGAAGCCAATGCTGGCTCCAAGATTGGCATGCTCTCCTGCGGCAGTGCCACTGAAGTCCTGCAGGC

CATCTGTAT

>CK185763_bb No definition line found

AACTTTTCAACAAAACTAGTGCTAATCTGCTTGCACTTCCCAGATTCAAGTGTTGTTCAGCTATCAAGTGCCATGTTCCA

GATGGTTAGCTATCGGTGCATGCTGATTGGTTGAGCCTGCCCATATATATCACTAGGTGGCTCAATAGTGCTATCTTGTG

AGGGTATGCTCCAATAGTGAAAACTGTTCCTAAATGTAATCAGTCAATTCTATGGAGACAGCTCTAATCTGCAAGAAGCA

GTTTATTACACTTGGTTAAAGTGGGCAAAGATGAAGATTGCTATTGCTGCTTATTTGTTCAGGGAACAGTCTGGTGAGCC

TTGCTGAACATTGAAGGCTAGCAATGACCTAAAGCTTGTGGCTGTGTGAGCTTAACTGAACTGCACAACGTGATCTTGAC

CCCTTTTGACATACATTTCAGAGTCATGTGTTCACCTTTAGGTGTTAAGATGTTGCGTCCCTATGAGCGAGCTTGAGTGT

TGGCCACTTTCTGTTTGATAATTTTGTATTGATGATGATGATGTCTTGTGAAAATGCAAGGACTGCGGCGACCTGGGTGC

AATAGGTACTTGCGATGATACCAATTGAAAATGGTATTAAGGTGTGGCAATGACTGTTTGACACTCCTATTTTATCATCT

GATTGTCCATGTCTGCTGCTTGGCAGCGAGTCGGGCACGTTGGCACTGTTGGCTTGCGGAAGGTTTCATTGAGGAATGTT

GATAATTTCCATGTATGTCTGTGCTCCCTTTGTGTATTTACAGCCTCGAAGTATTCTGATGACATTGATTGCCCGTTTTA

GCTGATGACATTGATTGCCCGTTTTAGCTTTCATGACCCCAAGTAGCTGAGCGTTGCCTGCAAGCGGCCATATCTATAAC

ATTGCCTCCTAGGTGTTGCTTTCTGCGTGAAGCAAGAGCCACTTATGCAACTGCGGATGCACTTGCATTATTTATTCATG

TCAAGGTGTTTCTATTTTTTAGCATTCTATGGTGATGCGTCAGGCTAAACGTGAGCTCCAGTTTTATATCTTTTTAGCTG

ACGTTTTGGTTGGCAGCCGAGTGCAAGTGC

>CK185769_bb No definition line found

TTTTCACAAAATGAGAAAGTTTATTGTAAAAAAACTTTTTTACTTGAACTAAATACAGAACTCTTCACAAGACCACTCTG

CCTGAACCAGCCCGCTTCTTGGCACACAAACACAAAACAGGGCAACAAAAACAAGAACTTCCTTTAAAGCTTTTGGTAGG

GCAGGCAATCAATGAAAGGAGGAACACTAACACTTTCCTGCACTCCTACAAATATAGGGGCACAGCTTTTGCTTAACCTG

AAAATGTCTTCTGGAAAAAAAAAAAAAGATGGGCCCGACTTCAGGCAAGCARARAGTTCGTAAAGACTGCATTTGTGTAG

CCGTCCACTTCAATCTGCTCATAAACGTAGTTGCGGTTAGACCTGCATTCGTCTCCGCACTTTTGCGAGTTACACTTGGG

GCAGGGAAAATGACAACCAGGGCAATTGGGCTGCAGGCAGTCACAGGCATCCTTGTTTGAAGCAATGTGGATACCTCGAG

CATTGTAGTGTCTGTGTGGCCGAGTCCTGAATAGGACTCTCAACGTGGAGGAAGGAACAGGTTTGTCATAGGTTGCAATA

CCTCTGCTGTAGGTCCATCTGAGCTATCATGTCATGCCGATACTGACGGCAACGCAGCAGTGCCATTGATCGTGCCAGGT

ACTGACTGCTGTCCTCTGTCGGCCCAAACTCCTCGCGCTGCTCTGTGCTTGGAGATGGGGGATTACTAC

>CK185845_bb No definition line found

TGCATTAACAATGTGAACAAACAAAACTGAAATCAGCTGCAAACTGGCGTACTACTGTCGATGCAACACAAATGTGCAAA

AGCCTCGCCTCCATAACTTTCACTCCAATGTCAAGAAAAGTTGTCGCCGAGTATAGTCTGCTGGCTAGCGCACAGATACC

ATGCACTCAATGTCGTACGAGCGTTGAAGGGATTATGTTTACTGAACAAAWTATTTGCCTCCGAGGTTTTCAACCAAACT

GWAAAATTGGGTGCTGATATCTGTGGACACAACCTTAATGTCAGACAAACTGCCGAACCTTCAACTGGCGTGCCAATAAT

GCTATGCGTTGTCTCTCGAGCCCCTCAGCCCTAATTACGATTTCTAACAGCACTATGAATGATGAAATAAAGCCCCTAAA

GTCCCAGGTGGGATAAGGCCCCGCAACAAGGGGTAACATCTCAGAGTCTTTGTGGAAGGGGGAGAGCTTGATATAAAGGA

GAAAGTGGATGCACCAGACACGAACACATGCACCTTTCTTTTACCACGGGGAGCTCACTTGCCTATTTACTCTCTCTAGC

TGCTCATGTGTGTTCTCTGTGCTTGTATATTTGAAGATTTATTTTCATTAACAAATATTTATCATCCTTATTGCTGAACT

CCCCCCCCCCCCCCCTTTGAAAAAAACAACAACAACAAAATACTAGGGATATGAATTAGAAAACAACCGGCCAATGAAGG

TCGGTTGTAGGTGTTACCGACCCTTTGTCTAAAAAATTGATCAGGCCGATGCAAAACCCTACCAC

>CK185856_bb No definition line found

TTGAAAAGAAAATCACTTTATTGTGCGTAATATTGTCGACACATTTTCACAGTGACACTGTTTTCAGGTTCTTCTGGTCA

ATTTGCAAATGTCGACTGTACTTGAAAAACTSTACTGTACTGAGAAAAAMRTTAGCCAGTTCAAAACTGAAACGAACTCT

TCAATTATGAAACAATARAACGCAGTAAGCGTTCGGTAAATTCACATTTGARACTCGTTAAAMAGTTAAAAAATATACAT

TTCCCCTCTCTCCTCTACTTCCAACGTTCTTATAGTCGAACAARATTCTTCGATATGCCCGGGTTTAGATGTGACATTCG

AGTTTTAACGCGTAAGCTTAGATAAACTTCTC

>CK185857_bb No definition line found

GTGCTGGCCCTGAACATATGATCATGTTCACGCTGCTGAGATTGTAGACAGGGCTAACGCACTCGCTATGCCTACTTAGG

GACAYGTGACGTGGAACCACATGAAATRGTGGTGCGACTGTCTCAAGAGTTTTGTGGCCTCTGTAATTTTGGGAAGAACT

GTAGCTCTCTTAGGCTGACAGTGCTTTCAAATCCCGTTCGCGAGAGTATTTTTCAATGCCATCGTTAATAAGAGCATACG

TAGCTGAAGATGTATCGATGTACAAACTCTGCAAAGGAGTACATCGAAGGAACGCTCTGTAAGTGCATTAGGGGCTGGCT

ATATTAACTATTGCAAGTTATTCAGAGTCACATCTTTGATCTTTAAAAACAAGGATGAGTTTACGTTGACGTGAAATGGT

GACTACAAAGACTGTACCGCATTAAGTCAAGCCAGGTTTTTTTTTTTCTCAAGGACAAGCTGCATAAAGGCTAACCTGAC

TTGAGGTCCGGGCTAAATCTAAATTTTCGACATATACTCAATAAAATGGTCAATCGGGAAACGTGTCTGTTCATATCGAA

AGAAATGTAGTAAACATTTCGAATTTCATTTGCACAAAATCGAAAAATGTGTGACCACTGGATACTGAAGTGGGCACGTG

TTTTGATAACGCCGGACCGAGAACGCCAGCTACAAGCCATGAAACGTGAAATCAGACACATTTTGCGTTTTTTTTTTTGC

TTCCTCTTAGAGCAAAAGTGGTGTACTGGCATAATTACAAAAGCTAGAAAATGTGGATTTATCGGAAAGATTATCAAGTT

AAAAAAAAAATGTCTCAAAGACATTGTCTTGCCAGACAAGTTCTGCCCTAGATCAGTGGTTCGTAGTTTTTCCAGGACCG

TGTCGAAAGTAAATGTGCACAAGCCGATTGGTAAAGGTGGATGACGAATAGTTAGCCGCTCTTAGCGCATAATTGCTCTG

TCTTCCCCCTCCCCCCCTGCGGTTTTTTAAACTGTGACGGGCGTTGTTCTACACTTTCAACAGGCAGAATTCATCGAGAG

CCTATACAGAGAGTTCCTTATAATCAGGTGGACAAGCCTGTTCT

>CK185891_bb No definition line found

TAAATTGTGTACATGTTTGGAATGCCAACTATTCCTTGTGGCCAGTAATTTAGTATTGTAGGTTTTAGGCAAAGGTATCC

CCATGCTTTGATGCTGGGGCTCTTACACAAATATTTACGATGGCYTRATTGCMGWGTTCTMCACGGGTATTACACTTAWA

CGCTGACATCTAGCATAACATTTTTGTCCTGCCATGGCTRTAATGRAAGTGGGGATACTWAWTTTTTTTTCAAGGAATTM

TTTTTTCAACACATAGGTAAGAAATTGTCCATTTGTATATTTGTAGAACAAAGGGTGCAAGTTTCACTTCTTTGCTTTAG

CCCCTAATCTCTGAAGGTAAAAATGTTTTGTTCACTTATTGCAAATGTGAAATATGAGGGCTTGTAAAGAGGCTGGTAAG

GGGCGAGACTGGGTAATAATGTTTCTATTTCTGAGCTAAACTAATGTTACACGTTCAACAATGCCTTGTTTATTTATTAT

TTTTTCTAGCAGAACTTTGATCAGCTGAAAGCTGCCTTGTCCTAACTGCTAGTCAACTTCTCTGAACTGCACGTGTGCCC

AACTGTAGTTCTAATGAAATTATGAGAGCCATTCCTGCATTTTAAAACGCATAATATATTAGAGATGTCTCATCATACAA

TGGAGATTAGACATAAAAGGTTGCTTTATAGTCATGGAAAAGATGTGTTATTTAGCAAACTGTATCTGCAGGAACGAAAC

GTGATTTGAAATACAACGCCATGGCAAGCCTTATATCGGGTGCAGTGTAGGGAAATGCGATGTGGATTATTGCAAAGCCG

TGATGTTATGAACTTATCTTCCTGCAATTGTTGCATCAAGCTGCACGTCATTGCATTTGTCATGTGCTGGAAAAACAACT

CAAAGGATATCTATCAACCTTTGTGGCTGGCTTTTTTGCACTAGCCCTGCAATAGGTGGTGAAGCAGACCACTTGGTGGA

AGAAAGTAAT

>CK185907_bb No definition line found

CTTGCTATGTACGCTACTAGAGTAAAAAAAAAGGTTAACACTAATTAAACACTGTGAAATTGCAACCCTAGTGATGGCAT

CTTTTAAGTAATAAAAGCCAATCTTGGAAGGCCATGTGATCGGATATCACAAGTTTTGCTAACACTGCAAGCGGTCACAG

CATACTGAATCAAATATGAATAGCCATGTTTTGGAGAGTTCTTATGCATGAATGGCAAACACTTTTAACACTGCAGTTGT

GAAATTGCAGTCTAGTTTTTCCAGTGCGAGAAGTTCTCAGTGTATATTATTGTGAGTAAGAGCGATCTGTAACACTGCAG

CTGGTCTGGTAGCAGTGACAACTGTTTATCATAGTGTGTTATAGCATCTTTGTATATCTGAGGCTGTGTGAAGTTTTCCA

AGATGTTCCAATTATATGCATTTATACAACAGGTTTTCAAGCTTGCTCAGCACGACTGCATTTGGACCATACATACTTTC

AACACATGTCCTTCCGTGATCGCTCTTAGATTTTGTGAACTCCTTTTTTCGTTTGCAGCGGTACTTTGTTGATATTTTAT

TTGATATTGGTATGGCTGTGTGATAGTAGAGCTTCCCAGTAATATTTACATCCAATTAGTACGGGTGTCGCATAAGCATG

TTCAACTGCAATTGAGCCCCGATGGGTATCGAACRTCGCGACAGTGATTAGCCCCCATCCGCTCCTGATGAATATAAGCC

AATCATTGCCAGAACACCCAATTCGAATTTGGCTTGATCGAGTACACTGTATGATGCTCGTACAAGTTCCACTATTAAAA

CAGCCCGACCTATTACATAGCACGCAATTGCTGGTACGG

>CK185922_bb No definition line found

TTTTGACCTGTATACAGCCCATCGTTTTATTAGGCGTTGAAATGGCAGGCAAAGCAGCTGGTTTCATCGCAAGGTAAGAC

AGTAGGGAATAAATAAATATTACACGCTCTACACATGTACACGTAAATACGGAGCTTACTCAACACATACACATACAAAG

GCACAAACACAGAGACACACATACAAACTATGAAACAGAAAGGACAGTAACCGACCAGAACAATTTCAGAGGAGGTTCCA

ACGTGTGACACAGCACACATACAAAGTCAACGTGAAGTAAAAAAAAATATGTGAGGGTACCTGGAAGTGATCAGAGTGTA

YCKAARAAATTAAAGAAAAAAAAGAAGGCGCTAAAGCCAGCGCGTTGTGTCACGCTTGGTTCTCTAGCATTGGAGGTCCG

ATTATGGCAACTCGAGAATTACGGATGCAGTTATACGATGAGTGACAGGTTGGACGTTCGTGAGTTACAGGTCGCTTTAT

AACYRTTCAAARTCACCTTSTAACGCGTACAACAGCGRGACCTARAWGTCTAAACAAACACAYATATTCACCACAGCTCG

TAATAATTGACTATACAACCAGCAGCAATACCTACCACACTTATACTSGAGCRAACGATATGGTTAACTATCAGAACACA

AAGCGGCCTGTTAGTTWATCACGACTAARCACAATGCAGGTGTGATGTGAATGGTCGGGTATATGYTCAAGAATGCCCCT

TGTGGAAAGTGATGGTAAATCGATTGACTGATAAACGATGCTTCAAACATGATTCGA

>CK185960_bb No definition line found

GATGTTGCACATCAGCCTTTTAAGCCAATCCCTTTGCTTTCACCTTCCGCAGCTGATATGATAAAAACACTTTACTTCTT

ATCATCTACTTCAACATCACTTCAAAATACTGACATGGTTTATCGAACCTAGGTCCTCGGTAGCTTACACAAATTGACTA

GAAGGCAATAAAAAATTTTCATGAACATATCTACTAGACAATGGTGAATTGCAAATTTTCGCGCAAACACAAGTCTATGT

TTGACCGATGCACAGACACAGCTTGTTTGTAAATCTCTCAGCATGAGGTAAAGTATAAAATTTGGTGAGCCAGGTGTCTT

GCTAGATTAAAAAATAAATAAAACAGCAGTCATTATTCAAGTCAACCTGGGTCGTATGTTTGTATAGTTTTCTTTTCAAA

GTTCAGTRGTTCAATAATGTCACTTAATTTCTCGAACAGTGATTGGAGACTGYGTAATTTTCCAACACCTGAGAGCATTG

ATTAAAACTGCAARTTAAGCTATGACAGACACACTGGACTGAACTGAAGCCACAGAAAAAAAAATTAGCKGACATTTTAT

GCTTTAACGCTCATGCTAAGTTARGTCCACTCATTCATTTTCTTGAACTGCAAGTGCTAAATTCACCTTGTGCAGTTTGA

GCAACATTTTAGTCTTCTGGTCAACCATCATTAACTTGACAAATAATAATTCTTCTATTTACATAGGAGCCTTAATTAGA

CAGCTCATTATCTCAAGATGCAGCCAAAGCTACTTTTGTTTCAGCCAACATTTACACCGGAATCTGCTCACACTGGATCA

ACCTAGATTCATCTTACCAGTGGTAGCATTGTGACAGAAGCCCATAGTTTATTTTCATTTTTCAAGTTTCCAATTGTAGA

AATGCCAACCAAAAATGTCAAAAAAGAAAGCTGTGATGCTACATGACAGTCA

>CK185975_bb No definition line found

ATAGAAAGCTGTAGCAATTTATTCAGTTACAAATGCATGAATTAATAAAAACATCTGTTCTAATGCAACACAACAAAGCT

TTACACTTAAAAAACAAAGCAAATGCAAGCAAATATTATCAACAGAACTTTGCTTAAAATCCTGTTTTCTATAAAAAGTA

ATATCAATATGACTGTTACATATAAATAGAACATGCCTGTATTGACCTGCAAGGCTTTGAAAATAAAATCTGCTTAAATC

AACTCTTGCATCGTAAGAAAGTGATTGATGCACCAACACTTCACAAAGAAAAACAAAAAAGGAGTTCCAATAGTGAAATT

ACACAAAGTGGTTCATGATGGCTCTCGTTTCACGACTGGCAGTTCGACCATTCGGACAACAGGGTTCAAATTTGTAACAC

TTGCAAGCTCGCTGTATGGCACTCGTGCATCACCCACTCGAATGACGTTGTCAGTGAAGGTTGAGGCTTTCAAACGTTTT

GATTCCATTTGCTTGAAATGTAACGATATCTTGCTGTTCGAGGGCATCAACTGAGCATCGCCACTGGGACTAGGCATGGC

ATTCCTGATGGTGTTGGAAATGGATGAAATTCTTTGTAGTTTCCAGTTACCTTGCAAACTTATTGYGCTGGCACTGTCCC

TGGAAGAGTTCTCGTGCATTTCTGCAAAGTGCTTGCAGAGRTCTTCAAAAGTACCAAAGGAGATCAGGCACACAGAGCAC

ACATGCGACCGTGCGTGTGCTCCAAATAGAACAGAATCAGGGCCACTGCCAGCCTCATTCTTGTGTACATGCATGGCCTT

TAGAGTAGCCAAATTCTGGAGAACTTCTTCCTCAACTACAATGTTT

>CK185976_bb No definition line found

CGAAGTGTGGCATTGTTGTTTCTCCTGTAGATCTTCTTGGACACATTGTTGAGAGGCACAGTGGGGTCATATTTTGTCGT

TATGATGACTGCTCATTTGTAAGTCAAAATCAGTGGGATGTAGATGAACACATATTTCAAGCCCATCAAATATACCAGAA

GGAGAAGGCAAAGGCTGACGAAGCTGTAGCAACTACAAAAGCTGCACTTTGTACTTCAGCTATTTCATCCTCTGCAAGTT

ATGCTGAAGAGGGTAGCAAAGATGGTGTTGATATTGCCAATGTCTTGTGCTATGAAAATGATGAGGCTGATAAGCAGTGT

GATGAAGGTCAAACAGAAACTGCACAAGACCTCACTGCAAGATATCTTTGTGGCACTTGTCTTACACAGTGGGACACAAC

ACCTTCCTACCTCCATCATATGACAACTGTGCACTCTGTGGCATTTTTCTGTGGACATTGCTTGAAATCGTACAAAAAGT

CAAGGAGTCTGTTCGTGCATGCTGGTTATTATCATCTTGGGCTGCCATTTTCAGTGTGGAAATTCGAGGATGGAAAAATA

GTTGAAGTTGGCAAGCTTGTTGCACCATCATGGGTTCATGAAGCTCAGTGTTATCTCAAGCTGACCTGTCGTGCAAGGCT

TACAGCAAACGGTAAAACCATGATAAAAAATCTCAAAACTGCAATCGATTATGTGACCTCTGCTGTTCTCTCCAACGAAG

TGGAACAGCAACAACGAGAGACCACATCCTCTAATATAGATGACCAAAGTGTATCTACAACTCCAGAGGCTCACACACAA

TGTCCAGCACCTATGGTAGACAGCTTGTATACAAGGGCCACTGGTAGCCACTCTTTTCAAGGCAAGGCATTGCATGAACC

TCCGTCAGTACTGGACAATATCAGGAACAATGGCATTGATCGATCCTTGGAGGAATCCAACCATTCAAGAGTTC

>CK186036_bb No definition line found

TGAGTGATATGCATGTCACCTTTATTTTTATCAAAAAGTACAATATACATCATCTATAWYGAATTAAAATGAYTCGTAGA

AASAAGCGCTCAACGAGCTCAACTCCACAAAAGGTCTGCGCAGACCTGCGCAGTTSGAAGTTATATCAATGCTCAATAGR

AAAAAAAAACAAAGTATAGTAAAGAGAGGCCTTCAAGAATGCCTTGCCTAATGCCCCATAAAATATACCACATTATAGCT

TCCCAAAGGAATGATAAATCATAACTAGAGGCCTGCTGTACAGCCTACAAMAAACATGTACGTCTAAGTAGCACTTCTAK

TCATAGAGMTAACACTGTTTAAACTTGTCACGGGGTATGATCAAATGGTTCAAGGTAAAAACACGGAGAAAAAATGTGGC

ACAAAGATGGATGCGACACCGTTTCTGCTAATTCAGTGCACCAATCGCAATAGTCCTTCCCGTATATTACGTTACATACT

CAGTTGGATCAGGTGAAGACTCATTTTAATGGGATCACGGTAAGTGTAGGTAATACTCATTCTAAAGGTATTCAAGTTGA

CATTTCTTGCATATCGAAACTGAAATAGAGAAAAGACAAGAGTTTTCCCTGGTGCACTGGCAGGAAGCAAAATACATTTT

AAAGAAAACACTGTGAGGTTATCTACAATGCAGACGAACAACACAAGACGTAAGTAGACGTAATCATCCTC

>CK186093_bb No definition line found

TCATGCAAAAGGCAGTCGCCTGGGAACAAAGAATCCTTATAATCGCACTCACATGGTAAAATAAGGCACCATGCCATTGC

ATCACCCTAAGCGCCTTCACAAGCGACATCTTTTCCCCCTTTTCCGGTCTCCCTGAACACTTCAGCCACCAGTAACATGT

TTAGCATTTTAATACACGCACTGCGTACACGCTAGAATCATCAGGCATCAGTGACTTTAACACGAGAACCGCGAGTCATG

GTTTTTGATCTAGAGGTGTGATATACGATAAACATTGTGCGAGTCAACAAGAGAAGACGCCGTTGAAGCTGCCAGCCCCA

CTCARAGGGCAACATAAAGACGCAA

>CK186131_bb No definition line found

CTGGGTGAACACAGGCAATTTTCACCGAAGTGAGGCTGCAGCTCTGAACAAAACTTCAACCTTGTTTTCACATCTGCTGA

GAATAACTTTAGCTCCTCCACATGCTGTGAAAGGTTCCTAAAACCTGACATGACAGGTTGGAGGAAGTGCAACTTCCTAA

AACCTGTTATTTAAGGCAGCAACAGAATTGCGCTGCTGAACATGACATTATAGATTCAATACATGATGTGGTGGACAGGT

TTTGATACGCAAAGTGCAAGAACTGCTCTTGTACTTAGGTGTCGGTGCAAATAGCTATGAATTGACAAAATTTATGGAGA

ACCCTCATTCTCATCTCAGTACGGAAACGTGTCGTCATGTGTTGAACACTTTTCAAAATGGTAATAGTTCCACCTAAGAT

TTTCATTGAGTCAACAATCTTTAGGCATCGACAAGCTTCATTCCTCCAATTGCAAAATGTATGCAGCGGTGTTTGGTTGA

ATTAGCCAACACTGTTTCACTTGTGTGTTAAAAGTCATTGCTGATGTGCATCACCCCTCATAATGTATGGCCTCTGTACT

CGTAATGTATGTATCTTTAGGCACCGACAAGCTTCATTCCTCCAATTGCAAAATGTATGCAGCGGTGTTTGGTTGAATTA

GCCAACACTGTTTTACTTGTGTGTTAAAAGTCATTGCTGATGTGCATCACCCCTCGTAATGTATGGCCTCTGTACTTAGG

GCCGTTCTTCGTTGAAGCAGCTGGTTTGCATGTTTATACACGTATCTAATATTTGCATGCCAACTTAAGGTCTGTTTKAT

TCAGCACAAGTACCCGCTGCATTGACTTGATAGCTGTGGGATTCTGCGGCAAAGTGGGAAATGATTGGTTTGATAGATGA

TCGTGGCAGGTGCGTTTCAAAGCAGGCAGAATATTACAGTGTTTGTGCACAAAGTTTCGAGMATTTAATGATTACAGCTG

GCCAGAAGTGTACTAAATACCTATCACAGTCTCCCTCACGCAGAGAGAGTGAAAGACAATGGAAAGACAGGGTA

>CK186134_bb No definition line found

ACACCAGGGACCGCGTAGAACACAGTCCGTTCATCTTTAATAGAGATCGCCACAATGAATAAATAAAGTAATAGCTGCCA

CGGAACAACACCTGCTCAGTGGGGCCATTTCTCACACATTATTYRTAATACTATTGCTACATKTCCGTAAATAACAAACR

KSTTTTCATTAGATTCAACATGWGGCATACGTRWWCWGCAGATCTTTYTTCTGCAAGTACAAGGTCATTCAAAATTTCCC

AGGCCACTATTCCAACTAAKCCTGTGGCAGCGTCACCTGGGAACWTTTGACTGCASCTRCACATGACTAAAAAWTTTACG

AAGTGAATTCAAAWGTTAAAGAAACCAACAAGGACCTGTTTCCTTTTCACTGAAATCGAATTTTACAAAAAAATAATTTT

ATTTCGGTGGCTTTGTATCTGAAATTTAAAATACGAAGGAATATCATAACTGCCTAATACCCAAAGCACCTGTGTTGTTT

TCCTATTCTAACAGTATTGTGCACTCTTCTTTTCTGAAAAAGAAAAAAAAAACATTTTGCCAGCACAACTACAACAGGTG

AGTACAGAGCAAAAAACAGTGCGAGCACAATTTGTTAATTTATTTCAAGGCTCCTAATAAGCCTTGTTAGAGGCACTGGC

TTGCAGCACAAAAATAAATGATCACACATAATAGGAAAAAATACGTATCATACAAAATAAAATAACTGAAAGCACAAACA

A

>CK186185_bb No definition line found

TTTTTCGAAATGGAGCATCAGCACAGCAAGTGGCACTGTGATATCATTCCCAAAATATCATTATGGCCTTCTGCATAAAT

TAGCCAGCTTGCTTGGGAGGTGTAAAAAAAAGTCTGAGCGTATTTACTAATCGTTTAAGCCCTGAATGAAACCCTACATG

TTGATTATACAAGAAATTTTATTACATTTAGTTTCCACTCATAAAAGAGCTGAGAAAAGAAAAACGGCAAAACCATGCAY

CATACAGGAAATTAAAAAAATCCCGCCATCATGCAGCCACCAAATTACTAGATACAAATTAATTTCAAAGAGCTCATTCC

CAGATAACATCACAGTAGATAGGTGAACTGATGTCAAAAGAACCATGAATAAGGAACCACAAAACGTAAGCCAAGAGAAT

GACAAAAGGAGAGACCAGCACTGAAATATGCTTGGCTGAAAAGAATGATGCCGCAGTGTTTTTTTTTAGTTGTTGCACAT

ATCAAGTTAAGAGGCATGTGGAAGAAAGTCAATCCTGACAAGGAACATGAGGAGTGAAATCTGTACAGCTGCCCATTTTT

TTTTACACCTGAACACGTGAGCATCGACTGCCGAGTCAATAAGTGCCAATAGCCGGTGTCCCGGTCTCCAGTCACTGCAA

CAGTGGGGCAAAAGCAACTGCACAGTACTGTTTATTGCGGACATGCCCATATTATCATTCATTTTGCCACCTTGCTTCAT

TATACGGCAATAATCAGCCTAGCAGTCAACGCATGTTAAAAAAATGGGTGGTTTTAGATGCATATGTGGAGTGCACATGC

AGCGTGACTTATTAAATCCCATTTTTATGCAACAAGAAATGTGTACCCTCAATGTTGCATGCACACACTACTACAGTCGG

TTTCTGTTAATTCAATCTTTGTGGAAATGCTAAGTTTAT

>CK186186_bb No definition line found

CTTGTGCTGTTAAGCTCAAGGTCAACATGGCTTGGTTCTCCGCCATGACAGCCACGTTTCGGTGGGGGTGAAATGCAAAA

ACACCTGTGTTTTAAATTAAGGTGCATGTTGAAGAACCTCAGTTGTTCAAAATTAATACAAGGTCTCCCATTGTTGTGTG

GGTCATAATATTGTGGTTTGAGGGCGTGAAACTCCAGGAATTATCTTGAACAGATTGTTAATTTCGCTCTTTCTTTAGCA

TCGGTTATTATCAGGGTGTGGCATATGTTTGCACATTTTTTAGCATGTATATAGAATGCATAGCCAGATAGCTGCACACT

GCTGTATACTGGCATATGTGTTCACTAAGCCTGTCTCTGAGACAGTGTCCAATCTGGCCCACACAAACCTTGTCACGTGG

CCATACTCGGGAATTTTTTGTGGGAGATATTCTGCKATTGCAGTTGTAGCCGTTCTGCATATAAAGCTCAACATGTGTTA

TTCAATTTGTCTGTAAGTTAAGTGCATTGCTGATACAATCAATTATTGTTTAAGGCAACTTTCTGCATAAATTTAATTCA

CTGTTATGTCTCTGCAAAAACCATACAATGCTGTGGACAGAATTTTTTTTTTCTAAACRCAAAACATTTGCTCACTTTGG

CCACAACTTGTGCTCCATCATGATCCTAGTGGTGGGGAATGGCTGAAAAAGAAACAAAAAAAGTAGTAAGGTGGTGTTGC

TTCTTTTTTACGGCAACAAGGGCAATGGCAGTAACCTGTTCTTGAGTGAAGACGACCGACACACTGTGCTCTGTGCTATT

ACGCGAGTGCCAATGGCAGTGTTCATGTCTTTGGGCCGCTGTCAAATGGCGACATGTATAATTATGATATT

>CK186215_bb No definition line found

TCAGCTATTTCTGGTGTTCTGGGGCACCGTATTTATTGACATTTAAGTGAAAGCGACCTGAATTCTTACGCCACCACCAA

TATTTTAGCTGTCTCATTGAAATAATTCTCCTCAATTATAATTAACATTAAGGCTTTGATGCATGCACAACAAAGCTGGC

AAACGGCAGGGGATTGTTCTTCTGGTATGCATTACGCCACGCAGGCACATGCTTTCTTGCGTCTTCTTCGAATTGCACCT

TCTCCTCTTCTGGTAGTATGGGCCACACCGGATTCGCCATCAGGAACATTTCTRWYRGSGTWWTCCGTGTCTAGAAATGA

AGACTTTCGTTCCCAAATATCCAATGTGATCAGAGTCAAACCAGCTGAGGCAACGAGGTTTTCTACATACGACCGTAGCG

TATTCACGTCGGTCATATCATGAGTCTTGGGCACTGCATTTAGGAAAACATCGCAATATTTTGTCCAGGGTTCGAGCTGT

GCCATTTGTCGAATGAAGTGAAATGTATCACAGCGACCTAAGAACAGCAGAATGCATTCTCCACCGGGTTCCATAAGGCG

GCAAATATTGGCAAATGCTCGAGCCTGGTCCTTTGCCCAGTGCAGCGTCCTTAGGCTGTACACGCGCTGAAAGGTTCCAT

GATCCGACAAGAAGCTCTGCACGTCAAGGCCAATGTCCAGCAGCTTGTATTCGATAAGTGGGTGGGCAGAGTGTTGCTTG

GCGTACTCAATCATTTTCGGTGAATAATCCACTGCCACGATCCGCCGACATGGAGGACACCTGGGCAGTATATGCTCTGT

AGTACTCACGAGAAAGCCCCCGAGTT

>CK186287_bb No definition line found

GTCCTATAAATCAAAAGTAAAAAGAGTGTCTCCAGATCGCTTTCGACAAAGAATAATATTTTCGTTGAAATAAACAAAGA

TGAACCATCAGAAATAAGCTAACGAATGCGCGTTGAGAAAAAGTTAGCATCCATCGGGTTCTTCGCCGCGAAACGTTCCA

AAGTGCAMTCTGAGGYGCGCGTGTAGACTCGGTGACAGTTACGCTTACCATTTGCTTTGTTMTTCACCCAATTAAATCTT

AGTCYTTTAAATCTGATAGCTCCGCTGCAAGGCAATCCACTGAAGAGCGTTGATAAGCGGGGGTTGGTTTAGCTGTGACA

GARCTGTTTAAAAAAAATACAAAGGCATCCACGGAAAGTTTGAGATTACATCCCAGCCATGTGGTGTGGGGATTGGAGCG

AGCTACCACAGGACTCCATCCGCATACTGGGCGCACAAAATGGCCCTGTACAAGAGCAGGATGCCACTAGTCTACTGTAT

ACACTTGGGTCTCACCAGGACAGAAACAGTCATCTTGTTCGGCTCGCCTGTCTAGGTCCCTATCTTTTTGGGTCCACCTT

TTTCAGCAAGGATTGTGAAGTATGGGCCAGGAGTGCAGGGATATACTTCGACCCTTCAAAGCTAGTGGACAGCAACATGA

GTGCTGAAGTTCGTGAAGCAATCATGGTTCTTGACACATTTGCATACCATATATCATTCGTCCTCGACATTTGCAACATG

AGCAGGTGTACGCCCAGCACGACGCTACATATGGCCGAAAAGCACAAGACATTTCTCAGGGACTTCGACAAGGAGGCACA

TTACTCCACTGTTCGGTCAATGGTGGAAGCTTCCGACTTCTTTCGGCTATTTCGGAGGAAACTGACAACGTCACTCGGAG

AAGAGCAGATTCTGGCCTGTTTCATGATAGCCCTGAAAGTGTCACTTGCTCATTGCCACGGGGCCCTGGACACCATGTTG

ACATACATAAGGTGGTACAGCGAACTGAATTAAAGAGAGAAAACGTACTTCTTTAGTGGAGAGAA

>CK186339_bb No definition line found

GGGGATTATTATTCTGTATTAAATACGACTTGATGCAATATGGATCCCTGTGTAGTGTGTTCTAAGTGCAGAAAATGACA

TGTTTGTGTGTTTATACAGGTCAAGGCATTTGATGTAACATGTCCAGAAAAAGGCCCAGTCTTTGAGATTCCAATCAGTG

TCATCAAGCCCAAGCCCGTCACCAAGGAGGACGGCTACCAGTGGAGCATTGAGAAGCTGACCTTGCATCCCGGTGTGTCG

CACCGAGAGTTCCTCGTCGTTCCTGTGGGGGCTACCTGGGCATGTATTCAGCTCAAGTCTCAGGACCCTACGAACGTGGC

CCATGTGGTTGTGCATGCCATGCAGCTGCAGCCAATGTACTCCTGTGAGACTGCAGAGTTCCAGAAGACGTGCATGCTGT

CTCCATTAGCCGAGGCATCACATGCCTTTGCTGTCGTGGGTGGCCTGACCCTGGAGCTGTGCCTCGCAAAGTGGTGGACC

AACCTTGGCGACGTGGATGTAGACTGCACACTCACTTTCTATGGACTTCAGCCCAAACCCAGTCGGTTAGTCATGCGTTC

TTCAGAAGGAGTCTACCGCTTTGATGTGACCTCCCACCTCAGACCAGAAGAAGTGTCTCCTGCAGCCTCCCTCAAGCAGC

ACGTCATTGTTCTCCGACCAGCAGAGTCTAAGGTTCGGCCACTGGGTGTTCGAGATGTGATTCCCGATGGCAGGGTCATC

TATGAGATCCAGCTCACCTATAATTTTTCCCTGAGCAAAGCAACGGAAGTGACACCCAGCTGCAGCCTGCTATCAGAACT

GCTTTACGAGTCTGAATATGAGTCCCAGCTGTGGATGATCTTCGACAACAACAAGCAGCTTCTTGCCTCTGGAGATGCCT

ATCCTGGACGGTACTCGACCAAGCTGGAGAGAGGGGACTACGTGCTGCGGATGCATGTGCGACACGAGCAGAGCGC

>CK186345_bb No definition line found

GTATGTACTTTCTTAATCGAACGTTTTAAGAAACCAGTACTGAACTGTAGTTTTGCAGTGCTCGAAATGTATGGTGAAGG

GACAAGTGTAAGTAATATTTGAAGGTGAGCAAGACCGAGGTTTAGAAGGGAGGACCCTTTACTCTGGAACTTTATTGAGC

CTTATCGATTCTAAATGCCGTGTGTGTTCACTTTGTGTGTTCCCGTGCTCACTTGAAACCCGTTTGTTCGGTGCGCTTGT

ACATCTAATAARAAAAATASAACGTCSCCCAGAAGGTTCTCGAGTGACTTCTGTCCAGGTCTAAGTGYCCGAGGTGTTCG

TAAATGTTTGCGAATAACTGCAAGAGACTACTCATCGAACGCCGCTCATTTCTTTAGCTTCTCAATGTAAGTTCGTGGTA

CTTTGTATCCACTTGGTTGAAAGGGCCCAATCTGCATAATATGCCGTTGAAAACATTTGACGTGATGCATAGAGTAGGTT

GGCGACAAGAAGCAACAAGTACCTTCTACAAAAAGTTTATAGAAATGGTGTGTCCAGAGCCTAACACTCAGGGAAACACG

TCGTAAACAACCCGAATGGTCCGTCTTTCGMGCAGGAAAATATATGCCCTAGTACTTATGAGTGAAATTAACARTKTATC

AAGAAATATGACGATAAATAGGTGAACTAACAAACGTYATACAAGTCGCTAGTTAAAAAGAACTGCTCGTCAAAGAAGMG

AAATGGTTTTAAAAAGGCGTTTCCTGAAACAGCACTGSTATTTCAAACGCCAAAAACTTTGGCACCGTTYGCAAATGGMA

CAGAYCCCCACTGTAACCAAAGTCATCTCTACCCTATGACTCGGAAACACAACATCGCTGGGTAACAAAAATGACATTTT

TTTTTACGCATACGCGCCGACGCTTCTTAAAACCTTGCTTTTCTGCCAAAGCTGCGTGGATGCTCCAAAGGAAAAAAAAA

AAAAARRAGCCCTCCCGCCCCTCCCCCCCCCCCCCCACCG

>CK186346_bb No definition line found

GCCGGGGCTAACGGCCTGGAAAAATCTTATTGTCTTCGGCAAAATGGAAGCAATGTCTTCGATATGTCCTTGCAATGTTC

TGCAATGTCCGGCAATATTCTTCCAGGAGAGAGTATCACTTCTCGAAACGTTRGCTACGGCGACACGCCATGTTCAAGAA

TATTACTCTCAGTCATGTTGGTGCTCGTGCTAAATAAGTTCTCGAAACATAAATATTTTGCATCTTTCAAATTATTTCAA

ACTGTCTTGCCAGCCCCGTAAACAATGATTTTGTTCTTAACGGCAACACTACGTGCACGAACTTTGACGAATTCTGCAGT

GACGTCAAAGTTGTCAAAACGTGATGCCAAAAGTAGTGCCACAGAGTTTCATAA

>CK186351_bb No definition line found

AGTGGAGGCAGTAGCAGCGGAATTTACGCCCTGTGAAAATGGGTCTTTTTTTTCCCCAGGTCWCAGACTTGATCTAGGCA

AACCAGGACTTTCGTAAACATCTCAATGCAATACTTAGCTGTGTGACGAAACTAACACTTCAATCTYGCCAACTGATAAC

WGTTGACCAAAGTCTTTTGCTAGTGCTAGGTAGAATTACGAAGAAAGGTTTGACCGCGTTAGCACATAGTCCTCACTGTT

GGAGACTCGTGTGTGCTGTTGGAGAGCAGACCTGACCACATCTTTTTTGAACTGTGTGCTGTCACGCTGAATTTCTCATC

TTCCCGTTCTTCACTAAAGCCTTTTAGCAATGAAAAGAGAGAGTGCAAAAGAGTGCTTGACTTATGTAAGCTTTCTTCTC

GCGCGTGTGTTGCACCAATCGCCTGGTCACACCGAAGTCGGTAACATGTAGTCATGGTCCTCCTCGTCTCAATTTCCTTT

TTGTTAGGTTCTTATCCTTCAAAGATTTTTATCTCTGTGGTCGGCATCTACTCAGGAATCGCTGTTTGTGGCTCCACCAT

TACTAGTATAACTGCAGCAACGACCTTGTGAACTTGCTGCAGTCACAGTTCGATCTTGCTCGAGGCAGGCGCGCGTCGAC

AGTAGCCATCACTGTTAGTCTTGCACTCTGGGTGGGACAGCCTGCTCAGTTCGTCCTATGTGCCTGTGAAGCCTTAACCA

GCGGTGAAGTAGCATCTGACGCTGTCCTTCCATTTTGTCACTTGACAACTATCGACATGTCGTATCAGCGTATTGATCGA

CCTATTGATAATGCATTGATTTCTTCCCTCTGTGCATTTGCCGTCATGAACTGTGGAACTGGTTTGTTATGAGAGATGCT

TTGAATATGGGATGCTGTCGAAGCCAAGGAATTCTTCTTCCATTTTTTAGCAGTGAGAAACGCATACCAGG

>CK186371_bb No definition line found

CTTCAGGCTTTGAATTCACGAAATATGCGCATCGCACTTTATTGCCCAGTCTGTTGGCACACGAAGTTGGCTGAGTAAAT

TTGCCAACTATTTACAATCCTTACAGCATTTTTCCTAGCAAACTGTGGTAATTCATCACAAGCACTGAGATGCAAGTAAA

GTGATTATAGCTGCAAAGTTTGTAATCACAAGAGAATGGCACAAATGCATACACATCAAAGAGTTAAAATTGCGTTTCTT

TTGGTTCATTGACAAATTATAACATAAACGAAAACTGTTTCCCTTGCCGAGTAAATAAAAGCAACACTAAAACACGCAGT

AATAGAACAAATGGCTTGCGCAGTGTTGCCTGCCTTTGTAAATAAAGCAGTCAGAAAAAAATGGTAGAATGAAATGCTTC

GTTAGAGATACGGTAGTCACTAAGCACTTTCTATTTACAGAGTCACTTTCACACCATCATGTTGGCAGTGACTGCACGAC

TGGTGGTCAGCACGCATGCATCTGCCTAACATTCATGTACCCATTGAGATCAAGTCTAAGTTCCTGGGGTGTGCAAATAT

TTGAAATTTTTGAATTGCAAATCAATTAGTACCCCATTTCAACTTACAGTCAAATAGCTGCTGTAAAGTAAATGAACACA

AAGTCTGCGCAAGAGTAGAACAGACTTCCTTACAGCAAGCATGCAGGCATAGCATAGACATAAGCTATAACGCCTCACAC

AATACAGCAGGCCATGTTGCTAGGGAAGCCATGCTTACACAACGAATCTTGCACTCTCTCTGAAGCAAGCAAAGRATATG

CTTAGTTGCTCCCGATGCTGTATATGCTATTTCAATGCTACAGTTATGGACATTAGAATACTAACATTGCCAAAGAAAAA

CCATAATTTTCATGTCTCTGAAATCGACAT

>CK186372_bb No definition line found

ATGTATTGAGTATAAATAAGCATCATATATTTGACATTAAAACACACTGCTCACATAAGTGTATGTCTGTGCTTGTTTCT

GGCATATCGCTGACTGATAGATTACCCCTGTTTGCTGTTAGAGCTCCATGAACCTAATTTACGGAGTCACTTTCYAAGAA

CATGCAGTAGCTCCTAATTYGTCATAACGAGTCTAGAGGTCGCTTCTTTGGCTTCTGCCCACACAGTGTGGCGCTATGTC

CATTCATTCTGGAGCCCGCTCGCTAATTTGAACTGCGCTTAGTTCACAGATTATCATAGTACTCCTCGAGTTCCAGTTAA

GGAAACTCGAGCCAAAGTGATTGCGAGCCCGTGATTCTGGATAAGCGAACGCGCTCTCTAACCACCAGTGCACATGCCTT

AGCTGCTAATGTAATACTGCTAATAAAAAGTTTAGACATCGGCAAGCTCCAGTATCACATACATTGAGTCTGTAATGACA

TGTGTAAAAATGCCGCATTGTACATAGAGAATGTGCACAGAACTTCTGCTCATGCGCGCTATGCTCACCACAGGATTCTT

ACACATGCAGCACCATTACGGACGCAAAATACGGAGCATTAAAACTCACGGCTTACTGGCTTAGCAGCTTTATGAAAGTT

TCTTCCATTCTGTGTGCTACTCCTTCATAATCAATTTAAACAAAGTGATGTTTTATTGAATTTGCCTGTGCAGATCCTTG

AGTAATTTCTCTTCATCATATTTAATTCTGTGGTCTTTATTCTGTGGTTCTGCTTTGCTGTACCCCTGCAGAGATTCAGT

GGCAGATAGGATGTGTGACTAATTATGGCAGCTGCGTTACCAACATTTGGAAATTCTGAGTCACTACAGTGTGCCCTGAA

CTGTTAGACGTAGATGTTTGTAGCGACGTACGTCATTCATTGTTTGTTTTATCCTGACACATTTGATGGCTGTTCACAGA

ATAAGTGCATCCTGTTTTCAAACAGATTGTGACACTAGTAGAGGTGCGCGAATGTCGATTTCAGAGACATGAAAATTATG

GTTTTTCTT

>CK186409_bb No definition line found

ATTTGTATGCATGTTTATTTAATAAAAAAAGTTAATACCCAAGGATTTCTCACTTGAGAAGATGCTGTTATCAATGTCAT

TGTTCATTTTTATTAAGTTTGGGTGTCTGGTAACACCATGGGGAAAAAAAAATGTCATTCAGGAACTTGATGTGTTGATG

ATTGAATGTCATTTTTTGTGCTTTTGTGGCACAAATATTGTTGTTGTAAGTGGCTAGTATATGATGAGCTATGTAATTGT

TTTATGATACCCTGTACTCTTTGTGGGTTGTGGGGAAAATTGAGTGGGTATTCAAATGGTCAACTTTCTAACGATGCCAT

GAAGCATTGGCATAGAAAATAGTAGGACAATGACTTTGTGGGAGTAATGGTTTGTAAGTTTCTGTGTTACTAATGCATTG

TATCGCAAGTGTACAACAATTCTGTGTGTTTTTGTTCACTGCCAATACACTTCCACTATAAATGTAATGTGATGTAGTAC

ATGTTGTAGGTACACGGGCCCCTGCCAGACGTTGCTCGCTTTTAGCTCTAATACCTTTTTTAAGAAAGTATTCAAGATGA

AATGAAGATACATTCACTCATATCTCGTATGTGTTGCATATATCTCATAACTATTGCTGTCTGTATTGCTAAATAGCAGC

AGAGTAATGAAAAAATACAAAAGGGTGTTTATTGCGTCCTCTTTACACAACAGTGCCAGATAGCCTGACTGCGTATACAT

TGTTGGAACTCATTTAGTTCTATGTTTGGCTCATCATGCAAAAGTACCAAGCAAGTAATGCTAACTTTTGCCTTAGACAC

TAATGCCTTAGTCCTATGCCTGTGCAATCTTGTCAACGGAGACAAGTTCGAGTGATCAAGTATG

>CK186430_bb No definition line found

GAAAGGGGGCAATGAATGTCTGGTGGTGTTTGTTTCTTCTGATTGCAGTAGCATCCTCGCAGGAAAATGCTTCAAATGAT

GCACCTGGATGCGGTGATATGAATGGACAAAGCGGTGCCACGAACTCTTCGACGTCAAGCACAGAGAAGACACCACCATC

AACGAGCACAACTAAACCTACGGCCCCACCGTCAACGTCGACCACACCGTCAACCACGACCACACAGGCAACCACAACTA

CACCATCAACCACGACCACATCGTCAACGACCACTACGCGAGATGCTCGTCAAGGTGGCAATGGAAGATATGCCGTGCGC

GTCAACAAACGTGGCTGTGTCCGAAAAGTCCTCACGTCTCATGGGAGTGAGTACCCTGCTAGTTGTCGAGTGCGATGTCC

CTTGTACGACAGAATATTGCCTGACAGGATGACCTGTTTGAAGGTAATTAGGAATCAACTTCAGGAGCGCCGAAGCGTTG

CGAAATTGAKGTGTTGGAAAGGATACTGCMKCGACGGCGTGTGCGTTACTACGCATYKCTCTCAGCAGTGTGAAGTACCG

RAAAACAGAACCTWTACTCGACGTCCMRATCYCCTTGCCGAATAAATGTTTCGCCTGAAGATGCTGGGTCAGATATAAAC

CACGAAGTGGCAACTGCTTGGTGCCAGTGAGCACTTCTGGGAAATGATTGTATGTATGTCCA

>CK186468_bb No definition line found

GTTTCCGGGAGTTATCTAGAGCTTTTGCTGTAAAACAACGTAATTGAAACAGGAGAAGTGCAATAAGTGTTTTTCATTCG

TTCAATTSAGGCAGTRCAGTCGTTATCTAGGACAGAATTAGCATGATTAAAAAGCTTCTTTATGACAAATTACTTTTGAA

GTAGTAATGATCAAGCACGTATGGACTTCATTCGTTTAGTTCCTGGCGGTATRCACAAACATTATCAAAAATGTTSCTGC

GGCTGAAGCCSARATCCGAAGCACCGAAGCAAACTGTTTTCAGTTGTTGATRTTCAACCCCAGCTTAGCGAATGGGATAA

AAAGTTTTTTTAGTAATTTGTATCAGCGACATTACAAAAAATAGTGCTCTATTGTTTCTATATCTGTGAAAAAAGTTGTA

TAGAGGGGATATTGCCAGCAGGGGGGTAGCAGGCGCGACATCCCGATTTGTTTATTACGACTTACAATTTTCCAGTTGGA

CAGCACTGAATATTCCACAGAATTTCAAGCGACTAAGTCATGGGGTAGTCATTATCGATTCTCTACATCTTTACGAAGCG

AATATTTTTTTCCATCTTAGCGCAGTTGTCACAATACAGAARAAACAATATTATTAGATTATTCAATGCCGCTCGTGGTC

TCGTTGAGAGTCAGCCATCTTATTTAACCTAAGGGCAGTGTCTTGATTTTTTTCTTCTCCTGATCTCATGGCTGATATTC

AATGCAGGCTATCGGCCGAGTTGCAGGTAAATTTTTCTGTACCCTTTGTGTATTACTCGTGTAACCTAGA

>CK186503_bb No definition line found

TACACATCTCTATTTATTGAAATACGTTCTCCATTTCAAACAGTTAAAAAAAAAGAAGGTAAGAAAGAAGGTGTCCAGTT

TTTCTYTTTTTTTTCTATCTAAAATATGCAGYARACAAGAATTWYCATGAGCTGTGGTTTGGCAATGGTATTGGCATCGA

CACAAAAGGACTGCCCTACAGTATACCGRARAGACRTAACACTCGGTTTTAGAAAAAATGTGCATATCGTAACGTTATTC

TTTCTCCTTTTCTTTTTTTTTTGTCTCCAACAACAGTATWGACATCATATTTACACAGAYACAAAAAAATCCTCAMGCAC

ACRCTCATACAYACACACAAACACAGGCGTGGTTACTCTTACAGAAAATGTGCTCGCACCGGCCAATGGTCGTCTTAAAA

ATTCTCTAAACGCTATACACACACGCCTTGTCTTTCAGGACCTTCCAAAAGCCATAATGCCACACAAAAGACGAATGCCA

GTGGTCTATCTGGAGTTGCTTATTCTTATTTTTCATTGTTGTAGCTTAAAGAGACAAACAATTGTCTCGTTTTAGCAAGC

TACTATTGCACAATACCAAAACCACCATGCTTGCTGCGTGAAAATGCTTGGCAAGAGAGACAGCACACTAAAAGAGTATG

CGGGTGGCGATCCCACAGTGAAGTTCTGGCAACAGCTGCAGCGACGTGKATGTATACTGRGGCTTGAGTAGTTTACAATC

GTCAAAAGTGAAGCRAATTGCCCTTGAAAAAGCCAGAGACATAAGATACCATGTCTCAGGAGATCTTATTAAAAGCTCAC

GTGGCTCAAAACGCAATCACTTTGAAATTGATGACGTCACGTGTACATTGGCATGCAATTTAAAAGTTACACTTTTTACC

TCGATTTTCTTCACAGTT

>CK186515_bb No definition line found

GGTATTGCCACACACAACATGTACAGTCGTGGCCTTTATAAAAGGGAACAATGAAACTACTCAAGAGGTCATCTGCCACA

GTCACAAAACATAAGCGTGCCCGGAACACTGAATGCTTAAGCACAAAGTTATATCTCTCAAATTGGTATGTCGGAATCGT

AACTGCTCACAAGTAGCCAAAACAAACCCTAGTGTTCTCCTTCACATTGTTCACGACTGTACTTATGACGCAAACATCCA

TGCGCACTTATGCAGTGGCAAGTAATTGCTGMATAAGTTTTCAAGARAAAATGCAAGTAATGCCCTCAGCCAAGGTATTT

TTAACTGCTTTGTCACCTTGCAGTCYCACCAWGTTTTTTCAGAAACCCATTCGTAAATGAGCTAGTAGTCCARACACAAT

GGCCATGTTTACTTATAATKAATTATGTACCGTTCACCAAAGCTGCTGGCCTCATTGCACTGATTTCTTGGGCCATGATA

AAAGTACGAAGCTGTGCACAACCCCAGTATTC

>CK186516_bb No definition line found

GATAAAAAAAGCTTGCAACGTGGTTGACTGTGAGTCAACCACGCAACTTGGTTGACTGTGAACCACTCAAGGAAACATGG

ACAGGGGAAGCACACACTACTGGCTGAGTTATGTTTACTTCAACTTACTATTGACACTTGAACATCGTTTCATGGTTGAG

CGAATAACAATGCTTGGCCACCGTGCTGCTCGTTGTTTTCTTTCATCTCCTGCATCTTCGAGTGTACTGCAAAGCATGCC

AGTGTGCGTGTGTGTTTCGGCACAAACTAAATATTAAATTTAGTTGATAATGTTCTGGTCCTGCAGCTCATGTTTTCTTT

CCCAGTTTGTGCTTACATGCACAGTCGGTCATGCTAAAAGAGTGGTCAAACATTTTTTCTTCAGTCGAATCTTTAATAGT

AGTTKAAAAAGAAAGCGATGCCGGTTTACTAGAATCCTGGTTAGATAAATGCTCTATAGGGGCCAGTTTATGAGCCTGTT

GGATGCTCGCAAACCAGCATTTATGCCAAGGTATGTTGTAATGTGACGTGCTACTTGAACGCAGTCTCAAATTTTGAAAA

TGGCACCATGTTGTAGGTCTTCACCGCTTTGACTAATCTAAAGCCTTCTACAGTGGGTGGAGCAGCCATTCAAGCGCATC

GAAACTTATCAGATTTGACAATACGTAGGAACTAAAGAGTTTCCAGGTGGTCATGGCTTGTGGTGGCCAAGGCGGCTTAT

AAAAAAACTGCTGAAGTGGAAATTATTGCTCAGGAGTGAAGTGGTATCCTACAAATTATGGCGGCTGTGGTCACCATCTT

GTTCTTGATTACCTATTAACTGTGGCGCATACGTACTTCAGGTCTAATTCTACATTGTCATCAGCCACTGCATGAAAAAT

TGGCTAAAAATCCTAGCAAGTGCGTAGCAGATACCACGCTTCT

>CK186586_bb No definition line found

GAGAGAGGTTTGTTATAATAATATAGAGCCAGGAATAAATTCAAACTAGTCAGAAACATTGAACAATTTATTTAAAATAG

TTTCTACAGAAYGCTTTTAATGTGTCACAGATGAAGACATAAAAACAGTAATATCTTATCATGCAATGAAGCCACAAATG

CTCTCACACCATAATTACAGAATACAAATCTTTGCTTACAAATACGTGCTTGTCATGCTATTGCAACAAAATAGCATGCC

AATATGCTATATCACAGTACCTTTCGTCACACAAGTGAGAAATAGGGCATGTAATACTGGAGGTCTTCACCATGAACCAT

TAGTCAAAATCTGGAGCTTCTAAAAATATCACCGCGGAAGCAATTACTGTATAGATGACTTCAGACAACACAAAAATATC

TGAAAAACAAGTACTCAATACCTTAAAACACAAAGAAAATTGCTGTCTTTGGTAACCACTTCCAATTAGAGGAGTATTTA

TATAGAAGGCCCCAATGAATGTACACCACATTAAAGAGAAACCAGGAGAGAGATATGCACCAATGAAACTAGTGTTATTA

CTATTGAGCAGAGGTCATCAGTACCGAATACTTCTATCATTACAATTTTCAGTTTTCTGGACAACTCAAAATATCAATTC

ACTGGTTTATGTTTC

>CK186608_bb No definition line found

CTTCAATTAACGTGGCACTTCCAGCCGTGAGTCGAGTAGGAAGGTTGAGTAGGTTTCAACATGCATAGGCATTTATTCCG

ATAGTATACTCTCTTGTACTTACATCATTCCCTAGCATGGTAACATTAACATCACCCATAAAACAAACGTAGCGCCAGTG

CTTCTTAAATTGTTCAACAGGTCTTCCACAAAATTGAGAAAACGATCTTTGCGGCCGTATGGTGGCCTCTACATAACGCC

CAGGTATTCACATTTTATACGAATGACAAGAAGTTGAACATTTTCATTCATGACACTATATTTCTATTCTACGTCATAAT

AAATCTGTGTTTTCACATATATGCATGGAAGCACCGTCACAACAGCGATTGTTTCGTGTTAGTCCTTCATACCTTTATAC

ATCGAATTGGGGTGTGCTGTCTTTTTGAGATAACCATYTTTCCGAAAAGCATTTTTCAAGCACTTCTTGCAAACTTTCCT

GCTTATTTTTCATACTTCTTGTATTCAGATGAAACAGGGACAAATTGTTCACAAACTGCATCACAATAAGAGGCATTCAG

ATAGGAATAATCATGGTAGGAACAGGCATTATCAGTGAAACCTCACCCATCTGCAGCTGTACTGCTAAAATACGAACTGG

AGGTATAAGTGCCACACAAATTCCTTCTCATTACTTGTGTGCTTGAAAACACTAGACAAAGTTATCAAGGCGCTTAACAG

ATTACGTCAAGCGCGCCACATCACACTCGGATATCCATTGCGCGATCTCCGTTCTTCTGTTGCACCAATTACCTTTCTAC

TTGCGTGCCAGGCAAACGCGTACCCAGTGCTTTTCGCCCAATCCTTAGTAACATATATAAGTTCACGAGACAAGCGAGTC

TTATTCTCACAGATGAATACACCACTGTTTTCTC

>CK186641_bb No definition line found

CAAGACTCAATTCACTTTATCACTCTAAGAGTGGAATTTAATACGAACACACACTGTTTCATCTGACACCACGTGTTKAT

CAGAAAAGTTGAGAATGGCATATTGTGTTGTGTGCCCGCTGGGAAATGTTCAGGGGCATGTGCTTAAATAAGGTTAGRTC

ACTGGTACTTTCACTTCATTGTCAGCTCATACAGCCACTGCTGTGTCTGATGAACTATTTGGCAACGAGTTGTTACTCAT

TTGTTTTACCAACTTGGAAAGCGCAGACATTTTCTTGGTGTTGTGTGCATTTTCTGGCTTACAGGATGTTCACAGGAGAT

CTGGACAGACGTGTGTAACAGCGACTGTCCTCTGTACAATTTTTTTCTTAAAGGCGCGTAGTTTTGAACCAGCGGGTACA

GGTTGGTGCTGTATGTTGACAGGCTTAGTAGCATTGCTTGTGTGAAATAAAGGCTAATAATTTGTCAAAGTTTTAAAGAA

TTTGTCCAAAAACAGCACTTTACATAACATCCTTCTTAAGGAAGTTTTCTTCCTGGCAAGTCTGTAAGGCTCGTAAAATC

AATGGAACGAAGAGTTTAACCGGAAAAAAATGGTACATTATTGTGACCAATGCAGCTGAAGGGATAGTCCGAATCGTAAG

TGTAAAGGCTAAGTAGAATGTCCTAGGTGATGTGTTTATCTTTGGGATTGTGAACCCTGTGTGCCTTTTAAATGTGAAGC

CATACAGTTGAGCAAGACAGTATGAAATTGCTATGCGTTTGATGTGTCTTGTTTGTGTTGTCATAAGCGTACAACCACTG

TTGTGCAGCTAGATTAACTTATCTGATGGACTGACACTGACAGGTCAATACAGTGTGTTTGAATGCACTC

>CK186649_bb No definition line found

AAAATATGTTCAAAAAAAACAATAACCACGTCCGTCTGGGGACTGACCAAGCATTTGGCATTGCATTCACACGTGAGCGA

TCGGTCGATTTGTGAAATCGAACGAGCGAGCGACTGTTATTTATTTTCAGTTTATCATCGATCTTAATGCATCGAGGTTG

TGGGTTGGGTTGGGAGAAACTGTTTTGTAGGTTGTGTTCTTTGAGAGTCTCTTTTGGGTAGTCTTTGTTTTTAGTTCTTC

TTTTTTTCGAGTTGGAGGGATTATTTATTTCATTGCACCACCTTTTGCATCGAGTGAAAAAATATATATATATCCATTTG

TTTCTCATGAGTATAAGATGTGATGTGAGCTTTAGGTGCTCAAGACTGGACGATGTTGCGGAATCTCCCTGGTACTTTTT

GTTTTGGCCGGCGCGGAGGCAACGAATCGAACAAGCTTTTAAAAACTCCTTTTTTTTTTTTTTTTGCAAGCAGCGTGATT

GACATGTGGCAGGAGGGCAAAGAAAATTCATAGCTTAAAGACACAGTCATTGTCGAACCACAAGACAGCGAAGCATATCC

AAGTGATTCTCGTGGCTTTGTGTGAATGTGGGCAATTTCTTTTAGCCCTCCTTGTACAGTAGCATCGTGAGATTGTTGAA

ATCCGCTGTTTTTGTGCAAATGTAGAAAAAAAAATGTGACAACTTAACTTATTGACAGTTTCATTATATGTATAGACATA

TACACCTGTGTTCGAATGCAGAGGCAGTGCTTACTACTATAATTATAACTACGATTAGCTATTCTCGCCGAGCTTTCTTG

GCAGAGCAGGGGCCCACACGTGTTCAGTTTTATTTTGTGGCAGCAATTTACACTAGCCATGTATTAAAAGAAAGCAAAAT

CTCACACTGTGTTCCTTTAGCACATCTCGCCCGTATGTTCCAGCAGGCGCATTTTATTTCATTTCTGAAAGTGCTGCGCT

TCGTT

>CK186764_bb No definition line found

AGTTAGAGGTCGCTTTGTCAAAACCACCACCCCCACCTTGCGATTTGGAGGTTGTGGATGTCAGTTAACAGTGCCTTGCG

GCAGGGACGATTCCAAATAAACTTGTGGGTGAAACGGGGTTTGAGACTGACCCTCATTTCGGCATTTATTTCTTTACTAT

CATTATCATTTCCAGTAACTCGATTACTATACAATTTGGTTGATGAACTATCATTTATTTGCRTTTATTCGATGCWCGAA

CAAATTATAACACAGCAAAATTGAAACTCACCGAGATATCGCAACGTGAGATCACTAGGATATATTCAATGAACAAAAAG

TATTCGATGCACTTGGATCGTGAATGATTGCCGCTTAAGTCCGGGGTGTACTCTAGTGACAGAACTGAAGCCGTTGTGAT

AGCCGCTCAAATTGAAAGTCCTTCCATGATTTGGAAGAGAGAGAGGCAAATGGTTAGCCAAAATGATTAAAGAAAGCTTC

AAAGCGACTCTTTGGTGTCACATATTTCGAACAAAGGCTGAAATTTCACAATATCCAAGATAGGCGTCAACTGTAAAATC

TCTCACAGTTGCATGTCTGTCACTAGAGTAAACGAAAAAAATGATGCTCAAGAACTAAAACAACGAAAAAAAAAACAAGT

ATGGCGTACCCAAGACACGAAGCGTACTTATACTACATATGTCCATAAACTCTGAAACACTCAACTACGAACGCGCAAAA

ATGAACATACGAGTAACTTAATAAAAACAGCGAAAAGACGTAATTGCTGATATAACGTACACGATCGAGCGTACCAAGTG

GAATATTTCGTATGTCTTTCTAACAATTCTATTTGTTAGCTTACATAATCCATAGTTCTTAAAAAATGATTATGTTTCTT

TAAGAAATCGATATTGTTGCTATATGAATTCTCTATGTTTACTTACGGAATCCTTACGTTTCTCACATTACTGCCGTAAG

TTACCTAAACAATTCCACATTATCCCTATTA

>CK186765_bb No definition line found

ATTTTCGTGTGGTACACGTGTTTCGGCAAGGCGTACCATGTGGCTATGATGCTGTATAAAAACATGAAAGTTCACTTAAA

AGAGGTACGGCRGTTATTTTCGAAATAGAAAAARTTAAAAAAAACCTTTACTCATGGGTCGCAGCTTGCACAGGATAACA

TGACTTTTTCYGAGTGCTATTCAATGGGTTTAGCCATGCCCRCGAAAGTGTAACACTTTRCAGGACAGCCAACTATCACC

AATAGGACGCGCGAAGTCACCAACCTACTGGCGCCACCTTGCGACACAGACATCAACTCACAACTGTATTTGCTATGACC

ATTATCAGTCTATCTGTCGGTGCAAAAATATTGGGGACAAAACGCTCAATAACGCAAATGTTTCGCGTACTGCTCGAAAT

GCGCTGTTGGGTGGTCACGAAGGCCACGCTAAGGCTTTGTAGTTGAGCAAAGGGTCATCAGATGGCGCTGGCTGTACTGT

GGGTGACGTCATGGTTGTTCTGCTGTACCTAAAACTTTAGTATGTTTACGTACAAATTGTAGCTCTAAGATATACCTCAT

CCCAATTTTTTCGCGGTGAATGCCTTTGCTCTTTCTTTCACGAACAAAATTTATGATATAGCGCTGCTTTCGCATAAGCG

TGTTCGGCACATAGCGACCACGTAGCTGATATGCAGATGTTAAGATACGATGTAACAGTATAGTGTCATTTTTGTTATCA

TCAATTTATTATGCACATTTCTTAGTAGTATTTAGTTACACAAAACGGCACTAGATTTTTTTTTCGTAAACTGTATTACT

GTTTCACTTTCGATACAGAGGTTTTTGT

>CK186795_bb No definition line found

TGGAAACGCATGTGTTGCCCGCTCTACGTCAAGCGTACGGGGGAAGGCTGATTGCTCACGTTGGGAAAAAAATAGCTGAT

GTAACGGCACCGCAGTGACAAAGCATGCTTTCATCATCGCACACATGAATTCTGAATCTAAAAAAAAGTGGTCGAAGTTT

GGAAGTTTTTTTCTATCTGGGAAATCGAATAGGCCTTGATTTTTTCCCCGTTTTGACTAGCGCCAAGTGCCGTAGCGTCT

CTCTCTTCGAGGCGCTTATTAAACGAGTGAAAACAACGTGATTAGGGCTTGCTAAATTATTCTTTCGCGCAAATTTTTAT

AATGATGCCGATTTCCATATGCGCCCTAGTGTTACTTTTAACGGAAAGTAACAGCCATAACGGTTGTGATATTTAGAAAA

AAAATTCTTCAAACATCAGGGTTATGTCAGAAAGGCAAAAGAAAGAATTGATTGATTGATTTGTGGGGTTTAACGTCCCA

AAACCACTATATGATTAAAAGAAAGAATTGAGTGAAGTTAATGTAGACACAAGACATATAGGCAGTTCTAGCAGTTCACA

ATGCGAAGTGGGCTATCACGAGGCAGCTAACGTGAAATACACAGTGACCATGSGCGTCTTACRTGATGGCTACYTTAGCA

GCTGCGAAAGACACTCAGGAGYGTTTTGATTGYGTGTCAGCWTGGCAGCGGCTGAACTGCCGACTWTKAAACAGTGTGCG

CTCGYTACTTGAGGCTACTTTWTGAGAGCTACAMRAAAAAAACGARCAAAGTGACTCATYYYTMATTTTAYTTATCCGCC

AGTTTTTKKGGGAAGATACCGATGACTGCGATAGGCAGTTCATTAGGGCGTTAGCTCTGTGAAGAAGC

>CK186884_bb No definition line found

TTAATGCAGCCCACTTTCCATCTTCCTATAGGCCAATTAACGGTTACGTGGCAAAAAAAAGAGATCACACAGCGATTTTG

GAAGACAGCAGCCTGTACTTTATTTCGTTACTTGAATGAAAGGCGTGAATTTTTCATGCGAAAGCTTTTTTTTTTTCAAC

TGTACAACGCTGATCGCGACAAACGCGCGCGGTTGACCAACCACGCGTCTGATATTTTCCGAGCAAGGCAACATGGCGAA

ATACAGCTTGAACGGAAAACAGTGGACAGAAGAAATACCGGTAACAACAAACAACAACGGCGGCAACTAAAAAGCAAAAA

ATAAAAAACCCAACAACATCCACAAACGAGTATTTATGAAAGTAAAATCACATGTGTTTCCTCTCGTGTCAGTTTTAAAT

TTTGTCAAGACTCCGGCACAGCCTTTGGCGTCAGCGTTCAGCAAACGCTCGCGTACCCGCTTGTGACGTACACTCAAGGC

GTATACGTCATAGGTGACGCACATGCCACGTGACGCACACGCTTGTCACGTGGGAAGGAAAATAACTTAAAACAGTAAGA

CGGCTGTACGGGAGATTATGCACGGTGCAAGAATAAAAAATGTTCTCGTCAAAAGCACTAACGAGTAATAGCGCGTGTTT

AGGCGTTAACCATCGTGAAAGCGGTCCGGGAAGTCGAATTTCTCCAAGAAGCGGCTCGTAACAGATGTGAAAGTGGCAGA

GGATTAGGAACGATGTGCTCATTAGGAACGATGTGCTCATTAGGAACGATGTGCTCAGAACAAGTACACAGAACTGCACT

GTGGCACAGCATTAAGCTGTCCCGCTACAACGGAAGCTTGATGTATTTAGAAAAAGTGTACTTAAAAGAGTACTCGGTAC

TCTACACTGGGAGGGCAGCAAGCCGCCACAATATCAAAGTTATTGACAAAACAG

>CK186908_bb No definition line found

GGCGTTTCTTCGGCCGCTTGTTATATTAGTTTTTGTCCGCCGCTGGCGGGCTCACTTGTCTATCGTAGGTTTCTAATCGT

AGGTAAGCTACTTGCAAATATGTTTTGAGGGAACAAATATCTTTAAGTTTCCACAATACTCTCAACTGTTCTTGCCAGGG

CACCTTCAGAGAGGGAGCCTAGGCTCCCACGAACGAATCGAGATGGATCCAGCACTTCTGAAGGAACGAGATGCGTTCAA

AAAGAGGGCCATGGCCACTCCTGTGGTTGAGGCCCGAAAACGCGACCGTGATTCATCAGCGGCAAATGCGGCGTCGCAAC

CAAAGAAAAAAGTGAAGCCACCGAAGCCAAAAGACACGTACAACTACAAAACTACCTCTGGAAGCTCGCAGTACAACTTC

AGTGTCCTGGCGAAAATTGTGAAGCACATGAAACAAARGCACTTGGAAGGTGACACACACCCCTTGACGCTTGAAGAGAT

CCTGGACGAGACGAATCAGCTGGACCTCGGAGCACGGCAAAAGCACTGGCTGGCAACAGAGGCCCTGCAAAACAACCCGA

AACTTCAAGTGACGCATGACGGCAAGTACTGCTTCCGGCCCGCGTACAACCTCAAGGACCGTAAGAGCCTACTGCGGCTA

CTCGACAAGCATGACCAGAGGGGCCTCGGAGGCGTGCTGTTGGAGGACGTGCAGGAGTCATTGCCCAATGTGGAACGCCA

CCTCAAGGTGCTGGGCGACAGCGTCATTTACGTGATGCGGCCCATCGACAAGAAAAAGGTGCTTTTCTACAACGACAAGT

CCCTGCAGTTCAGCGTTGATGAAGAATTCCAGAAGCTTTGGAGAAGTGTTGCTGTTGAGGGCATAGATGACCAGAAGATC

GAGGAGTACCTGCAGAAGCAGGGCATCACATCTATGCAGGACATGGGGGTCAAAAAAGTGAACGTTGCCCAGAAGCGGAA

GAAGCCATCGTCAAAGAGGGGAAAGACATTTCAGAAGCACAACGACCACATGGGGGGATATTC

>CK186938_bb No definition line found

AGATGGTGAAAACCTTTTAATGAGGTTCACATGGCGCTAGCTTTTGTAAAAGCTAGAAACTGAATTTCCACTAATGCATT

TATCCCAACAGCATCACACGTAGAGTCGATGATCTGAATATGTATGTGTAGGAGGGTCCCCAAACACGGGAGGCGCAAAA

GCCGCTGCTGCGGCACTTCGTTTATTGGCACCACTGCAGACATGCAATATTCCCAATGTGAAGTTCCGTGGCACTGATGA

GATGAGAAAGGATAGTGTACAACAAAAACATCTGGAGAACGGGGAGCACAATTGAACTCAAAACAACATGTAGAGCGGTA

CAAATTAACAATGTCGGCAGCTGTAAACGGTGACTACAAGTAGTTCAGTATGCGCACAGCTCAACACACTATAGTCTAGA

ACACCAAAGCAGTGCATTTTAATTTGCACAAATGGCATCTGCTCACAACACGTGCTTCGAGGTAGTCCGAATTTCCACAT

GCGCAGCTAGGCGTGATGCGCTATTAGATGTAGACATCACCCGTGCAGTACGAAATGCACCGATTGTGTGTCCCAGACAG

GCATGTTGAGCTGTGTTTTTTGGAAGCACCAAACGTGCCGAGCATGCAGTGAAACTGATGGCACATGGTGCCGGTGTATC

GGGGTTTTTTGATGAGGCGGGTGCGATGGTCAACGTGAGGAGCTGGAACTGGAGCGTGCGTGGCGCCTGGGTCCTCCGCY

CGTGCCAACAGGGGACCGCGAACGGCGTGAMGGTCTCGGAGAGCGGTGTCGGCTTGGCGGGGGACTTCTGAAACAGAGTC

AGCGGACTGATGATTTCTGCTTATGGGAGTTGCATGGTAGCTGTGAAAACGGCAGTGCAACGCGGTTTAGTCTTAGAAAG

TTACATACCA

>CK187086_bb No definition line found

GCAGCGGATGCGGCCGTGCGGCAGCCTGACCACGCTGTCGGGTGTTTTCTCGTTACTTTGTCGCCTACAATGCCGCGAAC

TCGTGAAGTTATAGAGCAGCTGCCGCACGCTGTACATCACGCCTCGTGCCGTAGCATTTGATATTTCGTATACCTGGAGC

AAAGGACTGCTGCCTCCACTAATGCGGCCAAGATGCCAGCACCCCCGGGACCCAAGGACCCATACCATTTCGTCAATTTC

ACCATGTTCCTGTTTGGTATTGGCTCATTGCTGCCATGGAACTTCTTCATCACCGCTGATGCASTACTGRAGGTACAAGT

TCCGCGACGTGAACGCRTCGGGMGAGGTGCACACGAAGAGCGACATGCAAGCAGCCTTCACCAGCTACCTGGCCATCGCC

AGCAAGGCACCCTACATTCTCTCGTTGGTCCTGAACACCTACCTCAGCCATCGCATTCGACCCGCCGTGCGCATCGGCTG

GCCTCTCCTGGGCTGTACACTCTTTTTCGTCGCCACGGCTTCACTAGTCAAGGTGGACACCGATCAGTACCAGACAGCAT

TCATGGCCGCCACACTTGTCATCGTGGTACTCATCAACATATTCTGCGGCTTTCTTCAAGGAGGCGGCACAGGTCTGGCT

GGTTGCTTTCCCGAAAAGTACATGGCTTCCAACCTCAATGGTCAGGCAATGGGTGGCATTTTCGCCACCGTAGCGCAGAT

CTTCTGCTTGCTCGGGGATGCATCGCCCACAACGAGCGCACTGCTCTACTTCCTGCTCGCCGTGGTGACGCTCATATTCA

CCCAGATATGCTTCGCTGTATTAGTGAAGATGGACTTCTACCGGTACTACACATCTACGCAAGCTGTGAGTTACAAAGAC

TTCGACAA

>CK187143_bb No definition line found

CTCGGATAGTTGAGAAAAAAAACAGTGCTTRAGATGAGACGAGGTGAAGGCAGGCACAGCTCAATGTCTGCGTCYCATTC

CATTTCGAGCAGCATTTTATCCATGATACACTGTGCATCAACCAGCCTRTCAGGTACATGCCCCCCATAGCTGTTTTCAT

TCAGCATTAGCTTTGGYCYTTTTTTTTTCTAAGAACTACACCTTATGACAAAGGCACCAGAAATTAGGGACCAACAAGGT

CCTCGACAATTTCTTCCTAAATTGTCTAGAATACAAAGCTTTCTAGCAATGCACCAGTATGCCGCAGGCATTGTAATCTT

TAGTGTGAGGTCCAGGAAAGTGACATATGGCCTTCATTTTCTTCATTAATGATCAGAAATGTTTTCACTAAATAAATAAA

CTTTTGAGACATGTTGTTGATTTGAGCAGATTTAGTAACATTATGCGGTGTCTCTGTAATTGCTGGATAATCACCATGCG

TGCAGATAATCCTTTTAAATGTGGAATGCAGGGCACACAACTGAGAGAGGAAAAATATTTTTATTGTCAAGTGTAGTGCC

AAACTGGTCATCTAGCATTTGTTTTTGAGATCATCATTGTATTACACCGCTTTAGTTTTGTGTTGAAGACCAACTGCTAC

AAAATTTTAAACCGCATTAATGTGCCATTTCATATAGTGTAGTTTAATTGAGGTGCCAAAACAGCTTTCTATACCGGAAG

TAAAAAAAACAATAAAGCAAATGTTACCCTTTCTGGATACTGGACACAACTAGGGCACAAACCTGCTGTATAGGGGGTAT

GCACTGAGCCTTAATCTACAAGGGGGCAACACTATCCCCAAGCCATTTTGAATGTCCATTCGTACGCACGTCCAGCTCTT

TGGCGTGCAGGAAGTGTTCCAGAGGTCACCGGACATCTAAGTTGCCATAGGAATCATGAACACAGCACGTTCAGCGGGGT

GCATGTGGCAGCGCGCGGATCGC

>CK187184_bb No definition line found

GCAGTCCCGTATCGACCGAGTCCTCGACGGGGCTCTGAAGAACGACGCCAGCATCAACGAAGCGATCCACGAGGCCAGCT

CGACTCGTCTGCCGAATGAACACCCCGACAAGAAGGCTGTGACACCATTCCCAGTTCCACTGTTGATCATTGGTTCCAAG

TACGACATCTTTCAGAACTACGAGCCTGAGAAGCGGAAAGTCGCCTGCCGCTACCTCCGGCACGTGGCTCATGCCAATGG

GGCTTCCCTCCTGTTTACGAGCTCCAAGAATGAGTCTCTCATTTCGAGGCTTAAGACGAACCTGGCATACCTGGCCTTCG

GTTCTGGCTCGGGCCCTATCAAGGCTTCACACATGGATTATAACAGGCCCCTGCACATAACGTTTGGAGAGGATTCGTTT

GAGAGGATTGATGGTTCCACGCCTGCCAATACGACTCAAGTTTCAAACTGCTACGCGGTAGTGAAAAAGGCATTCACAGA

GATCTTTCCACAAGTGGAGCAAAAGATGACCATTCCAGAAGACCCGTGCCGTGACCCGAAGTTCAAGGAAAAAGACATCG

ATCTCATGAAGGCACAGAAGGAAGCTGAACTGGCCGAACTTCGCAAGAGGCGCCAAGACGAGGAAAGAGCGAGRGAACTG

CCGGATTTTGACTAGGCSTGGTCTTTGAATGAGCGAGAATTTCRTATAGGAAAATATTTTATGCAATCATGTTTCTTGCA

TTCTCCCATCTGCACTACAGAGCAGCACGCCCATTTAGTCTTCGTGTGGTCGTCTGTACTAACTTCATTGGTGTTACAAA

TCCTGACCATGGCATTGGACGACTGAGATTGGTCACTCAAAAA

>CK187193_bb No definition line found

CTGATAAACATATTTTATTCACTTATGAAAGTTTTCTCTTGAATGTAAACATTTATGAATTGTGAAAAGTAGCAGCCTGT

TCTTGGTGTTAGGGACAATATATAGATGTCAATATTTGCTTTGTGATACTTAGTAAAGAGTTGTATTTTTATCCATTGCC

TGATTACCCACTTAAATTAAAAAGTGTGATACATCTGAATGATAGCCATTCATTAACACTTTGATTAAAATTTTTATTCA

CACAGCCACATGCACATTGTAGGGCATGCCTAGTTGAACACCAGCCAACAGCAGTTCTGTGGGCAAATATACAGTCCCTC

CCACAACAGTAAAGTGCTTGTTGAGCGACTTTCATAGACTGGGGTGCCACATGCCTCTCTACGTTGTGTTGTAAAATACC

CTATAGCCCATATCACTGGACTGGGTGCTGCTACCACCAGCTATTACCATCATGCCTTTTCTGCAGTCATTTAAAAGTGG

CCATTAAATAATTCTTTTCTTGCTCGAYGTTTGTCCAGTAAAAAACACACAGTGTAGGCACCTACTAGATAGGGAAACAC

AACAATCATTRTCRTCATCTGTGCATAAACACATGCAACATCAGTGTCACCAAAAAAATCAAAGYGAGTGTAGTTMTAAG

AMCAWTAAACTGACACTTGCCACAATTTCATGTGACGACATCACCAGATGTCTTTTCACATT

>CK187194_bb No definition line found

GGCTTTCTAAGGCTAAAGAGAACAAGTGGCAAGGATGTGAAGCTTGCCTCTTCACATCATGGCTGCTTGCCGCTTCAAAA

TATTTAGCTCTGCCTGTAAAGAACAAAATTTAAAAGCTCCTGCGATAGAWWGCTCTCAGTYCRATGCTAGTGCATATCTA

AAATTKACTTTGTGGCCTTGTGAGCGATCTTTTAAGAAGACTGATTTACAAGGCTTTGTCGCTTAGAGGGGCTTTGCAAC

ACTTTTTCAGCGGGGTCGGGAAAATGCTRYTGGTCGGTAGTCGAGGCACCTGAAAACATGCAAGTTATACATTATAGTTA

TACATTATAGTGCGGCAGACGCTCTGGAATTCACAAATTATTATCAGTCAGCTAGAGATAGCTCAGTCTTTTCTTCACAA

ATGATGCCATAACACAAAGTGACGTCATTGGCGAAAGCCCACGGTCATTGGCTGATTTGAGGAACATGAACTGCATAGTT

AATATGGCTGCCACAGGGTGCCTCAATGTGTCCCTGTTGTACTCTTTAGTGCTAGGAATACATAGTGTGTTAAGCTGTGC

ATTACAAARRAAAAAAAAAGAGAAAGTGTGTGAGGTCATGACGTGTGCTGACAAACAGACATAGGTTCTTGCCCCTGGGT

CATCCCTCCCTGCATAACTTTCAGTGAGCTCGTTAATACAAAAGGAAAGAGAAAGCACTTAAGTGTTCAAGAAATATCCA

TAGCTCTGTTTGTACTTGACAGATTCTAAAAATTTTTGCGGAAGTTGACTCGGGAGACAATAAGCTTTTAATCAAGTCAT

TTGATGCTTACCTAGAAAAGTGTTGCAGAACCCCCTTAAAGACAGAATGCTATCCAATTCTGCCGTTTTAATGCTCCCAA

AGGGAACGGCTTTAGTGCATTACGGTCTGAG

>CK187195_bb No definition line found

ATGGCATAATGCAACTGTGGGCACAAATGCTTCCACAATATTTCCTCACGGCTTTTGTTTTACATCCACAGTTAGGAAAG

CCCAAGGGGTGCAGACAAAGAATATTCGCAGTGCAATTCATTGGAGTGCCTTAAAGAAAAGTCTTCAAATGCATACAAGA

AAGAAGCGTTGGATAAGCAGCTTTGCTTCTTTTTTTCCTTCTTTTTAATCATGGTCTTCTTTGATGCTATTTTTAGAAAT

GAGCATGCATTCAAACTTTTGGAACTTGACTGCTTCTTTCGAGACTCCTGAGCTCCCATATGCTGCTCCAAAATAGTGAG

TGTTACAATAGCGAATTTGGGCCTTCAATTTAAATCCACAAGGTTATGTCTAAGACTCCCCAAATAATGTGCGTGCTTAT

GGTAATTTTGAAACCTCTAAATCTTGCAAGAGTTAGCATGTCTCTTTAAGCATTTAAGTCTAGACGAGTTCACCTTCAGT

GCACTTCTGAACATTAACTGCACAAATGTTGCTCAGCCTTGCTTGTGCATTTTTTATCTGTTCCTGCCCTGAAGGGCACT

ATATTACAGCAATGAATTGCATTCTGTAGGTATAGCCACCAGCATAATGCGCTTTGAGTGATGCCACTTATTTTTGTTTT

CATATACATCACATTTTGGTTAGTGACTAGTGCAAGCGTTTTTACAGCAGAAAAGGTTACTGTGACGGTGTCTTCCATAC

ATGATTTAATGCTGCTTCACAAACAGCTGCACTCAGCCGACATGGTCTAACCTTTCGAATTCATTGCAAACGTTTGATTG

CTTTGTGAAGTTGTGCAGTCAGTAGATTGTTTATTGAAATTTAATTATCTAAAAGATATAGG

>CK187216_bb No definition line found

GGAATCGCTGCACAATGCAATGTGCACTGCACAATTTACCTCTTTACAGCAATTTGCAACACACACAATCAACATTGTGC

ACTTCAAAACTTCATTTTTAATTTTTAATATGAAACGTTATGTACAGCATAAACATCATACATTAATATAGACAACACAG

CAAGAGCCTCATAATGTATACATAACTACACAATAAACTTATTCCTTCTGACATGTCAATAGTACATCCCCTGATTATTT

GCTTTTTGCATGATTTCTTAAAAACCGCTTTCTATGCCACTGCACCTGGCCTTGAGGGAACGAAGGGCAAACAAGAGATG

TGCGGGCAAAGTACTCTTTCTCCCCAACGTATCTATAATATACGTGAAAAGGACAGCAAATAAAATAACTGCAAGGCATA

CTGTCCAATAATGCACAGACTAACACCTTGACTGGAAAATGAGGCTTATAAARGCATTGTGTATATAAGCCATGAGCCAG

CCTATGATCTTACAATGCTGCAGCAGTGGCCATAAAGGAAAATTCCCAGGAGATGAGGGGACAATGTCGAAATGTCGACA

AGTGCTTGTACCCCCAAAAAAAAAAGGGGGGGAGTAAACAAGCAAGGCTGAAACAGAGCTATCTACATGTGACAGAGATA

GCTGCCATGTTTGTTTCCAAGAACGAGTAGGTTCACATTAAGCAATCATTCACTTGTCCATAACTGCACAAGTGGGTACA

ATGTTGACCTCGGAACAAAAATGGCAGCTGCATCCAGGGGGATGCTAAATTTGCCCAGCTCTACAAAGCACTGGTAGCAA

CACACAAGTCAGTGGTGCTACAGCAGATACTTCATGCCGGATGGTAATGGAGAAAGAAACTAGAGCATAATGCTCATGAG

TTCCTGACTGCACCTGCAGTTCTCTC

>CK187263_bb No definition line found

TCAATCTAGCATTCTATAGGTTACTCAAAGGAATACAAGTTGACTAATAAGTATGGAAGTAAAGGGGCAAAAAAAGTCTA

CGCAAAAATAGCTACAGTTTGCTCGTTCATAAAACAAAACAATAGGTGTGCAACTTGCTACAAAGTTGATTAGATATTTG

AAATTTGGCATAAGAACAAAAATTGTATGTCCCTATAGTCCGATACAACTTCAGAAATTCGCTACAACACTTTTCCAGGT

AACCATAGAATGGGTTCACTAAAGCAGCTCATTGCCTCCCGAATTCCCCTCCCCAAAAATTTTTAGAATCCTTTCAGTAC

GAGCGGAATTACGAAAATTTGTTGCATGCAGCAATTGCATTCTCCTTACGCTCGTCCCAACTAAAGTACTACTGRAAGCT

AAGTAGGGAGAGATGGCACGGGTAAAAAAATAAGTCGCACATGCCTCGTGACCTTGASCACTTCTTTTTTTAATGCGCAA

CTTTTCAGCGCAATTTCACACCAACACTTGGACAAGTCSCGGCGTCCCCGTGGTGGCCCTAGTAGTGACAGAACGTGYMA

TGCTTAAATCAGCCAACAGCTGATACTTGGCCCATGACGCAGCAATTTTCGAGCTTGTAGCATCATTTTTCTAGAGAAGA

GAAAGCGATTGTTAGCTCAGTCTAAGTTCCAAACCGCGTGTTGTGCTGTAATATTTGGCACGTATGTTCTCGGGAGCCTC

AAATACAGATCGGCAAGGCTTTCTGACAAAGCTCAAAAAGTGTTGCAGGGCCTCTTATAAAGGCGGATGCACACAAGCCA

ACGCTAATGAGCTTGTACTATGAACATGTTCAGTAGAACGGCCGGTGACATTGCCTTTGAAGATCATTATGTCGGGGAGC

GAGACTATTT

>CK187488_bb No definition line found

TCCACAAACATAGGATACATTTTAGCACACGATTACCATGCAATGGCAAGAGCAGTCAAATTTTATTTTCTCCATGCTAA

AAGAAAAACAGATAAAAAAAGAAATGCAGAACGACTGCTTTGTGAGATCTGTGGGTTTACTAGGAACCACTACAGCTGAA

GCATTGTGTTCATGAGTCCACCAAACTTGGGTGTTCTAGGTTGTGCTACATAAYCAGAATTAATKAAATAAAGGCAAAAA

GTGGGGAGGTGGTATTTTTGGAAAGAACAATTTCCAACGTAGCATAAAAGATAACTGGTTGTGACATTTTCTTTAGACAG

AAAATAATTTAACGTGACACATACAACAATTATGTCCGTTGGTATACCTTTTTCTTACAACTCATGATTAGCATAATAAC

AGTAGCATTACATAAGCTAAAGAAAAAGTGAATCATGCTGATGTGTATGAAAGGTTTTGATGTGAAAATGTGCTCTGTGT

GTTGCACCACAAAATCAAAGGCCAGACATTTACAGAATGGTGATGTTGCTTTGGTTATATAAACTGTGTAGCTCAGGCAG

CTGGTGCATTTGCAGCAATACAAAATGACAGCGTTTCTGTCATGTTTGAAGTTCAGTCACCTGTTTCATGACTTTATATT

GACATTACCTCATTCTGACGGTGGACTACAGGACAGACAAAACATTGGAACATTATTTAACAATGTCCACAATTTCACAA

GATTCTAAGCTGTATGTACAGCTAGGTTAAATGTAGGCTCAATCATGTTCACTTTAGCCCATTATCAAACAAAGTTCATA

CCTGCAGTTTTATCCAAATACTAGAAATGCAGCTCCTTACTTTAAGAAAAGAATTTC

>CK187489_bb No definition line found

GTAGTCCTGCAACATATATAGCTGCACTGTGCATATTCATTGTTCACTGAAAAATCTTGTACATTTGTCATAGGTAAAGT

TACAAGTTGTACTACACCTACATTTATATATATATTGCATAATTACGTACAAGATGGAATGTGATAACAGTGTCACTTAT

AAACCAGTGCTCATTTTCTGTAGTAGTTTCATAAAAAATGCTTAATGAGTATAGGTATACATGTATTACATTGGTATTAG

AGTTGGTTAAGCCGCCCGTGGTAGCTGAAACAGTTTTTGTTTTTTTGTGTGTGTGCATGGATGCATGGATGTGCACATGT

ATCACGTGCATGCATACCTGCACAGTGGGTCATGTTGATAATTATATAAAATGTATCATTCTCTATATGGTGCTTCGCAC

TGTCACAGGAATTTGGTTTTCTGAAGCAAAAAGTATTGCAGAATCTACACATACTAACTTGACTGAGAATTGTAATTGCT

CTGAAGCTTCTCGTATATAGTTTTCATATCACCTGCTCAAAAGGTAGGGTGTATTTGCATTACATTGTGACAGTAGCAGA

TCACTTGTACATCACTGAAACACTTTCCTGGTCAAGTTGTTGTATCTTTAAGCTGATCAGCAGTGTTAAAGTACTACATA

ACTTCAAGGAGAACTGTGATACAGACTTATAACATCATTTTTATAGTGTTTGAGCCTGCCTGGAGCTGCKCACAGTCTAT

AGTGCTACCAGCTGTGACATTCAGCAGTGGGAGAAACATGCTTACAGCCAGCACTATGTACGGGAAGAATTTTTTTTTGT

GCTGATTATTATGTCTGCAGCTGTGGCGTGAACAATGTAGTTGTGAATGTAGGTTCATCTGGAGCACCTTTACATCATAA

GCAATTTTTGAAATTTCTTTCTTAAAGT

>CK187512_bb No definition line found

GAAAAAGCCATGGCTGCGTCTCACACATGGCGTGTTTTGTTAAGCTCTTCCACGTTAGTCGCTGCGCTGGTCGTCTTATA

CAGGACGTCTTGGTGTTTCGCACTGGAGAAGAGCCGACCTGACTACACAGTCTATCACAACCTATCCGCTGTGTACGCCG

AAGTCGCCGCCTTGGCTGAAACTTACCCGACCTACGTTCAAGTTGATCACAGGTTCAAATCGCGCAACGGCCTCTCCCAG

CTGGTCGTGAGGCTGGCAAACTTCTCCGACAGCACGTTGCAAGCGGCACCGCAGTTTCGAAGCTACAAGGTTCGCGCGCT

ACTTCTGTTCGGGCAACGTGGTGGTGAGCTCGTGACTGTCGAGAGTGCCTTGCATTTCCTGCGGCAGCTCTTCAATGGAC

TCTCGGCGCCCAGTCACACCGTCGAAGGGGCTGTCAGTCGAAAGCTCCTGTCCAAAGCCGACCTACACCTGATAGTCCTG

GTGAATCCTGATGGGTTCAACCATGCCGAGCGAACGGGCGACTACTGCTTCGACGGGACAGTGTCGGGGGCAAAGATGAG

CTCTCTTTTTGCCTGGGACCTGGATAWGCGCTTRMACKCCGAGCCCAAAACCCACGTCTTGCTGAACTTGAGCCGGGCAC

AGCCGTACGACGCATTTGTTGCCTTCCATTCGGGTAGCCGGGAAATCCATCTACCTTATGCAGGGGTGCAGCCGGGGATG

TTTGACCACAAGCCAGCCAACTTAGAAGCGATGACAGCATTTGCCAAAGAAGTTGCATTGGCCRTCAAGCCGAAATTCGT

CTACGGGACAGGCACAGCACTTGTTGAACAGGCAGCTCAACGGAACGGCGTTGGACTTCATGGCTGGAGTGCGCAAGGTG

ACGACATTCCAAGAAACATTTGAAAGAAAGCAGTACTTTTATTATTCAGTGAAACATGGT

>CK187709_bb No definition line found

CCATCAGAAGTCAATATGCCGAATCTCCCACTACAATTATTTTCCCAACATTTCTTTAGCATAGTGTCACCTAGTTGCTC

TTTGTGTAACTCTTCTCTTTGGCTTAATGGCACATCCCTCTCCTAATTCATTAATTTATTTCCAACAATCAAAAATGTAC

ATGTTTCCCAGAAACTTCCTCTTCCAAAATAAGAATGCATACCACTTCTTCTGGTTTCTCATCTGATTGTTCTACCTCGT

GAGCARACTCGGCYGGTGCTCTACARAGTGCCARARAGCTGTTAACCTTGAACAAAAGTTTCCAATCTTCCTTGGTCAAT

AAGCAATCGGTGCTGTCAATGAGCTCATTGGTTAATACACARAGTAGGTGAACACTCAAAGGCTCCACAACCTCTTTCGG

GCAATTTCATTTGATYGGCGATGTAGCTAAATTTGTGGTGTCAATGAGCTCATTGGTTAACACCCYGAGTAGGTGAACAC

TCARATGCTCCACAACCTCTTCTGGGCAATTTCATTTGATCAACGATGTAGCTAGCCTTGCCTCGATAGTTTTAT

>CK187710_bb No definition line found

ACAACGTGCAGAGGGACGTAGCAAGCCGTTTCTGCCTCAGCAGCTAGCGCACCTGGACTTCTTTAAGTAGCATCCACGAA

GAGGCATAATTTAGGAAAAGAGAGACACTGAGCGGACCAGTTATCCAACACAGCTGTAGTTACTCACTGGTTTATGCGTT

TTGAAACTTTGAAAGTCTTGTTTGTGTATAAATGCGATGTGTTGCTGTAGTTCTTAAGCTCGGGGCAGGAGGATGGGTGA

AGCCTTCAAAACAGGCATGGAGGCTGCTAGCAACAAATACTTTTCTCACTGCTGCAAGATCTGGGCCTAACTGATGTGTC

TTGCAGCTTCATGCCACATGCTGCAGACCATTCCTTGTTTGTTTGTTTTTTGTTTTGTTTTTTCACTGGGCTACCAGCAG

CACTACTTACTATTTTTTAATATTGCAGGGGTGTGAAACCTTGAACATGCCGTGCCGAAATGCTTTACCAGCCTTGAAAT

TTTTCCAGTTGCACTAATTTTAGCAATAGACACAACCTGCACTTTGGAAGGTTATGTAATACAATCCTGGTGTGCACAAA

AAGATGGAAAATGTGTAGGCACACTTCATGTTGGGTGCCCATAACCTCTTGTTTTTTTATTTTGCTAGCTGCTGTGTTGC

TCAACTAATATGTTTCAAAATTGACTCATAAGCTTTGTGTAAAGTATGCTGATATGTTGACATTGTGCCACAGATTTATT

CAATGGAAATATCAGTCTTTTGCCTGCATACGAACTCTTTCGATTTGCCTAAGAAAGTCCAGAAGTTTTAGCAATGCTCC

TGATTGTGTGGGTATGGCCATTTTCGGATTAACAGCCACAACCCCACCATAAAAAGAAAGAATGCAAGAGAATGGTTCTG

GAATTTTCACGTCTTGTTCTGTCGTGTGCAGAACACTTAAGTAATTTTCAGGAAGTCTAGTAGAGCCAGGCAAAGTGCAA

TTGGTGTCTGCT

>CK187786_bb No definition line found

ACCTAGCATGACTCACTTGGGCAGATGAATGGCAAGTATGCCTCAATTTTTTTGGGGCGTGGGGGGGTGGGGGTAGATTT

ATTTATTCTTAGGTTGTAACTGACATAGTTGGGCAAGTGGAGCGTATAATTATGTATGTAAACACAAAAAGAAACAAAAA

CTTTCATTTRAACTGCAGCTGTGTATCTTTCACTTTACGCGTTACAAAAAATTTTTAAACCTACCGTATTTACCCACGGT

TCTGATGTATATATGTTTTATGGGCTCCAAGTTAACAAATCGTGTGTGTCACCAATTTAACCCATGCCTGATTCACATAA

ACGGAACATAGCCTGCTGAATTTGTATAATCATTGAGAGGAACAAGAAAAAAAAGAAAAAGACTGTAGATAGTACAAGCA

GTGTGTCACCATTACTGTTCCCAAAGCAGGCAGCCCACTGTGATAACACTAGATTTGATCGACTGTCAGCTAAAGCCTTC

ATAGTTTTTTTCATCAGTAAAAAGTTCAGTATGCTGTGTTTCAAGGTAGCCAGAGACCTAGCATGCCAAGTTTAAGAAGT

TCCGTTAAGTCATTGCAGCTAAAGTACGAAAAAGACATTGTGATACGTTGTATCACTAATACTCGCACTCGGGTGTTGGA

ACTTGGGCTTGTAATTCAATATTGAAACTTTGTCACGTAGTTTCTCTTTTAATTATCAGCTTATGTTTATGAAATCAACT

ACATCAGATTTCTGAAAGAATAATTTCTTAGTATAAACCTATGAAAGTGTCACTTTAGTGCCTCTTAGATGTAAATTGTT

CTGACTAGTAACTAAAAACGCGCCTTGTACTTCGATTAGTGATATGCAAGCTAAGAAATGAGCTAAGCAGATTTGCGATG

TACTGACCAATACCTAGTAGGATAAAAAGGGCTTCGGCAAAACGGGGACAAAACCCACGAGT

>CK187864_bb No definition line found

TCATCATCATGCTTATTGTGCAGAGCTTCGGCTGCATCTTCCTGCCTTTCTTGCAGGAGTACGGCTTGTCGATGAACATG

GACGCGTTTCGCGCTTATTACGGGGCGCCCGTGTTCATTGCTTGCATCGCGATTCACTACGCGTTGTTGGTAGTGGGCAC

CGCAGGCATAGTCGCCTGGTTCAAACTGGAGCGATCAAGTTCGTCTGGTTACGGAGCGCTATGAGTGCCGTTCCACTTTT

CCCCGCTGTACGCACTGTCGACAGCGTTCATGTGGCCGCAAAGCTATAGCACCGCGTGGATTTTGAGTTGTATAGCATGC

GGGATATTCTGGTCGCCCGTCATGTATACTTTTTGTCTGTTGTCCGCAAGGTGCGATTTCGTCTCTTTGTTGTCGCAGTA

YTCGAATTGCTGTATCATACTAACTAGCAGGTGAGCTTCGTTTACCCATGAGTATGAGCTCCTTTGCACTTCTGTTCGCG

ATGAGCCAGTCATGTTGTAACTTAAACGGCTGCGGAGAATTTCTTTTCCGCGTAATAAAAAAATGCACGTTGTTCCAGA

>CK187869_bb No definition line found

CATTCAAATCGTAGGTTATAAGGAAAGTATGATGAGCATAGCCTCTTTCTATGGGGAATTTTTTTTTTGTTTTTTTTTCY

ARAAGGGCTTGGGCATGCYAAGAGGGCCWGCATACAGTAACAATCTAGCCTGGCCTGATACTTYCCATTATTGCCTAATG

TGAACTGTGAACTCTTGAGCTACAGGACATGCCAAGCTAACAAGAAGTCCAGAGCTCCTGTATGGCTTGGCAATGTTGCT

GARATGCCATAAACAGCAGCCATGTGACGGGGACATTATAAAAGGAATATATCACATAACACCCTGTCAAGAAAAGGCTG

TTGCCAGTGTTGTGCGAGTATCTTGGATACGCAAAAATCATTARATACTGCTTGATAGATAAGCACACCTGTATAATAAA

AAAAAAAACACCACTGATAAAATGAAAAAGTTGTAAAAAGGTAGCTGCACGCAAAAATGCATATTGAACAGCAAACCATA

TCTGGCTCGCACAGCAAGGACATACTTTGGCATGAAGGACTGACTTCAATGTCTGTTCTTGCAGGTGATCATCTGCAGTA

AGTAGCACACATTATGTATCTCATTCGTTTGTGAAGCTCGTTGGTGTAAAAATAGTGTTTTGATTTATATTAGTGTAACA

CAAATGTAAAAATAAATAGATCACATAGATATCTTTCACAGCAGATAGTTAAACAAACACAAGTAAAATCCAGGGCAAAG

AGATTGTGATGCATGTTACATACAAGGCAAAAAAAAAATGCAGTGTATAGGAATGTCATTACATAATGCAGTGCAAACAG

AGTTCTCAAGGACTG

>CK187874_bb No definition line found

GGTTATTGTCCTATTTTCCACAGCTGGTCAGTGGAAATTGAAGAAAAAGGAAGAGAYCCARRAGGGTATGGCTTCCTGAA

GTTCCACGAGARGTTTATATGCAGTACTTTAGCCACTAAAAGTTGGCRTTTGCTTGCATTTTGAATTTTGTTYTTTGTGC

CACAGTGTTTTCTTTTTCTGTGCGTGTGTTGATGTGTTCATTTATTTTCAGAATGAGTGCGGTATTATGGCTGCGTCAAT

TCTTTCATTGCTTCGATGACATGTGTCCTGGATGCGGCGTGTTTCATTCATCCATGGTTTTACCATACGAACAAAATTGT

TGGGCTGATTTGTGGTTATCGTTATAGTCTGTTGAGTTTTTTTTTTTCTTTTTTGCATGGTTGCGTTTACTTCAAACTTC

CAGGGCAAACCACTTCTTAGTGCAGAATGAATGAGACAGTATGTTGTAACGTTCCCTTTTCCTTTTGCCAATGTATGTTT

GTTTACTTGTTGGACATACGTGCTCGATTCATCTTATTCTGCATCTACTCTTACCCTGTGAGCCGGTTGTGTACATGTCG

CATTCTCCTGCGAACATTATAACATGATTGATGCACGTTCCTTAAGCAGGTGTTGTGCTATAACTTCGATTTCTTCTTTT

TCTCTCAAGTTTTTTTTATTAGGTTAGGCATTTTGTGSACGATAGTTCCTTTTGAGACTGAAATTGTGTCGGGCATGTGG

TTGCCTGCATCAACGGAAACCTGCCTGTATAAGTGAAGTGCTTTGTTTCTGGAAGCACCACTAGTTGTTAAATGTTCAAG

TTTCTTGCTACATCTTTGCTTCGGTTGGGATACAAATGCCATTTAGATTTGCGGCTGCCGTTGGGATTAAATCAAGATTC

AGCCAGTAAGTGCTAGAACCGTAGTAACTGCTACTTTGGTCTCGTAAAGT

>CK187990_bb No definition line found

GACCCTACCACAACTGGAGAAAAAAACTTTTCAACAATGAAAGCTTGCCTGTGGATTACACTCATTTCCCTCATCGCCGT

ATGCTATGGCGCAACAATTCAACCACAGGTGACCAATCACAGGCCTGYYTTTTCKRMAGAGGCTGAGACGGAAGTGCTCG

AAAAGACTGGAAAAACYCTGGAAATCGTGGGGCGACTATTACAGGGCGAGGACGTCGCCTTGACCGATGASGTGCAGGAA

GACTTGATACAAAGTCCTTAAGGTTGCGTCCGCCAATGGTGAAGTTTTGAGTGAAGATGGCTCTGAGCACATTTGGCCGA

TCATCATTCGTGGCGTTGCCCAAGGTGCCATCGCACACGTTGTGCASAAGAAGCTGAACAAAGGCTAAACATAACGTCAA

GTCAGCAAGCGATATCGCCAAATATTGTTTGTTAGATGGGATATYRAACGCCACCGCTTGACAGCAACTAAAAATAGGGT

TTAAAAGTGATTCTTAAAAAGAATCGTATTTGATCACTTGAAATAAAGAGATGGTTGGTACAATG

>CK188002_bb No definition line found

ATTTGTAAAAACAAACAATAACAATTTATTGTTCTACATGCCACAACCATATGACAGTGAGGCCCATTGTGGTGGGGGAC

CGTGAAATACTTTCGACCGCTTGGATTTCTTTAATCTCCAACTAAATCCACGTACAGTGTCCTAGTATTTTCCCCCTATT

TAAATGCATCCACAATGGCAGGGAATCAAACGCACATACCTTATCTTAGCAGCATGAAGCGTTACGATGTTAAACAAACA

TGACACGTTATATAAACTGAAATGTCAAACATACCAATTATGGGTTAGGAAATAAAATTGAACAGCACTGCAGGCTCCGA

AAACTCAACACTGACCCTGAAAGGATCAATGCTGAAAACGTATATGCATGTCCTTTGCAAACATTTTCAAGACGGTCCAT

GAGATACATGCATGTCATCATCGATGTTTAAAAAGTTTGCGTAGTTCAATTGTTTCTCTAGCTTGGCATGTGATGGCAGA

TTTGGGAATGCAAGAAGTTTTTTTTTTTTTCATGCGCTGTTGTTTTTCAGTTTTACTACAGCGATGTGCCGGCAATCTCT

TGTGCATTCGATCGCGCACAGCCAACAGTTCCGGTATCGCCCTTTGGGTGGTTCTTGCTTCTTGCACCCGCATTACTGAT

GGCTATCTGGTTTAGAATGTCTCAAAGGATGGCAGATTTGTTACTCTCTCTCTCTTTTACTGCTCCCCAGAGCAGGCGTT

GACTTAACAAACAATGCTGCTGTAGCTGCACAGCGTTTCCAGATTCGGGAGTCTCAAAACTGCCTTTCATGTCGTTGGCA

AACAGATGAAAGATTATCGG

>CK188175_bb No definition line found

ATATAATTCAATGCTGAATATCAATACCTGAACGTTTATAGTAAACAAAAATTTTTTTCTTTTTTCAAGTCATGATTGGC

ATCATTACGTTAGCCGCCAGACTATAAGAAATCAAAGTGATGAGAAACAGATTACTAATGKGCGCTAAAACTTTATTGGT

CCCCTGCATATATAGGCAGTTTACGCCAGGCATTTGATCATATGGCACTTCTTGAACTTCAGGTGCACGAGCACGACAAA

CTTTTGCGATAGTCCACAGGAATGTTATTTTCGACACAGGATGTAAGACGCTCTGTAGGCTACCTGAACCTCCGGATCAT

CTTCACCGGGCTTGCCAAGCTTCGAGCGAATTCTCGTCTCTAGCCTTTCCAGGGCCTGCTTGGCACGCTCGGGTTCCTTT

TCTTCCATCCGTATAACAAAACTCCAAGTCCTCACGCACGCCAAAACAGCCGACAGTGTCGTCATCTCATTGACGACGTA

TTCCTTCTTTCGAACGTCACTGAAGGGCACTTCTGTCTTCTCGTAGC

>CK188188_bb No definition line found

TTGCATTTCAATAATCATCTTTATTGTCTGTAGTATATTTATTTTATTTTTTCATCCTARACCACAATGAGGCGTTGCGG

TTACAATGATTTTTTAAAAATCAGCTGTTCCTGTTGGCTTCAAGACAACCAGCAATACARARAAAAAAAAATAACAAAAA

GTTRCAATCTTAGCAGCTGARAAAAAAAAAAAACATGGTACTCCAGCAAACCAGTTGCACAAACTGCAGGTTTGGATGAA

AGGATGTACCAAAAAAACAATMCCCTTCAAGACAGTGGAGTGCTGTACAAAAAGCTAARARAGAACAAAAAAAAARAAGG

AAGCAAGAGAGGTTTTATATATTGCAGTTTAAAAATGAGCAGCAAGTGTAACAGTSSATGTGCTACGTGAAAATTCATAA

CACCAAAAACATGAGTGCAATTAAAACTTAGCCATGGGAAACAGCTGGCAATGCTCGAACGCGCTAAAACTGGTACAACT

CGGCCAAAGGAAAAATGTACATCATTGAGGCTGCTCAGCTAAGTTCTCCTARAGGTTTGCGAAAACCAATGCAGCAAGTA

CTGCACGGTCCGCTCTCTGTGAGATACTTACACGCATATGAAATACATGTACTAGTAGCATGAAACACTACACATGTGCT

GCAGAAGCTCACTGCACTGAATTAACACTTTGCAATGAGAGAGTACCTCTGCATTTTTTCTTAGAGGCCCCAGAGGTCTC

GCACCTTTCGACAGGACAGGAATTATGAAAAAGTGCTGTTGCCAATTCAAAGTCGAAAGGTCATCAATTTCTGTGTCTCT

GCA

>CK188189_bb No definition line found

AACACCGGGTTGTGTGGGGCAGATGATGAGGGAACCAATAGAACCAAGCTACAGAAAATTTTCCCCGATTTATTCATATG

CTGCATTTCTGTGTGAAATAGTTAAAAAAAGCTGTGAAAAACAAGAGACCAGACAAAGTACACATACAGCGCTCTGTATG

TGTACTTCTTTGTCTGGTCTCTCGTTTTTCGCGCTGTTTCTTTTCCACTATGTCTTACCAACATGCCCAAGCAACCACTT

TAGCCACTTTTTCTGTRTGAAGTTGTATTAAACTGCATCGCGATGATGGTTTTGCATTTCGTCGCACGTACAGCTTGCAA

GCGTAAGTAGGCAGTGTGTCTAGCTTGCCCAACGTTGCCTACATAGTGCGAGATGTGATAGTATTTCGCAAACAATACAA

GCTGCTTCTTCTCTAGAACTGTTTGATCTTGGGTTCCACAAAATGGAATTGAGGCAAGCTTGGTGTGGTTGCATTTTTTA

TTCAGCAGCTTCGGAGCAATGCCATTTGTTTAGTATCACTATGGGCACAGAAGTACAGTGCTGGTCTTGGCAATGTTTGT

TTGTCTAATGTTTAAGCCTGCAATGCTTGCAACTTTTCATGCTTGTACTTTGGCTGTGTTAACCTTATGCAGTGCATTTC

TGTTAAGTGGCCCCTAGGTCTTTGCAAAAGATAGATTCTGCTAAGCATTGACACTACTATTTGCAGTGAGCCAAAGAAAA

AAATGAACGTGCAGATAGTCTTTGTAAAGCACAGTGCAAACAAACAAACAGGCCTCGTTTGAAATCTTTATTGTGTCTGA

ATGCTGATTTCCCGACATCAGTGCAAGTTTCCCCAACTGTCCTGGTGCTGCACTGGCAATTGAATGGCATTCTTAAATAA

CTCTTWATTGTGTCATCTCTCTTAACGCTCCTAGCTGCTCTGTCACTACAGAATGGCTTTTGCTCGAATTGATTACTGTG

CAGGTGGCCTCATTTGAGGTGCTTGCTGGGCACTTTCTGAATGGTGCATAGGAGTGCCAGTTTCTGCACACAACTTTGGT

AAATAGTTTCCTCTTTTTGTGCAATCAAAGTG

>CK188213_bb No definition line found

GATGTCAAATCACTACTCGGGCACCAGAACTGCTGAACCACCACAGAAGGCACCAGCATAGGCATGTACTCTATGCTTAC

ATAAGCAGCAACCATATTGCATGCAACAGGCATGTAGTTACAAGTACAAGAATTGTATGTGCATTCTTCTGGTGGTAGCT

TTACAAAGCATGGCCTTACTACATTGCTCTGCTGTGCAGCAATACATGTTCCTTATCCATGGCATCGAAGAAGCACCGTG

CTTTTGTGGCTAACAAAGCAGCAGGCTTTTATGTTTTCTTTGTTAATGGTTACTAATAGCCTGTTTCTGGTTTTGCTAAT

TAATTTTTTTATGGCAAGGGGACGTGTATAGGTTGTTGTATTTCTATGTTTTTATAAGGGCTGTTTCACGGCTTTATTTT

TTAAATTTCTCCAGTTATTTCCCAAGTCCAAAAATGTGCACAGTTTAAAAAAGTGCCAGACGAGCAGCTTTATTTAAGCA

CTTGTGACAATACTGCCGGTGACTGTTATTATAGGTTGTTGGTATATTTATTTCTATTTTCGAACCTCACAATTGGCTGG

GCCGTTTTCTTACTAAGTGGGAACTGGCCTATTGCAAGGAAGGTTTATTGTTGATTGTTTTACTTGTTACTTTTTCAATT

TATATTCTTTTGTTTTTAATTTTTCCCGTCTGGAAGTTCACTGGGAAAGTATATTTTTTGTTGTTGCTTTACATTAACTA

GAAAGGTGTCTAGTTGGTAAGAAAGAAGTAAAAGTTATTTTTTATTTACATTGTCTTTAGTTAAATGTTACTTATGTAGC

ATATGATTGTGCATYTGTACATAGTTTGCGTTCTTTTAATTTGGCGTGTGTAATTATTTGTATCAAGGCAGAAAAGGAAA

AAGCAGTTAGAGGTGGTCGGATGCGCAGGTGCTTAAATAAGACTGCTTGTTGAGGCCAAAGCGTGTGCCTTCGTGTTTTT

TCCCTTGCTTCATTGCTT

>CK188220_bb No definition line found

TTAAAATTAGTTTTAATTAACGTAATATCAACAAAGAAACAAGCTGTTACACAGTTTCATTCTTGAATCKTTTATTTGAT

GCCAWTTTTSTGGCTTTTMAWATGCASATACATCGGTAGAAATGAAAAMAAATCTRGTTCTTGCCGATTTSAAAAGTTGA

GATTGATTTCATTCGACTGTGAAAACCAACTCTAATAAAAATTGCATGCGGCAGCTGCTTRCACCAGCGCCATCTCTCAG

GGGAGTGCATGAAAGTGCCTGTGGTTGTCAGGCTTTCGTCATTTAGTACACGATTGGGCGGGTGTAAGCGAAGGCAAGCT

GCTTGTCCCACAGGAAGATGCCGGTCTTGAAAATTGTGTACACACGGCGCAGGAAGTTCTGGCCCAGCAGCCAGGTGGAG

GCACCAGGCTGTTGGTGGTCCTCGTCGACGACAAAACCACTGTAGCAGGTGGAGTTCACCTCGACAATGTAATCCTGGGG

GCGAAGGTCAAAGTTCTTGCCGGAAATCTTGATACTGATGATTGGCAGGGCCGTGATGTTCGAGCAGTCAACGGTGTACT

CGTCGCCAGAGGTCTTGGAGGCGTTGAGGGCCTTGTTGATGGAGTCAATCTGGGACTGGGGACCGTAAATGTAGGGAAGT

GTGGAGACAGGCTTGGCGTAAATGTATTCGGTCTGCAGCCAGCGCATACCAACCTTGACACCCTGCAGGCGGATCAGCCA

TTCGTCGGTTGCGCTCTGAGTGTAGGTCAGCTCTCCTTCGTAGTGCGACTTCTCAATGCCGCCCAGAATGAGCTCACC

>CK188244_bb No definition line found

AAAACTAAAAGTGGAACGGTTGGCAGCCCACCAACATTGCACAACTGATCACACACACACGTACACTGKAKGAAKGTTCA

GGCYCCRAAACAAACAAAAATGCATTGCTCAGGGTTGTTGKGGTCCGGCACAWAAACMCSCCCGCTAAAACAKGGCAAAA

GKGACAGCTAAAATCATCAAARAAATGCTCTACCTAAAAACACMCCCTTGAACTCAACTTATCCCTTTTGCATACTCTAC

CAGGGAAAACAACAACACGTTAAAACAGTCCCGTTTTT

>CK188336_bb No definition line found

GAGCATCGTCCAAGCCCGAAAGCTGGCCGCCAAACACACAGAAAGAACTCATCTCTTCCTCGGCTTAMGTATATTAGAAA

CYCATCRCCGGCCACTTCTGTACGGATTTCCGCTCCTTCGCTTCGGAGGACGTTCAAAAAAACCRAGTTCGGGATCAAGC

TTGAACAAGAAAAGATGCAMTTTCTSGCAGTACCAGTTCTTCTAGGCCTTCTGAGCAGCACCTTTGCCGCCTACAATCAC

GGCGCCGGTGTCTCACTTCATGGTGCCCCAGCGGTAGCAGCCGTGCGGCCAGCCACTTACACAGTCACTACGGCTGCTCC

GGCTGTCACTAGACTGGGATACGGCGCCGGACTGGGATCGGGAGTCGGCCTCGGCTACGGCGTCAGCCAGAGGGTCGTCA

GCGGCGTTCCCGCCACCGCTACGTATGTCCAGCAGGCTCCGACCGTGTCTTACCGGGGATACGGTCTCGGATACGGCAGC

GGTCTCGGATACGGCAGTGGTCTCGGCTACGGCAGTGGTCCCGGCTACGGCGGGCTGCGCTATGGAAGTGGCGGCGTCAC

CACGATATCCGCTCCGGCTGTCTCTCAAGTGCGCCGCTTGTCCTACTCGGCGCCCCTGGTTTCCACCGCACCGGCGGCCG

TCAGCTATTCCGCTCCCGCCTCCGTGACGACCATCCACAGCGCCGCTCCCACCGTAGCCGTCAGGCCTGCTGTCAGCTAT

GCCACGGCGCCTGCCGTGACCGCTTACCGCACGGCTGCGGTTGCTGCACCCGTCGCCACTTTCGGGGCTGGTCTGGGCTT

CGGACACACGGTGACCGCGACACCTGCGGGCTATTCGACTCTCGGCCGGTACAGCTACGCAGCTCCAGCCGTTGCTGCCG

TCAGGCCTACCGTGAGCTTCGCTACGGGTCCCACTTTGACAGCCACGCGTCCAGCTTCA

>CK188347_bb No definition line found

ATGTATACTTTGGTATTTCTGACGCAGGGTGTGTCATGCCTAGGTCATTAAAATTTAGGTTAAGCTACAATTCTGAACTG

TGAGCACTTCGGAACTTTGCAGACACTAGAGGCCATTTCTAGATGGGCACAAATAAAGGAGCAAAAGACATCAGGCTGTT

TCTGAAAAATTTATTGTCATTCAATATGTCACACTTTGTATTTACATCTTCCTGTARACAGTGCACTGTAAAGAGAGCTC

GGCAGGGAAAGGACAAAAAATARAAACTCCCTTTTTTTTTTTCTTGCCTCTGTCTCTACGTACAGGTCTGTTCTTGCAGC

AGCAAAACATCAAAAAACCTAATTTACACCTTCCACAAGTCTAAAGGGGGCAGGGAGGGCCAATCACAACACACTTGACC

TATACTCAAGGAACAAAAGCCAAGCTCATTGTGCAGGAGGCAGGAACAAATTTTTGCATGTAGCAACAATGCACCAGGGA

AGGGAAGTGTCTGTCTACCCGTGCTCCAATATAACAATGAATGGCTCATTTCCAACTCCAACCATGTAGCCCTACGACCC

ACTTATAGGCTGCAAATGCAGCCACCAACATGTCATGTGATTGAATATGAAGCAAGCACATAATAAACTGGCACAAGGTA

AGTCCTTGGTTCTTGCTTTTTCTTTGGAAGTTGAAACTGCCCCCTTTTCACTCCTGCCTCCACTATTTCTTTTTTACACT

AAATAATTTTCTTTATTCTGGCCCCAGTTACTC

>CK188467_bb No definition line found

ACCAGATAGAAGAGGCCGTTGTACGAGTCGTGCTTGTCCTTGAAGTAGCCTGCGAAGCGTCGAATCGGTTATCCCTGCTT

TTGGTATACGTCGGACACGCACAACTCCTTCCATAAGACGTGCGCCCGCATTAGTTAGTTGTACGAGACCGAACAACGTG

CCAGGAGCCATTCATTCTAGATGCATGAGTWGKWCTMACTATWTRGTGMYRCWSWGYRKCACYAWATAGTTGTCCAATCA

TTCGTCGACGGTTAAAAGGTCGTGTTCCGAGCCCGCGAGAAGATTCCTTCACTGCAAGAAAAAGTGCCGACGATTCCGTA

GTTTTGGGTTCGTATAGGTTTTCCATTATTGTAAAAGTTAATTCCTAAGTTTGTTTTGCTTATTTGTATTCCTTAAATGA

TGCCTCTAAAAGTAATCCTTATGGGTCAACGGTCACTCTTTTTTTTTCTTTTCGATAGCATACGATGGCCAGACAAGCTG

TCGTATTTGAGTAGAGTATCGCTCTTGTATATATTCCACGTTGCGGGTATAGTGACGTATTCGGCGCTCCACGCTGCCTT

TGTTGTACCGTACGTTTCGCACGGTAACGTTTATGTTTAGAGCCTTATAAGTGGGTGTTAGTTGCTATACTGTACCGATT

TAAATGTGCGCCGTGCCATGCCTCCTGCAGAAACTCAGAAATAGAGCCTCCGTCCTAAGTAGGTAGTTCCAAACCTTGTT

CTATCCTCTGTACGCAACCACTCATCCCGTGCCTCACCACGTCTTTAGTCATCTCTGTAATATCACTTTGCACATAATTT

AGGTTTTATTTTACTATTCGCGCGCTCTGGAGGCATGATGGACGATTGGAGAAAAACTTCGCGTGAGCTGGTTGTTGTGT

AAACAGATTCACAATCAATACGTGTCAGTTAACGGTTTTCTTTATTTTTATGTGTACGTGTGTATGTTA

>CK188504_bb No definition line found

TACAGAAGCAAACAATATATACACAAAGTTTCAGCCAGTTTTAGATAGTCGGGGTATGAGAGAAATTTCTGAAACAGGTA

CAGTAAATTCACTTATAGGGAACAGCATGTCATCTACAAGGGTCCGTGATACTCCTCTTAATTTGCACCTTGGGGTACAG

CCTCTTCCACTCTTGCACAACATCGAGGCTGATACTGAAGCAGAAGGACACGTCGAGTATCTTCAGTTGCTTGCATTCGT

TCAGCACCTGAATGACTCCAGATGAGCTCGCCTCTGCAGATCCTAGGATGTCCAGATGTTCAAGGGCGTGACAATATTTC

GCAATAGCATACAGGTCCGTGTCGCAAAGGACGCGAACAGCAGGTAGTAGCAACCTCTTCAAATGGGGACAACCTCTTGC

GATTTCCTGGATGGATCCCGACTCAATGCTTGTGCACCAGCCTAAATCGAGGCTGACTAGATGTGGACAGGACCGGGCAA

ACAAATTGGCACCAACAGATGACAGTGTTCTAGCACGGTAAAGGTCTAGGCTTCTCAGGTTTCCCAGGTAGGTGCTGATC

TCCAGAGCAATGTCGTCATAGTTGTTGACAACGGGACAGCTGCCTAGGGACAGATGCCTGAGCTTGTTGCAATTCCTGAY

AATKSARATACATTCTTATCCATTGCGCCAGGTCCCCACTCAGGCTCCGCACAATCATCACAAGTTTTACTCCTACTAAT

GATTGTTTCAAAAATTCAAGACAACTAACAACAACATAGTAAACATCAATTTGCAGCCCATTCTTTTAGTAAAATTGATA

CATTAAGCAAGGT

>CK188613_bb No definition line found

ATTAAGGTTGATCGGTTTTTCTTACATCTAAAATCATGACACATATAATCTATTTGGGCAAGGAAGGGCTCCGAAAAACT

TGCGTGCTCCGATTTCGATGCGATATCGTCTAACGCGTATATACATGTATTTAGCGCAATTTTGTAACTAAGCGACATAG

AAACAACTCAGCAAGTTTACACAAGAACAGATGACACAGTTTATTTACAAGTTATTTACAAAGCACTGCGCGCATTTGTT

GTATGAACAAAATAAATAGGTTCGCATTTCACTCAAATGGCAGAATGCAATATGCTGATAATGTGAAAGTCTTGCGCGCT

GTGCGAAGGTTTGTTAGTAAAAATAAATATTTAAGATGTTTTCATTCACGTACAAGTGCTTGCCCCTTTTTAGCGCATCA

GCATTCTGTGGGTGAAAGTACGGCTATAGGCCGACCAGCCACTTCGCCTTTGATCCACATAGAGCCTTAGTAAATGCGGT

AGCTTCACTAAAGCATTGTCATGTTTGTTGTTTTTGGCTTTTGATTCTTAAGCAGCAAGCACAGCATAATAGTGGCGCGA

GATCCGTTTGATTAGTAGTGTGAACGTGAGTAACRTCMAAGGGTCGAAGTKATTGTTTCTTGCCAAACAAAGYGACGCTA

CTGTCATTACAASCGGTTCCTTCCTAGATGCGACCACATATYAGTATGACGTTGCGAACGGAATTAACAAAAAWTGAGAG

AGAGTGGAAGATTGGAAGGCAGTAGGATGTGCCCATATAGCTCATTACATGCCACTAGCCCGTAGGCACAAGAGGAATTC

ATTGTTCAACCAACATGTGACGCAAACAATGATATCATCCCGCGTGAAAACTTTCCCGAGCGAAGCTTCCGCGTCGGGCA

ATTACGTGACACGTAATGAGTCGACTCGGTTCGGTTGCAGCCGTCACACATGGTCGCAGACGTCAGCGAAAACAATCAGC

>CK188633_bb No definition line found

CATTTGTAGAGAGGATTTGCATTTCGGGGTATGTCATGTACGCTGCCCCGTGTAACGTGGCACCTGCTGTCATTTGAAAA

ACACCTTGTAGGGTCAGTGCCTTGATTCGTTTGCATTGTAAGACTTTCAAGTGCGTCAATGCCTTCATGCGCTGGGTCTT

TGCGTTTCGAGTCTACAACGACATGGGTTCGGTTGCTCACGATTGTCCACGGTGTGCATTAAGGCTATTAACTATCATGC

TTGTCTTCAGCTTATGCTGACTATACTGAAATGCAAGGTGGGCATCAGCATCTTGCACGAATGCTTCACGAGAGCTGGTM

CTACTGCCGTGTTATTTATTTATTCATTGTTGCTTACAAATTGTTACTCCTGATGCTGTGTTCTTAGTAGCAGTTTGGGC

ATTGTGCTCTAGGCACAATGCGTCAATGTGGTGAAATTGAACAGGGCTGTTCATTTCTCATGCACTAGCCACATCATGCA

AGGCCGTAACTGTACTTATTTATGGAAAACTTCCTGGAGTGCCCAACTCTTTTACAATGTTACGGCACTTTGATTTTTAA

CTGTCAAAAAAAAAAAATGATTGTCTATTCATAGTATTAGAGGAGAACTCCGTGCTTACGCTTCTGATTTTTCATATTTC

GTAATGAAAGATTAGATATCAAAATTAATGTGATCCCTCTTACGTGAGATTTTGTTGTAACATGAATTTAAGGCATTTGT

TCTGATAATAAGGCATAGGGCCACGACCTCCTTCGCTCATACATTGAAAATATGCACAAATGAATGTATCTATTTTACTA

ATC

>CK188667_bb No definition line found

TTATTTAGAGCTAATGATGCCCCTTTATTGTTTGTATGAAAGAGTAAGAGCGTGTTTGTTGTTTCGCGTTTTGATCAATC

TGTCTCCCATTGATTGTTGTTTTTTACTCACGCTATCTTATACGCTTTTTCATTTGTTTCCAAAATGGCGTTTACATTTC

TACTCCAGTTTACAGCGTATGATAAATCCGTATCCACTTTACTTCAAAAGAGGGGAGCCACCTGTTGTTGATCACGCTCT

TGCAGTCTAGTCTCTCGTGGCTGATCCACGCAAACGCTTTTTATGCAAACCTTGAGTGTCCCACATGACATGTGGTCAAG

TGCATCGCCGGACCTGGAGATGGTCTCAGTATTGTACGACACATTCGGTACTGTCACGATAAGCGGCTAATTTTTCTGTA

TGAGCACTTCACTTTGCACGTTGTTTGGATGGTCTCAGCCATTGTAAACGTATAATTCGTTTTATCTTCAAGTTCCAGTA

GGCTCATGCAGAATTTCGTCATGTCTACCACCGTTCCGGGGTAAACGTCTACGACGATATTTTGTATATTCTTGTGTTCC

CAGCATTTTCGTCCCCTAAGCCAGACCACGTGACGCATTTCTTGCACACTGCACCACGAAAACTCCTGGTGGTTGGATCC

CTTGTTGATGTAGCTCATGAGGTACCCGTCTTCCCAGGGACAGGCTTTCGATCCCGGATGTCCGGTTACACCCAAAAGAG

GGTCACTTCCATCGTGTGTTGCACCCAAGAGGTGGGCCGTCTCGTGAGTGAAAGTGTGCACACCAGTGAATAATCCTGCT

TCGTCTTCGCC

>CK188781_bb No definition line found

CACCTGTGCTCTTGCTGAATTAATTCGCCGAAAGCAGTGTAGTGCGTTGCCATAGTCCTCTCTTCTTTTCTAAATAGGTG

TGATCCCCTCCCCAAAAAACGCACTGCCGAGGCTGTTGTGACGTCAACCACTGTACTTTCCCGCGCGAACAGCGTGGTGG

AGTTTTTTTTTGTTCTTGTGAAAAAGTCTATTGGCAGATTTTTACATGCATTACTTTCAGTTACAACATAAATCAAACTC

TGTCAGATCATTCAGGCATTATGATCACATTTAAAGGGCGTCTTCTTCACTTCTTTCGTAGTGATGGTGTTGTTCTCATA

CGCTTCCCAATATTGTTTGGAGTTATTGCATATTCTTGACCAGAGATGTAGAATCGCGCTTAATACAAGTTGCAACACGC

TATCTGTTCTCAATAAGGCGCCAACTCTGCAAAGTGTCACTTACACAAGAAAAAACATTTCGGAATTAAACCGTCTTTAA

TTAGCTGTACATTCTGAACCAATGTAATGTTTGTTGGTGTTATTGTTCAGGCTTGGGGTCCCTCTAGTCACAGCCAGCGT

GTTGTAATTGATATTGAGCGCGACTGTGCATTGTCATTAGCCTTATCTTCTACGTTTATTTATGTTGATTGTACCTCGGT

TTTTATACTTGGGAGTTTTTGTATGCGTTTGTTTTATACGTGCTATATATGCGCTAGTTTTCGCAAGTACAAAGATATCC

CTGTCAGGCAGTTGTCTTTAGTGTCTCCTACCTTTGTTACGACAAATAAGCCTCGATTTCATTCGGTTATGATGGCCCTG

AGCCAGTCAATTCAAAGAGCGGCATCAATACGCGGCAATAGTGAAAAAGTATCAAATAGCAGCTAGGTTGCGCAGCGTCA

CCAGAAATGTTTTCCAGGGTGCGTGGTGTTCAATCATACGTTGTGCATGTCAGCGCATGCATAAAGACACATGCCAAATC

GTAATTTTGGGAGGAAGGTTTCGAACCCTTCACTCGCATGCATACATGGCTGATAGTAAAGCAAAAAAAATTACATAAAG

GTGATAGGATAGACGACTTAATAC

>CK188830_bb No definition line found

GCAACAACAAGCATAGGGGTAGAGTTTATTCACGACATGCGTCAAGACTAAAAACACGTGCAGCATCTTCCCACTGTTGG

TAACAAGGGCAACAGTATAATACAACTCCCTCATGCAGCGCATGACAATATCGCAACTTACAAAGCGTTCATGCCCATGC

CATTACCAGATGAAAGCGACGCACCGGCGAATGAGTGGAAGGATGAGGCGCACTCCGTTGCAACGAACTCAGCCTTCCTC

GTCCGCGATTTGCAGGGCAAAGTGTTAAAGCGTGAGCGTGCTAGCGGGGCGCATCGGTGCATGGAGTGATGGACACTGTA

GTCAGTACCGTAGTTACCAACCTGGAACGTGATGTGCAGTGTCCGAAATAGGTGTTTTAAATTAAAAGCATGATGGACGT

TGTTGTATGAAGCAAGTGAAGTCAGTCTAAAATATGAGTATTATTTTACATTGGTGTCCAGTTACTGCGTTTGGTAAATA

AAAACTACATACGGGGGGTACAAATTATTGTCTCCACCACACAGCAGTATAAACAGCACTGTTCTGGAATACGCGCAGTC

TACTCGTGCCTGTGAACATACTTGGGCGTTGCTCCATACCGCGCAAACTACGATTTCGACARCATTATTTGTTGGGGAAA

AACACAGACCACTCAGAGCGTAAACACTTATCCTGTGCTCGAATTTCATAGATAGTGACTTCGYATCGCACCGTAAAAAG

CTTCCTCCTTGTTTGTTACGCTCAAGTGAATCTGGTGATCATAGGCAAGCTGGCTTTCTCGTTTCTTACTGAATAAAAGG

AAAGTAGGATCAGGCCACCACAGTGCGAGACACAGCTCGAGCCTTGTTCATGTGGC

>CK188831_bb No definition line found

CACATAAAAAAACTATAGTTTATTTGCAGGAGATTCATAACTTCGCGTTTTGATGCACACAGTCTATATGTAACACTCAG

AGAAACAGTTCATAAAAAAAAGCAGACCTACTGTTTCATCGATATCTTACAGTATGCACAATGAACAGTGAAGATTACAC

GATGTAGGCATCTTCACCCGCGGTACCACCAGGTATTCACCTTTGAAGTCTGAATGTTTCAAGAGGAATTGCGCAGCTTT

TTCTGCTGCCATCATCTCGCTGTAAATAGGCACTTGTGAGTTGTATCACATGAGACAAAAAAGACAGTTGAAGCACATAG

CAAATAAGTGAAAGAGAGAAACTTTGTTAAACAAGCGCGCAATTCGTATACTCACAGAAACGCTCGTTAAATAGTGTGAG

CGATGGCTTTTCCACGCCATTTCACAGCCTCTGCGTACTTGAAACACTGACAGGGTGAAAATGTAATGTTTCAGTTCACA

CTACAAAAGGAAGATGGAAATTAGTGGATGACTAGTAATGGATAACTAAATGGGCACGATTCTAGCTCTTTCCTGTAATA

CCATGGTCAGGACGATGACGCTATTTACAATGTGATTTGCTTTTTGGTACAGTTTCGTTTGATTGCGATTTGACGGAAAC

AAAGACCGTCTTTACTTTCAGACTGAGATTAGACCATTGTGGGAAGTCTAGCGACCATTGGGAACTTCAAGAAAATTAAT

ATTAGGTAGAAGATACATTCGCGAATATGGTGTGGTGGCAGTGTTGCCAAAAAGAGAGCGTGGCTGCAACAGAAGGTGAT

GCACGCCTGAGAAGAAATTGGAAGC

>CK188915_bb No definition line found

AATAATTTGTGTATACGGTACAATTTTCACTGAAGAATAAAAAAAAATGTAAAATGCACACCGATGGAGAAAAAGGCACA

AACATCCACATACCAAAAGCGCTTGTGTAGGAACATTTTTTTTTTTTTATAATACCAGCTACAATGTTACAGAAAGCCAA

CATACAAAARCTGCCAGCCTTGTGAATTTTTATTGAAGCTAGCAATGCAACTCATCTGTRTCAAATATATTATAAAAAGA

ATAGGCAYGTCTTCAAATTGCCTAARAGTATTCTTATGCTAACTGTAACCCGACTCAATGTGCAGCCRTCARATAACACC

CGAAATCACGTGACATGTTATGTCACGCTTCTCTTTATATAGCACTAACCCAACTGACAATAAAGTTGTTGTTCACACGA

CGGAAGGCCTTGCTACACTAACTAATGTGGCTCTTCATCAAGGATGCTGCAAAATTGCATGAGGCACAGACAGCTACCTC

ACATGAGAATTCAACAAGTAACTTGTACAACACTTTCATCTGCTGAATCAGTGACTAGTTGCTCTACTTCTGTGCACAAG

ACGAGTATGGTGTTTATTTTAATAATAATAACGTTGTTGAACAGTAATGAATATGATAGTTGCAGAGC

>CK188978_bb No definition line found

GAGTTTCGTTCCACGGTTATATATCAAAACGTGGTGACCACAGGGATGCCTAACAAAAAAAAAATGCCTAAAACCAGCCA

TGCCTGTGTAGCTTATGAACAATCCTCATATGAAGTATTCATATGACCATATTCGGGCCACGTGCTGCTAAATAGTTGTC

TGCGGGCCACGTTTGAGGCCTCTGCAATAAACTATGGCCATCCCTCTCCCACCCAATGCACACACCTATTAGCTCAGTCA

CATGAACTTATACTAAGAACTTTCAGTTAGTTTGTCATTGTGCCAACGGACATGCTTCCATCTATTACAGATGGTCAAGG

GTGGCTCGTAGCACAGCATAAAATGAAAATGTCAAATATAAGCAGCACTGAGAGCAACTTAGCTTTGTCATGTCCGTTAT

ATAAAACAAAGCCYGTCGWAAGWTATCTTTAGTTTAACCAGATGGCAGAACCAGYGTATGGATTCTTGGCATTCACATGT

TATTTCSGGCATGGTCATGGCGCACATCAGTGGCCACACAAGTACGCTCAGGCACAGCACCTAAATATTCTAAGAGTAGG

GCAAGTATTTCTTTATGCTAAAAGGAGAAATAATAATCAAACAA

>CK189048_bb No definition line found

GACTTATAAGAATGAGGCACCATCTGGTAAAACTGTGTCTCTTCCAACTTGGTTGATGTAACTATGAAGTTCATAATTAY

TACTCATAAAATCAGTCATATTTTAYTGCTTTCAACGTGCCGCTGTTACAGTTGCATGGCCTCCGAGGCAACAGCTCTGA

TTAAAACCCTGATCCTCAAGWAAGAAWTSAAATTTCGTCRGCTGCTTAATAGTTAAAGCAATGCACAGTCAGAAAATGCT

GAATATGCTTCCAGCCTGGCTAGAAGCAAKAGCATCCTCCGATCATCTATCTGTGTTTGGTGTGCTGCTAATCTGCAAGG

GCGACACCTGTTGGAGGGTCATCCTCATGTAAATATTACATGCGCAACAAGCACTTGCTAACCTTTCCAATTGTGTAAAT

AAGCGCTTCTCACACACCAAAGCCAGTGTTGTGCTTCCTCAACTCGCAAGGAAAAGCCTGCACCAAGCGTGTTGTAATCG

TTACTGTTTCATTAACACTCGAAGTGCCCCAACGCACTCCCTCAAGCCACCCTACCCTCATCTATCTATCCAGTCAAACA

CTTAACGAACACCTGTCAATCGAATCATGCTTTTCAACAAAGATCCTTAACTCGTAGCCTCGTGCACTGCAGTTGGGACA

CCAYATAGGATACTACATCCATCATTTTTCATCAGTCACTGCGGACTTCAAGAGGATCAGTGACAAATCCACACTATGTG

TAAGCCATAAAGGAAGGTCCATAATGTAGTCCACCTGATACACGTTGCCGTGRCCCTGGYTATTCTAAATGCAGAGCRYC

TAGATTATTTTTATAGAAACACAATTAYTGGAGAAAAAGATACTGTTGCTGTTAGTAAATATAACATGTTAGAGTCTTGA

TACACTTCTAAAAAATGTTTTGATCATGCAGTGTCACAACTTGAAKGCTGGACAAAGATGCTTGRTCTTACTTCACTTCG

ATTGAAAAATAGT

>CK189050_bb No definition line found

CTGGGGAAAGGGAGGGGGCACGCCAACTCCCAAGGCAGCCACGCCTACCGGAGCTGCACCATCTTCCTCTCATAAAGGTG

CCAAGGCTGCTGCCCTTGGACCTCCGTACCCGTATCCTCTGCACGGTGGAGGACCTGGCGCTCATGGTATGCCAGCTGAT

CTCAGTGCGGCAGCCTATCCACAAGGTGTTCTGCATAACAGTATTGCACCAGGGCTCAACTCGTACTCCAGAGGACTGGT

TGGCTATGAGCCGCATCCATCGATGCGAGGATTCGGCTCCATATCAGCAGGAAAACCGGCATATTCCTTTCACGTGAGTG

CCGAAGGTCAGATGCAGCCAGTGCCATTCCCCCCAGATGCATTGGTAGGGGCAGGCATTCCGCGGCACGCACGGCAGATC

AACACACTGGCCCACGGAGAAGTGGTGTGTGCTGTCACCATCAGCAATCCAACCAAATATGTCTACACAGGAGGCAAGGG

CTGCGTCAAGGTCTGGGACATTAGCCAGCCAGGCTCCAAAAGCCCAGTGTCTCAGCTGGATTGCCTGCAAAGAGACAACT

ACATCCGATCTTGCAAGCTTCTTCCCGATGGACGGACGCTCATAGTTGGAGGCGAAGCCAGCACTATTTCCATTTGGGAT

TTGGCAGCGCCGACCCCACGCATCAAGGCAGAGCTGACCTCAAATGCTCCAGCTTGCTATGCCTTGGCCATCAGCCCTGA

CTCGAAGGTTTGCTTCAGCTGCTGCTCAGATGGAAACATTGCTGTCTGGGACCTGCACAATCAAACCCTTGTGAGGCAGT

TCCAGGGCCACACAGATGGGGCCAGCTGCATCGACATCTCGAGTGATGGCACAAAGCTCTGGACGGGTGGTCTTGACAAC

ACCGTGCGCTCTTGGGACCTTCGAGAAGKACGGCAKYWGMWKCMKMAGATCTTCTACACATGTAATAAAAAACTGTTCAC

CACTCGTAAAGGATGACTCTGCA

>CK189070_bb No definition line found

GTCACAGCGTTGCTACGCAATTTCGAAGCACAAAGCGTGAGTGTTTGTGCAAGAAAAATGTTGGCCGTAATTCTGCAGCA

ATGCTTCAACAACGTGAGAACTGTACGGTGTTCCCCGCGAAACCAGGAGGGTGTTTCCCACATTTCCTGTGCTAAAGATG

TGTGATAGTCAGTGCCAAACGTTGCATTTTCAAAGCTCACAAGTGTTTCGTTGTTTGTTGTTTTACTTTGGTTTGAGACT

TCACGTATGTCAGTTGTACGCACGCCCATCCTACGCATAGAAGTGAGTCACGACAAGTGACTGGAGTAAACCGCTCGTCC

CATACTTAGCTCCACTTTTGCTGTTTCGATGCTGCTTCAAAAAAGGACAGTAGCTGCCACTGTAGCTAATCGCGTTAGAC

GTATTACCGAAAGAGCTAAGAGCTCTCGCTGAACTACAGTTATACGAAGAACGAAATAACATTAACGCAAAAAAGATCAC

CACTATTTACTTTCTTCTGAAAGTATCGCTGTCATAAGTTCACGTTCAGTCATCCGATCGGCTCCAATCTCAAGCTTAGT

AGGTGGCTAACGCCAGTGTGGGGTACTTCTCGCCAACTCATCAAAGCAACGTTCTGTTCTGTTCTGTTCTAACTGATTTG

CTGTGGCGAGGTTGAGGTTCATATTGCCTYGGCTATTTYATATCGATGAGTTCTGGAGYGAAAATAAWACTTAAAAAAAT

AATACTGAATGATATCCAAAAAATAAACRATCASAMAAAAAAACATGATAGCACACTGAATCAACGCATTTCATTTTGCT

GGAGTACGGCTGTTAGTGTAAAAAAATACCATTGATGACCATTATACCGTGTGATGAAATTTCGTACCTTTCACGACGAC

AAAGAGACAAGAAATACTCGACACTCA

>CK189219_bb No definition line found

CGCTGTCGAACGTGGATATCAACGTAACACCGAGAAAGTAGAACCGCAAGTGGTTTCCCCAACATCTGTGAGATACAACA

GCTTTAAAACGTCACCGCTTCACATCAGACTATCATGCGTGAGCAAACCTTCTGTTCCTGGTTCCATTTGAGTCACTGGC

ATGAGGATATTTCCACTGGGCACGTCATTCCACGAGAAGCTTGAGTTTGGTGGCTGTAGCAACAGCGAAGTGGGTTGCGT

CCGTAGACCACTTTCCGAACGAAGAGGCCGACGCCAATGTGGTGGTGCAGTCTTGCGAAGCGTGGACCAACCAACACGAA

CTGGTGCTCCAGGGAGTCGCCAAGGTGCTCAAGGATGACAAGATCGATGCTCTCATATGTGTAGCCGGCGGATGGGCCGG

GGGAAACGCAGCTTCCGCCGACTATGTGAAGAACTGTGATTCTCTTTGGAAGTCCAGTGTTTGGACATCAGTGATTGCCT

CTAGCCTTGCATCAAAGCACTTGAAAGATGGTGGTCTGCTTGCACTGACTGGAGCCAAGGCTGCTCTCGAGCCCACACCT

GGCATGATTGGTTATGGCATGGCAAAGGCGGCCGTGCACCACCTTGTGCAGAGCTTGGCTGCTGACAAGAGCGGGCTGCC

ACAAAATTCAACTGTGGTGGCTATACTTCCTGTGACCCTGGACACTCCGATGAATCGGAAGTTCATGCCCAAGGCAGACT

TCTCCTCTTGGACTCCCCTAAACTTTGTGGCTGAGTTGTTCTTCAAGTGGACCACCGGTCAAGAGAGACCAGTGAATGGT

AGCCTAATGAAGTTGGTGACTGAAGGGGGCAAAACCAATGTTTCTGCCTGATTCAGCCATTTGGCCAGGAGTGGGGCCTA

TCAGTGATGATATCTCAAACTCTCCCAGATCTTCTCCGTGCATTTCACATATTTTCCTTGATGTGTGCTTCTTATATGCA

AAAGAATGGTCATCTACTTATG

>CK189289_bb No definition line found

TTGTAGTATAGCAATAAAGAAAAAGTGGTCGCGTACGTGCTAACAAACAAAACGGTGCAAAGACTTCTGAAACCCCACAC

AGGAAGCACAAACTTTGGCTGCAGCGTGAGTAATCACGATCCCAACAAAAACACACATGATCACTGATTGCAGGAGTGAA

GCGATAAATAAAAACAAGGGTGACAAAACAAAAGCGTCAAGTCACCCCACAAAACATGTCAAACATTTCTTACAGATCAT

TCCAACAGCATGACTACATGGCAGCATGTACACTGTAGGATCCCCAACCAACTCTTCAGCAAAACATATATCGAGCAGAC

AAGGCATAGTAGATAGGCAAGAAAGAGTTACCTCTCCTAGCAAATGAAAAGGTAGAAACACACATCACAAGTAACATCAC

TCTGCAAGAGCCCAGAAGAGGGAAAAAGACCCGTGAGCACGCCAAAGGCAAAAATGCGAGGCACAGAAAAATAAATTAAA

ATGAAGAAATGGAAGGCGGCAAATGGCTCATCGTTGCTAAATGTGCAAAGGTGCAACGTCGTTACAAGAGAAAAACATTC

CGCAGACAAAAGCTTGCGATGATTCTTCGCTACACTTATAAAATACCGCTGCCAACCATTTCATTAATGCCTTTAGCCGT

CAGCCAAAGAGGAAACCCCTTTAAATAGATAGGATGAGTCATTAACAAGAACTTTTTACTCTATGTCCTAAATATATAGA

TTGTAGAACACCGACAATGCCGATATACTTTGAGAACTCTTTGTGACAAGATGAGAAAGTGAAAAAGGTCACTACGAGCA

CACGTTCTGTTCAGACATATACAAGGGTTTGGAAAAATATTTTTCTGCAAGACCCAGAGAATAATAACCTTTCTTTTCAA

TCACGTTCAAATAAGCGGAAAGTGGCCCGTCGCACGCACACCCGAAATGAAGCGTCCTGGTCCTCTCATGTTAAGCATCC

TACATAGTTTAT

>CK189309_bb No definition line found

GTAGTTGTTTCTTTGTCAAATACAACTTTCTATTTCAAACATATCGAAAATGTCAACCACGACATGCACACTAGTAGGCT

TGTTTTTCCYTTKAAAAGAGAAACAASTTGTGTGCAATATATTTCGTTTCAAGTCTARAAAAAAGAAAAGAAATTATTTT

ACTCCARACRGAACACTAGAAAGAGAARTAWTGCTTAATATGACACTCATGCARGRCAGCTGCTACTGCTCCAAAATACA

ATATTTGAACTAGAGACACCATGCGTAACGACCTGTTCTGCATGGCAACCTACTAAGCCTCATGATCACTTAAGTTTGGA

AGACAACTTTTTG

>CK189310_bb No definition line found

GACACTATTTATACATGATATATAATGCCTCAATAGAGCAAAAGCGTGTCCCAGACGACTGGCTTGTGGAGAAGGTCGTG

CCAACGCATAARGGTGGAGACCAACAAAAGGTGGAAAATTACCRTCCSATTTCCCTYACCTGTGTRTGCTGCAAGCTTTT

AGAGCATATTATATCCAAAGCTATGTATACGTATCTTGAGGGCACAAAAACAYTTTTTYCTAATCWGCATGGTTTYYGGC

MRAATTTATCCACTCTAATCCAATTAATAGAAACAATCGATGACTTCACACGTGTCTTAAATAATAGTGTTCAGATTGAC

CCAATATGTCTCGACTTCTCCAAAGCCTTTGACAAGGTTCCTCACAGGGAGTTGATAAGTTAGCACACCTAGGCATAAAT

TATAACATAATACAATGGATAAGTGCGTACCTG

>CK189317_bb No definition line found

CTGGAATAATTTCCAGTCATAAAAACGCTGGAATAATTTCGTCCACCTAGGCTTGTTTACCATGTCTTAGTGAAAACGCT

GGAATAATTTCGTCCACCTAGTGTTACGTATTCGGCAGCTGGACGACAGCCGGCCCAGGAAAGAGACGACTCAAGCAGTG

CCAGGAAGACAATCCCAGTTTATTCACCTTGCGGCGCKTTTTTAAGCGCTACSCAGCCAATCGCAGGTTGGCACGAAAGA

AAAGAAAACAGCAAGATAACAGCAATCGGGCAATGTTATCAAAAAGCCCCCACGTAAAAAGAGGGCGAGCACTGCAAACG

TTACAACTCGGCCAAAGCTACCCCTAGCGGAAGG

>CK189318_bb No definition line found

TGTTGTTGGCCAAGGGAAGCGACGTCCTTCCGAAATAAAAGTTGAAGCAATCACTAGCTTTCCTTTGCCGAAAACAAAGA

CTAACTTGCGTTCCTTTTTGGGTTTAGCCGACTACTACAGAAGCTACATTCCCAACTTCGCAAATATCGCTAGTCCTCTC

ACAGACGCACTGCGCAAAACTGAGCCAACTATTGTAAGTTGGAATAAGGCTCGTAAAGATGCTTTTGCTGAGATCAAAAA

GATTCTTGTCAGCAAACCGGTCCTCGCGGCACCAGATTACTCTCTCCCCTTCTTAGTTCAGTGTGACGCCAGTGACCGCG

GAATGGGCGTTGTTCTAAGTCAAGTGAACAAAAAGGGCGAGGAACATCCTCTTGTCTACGCCAGTCGAAAACTAACATCA

CGCGAGCAAGCCTACAGTACAACTGAAAAAGAATGTGCTTGCTTGGTCTGGGCTCTCGAAAAGCTGTCGTGTTACCTCAA

AGGCTCCTCATTTGTTTTTGAAACTGATCACTCACCTTTAGTGTGGTTAAATCAAATGTCGAACAAAAACAGCCGCTTGA

TGCGTTGGAGTCTAGCACTTCAGCAATTTGATTTCTCAGTGCGGCACAAGAAAGGCAAACACCATGCTAATGCGGACTGC

CTTAGCCGAACCTATTAAGTGTTTCTTGTTTCTTTTGTGTGCATGCAACTTGGCGCAAGTATATATATTTTTTTTCCTTA

TCGTGTTCGGCTTTCACGTTATCTGCTCCCAGTGAAATCAACTGTCGGGCTTTTCTAAGCTTGGGCGCCAGGTTGCTTTT

GACGTAGTTTCTGCGTTTCTACCGCTCGTCCACTCAGCYATTTCAGATCCTGTCGTACCAGATGGCGAAATCAACCCTGC

TCCAGGCGCCTGCAGCCAAYTCCCGTGCTGCAGCCWATCRACACTCGGCGAGTGG

>CK189372_bb No definition line found

CGGGAACAACCTGCTTAAATGTAGCGAGCCATCAAGGACACTGAGGACGAACGTGATATTCATCTGTGACCGGGACAAGC

ACATTCCCATTGGACCTGGGTCCCTGGTCACCAAGCTTCCGTACGACAGCATTGACGAGCGAGATGCGTGTGAGCGTAAT

GTGACCGTGAAGTACGACGGGGCATGTGGCTCCAGTTCACCGGTAACTTCATCAGGAGGACTCAGTACTGGATCCGTGCT

CCTCATACTGTTCTTCGTGGCGCTGCTGATATACTTCGTGGGAGGCGTCCTAGTCAACCGCAACAACGGCGCTCAAGGTG

TGGAGATGATACCTCACCTGCAGTTCTGGAAGGAGCTGCCTTCGCTCATAGTGGAGGGGTGCGTTTTCTTCGTCCAACTG

GTCACCTGTCAAACTGGACGCACGGCGAGTTCCTACGACAACATATGAAAACGTGATCTCTACCACTTCTTCCTGTCACA

TMATCATCMACGAAAGCGTCTGCTRATGTACTCCRTATGATATTCACTCTGAGTGTAYTSATACTTCRTGAAAATTATGG

GTTGCTGCTTTGGCAAAACGYTTTGTKGGCCAAGTTGGTTAATGAAGGCAAGAAGAAAAAAAATCGTAAAGACATTCTGT

TGGGCGAGTTGGTTAATGAATGCGAAAATGGAAAAAAAAATAC

>CK189403_bb No definition line found

CACCTGGTCGAGTGGCACCGCACACCCACCACCACCGAGACAGACGGCTTCCAGGTGAAGCGGCCGGGGGACAAGAACGT

CCGTTGCACCATCCTGCTCCTGCTGGACTACCAGCCCCTGCAGTTCAAGCTGGACCCCCGGCTGGCACGCCTGCTTGGCA

TTCACACGCAGACGCGACCCGTCATCATAGCCGCCCTGTGGCAGTACGTCAAGACCCACCGGCTGCAGGATCCACATGAA

CGGGAGCACATCAACTGCGACAAGTACCTCGAGCAGATCTTCCAGTGCCAGCGCATGAAGTTTGCCGAGATCCCCCAGCG

CCTGCACCAGCTGCTGCACCCACCCGACCCCATTGTCATCAATCACGTCATAAGTGTCGAAGGTCCGGACACTAAGAAGA

CGGCATGCTATGATATAGACGTCGAAGTGGATGACCCACTCAAGGCACAAATGAACAGCTTCATCCTGTCTACAGCCAAC

CAGCAAGAAATTCAGGCTCTGGACAACAAGATTCACGAGACTGTGGAGACCATCAACCAGCTGAAGACCAATCGAGAGTT

CTTCCTGAGCTTCGCCAAAGACCCCCAACAGTTTATCAGCAAATGGCTGGTCTCTCAGATGCGAGACCTCAAGACCATGA

CGGACGTAGTGGGCAGCCCTGAAGAAGAACGCCGTGCGGACTTCTACTACCAGCGCTGGGCCCAGGAAGCCGTGTGCCGC

TACTTTTACGGCAAGGTGCAGCAGCGTCGAGCCGAGTTGGAGCAGGCGCTGGGCATTCGCAATGCCTAGTTAAATAAATT

ATTCTTTTTTCAACTGGCATGGTATTGCTCCAAGCATGCATGCTCTGCCTATGTTATTTCCTTAATTTAGGCCATCCTAG

TTCTTGTTCGGACAACAGTGAGGATATGTAGGTGGGGGGGGGGGGT

>CK189404_bb No definition line found

CAGCGAGTTTTCATTATCGCCCTTTATTTAGTATCTGTTTCTTTCTAATGTAAGTTTAGTACAAGAAAAGCGTATTCTAG

TGGGGAACGTGTTGCTTTATGAGCAATCACGTCTTGCGCAAAAAGCGCGTATTTTTTACATCCAATAAATAAATCTGCTT

TGCAATTTTATTTAAAATCTTAATACGTCAGTTCCTTTTCCACGACTGCCTTGGTCTTTATTCCTTGTAGCACGACCCGA

CCTTCAATGGCGGCTTTGATTGGAGACATCTGTTTGATGTAGTCGACCAGAAGCTCGTACTCAACCGGACCTCCTTCGCT

GACGTTGGCGCACACGTCGAAACCATCTCCACCCTTGGCCAGGAAATCGGTCGTGGCGATGTTGTACACGGCGTCGTCCC

GGACATCTTCGTATTTCGGAACGTAGCATCTGGTGCAAAGGATCTCTAGCCGCACAAGTCGATCGTAGGCCGGTCGTGTC

ATATCAAACGCAACTTTCATACCCGACACGTGAAGGAAGGAACCGGCCGGTGAAGAAATGTTGTACGTCGCCACCGAGTA

CTCCATCATCTTCCTGAGGCTCTTTCCACTCATAGTTGCGACGACGATTGTCTGACCGAAAGGCAGCGCTGACAAGACGT

CTCCCATCGTCACACTCGCTGTTCTTGGAATCGGTGCACGCAGACTACCTCCGTTCACGACAGCCGCATTCACGTTCGAC

CACACGTCGCGCGATCGTGGCTTCCGGTTGGAATAGTAGTGGAAGTACGAGTCGGCCGCCAGGTT

>CK189448_bb No definition line found

TGGTTTTACAGCGTTGACGAGTGCGAATGATAAACGCAGAAATAGACAAGCGCCGYTTTTCAATTTATTAAACARRARAC

GCGTGCACACATGCAGTRCAGAGGGGGTAAAGSAAGTTCAACAAACCTACGTATGMGCTGATTGCATGCTCTMACAATCT

TCGGGAGCATAGTGACCGGACATTTGATTACACAAACTTCAAATGAACAGCACAGCCCCCTGTCGACTCGTATCACTTCG

TACATTTCACTTGTTAACGCTGTAAAACCATGCATATCTTACACTACCTTTGATGATAAACTCTACCTAAGGCGATCTTC

ATATTATAATTACCATGACTATCATGTGGTTTTTTTACATCGGAATCTTTTTCAGTGCACGTAGCATGAACTTTTGTGGT

AACTGTACACCTGTAAAACACCTCC

>CK189449_bb No definition line found

GCGAGATCGACATTCTCCTCAAGCACCTCGCTATAGGCTTGAGGGAAAACCGCAGTCTGTCTGAACTTGGCATTTTGCGT

GCCCCTGACTTTGGCGTGGTGCGGGACCCAGCCATCATGCAGTTGCTCCGAGTAAATCGAATGGCGGCGTCGCTTGCGGC

GAGGTTGCTTTCAGGGGCTAGGCTTAGCACATGGGCTGTACATTCACTGGAACGAATAATATCGTGTGATTCAATGATCT

CAATTTTACAGTCTCATTTGGCAATAGGCAGCGATGTCATAAATGAGAAACTCTCTGAAGTGCTGGTTAGGATAAGAAGC

GAGAACTTCAAGTTGGAGAATCCAGATAAGCAATGCAGATGCGCTTCTCCAGCGCATCATGACAATAAAATTGAACTGAG

AAATGCTCTTCTTTGCGAGATTGGGAAATATTTAAGCCTTTCTGACGCTTCCTCGAAACAGTAATTGTTGAAAAGGGCTG

CATTTTCGGCCTTACGTCAATAACCTTTTCTTTCAACGTTTCCCCAATATTCATATCCATCGCCCAAGAAAGCTTGTTGT

TCTCGTTTCTTCTGGTATAAACTGAACTTCGCAAATGCCCAATGGTTCATTGGTTGGATTTCTATTATGCACTGTCTTAC

AGTATGAACTTGAACGTGTAGTCTGTGGTGCTGTTATGCCAAAAAAKCTTTCGAAGTGCGATGAAACTTCATCTGTGCGC

TGCTAAAKCTTCATTTGTGAMATACTTTATGGCATYTTATGTAGAGTTTTAGCCCATAAACGACGTTTTGACTCCCCACA

TTTCTTCAYTAGGTTGGCGACGTCATGYTAACGAAACCTGAAATCYGATCATTATAACTGGTTAYYATTAAAGGTAGAAA

CATACTAGAAGTCTTATGCACTAGCGCGGGCTT

>CK189499_bb No definition line found

ATTTTTTTAATTCGAGCGCCATGACTCTCACGGATGAGATTGCTGGTCTGGCTACTTGTCTTTTGAAGACCGAGAAGCGC

TGCTGGTTCGAAGCACTTCTCAGAATAAAGGGCACTAATGACTCGAGGTTTAGTGATACACTCGTTTTTATTGGCCCTCA

TGCCGTCACATATTCCAGGCATAAATCAAACAGAARGGCGTGCTTCGCTGCTCCTGYGCACRCTACGTTCACAATACAGC

TGAAGAAAGTTGTTCGCGGTTTGACAAAGGGTGCATACAGTGCGACGACGGGAAAAAAACAGAAATAATCAGCGGAGAGG

TGAATATTCGGTGAGGTTTTCATTACCCGATTACACCAATGTCAACCAATTTCCTGCCATCGCCCAACTTCTCACAGTCA

TTCGCTTTTGCACGAGCACCATGCAGTGCGATAGTGATTGAAGGAACGGCGAATCACAAATGAAGCCTACCGAAGGCATG

AACTCGTATTTGCAAGTTAAAGTAGATTACTGATTAAGAAATGTGCCTTCTGAGCATTTTTTCTTTACTACTCACAGTAC

GTTATGGAGTTTTTATTAACAGTATCCTGTGTTCACATAACCCTTGTAACTAAAATGCCATTCCTAATTTGCGGTCAACA

TGAACCCCCTCATATGCATTGCGCCGATGCATATGGTATGGCAACCCACAGGCCAATTTAGAGAAACGTTTTTACGTAAA

TGCGGTTTGTAAATAGGTACTCTGTATACTATTAGGTCTTGCGCATTAGATCGTTTAGACTTAGTCACTCGGCTGTTTCC

TTCGCGCGTGGTCCTCACGTCGTTCACCTCATTGTATTAACACTCGATAAAACTGAACCCGGAGACATGATAAC

>CK189512_bb No definition line found

ACCTTGCAATATGATAGTGTATTTACACACTTGGACAAAAAACCTGTGCATAATATAGTTGACTGCACAGCATTATATTG

GCTTGCACAACTTCCAAAACCAGTTTGTACATACCAAGTTCATATACATTGATAATCAAATTATACGTAACATAAGGCAA

ACACACATAATGGCCACATTCATGTGAATGTAAAAAAAAATTTCCAGCCAGGAATCTTATTCCTGTATTTACAGATGCGG

GTTTAACTTTTCAAAACAATGATAAATGCAAGTTTTGACCTATGAATGAGTGAGAAAGAGAAAAAAAAAARAAACTTAAA

GCATGTGTTATGGTGATGCCAAARATATAAAACACAARACTACTGCTACCTCACATAAAAGAGGTCTATTAATTATTGGA

ATGCAAGGGCACTTGCAGCCATAAACCCCCACATTCACACTACACCAGCCACACAAACTTCATTCCCTACATGCAGCTRT

ACCAATCATGGACTCTCTAGCATGTTCACACCTCTCGCATGAAAGTCCACTCACAAACACATCCATCATGCAAATGCAAA

AATTGCTATATTCAAATCCTAAAACAAAAAGAACTAAGCTAGTTCCCAAAGTTCGATTCAATCTTGCCTGCAATATGCAA

ACAGTGCAACATGGCATAATTAATGCTAAAAGCACACTACTACTTTTGTCAGT

>CK189591_bb No definition line found

GTGCGTGTGTTGCGAGCTGAAATACCACAACATCGCGTGACAAGCCTGCTTTTTGTGCGGGCTTGAAGACCACACTAACG

AGACAACTTYCGTTTGCAAGATTTTATTTCATCACTTTAGTAGGTCTGATCATGTCACTTTCACGTGTGCACGAGGGGAT

GCACACACGCTAGGCGGAAGGATGTGATCTGCTCCACACACAGGGGAGAAATAAGAGCCGGCTAATGCTGTGGCATCTAT

GTTACACACTGCTCGCTGCACAAGA

>CK189592_bb No definition line found

CGAGATAGGCATGTTGAGCATGAGTAGCTGAATTCGTATTTCTCTGCACAGCTGCTAATGMTATGAAGCTTACCTACATG

TAATCATGTGGAAACTTGTGGGGAGGAAACGTAGCTGCTGCCTTTTGAAAATACAGCATCAAGGCTGACCAAGCAAGTAA

AGCTTCGATTCCTAAACTCGTAGTCAGAACCATGTAAAAAAAAAAGCCGCACAAGCGCTTGGCTGCTCAGAATATTTCAA

ATCCATGCAGTTTTTTCTTAATGGTGCCATAGCAACTCTAGTTCACTAATTTGCAACCCTTTCATGCCGCGATCTGTATG

GGGCTTGATACATCGGTAGAAACATGCAACATTACTGTTGAGAATATCCATGTCGCCGTTCGCTATAAGAGAACCAGGTG

GTCATCAGGTTATGCCTGCATTGTGAAGGAAAACAATCGTGGTTTTTATATGCGCCTATGCGGGCCATGGAATGCAACAT

ATGCCGATACTTCACACTGTGGCACTTGAAAACAGCCGCCTGGTTTTTGAGGCTTGCTACCAGCAACTACAGATGGCCAC

TCTTGGTTGTGCGCTTGCGAATTGATCATGCAAGATATGTACCATCATGGACAAATATAATATGTACAGTGTAAGTAGGA

CACCTAGAATGCCAACTGTCTAGCAAGGTCTTGCTGTGCTGCAATTGTGGAGGCACTGTACTGCRGATAATTTGTAAAGC

CCCTATTTATGCTTATTTGTCCATGGTAGTGCAGTCAGTAAGGACTTTTTTTTCTTTCATTTCCTGTTGCTACACAGTAG

CATTTACATTCTTGGCTTTAAGATCACACTGTGATAACTGTTTGCAAGAGCTTGCAACTGTGGTGTAAGTTGTGTCCCAC

TTTTTCTTCTTTTTTTYWTYTTKTKYWKCTATTCTTGTGCAGCGAGCAGT

>CK189620_bb No definition line found

TTTTAAGGTTTCAATAACATTATTTATTAAACATTCAACAATCATTTTGCAACACAAGATATGTTATATTCTTGCAAACC

AATTGTTTTTTTATATTCATCTATTGGTCATTTATTAATGCACAGCCCATGTTGTTGACATAGCCCACAATTACGACTTT

TTTTAGTCAGCCACATAGCAATGTGGCCAATCACCGGARATCWACATGCCAGGTATTGAAGGCTAGGTCTTGCCGGTTCG

CTTCAAACCTGTAGGAAGCTGCATTTCAAAACTGTTGTGCTTATAGTTTGCTACCATAGTTCTTGCTTGACCTTGAAATA

TCTCCCCACAGCACCGAAAACACARAARARAATCACTGTGGCATCGGCTATGACGGAAGTGCGGCCCAGCCAGATCACTG

TGCACAGCAGTTGAACTTCACTTCAACGGGACCTCTAATTATGCGGGCAAGTGCTTATCATTGAATGCCTGTTTTAGGTA

ACACCTAACGCCTTGAATGTAAAGTTTGAATGACCATTGCACTGCCAGATGCAACTAGTCATGCCACCTGCTTTCAGTTT

TTGTCATTCACCCAGTATGAATGTAATGGGCCAGTGTGGAACTTGTCTCTTTTGGACATCTGCTCTAGGACTACAGATCT

ACAGCTATGGGAGACAGAGCACTGTCATGGAATGTTCACTACTAGTGTTGATTGGCCCTCATAAGGTTCTGCATTCACTA

ATCCTCTCCACCAGAAGCTGGCATTCTATTTCCATTCCAATATATTTT

>CK189621_bb No definition line found

ACCTTCACTTTGCTTGTCATATATTTCCTGTTGTCTTGAGCTGACCAGAGCCGGTAATTTGTGATCCCCTATCTCCTCAC

TTTTTTGTTTCTTTTTTTTCCTTTTTGTTTGCACCACCTCAGTTCTGCGAGTCTATTTGTGGTGAGACTGAGGACGAAAG

TGATTGTCTGCGTGCTCATGGGATAAATACCAAAGGCTCGCTCCAAGAACAGAAAAGGCCACAAAGTGATTTGGTCACAA

AGAAGGCTTTTGTGCTCTATTCTACTCGACCGGTGAACGGACACTTGTGAGTTACAGCTTTTGTTCTTCAAGTCTGCCAG

AAGGACTTCTACGAATGAAGAAACTTCCAGGTCACATCCAAAAGTCCCACAAAAGAAAGGCCTCACGAATGTTCAGACTC

AAGCCTGTGCTCCATGGCAGCATGCAGCGCGGCTCATTACGCTAGCTGTGGTGAATGCAGGTCTACTGGGCAGCTGGAAG

AACTTCACCATCAAAACTAGCCTTGCCTATGAATTGCCCCAGATGAAAAATGCCATTCTGCCATGGCGTGCATTCATGGC

AACACAGGAACGGTGTCTTTTCTGGGAGTGTGGGGATCGGTTGCAACATTGCTTGATAGTGTGTAAAGAAGTGGCTGCTG

ATTGGCTAACGAGCATTCAGTGGCGCAAAGGATGCTGCGAAGATAGCGCAATGATTGTCATCTTGTAAGCAGCTGGTGGA

AAAACTCTGGAGGTTGACTGCTGTGTCAGCCACTGCCAAAAGTGTTCTATCTGCGAGTTTCACCCGGTTCGGTGCTAATT

TGGGATAATAGTACTAAAATAAGCAAGTATTATGAGTGCGAACATGTGAGTGTGTGGCAAATATACTAAGACACGATAGA

GGGCACTTTGTAATGGCTGCTAAAATGTACAGGGTGCTCCGCTAAATGTCAGCACGCTTACTTTGCTAATCCTTGTAAAG

ACTGCATTTAAGTGCTTATTGTGGGCCGT

>CK189655_bb No definition line found

CGACTTTAAACAAGATGGCGCGAACACTGGTGTTTAAATTGATTCTCTACGCAAGTGGTTTGAGTGTGTTAGATGGCGCT

TGTTGGTCATGTTTAAAACGGTCACACAGCATAAGAGAGTTTCTCTTTACGAAGGAGCCGATATGGACGCACACTACCTC

TGCAAACACCAGAATAAGATGCTTAGTGGACAACGTTAACGCAATAGAGAAGAGATCTGTAATCTTCACACGATCTTGTT

ACAGCCAGGGACATAAGGTGTCTAGAATATTCCAAGGAACTTTTGACAAGTTTCGAAAAAAGCATATGGACGTGACACCT

AGAGGCATGGGCACCTACTTTCAAGAGGACATGCTCTATATGCGTAGTGATAAAAGCTGTGCTGTGATTATGGTCACATA

TCCTTTTTGCGGTAGATATATCGTCTATGATCTCCGAGTCAAGAATTCCCACATTCAAAGACCACCGCATCCCAAATGCG

TTCAGGAATACCTGAAGTACGAGAAGTATGGCAAAGTTATATATGACTCAAGCTGCCAGGGTATACTTGCAGCAAAGGGA

AAGCCGGGGCTATACCAACCAAACGATCGCTGCCCGAATCGCATTAACTGACTGAAACGCATTTTCGCGACATCCGTAAT

ACAAAATAATGCTACAATAAACATGTTCCAAGCCAATGGGCATTCCAAGACATTTATGTGAGCAAGAACCAGGATTTTTC

CAGCAATAAAATACTCCAATAGGTCTTTGCTCTTCACCGCAAAAGTGTTTGTGTATGAGCCTGGCGCCTCGCAATATTTT

GGGGCTGTGGCTACCTACTCACTGCGTTTTTTTCATGATAATAATCATCATAGGTCTTGTAGTTTCAATTGAAGGCCAAA

GACCTCTCGCATGTTCCGTCAATAAACCTTATAGTGTGCGTACTGCGACCACGTTATACCCGTAAACT

>CK189708_bb No definition line found

TGAACCAACTGATCTGAAAGCATCTGAAAGCGGGTATCATGCCGGCATACGAAATGACTATGATCTTACGTAAGATGTCC

AAACCAGAGATCACAAGTGCACTGAAACGCACGGGAGAGTACGTACTGAAGAATGGAGCCGTCCTCCGCTACATCCAAAA

CCTGGGAACCAAAGAACTTCCCCTCAAGATGAGCAGGCACGGTCGCACAAATTGGCACGGGAGCTACTTCCTTTTCAGAT

TCGATGCATCACCGCAACTTACAGTGGCAATGCGAGGAGAGATCAAACGAGATGTGGACATTATTCGCGCCACATTTATT

ACCCTTTCACCGCCAAAGGCCATCACATGCACCCTCGAAGAAGAGATGCAGCCGCCTGCGTATCGACCMAGTGTGCAGGC

ACTCATGRCCCAAAGCCAAGTGAAGAGRAAGCAGACCTTTGAGAAACACAYGGATGGTCCAGTCTGACCTGCCGCTTTAG

GCATAGTRCAGTGTTTCCACATCCCTTAAGCTGTCATACCTAATAAARAGAAGTTRCATGATCTGTTACAGGTGGCACCT

GAATCTGGCACTTAGTTTATTATACTTGACACAGTGCCAGTAAAATGGCACTTTTTTAT

>CK189732_bb No definition line found

TAGAAATATCGTGCACGAAAGGCGATTGCGGACATTTTGGTGACTCTAGACTGCTTTTTGGTACGAACCTACCTCGAGAG

GTCCGCTTCACTTGCTCGAGCCTTTTATAAAAGTACAGTCATTGGATTCAGAGCACGGAACCATGGCGGAGAGCGGCCAA

TTGCAACTTTCTCACGACGAGCTTTTGCAAGCGCATAAAAAGGAACGCAAAGAATTGCAAGGAAAAGAAGCGGCAAAAGG

AAGAGCAGCGTGAAAGAGAGATTGCAAAGCAGGAAGTGGAAAACCAGTTTCTTCCACGTGCTGTGGAGGAAAGAGAACTG

CGGGAAATTCTCGAGAAGCTTGGATTGACTATTTACGAGATTCCTTCTGATGGAAACTGCATGTACAAAGCCATGGAACA

TCAGCTTGGACTTTTTGGTGTCTTGAAATCCATGAATGAATTACGGCAAGAAACTGCAAAGTACATGTTGTCTCACTCTG

AAGAGTTTCTTCCTTTCCTGACAAGCAGAAAGAGTGGCGACGTGATGACTGCAGAAGAATATGAAGATTACTGTTTAGAG

GTTTCAAGCACGACAGCATGGGGTGGCCAAGTTGAGCTCAAGGCTCTCTCCCATGCTTGTAAGGTGCCCATAACAATAGT

TCAAGCTACTGGACCTTCCATTGAAATAGGAACAGAATATAATTCTAAGCCTATCCTTCTAAGCTACCACAGATGCTTGT

ATGAGATGGGAGAGCATTACAACTCTCTAGTCCCTAAAGAAGGCGAAGTGGATGAAGGTGACTGCGTAGATCTGCAGGGA

GAGCATTACAACTCTCTAGTCCCTAAAARAAGCGAAGTGGATGAAGGTGACTGCGCAGATCTGCAGATCTAGTAAGATGG

AATATTCCAGTCAAAAGKTWMTGTGAAAAAAAAGAGTCTCATTTGTTTTTTTATATGAAAACACGT

>CK189823_bb No definition line found

ATTGAGCGACGTGTCATGAAATAACACTAAATATATATGTAAATGTTGCACGCACACAGACATATGTTGAAGAGTTGCAA

ATGTTTACATAACCGGTTGTTTGTTCTTGCACGACGAGCCCCGTTGTGGCGGTCTAGTGGCTAAAGCATGCAATGCGGCT

GCTGACCCACATGTCGCGGGATCAAATTCCCGTCTGTGGCGACTGCATTTTCGATGGAGGCAAAAATGCTGTAAGCCTGT

GTGCTCAGATTTGGGTGCCAATTAAGGAACCCTAGGTGGTCGTGGTTTTCCGAGCCCTCCACTACGGCATCTTTCATAAT

AATATCGTGATTTTGGAATAATAAATGCCACATGTTATTATTATTACTTGCACAAGGATGACAACGAGAACATAAGCATT

AGCAACACCAGAAGCGATAAGCTGATATGGAGTGCTGGTGCTCGTGAAGATGGCCCTGGACACTGCCACRTCAATCAACW

TGAGTGCATGGRACCTAACTACAATTGACKTTAACCTGACAGKATTGCAAGTGGSCGGGCAGGTGCCATAAGTGCTTGCA

GTACACRAMARWTCTTCGCATWAARTAACMGAAAATKCTGGGTCAGCAAGGGWATYGMACCTGAGTCATTTGCGTGGCAA

GCGGTTGTTCTACCACATAGCTACGTGTCTGCTTGTTAATATAGTGAGAATAACTTTCTCTGCT

>CK189824_bb No definition line found

GAATACCCTATGATAACAGTCCTGTCGGTAGGGTTTCCGAATATAAGTATCTCAGTGTCCTTTTCACACATGACATGTCA

TGAACCAAGTACATTGACTATTATTGAAACTAAGCATTAAAAATAACTCTGCCACCTATGCCGCACTGTGTCCAAATCCC

CAAAAGACACAAACTTCCTCTTATATCAATCACTAATTCGTCCTGTAGCGACTATACGTCCCCTGTCTGGAACCCGCATA

AGCAGTGTAATATTGAAAGTTTGGAGGCAGTGCAGAAAAAGYAGCAAGAATCATACGKCATCATTATGAYCGCGATTTCT

TYGCYCTTTTTTACWCTACCTTCAATGCAATTACCTCCATTCCTTTAGCCGAATTGAATAGCTGTTCTTGCAAAGTATCG

TTGTCTCGACAGTAAACTGTCAAACAACAAGAAATGCATAAACTTCACTCTACAGTCAACGACGAGAAGCTCTCAAAACT

TAAATTTGAGGCCTTATTATGCTTCAACTAACATCTTCAAGTTCAGTCTTTTCTACCTTTCCATCGAAGACTGAAATTCG

TCACCTGGTTCCGTTTTTCATGCCCATCGGAATGTTTCGCAGACGAAATCCACAGCGCATGTATGTAAAATACACCGATT

TATATATAAGTGGTACTTGCTTGATAGATTTTTTTTTCATGTTCCCAATATTTTGTATTACTTTAACTTTTTATGTTGAC

ACGACAATATTATGTTACCTGTTAGTGTTTTTCTTATTGTGTTTACTTATATTTCTTCGTAATCTTGCATAACCACTCCT

GCCATGGTGCACCTAGTGCTGCAGTATGTGTAAATAAATAGATAAAATAAAGAAAATAGGCACAGTAATCTGCAAGCCTT

TT

>CK189829_bb No definition line found

TGCAATTCAGTTTTATTTCACACTCAACAAGATATAACAGAAAGTATTCTTCGACGTGTCGCTTACAACCTCTTCTATCA

CGTCACGTGGTTACAACTTTGGCCTCGTTTTGCGAACGCTTCGAGTTGCTYAGCGTCCGCRACGCCAAAGAGAGMTGTGA

TTGGTTCAGCGACAAGCACGCGCACGAACGTCACGTGATTTCCCGTCGACTTCGGTCACGTGCRCACACAAACACACCAA

CSACGACGACRATCTGCGCACCTCGTTTTTTTTTTAATTCACGCGGACAGACAGAGGAGACCGCGAGTTAAAAACGCGTG

ACGTCACACCACTGGCTGCACTTACAGGCGTCACACTGTCGGAACGATTTGAGGCACGGGTTTTTTCTTTTTCTGACACT

GGGAAAGWTGTAGAGCKGTGAATGCAAGYGCAGTGAGGATTGTAGAGAGAAGTTGTATGTATAGAAACRGYGGCGTCGTC

GGTACGACCCTGTATCCRCGTGCAGACGTYGGGCACAGTGGGGCATASTGCTGTCWCGTGYGCGGAGCTCYTCCTYGGAG

GCCTTGTTCRTGATCCGACACATAAATCGTCATGTTGCTCGTAAGCGTGTGCGTATCACTATTCCGATTCACCGAAGTGT

AAACAAAAAAATT

>CK189839_bb No definition line found

GAAGTACACCTAAGATTCAAATTTTATTTACACGAAAGATAACAAAAAAAAAAAAAAGACATCTCGCAAAAGTGGCAGTG

GAGTGTGCTGGTTGTGCAGTGCATTGCCACAGGCACGAAAACAAAAGGAWTGGGAGGCTTAGCCCTTACCATTCCAAAAA

ATGCATTCAATCATTGCTGTATGAAGAATGCRTCGRTGCACACATATAAAATTGAAAAATKGGAACATACAAGGTATTTC

TTGCRGGCTTGTAAAGRAGGCTTTAAGCAGACAAGTGACTTGATATTKAGTAGATTGCAAACAGAATCCTTTGAAATACA

TGATGCACTATGATAAATGTTGCCCCACAACAAACTAAGCTTACTAGTTGGTTGTGAATAGAAAAGTTGAACTGAGGAAC

AAGATCAGTTTCATACATTATTTACAGTCCTTGAGCACTTAAACTGCACCACAAATTAGTAGCGAACTTTGCCGAGACAA

AAGTAGCTAACGAAAAGAACTTGTTGCCTCARACAAAGGTGATCTTGGTTCTAGCCTGGTTGACCTGCCAGACATTGAGT

TCCTCATATTCTTCGATGGCTTCTTCAATCTGGTTGGGACGGAATCCACGTGAAGAGCAGCGCTCGACGGCATCAGTCAT

CTTCACAGTCTTGCCTCCAGATTCTGTGACCATGTCTCGGATGAGCGCGAAGATCTTGTCAGTTACTGTCTGGACCCTGC

CGCCAGACTCGGTGTGCTGCGCAAGGGAGTCCCTTGACATTTCCATGAGGCGCATGGCCTCATTAACGTCATCTTTTTCG

ACAACTTCAACCAGTCGAAGCCTAGCCAGAGCTGTGGAAAGGCGCAGAATCGC

>CK189944_bb No definition line found

CCAAGCAATGATTTAAAATCAAGTGCTGCAAGCCTAGACTCTTTAAGGGTAATGTTGCCTTGTAGCATTGTATACCTCGA

AAGATGGGAGAATGTTCTGTCGGATTTTAGCACCAACATTGTGAAATGATGCATCATTTTACACTCTTCATGCTCAAATT

GCTTAGTAGGACATTCTATRACCCTGTATTTAATCTTAAGCTTCTGAAATCGTTAATTTTATATGATCAAGTTTTGCACT

TTCTTCATACTATTTCTTTTTCTCTACCACGATTTTTCGTATTTGATATTCATGCCTAATGCCTAAAGTTACACAAGTCT

GAAGCAGCTGTGAATTTGGGTGTGCCAAGTTTCAACCATAAAAAGTGCACATCTTGGTAACCTCATGCCTGCGCCGCCAT

TACTTAAAGGAGCCCTACAACACTTTTTGAACACGGTCAGAAAATGCCACCAATCTGTTTGGTATAGATGCCTCTGTAAA

CTTGTGAGCCAAATATTATAGAATTGCATGCAGCAGAGAGTTACAGTCGCGTTTCAAAGACTGCTAAAAATTGGTCACTC

TTCTCCCATCGAAGTAAGTCATGCAGGGCCTCTTGATAAGTTAACGGAGGGAGCGATATGGCCATTGGCTGATTTAAACA

TTCTTGTGCTGCCTAGTTACCACTGCTGCCACAGGGTGTTGCCGCGTGCTGGCGTGGAGCTCTCTAAACTGCTAAAATAA

ATCATAGTGGCCTTAGCGGGTGATCATCTGAAAGTGAGCCAACTCGTTCCAAGAAAAAAATATATATCAGATTTGCTCAA

GGTTATAACATGCGCTGACCAAATTTCTTTCCCCTTTGTGATCTCCTCACTTGTGCAGCTTGGAAAGGTGTTGCCAGGCC

CCTTTAATTAAGGAAGAACAAACACGTTCAATGGTATTGCCATGATTTCTTTGTTTGTTTGTTTTTTTACTGGCTAGAGC

TTTGTGTG

>CK189985_bb No definition line found

GGTGTGCTGCGCGGGCATAACAGCATCATTTATTGTCAACGCAGTATCTTACAGAACGTAACCGTCGTTTCAGCATCACT

AAAATAGTCTTTTCTTTTACATACTTTTAATTCTTACAGGCTGACTCATGTGTCAATYCGCACTGGTATGATGTTYACAT

ACACAACAATGCACACAATGATTCGAGCARTCATGACCACACACGAAAAAATAAAAAGCGAAAACACGCAGCTATCGAAG

AGAAGAAAGTTCAGTGGCACGATAAGAACACACTTGCATCATACAAGCAACACATATTTCTTTAAAAGATTCGGCTGAGA

GACAACAGCGCAAGCGCACCCACTTGCCTTCTGGTGGCAAGTTCGTCGCATTAACTTCTCCCGAACAGTCTCCTCCCGAA

CAGTATCGTCACTTTGCAACCGCAAGTGGCGTTGAATTCAACTGCATCACAAGCGTCAGCGTTGCTAAGTACGTCAACAA

GGCTCTTGTAAACAACGAACTCGGTAACATCACTCCCACTTGCTTCAAAACTATCTTTTTCACATCACGCAAGGTTTCAA

CATCAGCGATGGCACAGTGTCTTCTGGTAAAGAATGAACGTGACGGGATGAATCAAATAGAACTCTCGAAAAATAATAAT

GTGTGAGAAAAGATACGGCTTAATGTTGACCAAGATTAAGCAATGCGGAGCTGTTACTTGTGCGGAATATGCTTGCATTT

ACGTAGGGTTCTTCCCAAACTAGCATGTCCGTTGAAAACCCTGAACTATTCGACAACCACTTATGAAATGATGAAGTCTG

TTAAGAAGGGCAACATAAGTTATTTCAGC

>CK189986_bb No definition line found

CCACGAACACGCTGTGATGACGTTGTTRGTGTTTCAGAGCATCGACCAAAGACTGGGCAATCTTTGGTCACGTGTGTAAA

GCTCCCGATCGTGTTGCCAGTTTCTAGGGTGCCTGGCGGGAACTGTTTTTAACTCGCTTGCTTTTGGCCCCAAGAACTGT

TGCTCCAGTTGCTCCAGTTCCGTTTCACTGTCTGTACCACAAACAAGTAGAGTTTGTTGTTGACTTTTCTGCACGAGCGT

TTATGTSTCACGTTGAGTGTCCCCCCCTATGGCGCTTGAAAAGAGTTGTTCATCGACCATGCCTCAAACTTYGTATTTTT

CTGTTTCGTCCTTGACATTCTTCTAAACGCTTAGAAGTCTAGAATGAACTTCTAATTCAACAAGAAAAATTCTTCATGCA

TCTCAATTTCTTCTTCTTCATATTCCCAATTTTCTCAAACAGAACCTCTAATATACACTTGTCTCAGCACATGCCATTGA

GTTCGCATTTTGTTCGCACTGATCAGCGGTATGTCTGGTTAGCTGTATTGGTCAGATTGAGTGCATTAAGCACGTGAGTT

TTAATTCATTATTTGCATGCGTAGGTTGAGGCAGTGTCGTACCAAATTGGTGACTCGGCTTTTGCCACACTGTAGGGAAT

AGTAGCTTTCTTTTGTGAACCAGGCTGAAAGAAGATATCCAAAAGATATCGCTACTCYAACAAGCACTAAAATTTATTTT

GTGGGTTGTTTATTTTGAACCGACGAAGGCTCTTTTCGAAGTACATATTTACTGTTTCTGCAAAGCACAAACTTACACTT

ATTGATTAAGCATAGTATAAAGTTGAGAAGTACCGAAAGAGGCCAATCGCCATCATGACTGATCGGGAATCRCCGCMAGT

GCCGCTRAATGGTGAAGGTTATAGAAGTTTAGCACATATGTTACACGCGAACATTGAGGAGCAGCGCTTATTGCACTCAA

CGCGAATGACAAACGCAGTCATTGTCACGTGACGAGTAATATGAAAATTAATTTCTAGCTTGTTTGTAGATGCTCTGTTT

CGCTGCTCAAATCATCCTTAACAAAACAGATTATATTGGA

>CK190069_bb No definition line found

CACCACCATTTTTTTTCTTCTGATAGATGCGTTTTTCTGGTATCTTGACACTTCCGTGTTTCAATCACTGAAAGCTTTTT

TTTTTTATCTTGCACAATTTTTGTCACTCATGACTACAGTTGTTGTTGAAAAAGTGATTTCATAAACTTGTGGCTACAAA

TTCAGGTAGGGAAGGTCCAAGTTAGGCTGTGTCACAATAGCTTCTTTAGTAACAACAGTCCACCACTGGCTTGCCTCATG

CAGATGTTTGGGGAAGTGCGTGAATCCTTGTAGCTCCAGATTTTACCATAATCCAAGCAAGCTGCGTTGTATGAGTGTAA

TGATATTATGAGGAGCAGTGTACAGTGAAGAGAGCATCTATTCTTGGAAGTTGGAACTCCTAGAGAGCTGTTGTGAAAGA

CTATGCACGCTTTGGCATCTTTTTTTTTTTGTGTATACATTATTGAAGCAATCAAGTGTTGTGTTACTGTTGGTTTGCAG

AAATGCATTGTAGATATTCTAAAGAAGTGAGTTGAAACCAGAAGCCATCCTTACGCATAAAATTAGACTGGCCATATCCA

AGTGACCTCACTCGTGAAATCGTGATTTTGCGCAACTGCAGAATGAGGTGCCGCACACGGTGTAGTTGCCAACATTGAAT

GCCGTGGAAAGAATACTGGCCAGGAATGGGTGGGGTTTTTCATTTTCCCGAAAGGCAAAATGATTTTGTTGCTTTGTAAG

ATAATGACGTCTAGTCTAGCTATTGTTACTTACCCATTCAGGAGCGTAGCTTTTGTCAGTATTTTGCGAGATGAAAATGT

GTGGTGGATGACACTTTCATAACCTTGGCTACAGCCAGTTGCAAAACGGCGCCCGCCTAGTTTATCGTAGGTAATCGTCA

CACCTTGTTCCACCACTTATTCACTAATTGATTCACCTTTATTGCGTCAAATAAGCAGTTTTGGCATTCCACAAAAAAAG

AATACATTTCTTCCTTGTTGATTGCTAGCTCTACTTATCTGATGTCATCTTAGC

>CK190100_bb No definition line found

ATGTTATACTCATATTTCCATGAATCACACATGTGCAACCCTTTTGCTATACTGTCAATCATTTCCAGAATAAAAAGAAT

ATTTACAAAACTTTTATGCAAACATGCCACTTGTAAGATTTATTTTATTTTACAAAGGCTGGATGCCTTAAAAATGATGC

CACGACTCCATTATTCCCACTTGTGTGAAAAATATTCTCTCGTGTGATGTATGTAATAAACTTAGTTATCAGGCTAACAA

ACAAAATACAGTCGCCTGCGGCGATGCTTCTCTCCTGCATCAAGCATGAAAGACGGGGTTGAAAAACCTCAGTTAGTGCT

AGACGCTGACATCATTACACGCATGCCTACCTCACAAAAGGCACCTCTTAACAAAGGTTATTACGAAGTGTATGAAGCAT

CCCACCTGCATGTGCAGGACACTTTTTTATTGGCATGTGCAACGAGTGCCAAAAGCAAAGTGAAGCAAATTGTAAATACC

AAATACTTTTTGAACATTGAACAGCTGTTTCTATCAAAAATGTACAAAAAATGTTCACACTCTCATATTCGTGATTTTTA

CACTAACAAAAAAAATTGACTTTCTGGAACCAATAAGCAAATTCTTTACTTTAAAGCACTTTCAAAGAGAGCTTGAGTAA

TCTTTGCACGTAGCGAGTAAAGAATGGGCTTGCTGAATGACTGAGTCTGTTGGCTTTCACAAGCTTTCATTTTTGCTTAT

ATGGCACTCGATTAAATGAAATTCATCACACGTTTGTACCAATATCGCACTTATTTGGGCGTAGTTAAGTTGAGTTATTC

GTAAAATATCCGAAAAACATTTGTATGAACAAATAGTGGCTATTTCGTTCTATATATTAAATATTAGAATATATTTG

>CK190118_bb No definition line found

CCCAAAGCGGTAAAAGGCCATTGACGACGTGACTGTGTCGTGTCTGTGGTTTCCTCATTACACTGTGAATCCACAGAGAA

AAGCGTAGCATCACCTGCCTGAATCGATGGCTGACCAGAAAGATGCGGGACCGGATCTCGGGGCACGGGGCCCGCATTTT

CAATGGAGGCGAAAATGCTTGAGGCCCGTTAGACTTAGGTGCACGTTAAAGAACCCCAGGTGGTTGGGGTTTCTGGAGCT

CTCCACTATGGTGTGTCGCATAATAATATCCTGGTTTAGAGACCTTAAACCCCAGCAATTATTATCATCACCCATCTGAA

GCCCAAGCATTGGAATGCTCTGCCCCACGATTCCCCTATKCTCGACAYGCCGAGATGAAACTATTCCCTTTTCTTGTCGC

TTAAGTCTGCTTCTTTGCCTGTATTTGTCCCTTGTTTTTCACGAATTCTTAATGTACATGAATTTTGTTCTGCAGTGAAT

GTATACTGCAGCGGAAGTGTATGTATGTAGAAGAATTTTGAACTGTATTGCCCTCTCCTGCATGGACCATACTTGGTCCG

CAGTATGTGATAAATAAATAAAATAAATAAATGAATARSYAWRTWGGCAAAAGTTAGCCCGCCACTGTCACGTGATACTG

TGTCTATAAAAGATGGTGGCTGTACTACCACCCCAATGCTTAACAAAGAGTGTTTAATGAAGGCGAGTGATACAAGACAT

ACGAAAGACGTGAAAAATATAGAAA

>CK190136_bb No definition line found

TGACTGTTTTGTTTTTTCACACCATGTTTGCACCACAGTCACATATTTTTGGCGCGTTCCTCGCAGGCATGCAAGACTTC

AATTACTTGGGCTCAAACGATTTCGAGATCACAGTAGAGCTCGGCTGCCGCAAGTTCCCGCCGGAATCTGAGCTGCAGAA

AGAGTGGGAGGACAACAAACAGGCGCTACTCAACTTTCTCTGGCAGGCACACATTGGAATCAAAGGCTTGGTCACCGACA

GCATTTCGGGCGAGCCTATTGCCGATGCGGATGTGGAGGTGACCAACTTCACTGATGGAGAACCGCTACTGATTCGCCAT

AATGTTATTACGAGCGAGAACGGCGAATACTGGCGACTGCTGATTCCAGGCACATACATGGTCAGAGTTTTTAAGCAAGG

CTACATACCCGCCGAGAAAAAAGTGGTTGTCAAGCACCACCCACACACGGAGGCCCAGCGACTAGACTTCCAGCTGAAGC

CAGTTGCTGCACGGGCCATGGAAATGCTACTGGAATCTGAAGCTTACATGGACGGAAGCCAAGAGCACAGGCTGCTCTCA

CTACCACACCCACTGAAGCAGATGCAGCCCATGTGATTGCCACATCTCACCCAAAGCTTCAATATGCCGATACTTTAAGC

TGAAGGTTTCAGGAACAAAATATTTCACAGAAAAGGACATCTAGCTGTTGATAACAATGAACAAAAGTAGCAGCATTTGC

TTAAAGGCTCTCAAACATATCCAAGCAACAAACACAAAGAAATGAAAAGAACACTCATGTGCAGGTTTGTACATGTATGA

ACATGGTTTCAGAAGAAAGATGGTATACAGTATAATTGGCAAAAGCATTTGAAAGAGACTAAAAAATAGCGAAAAATACT

GCCACTTTATGAGCTTGAACATGCAGTGAAAGCTTGAAAAAAGTATTCCAAAAGGGTAATTGGCATTAACAGTAAACAAA

ATGCAACAAAGGCATAAGGTTCGCAT

>CK190144_bb No definition line found

CAATTGTTCGCTACACAATACTATGGCTACGAAACATGGACTCTGAAATGTCCCACCCCAAAAAGTTTCCAACACGTGCA

GTTGAGMATATACAGTWAAGTACACTGTCTAAGGGCTACCGCCAAATTCACAAAGCCCCATTATTTGTCGAACACATCGC

ATGCTTCGTTTGGCTAAGAAAAAAATAGGTATGTCCTTTTAGCATTGCTTAAGCTCTCTTACCGAGCACGTAATCATTTA

GTAGGTGAACTCACAGCTCCATAGTGAAAGATGAAGACTTTGTTGATGTTCGCCCTTGCACTTTTGGCAGTCACTAAATC

TTTTTCTACAGTTGCTAATGCGGGCTGGAGAATTCATGATCCTTTCAGCAATCCTAAGTTCTTACAGCTWGCGCACTACG

CATTGACTCGTCAACCAAACGCGGGTGGCATTCCTGGGGTTGCTGTGCGACTACATCAAGTCGCAACACAGGGTACCTAT

CAGGAAGGGGTCCGGTATTTGCTCAGCTTTGACCTCGTTCCACTAACCTGTTCCTCTCACCAAGTGCCAAGTCACAACAA

CAGTGCCAAAGCACAAGGCACAACGAGCTACACATCGGTATTTTGTATCATGGGAGAGGCGATGAACTCCTGTTCAGCAC

TCGTTTTCGAAGAACCAGCAGGCAACGCTATTCACATTTTAGGAATAGACTGTCTTCACTGAATCCACTGTATAATTTTA

CCAGTAGACAAGAGGCATAACAAATAAATATTCCGACTGCACTAAAAATAATGAAACTTAGTATTGCGTTCCACTGTAGG

ATGAGTAGGTCATAACACGTTCCTTTTTATACTTTTGTGGTATCAAGGTCCCTTCTACGCAAAAAAGCACGTCACAGCTC

AACGTATGTTGGGTGAAATGGAAGCGTTGTATCAGTGATGGTTAATTGTACTTTAAAAAAATTATGTTGACTCACCACAC

TGAAAATAAGAAATTACTAAAACATTTTGGAGAGCAAGAAGGCTTGTAACACTGGAGCCTTCCAAATGTCTTCAAACTTC

ACCTGAATGATGTTCATCAGAAAAYTTCTTCAAAAAATGTGGTTTCAGATTTAGTGCCAAGTTTGGATCGTATATTAAGC

AAAGAAGCTGGTAGGGGTTGCTCGATGTTGAATAGTATCACTTAAATATTTTCTTCCACAGGCACCTCCAGAAATCTTCA

CCTTATCAGCTATTTTTCATTTGTCTCTTTAAATATTGCACTATATATGAACATTTGAA

>CK190172_bb No definition line found

CCTAATGAAATATGCTTGTCGCATGTTTTCATAACTGGAACTGCAGCTGGGCTTAAAAGCAATCGTAATCCTAGAACAAG

GCAACGAAATAACAAGGCTTCATTCTCATAACGTGTTAATTACTTTGTTTTTTACCCCTGGTCAAGCATGTGATGGTATT

GTCATGGGGCCATGGCGTGGACGAAGACAGCATACTGTAGATCGACGACGGAACTGTTTATATTGGCCAAGCGTGAGGCC

AACAAACGAAAAGTAAGATTAGAGCAATACACGCTGGCACCGATGGCAGCGAACAGAGCGTCGGCAATCGATCAACTGAC

AAGTGGTGAAGCACGTTGGCACTTACACCCGAGCCGTCGAATATTCCAGCGTTATCGTTAGCAGCCGCGTAGCTTCCATA

ATAATGTATATTGTTTGCGAAAGCGGAGGGGATAATCTTAACAAAATAATCTACTGAAGTCCTGAAGCTTCTCGTGTACT

CCAGGCATGGGTTTGCGCTGAAAATATGTATAGTATAATGGGCGATAAAAAAAATGTTGTGGAAGGAACATACCGTTGAA

ACAAGTATCGTCCCTATGCTTTGAAGGAAGATGAAAGTACAATAAAAATATCGATATGTGGGGTTTAACGCCCCGAAATC

ACCATAAGATTATGAGAG

>CK190251_bb No definition line found

GGCGAGGTCGCTTCGCTTGTTTTTTTCTTGCTTGCTCTTGAGCGAGCAATGACGCCTGCTGTAGAGAACGAGTAGAATTC

GCTGAAGTGAATCTTGGATGTTCTTCCGGACACTGTTTAAATTCCATATTGTTTCAAAGACTGATTCAATCAACTATACA

ACAGTTTATTTCAACAGAAAAATCTGGAGTGGTGTCCATGGTTAAGAGTGGACGCTAAGCAGTCAGACTAGGTCTCTGAA

ATTCAATCTCTTACATTGGCAGAATTACAAGTTCCCAGCATAAATATGCGGATCTGATTATAGCAACAATATTACGTGAG

AAATAATATACTATATATTATTTTATGCAAGATTGCAAATATTATTGCAAATTTTCACGATACGGCGGAATTAAATAGAG

CCGAAAATAGCACCGCCAGTCACACAAAAAAGAGTGCGCGTGACTTGGAACCATGTTGCTTCCTTATAATCATCATCTAG

CTGCCATTTCCTTACACCTCTTACCACACGTTACAGCGTACGATACAGTATACGAACCATAACAAAACTGATAACTCAAT

ACGATGGACAATTCAATCACAAAATAACGACAAATATGTTTTGTACAACGACTAAATACAAACGGCGTATCGCAGTACAA

GCAATAGAGAAAAGTGGCGACCAAAGTTGCTTGCTGGCAATGGCTTGGCAAGCCAGAAAAAAGAAGCAGGAGATAATCTA

ACAATCAAAACAACATAACCGCAGCGCTTTGCTAGTATGGTGGAGTTTGGTTGGTATTCTAACTGGTTTATGACAAAAAC

AAAAACAAGTACTTTCATAATACCTTACTCAAACTCCACTTTCACGAATAGAGTGTCCTTTACAATGCATCCACGAAATT

CAATTTCATTCCAATATACCGCCTCATCCCAGCACCCGTCGTTCATATGAC

>CK190289_bb No definition line found

ATCCATAAAAAAAAAGTTATGTACAGAAAATGAACATGGGACTCTAAAGTTAAAAAAAACATCGTCTGGGCTAAATTGAC

CTACACAAAAACTCCCCATTGGATAAAGCAAAGCAACCTGGCTATATCACACAGCTGCTTGTCTGACGCTTGTCACGAGA

AAACACAAGCCCCAAGTAAGTAGGGGACATGCACAAAATTTCACGTGCTATGCATATTTCCTGTTGCTTAAGCACAGCCC

AGTTATGTTCAGCTCTTGCAACAGGAAGCTAGGAGCTCATTTTCAGTAGCAGCACAGAARAAAATGCATTATTCACATAT

ATCTGTTARACATTCATCTTGGGGCACC

>CK190403_bb No definition line found

CGTCGTGAGAGTTCGCAGTTGACCTGTTCCTCGGGAGTATGACTCGTTATTCGCGCAAAAAGACGCACAAGGGCTACACC

GCGGCGCACAAGAGGGACAAGACGAAGCGTAGGATGAAAGACCTCGACCAAATTCACGTCGACATGCAACCGGATAACGC

CGAGAGACTGCTCAACCAGGAGGCCGACTACGACATGCCCGGGGAGGCGCAGTTCTACTGCCTACATTGCGCGCGGTACT

TCATGGACAAGAACTCTCTGAATGACCACCTGAAAAGCAAGAACCACAAACGAAGATTGAAGTCCTTGGAAGAGGAACCA

TACAGCCAAGCCGAAGCTGAAGCTGCTGCCGGCATGGGAAGTTACATTCTTCCCAAGAGATGTAAAGTAGAATCGCAGCC

GCTAAAGGAGTAGAATCTGTGCATGAATGCCATTAAGCTTCGTTTCATCCACCCTCAAAGTGCACATGCTCAAAAGGAAT

ATTCGTTTCAATAAATATATGTTGAACAAGATCCTCAAAGGTATAATTCCTGACTTGCTCTGCTAATTGTGGCATGGCAA

TTGACACCACGCACTGCACCACCCTTCCCTGTGAGTTCAGTCTGCAAAG

>CK190619_bb No definition line found

GCCCCCACAATGCAACATAACAGAGCAGAAGTAAAGTCGTCAGAGAGGCCTGCAGGGTTCAAAGGCTCAGACTGGACAGG

GAGCAACAGTTCTCAACAGGAGAAATGGGACCATACAGTAAAGGGTTCTGGAATGACCCACTGGGATTGGGGCAAGCGTC

CTTCTCTACCTTCCAGGGCAGTACCAGATGCACCACCAAAGCAGCAGGACAGCGAAGTCAAGGAGGATATACCCCTAGGG

CATCGTGTCAGCAGCCCTGCACCACAGTTGCCAGTGATGGCTGTTGAAGTGCCTCAAGTTTCACCTACTGCTGGCAGAAG

TCGAGCTATTCAGCATCTGCTGAGCAAGGATGACCAAAGCAAAAGAGAGTATGAGGCACTCAAGGAGCTTGCTGCTAAAG

GAGAGCTTCAGGGTGGTTCCTGCTGCAAAGAAGGGATCCCCCAACAGAGTGACCTACAATAGGCTTCGCACGGAAACGGC

GGAGATGCTCGCCGTTGGCCGCTTYGATCTCATGAAACGGCTCGCTCTGGACCTTAGTGTCAGCTCACCTTTTGAAAAAA

AAAMTTAGATAAATAAAACTGYTGTTYCTCATMMTTTTTTGCAAGATGGAAGTGCCATCACAGTCACTCCTTCGTGCCTT

CATGCCGTTATTACGACTCTTTTTCTTTAGCTTGTTTCGAAGGTCACAAGTAACTATCTCTGTCCCACACTGAATGTCCA

ATTGTACACTTTAAAATGCAAAAAGAAAGAAAA

>CK190735_bb No definition line found

CACGTCGGAAGAGCAAACCAGTCGAGAGTGGATGAATGGAGAGGTGATAGTCTCTTCTCTTCCCATACTTCGAGCTTTTC

TAACCAATGTTTCCGGGCTTCCATGTCAGCGGATGATATATAAAATGTATGTGAAGGGTCTGAAGGGCAGTCTGTCAGTG

TTCAGATTTGTCAGGCCTGGTTCACTTTTTTGTGTGAAAAGTCAAAATCTTGTTAATGCCATTCCACAATCTTTACAGCT

TGTGCAGGCTGCGTAAGTAGCTTTCTCGGGTAAGCACTGTTAAGTTTGCTCTAAACACCGGCACTGTTAGTATGCATGAG

CTGAGGTGTGTGCCAAGGTGTCAGTGCATCATTTTGCTGCGGAACAACCCCTGCCGACGTGTTCTCTCAGTGTGGTCAGC

GTGTCAGCAAAATGGCGGAGAACTTTTTTTTTCAATAAACCTACCTGTATTCGGCACCTGTCACCAGCAAACACTCTGCC

TACTTGGCAAATGCACCCCATTTGATTTGCCTGCCGCCTGAAGTTATTAACTGGACCTGAAAAACTTTAGTGCTCTAAAT

GATGTGGAAATGTTAGATTAAAACAACTTGAATTCTAAACTACATTTCAGTTTCACTGTCTGTGACAGTGCTAACGCTGG

TGTTTGGCGAGGACTGTCAACATATTCTGGCTGAAGAGTCTATTCCCTTCCACATATGTATTATTTGAGGTGGGCTTTGA

ACAATAGGCGATAAAATGCAAAGAGAGTCTGCACTGAAATAAATGACAAAGCAAGTCAAAGAACTGCTGTTCAAAAGGAA

GGGCAAACTGACAAAGTGCTTTATAAAAAGATCCCCAACCTGTGGGTTTACAGGGTTAGCAAGGTATAAAAATTGACTTG

GTAGGGCACATAACTATAAAGTTGGTGTGAAGCAACGCTCGGGACGTGAATGCAGCTCTTCTAAGTCTTCCGCTTTGTCC

GGTGCTCTGTGTGATGTGCTTCACGCCAAATGCCAGGCTACCAGAGTAATATGCACCTTCTACCCAGGAATAAATTGGGC

TAATGGGATCTAGATGCCGATTTG

>CK190744_bb No definition line found

GATCAAACTAGAAACGATGAAATATGCGAAAGYGATTCACGCACGCAACTTCRAAGCTTCGAGAAGACCGGTGTAATTTT

TTGTTGGCGGCACCAACAAGCTGCTTACTCTGATCTTTCAGGAGTGTACCCCTAGTCTCTGAGGTAATGCCGTCCTAGTC

TTGAATGTCTTTTAAAGGGGAGCAATAATGTTTCCGTAATTTTATCCCCRTAAACTCCTATTTTAGCGAACCATCTCTCT

CTATGCCTCTTGAGTTTTCCTCAAATGTGTGATTTGATGCGACACTTTTTCTTCTGCACAGTAAAAATATCTAGGCTTCT

TATGCATCTGAAATTTTCTGCAGTACCCTTGAAGGACACTTCTAAGAGAAGTGACCTTGAAGGATAGGCCTCAATCATTG

CATACAAGGTTGCTGCATGCCTTTGCAAAGTACTTGCATTCAAATGTCCCTCATGTGTGCACATGTGTGTTTGGTTTCAG

TCAGGATCAACTTCCCTTGCGTGTGCTTGGTTGCATGTGTGTGGCTTCGGTCGAGGTTGCTGTAATGTTGCGAAGACGTG

CATGAAGAGTGTCGAGTTCAGTGTGTTTTGATACGGAAGTGGGAAGGGCGGTTGCTCACGCTAGCCAGTGGCACTGTGTC

TGTACATAGTTTGTAAATTTGTTCATTGAGTATTTTTTTTAAAAAGCTATCACATTGCTCATGTACATATTTCACAACAT

AGATCATCATATATGTGACCTACCTTTCATTGTAGTTTTTTCTTTCTTTGCTTTGTTCTTTTCTCACGCACAGTTTTGGC

ATCGTGCTGAAAAAGGTACATTGCCATCACAACTACATTTAAAACGTGGTTTTT

>CK190830_bb No definition line found

ATTTACTACTTCACACAGGCCTGAGCCAGGATGAGTGGCGGTGTCTACGGTGGAGATGAGGTCGGAGCCTTGGTCTTTGA

CTTCGGACACTACTCCGTGCGAGCCGGATACGCTGGTGAAGACTCTCCAAAGGCAGAGATTCCTAGTACTGTTGGAGTCA

TAGAAGAACTACTGGCTGATGGACCTGACACAGAAACAAAAGACCTCGGTGCGTCAGCACCGGCTCCTCAGAAGAAGTAC

TTCATTGACACGACTAGCATTCATGTTGCAAGGGCTAACATGGACACTGCCTCGTTTCTCAAAGACGGCATGGTTGAGGA

CTGGGACCTGTTCGAAAAAGTGCTGGACTACACCTACAGCCGACACGTCAAGTCCGAGCCACACCTCCATCCTGTACTCA

TGTCTGAAGCATCGTGGAATGCCCGAACAAAGAGGGAGAAGCTCACTGAAATAATGTTCGAAAAGTACGGCGTGCCGGCA

TTCTTCCTCGTAAAAAACGCCGTCTTAGCAGCGTTTGCGAATGGACGCTCGACCGGCATCGTTGTCGACAGCGGAGCCAG

CCAGACCTCGGCCGTCCCTGTGCACGATGGCTATGTTCTGACACAAGCTGTCGTCAAATCGCCGCTGGCCGGCGACTTTG

TCACCATGCAGTGCAAGCAGTTCCTCGAAGAGCAGGGCGTCGACATCGTCCCAACGTACATGCTGGCCGGCAAGGAGGCC

GTCAAGGAAGGAGAGACGCCCAAGTGGACGAAAAAGAACAATTTGCCTGAAGTGACCAAGTCGTGGCACAACTACATGGT

CAAGGAGGTTATCCAAGATTTCCAGAGCTCCGTTCTTCCAGTTCTAGACTCTCCTTATGACAAAGAGACAGTGGAAAACA

TGCCCACAGTGCAYTACGARTTCCCCMACGGCTACAACCAGGACTTTGGCTCWGAGCGATTCCTCATCCCCGAATCTCTC

TTCGATCCATCAACAATCAAGGCAAGTTGAGCAGCCCTAAAAAACCACTGAGGATTGTTGGCCAGTGGAGATCGGTGACT

CATCATGATAAGGAGCAACAATGGCTTGTGTTT

>CK190840_bb No definition line found

GGGAAGTGCAGCAGCGCAGTCGTGTTTGCGAGCATTTACGAAGCTTACAGGCGTGACTAAGAGCGAGCGCGACGCAAATT

TAACTACCGAGTAACTTCGAACACGATGTAAGCGTCACCAGAACACTACACCACGTACGCTGTGCTAGAAAGACCTCGAG

CGGTACGAAGAAGAGACGAGACTTTGATGACTGGACCCATGCTTCGTGCGGGCGACTCTGTGTAAGCTGCGGAGACTTAA

GGCCGGCGTCGACAGTTCCGATATTGATGAATTTCTCGTTACAAGATGAGTTTGATTGCCCGGGAGCAGTTGAAGTCGGT

TCTCGAGGGCCACGACTTTGTGATGAGTGGCTAGTTGTGGGAGGAGCCGTCGGTGGAGAAGGTCGCCGCCAACACCGTTA

TCATCGTCACTCAACTCCTCCGGCGTGCGCCGAAGCAAACCAGGCACTTGCAAGACGTGTCGTCCTCCACAAGTGTTGAC

AAACTGAAGGCAGGCTCTACATTGACCGTGTTTTTGGAAGATTCGTTGCGCCAGGAACCCGTGAAGGCGGCTTCAAGTGC

ACGCAGCTGGCATCATCAAAACCCCAACGTGCACTTCTGAGACTCTCTGAAGACGTGTATACTCAAAGGAAAATTCTGTG

GAAGGACTTCATCTAGAAGGTGCCACGACACATYRAATGCTGCCGTTCCTTGYTTYYATATAGTGSTATTCCACAATCGC

CACACTCTCGGAAAATTATTTCCACAACCGTCAGGAACCTCACCTTTCTGCATTCGTCATCGGTGTTCTCCAAGCCCCCT

CGCTTCTTTTCGCAGTTTGTACCTAAAGAAACACAGCACAATTACCTGTGCYAARGGCAAGAAAAGCAGTAAAATATCGT

GACCTCGRMATKGACAACGTCACCACTGAGCCTGTTGACATTCAAGACCCTGATGTTCAGGAGCTTTACAGGAAGG

>CK190958_bb No definition line found

CAAAATAGTCCGGAATCGAATGAACCAAAGGGCAATTTTAATTTTGCATATCTTATGGGGCGTGCCACAAACACACAATA

TAACATATGTTCACTTTATAATGTACACGTTAATTTACCAACCGTAAGTTCAGACACACAACCGTAAGTTATTGCACATT

CTGGGTTAACATATTTGTTCGGCAAGATGTGTGTAGGTTGGTGATCGTTGTACAGCATAGTTGAGATTTCCTTATGTGGG

CATTTTTTAGAGCTCTAGGCGAATAATTTGTGCGGCCTGTTTATTGTACTTTCGTTATTTATGTCTCTGTGTTGCATTCT

TTATGTGCGTTTTAGTGCCTTAGGTGTTTTGTTGCCAAGTCTTCTCCTTGTGGCTGTGTAGATTTTATACGTGGTACTTT

TAGACGTCATGACCGCTGCTCCAGTGCCACTGAAAAGTTCTTACTCTGGTTTGAARCAGTAGCACCACCAGATAAGAAAM

AAAAGAACAAAAGGAACACTTTAAATCTTCACGTTCARTGTCGCCGCTAACAATGCATGCCTTCATCTGTGATGGTRATA

CGATTAATGTAGAGTTATGGTTGTGCAACCACATAATTACAGCTTTGCACGCTTAGTCTTGTTGCGAGCCGAGAAGCTGC

CATTTATTATTAGGGTGTTGCAGTGTCAGGGAAGTACACTTACTCATTTTGGAACTGCCGAGCTTTTAGCAGGAGGTTCG

TTAGACCACGTAACACATTCCGTATCGGAAGAAACATGCTTATTGTGACACAGGGAGTCTGCTAAGAAACGCACAGCTCA

AAGGACAAATATGATGACGTCAGCAGTTTCTACGTCTCGCTGGTTTGCTTTTAGCGTAA

>CK190977_bb No definition line found

GCTATAATAAAATACCATTTTATTAACACAAATGTACATTTCCTTCGAACATGAACACGCCGCAACTTAAACTCGTATTT

ACACACAGGAAGAAGATGCACCTTCAGAAACTTTTTAACAATAAATTAAAGTAAATTTTGTACAGAAGCTTATCCAATTT

CAATGTTGGTTTAGCTTTACATCCTTTGAGAAACCATGTCGCAATGTTTTCAAAAAAGGGCAGCCGACATTTTCTTTTCC

CTTTTTCGAGGGAAGCAAAAAAAATTTTTAGCTTCTCTGCAAGGCATGTGCAGTGTGTTAAATATCTTCAACACACTTAA

TCATGATGTGAAATTTCATACTTTTTCTGCCCTTCAGTGGGCAGCAACGATTTAGATAGACCAGAAACACAAATGTCATC

CAATTAGCTTTAGCAATGAAAACAAACTTTGAGGTGCATCCTGACCCTGGTTACGCACCAAAAATGCTTCACTTTAGCAA

AGTAAATAATACATGACAGCTCATCTGTGCAATACAAACTGTGCCGTGTGACAGATCATTTACAAATAACAAAAGTTGCC

CCAGAACCTTTTTGATCCTAGGTACACTTCAAATAAAATTCAAAAAGTTTAAATATTTAAAAGTTCAAATAAAATTAATA

TGCAAAGCATCCTTAAGTAATGGCAATAAATTTCATAGAATAGGGCACAGTGAACAAACAGGTGGCGAGAAACGACTGTA

GGGGAAAGCCCTGAATGCATTAATGGATGCATGGGTAAAGTCTGAATGCATTAAAACAGCAGTGTTATCATGAGTCTAAT

AAATTTAAAAATACACTTCATGACAATTGTGGGTGTAAAGAAAGTTTTCAGGTACAGTTTACGGCAGAAAGTGAAAAATT

TTCTTGTATCTTAAATTTAAAAAATGCACTCCTAGATGTACATATCCATACCCTTCTCCTACTCCCCACAATATTGTCAA

TACATTTAATTTCGAAAATTTTGCGTGGGACCGCAGTTGCTCCA

>CK191107_bb No definition line found

CAGGGTCAAAAATGATAAATGTGAAATACAAAAGTCATATGTAKATTTATTTGGCCACAAAAGAACAAAACAGGAACACT

TCAAGTCAACATTGAGYGGKTTGAGCTCCACAAGTAGCTCAAGAAATCAAATTTTACTAGATAACAAATGCTCAGACATA

TAATTCAATAAAAATAGAGTATTAAAATGTAYACACATTTCGATGTAAAGTTACAAAAGGTTATTCCATTCCTTGTGCTG

CCTATGTGATACCCAGGAGTGAAACTACGATCACTAGTACCTGTGACACCAAACATTAAATACATTATCAACTGCACTGT

CTATCTTCCTAATACACACACACACACACACAAATAGAGGGGGAAAAGGGAGGAGTTCCTGCAACACATTTACGATATAG

CGAATCTAGTCTCAAGGTCAGGTTATGAACACCTCGGTCAAATAGTATTCCTTTGCATGTAGCGCAAAATTTGTTACGTT

GGTACTTTTTGTGCTTACTGTCACTTACCTGTTCTTGGGCAAACATGTTTACTATTATCCTGATTTAATTATATGTCTTG

CAAAACATATAACTGAGAAGTCTCATGACTGATTAGGCTGTAGCGTCGTCTGATGGTAGAGAGAAGAATAACACTGTACC

AGTACTACAGCCTTTATTATTGGCAATTGTTATGAGCCATTCCCAGTAGTTATGCCAATACCAGGGTCCTTCAATACTAT

TACTGGTCATGAAGCTTTGGCGAAAGTTTGACATAGGAACAGGAGCAACCACTCCCGCGCTCTTGTCTGCGCGGGAACAT

CAAGGTGGCCCCGGCGCTACACACACGGTGGGGACTG

>CK191187_bb No definition line found

TTTTGCAAGATAATCGACTTCTTTTATTCATCCACAACAGTCACATAATGTTGAACACAACTGGCACTGTGTTGCTTGCA

CCTGGCGAATGAGGAGTACCTCAATGCTACAATAACAGCCTGGAACCTAAGGCTGTGTGAAGATATTAACACAATGTATA

CCCTGCACAATTGTTGACGCGAGTCACCTACTCTCATAGTTGTCTTTAGAAGTGCATATTTGAATAAAATTTAGGTTCAG

TGAACTTGGAACTTTTGTTTTTTTTAATGTAAAGTTTGTTTTAGTTGTGAAAAAAATTGGCAACTAAAAGTGGATTGTGT

GTATACAATAGGTATGCCTCTACATATTCCCGCTGAAATGTTATTATATTCTTGTATATACTTATCAAAGCTCTAGTACA

TGCGATGTAATGAATGCGTAGCGTAACATGTGTATAAAATTAAAATATCATTTTTTCCATTTCTGAGGTTTGACTCTAAA

ATTTTGGGTCAGTTCAAGACGCCATTGGTGCTTATTCTGTGGCTCATTTGAGCCTCGCCAAATTAGTTCTGTACTTGAAC

AATAAAGAATTTAAACCAACAGTGGTCCCAGAGTGGCTTCCTGCACATTTTCGGGAGGTCAGGTACAGTTGCACTCTGCT

TACTCAACAAGATATTTCAGCAACATTACGTTCACCAGTGAAAGTACTCAGCAATGTTGTGCTATGCCTGTCCCGTTGTT

TTCTGTCACGCAAGTGATTCTAATGCACATAAGTTTSATTTATTTACTYAAATTTGAATATACGATYAATTGTCGTAAGC

ATATGTTGGATAGAATAACTCTACAGACACTTCATTGTAATGCATGGCACACACACCCTTCCACACCGATTTAAAGACCT

AAGACACTTGGTTTCTGATGAAGTCACTGCAGTGTTAATGTCTAA

>CK191249_bb No definition line found

CTTGTTCGTAAAAAATAGGGACATTTATTGCTCAAGATGCAAACTCTAGCACAGGAAAAACTTCAAAACAAATGCATAGA

AAGGGGAAAATGACTATGTATAARATCAAGTCAAACAGCACATTTTATTTCCCCTAATTCARAACATCGTTCAAATCTTT

AATATTTTGTGTTAGCGCGAAACATCSCCGTACACAGAGCATCCAAAGTAAACGACATGGCCGTTGACGAAATCCATACA

AGTTAGTCTACAGAGCTTTTCGCTTCATAAACAGCAAAGGCAGCTTCCACTACAAAGCTATACAAGCGAGCATCTACACA

TCCGTGAATAACAAAAACATTTGCACACGCAATGAAAATGAACAAAGGTAATAACATGTGAGGGCAGTTATGTCACTGTT

CAAAAATTTGACAGGTGTAGCAGGTCCCCTGAACGATACTTAGGGAACACGTTTATTGCATGCAGTGAATTCAAAAGCTG

CCTGATTGACTGGGGGTCAGTTGTAAATAGTAAGATATCAAAACAGAGCCGTATTTTGCTACAACTCTTCCGCAAAGCTC

TTCCTTACGCGCCTGTGTGTTGAAAACTGTATTGCTAATACAATTCCTCGTGTTATATGCGCGGCAATTAAAACAGGTCG

ATTCTCAAATATTTTCAAATAGAAATAGTTTGCTTGAGAAGCCTACACAGTGGCGTCCAAGCTGCTGCCAAACGATGTTA

GCAAGAAAGTACCTCAGACACCACATTCAATTTCATTAACGTGTGAAAGTCATCCGACCGTTAAAAGTTATTAGTGAACC

TGTTAATTCCTTCATAACAG

>CK191293_bb No definition line found

CTCACATCCTGTCATATATTCAAAATGGCTTAATAGCTTTCGTAAATATGCTTCAGGAGCAGCTAAGTGACATTTGTACG

TGTTGTACGCTGATTGTGGACTACACGGAATGCATGATAGGARTTTTCTAGTGCTTCGRTTGTGTGGAACGTGATAAAGC

GACGCTTACGTTCACTGTACTGTAGAAACACGTRTAGATAGGTTTCAGAACTGTTATCACGGAAATRCCTCTGCTATCTC

AAAAAAAGCGYCTAAAGCTGTTCGCCAGGGGTACATCGACAATTTAATGTTGCCAWYAAAAATAMTCCGATAATGACAAA

TCTGATATACGAGATTATTGACAACTCGACTTCGTAACGTTTATTCTGCTCTATTAAACAAAATTCATCATGCCTAAAGT

AATTTTCTCTGATAAACAACCAAACGGTTTCATCGTAAACGATGACCTATAGTCTAAAAATATTAAGTAACTGACATGGA

CGATAGCGACAACAGTTCTGATAACATGCGGGCGCAGCAATATGGAGGTGCTACAACAGTATGTTAACAGCCAAGTTCCC

TTTATACGGTGTTACGCTGCTTTACTTACAGCCTATAACTACTTAACATTGTACGTGTAGAGCAGTATTTACTGAAATGT

GCGTATGCCATTTCGATAATAATTCCATGCTACGTTTTCTTAAATTTACGGAACGTTATGGAAAATATACGGATTGAATC

GTAACTTTCATACAGAAGTACAATGAAAATAAGTGGACTCTATATGGATGTCACACGAATGGAATGGAATGAAAAAACTT

TATTTCAGTCCTACAGAACGTGCTTAGCGCGTAGCGGGCGTCTCCCACGTAGGGACCG

>CK191332_bb No definition line found

GGACTCTCTCTCTTACCTCTAGTCTCTTGATGTGTGCATGTGATTTTCTGCACTCTAATAAGGACAGTACAACAGAGCCC

TGTCACAGTGAATTTTCATGTTCCTAAACTTGATATTTGCCATGCTCAGTTCTATGGTGCTCTTTTGTTGACCACAGAGT

CACAGGTCCTGGTACCTGACTGTCGTTGCAGTGCTGTGATTGAGGTCCGAAGTGGAAAATAGGTGCTTGCTTATATATTT

CAGATATTGATAGTTTGTTGGCRTTTATATGGATTGACAAATACTGTTGGTTCACAGGAAGGGAGCSAGATACAAAGTTT

AACAATAAGTCTTTAAAGTTCCAATKTCTGACACACTTCCACATTTCTGGTTTCTATGATGCACATAAAAAACAGAGACA

AGGGTGCTTGTGTACTATGGCCAATACTGAAGTCGATGATCTTTGTAAAAACAAGGCCGCTTGTCTAGACTACTTTTTAC

AAAACCAGATGGCAGGAGGTATGGCTTGCTTATGTTGCGCATGCACAGTTATTTGTCTCTCAGACGTGTATTTCAAAGGG

GTCAATCAGAGTGGTGGGCAGTTAAAGGGGAGTGCAAATCCTACTGTAGAGAATGTGCGCACGCATGCACACACACACAC

ACACACACACACACACACACACACACACACACACACACACACACACACACACACACACACACACACGTGTGGTGTGCGCT

TCAGCTTCATTTTTCATAACAAATAGTATTTACGGCACCACAATTAGATTATAAAAAAATTTGTGTAGCTTTTAGAGTGT

AACCTTGTCGGGTATAACGTAGAAGCAAATAATTCCAAACAAGAGAAGCCTTTCTTGCGCCTCTGG

>CK191354_bb No definition line found

TATACAGACTGGTCTGCAATTCAAATTAAAGGGGCTCGCCTTGAGGCAATGATATTACACATGAAGATTAGCACATTCAA

GAGGGGATAATTGTTATGCCTAAACTAATAAAATGAAAGTTGCAGTCTCATCATAAAAAAAAAATTCTATGGTAGTCCTA

AGAATGGAAAACTTAGTAAGTTCAATTCCGGGCCATTGAAATTAACCGTTTCCGTAAAGGTGCATGTTGTATGAGTGTTA

TGCGCAGCATCGACGTAATATTAACTGCAAACCGAAATTGTGTCAGTGCCTAAGCAGATGTACCATTCTGCCTACCTTGT

TGCGAAGCTCTCGCAGCCAAACGTTCAAGTGTTTTCCTATGCAACTCTGTCCGCGTAGAATCCTGATATTTCGTACATAC

CTTGTCTGCGCGTTCTACGCACTTCTTCCCGCTCTTTTAGGCAGCTGTGCGTAGTCGTTCGGCTGTTCTGACTTACTCTC

TCACACATGAAGCATAGGACCTCACACATAGATGATACAGGAGGCTTCTAGAGACCTCCAAGTCAGTCCCAAAGGTTGAC

TAAATATGTGGGGTGTAATGAACATTCCGCTCACCCCCGAAGAGGAGCTCTGCGCACACTATGACATAGACTACTAGTTG

TAGTCTTCGTAAAGAGTCTTTCTTCAAAAGGGAGCCTCCGATTTTTTTCCGTTCAACAAGAATGAGATGTCATGATCTTG

CATCATAGGTGAGGTTCAAAACTGACTGCTCCAATCCTCAGATCGCCTAGGCTCAGTTTTACAATCGGGCGCATTGATGA

CTTGTATTCAAGAGTCTGCGATGAATACATTTGTATATCCCTGATAACTTGCAAGCTGAAGTCTACCAACATGAGCCAGA

ACCTTATCATGCATTATGTGTGCGGAGAAAGGCTTCTGCAGGGCATACTTTAAGCATGTCGTCTGCTTGTCACTCTGCAC

TTGATGACCTCCTATTGGCTAAACAAAAAGGCGACATGGGGCTCATACAACAGACATGAATATTGTAGCTTGGTACTCCA

CTTCAAAAGATACACTTCATTGTTTAACAACCTTAACTTTGTTTTTYMATGAGATACGTATCTTTTGTSAAAACATTGGA

G

>CK191471_bb No definition line found

AGACCATGGCATATGGTGAACAAGACACTTCCTATGCCACAACAAGGAGGTGACACGCGGGCACTGGAGCTGTCGCGCCT

TTCTCCAAAACCTGGTGAATACAGCAAACTAATTGCCACCTACTTAAAGCTGAAGAACCCCGAGAAGAGTGTGTCACCAA

AGAAATATGAACAGGCAAGTCCCAAGCAAACGTACGACGACGTTTACACGGAAGATATGGCGGCTGCAACATCACTCAAG

AAAATGGGCAGCCATGGTAGCCATGTTCGAATAGATGAATTCAAGGCTGYAGAAACGAAGTTTAGGCATTTATACAGGTG

AAGGCGTGGATCSCAGAGATGCAATACATGTAGGCAGTGWGAATGTTTCASACACACCATCAACAGAACTACCAGGACAC

AGGTGGCGCTGTGACACCACAAGGCACTACTGAAGACGTGGTAACATTTGACATTCTTGAAGCTCACAAGTATAGAAAAG

ATGACACATTGTCACACCGAAAC

>CK191521_bb No definition line found

GTCCCATCGAGTTTGAATTACGGAGAGTCGCAACTATATTGAAATCATCTTCTCGCATGAAGTTCAGCAAAGAATTTAGG

CCTTTGTCTAGACTGCTGTTAACTCTTGATTGAAAAAAATACAAATTTTTACTATCAGTGTAGCGATCTGTATAATAATC

TTACTTTTGTGTAATGCCCCTCTTTTGTGTTTTTTTGTGGCTATGTCGCAAACAACTTCATGCGTAAGTGTTGATGTGCA

CTTCGTGTATGTTGTCTCTGCGCTAAAGGAAGCTAAAACATGACGTAGACACACTTTGCAAATTAGTACACTGTTTATGG

CAGTGACATACCCATAGGGAGCTGCAATTATACTACAAGCTTTGTGCTTAGCACACAAGGTAGATAGAAATGTCAAATAG

AGCAAGAATGATTGCTTCTTTAGTGTGTCCTGTACAGTGGGACCGAAATAGTATAGCAATTTCATTTTTTTCCCCTTTGG

TGCACTTTTTCAGAAGCCCAAGGAGCACTCTGCCTACGTCAGTGATGAGAAAGAAATGTGTGCAATGGAAGGAATAGCTA

TTTTATACCCGCTTTATATCTGGGCACATTTGCTTTTCATAAATAACTCTTGGCATTGGCACCTTGTATAAAGGTGGTGC

ATGTTTGATCACGATTCTGCCAGCAATTTGTAGCACATTATMAGCTTTTACTTATTGTTTGCCTTCACAATTTCGTGCTA

TAAATATTCCTTTAATTCATAGGGTTGGGCCTGGCAAAACAACTCTGATTTGGACATGGTCATATGAAACTGATCTTTAT

TCATTAATTTTTTGGGTTCTTGTCAGCTACAAAGCATGGCAATTATGAAATAAACATACATGCTCATAAAA

>CK191548_bb No definition line found

AATAATCAGTAAGTGATACAAAAACAGCACGGCCACTTTTATCGTAAGTTACAGAACACAGGATCATGCACAAACCAGGC

GACAAGACACCTTGATTATTTTTGTGCACACTTACTAAAAACAAAACAAGTTATGGTGAGCCGCTTTGGAATGTCTTGTT

CATTGCACTATGCATTTATGATCACGTAATACTCTGTGCAGCTAACTTAACTGTTAGCATAGTAGGACCTCACTTACACA

GTCTTTGACAATGTGTCTTCATGGTTACAATGATGTCGAGTCCCAAATCCCACACTGCTAATGTATAACACTTCCCATAT

ATTAATTTCACTGCATGTGGTCCCAATAAACATCTTTGAAAAGCCTCATTGTAAATGCATCTAATATATGTAGATACACA

CTAGCACGAATTAGGTAGACAATAAAATTTACTGTACAGCACCAGTAACAATGCTTTATGCCACATTACAATTTAAATCA

TTGAAATCACATACATTATCAGAGCATCGTGTAACAAAAAAAACGAGCGTGCACAGCACCACAGCTAACAAGAACATGAG

TAAGCAGCAAGTGAATATCCTGTTTTCTCGCATATAACACACATCCACAAATGGCAGAAATTTTTAAAAACATATTACCC

TCACATTTTAGCAGTGCAAGTTTACACCCCAACAGAAGTGATCCCTTAACAATAGTAGAAGATTGCAAAAGCTTTATCGT

TATTCTGTTCCAGTCATCAATTTCAAGGCCCT

>CK191592_bb No definition line found

TTAATTGAAACGTAACACCTAGCCTTTCACGGTTACCGATGCAGTCTAGTGGCCAATATTGTTTTCATTCAATGTTTTCT

TTTGATGTGACATGAGCAACCACCATTTTGCCATGTTAGAAAACTTTGTAATTCGCATCAGGGAGGCAGAAGCGACCGAG

GCTGCTCATGAGGCATTGAGCAAAGGGTTGTGTGCCTCGGCACTCGCAGCAGTTTGTAGCATGCTGTGTTAGTCTTAAAC

TGGGGCTGGTTGGTTCATATAGATTTTGAATGGAAAAACTGCTCTGGAACAGGACAGAGACGAGATGACACAAACACGGT

GCTAACAACCATAGTTTATTGCATTTGCGCATCAGTATATTGAGGAACAAGATATCCCTGTAACAATATCAACTCACACA

TTTGAAATATACATAATTTTATGAAGTGATAGTAAAGCCACAGTGACACAACATTTTGCACTTTTCTGCATGCAAAAAGC

ACTCTATTGTGCCTTAAAGAAAATTTCACTTCTTTGTGAAACTTTTCGGGCTTTTTTTCTTCCACAAAGGATTGTGGCAA

AGGCCTTTATCAAGCTTTTTGTTCTTGTCCTCATGTTCATTATCTTGCTGTAATAAAATCGAATTATTGAAAACCAGAGG

ATGTTGAGTAAGCTTCACTGTTGCTTTATAAAAATGAAATTATGTATGTTTTGAATGTGTGAGATCGCATCTTTGTTCCT

CGATATGTGTGCTGATCTAGTTTTTGCCTGTATGTAATGACGAGCGAACACAGTAAAATGTGGCTCTTAGTCGGCACATC

TCTTCTTCGTCCAGCACTGTTTCTTCATTCAAGATCTCTATTGTGCTGTCAAGGATGACTCTTTGGTATTGTGAAATTAT

TATTTCATCCTGGAACTGTGTGGTTGCCATCCATGATCGTAGCTACACTGGATTAACGTGCAGTGGCAAGCAGTGGAGTT

ACGTCACG

>CK191660_bb No definition line found

ATCTCCTGTTCCCGATTTTTTTCCTACACTTCATTCCACAATGACTTTGCCCACCACTCAATCTTTTCAAGAACCTTGCA

TAATGGGCTTGTGTTAAGTAAAGCGAACCTTGTCTGACAAAAGCAACATAGTCTCACAAACAGTTATTGCCGTGGCGATC

AGCTCAACACTGCACCAACAACCTACATGGGAGGCGCTCAAACGACGAGGACTAAGGAACCGTTGCTTCCGTAACAGAAA

ACTTCCAAAATGCTGGCCAATGTAAATGTCACATGTAAAAATATAGAAAGAGCTCACAAATGTTGACACATGGTATAAAA

GCAATCATGCAAAGATGCGGTYAGCATGAAGACTTTTCAGTGGAAGGACAGGGCTGCAATGACACTGTGAAACAATGTGT

TTACAATATGCACAGTTCAGCAAAATGGAGCAAAAAGGGTCCATTTAACAACTTGCCTCGAAGAGGGAGAAAAAAGAAAC

TAAAGGAGAGGAATGGGTTTGTTGTTGACTCTTTCACAAAGGGTGAAGAGCAAAGGTAAAACTGCTTAACTAATAGCGAA

GTTCACAGAAAATTAATAAGAAAAAAAAAAAGAAGCACTCAGGCTTCGAGGTATCTCTAAAGTACAAGGACAAAGTTAGC

CATAGCAACATCGTACACACTTTAAAGATGCCTGAAAACAAAGTGACATCCTTAATCCGCCACGTGTCGGACTAAACAAC

TCTGAGCACCGCTGCAGTTTCTAACGAAAATGTGTGCGCTGTTAAAGCGCTAAACAACACAGGAACGTGCACATCGAAGA

CGGGTTATGCAGAGACAGGAAGACGGC

>CK191770_bb No definition line found

ATGCAAGCTATGCATTCTCGATAACTGGCCTGAACTGGCACTAGGCACACTCACAAAGTGTTTACATAGTTTGTAATGCA

TTGTTACCTGACCTCACAAGAGAATCAAACAGAGAAGACAACTTTTTTTTATGCATTTATTCAGTACCACTTCATCACAA

TACCATTTCTCCGTTTACATAGAAACAGCATTCAAGTCCATAGTTCAACRYCGCTGCCTATTCACATAAATTTTGCACAC

AACCACTATGGTAAAAAAGCGCAAAAACTTGCTAGAGGCAGAAAGAAAGCAAATATAAGATCAAGTACGGACACATGGAG

ACAAATACACAACAATGTGGCTTTAAGATGCAAACACATTTAAAACATTTGCATTAAAAGAAGACACACAAACTCGACAA

TCGCACACAATCACATTAAACAAGTTGCAACTCGCAGCACCATACCACAGTGAAAAGGAAGGCAGTGCTATGAGGTGTCA

ACAAAGACGGGAGGCACATTTGCATGTTCACAGACCAAATTATGGAACTATTCAAATGATTACCACATTCCCTATGCCAA

CAAAAAAATGTATCAACTATAGGTGAGCATGTGTGCTTCTCCCAAGCACCCGCTTTCCATTGCAATATGCATTAGTTAAA

TGCTTTCGACAGATATACAACACAGCTCTAGACACAAGTGCCAGCTCATCGCTGCCATGAATTCAACACACTGCACATTG

TCAATGCCAAGAAAAGTGTCACGGAAACCACTCATGCTAGACTGCAACCATAACCTGCTTGTCACAGCACTGCCCTGTAA

ACCACAAGGCCAACTTCAAGTTCCTCTCCGCACACTAC

>CK191874_bb No definition line found

TCAGAATGATGAAACATTGCTCACTGCACTTACCACGTTTTTTTTTCCYTGCCTACACATGTCTCAAGTTGTTGTGTGTT

GCTGTGGCTGCTTGGTGTAACGCACCGACAACAGCTGTAATGCGTTCAGAGCTTTTACWTGCTTATTTTGGTGAAGAAGC

WARCTGGTCAGGAGGTCATTAGCTTATATGTAGTGTTCTTGAGGACATGCATGGCTGGATGTTTTTGAATAAAAAAAAAG

TTGCCTTCTGCACCACTTTACCAGTAAAAACAGAGCCAAAAGAGCATACTGATGATGTGAAATTGAAATTCTGTACTCAA

CTAGGTGAGACTAAAGAAAGAAAAGGCTTCATCAACATTTTTTCACTGTACCAACAGGCACACAGCATATTTATCATTGG

GATATTAGGAGTGACTTATTCGTACGGAATAAAAGAGAAAGTAGCTGTTGATGTGCTTTATTAGTTCTGGCTGTTCTTCC

TTCACACTTTCCCTGTTGAGATATTTGTGCAGAATTGCGTGGAGACGTTTTGCAAAAAGAAAAAAAAATTATAGGCACCT

CTGCCATGGCTTGTGTGCTCTAACGGTGACATTAGGTTCAGCCTGTCTGTACAAATGATACATTTAACACTAAAAGAAAC

TTTGATTGGACTTGTCTACATTATCTGCTGCATACCACTGTTTTTTTTTTTTTTTTGAAAAATAATGTTTCGCCAAAATG

TTTTGGAAAAAATGGGCATGACTAGCGAGTAAGTCAGTATGATGTGTTGCTGCAGTGTAGTCTAGAAGAGATTGAAGAAT

AATAGCATGTGTTTTTTTTTTTATCTTG

>CK191978_bb No definition line found

TTTAGATCATTTTTTTTTTTTCTAACTTCAAATGTCGGTGGAGAGTTGCACATTCATTATCTTGCATTTCATTCAATGAT

GGCRWTGTGTAATATCACATTAGTATGTGTGCTGTCGTGGTGATATGTTAGATGTATCAAAGTTGCCTACTCAACATAAG

ACTGAGTACGTCACGTAAGTAGTCACCTGTARAACAAGTCTCAACGCTTGTTGCATAACTACTTTTTTGTATATTGCACT

CCACAGTCACCTGTTTTTGCTCTTTAACGGCAATAAGTGAAAGTAAGCATTGTGTAGCTGCAAAATGTGTAAAAGCTCTA

AAACCTGATCTCTTTCCCTTTGTATCCTAAGATGTTAATTAAATGGTGTTTTTCCCCTTATTTTTTTAGGATGGTTTTGT

GTCATAGGTGTTAGTGTTATTTATATGTAAACTTCCTTGATTTCTTTTTTGCTGTGACTGTGATTAAGATTGAAAACAGA

GAGAGTTGGTGATTTATTTTGGGATTTGTGCACATGCTTCTTTTGTTGTTTCTTGTTTTGTGATTACTTCACAAATCACA

AAATATGGTTTGAGTATTTGTTAATTTGGATGGTGGTGGTTGATTGATGAGCAAAATGATGGTTTATAACTCAGCACAGT

GCACAGAACATTTAACATTTTAATAATATTGTAATATATCCTGGAACATCGCATAAATGGTAAATTAGCAGTGAGAAGCA

CTTGCTCATAATTGTGTGGTATTACTGCTATGGACATTCTCAGCTTATCCAAAGCAGTAGCATTTACCAGAATCATAGTC

CTGATCTGTGCTTGTAAAAAGTGCTTGCTGTAACTTTTGTTCAGTACTAAAAACATACTGGAGGCATTGCAATGCCTTTC

AAATAATCTTTTCCTTCAGAAATTTTTTGTAAAGGCTTATTGTAGTAATTTAATATAAAGAGTGTAGTGCTTACTTTCTC

CATATTGAAA

>CK192035_bb No definition line found

CTTCAGCACACATGCTTTTCAGACATTTTAAAACGCACTTTGAGGAATTTAATTGAACTTTTTTTCGGCAATTCATGAGT

GAACTTGAGGCCCTTTGAACCGGTGCCAAGCACATTTAAAATGCCCTGGACATGTTTATCTCGCAYGTGAGGTTGCWCCY

CCCTTAGCACAACCAAATAATCATCAACATACTATCTAAAAATCTTCTCTCCAAGAGCCTGATCAATGTGTCCTGGAATG

CGTTTGTCACAGTCAGCTAAAAACATTGTGCTTAAAATTCGAGCTATGCTTGAATCAATACATACACCCTCTCTCCGTAT

AAAAAAACTGGTCATTGAAAAATACAAGGGTATTAGCACACTAAAAAAGATGGCGATTTTCGCAAAACTTCTGGTCTATA

ACCTGGAGGAGCTGTGAAAACTTAAAGGGTCCGACTCCTAGATGGGGACCCTTAAAACTCTGAGAATGTCATGCTAAGGA

ATAGAAAAAAGATCCTCAGCATCAATGGCAAATGCAAAACTGACTTCTGAAGGGCTTCCATTTATTGAAAAAAAATTTTT

TCCTTGCAGTAAAGCATGTTGGTGTCCATCGCCCGTCCTGTCTGTGTGGCCTCTCTTGTGTCTGCATCCTTATTTTTTGT

CCTACTGAAATGAATGCCAATGAGCATTTTAAGTTATAACGCTCATGAACTTGGAAGCATTTGATAGCCGTAGGCCTTAA

TATTAGAGTGCCAGTGCAGTTGACTGAATTTGATGATTTTAATCAATGAGCAGCTGTGCTACAAACCAGTAAACGTGCTG

GCGATCACATTAACATCCGTCCATTTTTTGTACATAAATATATCTTTTAATCAGTCTTTGCAATCATGTATATATTATGC

ACTTTTACTCAAGAAATGCGTTTGCTGCTCTTCAGATATTCAGCTTCAATTTTATGACTGCCTCTTGTGTAAACTCTCGA

TGTATTTTAAAATGTCTAAAGAGTGGATGTTTCAGAGCGGCACTGAAGCTGTAAGTTCTGTTATATTTTATCTGAATACT

GTGTTATGT

>CK192052_bb No definition line found

GGAGGAACTTGGGAGAAGTGTCTTAGTTATTGAATTGATGCAGTTGCAATGATGAAATATGCACCATGCAATGAGAAAAG

GTTCCTGGTAGATGCTGTCATGCAGTGGTTATGGCTGGCTCCTGCATTTTTAGAGGGTACATAGTAGGGCAAACTTGGAA

AAGACTGATCAGAAAGGTCGGGTGCAAAACTTCAACTGTTTTTTCGAACGGGCAGGTCGCCGGATTGCAGTTCTCTGTGC

ATGAGCGCATCGGGACCGGTGGGCCCTGGTCACGCTCGCATGTCGGGGGGCTCTGGCTCATTTTGGTAAAGTAAAACGGA

CGTATCACTAATGCAACCTTTGTTATGTCGGTTTCACTTTACCCAGGTGAGCTGCAGCTTCTTGGCATGCGAGTGTGACC

GGGGTTTGCTGGTCTTGATGTGCTCATGTGCAGAGGGTTGTGGTCTGGTGGTTTGTTTGTTTGAAGAAACAGTTGAAGTT

CAATGCCCAATCTTTCTGCCCCCATCTATTTCCTTCTTTTGTCTGATCAGTCTTTTCTAAGTTTGTGCTATTGTGTACTT

TCCAGCAACAAGTCTTGCTCATCCTTTTCTCTTGTCCTCAATCATAAAGTTTTAAACTCCAAGTTCATTCTATGCTTGTT

ATAATAAATCTGAGTGAGTTAATACATATTATGCTGTAGTAGAGCAATGACCTCTTCGCTCATTTATTTGACCTAGGTAA

AGAAATGTACTTTGTGTAGGCTTACTACTAATTACAGGCATTTAGCTCTAACAGAAGAATTTTCATTTTAAATATTTGCA

TCAAAACTTCATTTGGACAAGAAACGGTGGTGCCTTGAAACATAATTACATTAAGAAGCTCARATTAAACCAGGCTATAG

AAATTGATGTGCTTGGCTTCGGATTTTTTTCCTTATTGTCTTGTGCTTCCACATTCAGCTGAGCACCTTGTTGTCGGCAG

ATTTAATGACTCTTTGGCAGCGCGGCACTACACAACAAAAAAAGCGACCTTTCGAATTTCTGATAACAGCTATGGATGAA

TCGGTACGTAATATATACACG

>CK192108_bb No definition line found

CAAAATCTTACTGTTTATTGACAAAACATGCAGTGTTGGTACCATAAAATCATGCCAAAGAAAAAAGTGTCACAGTTCCC

ACAAACATTTAAAAATGTAAAAAATAATGATAGAACAAATGCAGAACTTGGGTCCAGATCAACCAACTTGCTGAGATTCA

CAAATCACGACATCACCCAGAGACAGTACAATCTCCATAATTTGTGGAATGCACTAGCCCTCTAACAGTAGCTGTTGATT

ATACATACAACCAACATCACTTCATTGAGACCTTTCAAATATCCTCTCATACATTTTGTCAATACCATGCAGTTGCCACT

GGATTCAGAAAGCGAAGTTGACCGATATGACATTAAATTTTCTAGTCGTTATGAATAACATTAAAGCATTATATATATGT

ATATTTGCCTAACTAGCCATCAGATATTTACAAGCTGCATACAGCCAACCTGCACAATAAAAGATAACCAATCCATATGG

TGAGTAAAAGAGTCATACATATATGAATGTGTTGCCTTTGTGATTAAATCAGCACACTGCACATTCACAGCACTTGAACC

CTTGATGAGCTTAATAAAACTGTGTACATGTGCTAGAGATTGCCTGCAACACTGCTAGGCCTTGACAAAACTACTTGCAA

ACGCTAAAAAAACAATTTTTTTTTTAATCACATTATGAATGTGTTGCCTTTTTGATTAAATCAGCACACTGCACATTCAC

AGCACTTGAACCCTTGATGAGCTTAATAAAGCTGTGTACATGTGCTAGAGATTGCCTGCAACACTGCTAGGCCTTGACAA

AACTACTTGCAAACGCTAAAAAAACAATTTTTTTTTAATCACAATGCTACACAACAGGGCTTTGTTGTCACCAATGTGCG

ACAGAACTAAGACAGCACTATGCTCAACTGCATGCAC

>CK192124_bb No definition line found

TGGAGATGCAAAAAGGTTTTATTGGCTATCTCAACAGCAACAGGTACAAAGTCCAGTCAAAAATTGCTGGAAAAAGACAA

CAAAAAAAAATAAAACATGTTGGGGGGGGGAGCAAACAAACAAAACTCAACAAACAGAGACCCGCGTGCGGGGTGCGCCT

GCATTTGCCATGCCTCACTACGGCCCCGTCGGCCTTTTATCTTCCTCGCTCTCATTCCAAGGATGCTTGACTAGAACCCT

CAACAAAAACTCCACCAGTGGACATACGGGGCCGTTCAAGCAGCCAGCCGCCCGCAAAAACAAGCGCACGGCTGTCACGT

GGCCCTCAACTCTTTTGATTGCTTCCAGCTGCAACGGTGCCCGCATGTGGTGAGCTCGCAAAAACAAACAAGATCATCTT

ACCTAACAGCCAGAGAACTTTCCAAGATTTTGTCTGACTGTGACGAATCAACCAACTAATAAAAGCATAATTTTTCAACA

AAAAACATTTCACTATACAAAACTAACATTTTCTTTATTGCATAGCCCGACTTGCCCTTGAAAACGAGTCTGTCTGTCTC

TGTGGCGAGTCCATGCATCTTACGAAAACCAAGAGACCAGCGTTTGGTGCACTCAGGGTATTTTACAGGCACTCTTTAAC

ATCTGTCAAGCAAAGAGTGCTTTATGATCGCAGTTAGAACAAAGATAAAGAGYAAGAGTGGCAAGTCCACTAATTGTTGT

GCAAAATATCCTCGAGAATTCAGTGAGGTCAACCGTCACCATCTAGGTCTATTCAAGGGGTTCCACAGCTTGCCAAAATG

AGTTAATAGGTAAGCAAACCTTACCAAAG

>CK192166_bb No definition line found

CAAGGAGTCTTGAGGGTGCCTCCCAGATCTTTATGGGGCAGCTGTATTTTTCTGGCTCGCCTTTATGGCAGAGCTTGCGA

ATTTACGTAAACTTCTAGCAGATCTAAGGTATTGGAGCGATATTTGCTCAGGTTGTCTTGGTGAAACAACTTGGACTCTG

CTTAACTGTCTCATAACTATTTAGTAGATGTTCAGGTGTTGAGTCGACCGTCGACCCTTCTTGGGCTGTCGCGCTGGCAA

TGTTGTAAAAGTGACTGTCCTGTGTCGGTAACTGCGAGGAAACCAAAAAGGTAGAACAACTTTTCCAGAGTGGATGCCGT

GTGTGGTCATTTTTACGAAGTRMTRWAAAAAAAAAACTAGCCTTGTGATAACTGGTGTTTCCCAACAAACAGTGGTGTTT

GTCCCTTYGTGTGTAAACTGCTSTAGCTGTTGTATCTATCCCTCAATGAACATTTGCTTATCCCTTCGTTTTCCATTTGA

ATTTCGTTCTGTACACAGCAATGCCCTGTATCTGAAGGTCTGTAAATGTCTCGTGCATCTTTCTCCTCCCTTCACCCGTG

TGTATTCCTGCCCTGTGAAGTCATATAGTCATTTTTTTTTTTTGCTCACTGTCCTCGCGACTTGGCACTTGTGTATTACT

TTTGTGAGTTATCAATAAACCGATGAGTTCG

>CK192167_bb No definition line found

ACATCATCAATTTGGGGAGGTTGTTCCGTTTCCCCTTTAGGGTKGTCCATGTTTGACACGCTGTCATCAATTAAGAGTCT

GGSGGAGCTTTTAGGCGTTGACTCAGAAACTCCAGGAACACGCTCTGCCGCATTTGTAGTAGCTAAGGTTTCGGATCCCG

CAGCCACTGCATCGCTTGGCTGTAAAGCTGTATTCTCCCCCTAGAGTACTTGATCCGATGGCACTTGAATCATCTTGCTG

ATCCCGC

>CK192215_bb No definition line found

TGACTCATTTGACTTGGAGAAGCAACTCACTCCAATAACATGCTACAACCGATATTACACGGAGACTAAAAGCTTCGTGT

GTGATTGATTTGACTTTATTCAGTTCTTTAATTTATCTTTTTTTAAGATTGACGGCAAACAATACGAGAAAGACTTGTAC

CAAGATGTCGATACGCAAGCATCATTACTTCTTCGACCTCAAGCTGCTGGTAATTACCATATAGTTGGCCAGATCAATTC

CACGCACTTAATACAGCCCATCGGGCGCATGGAGCGATCCTCTGTCGCCGGAATCGCTCACAGGATAACAGCCATCCCCA

AGTACAAGGGAATC

>CK192312_bb No definition line found

AGAAACTTATTTATTCACGTCGCACTTTTATTGGACTGGAAACTTGACAACACACGCACAATCGTAAATAAATCACACAC

ACTTTCTATACAGCATCACAATTGCACAGATAGTTGTCTTGGAGCTTATTCTTTTTCTCTGAAACTAGTTATGTTCTGCA

GGACAGTGCACAGGTGCGGTTTGGCTTTTTTTTTTTCCTCGTTTGGAGAACTTGGTCGACAAATGTCCTARAAATGGAAT

ATTTTGTCAGCATTACATTAGTACTATGATTGTACTATATCGATTATCTTGGTCTGAATTGATTCTTGTGTTTTARAGGY

AATGGCAGCCAGAGCTGTGATCTCGTATAGAGCAGTTAAAATATATGTGTATATGCTAGTACACACTACTTTGTTGCAGT

AATTACATACTTTCCAGCAATACCTACACTTCTCTGCGGCAGGGTAAGTGTGACAGCTAGCTACTGCGCACCTTAGCGAA

ATGCTCTCATAGCTACTCGTGTGACTCCTGCAGGATTTCACTCTGCTATTCCTTGAATGGGAGGGATTAGTGGAASACRA

CACGTRAGCTCTTCTGGCTTAACTCGAACTTATMCACTRATGASAGATGGTACRAATTTCTTAGTGACTCTCRCATTAAT

CCCRTACGAGGGCTGCTCTRTTTTCCACCATTGGTAGTGATCCAACCAAATGTYKGAGTACGTGTACCTTGATATACTAA

ATGAGCTYGATGRTGTCAGGCTCACTCGTAATGCCTAAAACACAA

>CK192373_bb No definition line found

ACTCCWTGCCCTGCAATCAAAATTTTAATKCCGCARTAAAACACAGGCAATGARATACTGGAAGCTCCCAATTCAYGCTG

TTCGTGAACCACCACCCCAAACATGGCACATGATAGTCTCTGCAACAATAGTTTTTGGGTTCATGAAACTGCTGTGGAAG

CACAGGCATAAACAAAACGCAAAACTCGAGACAGTGAGACTACGTGCAAGTCTCCTGTCGTCTCAACTCATCTGCTCGCT

TGCACCGAAAGAATTCATGTGCAAGTGCAATTAGAACTGCACATGACTTTTTTCTTAACAGCCACATTCTCAAGAACAGA

TGATGCAGTTATCAAAGTGAACTGATTGCAGCACTAACTTTAGGGTTTCCCAAATTGTCAGACAAACCACCTCCACATTT

TTCATTTTAGATCTACAGAATTTCAAGAATGAAGTTTCAGATCACCTGCACTATTGAAACTGATGCTTCAGCTTAATTCT

ATTTTTGACAAAATGCTGAAGAGCTATTGTGAATTCACATTGGATATTGTTTACAGCACAACAATAAATGCACAATGTGG

CTTTACAAGTTATTTGTGCTGGTTTGCATACAGTTTCCAAGGGCCTCCTAAAACAATTTACGTCTGCTAAGGCCACCAAA

AGAATACAGTGTTCATAAATGCGTGTGTTTGTACCATGCCCGACTGGTTGAGAGTAAAGAAAATGGAGAAGAGGTCTAGT

TGAATACCCGAAACAACTAGCATAAAGATGCTCCCACTTCTTGGTCAGTTTG

>CK192452_bb No definition line found

GAGCAGTCCACCGTTTCCGAGAGGTACTTACAGTCCACTACCACGACCGGAAGCTTGTCCAAACCCATCGAGTCAAGCTC

TCTCACGAGCGTGAAATCTGGCGCATTCACGGGATCCTCATATACCACAGGAACTACAAGTGAATCGCGTACGTCAGCGA

ACGCTCCGACACTTGTCGAGGAGAGATACCGAGAGTCCCATCGGCCACGTACGCTTCACCTCCGGACAATAGAGATCCTT

GGCGTGATGCCCTCGCTCTTTTATGTCTCTTCCTCGTCCTGCTGTTCTTGCTGGCTGCAATGGTCTTCATGTTATGGACT

GTCAATAAAGGTTACCCCAGTCCCTTGCCGCCGACAAGCTCTACGACTACTGCAAGCTCCATGACTACTATCACGACTAC

CAGTGCCACGACTATGCCGACCACTACCACGACTACCACTACCACTACTACTACCACCACTGCGATGACTACTACCACTA

CTACTACCACAACGACTACCACTAGCACCACGTCAACTACTACTACGACGACCAGCACAACTACGACAACAGCGTCCCCT

ACTACTGTCCGACCCATGGGCAAGCACACGCTCTTCTGTGTGTTTGGCCCGTACAAGCAGAACTACCGATGGGTACACAT

CGATGGCCACTGCGACTACGCCTTCATGACGTACTACGTCAACAACCAGGCCACTTTTGTCGACCACAGTGATCCTGACG

TTCAGATTTGGCTGGGAAAGGCGGCCAGTTCGAACTTCACGTCCTTCGGCATCCACGTGCCCCTACGGCAAGTGCTGCGA

GTTGGAAGGCAACCGCTGCTATGAACCACGCGAGGAGCTTACAAGGAGAGCTTAATCTGAGAATGTACTGGACCACCTAT

AAGATATTTCACTACGGCATTTTCAACCTCGAGGTGAAGCCATGGGAGACTGTGAGCATCGACGTGAGGGTCAAGCAAGT

CTTCGAATTTTTCAAGGTTTGCCTTTACTTCGTATTCACCTGCCACTAGCATTGGCAAAGAGAAAGTGGTTGAAAACCTG

GAAAAAAGCATTC

>CK192538_bb No definition line found

TAACAGCAAAAAAATACTTGCATAAAGTTCGAAAATAATCGATCCTGNGTGCCTTCTGTCTTTCTGTCTGTTTGCTTGGA

AGGGATACCGATATTTTCTTACTAAGTCGTTCATAAAAGTATGCGGCTGAAGCGCGTACAATATGAACGAGTTCCCCGCA

GTGTATGCCAAGCAAATCATCCTTTTCAGTGTKKYAAGAATAAAACACATTGTCTTTATTAAGAGCGGAAGGAAAACAGT

GTTTATACACATAACACAAGTGCAATATGTCCCGCAAAACAAATCATGGTCATGTAGAACCACTTAATTTGAGTCCTTGT

TTGGAAATGGTGCCACCAGGCCAGCTGTGACCTATAGGCTGATCCATTGAGCACTACGACTTCACCGGCGAATTAGGAGT

ATGGGCGCTCTAATGAATGCCCCTTGATCTACGCCTTGAGCGACGCGATCGGCGATGTCGCGCAAACTGTGCTGTAGCGC

GCGGTTGACGATGGTGAGTAGGAGTTGTGTTCTGCACAATAGATGGGACTTCCATGGTTGAGTTCTTGACCGGTGCTTCA

GTACTCAGCGTGATAGATTTCGAATTCATGCTCGCTGGATTTATCTTGGTTTCGAGAGTTGAAGACGTGCTCGAAGTCAT

GCGTGAGCTGTTTGGTGGTGTGGCGTCATCTGCTATTTTCGAACTGTTGACAATTACAAAAGCTTGAGACGTTGTAGCGA

AGTTCTTACTTGTTGGTGGCGCATTAGTTTTACAAGGTTCTTTCTTATTTTTTGCAGTACTTGAGTCGTTACTTGAGTTG

CTCCCTGTTTCTTTGGTAATGAGGGGGCTAATAGTACTGGTCAGCTTTGAAGGATTGGTTTTTGTATTGTTTGAGTTGTT

CCCTCTCGGTGTTGTTTGCGTATGATTAAAATTTTTGACAGGCAGTACAATGCGCGGTCCTGCATAGGAGATGTTTGCTG

TC

>CK192539_bb No definition line found

TTTATGTAACAGGCAAAAATAATAAAACGGCAACAATTTCCACTTCCATTCACATGACTACACAATCTCCTGCTGAGCAA

GTGAGCCATAGCACAAGTACTGATGACGTTGTAAACGTTACTGAACATGCCAASACAAATTCAGTGATACRAAATACTCA

GCCTTGTAAAAATTCCAGCTCAGGCGATTCCACTMTTGGAATCGTTAGGACWAACATGAATAACTCAAATGAAAASWCAA

ATGAARARGCTAATATTTCGGCAAATGGAACAAWAATTTCTTCGCATACTACGAACCCGACTTATACTGTAACCAAGCAA

TCAACGGCCAACCCGAACATTTCRGCAAMAYYTTTAATTYCTTCGACACCCGRCACTGCTCARTCTGACACCTCMYTTTC

CTYAAGTTCCATGACGGAAAAYAATTCCAGAGATGCTATCAGCTATTCRACAGGTCAASATTTRYCRCGCTCTGAGCCCA

GCGTAGAACAAMAGAATAGTGTGACCACACCRGCCGATAACTCTTCMCTKGAGCGTAGTTCTACKGTTTCATTTAGYAGT

GGGAAAGCRGGCAACTGCTCAGGAAAGTCGGAAAAGGCAGGCRCGCAACRWACGAAAGCGTCATCTGTYRCTAGCCCAAC

GTCATTAGAGCACAGGTCKACATCYRCATACKCTACACCGCAAGTTGTCAACAGCTCTAGTTTTGCGCCCACCAGTTATT

CCATGAATCTAAACTCGACTGTAGGTACTACTTCGCAAACGTTCGCAGAGAATACAAATATATCGTCAGGGAGTAATGAC

TCCCTGTTTTTGCTGAGCACTTATGCCACGAGCAGTGGCACAATGCCGGAGCGTAATTTCACGCAAAAAGGCCACAACTC

AAGCGTTGCACCATGTATTTCTC

>CK192548_bb No definition line found

CTCGAAAGGCGGAGGGGCGCTCTCGCAGCATCAGAAAAGCGCCTCGTTACATTAGTCCACTTAAATACACCCACCACGGG

ATGATAGCCTACTTTGTTTCACCTAGTGTCTCCGGAACTCATKTGCCGTGAATGTAGTGYCTGCTGAGTGCMCTSTTACA
[truncated: 4,100,002 more chars]
