# Supplementary material for: Reassociation kinetics-based approach for partial genome sequencing of the cattle tick, Rhipicephalus (Boophilus) microplus
Source: BMC Genomics. 2010 Jun 11;11:374. doi: 10.1186/1471-2164-11-374 (PMC2893602; doi:10.1186/1471-2164-11-374)
Supplement: Additional file 5 — Primers for BAC probe synthesis. This Word document contains the sequences to primers used to synthesize probes to screen BAC library for BACs containing sequences from CzEst9, AChE1, and TC7171. [file 1471-2164-11-374-S5.DOC]

**Table**. Sequences of various primers.

Primer Sequence Description Annealing site

FG-328 CATGGCGGTGAAAGCAGCTGT Forward primer for CzEst9 nt 147-167^a^

CzRev AAGAGTGACTTCCAGCGCTC Reverse primer for CzEst9 nt 1759-1778^a^

FG-394 CCGGACTATACGACCAGTACATGG Forward primer for AChE1 nt 733-756^b^

FG-400 GCGAACACGTACTGGTACACC Reverse primer for AChE1 nt 1475-1495^b^

KB-SG-9 AACAAAGGTTCCCCTCGAGT Forward primer for TC7171 bp 739-758^c^

KB-SG-10 CAGATAACCAACACCAGCACA Reverse primer for TC7171 bp 937-957^c^

^a^Numbering based on sequence from AF182283.

^b^Numbering based on sequence from AJ223965.1.

^c^Numbering based on sequence of BmiGI Version 2 contig TC7171.
